# A sodium channel inhibitor ISTX-I with a novel structure provides a new hint at the evolutionary link between two toxin folds

**DOI:** 10.1038/srep29691

**Published:** 2016-07-13

**Authors:** Mingqiang Rong, Jiangxin Liu, Meilin Zhang, Gan Wang, Gang Zhao, Guodong Wang, Yaping Zhang, Kaifeng Hu, Ren Lai

**Affiliations:** 1 Key Laboratory of Animal Models and Human Disease Mechanisms of the Chinese Academy of Sciences & Yunnan Province, Kunming Institute of Zoology, Kunming Yunnan 650223, China; 2 State Key Laboratory of Phytochemistry and Plant Resources in West China, Kunming Institute of Botany, Chinese Academy of Sciences, Kunming, Yunnan 650201, China; 3 Life Sciences College of Nanjing Agricultural University, Nanjing, Jiangsu 210095, China; 4 Yunnan Academy of Grassland and Animal Science, Xiaoshao, Kunming 650212, China; 5 State Key Laboratory of Genetic Resources and Evolution, and Yunnan Laboratory of Molecular Biology of Domestic Animals, Kunming Institute of Zoology, Chinese Academy of Sciences, Kunming, 650223, China

## Abstract

Members of arachnida, such as spiders and scorpions, commonly produce venom with specialized venom glands, paralyzing their prey with neurotoxins that specifically target ion channels. Two well-studied motifs, the disulfide-directed hairpin (DDH) and the inhibitor cystine knot motif (ICK), are both found in scorpion and spider toxins. As arachnids, ticks inject a neurotoxin-containing cocktail from their salivary glands into the host to acquire a blood meal, but peptide toxins acting on ion channels have not been observed in ticks. Here, a new neurotoxin (ISTX-I) that acts on sodium channels was identified from the hard tick *Ixodes scapularis* and characterized. ISTX-I exhibits a potent inhibitory function with an IC_50_ of 1.6 μM for sodium channel Nav1.7 but not other sodium channel subtypes. ISTX-I adopts a novel structural fold and is distinct from the canonical ICK motif. Analysis of the ISTX-I, DDH and ICK motifs reveals that the new ISTX-I motif might be an intermediate scaffold between DDH and ICK, and ISTX-I is a clue to the evolutionary link between the DDH and ICK motifs. These results provide a glimpse into the convergent evolution of neurotoxins from predatory and blood-sucking arthropods.

There are many venomous arthropoda species. Venomous arthropods with specialized venom glands have been found in groups of chelicerata, hexapoda, myriapoda and insects^1^. Most chelicerate arthropods belong to arachnida, which contains many venomous species, such as spiders, scorpions and ticks. Scorpions and spiders are well-studied terrestrial predators, and the major components of their secreted venom are small, disulfide-rich peptides that commonly interfere with voltage-gated ion channels^2^. In total, 922 spider toxins have been described in the venom from 86 species, most of which are Nav channel-specific modulators^3^, and approximately 730 scorpion venom peptides have been isolated from 56 species^4^. Toxins that act on the sodium channel are long-chain peptides. Although ticks are arthropods, few studies have been performed on tick toxins.

Small peptides in which disulfide bonds form the scaffold are referred to as disulfide-rich domains. One approach to classify the disulfide-rich domains is based on the “disulfide signature”, which considers disulfide connectivity and the loop length between cysteine residues^2^. Venoms containing spider toxins predominantly share the inhibitor cystine knot (ICK) fold^5^,^6^,^7^. Peptides with the ICK motif exhibit a characteristic C_1_-C_4_, C_2_-C_5_, C_3_-C_6_ cysteine residue connectivity (it is conventional to label the six cysteine residues involved in the knot as I-VI in order from the N- to C-terminus) and the consensus sequence CX_3-7_CX_3-8_CX_0-7_CX_1-4_CX_4-13_C (X indicates a variable amino acid), demonstrating the conservation of cysteine spacing^8^. It was previously suggested that the disulfide-directed β-hairpin (DDH) fold, which has only two mandatory disulfide bonds with a C_1_-C_4_ and C_2_-C_3_ connectivity pattern, is the evolutionary precursor of the ICK motif 9,10. However, recent studies have proposed that the ICK fold is an evolutionarily simpler ancestral fold of the DDH fold^11^,^12^. Direct evolutionary evidence connecting the DDH and ICK folds is still lacking^13^.

In contrast to predators such as spiders and scorpions, ticks are obligate blood-feeding ectoparasites of a wide range of vertebrates and have drawn extensive attention as pathogen vectors. They successfully obtain blood meals using pharmacological components in their saliva, which contains a large number of peptides and proteins that have been classified as venoms^14^. Many components from tick saliva act as blood coagulation and platelet aggregation inhibitors and as vasodilatory and immunomodulatory compounds^15^,^16^,^17^,^18^,^19^,^20^, although few studies have focused on tick toxins. To date, only one functionally and structurally characterized tick peptide has been reported to be an ion channel effector: the maxiK channel modulator Ra-KLP from the brown ear tick *Rhipicephalus appendiculatus*^21^,^22^.

Biologically active substances from ticks can be considered weapons that interfere with host responses and aid in obtaining blood meals. It has been suggested that there are compounds in tick saliva that prevent the host from feeling itching and pain^23^. In this study, a new neurotoxin peptide (ISTX-I) from *I. scapularis* was identified and characterized as an inhibitor of the Nav1.7 ion channel. Moreover, the structure of ISTX-I is a novel scaffold that provides an evolutionary link between the ICK and DDH motifs.

## Results

### Assignment of the ISTX-I disulfide bonds

**Figure 1:**
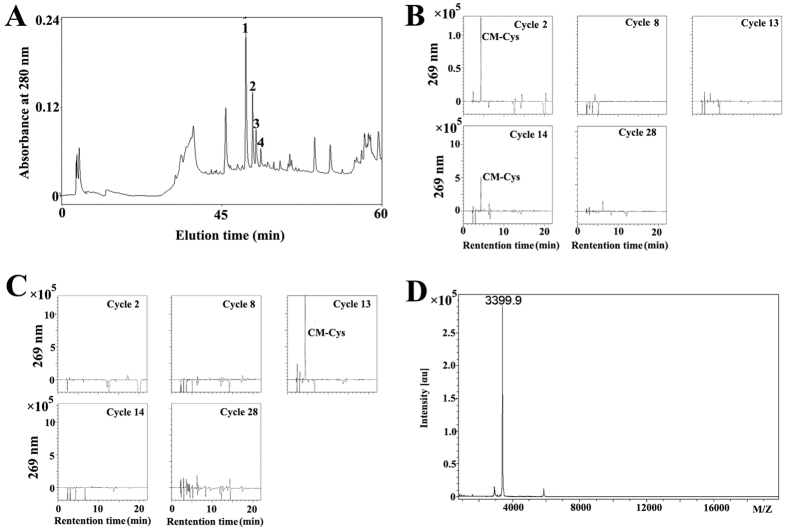
Determination of the disulfide bond pattern in ISTX-I. (**A**) RP-HPLC chromatogram of the ISTX-I reaction mixture after partial reduction by TCEP. (**B**) The sequencing maps for the Edman degradation phenylthiohydantoins observed on cycles 2, 8, 13, 14, and 28 of the two thiol-reduced and carboxyamidomethylated peaks 3. (**C**) The sequencing maps for the Edman degradation phenylthiohydantoins observed oncycles 2, 8, 13, 14 and 28 of the two thiol-reduced and carboxyamidomethylated peaks 2. (**D**) The MALDI-TOF-MS analysis of the two thiol-reduced and carboxyamidomethylated peaks after enzyme digestion.

We identified a new neurotoxin peptide (ISTX-I) from the salivary gland transcripts of *I. scapularis* and expressed it in *Escherichia coli*^24^. Detailed information on the expression, purification and molecular mass determination of recombinant ISTX-I is described in the Supplementary Methods and in Supplementary Fig. S1. To investigate disulfide bonds connectivity, ISTX-I was first partially reduced by TCEP, and the intact peptide and partially reduced intermediates were analyzed and identified as four peaks utilizing MALDI-TOF mass spectrometry (Fig. 1A). Peak 1 represented intact ISTX-I (Supplementary Fig. S2A). The masses of peak 2 and peak 3 were both 2 Da heavier than the intact ISTX-I peptide, indicating the breakdown of one pair of disulfide bonds (Supplementary Fig. S2B,C). Similarly, peak 4 corresponded to a peptide with only one pair of disulfide bonds (Supplementary Fig. S2D). Peak 2 and 3 were collected and alkalized immediately with iodoacetamide followed by further purification using RP-HPLC. A 58 Da increase in molecular mass was detected, suggesting that the free thiols in peaks 2 and 3 had been alkylated (Supplementary Fig. S2E,F).

The sequencing results for peak 3 revealed that the PTH-CM-Cys signals were observed at the 2nd cycle and the 14th cycle (Fig. 1B), verifying that Cys-2 and Cys-14 form one pair of disulfide bonds. In addition, we speculated that Cys-13 and Cys-38 formed a disulfide bond because a PTH-CM-Cys signal was only observed at the 13th cycle, and no signals were observed for Cys-8 and Cys-28 in the sequencing results for peak 2 (Fig. 1C). Consequently, the third cysteine pattern should be Cys-8 and Cys-28. To confirm this speculation, peak 2 was digested into fragments by trypsin, and ZipTip C_18_ was used for desalination of the reaction mixture. If Cys-13 was bound to Cys-38, the peptide would be divided into two fragments with molecular masses of 3398.6 and 1578.4 Da. As shown in Fig. 1D, fragments with a molecular mass of 3398.9 Da were detected, demonstrating that Cys-13 and Cys-38 pair formed a disulfide bond. Consequently, the third disulfide bond should be Cys-8 and Cys-28. Taken together, our results indicate that ISTX-I has the C_1_-C_4_, C_2_-C_5_, C_3_-C_6_ disulfide bond pattern, which is the same pattern as the ICK motif.

### ISTX-I adopts a novel structural fold

To gain insight into the structural features of recombinant ISTX-I, we determined the solution structure of the peptide using 2D ^1^H NMR spectroscopy. In addition to the ^1^H NMR-derived information, the ISTX-I ^13^C resonances were assigned at natural abundances. The chemical shifts of cysteines^13^C Cβ are all between 38.36 and 42.39 ppm, indicating the formation of a disulfide bond^25^. Initial structural calculations were performed without any disulfide bond restraints, yielding results that were consistently in agreement with the Cys-2 and Cys-14, Cys-8 and Cys-28, and Cys-13 and Cys-38 disulfide bond formats. Except for the C-terminal (residues 42–45) residues, the other residues of ISTX-I are well defined. The detailed statistics for the structural ensemble are presented in Table 1.

**Figure 2:**
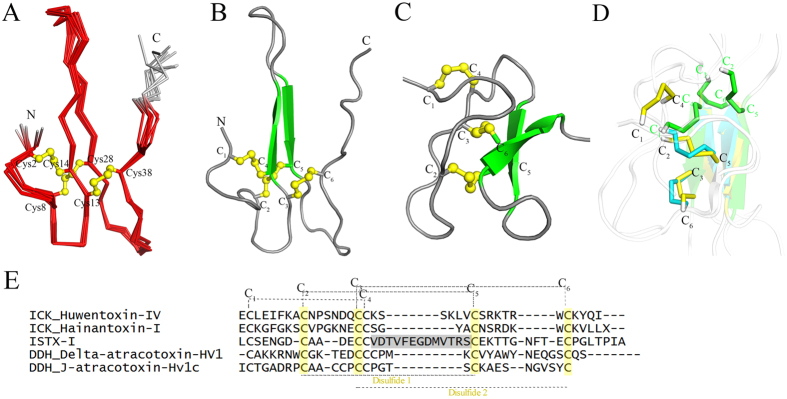
Three-dimensional structure of ISTX-I, with the yellow ball-and-stick format representing the disulfide bond connectivity (C_1_-C_4_, C_2_-C_5_, and C_3_-C_6_). (**A**) Backbone traces from the structural ensemble of ISTX-I with the well-refined region in red and the terminal disorder loops in gray. (**B**) Cartoon diagram of ISTX-I illustrating the locations of the secondary structures. The cysteine residues are labeled with Roman numerals. The β-strands are colored green and others are in gray. (**C**) The structure of Huwentoxin-IV demonstrates the canonical ICK fold (pdb code: 1MB6). (**D**) Superposition of the structures of ISTX-I (green), ICK fold Huwentoxin-IV (yellow) and the DDH fold delta-atracotoxin-HV1(1VTX) (cyan) with the two beta sheets. The disulfide bonds are labeled and color-coded to demonstrate the differences between the three different folds. (**E**) Sequence alignment of the selected ICK, ISTX-I and DDH motifs. The disulfide bonds in ICK motifs are described above the sequence. The positions of the disulfide bonds in DDH motifs are colored yellow below the sequences.

The structure of ISTX-I consists of an anti-parallel β-strand formed by residues Cys-14 to Thr-17 and Thr-25 to Cys-28 and three loop segments including an N-terminal loop, loop 2 connecting the β-strands and a C-terminal loop (Fig. 2A). Our solution structure confirmed the key disulfide bridges described previously, between Cys-2 and Cys-14, Cys-8 and Cys-28, and Cys-13 and Cys-38. The N- and C-terminal loops are primarily stabilized by the formation of disulfide bonds.

Two disulfide bonds in ISTX-I, Cys-8 and Cys-28 (C_2_-C_5_) and Cys-13 and Cys-38 (C_3_-C_6_), form an embedded ring, whereas the Cys-2 and Cys-14 (C_1_-C_4_) disulfide bond is perpendicular to this ring and is positioned on one side (Fig. 2B). This is different from the standard ICK motif, which is an embedded ring formed by two disulfide bonds (C_1_-C_4_ and C_2_-C_5_) with the connecting backbone segment penetrated by the third disulfide bond (C_3_-C_6_) (Fig. 2C). We superimposed the structures of ISTX-I (green), the canonical ICK fold Huwentoxin-IV (1MB6) (yellow) and the DDH fold delta-atracotoxin-HV1 (1VTX) (cyan) with two beta sheets (Fig. 2D). The spatial arrangement of the disulfide bonds in ISTX-I is distinct from those in the ICK and DDH folds, whereas the ICK and DDH folds are very similar.

For peptide toxins from ticks, which are also arachnids, very little structural information is available. There is currently only one structure for the tick neurotoxin holocyclotoxin-1(HT-1), which contains four disulfide bonds and an ICK motif 26. We analyzed whether the ISTX-I structure identified in this study belongs to the ICK family. First of all, ISTX-I possesses an unusually long spacing between C4 and C5 (with 13 residues) which forms two parallel beta strands (Fig. 2E) and differs from the consensus spacing observed in the ICK peptides (generally no more than 6 residues). which constitute part of one beta strand, demonstrating the difference in the primary sequence and the relative orientation of the disulfide bond pairs and the secondary structures between the ISTX-I and ICK fold. Furthermore, we used our structure to search against the KNOTTIN database, which provides standardized data on the knottin structural family^27^. Both the Knoter3D and Knoter1D tools suggested that ISTX-I does not exhibit the inhibitor cysteine knot topology. Remarkably, searching the database using the DALI server^28^ revealed no matches to ISTX-I, indicating no related three-dimensional structures. Therefore, to the best of our knowledge, the structure of ISTX-I can be regarded as a novel fold and has not been observed for any toxin to date.

### ISTX-I specifically inhibits sodium ion channel subtype Nav1.7

**Figure 3:**
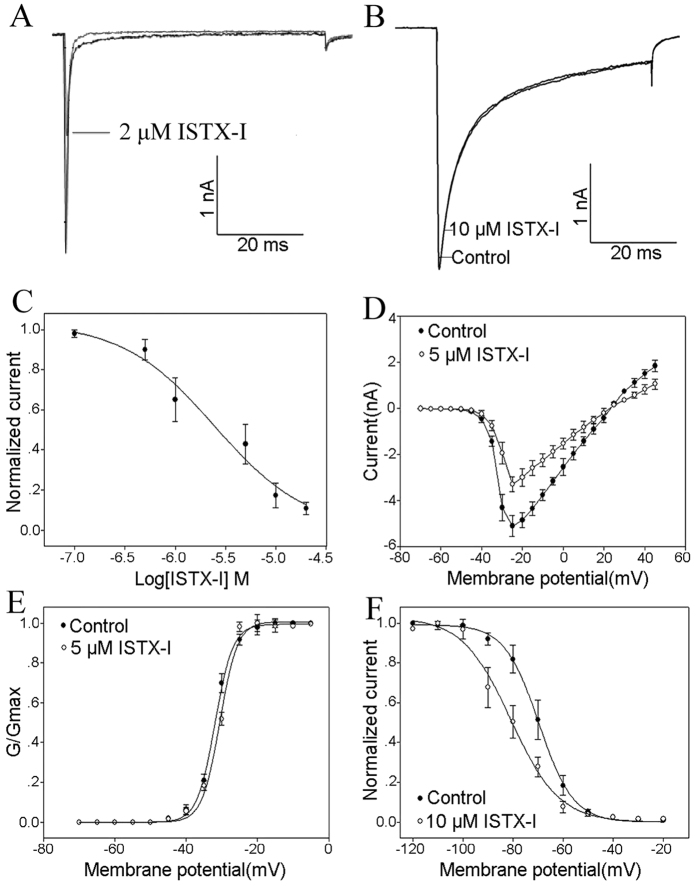
Effects of ISTX-I on voltage-gated ion channels in rat DRG neurons. **(A**) Inhibition of TTX-s Nav channel currents by 1 μM ISTX-I. (**B**) Effects of 10 μM ISTX-I on TTX-r Nav channel currents. (**C**) Dose-dependent inhibition of ISTX-I on the TTX-s Nav channel (n = 5). (**D**) Current-voltage (I-V) relationship for the TTX-s Nav channel currents before (solid circles) and after (open circles) the application of 5 μM ISTX-I (n = 5). (**E**) Conductance–voltage (G-V) relationship of the TTX-s Nav channel before (solid circles) and after (open circles) treatment with 5 μM ISTX-I (n = 5). (**F**) Steady-state inactivation of the TTX-s Nav channel currents before (solid circles) and after(open circles) the application of 10 μM ISTX-I (n = 5).

The effects of ISTX-I on voltage-gated ion channels were investigated in the dorsal root ganglion (DRG). DRG neurons were held at −80 mV for over 4 min to allow adequate equilibration between the micropipette solution and the cell interior, and then current traces were evoked using a 50 ms step depolarization to −10 mV every second. As shown in Fig. 3A, 2 μM ISTX-I reduced approximately 50% of TTX-s sodium channel currents, but 10 μM ISTX-I showed no effect on TTX-r sodium channel currents (Fig. 3B). Fitting the Hill equation to the data points in Fig. 3C yielded an IC_50_ value of 2.5 ± 1.4 μM. In addition, no obvious effects of 20 μM ISTX-I on potassium and calcium channels were detected.

The current–voltage (I-V) curve for TTX-s sodium channels is illustrated in Fig. 3D, in which the initial activated voltage and reversal potential are approximately −40 and +20 mV. No changes to I-V curve and conductance-voltage relationship were induced (Fig. 3D,E). However, 10 μM ISTX-I significantly shifted the steady-state inactivation curve of the TTX-s sodium channels by approximately 11 mV in a hyperpolarized direction (Fig. 3F).

**Figure 4:**
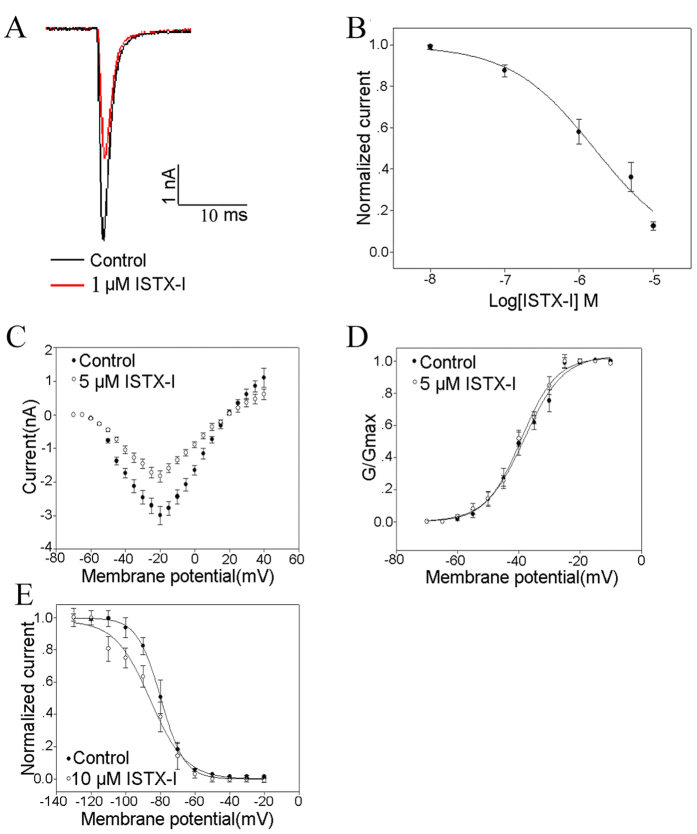
Effects of ISTX-I expressed in HEK293 cells. Current traces were evoked with a 50ms step depolarization to −10 mV from a holding potential of −80 mV every 5s. (**A**) Inhibition of hNav1.7 by 1 μM ISTX-I. (**B**) Dose-dependent inhibition of the hNav1.7 Nav channel by ISTX-I. Notably, 5 μM ISTX-I had no effect on the current-voltage (I-V) relationships (**C**) or on the conductance–voltage (G-V) relationships (**D**) of hNav1.7. (**E**) Steady-state inactivation of the hNav1.7 channel before (solid circles) and after (open circles) the application of 10 μM ISTX-I (n = 5).

To further characterize ISTX-I selectivity, different human(h) sodium channel subtypes were transfected into HEK293T cells. ISTX-I showed no effect on the hNav1.1, hNav1.2, hNav1.3, hNav1.4, hNav1.5 subtypes at a concentration of 10 μM (Supplementary Fig. S3). 10 μM ISTX-I only inhibited 10% currents of Nav1.6 (Supplementary Fig. S3F). In contrast, 1μM ISTX-I significantly depressed hNav1.7 channel currents (Fig. 4A), with an IC_50_ value of 1.6 ± 0.1 μM (Fig. 4B).

The effects of ISTX-I on the activation and inactivation kinetics of hNav1.7 were also analyzed. The current-voltage relationships were determined for Nav1.7 channels using step depolarization ranging from −80 to +40 mV from a holding potential of −80 mV. The results revealed that 5 μM ISTX-I did not shift the current-voltage and the conductance-voltage curves (Fig. 4C,D). However, 10 μM ISTX-I significantly shifted the steady-state inactivation curve of the hNav1.7 channels by approximately −10 mV (Fig. 4E). These results strongly suggest that ISTX-I is a sodium channel inhibitor that has specific activity on Nav1.7 subtypes.

### Phylogenetic analysis of sodium channel toxins

**Figure 5:**
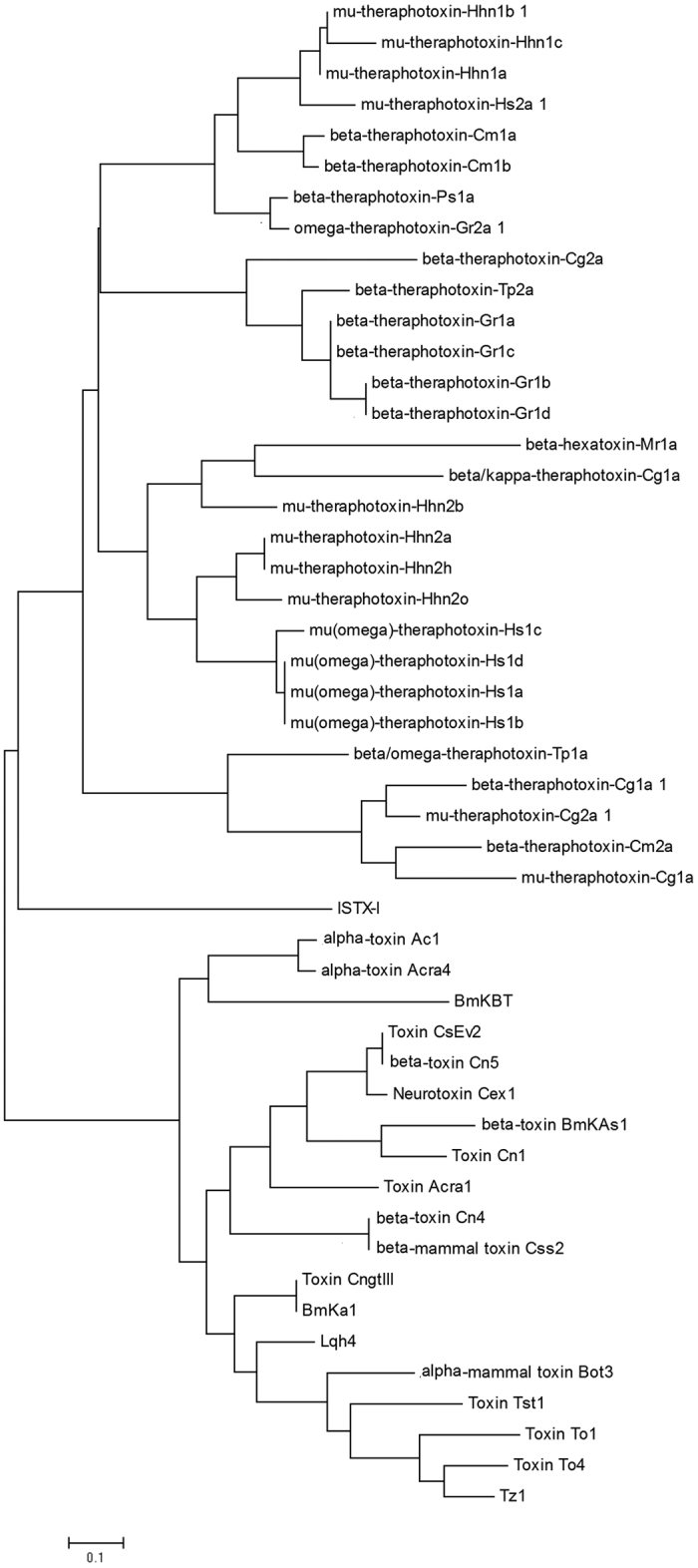
Phylogenetic analysis of ISTX-I with spider and scorpion toxins. Phylogenetic dendrogram obtained with a neighbor-joining analysis based on the proportion difference (p-distance) of aligned amino acids of the full-length peptide sequences. All of the spider and scorpion toxin sequences are from UniProt.

A larger number of sodium channel toxins have been identified from spiders and scorpions, and peptide toxins that act on sodium channels from spiders and scorpions were selected to test the evolutionary potential of sodium channel toxins in arachnida. The sequences of these peptides were clustered to construct a phylogenetic tree separately, using ClustalW. Evolutionary analysis was based on the multi-sequence alignments of mature peptides with MEGA software. Notably, two major groups were observed in the phylogenetic tree. One group contained spider toxins, whereas the other group consisted of scorpion toxins (Fig. 5). ISTX-I belongs to the spider toxin group and is most closely related to mu-theraphotoxin-Cg2a. The two toxins exhibit the same cysteine pattern (C-C-CC-C-C), but a large difference was seen between the fourth and fifth cysteines. There are four residues between the fourth and fifth cysteines in mu-theraphotoxin-Cg2a but a long chain of approximately 13 residues in ISTX-I. Sodium channel inhibitors from the scorpion toxins group were generally long-chain peptides containing 8 cysteines, whereas most spider toxins contain 6 cysteines, indicating that ISTX-I, with 6 cysteines, is more closely related to spider toxins. The phylogenetic tree further supports that ISTX-I is closely related to spider toxins.

## Discussion

It is well established that spiders and scorpions use neurotoxins to prey on and capture food. However, few toxins from tick saliva have been studied. ISTX-I is the first neurotoxin acting on a sodium channel identified from *I. scapularis*. ISTX-I specifically inhibited TTX-s sodium channels and not TTX-r sodium channels in rat DRG neurons. Furthermore, ISTX-I was demonstrated to be a subtype-selective inhibitor of Nav1.7. Ticks normally use their mouthparts to penetrate the host skin and probe for blood. To obtain enough blood, ticks must adopt a strategy to prevent themselves from being discovered. The Nav1.7 ion channel has been demonstrated to be a critical mediator of the upstroke of action potentials^29^. Subtype-selective Nav1.7 blockers potentially inhibit electrical signaling in the nervous system. As a new selective inhibitor of the Nav1.7 channel, ISTX-I might modulate Nav1.7 channel function and prevent signal transmission caused by tick penetration and the blood taken, allowing the tick to avoid discovery.

ISTX-I inhibits sodium channels without any shifts of the I-V and G-V curves. The mechanism is the same as that of spider toxins such us mu-theraphotoxin-Cg2a. In contrast, scorpion toxin shifts the inactivation or activation of the sodium channel. The mechanism by which ISTX-I acts on the sodium channel is closer to the mechanism of spider toxins. Moreover, the phylogenetic tree analysis also demonstrated the close relationship between ISTX-I and the spider toxin mu-theraphotoxin-Cg2a. Taken together, the evolution of ISTX-I is close to that of spider toxins, which might suggest that ticks are more closely related to spiders than to scorpions.

In toxins, two common folds are the ICK motif and the two-disulfide bond DDH motif. The evolutionary relationship between the two motifs remains controversial^8^,^10^,^11^, and our structure might provide some interesting clues. If we compare the structures of ISTX-I and delta-atracotoxin-HV1 (pdb: 1VTX), the spatial arrangements of the disulfide bonds show certain similarities. The cross formed by the two disulfide bonds within the β-sheets in ISTX-I (C_1_-C_4_ and C_2_-C_5_) is similar to the cross formed by the two disulfide bonds (C_2_-C_5_ and C_3_-C_6_) in the DDH fold (Fig. 2D), indicating some conservation between the ISTX-I fold and the DDH fold. Comparing the short spacing in DDH fold which form half of the beta sheet (1VTX), the long spacing between C4 and C5 (Fig. 2E) which forms two parallel of beta sheet make it possible to rearrange the orientation of the disulfide-bonds. During the evolution of peptide primary sequence, shorter peptide is more stable. Losing the loop and reconstitution of the similar DDH fold are evolutionary preferred. With the extra pair of disulfide bond, the motif may form as ICK fold. Taking the structural conservation and divergence together^2^, ISTX-I fold may represent a possible selective gain of scaffold and function as an intermediate state linking the ICK fold and DDH fold during evolution, resulting in structural diversity within the cysteine-rich toxin family.

In conclusion, a selective inhibitor of Nav1.7 was identified from the saliva of *I. scapularis.* Notably, ISTX-I shares the same disulfide bonding patterns as spider toxins but exhibits a completely different fold due to the difference in loop spacing between cysteine residues. This novel peptide toxin will improve our understanding of tick-host interactions and the evolutionary relationship between DDH and ICK toxins.

## Methods

### Ethics statement

All of the methods and experimental protocols used in this study were reviewed and approved by the Institutional Review Board of the Kunming Institute of Zoology and Kunming Institute of Botany, Chinese Academy of Sciences. The experiments were carried out in accordance with the approved guidelines and regulations.

### DRG cell preparation

Rat DRG cells were acutely dissociated from adult Sprague-Dawley rats^30^. Briefly, adult rats of either sex with body weights of approximately 200 g were killed by decapitation. The dorsal root ganglia were isolated quickly from the spinal cord and then transferred into Dulbecco’s modified Eagle’s medium (DMEM) containing trypsin I (0.15 mg/mL) and collagenase II (0.3 mg/mL) for incubation with an oscillation frequency of 200 rpm at 37 °C for 20 min. The trypsin inhibitor II-S (1 mg/mL) was added to terminate the reaction. The isolated DRG cells were split into 35 mm culture dishes with DMEM solution containing 10% fetal bovine serum (FBS) and were incubated in a CO_2_ incubator (5% CO_2_, 95% air, 37 °C) for 1–4 h before the patch-clamp experiment.

### Cell culture and transient transfection

HEK293T (human embryonic kidney) cells were grown in DMEM containing 10% heat-inactivated FBS at 37 °C in a 5% CO_2_ incubator. The cDNA genes encoding human (h)Nav1.1, (h)Nav1.2, (h)Nav1.3, (h)Nav1.4, (h)Nav1.5, (h)Nav1.6 and (h)Nav1.7 were transiently transfected into HEK293T cells for electrophysiology using Lipofectamine-2000 (Invitrogen) in serum-reduced medium (Opti-MEM, Invitrogen) following the manufacturer’s instructions. The auxiliary subunits hβ1 and enhanced green florescent protein plasmids were cotransfected into HEK293T cells to increase the current density and efficiently select cells with green florescent protein for whole-cell patch-clamp recordings^31^. After 12 h, the HEK293T cells were used for electrophysiological analysis.

### Whole-cell patch-clamp recordings and data analysis

DRG neurons with a large diameter (>35 mm) and those with a relatively small diameter (<20 mm) were chosen by measuring TTX-s and TTX-r currents, respectively. Meanwhile, TTX (final concentration at 100 nM) was used to separate TTX-r sodium currents from TTX-s sodium currents. Suction pipettes (2.0–4.0 MΩ) were made of borosilicate glass capillary tubes with two-step pulling on a vertical micropipette puller (PC-10, Narishige). Potassium, calcium, and sodium currents were recorded in experimental cells using whole-cell patch-clamp recordings at room temperature, which were performed with an HEK EPC10 patch-clamp amplifier. The P/4 protocol was used to subtract linear capacitive and leakage currents. Experimental data were acquired and analyzed using the programs patch master and Sigmaplot 9.0 (Sigma, USA). All of the data points represent the mean ± SE (*n* = the number of separate experimental cells examined). Dose-response curves were fitted using the following Hill logistic equation: y = 1 − (1 − f_max_)/[1 + ([Tx]/IC_50_)^n^] in which n is an empirical Hill coefficient and f_max_ is the fraction of current resistant to inhibition at high toxin (Tx) concentrations.

### Assignment of ISTX-I disulfide bonds

ISTX-I (0.1 mg) was modified in 10 μL citrate buffer (0.1 M, pH 3.0) containing 6 M guanidine-HCl for 30 min at room temperature. Partial reduction of ISTX-I disulfide bonds was performed by adding 10 μl 0.1 M Tris (2-carboxyethyl) phosphine (TCEP) at 40 °C for 10 min at pH 3.0, and the intermediates were separated via a C_18_ reverse-phase HPLC column with linear gradient elution (20–35% acetonitrile in 30 min). The partial reduction intermediates were collected, and their masses were determined by MALDI-TOF mass spectrometry. Appropriate intermediates containing free thiols were lyophilized and then alkalized by adding 100 μL 0.5 M iodoacetamide (pH 8.3). The alkalized peptides were desalted by reverse-phase HPLC and then run through an Applied Biosystems Model 491 gas-phase sequencer. Edman degradation was performed with a normal automatic cycle program. The protease digestion strategy was also used to identify disulfide bonds. The intermediates after alkylated modification were dissolved in 10 μL NH_4_HCO_3_ (25 mM, pH 7.8) buffer containing trypsin gold (Sigma). The mixture was incubated at 37 °C for 16 h. After desalination in accordance with the ZipTip (ZipTip C_18_) pipette tip protocol, the masses of the reaction components were analyzed using MALDI-TOF mass spectrometry.

### NMR spectroscopy and structure calculations

The lyophilized peptides were dissolved in NMR buffer with 10 mM MES, 20 mM NaCl in 90% H_2_O and 10% D_2_O (pH 5.7). NMR experiments were conducted at 298 k on a Bruker Avance 800 MHz spectrometer (QCI-cryoprobe). Individual residue spin systems were assigned using total correlation spectroscopy with a mixing time of 80 ms^32^. The recording data points for t1 × t2 were 512 × 4096. Sequential residue assignments were achieved from NOESY experiments collected with mixing times of 100, 200, 300 and 400 ms^33^. The data points for t1 × t2 were 2048 × 4096 and 1024 × 4096, respectively. In addition, ^13^C HSQC and ^1^H-^13^C TOCSY-HSQC spectra were collected to aid in sequential assignment and elucidate the cysteine oxidation states^34^. The recording data points for t1 × t2 were 1500 × 4096 for the ^1^H-^13^C TOCSY-HSQC experiments.

Hydrogen-deuterium (H/D) exchange experiments were performed to observe the hydrogen bonds within the peptide. After dissolving the lyophilized peptide in D_2_O, a set of 1D spectra were collected, including time points at 10 min, 20 min, 30 min, 1 h, 2 h, 4 h, 8 h and 24 h. The TOCSY spectrum was also collected after 8 h of H/D exchange. NMR data were processed by NMRPIPE and analyzed with SPARKY^35^,^36^.

NOE cross peaks were analyzed with manual assignments and converted into distance constraints. Structures were calculated using the program xplor-NIH^37^. The ensemble was analyzed with the programs PROCHECK and PyMOL.

### Sequence and phylogenetic analysis

The sequences of scorpion and spider toxins were obtained from UniProt and then aligned using ClustalW (Version 1.82). The alignment results were used to construct a neighbor-joining (NJ) tree on the basis of the p-distance substitution model with pairwise deletion of gaps (MEGA 4.1).

## Additional Information

**How to cite this article**: Rong, M. *et al*. A sodium channel inhibitor ISTX-I with novel structure provides a new hint at the evolutionary link between two toxin folds. *Sci. Rep.* **6**, 29691; doi: 10.1038/srep29691 (2016).

## Supplementary Material

Supplementary Information

## Figures and Tables

**Table 1:** Structural Statistics forthe ISTX-I Structure^a^.

Experimental constraints	
NOE distance constraints	468
Intra-residue (i–j = 0)	161
Sequential (|i–j| = 1)	207
Medium range (|i– j| ≤4)	28
Long range (|i–j| >5)	72
Hydrogen bonds	6
Energies (kcal/mol)	
Bonds	7.18 ± 0.51
Angles	28.24 ± 1.81
Improper	5.00 ± 0.22
Van del Waals (repel)	18.41 ± 1.17
NOE	19.94 ± 2.24
r.m.s. deviations from idealized	
Bonds (Å)	0.003 ± 0.000
Angles (deg)	0.429 ± 0.010
Improper (deg)	0.327 ± 0.007
NOE	0.020 ± 0.001
Ramachandran Plot^b^	
Most favored regions	78.9%
Additional allowed regions	21.1%
Generously allowed regions	0%
Disallowed regions	0%
Mean pairwise r.m.s.d. (secondary region between ISTX-I 2-41) (Å)	
Backbone	0.66 ± 0.16
Heavy Atoms	1.10 ± 0.16

^a^None of these structures exhibit distance violations >0.5 Å or dihedral angle violations >5°.

^b^MolProbity was used to assess the quality of the structures.
