# Supplementary material for: A sodium channel inhibitor ISTX-I with a novel structure provides a new hint at the evolutionary link between two toxin folds
Source: Sci Rep. 2016 Jul 13;6:29691. doi: 10.1038/srep29691 (PMC4942781; doi:10.1038/srep29691)
Supplement: Supplementary Information [file srep29691-s1.pdf]

A sodium channel inhibitor ISTX-I with novel structure provides a new hint at the evolutionary link between two toxin folds

Mingqiang Rong[1 §], Jiangxin Liu[2 §], Meilin Zhang[1 §], Gan Wang[3], Gang Zhao[4], Guodong Wang[5],Yaping Zhang[5], Kaifeng Hu[2*], and Ren Lai[1,3*]

Supplementary methods

Recombinant expression and purification

The DNA fragment encoding ISTX-I was PCR-amplified and cloned into a modified pET-32a (+) vector. The construct yields a Trx-His_6-tagged protein with a TEV site between the tag and the ISTX-I. The Trx-His_6-tagged ISTX-I was overexpressed in *Escherichia coli* BL21(DE3) cells. Cultures were grown in LB medium at 37 ℃ until the O.D. 600 reached 0.6. Cells were induced with 0.6 mM isopropyl-thiogalactopyranoside (IPTG) at 37 ℃ for 4 h and harvested by centrifugation. Recombinant ISTX-I was isolated from the lysate via Ni-NTA purification and digested with TEV protease at room temperature overnight to remove the tag. The ISTX-I protein was further purified with a Sephadex G-50 (Superfine, Amersham Biosciences, 2.6×100 cm) gel filtration column, an AKTA Mono Q (1ml volume, Amersham Biosciences) anion exchange column and a C18 reverse phase high performance liquid chromatography column (Hypersil BDS C18, 30×0.46 cm). Finally, the purified ISTX-I was examined by MALDI-TOF mass spectrometry to confirm its purity and molecular mass.

Mass spectrometry

Matrix-assisted laser desorption ionization time-of-flight (MALDI-TOF) mass spectrometry (Bruker Proflex III) was used to detect the purity and molecular mass of the ISTX-I and its intermediates. MALDI-TOF was performed on a Finnegan Lasermat, and the absorbing matrix used was α-acyano-4-hydroxy-cinnamic acid. All masses reported are for the monoisotopic $M+H^+$ ions.

Electrophysiological studies.

Currents were recorded from experimental cells using whole-cell patch clamptechnique at room temperature (22–25 ℃). The patch pipettes were fabricated from borosilicate glass tubing using a two-stage vertical microelectrode puller (PC-10, Narishige, Japan). After establishing the whole-cell recording configuration, the resting potential was held at −80 mV for at least 5 min to allow adequate equilibration between the micropipette solution and the cell interior. The micropipette solution of sodium currents for dorsal root ganglion neurons contained (in mM): 105 CsF, 35 NaCl, 10 HEPES, 10 EGTA (pH 7.4). The external solution contained (in mM): 50 NaCl, 100 TEA.Cl, 2 KCl, 1.5 $CaCl_2$, 10 HEPES, 1 $MgCl_2$, and 5 glucose (pH 7.4). K^+ currents were recorded using an internal solution containing the followings (in mM): KCl 140, $MgCl_2$ 2.5, HEPES 10, and EGTA 11 (pH 7.2). The external bathing solution contained the followings (in mM): NaCl 150, KCl 5, $CaCl_2$ 25, $MgCl_2$ 12, HEPES 10, and D-glucose 10 (pH 7.2). Calcium channel currents were measured using Ba^{2+} as the charge carrier. The external solution contained (in mM): 160 TEA-Cl, 10 HEPES, 2 $BaCl_2$, 10 glucoses, adjusted to pH 7.4 with TEA-OH. The internal solution contained (in mM): 120 CsCl, 5 Mg-ATP, 0.4 Na_2-GTP, 10 EGTA, 20 HEPES-CsOH, and adjusted to pH 7.2 with CsOH (Lewis et al., 2000). Ionic currents were filtered at 3 kHz and sampled at 20 kHz on EPC-9 patch clamp amplifier (HEKA, Lambrecht, Germany).

Supplementary Figure legends

Figure S1. Expression, purification and molecular mass determination of recombinant ISTX-I. A: Separation of ISTX-I by Sephadex G-50 gel filtration. The absorbance of the elution was monitored at 280 nm. Inset: Expression of ISTX-I fusion protein in *E.coli* Rosetta–gami (DE3) and analysis by SDS-PAGE (12%). M, protein molecular weight marker; lanes1&2, ISTX-I/pET-32a(+) uninduced and induced cells, respectively. B: The protein peak (indicated by an arrow) from Sephadex G-50 gel filtration was subjected to AKTA Resource Q anion exchange chromatography. C: The elution peak(marked by arrow) after Resource Q anion

exchange chromatography was further purified by C_{18} RP-HPLC. The elution was performed with a gradient of acetonitrile in 0.1% (v/v) trifluoroacetic acid in water at a flow rate of 0.7 ml/min. D: MALDI-TOF mass spectrometry analysis of recombinant ISTX-I after the TEV digestion and purification.

Figure S2. Mass spectrometry of partially reduced intermediates of ISTX-I.A: Molecular mass of intact ISTX-I (peak 1). Molecular mass of peak 2 (B) peak3（C）and peak 4(D). Molecular mass of alkylated peak 2 (E) and 3 (F).

Figure S3. Effects of ISTX-I on subtypes of sodium channel expressed in HEK293 cells. Cells were held at -80 mV for over 4 min and then the current traces were evoked using a 50 ms step depolarization to -10 mV every second. No effect on the hNav1.1 (A), hNav1.2 (B), hNav1.3 (C), hNav1.4 (D), hNav1.5 (E) and hNav1.6 (F), (G)hNav1.8.

Figure S4. **Effects of ISTX-I on** Kv currents and Cav currents of DRG neurons. For record Kv currents, cell were evoked by a 500 ms depolarized potential of +10 mV from a holding potential same as recording Nav currents. For record Cav currents, cells were activated by a 150 ms step depolarization to +10 mV from a holding potential of −90 mV. No effects were detected on potassium channel (A) and calcium channel (B).

Fig S1

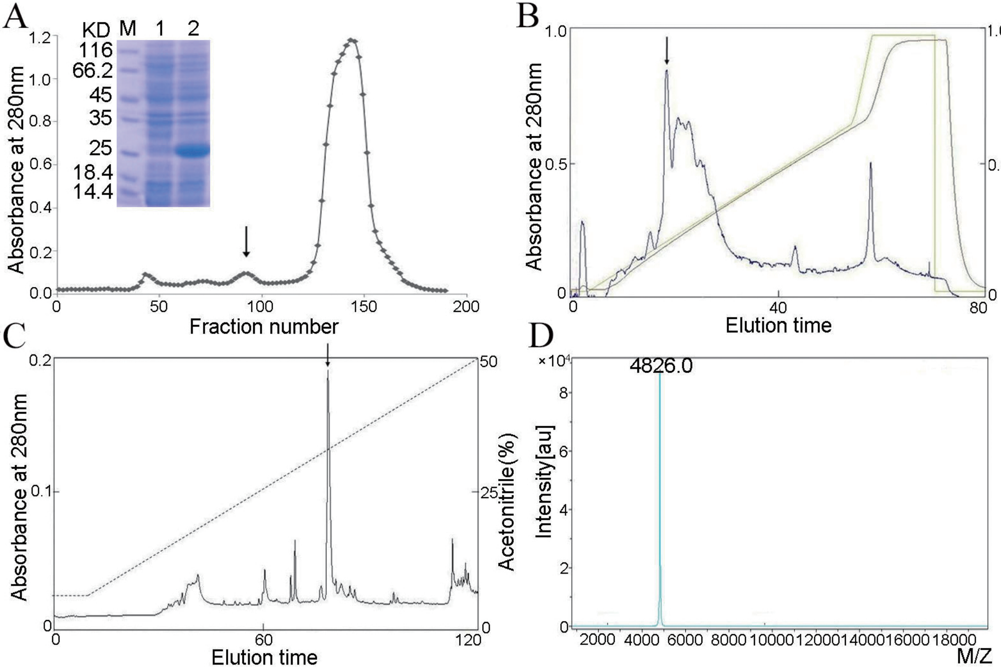

A
KD M 1 2
116
66.2
45
35
25
18.4
14.4
Absorbance at 280nm
Fraction number
B
Absorbance at 280nm
NaCl(M)
Elution time
C
Absorbance at 280nm
Acetonitrile(%)
Elution time
D
×10⁴
4826.0
Intensity[au]
M/Z

Fig S2

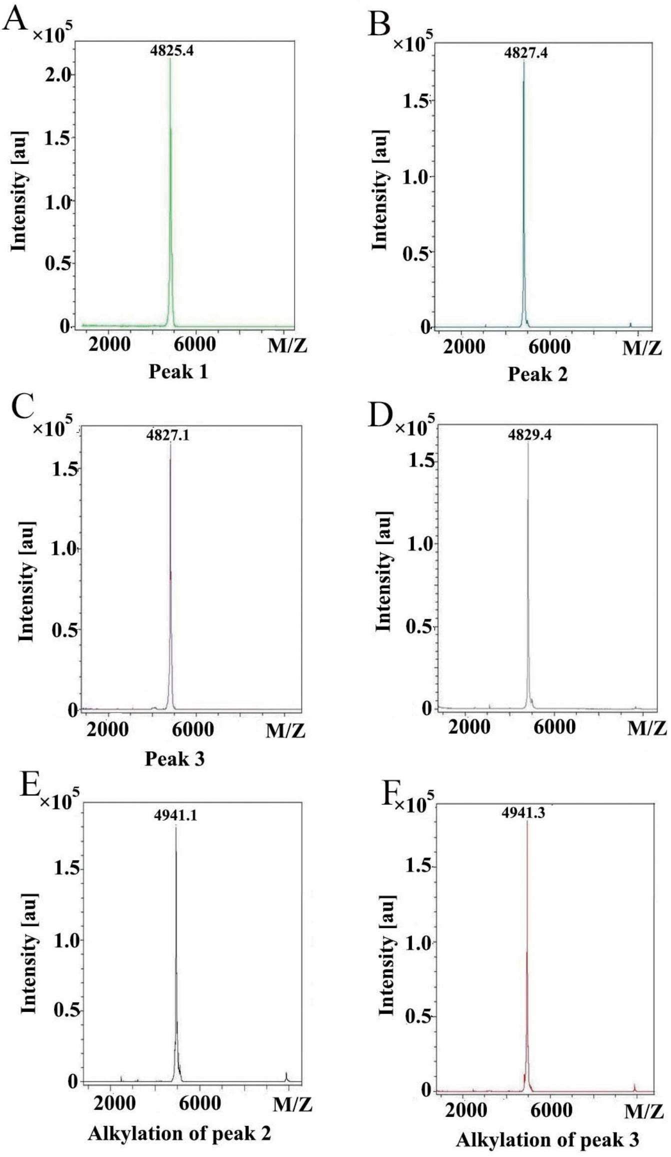
A
×10^5
4825.4
2.0
1.5
1.0
0.5
0
Intensity [au]
2000
6000
M/Z
Peak 1
B
×10^5
4827.4
1.5
1.0
0.5
0
Intensity [au]
2000
6000
M/Z
Peak 2
C
×10^5
4827.1
1.5
1.0
0.5
0
Intensity [au]
2000
6000
M/Z
Peak 3
D
×10^5
4829.4
1.5
1.0
0.5
0
Intensity [au]
2000
6000
M/Z
E
×10^5
4941.1
1.5
1.0
0.5
0
Intensity [au]
2000
6000
M/Z
Alkylation of peak 2
F
×10^5
4941.3
1.5
1.0
0.5
0
Intensity [au]
2000
6000
M/Z
Alkylation of peak 3

Fig S3

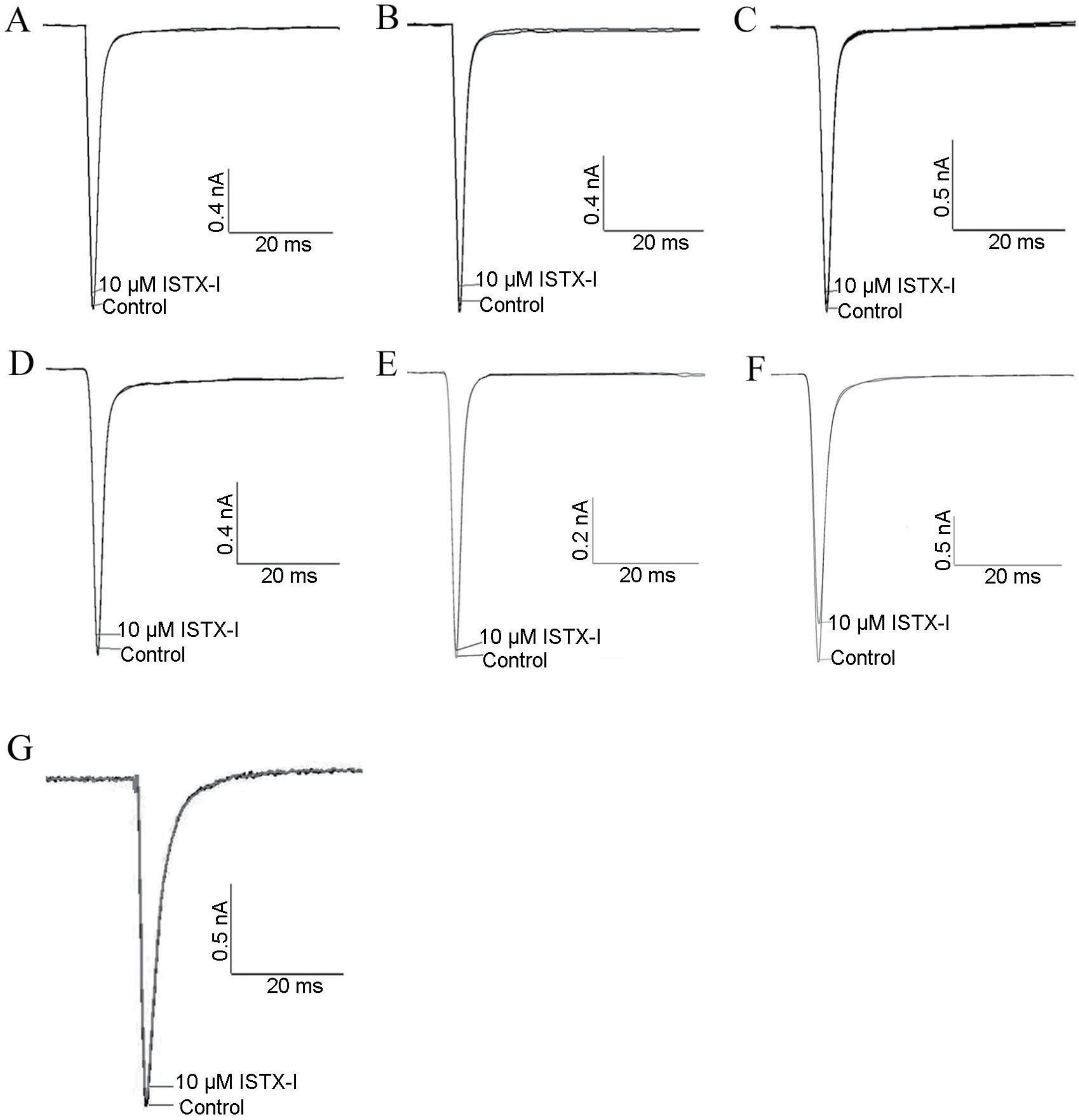

A
0.4 nA
20 ms
10 μM ISTX-I
Control
B
0.4 nA
20 ms
10 μM ISTX-I
Control
C
0.5 nA
20 ms
10 μM ISTX-I
Control
D
0.4 nA
20 ms
10 μM ISTX-I
Control
E
0.2 nA
20 ms
10 μM ISTX-I
Control
F
0.5 nA
20 ms
10 μM ISTX-I
Control
G
0.5 nA
20 ms
10 μM ISTX-I
Control

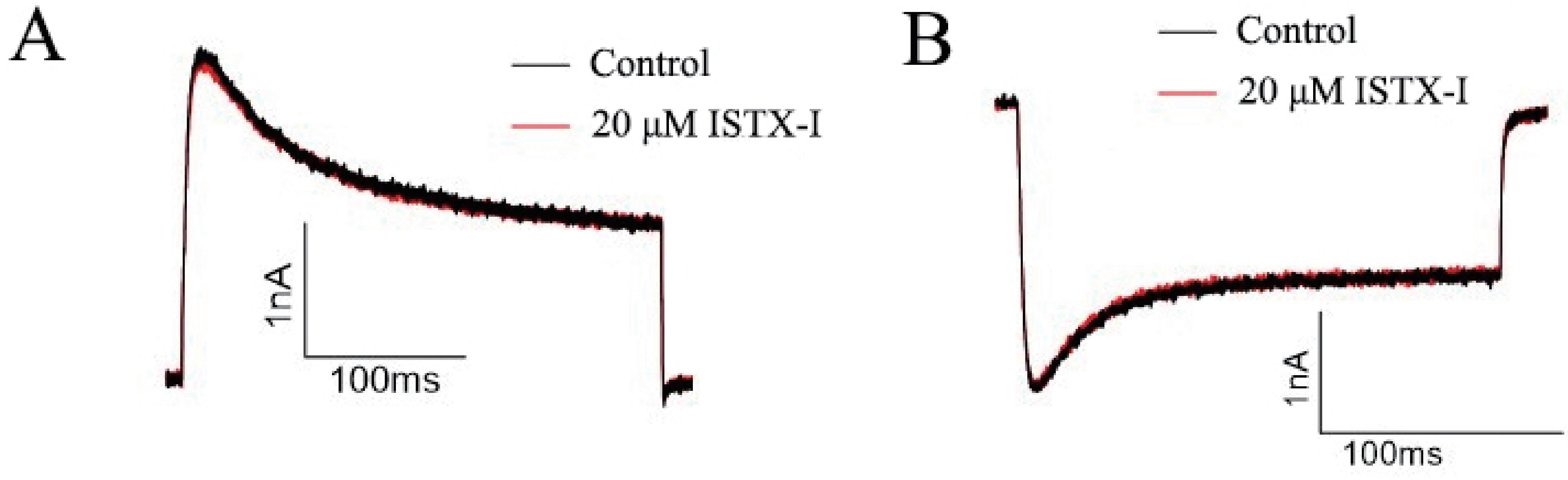
A
Control
20 μM ISTX-I
1nA
100ms
B
Control
20 μM ISTX-I
1nA
100ms

Supplementary table 1

Name of toxin	Sequence
mu-theraphotoxin-Hhn1b	ECLGFGKGCNPSNDQCCKSSNLVCSRKHRWCKYEI
mu-theraphotoxin-Hhn1c	ECLGFGKGCNPSNDQCCKSSNLVCSRKHRRCKYEIGK
mu-theraphotoxin-Hhn1a	ECLGFGKGCNPSNDQCCKSANLVCSRKHRWCKYEI
mu-theraphotoxin-Hs2a	ACNPSNDQCCKSSKLVCSRKTRWCKYQIGK
beta-theraphotoxin-Cm1a	DCLGWFKSCDPKNDKCCKNYTCSRRDRWCKYDL
beta-theraphotoxin-Cm1b	DCLGWFKSCDPKNDKCCKNYTCSRRDRWCKYYL
beta-theraphotoxin-Ps1a	DCLGFLWKCNPSNDKCCRPNLVCSRKDKWCKYQI
omega-theraphotoxin-Gr2a	DCLGFMRKCIPDNDKCCRPNLVCSRTHKWCKYVF
beta-theraphotoxin-Cg2a	YCQKWMWTCDSKRACCEGLRCKLWCRKIIG
beta-theraphotoxin-Tp2a	YCQKWMWTCDSERKCCEGMVCRLWCKKKLW
beta-theraphotoxin-Gr1a	YCQKWMWTCDSKRKCCEDMVCQLWCKKRL
beta-theraphotoxin-Gr1c	YCQKWMWTCDSKRKCCEDMVCQLWCKKRLG
beta-theraphotoxin-Gr1d	YCQKWLWTCDSERKCCEDMVCRLWCKKRLG
beta-theraphotoxin-Gr1b	YCQKWLWTCDSERKCCEDMVCRLWCKKRL
beta-hexatoxin-Mr1a	GCKLTFWKCKNKKECCGWNACALGICMPR
beta/kappa-theraphotoxin-Cg1a	ECGGFWWKCGRGKPPCCKGYACSKTWGWCAVEAP
mu-theraphotoxin-Hhn2b	ECKGFGKSCVPGKNECCSGYACNSRDKWCKVLL
mu-theraphotoxin-Hhn2a	GCKGFGDSCTPGKNECCPNYACSSKHKWCKVYL
mu-theraphotoxin-Hhn2h	GCKGFGDSCTPGKNECCPNYACSSKHKWCKVYSGK
mu-theraphotoxin-Hhn2o	GCKGFGDSCTPGKNECCPNHACSNKHKWCKAYLGK
mu(omega)-theraphotoxin-Hs1c	ACKGVFDACTPGKNECCSNRVCSDKHKWCKWKL
mu(omega)-theraphotoxin-Hs1b	ACKGVFGACTPGKNECCPNRVCSDKHKWCKWKL
mu(omega)-theraphotoxin-Hs1d	ACKGVFDACTPGKNECCPNRVCSDEHKWCKWKL
mu(omega)-theraphotoxin-Hs1a	ACKGVFDACTPGKNECCPNRVCSDKHKWCKWKL
beta/omega-theraphotoxin-Tp1a	ECRYWLGGCSAGQTCCKHLVCSRRHGWCVWDGTFS
mu-theraphotoxin-Cg1a	ACREWLGGCSKDADCCAHLECRKKWPYHCVWDWTVRK
mu-theraphotoxin-Cg2a	ECTKFLGGCSEDSECCPHLGCKDVLYYCAWDGTFGK
beta-theraphotoxin-Cm2a	GVDKEGCRKLLGGCTIDDDCCPHLGCNKKYWHCGWDGTF
beta-theraphotoxin-Cg1a	ECRKMFGGCSVDSDCCAHLGCKPTLKYCAWDGTFGK
ISTX-I	LCSENGDCAADECCVDTVFEGDMVTRSCEKTTGNFTECPGLTPIA
Alpha-toxin Ac1	DGYIVYPNNCVYHCIPACDGLCKKNGGTSGSCSFLIGSGIACWCKDLP DNVPIKDPSQKCT
alpha-toxin Acra4	VRDGYIVDDKNCVYHCIPPCDGLCKKNGGKSGSCSFLVPSGLACWCK ALPDNVPIKDPSYKCHK
BmKBT	KKSGYPTDHEGCKNWCVLNHSCGILCEGYGGSGYCYFWKLACWCD DIHNWVPTWSRATNKCRAK
Toxin CsEv2	EGYLVNKSTGCKYGCLKLGENEGCDKECKAKNQGGSYGYCYAFAC WCEGLPESTPTYPLPNKSCS
Beta-toxin Cn5	EGYLVNKSTGCKYGCLLLGKNEGCDKECKAKNQGGSYGYCYAFGC WCEGLPESTPTYPLPNKSCS
Neurotoxin Cex1	EGCDKECKAPNQGGGYGYCHAFACWCENLPESTPTYPIPGKSC

Beta-toxin BmKAs1	DNGYLLNKYTGCKIWCVINNESCNSECKLRRGNYGYCYFWKLACYCEGAPKSELWAYETNKCNGKM
Toxin Cn1	DGYLVDAKGCKKNCYKLGKNDYCNRECRMKHRGGSYGYCYGFGCYCEGLSDSTPTWPLPNKTCSGK
Toxin Acra1	ADVPGNYPLDSSGNKYPCTVLGDNQSCIDVCKKHGVKYGYCYSFKCWCEFLEDKNVSI
Beta-toxin Cn4	EGYLVNSYTGCKYECFKLGDNDYCLRECKQQYGKGAGGYCYAFGCWCTHLYEQAVVWPLKNKTCNGK
Beta-mammal toxin Css2	KEGYLVSKSTGCKYECLKLGDNDYCLRECKQQYGKSSGGYCYAFACWCTHLYEQAVVWPLPNKTCN
Toxin CngtIII	KEGYLVNKSTGCKYGCFWLGKNEGCDKECKAKNQGGSYGYCYAFGCWCEGLPESTPTYPLPNKTCSKK
BmKa1	DGYIADDKNCPYFCGRNAYCDDECKKNGAESGYCQWAGVYGNACWCYKLPDKVPIRVPGKCNGG
Lqh4	GVRDAYIADDKNCVYTCGANSYCNTECTKNGAESGYCQWFGKYGNACWCIKLPDKVPIRIPGKCR
Alpha-mammal toxin Bot3	VKDGYIVDDRNCTYFCGRNAYCNEECTKLKGESGYCQWASPYGNACYCYKVPDHVRTKGPGRCN
Toxin Tst1	GKEGYLMDHEGCKLSCFIRPSGYCGRECTLKKGSSGYCAWPACYCYGLPNWVKVWDRATNKC
Toxin To1	KKEGYLVGNDGCKYGCITRPHQYCVHECELKKGTDGYCAYWLACYCYNMPDWVKTWSSATNKCK
Toxin To4	DGYLMEYGGCKMSCLMKKGTFCAEECTRMKGKDGYCYAWLACYCYNMPDWVKIWNRATNKCGKRK
Tz1	DGYLVGNDGCKYSCFTRPGTYCANECSRVKGKDGYCYAWMACYCYSMPNWVKTWDRATNRCGRGK

```
NUMMDL    20
MODEL        1
ATOM      1  N   GLY     1      27.471  -3.636  16.180
1.00999.99           N
ATOM      2  CA  GLY     1      26.154  -3.730  16.872
1.00999.99           C
ATOM      3  C   GLY     1      25.347  -2.465  16.608
1.00999.99           C
ATOM      4  O   GLY     1      24.697  -1.933  17.508
1.00999.99           O
ATOM      5  HA1 GLY     1      26.312  -3.837  17.934
1.00999.99           H
ATOM      6  HA2 GLY     1      25.613  -4.589  16.499
1.00999.99           H
ATOM      7  HT1 GLY     1      27.469  -4.255  15.345
1.00999.99           H
ATOM      8  HT2 GLY     1      28.226  -3.933  16.831
1.00999.99           H
ATOM      9  HT3 GLY     1      27.636  -2.654  15.880
1.00999.99           H
ATOM     10  N   LEU     2      25.390  -1.987  15.369
1.00999.99           N
ATOM     11  CA  LEU     2      24.655  -0.783  14.999
1.00895.59           C
ATOM     12  C   LEU     2      23.160  -1.077  14.935
1.00532.27           C
ATOM     13  O   LEU     2      22.750  -2.166  14.532
1.00625.21           O
ATOM     14  CB  LEU     2      25.134  -0.275  13.638
1.00999.99           C
ATOM     15  CG  LEU     2      26.641   0.005  13.682
1.00999.99           C
ATOM     16  CD1 LEU     2      27.111   0.435  12.289
1.00999.99           C
ATOM     17  CD2 LEU     2      26.943   1.123  14.698
1.00999.99           C
ATOM     18  HN  LEU     2      25.923  -2.453  14.692
1.00999.99           H
ATOM     19  HA  LEU     2      24.829  -0.020  15.742
1.00890.87           H
ATOM     20  HB1 LEU     2      24.609   0.636  13.391
1.00924.07           H
ATOM     21  HB2 LEU     2      24.931  -1.023  12.885
1.00999.99           H
ATOM     22  HG  LEU     2      27.163  -0.896  13.972
1.00999.99           H
ATOM     23 HD11 LEU     2      26.827  -0.316  11.567
1.00999.99           H
ATOM     24 HD12 LEU     2      28.185   0.546  12.289
1.00999.99           H
ATOM     25 HD13 LEU     2      26.651   1.377  12.029
1.00999.99           H
```

```
ATOM     26 HD21 LEU     2      26.145   1.852  14.688
1.00999.99           H
ATOM     27 HD22 LEU     2      27.874   1.611  14.440
1.00999.99           H
ATOM     28 HD23 LEU     2      27.029   0.697  15.686
1.00999.99           H
ATOM     29  N   CYS     3      22.348  -0.101  15.335
1.00271.28           N
ATOM     30  CA  CYS     3      20.897  -0.267  15.321
1.00104.50           C
ATOM     31  C   CYS     3      20.312   0.145  13.974  1.00
90.98           C
ATOM     32  O   CYS     3      20.387   1.308  13.578
1.00207.28           O
ATOM     33  CB  CYS     3      20.264   0.571  16.433  1.00
37.83           C
ATOM     34  SG  CYS     3      20.603  -0.207  18.031  1.00
81.56           S
ATOM     35  HN  CYS     3      22.732   0.744  15.648
1.00296.55           H
ATOM     36  HA  CYS     3      20.659  -1.301  15.498
1.00166.45           H
ATOM     37  HB1 CYS     3      19.198   0.629  16.279  1.00
40.38           H
ATOM     38  HB2 CYS     3      20.685   1.567  16.420  1.00
98.45           H
ATOM     39  N   SER     4      19.720  -0.824  13.280  1.00
85.31           N
ATOM     40  CA  SER     4      19.110  -0.563  11.983  1.00
92.94           C
ATOM     41  C   SER     4      17.816   0.212  12.165  1.00
67.37           C
ATOM     42  O   SER     4      17.702   1.373  11.770
1.00102.28           O
ATOM     43  CB  SER     4      18.792  -1.886  11.286
1.00123.83           C
ATOM     44  OG  SER     4      18.477  -1.635   9.923
1.00178.89           O
ATOM     45  HN  SER     4      19.687  -1.729  13.653
1.00163.03           H
ATOM     46  HA  SER     4      19.791   0.008  11.370
1.00129.78           H
ATOM     47  HB1 SER     4      17.945  -2.355  11.781
1.00105.26           H
ATOM     48  HB2 SER     4      19.646  -2.540  11.340
1.00156.87           H
ATOM     49  HG  SER     4      19.121  -2.098   9.381
1.00218.16           H
ATOM     50  N   GLU     5      16.846  -0.457  12.773  1.00
41.67           N
ATOM     51  CA  GLU     5      15.544   0.141  13.027  1.00
41.21           C
```

```
ATOM     52  C   GLU     5      15.583   0.993  14.289  1.00
41.92           C
ATOM     53  O   GLU     5      16.451   0.814  15.144  1.00
74.72           O
ATOM     54  CB  GLU     5      14.488  -0.952  13.168  1.00
49.24           C
ATOM     55  CG  GLU     5      14.952  -1.961  14.207  1.00
55.48           C
ATOM     56  CD  GLU     5      13.930  -3.084  14.342  1.00
92.06           C
ATOM     57  OE1 GLU     5      14.172  -3.988  15.125
1.00206.49           O
ATOM     58  OE2 GLU     5      12.918  -3.024  13.661
1.00197.24           O1-
ATOM     59  HN  GLU     5      17.012  -1.376  13.058  1.00
45.52           H
ATOM     60  HA  GLU     5      15.281   0.760  12.193  1.00
60.63           H
ATOM     61  HB1 GLU     5      14.351  -1.449  12.220  1.00
61.59           H
ATOM     62  HB2 GLU     5      13.558  -0.513  13.482  1.00
63.66           H
ATOM     63  HG1 GLU     5      15.074  -1.470  15.158  1.00
58.03           H
ATOM     64  HG2 GLU     5      15.894  -2.369  13.892  1.00
57.77           H
ATOM     65  N   ASN     6      14.644   1.926  14.397  1.00
50.73           N
ATOM     66  CA  ASN     6      14.585   2.806  15.558  1.00
71.04           C
ATOM     67  C   ASN     6      14.379   2.004  16.841  1.00
65.17           C
ATOM     68  O   ASN     6      15.006   2.281  17.862  1.00
94.86           O
ATOM     69  CB  ASN     6      13.444   3.810  15.395  1.00
94.83           C
ATOM     70  CG  ASN     6      13.434   4.784  16.567
1.00160.94           C
ATOM     71  ND2 ASN     6      12.371   4.874  17.319
1.00244.88           N
ATOM     72  OD1 ASN     6      14.419   5.483  16.804
1.00219.73           O
ATOM     73  HN  ASN     6      13.981   2.027  13.682  1.00
72.81           H
ATOM     74  HA  ASN     6      15.516   3.348  15.632  1.00
96.02           H
ATOM     75  HB1 ASN     6      12.503   3.281  15.362
1.00111.09           H
ATOM     76  HB2 ASN     6      13.579   4.358  14.473  1.00
98.13           H
ATOM     77 HD21 ASN     6      11.587   4.317  17.130
1.00272.43           H
```

```
ATOM     78 HD22 ASN     6       12.357   5.499  18.075
1.00336.78           H
ATOM     79  N   GLY     7       13.489   1.015  16.786  1.00
51.80          N
ATOM     80  CA  GLY     7       13.199   0.184  17.957  1.00
71.47          C
ATOM     81  C   GLY     7       14.008  -1.106  17.939  1.00
41.04          C
ATOM     82  O   GLY     7       13.453  -2.199  18.048  1.00
44.54          O
ATOM     83  HN  GLY     7       13.014   0.843  15.946  1.00
45.49          H
ATOM     84  HA1 GLY     7       12.154  -0.064  17.958
1.00103.52           H
ATOM     85  HA2 GLY     7       13.432   0.734  18.858
1.00108.67           H
ATOM     86  N   ASP     8       15.318  -0.971  17.806  1.00
27.00          N
ATOM     87  CA  ASP     8       16.196  -2.137  17.781  1.00
14.15          C
ATOM     88  C   ASP     8       16.121  -2.896  19.100  1.00
9.37          C
ATOM     89  O   ASP     8       15.998  -4.120  19.117  1.00
14.61          O
ATOM     90  CB  ASP     8       17.640  -1.706  17.523  1.00
15.06          C
ATOM     91  CG  ASP     8       18.535  -2.935  17.399  1.00
21.22          C
ATOM     92  OD1 ASP     8       18.044  -4.027  17.630
1.00119.56           O
ATOM     93  OD2 ASP     8       19.698  -2.765  17.075
1.00133.27           O1-
ATOM     94  HN  ASP     8       15.700  -0.074  17.728  1.00
35.63          H
ATOM     95  HA  ASP     8       15.881  -2.791  16.987  1.00
20.92          H
ATOM     96  HB1 ASP     8       17.983  -1.097  18.345  1.00
29.39          H
ATOM     97  HB2 ASP     8       17.685  -1.134  16.608  1.00
44.14          H
ATOM     98  N   CYS     9       16.197  -2.160  20.201  1.00
5.93          N
ATOM     99  CA  CYS     9       16.137  -2.774  21.522  1.00
7.54          C
ATOM    100  C   CYS     9       14.787  -3.448  21.726  1.00
14.60          C
ATOM    101  O   CYS     9       14.699  -4.555  22.256  1.00
25.78          O
ATOM    102  CB  CYS     9       16.348  -1.719  22.600  1.00
6.63          C
ATOM    103  SG  CYS     9       18.047  -1.147  22.526  1.00
10.91          S
```

ATOM 104 HN CYS 9 16.295 -1.190 20.121 1.00
6.51 H
ATOM 105 HA CYS 9 16.923 -3.506 21.604 1.00
11.12 H
ATOM 106 HB1 CYS 9 16.157 -2.147 23.572 1.00
11.28 H
ATOM 107 HB2 CYS 9 15.686 -0.883 22.436 1.00
5.04 H
ATOM 108 N ALA 10 13.738 -2.760 21.290 1.00
16.26 N
ATOM 109 CA ALA 10 12.379 -3.272 21.408 1.00
32.04 C
ATOM 110 C ALA 10 11.418 -2.380 20.626 1.00
45.47 C
ATOM 111 O ALA 10 11.789 -1.289 20.191
1.00119.04 O
ATOM 112 CB ALA 10 11.960 -3.328 22.881 1.00
30.10 C
ATOM 113 HN ALA 10 13.885 -1.885 20.874 1.00
12.81 H
ATOM 114 HA ALA 10 12.345 -4.270 20.996 1.00
47.14 H
ATOM 115 HB1 ALA 10 11.143 -4.025 22.996 1.00
84.37 H
ATOM 116 HB2 ALA 10 11.647 -2.349 23.207
1.00118.20 H
ATOM 117 HB3 ALA 10 12.798 -3.656 23.480
1.00117.32 H
ATOM 118 N ALA 11 10.189 -2.847 20.445 1.00
30.62 N
ATOM 119 CA ALA 11 9.196 -2.074 19.708 1.00
35.24 C
ATOM 120 C ALA 11 8.927 -0.736 20.394 1.00
22.78 C
ATOM 121 O ALA 11 8.811 0.297 19.733 1.00
53.33 O
ATOM 122 CB ALA 11 7.891 -2.866 19.604 1.00
60.49 C
ATOM 123 HN ALA 11 9.946 -3.723 20.810 1.00
50.37 H
ATOM 124 HA ALA 11 9.567 -1.887 18.711 1.00
41.70 H
ATOM 125 HB1 ALA 11 7.589 -3.190 20.589
1.00157.06 H
ATOM 126 HB2 ALA 11 8.041 -3.727 18.971
1.00148.83 H
ATOM 127 HB3 ALA 11 7.122 -2.237 19.181
1.00137.06 H
ATOM 128 N ASP 12 8.828 -0.760 21.720 1.00
18.98 N
ATOM 129 CA ASP 12 8.568 0.460 22.484 1.00
31.05 C

```
ATOM    130  C   ASP    12        9.859   1.238  22.730  1.00
22.98          C
ATOM    131  O   ASP    12        9.850   2.467  22.804  1.00
37.14          O
ATOM    132  CB  ASP    12        7.925   0.107  23.825  1.00
48.35          C
ATOM    133  CG  ASP    12        6.511  -0.422  23.602
1.00104.83          C
ATOM    134  OD1 ASP    12        5.965  -1.001  24.527
1.00258.73          O
ATOM    135  OD2 ASP    12        5.997  -0.240  22.512
1.00213.46          O1-
ATOM    136  HN  ASP    12        8.927  -1.612  22.195  1.00
36.19          H
ATOM    137  HA  ASP    12        7.885   1.085  21.928  1.00
49.76          H
ATOM    138  HB1 ASP    12        7.883   0.989  24.447  1.00
95.71          H
ATOM    139  HB2 ASP    12        8.517  -0.651  24.317  1.00
54.37          H
ATOM    140  N   GLU    13       10.965   0.514  22.863  1.00
17.60          N
ATOM    141  CA  GLU    13       12.260   1.145  23.109  1.00
12.01          C
ATOM    142  C   GLU    13       12.801   1.793  21.838  1.00
10.07          C
ATOM    143  O   GLU    13       12.294   1.552  20.742  1.00
12.81          O
ATOM    144  CB  GLU    13       13.253   0.102  23.624  1.00
11.74          C
ATOM    145  CG  GLU    13       12.786  -0.412  24.987  1.00
13.49          C
ATOM    146  CD  GLU    13       13.681  -1.556  25.450
1.00142.36          C
ATOM    147  OE1 GLU    13       14.889  -1.395  25.396
1.00339.06          O
ATOM    148  OE2 GLU    13       13.146  -2.576  25.851
1.00335.28          O1-
ATOM    149  HN  GLU    13       10.910  -0.462  22.800  1.00
28.36          H
ATOM    150  HA  GLU    13       12.138   1.907  23.863  1.00
13.06          H
ATOM    151  HB1 GLU    13       14.228   0.554  23.726  1.00
13.76          H
ATOM    152  HB2 GLU    13       13.307  -0.720  22.924  1.00
9.94          H
ATOM    153  HG1 GLU    13       11.769  -0.763  24.910  1.00
66.02          H
ATOM    154  HG2 GLU    13       12.834   0.392  25.707  1.00
56.23          H
ATOM    155  N   CYS    14       13.833   2.622  21.998  1.00
9.12          N
```

ATOM 156 CA CYS 14 14.451 3.320 20.865 1.00
10.69 C
ATOM 157 C CYS 14 15.967 3.159 20.897 1.00
9.56 C
ATOM 158 O CYS 14 16.577 3.148 21.965 1.00
13.62 O
ATOM 159 CB CYS 14 14.103 4.807 20.910 1.00
13.96 C
ATOM 160 SG CYS 14 14.971 5.664 19.572 1.00
42.96 S
ATOM 161 HN CYS 14 14.187 2.774 22.900 1.00
9.76 H
ATOM 162 HA CYS 14 14.078 2.905 19.938 1.00
15.54 H
ATOM 163 HB1 CYS 14 14.406 5.218 21.861 1.00
50.72 H
ATOM 164 HB2 CYS 14 13.036 4.932 20.788 1.00
46.13 H
ATOM 165 N CYS 15 16.567 3.042 19.713 1.00
9.60 N
ATOM 166 CA CYS 15 18.015 2.887 19.591 1.00
9.28 C
ATOM 167 C CYS 15 18.525 3.792 18.484 1.00
9.92 C
ATOM 168 O CYS 15 17.900 3.902 17.429 1.00
13.31 O
ATOM 169 CB CYS 15 18.369 1.442 19.244 1.00
12.90 C
ATOM 170 SG CYS 15 20.159 1.219 19.390 1.00
39.11 S
ATOM 171 HN CYS 15 16.025 3.069 18.899 1.00
13.12 H
ATOM 172 HA CYS 15 18.496 3.156 20.523 1.00
8.26 H
ATOM 173 HB1 CYS 15 18.062 1.233 18.231 1.00
24.82 H
ATOM 174 HB2 CYS 15 17.862 0.770 19.917 1.00
27.31 H
ATOM 175 N VAL 16 19.659 4.441 18.716 1.00
9.56 N
ATOM 176 CA VAL 16 20.222 5.331 17.710 1.00
11.99 C
ATOM 177 C VAL 16 21.742 5.334 17.795 1.00
7.73 C
ATOM 178 O VAL 16 22.319 5.701 18.818 1.00
8.83 O
ATOM 179 CB VAL 16 19.668 6.746 17.912 1.00
18.37 C
ATOM 180 CG1 VAL 16 20.279 7.385 19.167 1.00
39.61 C
ATOM 181 CG2 VAL 16 19.991 7.596 16.683
1.00115.21 C

```
ATOM    182  HN  VAL    16      20.125   4.320  19.573  1.00
9.76            H
ATOM    183  HA  VAL    16      19.935   4.985  16.725  1.00
17.27           H
ATOM    184  HB  VAL    16      18.596   6.689  18.032  1.00
52.63           H
ATOM    185 HG11 VAL    16      21.258   7.781  18.937
1.00128.07           H
ATOM    186 HG12 VAL    16      20.363   6.642  19.946
1.00154.13           H
ATOM    187 HG13 VAL    16      19.639   8.187  19.507
1.00135.74           H
ATOM    188 HG21 VAL    16      19.352   7.295  15.866
1.00229.05           H
ATOM    189 HG22 VAL    16      21.025   7.451  16.406
1.00261.82           H
ATOM    190 HG23 VAL    16      19.820   8.637  16.910
1.00210.07           H
ATOM    191  N   ASP    17      22.386   4.904  16.716  1.00
14.34           N
ATOM    192  CA  ASP    17      23.840   4.847  16.684  1.00
11.90           C
ATOM    193  C   ASP    17      24.440   6.238  16.513  1.00
13.15           C
ATOM    194  O   ASP    17      24.215   6.903  15.501  1.00
25.96           O
ATOM    195  CB  ASP    17      24.303   3.961  15.529  1.00
20.80           C
ATOM    196  CG  ASP    17      23.727   2.560  15.676  1.00
28.72           C
ATOM    197  OD1 ASP    17      23.227   2.043  14.692
1.00135.42           O
ATOM    198  OD2 ASP    17      23.794   2.024  16.771
1.00114.17           O1-
ATOM    199  HN  ASP    17      21.873   4.606  15.933  1.00
28.29           H
ATOM    200  HA  ASP    17      24.194   4.425  17.612  1.00
10.49           H
ATOM    201  HB1 ASP    17      25.382   3.906  15.532  1.00
22.42           H
ATOM    202  HB2 ASP    17      23.970   4.389  14.595  1.00
29.52           H
ATOM    203  N   THR    18      25.228   6.659  17.497  1.00
11.55           N
ATOM    204  CA  THR    18      25.892   7.960  17.446  1.00
17.10           C
ATOM    205  C   THR    18      27.320   7.778  16.954  1.00
10.63           C
ATOM    206  O   THR    18      27.864   6.678  17.022  1.00
6.49            O
ATOM    207  CB  THR    18      25.908   8.606  18.832  1.00
28.52           C
```

```
ATOM    208  CG2 THR    18       24.514   9.122  19.181  1.00
45.38           C
ATOM    209  OG1 THR    18       26.319   7.648  19.793  1.00
26.49           O
ATOM    210  HN  THR    18       25.384   6.074  18.268  1.00
13.96           H
ATOM    211  HA  THR    18       25.363   8.611  16.761  1.00
26.43           H
ATOM    212  HB  THR    18       26.601   9.433  18.835  1.00
37.43           H
ATOM    213  HG1 THR    18       25.574   7.070  19.973  1.00
71.62           H
ATOM    214 HG21 THR    18       23.778   8.376  18.922
1.00100.44           H
ATOM    215 HG22 THR    18       24.320  10.030  18.629
1.00129.42           H
ATOM    216 HG23 THR    18       24.463   9.327  20.239
1.00156.21           H
ATOM    217  N   VAL    19       27.923   8.858  16.476  1.00
16.32           N
ATOM    218  CA  VAL    19       29.281   8.819  15.988  1.00
13.34           C
ATOM    219  C   VAL    19       29.676  10.195  15.503  1.00
25.88           C
ATOM    220  O   VAL    19       29.085  10.754  14.579  1.00
42.11           O
ATOM    221  CB  VAL    19       29.448   7.804  14.856  1.00
15.48           C
ATOM    222  CG1 VAL    19       28.403   8.052  13.765  1.00
29.06           C
ATOM    223  CG2 VAL    19       30.856   7.951  14.264  1.00
20.67           C
ATOM    224  HN  VAL    19       27.452   9.711  16.465  1.00
26.89           H
ATOM    225  HA  VAL    19       29.931   8.538  16.804  1.00
8.08           H
ATOM    226  HB  VAL    19       29.329   6.811  15.252  1.00
11.49           H
ATOM    227 HG11 VAL    19       27.439   8.230  14.219
1.00137.43           H
ATOM    228 HG12 VAL    19       28.342   7.185  13.123  1.00
93.29           H
ATOM    229 HG13 VAL    19       28.689   8.913  13.178
1.00108.69           H
ATOM    230 HG21 VAL    19       30.894   8.837  13.645  1.00
99.42           H
ATOM    231 HG22 VAL    19       31.091   7.083  13.668
1.00111.36           H
ATOM    232 HG23 VAL    19       31.577   8.046  15.067  1.00
77.11           H
ATOM    233  N   PHE    20       30.672  10.732  16.161  1.00
24.58           N
```

```
ATOM    234  CA  PHE    20       31.184  12.056  15.859  1.00
40.34         C
ATOM    235  C   PHE    20       32.490  11.982  15.086  1.00
40.37         C
ATOM    236  O   PHE    20       32.615  12.565  14.010  1.00
66.02         O
ATOM    237  CB  PHE    20       31.397  12.825  17.171  1.00
48.79         C
ATOM    238  CG  PHE    20       31.868  11.897  18.282  1.00
35.75         C
ATOM    239  CD1 PHE    20       31.017  10.895  18.797  1.00
30.20         C
ATOM    240  CD2 PHE    20       33.152  12.055  18.820  1.00
40.95         C
ATOM    241  CE1 PHE    20       31.459  10.067  19.832  1.00
30.41         C
ATOM    242  CE2 PHE    20       33.589  11.220  19.855  1.00
45.84         C
ATOM    243  CZ  PHE    20       32.743  10.228  20.361  1.00
40.81         C
ATOM    244  HN  PHE    20       31.061  10.223  16.892  1.00
16.73         H
ATOM    245  HA  PHE    20       30.461  12.594  15.258  1.00
59.91         H
ATOM    246  HB1 PHE    20       30.474  13.268  17.461  1.00
67.15         H
ATOM    247  HB2 PHE    20       32.128  13.609  17.022  1.00
58.17         H
ATOM    248  HD1 PHE    20       30.022  10.754  18.395  1.00
33.34         H
ATOM    249  HD2 PHE    20       33.808  12.818  18.433  1.00
49.35         H
ATOM    250  HE1 PHE    20       30.805   9.303  20.223  1.00
30.61         H
ATOM    251  HE2 PHE    20       34.579  11.343  20.266  1.00
61.27         H
ATOM    252  HZ  PHE    20       33.081   9.586  21.161  1.00
52.66         H
ATOM    253  N   GLU    21       33.478  11.298  15.657  1.00
41.31         N
ATOM    254  CA  GLU    21       34.781  11.195  15.045  1.00
56.16         C
ATOM    255  C   GLU    21       35.089   9.769  14.609  1.00
58.61         C
ATOM    256  O   GLU    21       34.238   8.882  14.682
1.00201.19          O
ATOM    257  CB  GLU    21       35.780  11.665  16.075  1.00
57.37         C
ATOM    258  CG  GLU    21       35.804  10.687  17.248
1.00193.56          C
ATOM    259  CD  GLU    21       36.625  11.273  18.392
1.00304.62          C
```

```
ATOM    260  OE1 GLU    21       36.667  10.657  19.444
1.00451.81          O
ATOM    261  OE2 GLU    21       37.201  12.332  18.199
1.00442.88          O1-
ATOM    262  HN  GLU    21       33.344  10.883  16.532  1.00
50.82          H
ATOM    263  HA  GLU    21       34.842  11.847  14.184  1.00
85.72          H
ATOM    264  HB1 GLU    21       35.475  12.635  16.430  1.00
46.37          H
ATOM    265  HB2 GLU    21       36.751  11.725  15.631
1.00131.81          H
ATOM    266  HG1 GLU    21       36.244   9.759  16.929
1.00314.91          H
ATOM    267  HG2 GLU    21       34.796  10.505  17.582
1.00266.92          H
ATOM    268  N   GLY    22       36.316   9.570  14.140  1.00
86.70          N
ATOM    269  CA  GLY    22       36.755   8.254  13.666
1.00104.66          C
ATOM    270  C   GLY    22       37.491   7.473  14.753  1.00
71.50          C
ATOM    271  O   GLY    22       37.779   6.288  14.585  1.00
89.87          O
ATOM    272  HN  GLY    22       36.936  10.330  14.105
1.00214.64          H
ATOM    273  HA1 GLY    22       37.419   8.391  12.826
1.00152.47          H
ATOM    274  HA2 GLY    22       35.895   7.682  13.344
1.00118.15          H
ATOM    275  N   ASP    23       37.800   8.136  15.860  1.00
44.68          N
ATOM    276  CA  ASP    23       38.511   7.478  16.953  1.00
43.33          C
ATOM    277  C   ASP    23       37.700   6.312  17.503  1.00
37.55          C
ATOM    278  O   ASP    23       38.245   5.252  17.808  1.00
61.37          O
ATOM    279  CB  ASP    23       38.786   8.478  18.076  1.00
40.74          C
ATOM    280  CG  ASP    23       39.842   9.486  17.634
1.00141.86          C
ATOM    281  OD1 ASP    23       40.488   9.234  16.630
1.00328.18          O
ATOM    282  OD2 ASP    23       39.989  10.493  18.305
1.00304.58          O1-
ATOM    283  HN  ASP    23       37.556   9.081  15.942  1.00
40.86          H
ATOM    284  HA  ASP    23       39.451   7.105  16.582  1.00
72.12          H
ATOM    285  HB1 ASP    23       39.142   7.948  18.947  1.00
94.15          H
```

ATOM 286 HB2 ASP 23 37.873 8.997 18.323 1.00
93.26 H
ATOM 287 N MET 24 36.397 6.519 17.631 1.00
23.24 N
ATOM 288 CA MET 24 35.517 5.478 18.150 1.00
32.50 C
ATOM 289 C MET 24 34.060 5.783 17.818 1.00
25.13 C
ATOM 290 O MET 24 33.730 6.880 17.366 1.00
54.40 O
ATOM 291 CB MET 24 35.689 5.357 19.666 1.00
47.36 C
ATOM 292 CG MET 24 35.315 6.680 20.337
1.00151.73 C
ATOM 293 SD MET 24 35.637 6.565 22.115
1.00209.23 S
ATOM 294 CE MET 24 35.098 8.235 22.556
1.00243.29 C
ATOM 295 HN MET 24 36.021 7.386 17.374 1.00
17.32 H
ATOM 296 HA MET 24 35.787 4.537 17.695 1.00
48.94 H
ATOM 297 HB1 MET 24 36.718 5.120 19.893
1.00124.40 H
ATOM 298 HB2 MET 24 35.048 4.571 20.038
1.00166.96 H
ATOM 299 HG1 MET 24 34.267 6.883 20.174
1.00331.71 H
ATOM 300 HG2 MET 24 35.907 7.478 19.915
1.00302.30 H
ATOM 301 HE1 MET 24 35.664 8.582 23.408
1.00340.44 H
ATOM 302 HE2 MET 24 34.049 8.221 22.805
1.00373.88 H
ATOM 303 HE3 MET 24 35.258 8.897 21.716
1.00386.81 H
ATOM 304 N VAL 25 33.191 4.800 18.048 1.00
22.44 N
ATOM 305 CA VAL 25 31.758 4.949 17.776 1.00
15.37 C
ATOM 306 C VAL 25 30.952 4.789 19.059 1.00
17.47 C
ATOM 307 O VAL 25 31.291 3.985 19.928 1.00
29.52 O
ATOM 308 CB VAL 25 31.304 3.905 16.751 1.00
23.36 C
ATOM 309 CG1 VAL 25 29.776 3.959 16.598 1.00
58.39 C
ATOM 310 CG2 VAL 25 31.969 4.199 15.403 1.00
55.25 C
ATOM 311 HN VAL 25 33.519 3.949 18.409 1.00
48.11 H

ATOM 312 HA VAL 25 31.566 5.939 17.373 1.00
9.25 H
ATOM 313 HB VAL 25 31.595 2.920 17.090 1.00
52.45 H
ATOM 314 HG11 VAL 25 29.443 4.986 16.649
1.00171.07 H
ATOM 315 HG12 VAL 25 29.317 3.396 17.398
1.00166.22 H
ATOM 316 HG13 VAL 25 29.488 3.532 15.648
1.00134.76 H
ATOM 317 HG21 VAL 25 31.499 5.060 14.951
1.00141.88 H
ATOM 318 HG22 VAL 25 31.858 3.345 14.752
1.00184.50 H
ATOM 319 HG23 VAL 25 33.019 4.401 15.555
1.00133.90 H
ATOM 320 N THR 26 29.888 5.579 19.169 1.00
13.60 N
ATOM 321 CA THR 26 29.021 5.559 20.347 1.00
21.19 C
ATOM 322 C THR 26 27.603 5.124 19.981 1.00
16.55 C
ATOM 323 O THR 26 27.050 5.555 18.970 1.00
11.43 O
ATOM 324 CB THR 26 28.986 6.960 20.954 1.00
27.07 C
ATOM 325 CG2 THR 26 28.058 6.982 22.173 1.00
44.39 C
ATOM 326 OG1 THR 26 30.299 7.325 21.354 1.00
34.75 O
ATOM 327 HN THR 26 29.684 6.203 18.442 1.00
10.81 H
ATOM 328 HA THR 26 29.419 4.873 21.084 1.00
33.16 H
ATOM 329 HB THR 26 28.627 7.658 20.210 1.00
20.60 H
ATOM 330 HG1 THR 26 30.917 6.959 20.716 1.00
77.72 H
ATOM 331 HG21 THR 26 28.346 6.198 22.856
1.00126.99 H
ATOM 332 HG22 THR 26 27.039 6.827 21.858
1.00108.32 H
ATOM 333 HG23 THR 26 28.140 7.937 22.668
1.00151.99 H
ATOM 334 N ARG 27 27.021 4.268 20.823 1.00
22.38 N
ATOM 335 CA ARG 27 25.659 3.766 20.610 1.00
20.21 C
ATOM 336 C ARG 27 24.817 4.030 21.856 1.00
16.96 C
ATOM 337 O ARG 27 25.360 4.173 22.952 1.00
20.06 O

```
ATOM    338  CB  ARG    27       25.698   2.262  20.333  1.00
22.14           C
ATOM    339  CG  ARG    27       26.549   1.990  19.090
1.00124.30           C
ATOM    340  CD  ARG    27       26.775   0.484  18.947
1.00109.38           C
ATOM    341  NE  ARG    27       27.556  -0.016  20.073
1.00227.73           N
ATOM    342  CZ  ARG    27       27.809  -1.313  20.211
1.00426.12           C
ATOM    343  NH1 ARG    27       28.512  -1.735  21.225
1.00767.09           N1+
ATOM    344  NH2 ARG    27       27.353  -2.165  19.333
1.00581.78           N
ATOM    345  HN  ARG    27       27.518   3.969  21.612  1.00
30.75           H
ATOM    346  HA  ARG    27       25.208   4.270  19.765  1.00
23.13           H
ATOM    347  HB1 ARG    27       24.694   1.901  20.164  1.00
87.46           H
ATOM    348  HB2 ARG    27       26.126   1.751  21.182
1.00103.36           H
ATOM    349  HG1 ARG    27       27.503   2.487  19.191
1.00281.97           H
ATOM    350  HG2 ARG    27       26.038   2.363  18.215
1.00276.19           H
ATOM    351  HD1 ARG    27       27.305   0.290  18.026
1.00183.60           H
ATOM    352  HD2 ARG    27       25.821  -0.019  18.924
1.00142.93           H
ATOM    353  HE  ARG    27       27.901   0.615  20.738
1.00372.53           H
ATOM    354 HH11 ARG    27       28.860  -1.083  21.899
1.00910.59           H
ATOM    355 HH12 ARG    27       28.702  -2.711  21.330
1.00999.99           H
ATOM    356 HH21 ARG    27       26.814  -1.840  18.555
1.00532.54           H
ATOM    357 HH22 ARG    27       27.544  -3.140  19.438
1.00948.84           H
ATOM    358  N   SER    28       23.493   4.100  21.696  1.00
14.60           N
ATOM    359  CA  SER    28       22.616   4.358  22.841  1.00
14.92           C
ATOM    360  C   SER    28       21.270   3.662  22.674  1.00
12.12           C
ATOM    361  O   SER    28       20.862   3.317  21.566  1.00
13.41           O
ATOM    362  CB  SER    28       22.396   5.862  22.997  1.00
23.26           C
ATOM    363  OG  SER    28       21.680   6.351  21.872
1.00146.32           O
```

```
ATOM    364  HN  SER    28       23.102   3.986  20.802  1.00
15.23           H
ATOM    365  HA  SER    28       23.086   3.984  23.740  1.00
15.44           H
ATOM    366  HB1 SER    28       23.355   6.359  23.070  1.00
88.73           H
ATOM    367  HB2 SER    28       21.827   6.054  23.891
1.00124.68           H
ATOM    368  HG  SER    28       20.812   5.941  21.872
1.00242.47           H
ATOM    369  N   CYS    29       20.584   3.468  23.798  1.00
10.48           N
ATOM    370  CA  CYS    29       19.278   2.822  23.803  1.00
9.97           C
ATOM    371  C   CYS    29       18.705   2.859  25.218  1.00
11.60           C
ATOM    372  O   CYS    29       19.427   2.627  26.189  1.00
15.64           O
ATOM    373  CB  CYS    29       19.399   1.370  23.315  1.00
9.52           C
ATOM    374  SG  CYS    29       17.849   0.854  22.574  1.00
10.40           S
ATOM    375  HN  CYS    29       20.964   3.776  24.647  1.00
11.10           H
ATOM    376  HA  CYS    29       18.617   3.363  23.142  1.00
11.08           H
ATOM    377  HB1 CYS    29       19.617   0.711  24.142  1.00
9.33           H
ATOM    378  HB2 CYS    29       20.183   1.293  22.580  1.00
10.78           H
ATOM    379  N   GLU    30       17.412   3.159  25.336  1.00
13.52           N
ATOM    380  CA  GLU    30       16.763   3.234  26.649  1.00
17.41           C
ATOM    381  C   GLU    30       15.779   2.085  26.837  1.00
10.81           C
ATOM    382  O   GLU    30       14.988   1.777  25.946  1.00
23.22           O
ATOM    383  CB  GLU    30       16.025   4.567  26.780  1.00
37.83           C
ATOM    384  CG  GLU    30       17.030   5.714  26.659
1.00108.13           C
ATOM    385  CD  GLU    30       17.929   5.750  27.890
1.00240.52           C
ATOM    386  OE1 GLU    30       17.580   5.119  28.873
1.00422.56           O
ATOM    387  OE2 GLU    30       18.954   6.410  27.831
1.00410.81           O1-
ATOM    388  HN  GLU    30       16.886   3.343  24.529  1.00
15.61           H
ATOM    389  HA  GLU    30       17.510   3.180  27.429  1.00
24.60           H
```

```
ATOM    390  HB1 GLU    30       15.538   4.617  27.742  1.00
72.67           H
ATOM    391  HB2 GLU    30       15.285   4.648  25.995  1.00
56.47           H
ATOM    392  HG1 GLU    30       16.498   6.650  26.576
1.00196.66           H
ATOM    393  HG2 GLU    30       17.636   5.567  25.778
1.00140.67           H
ATOM    394  N   LYS    31       15.833   1.460  28.010  1.00
11.96           N
ATOM    395  CA  LYS    31       14.941   0.347  28.320  1.00
11.40           C
ATOM    396  C   LYS    31       13.630   0.881  28.885  1.00
10.99           C
ATOM    397  O   LYS    31       13.626   1.835  29.662  1.00
12.86           O
ATOM    398  CB  LYS    31       15.596  -0.579  29.352  1.00
21.40           C
ATOM    399  CG  LYS    31       17.033  -0.920  28.932  1.00
57.81           C
ATOM    400  CD  LYS    31       17.034  -1.734  27.633
1.00115.52           C
ATOM    401  CE  LYS    31       18.440  -2.282  27.383
1.00250.23           C
ATOM    402  NZ  LYS    31       18.451  -3.056  26.109
1.00462.10           N1+
ATOM    403  HN  LYS    31       16.483   1.755  28.682  1.00
26.13           H
ATOM    404  HA  LYS    31       14.732  -0.212  27.423  1.00
13.87           H
ATOM    405  HB1 LYS    31       15.022  -1.491  29.428  1.00
37.63           H
ATOM    406  HB2 LYS    31       15.612  -0.087  30.313  1.00
47.19           H
ATOM    407  HG1 LYS    31       17.506  -1.497  29.713
1.00126.32           H
ATOM    408  HG2 LYS    31       17.587  -0.005  28.780
1.00114.19           H
ATOM    409  HD1 LYS    31       16.751  -1.099  26.808
1.00198.45           H
ATOM    410  HD2 LYS    31       16.334  -2.552  27.718
1.00200.14           H
ATOM    411  HE1 LYS    31       18.725  -2.929  28.199
1.00374.64           H
ATOM    412  HE2 LYS    31       19.138  -1.463  27.312
1.00403.27           H
ATOM    413  HZ1 LYS    31       18.341  -4.068  26.316
1.00627.38           H
ATOM    414  HZ2 LYS    31       19.355  -2.898  25.616
1.00622.26           H
ATOM    415  HZ3 LYS    31       17.667  -2.742  25.504
1.00619.20           H
```

```
ATOM    416  N   THR    32       12.517   0.267  28.490  1.00
15.51           N
ATOM    417  CA  THR    32       11.206   0.702  28.967  1.00
22.44           C
ATOM    418  C   THR    32       10.827  -0.036  30.247  1.00
26.60           C
ATOM    419  O   THR    32       10.412  -1.195  30.211  1.00
57.43           O
ATOM    420  CB  THR    32       10.148   0.437  27.892  1.00
56.28           C
ATOM    421  CG2 THR    32        8.781   0.908  28.391
1.00102.70           C
ATOM    422  OG1 THR    32       10.492   1.146  26.709
1.00111.17           O
ATOM    423  HN  THR    32       12.578  -0.487  27.867  1.00
18.79           H
ATOM    424  HA  THR    32       11.233   1.763  29.170  1.00
19.67           H
ATOM    425  HB  THR    32       10.104  -0.620  27.680  1.00
84.25           H
ATOM    426  HG1 THR    32        9.680   1.463  26.306
1.00205.03           H
ATOM    427 HG21 THR    32        8.407   0.210  29.126
1.00220.71           H
ATOM    428 HG22 THR    32        8.093   0.959  27.560
1.00174.67           H
ATOM    429 HG23 THR    32        8.878   1.885  28.839
1.00217.44           H
ATOM    430  N   THR    33       10.964   0.654  31.377  1.00
22.47           N
ATOM    431  CA  THR    33       10.627   0.077  32.679  1.00
41.60           C
ATOM    432  C   THR    33        9.903   1.088  33.517  1.00
38.03           C
ATOM    433  O   THR    33       10.377   2.202  33.732  1.00
52.73           O
ATOM    434  CB  THR    33       11.887  -0.377  33.408  1.00
63.25           C
ATOM    435  CG2 THR    33       11.495  -1.193  34.641
1.00121.65           C
ATOM    436  OG1 THR    33       12.676  -1.179  32.540  1.00
96.20           O
ATOM    437  HN  THR    33       11.292   1.576  31.335  1.00
23.82           H
ATOM    438  HA  THR    33        9.968  -0.761  32.560  1.00
67.12           H
ATOM    439  HB  THR    33       12.446   0.486  33.719
1.00107.59           H
ATOM    440  HG1 THR    33       12.111  -1.487  31.827
1.00176.27           H
ATOM    441 HG21 THR    33       10.931  -0.571  35.320
1.00255.23           H
```

```
ATOM    442 HG22 THR    33       12.388  -1.549  35.135
1.00245.37           H
ATOM    443 HG23 THR    33       10.891  -2.036  34.338
1.00185.78           H
ATOM    444  N   GLY    34        8.750   0.677  33.999  1.00
40.93           N
ATOM    445  CA  GLY    34        7.960   1.542  34.825  1.00
45.59           C
ATOM    446  C   GLY    34        7.496   2.761  34.037  1.00
40.97           C
ATOM    447  O   GLY    34        6.319   2.887  33.699  1.00
82.17           O
ATOM    448  HN  GLY    34        8.438  -0.226  33.797  1.00
54.00           H
ATOM    449  HA1 GLY    34        8.579   1.854  35.633  1.00
54.41           H
ATOM    450  HA2 GLY    34        7.104   1.001  35.205  1.00
62.09           H
ATOM    451  N   ASN    35        8.441   3.652  33.733  1.00
32.51           N
ATOM    452  CA  ASN    35        8.147   4.865  32.965  1.00
33.20           C
ATOM    453  C   ASN    35        8.813   4.783  31.595  1.00
23.17           C
ATOM    454  O   ASN    35        9.927   4.276  31.467  1.00
27.01           O
ATOM    455  CB  ASN    35        8.668   6.094  33.713  1.00
54.51           C
ATOM    456  CG  ASN    35        7.979   6.211  35.068  1.00
80.73           C
ATOM    457  ND2 ASN    35        8.676   6.552  36.118
1.00220.46           N
ATOM    458  OD1 ASN    35        6.774   5.985  35.175
1.00117.80           O
ATOM    459  HN  ASN    35        9.360   3.486  34.024  1.00
51.15           H
ATOM    460  HA  ASN    35        7.078   4.963  32.831  1.00
47.21           H
ATOM    461  HB1 ASN    35        8.463   6.980  33.131  1.00
68.31           H
ATOM    462  HB2 ASN    35        9.734   5.998  33.858  1.00
57.75           H
ATOM    463 HD21 ASN    35        9.636   6.731  36.030
1.00405.39           H
ATOM    464 HD22 ASN    35        8.240   6.629  36.992
1.00245.64           H
ATOM    465  N   PHE    36        8.124   5.280  30.573  1.00
35.72           N
ATOM    466  CA  PHE    36        8.659   5.250  29.220  1.00
33.41           C
ATOM    467  C   PHE    36        9.816   6.233  29.073  1.00
28.51           C
```

ATOM 468 O PHE 36 9.804 7.315 29.661 1.00
45.23 O
ATOM 469 CB PHE 36 7.553 5.603 28.230 1.00
67.53 C
ATOM 470 CG PHE 36 6.562 4.466 28.153 1.00
86.71 C
ATOM 471 CD1 PHE 36 5.492 4.409 29.054
1.00106.51 C
ATOM 472 CD2 PHE 36 6.713 3.469 27.182 1.00
99.48 C
ATOM 473 CE1 PHE 36 4.573 3.355 28.983
1.00134.48 C
ATOM 474 CE2 PHE 36 5.795 2.416 27.111
1.00134.27 C
ATOM 475 CZ PHE 36 4.723 2.359 28.012
1.00149.48 C
ATOM 476 HN PHE 36 7.240 5.668 30.728 1.00
62.87 H
ATOM 477 HA PHE 36 9.016 4.253 29.005 1.00
28.53 H
ATOM 478 HB1 PHE 36 7.982 5.771 27.259 1.00
73.56 H
ATOM 479 HB2 PHE 36 7.047 6.500 28.559 1.00
88.42 H
ATOM 480 HD1 PHE 36 5.377 5.178 29.804
1.00110.20 H
ATOM 481 HD2 PHE 36 7.540 3.512 26.487 1.00
93.07 H
ATOM 482 HE1 PHE 36 3.747 3.312 29.678
1.00155.06 H
ATOM 483 HE2 PHE 36 5.911 1.647 26.361
1.00159.46 H
ATOM 484 HZ PHE 36 4.015 1.546 27.956
1.00183.25 H
ATOM 485 N THR 37 10.816 5.848 28.282 1.00
20.06 N
ATOM 486 CA THR 37 11.983 6.694 28.054 1.00
27.18 C
ATOM 487 C THR 37 12.514 6.489 26.639 1.00
41.87 C
ATOM 488 O THR 37 12.944 5.394 26.278
1.00169.86 O
ATOM 489 CB THR 37 13.080 6.355 29.067 1.00
26.52 C
ATOM 490 CG2 THR 37 12.550 6.571 30.485 1.00
75.69 C
ATOM 491 OG1 THR 37 13.467 4.999 28.906 1.00
81.95 O
ATOM 492 HN THR 37 10.767 4.975 27.840 1.00
18.47 H
ATOM 493 HA THR 37 11.703 7.731 28.175 1.00
38.80 H

```
ATOM    494  HB  THR    37       13.932   6.996  28.904  1.00
81.60           H
ATOM    495  HG1 THR    37       14.358   4.901  29.251
1.00188.80           H
ATOM    496 HG21 THR    37       13.376   6.580  31.181
1.00196.98           H
ATOM    497 HG22 THR    37       11.871   5.771  30.742
1.00196.23           H
ATOM    498 HG23 THR    37       12.027   7.515  30.535
1.00168.92           H
ATOM    499  N   GLU    38       12.476   7.552  25.845  1.00
26.53           N
ATOM    500  CA  GLU    38       12.951   7.487  24.465  1.00
30.29           C
ATOM    501  C   GLU    38       14.469   7.623  24.403  1.00
25.39           C
ATOM    502  O   GLU    38       15.102   8.096  25.347  1.00
51.60           O
ATOM    503  CB  GLU    38       12.303   8.600  23.638  1.00
54.82           C
ATOM    504  CG  GLU    38       10.804   8.330  23.504
1.00165.84           C
ATOM    505  CD  GLU    38       10.137   9.458  22.724
1.00292.92           C
ATOM    506  OE1 GLU    38        8.968   9.322  22.407
1.00414.19           O
ATOM    507  OE2 GLU    38       10.807  10.443  22.457
1.00537.65           O1-
ATOM    508  HN  GLU    38       12.118   8.393  26.191  1.00
84.50           H
ATOM    509  HA  GLU    38       12.669   6.535  24.042  1.00
32.15           H
ATOM    510  HB1 GLU    38       12.752   8.626  22.656
1.00144.82           H
ATOM    511  HB2 GLU    38       12.457   9.549  24.130
1.00106.49           H
ATOM    512  HG1 GLU    38       10.362   8.265  24.487
1.00303.31           H
ATOM    513  HG2 GLU    38       10.654   7.396  22.981
1.00323.99           H
ATOM    514  N   CYS    39       15.043   7.207  23.278  1.00
15.84           N
ATOM    515  CA  CYS    39       16.488   7.284  23.084  1.00
14.30           C
ATOM    516  C   CYS    39       16.943   8.747  23.083  1.00
22.31           C
ATOM    517  O   CYS    39       16.182   9.631  22.689  1.00
32.92           O
ATOM    518  CB  CYS    39       16.853   6.629  21.749  1.00
18.08           C
ATOM    519  SG  CYS    39       15.785   7.295  20.447  1.00
17.41           S
```

```
ATOM    520  HN  CYS    39      14.484   6.846  22.562  1.00
24.87         H
ATOM    521  HA  CYS    39      16.975   6.747  23.881  1.00
14.60         H
ATOM    522  HB1 CYS    39      16.714   5.561  21.818  1.00
27.71         H
ATOM    523  HB2 CYS    39      17.884   6.843  21.511  1.00
34.91         H
ATOM    524  N   PRO    40      18.154   9.030  23.509  1.00
27.27         N
ATOM    525  CA  PRO    40      18.676  10.427  23.541  1.00
47.66         C
ATOM    526  C   PRO    40      19.039  10.928  22.145  1.00
71.54         C
ATOM    527  O   PRO    40      19.635  10.205  21.346  1.00
87.70         O
ATOM    528  CB  PRO    40      19.915  10.321  24.438  1.00
53.85         C
ATOM    529  CG  PRO    40      20.409   8.928  24.226  1.00
46.04         C
ATOM    530  CD  PRO    40      19.159   8.070  24.001  1.00
25.70         C
ATOM    531  HA  PRO    40      17.954  11.085  23.997  1.00
58.04         H
ATOM    532  HB1 PRO    40      19.644  10.464  25.473  1.00
67.93         H
ATOM    533  HB2 PRO    40      20.665  11.045  24.141  1.00
70.99         H
ATOM    534  HG1 PRO    40      20.942   8.579  25.098  1.00
65.91         H
ATOM    535  HG2 PRO    40      21.054   8.892  23.355  1.00
54.99         H
ATOM    536  HD1 PRO    40      18.830   7.627  24.928  1.00
24.22         H
ATOM    537  HD2 PRO    40      19.352   7.310  23.259  1.00
26.94         H
ATOM    538  N   GLY    41      18.675  12.174  21.866
1.00105.24         N
ATOM    539  CA  GLY    41      18.959  12.791  20.569
1.00147.55         C
ATOM    540  C   GLY    41      20.150  13.737  20.669
1.00161.74         C
ATOM    541  O   GLY    41      20.460  14.462  19.723
1.00244.37         O
ATOM    542  HN  GLY    41      18.205  12.692  22.548
1.00121.79         H
ATOM    543  HA1 GLY    41      18.094  13.350  20.247
1.00184.40         H
ATOM    544  HA2 GLY    41      19.177  12.023  19.838
1.00157.34         H
ATOM    545  N   LEU    42      20.812  13.732  21.826
1.00162.48         N
```

ATOM 546 CA LEU 42 21.970 14.600 22.055
1.00191.48 C
ATOM 547 C LEU 42 23.259 13.784 22.067
1.00166.66 C
ATOM 548 O LEU 42 23.271 12.629 22.492
1.00251.38 O
ATOM 549 CB LEU 42 21.815 15.321 23.395
1.00307.99 C
ATOM 550 CG LEU 42 20.438 15.991 23.467
1.00452.28 C
ATOM 551 CD1 LEU 42 20.306 16.733 24.799
1.00681.24 C
ATOM 552 CD2 LEU 42 20.274 16.981 22.303
1.00527.46 C
ATOM 553 HN LEU 42 20.514 13.135 22.544
1.00195.47 H
ATOM 554 HA LEU 42 22.035 15.338 21.268
1.00229.77 H
ATOM 555 HB1 LEU 42 22.583 16.075 23.488
1.00342.49 H
ATOM 556 HB2 LEU 42 21.910 14.607 24.200
1.00335.33 H
ATOM 557 HG LEU 42 19.670 15.233 23.405
1.00427.79 H
ATOM 558 HD11 LEU 42 19.370 17.270 24.820
1.00909.71 H
ATOM 559 HD12 LEU 42 21.124 17.430 24.905
1.00706.80 H
ATOM 560 HD13 LEU 42 20.331 16.021 25.611
1.00858.82 H
ATOM 561 HD21 LEU 42 19.500 17.696 22.540
1.00631.69 H
ATOM 562 HD22 LEU 42 19.996 16.441 21.409
1.00628.06 H
ATOM 563 HD23 LEU 42 21.206 17.502 22.134
1.00656.75 H
ATOM 564 N THR 43 24.345 14.399 21.597
1.00178.52 N
ATOM 565 CA THR 43 25.648 13.734 21.551
1.00240.84 C
ATOM 566 C THR 43 26.766 14.705 21.928
1.00372.31 C
ATOM 567 O THR 43 27.590 15.075 21.091
1.00500.13 O
ATOM 568 CB THR 43 25.895 13.177 20.153
1.00354.13 C
ATOM 569 CG2 THR 43 25.579 14.241 19.097
1.00463.04 C
ATOM 570 OG1 THR 43 27.249 12.765 20.040
1.00508.50 O
ATOM 571 HN THR 43 24.268 15.321 21.274
1.00229.59 H

```
ATOM    572  HA  THR    43      25.654  12.908  22.247
1.00249.90          H
ATOM    573  HB  THR    43      25.251  12.328  20.006
1.00481.58          H
ATOM    574  HG1 THR    43      27.257  11.842  19.780
1.00624.31          H
ATOM    575 HG21 THR    43      24.510  14.301  18.956
1.00650.95          H
ATOM    576 HG22 THR    43      26.050  13.970  18.164
1.00580.73          H
ATOM    577 HG23 THR    43      25.953  15.200  19.422
1.00571.99          H
ATOM    578  N   PRO    44      26.805  15.116  23.167
1.00488.39          N
ATOM    579  CA  PRO    44      27.841  16.063  23.674
1.00760.13          C
ATOM    580  C   PRO    44      29.185  15.368  23.883
1.00735.36          C
ATOM    581  O   PRO    44      30.199  16.019  24.140
1.00999.99          O
ATOM    582  CB  PRO    44      27.248  16.554  24.999
1.00999.99          C
ATOM    583  CG  PRO    44      26.420  15.408  25.486
1.00856.94          C
ATOM    584  CD  PRO    44      25.866  14.719  24.232
1.00548.09          C
ATOM    585  HA  PRO    44      27.953  16.893  22.996
1.00939.00          H
ATOM    586  HB1 PRO    44      26.621  17.418  24.833
1.00999.99          H
ATOM    587  HB2 PRO    44      28.035  16.789  25.706
1.00999.99          H
ATOM    588  HG1 PRO    44      25.604  15.765  26.098
1.00999.99          H
ATOM    589  HG2 PRO    44      27.036  14.719  26.052
1.00853.53          H
ATOM    590  HD1 PRO    44      24.873  15.075  24.004
1.00602.45          H
ATOM    591  HD2 PRO    44      25.867  13.644  24.357
1.00463.32          H
ATOM    592  N   ILE    45      29.179  14.043  23.780
1.00518.33          N
ATOM    593  CA  ILE    45      30.398  13.268  23.967
1.00582.79          C
ATOM    594  C   ILE    45      31.375  13.518  22.822
1.00795.93          C
ATOM    595  O   ILE    45      31.013  13.414  21.651
1.00898.75          O
ATOM    596  CB  ILE    45      30.062  11.778  24.033
1.00474.41          C
ATOM    597  CG1 ILE    45      29.167  11.512  25.247
1.00516.90          C
```

```
ATOM    598  CG2 ILE    45       31.354  10.968  24.166
1.00760.75          C
ATOM    599  CD1 ILE    45       28.616  10.086  25.174
1.00566.81          C
ATOM    600  HN  ILE    45       28.339  13.581  23.579
1.00395.72          H
ATOM    601  HA  ILE    45       30.859  13.563  24.896
1.00711.06          H
ATOM    602  HB  ILE    45       29.544  11.484  23.132
1.00477.81          H
ATOM    603 HG11 ILE    45       28.346  12.213  25.250
1.00624.28          H
ATOM    604 HG12 ILE    45       29.745  11.629  26.153
1.00744.84          H
ATOM    605 HG21 ILE    45       31.969  11.399  24.942
1.00920.57          H
ATOM    606 HG22 ILE    45       31.889  10.990  23.229
1.00999.99          H
ATOM    607 HG23 ILE    45       31.114   9.947  24.422
1.00865.46          H
ATOM    608 HD11 ILE    45       27.879   9.944  25.951
1.00658.89          H
ATOM    609 HD12 ILE    45       29.423   9.381  25.309
1.00734.30          H
ATOM    610 HD13 ILE    45       28.157   9.927  24.209
1.00675.84          H
ATOM    611  N   ALA    46       32.614  13.849  23.170
1.00999.99          N
ATOM    612  CA  ALA    46       33.636  14.113  22.164
1.00999.99          C
ATOM    613  C   ALA    46       35.023  14.126  22.798
1.00999.99          C
ATOM    614  CB  ALA    46       33.369  15.459  21.487
1.00999.99          C
ATOM    615  OT1 ALA    46       35.098  14.266  24.008
1.00999.99          O
ATOM    616  OT2 ALA    46       35.990  13.997  22.065
1.00999.99          O
ATOM    617  HN  ALA    46       32.845  13.918  24.120
1.00999.99          H
ATOM    618  HA  ALA    46       33.600  13.334  21.416
1.00999.99          H
ATOM    619  HB1 ALA    46       33.655  16.259  22.153
1.00999.99          H
ATOM    620  HB2 ALA    46       32.318  15.542  21.254
1.00999.99          H
ATOM    621  HB3 ALA    46       33.946  15.524  20.575
1.00999.99          H
ENDMDL
TER
MODEL        2
ATOM      1  N   GLY     1       23.748  -0.455  11.093
```

```
1.00999.99           N
ATOM      2  CA  GLY     1       24.412  -0.156  12.392
1.00999.99           C
ATOM      3  C   GLY     1       23.820  -1.040  13.483
1.00999.99           C
ATOM      4  O   GLY     1       23.080  -1.983  13.199
1.00999.99           O
ATOM      5  HA1 GLY     1       24.255   0.880  12.648
1.00999.99           H
ATOM      6  HA2 GLY     1       25.473  -0.349  12.306
1.00999.99           H
ATOM      7  HT1 GLY     1       23.780  -1.478  10.913
1.00999.99           H
ATOM      8  HT2 GLY     1       24.242   0.047  10.327
1.00999.99           H
ATOM      9  HT3 GLY     1       22.757  -0.143  11.129
1.00999.99           H
ATOM     10  N   LEU     2       24.149  -0.731  14.734
1.00999.99           N
ATOM     11  CA  LEU     2       23.645  -1.505  15.861
1.00895.59           C
ATOM     12  C   LEU     2       22.125  -1.408  15.940
1.00532.27           C
ATOM     13  O   LEU     2       21.447  -2.390  16.238
1.00625.21           O
ATOM     14  CB  LEU     2       24.266  -0.999  17.168
1.00999.99           C
ATOM     15  CG  LEU     2       25.721  -1.469  17.269
1.00999.99           C
ATOM     16  CD1 LEU     2       26.550  -0.865  16.129
1.00999.99           C
ATOM     17  CD2 LEU     2       26.299  -1.025  18.616
1.00999.99           C
ATOM     18  HN  LEU     2       24.743   0.032  14.899
1.00999.99           H
ATOM     19  HA  LEU     2       23.918  -2.536  15.723
1.00890.87           H
ATOM     20  HB1 LEU     2       23.706  -1.388  18.006
1.00924.07           H
ATOM     21  HB2 LEU     2       24.235   0.081  17.186
1.00999.99           H
ATOM     22  HG  LEU     2       25.754  -2.548  17.202
1.00999.99           H
ATOM     23 HD11 LEU     2       26.384  -1.428  15.224
1.00999.99           H
ATOM     24 HD12 LEU     2       27.600  -0.900  16.385
1.00999.99           H
ATOM     25 HD13 LEU     2       26.255   0.163  15.972
1.00999.99           H
ATOM     26 HD21 LEU     2       26.458   0.042  18.604
1.00999.99           H
ATOM     27 HD22 LEU     2       27.239  -1.529  18.788
```

```
1.00999.99            H
ATOM     28 HD23 LEU     2       25.606  -1.278  19.406
1.00999.99            H
ATOM     29  N   CYS     3       21.598  -0.220  15.665
1.00271.28            N
ATOM     30  CA  CYS     3       20.155  -0.012  15.704
1.00104.50            C
ATOM     31  C   CYS     3       19.510  -0.526  14.420  1.00
90.98           C
ATOM     32  O   CYS     3       19.640   0.090  13.362
1.00207.28            O
ATOM     33  CB  CYS     3       19.836   1.478  15.868  1.00
37.83           C
ATOM     34  SG  CYS     3       20.487   2.078  17.447  1.00
81.56           S
ATOM     35  HN  CYS     3       22.188   0.524  15.429
1.00296.55            H
ATOM     36  HA  CYS     3       19.742  -0.548  16.546
1.00166.45            H
ATOM     37  HB1 CYS     3       18.766   1.621  15.842  1.00
40.38           H
ATOM     38  HB2 CYS     3       20.291   2.032  15.060  1.00
98.45           H
ATOM     39  N   SER     4       18.815  -1.653  14.521  1.00
85.31           N
ATOM     40  CA  SER     4       18.150  -2.236  13.359  1.00
92.94           C
ATOM     41  C   SER     4       17.120  -1.266  12.814  1.00
67.37           C
ATOM     42  O   SER     4       16.986  -1.086  11.604
1.00102.28            O
ATOM     43  CB  SER     4       17.431  -3.520  13.757
1.00123.83            C
ATOM     44  OG  SER     4       16.908  -4.144  12.591
1.00178.89            O
ATOM     45  HN  SER     4       18.742  -2.098  15.391
1.00163.03            H
ATOM     46  HA  SER     4       18.881  -2.456  12.598
1.00129.78            H
ATOM     47  HB1 SER     4       16.618  -3.273  14.433
1.00105.26            H
ATOM     48  HB2 SER     4       18.117  -4.192  14.245
1.00156.87            H
ATOM     49  HG  SER     4       17.530  -4.819  12.310
1.00218.16            H
ATOM     50  N   GLU     5       16.378  -0.661  13.734  1.00
41.67           N
ATOM     51  CA  GLU     5       15.327   0.280  13.381  1.00
41.21           C
ATOM     52  C   GLU     5       15.250   1.397  14.417  1.00
41.92           C
ATOM     53  O   GLU     5       16.174   1.579  15.212  1.00
```

```
74.72           O
ATOM     54  CB  GLU     5       13.994  -0.459  13.312  1.00
49.24           C
ATOM     55  CG  GLU     5       13.737  -1.149  14.645  1.00
55.48           C
ATOM     56  CD  GLU     5       12.526  -2.071  14.533  1.00
92.06           C
ATOM     57  OE1 GLU     5       11.950  -2.133  13.459
1.00206.49           O
ATOM     58  OE2 GLU     5       12.194  -2.702  15.523
1.00197.24           O1-
ATOM     59  HN  GLU     5       16.533  -0.868  14.679  1.00
45.52           H
ATOM     60  HA  GLU     5       15.536   0.706  12.420  1.00
60.63           H
ATOM     61  HB1 GLU     5       14.029  -1.198  12.526  1.00
61.59           H
ATOM     62  HB2 GLU     5       13.205   0.242  13.111  1.00
63.66           H
ATOM     63  HG1 GLU     5       13.550  -0.404  15.405  1.00
58.03           H
ATOM     64  HG2 GLU     5       14.607  -1.723  14.911  1.00
57.77           H
ATOM     65  N   ASN     6       14.153   2.143  14.404  1.00
50.73           N
ATOM     66  CA  ASN     6       13.982   3.236  15.353  1.00
71.04           C
ATOM     67  C   ASN     6       14.023   2.704  16.781  1.00
65.17           C
ATOM     68  O   ASN     6       14.600   3.328  17.671  1.00
94.86           O
ATOM     69  CB  ASN     6       12.648   3.942  15.106  1.00
94.83           C
ATOM     70  CG  ASN     6       12.715   4.743  13.809
1.00160.94           C
ATOM     71  ND2 ASN     6       11.609   5.086  13.208
1.00244.88           N
ATOM     72  OD1 ASN     6       13.802   5.063  13.331
1.00219.73           O
ATOM     73  HN  ASN     6       13.448   1.955  13.750  1.00
72.81           H
ATOM     74  HA  ASN     6       14.784   3.946  15.220  1.00
96.02           H
ATOM     75  HB1 ASN     6       12.439   4.612  15.927
1.00111.09           H
ATOM     76  HB2 ASN     6       11.861   3.207  15.033  1.00
98.13           H
ATOM     77 HD21 ASN     6       10.742   4.830  13.590
1.00272.43           H
ATOM     78 HD22 ASN     6       11.643   5.600  12.375
1.00336.78           H
ATOM     79  N   GLY     7       13.410   1.538  16.989  1.00
```

```
51.80           N
ATOM     80  CA  GLY     7      13.378   0.908  18.313  1.00
71.47           C
ATOM     81  C   GLY     7      13.973  -0.493  18.255  1.00
41.04           C
ATOM     82  O   GLY     7      13.280  -1.485  18.468  1.00
44.54           O
ATOM     83  HN  GLY     7      12.972   1.090  16.237  1.00
45.49           H
ATOM     84  HA1 GLY     7      12.360   0.838  18.646
1.00103.52           H
ATOM     85  HA2 GLY     7      13.943   1.504  19.018
1.00108.67           H
ATOM     86  N   ASP     8      15.265  -0.559  17.961  1.00
27.00           N
ATOM     87  CA  ASP     8      15.955  -1.842  17.872  1.00
14.15           C
ATOM     88  C   ASP     8      15.900  -2.573  19.206  1.00
9.37           C
ATOM     89  O   ASP     8      15.681  -3.783  19.254  1.00
14.61           O
ATOM     90  CB  ASP     8      17.413  -1.627  17.465  1.00
15.06           C
ATOM     91  CG  ASP     8      18.096  -2.975  17.256  1.00
21.22           C
ATOM     92  OD1 ASP     8      17.493  -3.981  17.588
1.00119.56           O
ATOM     93  OD2 ASP     8      19.214  -2.980  16.766
1.00133.27           O1-
ATOM     94  HN  ASP     8      15.760   0.269  17.801  1.00
35.63           H
ATOM     95  HA  ASP     8      15.471  -2.445  17.125  1.00
20.92           H
ATOM     96  HB1 ASP     8      17.926  -1.085  18.244  1.00
29.39           H
ATOM     97  HB2 ASP     8      17.450  -1.059  16.547  1.00
44.14           H
ATOM     98  N   CYS     9      16.095  -1.830  20.288  1.00
5.93           N
ATOM     99  CA  CYS     9      16.059  -2.418  21.620  1.00
7.54           C
ATOM    100  C   CYS     9      14.685  -3.020  21.892  1.00
14.60           C
ATOM    101  O   CYS     9      14.567  -4.108  22.454  1.00
25.78           O
ATOM    102  CB  CYS     9      16.382  -1.359  22.665  1.00
6.63           C
ATOM    103  SG  CYS     9      18.135  -0.958  22.541  1.00
10.91           S
ATOM    104  HN  CYS     9      16.259  -0.870  20.186  1.00
6.51           H
ATOM    105  HA  CYS     9      16.808  -3.190  21.678  1.00
```

```
11.12           H
ATOM    106  HB1 CYS     9       16.171  -1.737  23.652  1.00
11.28           H
ATOM    107  HB2 CYS     9       15.792  -0.473  22.481  1.00
5.04           H
ATOM    108  N   ALA    10       13.653  -2.300  21.472  1.00
16.26           N
ATOM    109  CA  ALA    10       12.279  -2.756  21.649  1.00
32.04           C
ATOM    110  C   ALA    10       11.332  -1.896  20.812  1.00
45.47           C
ATOM    111  O   ALA    10       11.719  -0.840  20.315
1.00119.04           O
ATOM    112  CB  ALA    10       11.883  -2.690  23.131  1.00
30.10           C
ATOM    113  HN  ALA    10       13.819  -1.446  21.022  1.00
12.81           H
ATOM    114  HA  ALA    10       12.208  -3.779  21.313  1.00
47.14           H
ATOM    115  HB1 ALA    10       12.743  -2.921  23.741  1.00
84.37           H
ATOM    116  HB2 ALA    10       11.100  -3.409  23.327
1.00118.20           H
ATOM    117  HB3 ALA    10       11.527  -1.701  23.371
1.00117.32           H
ATOM    118  N   ALA    11       10.098  -2.359  20.653  1.00
30.62           N
ATOM    119  CA  ALA    11        9.116  -1.619  19.866  1.00
35.24           C
ATOM    120  C   ALA    11        8.862  -0.238  20.469  1.00
22.78           C
ATOM    121  O   ALA    11        8.748   0.751  19.745  1.00
53.33           O
ATOM    122  CB  ALA    11        7.803  -2.400  19.806  1.00
60.49           C
ATOM    123  HN  ALA    11        9.843  -3.209  21.067  1.00
50.37           H
ATOM    124  HA  ALA    11        9.493  -1.497  18.862  1.00
41.70           H
ATOM    125  HB1 ALA    11        7.979  -3.367  19.359
1.00157.06           H
ATOM    126  HB2 ALA    11        7.086  -1.854  19.211
1.00148.83           H
ATOM    127  HB3 ALA    11        7.415  -2.530  20.807
1.00137.06           H
ATOM    128  N   ASP    12        8.769  -0.179  21.795  1.00
18.98           N
ATOM    129  CA  ASP    12        8.521   1.089  22.482  1.00
31.05           C
ATOM    130  C   ASP    12        9.824   1.846  22.723  1.00
22.98           C
ATOM    131  O   ASP    12        9.837   3.076  22.773  1.00
```

```
37.14           O
ATOM    132  CB  ASP    12       7.832   0.823  23.821  1.00
48.35           C
ATOM    133  CG  ASP    12       6.417   0.303  23.586
1.00104.83           C
ATOM    134  OD1 ASP    12       5.935   0.439  22.474
1.00258.73           O
ATOM    135  OD2 ASP    12       5.837  -0.223  24.521
1.00213.46           O1-
ATOM    136  HN  ASP    12       8.864  -1.001  22.320  1.00
36.19           H
ATOM    137  HA  ASP    12       7.870   1.699  21.875  1.00
49.76           H
ATOM    138  HB1 ASP    12       7.784   1.741  24.388  1.00
95.71           H
ATOM    139  HB2 ASP    12       8.396   0.088  24.376  1.00
54.37           H
ATOM    140  N   GLU    13      10.918   1.106  22.878  1.00
17.60           N
ATOM    141  CA  GLU    13      12.221   1.723  23.121  1.00
12.01           C
ATOM    142  C   GLU    13      12.796   2.317  21.839  1.00
10.07           C
ATOM    143  O   GLU    13      12.395   1.949  20.735  1.00
12.81           O
ATOM    144  CB  GLU    13      13.190   0.686  23.687  1.00
11.74           C
ATOM    145  CG  GLU    13      12.697   0.221  25.059  1.00
13.49           C
ATOM    146  CD  GLU    13      12.862   1.343  26.078
1.00142.36           C
ATOM    147  OE1 GLU    13      13.575   2.285  25.780
1.00339.06           O
ATOM    148  OE2 GLU    13      12.272   1.243  27.140
1.00335.28           O1-
ATOM    149  HN  GLU    13      10.849   0.130  22.833  1.00
28.36           H
ATOM    150  HA  GLU    13      12.100   2.511  23.847  1.00
13.06           H
ATOM    151  HB1 GLU    13      14.169   1.128  23.788  1.00
13.76           H
ATOM    152  HB2 GLU    13      13.245  -0.159  23.016  1.00
9.94           H
ATOM    153  HG1 GLU    13      13.269  -0.636  25.376  1.00
66.02           H
ATOM    154  HG2 GLU    13      11.652  -0.047  24.993  1.00
56.23           H
ATOM    155  N   CYS    14      13.745   3.243  21.999  1.00
9.12           N
ATOM    156  CA  CYS    14      14.390   3.901  20.860  1.00
10.69           C
ATOM    157  C   CYS    14      15.838   3.440  20.731  1.00
```

9.56 C
ATOM 158 O CYS 14 16.458 3.050 21.717 1.00
13.62 O
ATOM 159 CB CYS 14 14.360 5.420 21.050 1.00
13.96 C
ATOM 160 SG CYS 14 15.319 6.212 19.733 1.00
42.96 S
ATOM 161 HN CYS 14 14.020 3.490 22.908 1.00
9.76 H
ATOM 162 HA CYS 14 13.861 3.655 19.949 1.00
15.54 H
ATOM 163 HB1 CYS 14 14.788 5.669 22.008 1.00
50.72 H
ATOM 164 HB2 CYS 14 13.338 5.767 21.011 1.00
46.13 H
ATOM 165 N CYS 15 16.370 3.490 19.511 1.00
9.60 N
ATOM 166 CA CYS 15 17.751 3.080 19.260 1.00
9.28 C
ATOM 167 C CYS 15 18.350 3.970 18.182 1.00
9.92 C
ATOM 168 O CYS 15 17.771 4.126 17.107 1.00
13.31 O
ATOM 169 CB CYS 15 17.793 1.619 18.799 1.00
12.90 C
ATOM 170 SG CYS 15 19.499 1.006 18.848 1.00
39.11 S
ATOM 171 HN CYS 15 15.825 3.816 18.764 1.00
13.12 H
ATOM 172 HA CYS 15 18.331 3.182 20.166 1.00
8.26 H
ATOM 173 HB1 CYS 15 17.418 1.554 17.788 1.00
24.82 H
ATOM 174 HB2 CYS 15 17.178 1.020 19.450 1.00
27.31 H
ATOM 175 N VAL 16 19.505 4.562 18.468 1.00
9.56 N
ATOM 176 CA VAL 16 20.155 5.441 17.502 1.00
11.99 C
ATOM 177 C VAL 16 21.670 5.358 17.636 1.00
7.73 C
ATOM 178 O VAL 16 22.216 5.500 18.731 1.00
8.83 O
ATOM 179 CB VAL 16 19.685 6.878 17.725 1.00
18.37 C
ATOM 180 CG1 VAL 16 20.154 7.367 19.098 1.00
39.61 C
ATOM 181 CG2 VAL 16 20.265 7.777 16.633
1.00115.21 C
ATOM 182 HN VAL 16 19.927 4.409 19.341 1.00
9.76 H
ATOM 183 HA VAL 16 19.880 5.138 16.500 1.00

```
17.27           H
ATOM    184  HB  VAL    16       18.607   6.912  17.683  1.00
52.63           H
ATOM    185 HG11 VAL    16       19.980   6.597  19.834
1.00128.07           H
ATOM    186 HG12 VAL    16       19.602   8.255  19.370
1.00154.13           H
ATOM    187 HG13 VAL    16       21.209   7.598  19.060
1.00135.74           H
ATOM    188 HG21 VAL    16       19.884   8.780  16.754
1.00229.05           H
ATOM    189 HG22 VAL    16       19.977   7.396  15.665
1.00261.82           H
ATOM    190 HG23 VAL    16       21.342   7.789  16.710
1.00210.07           H
ATOM    191  N   ASP    17       22.344   5.121  16.513  1.00
14.34           N
ATOM    192  CA  ASP    17       23.802   5.017  16.502  1.00
11.90           C
ATOM    193  C   ASP    17       24.432   6.323  16.038  1.00
13.15           C
ATOM    194  O   ASP    17       24.029   6.897  15.026  1.00
25.96           O
ATOM    195  CB  ASP    17       24.235   3.889  15.563  1.00
20.80           C
ATOM    196  CG  ASP    17       23.840   2.540  16.150  1.00
28.72           C
ATOM    197  OD1 ASP    17       23.839   1.572  15.407
1.00135.42           O
ATOM    198  OD2 ASP    17       23.540   2.494  17.332
1.00114.17           O1-
ATOM    199  HN  ASP    17       21.850   5.013  15.673  1.00
28.29           H
ATOM    200  HA  ASP    17       24.155   4.791  17.500  1.00
10.49           H
ATOM    201  HB1 ASP    17       25.310   3.922  15.438  1.00
22.42           H
ATOM    202  HB2 ASP    17       23.758   4.019  14.603  1.00
29.52           H
ATOM    203  N   THR    18       25.440   6.777  16.779  1.00
11.55           N
ATOM    204  CA  THR    18       26.155   8.009  16.446  1.00
17.10           C
ATOM    205  C   THR    18       27.633   7.706  16.270  1.00
10.63           C
ATOM    206  O   THR    18       28.120   6.682  16.741  1.00
6.49           O
ATOM    207  CB  THR    18       25.980   9.044  17.557  1.00
28.52           C
ATOM    208  CG2 THR    18       24.544   9.566  17.559  1.00
45.38           C
ATOM    209  OG1 THR    18       26.276   8.448  18.809  1.00
```

```
26.49           O
ATOM    210  HN  THR    18       25.718   6.263  17.567  1.00
13.96           H
ATOM    211  HA  THR    18       25.769   8.417  15.522  1.00
26.43           H
ATOM    212  HB  THR    18       26.654   9.869  17.386  1.00
37.43           H
ATOM    213  HG1 THR    18       25.994   7.529  18.773  1.00
71.62           H
ATOM    214 HG21 THR    18       24.368  10.132  16.655
1.00100.44           H
ATOM    215 HG22 THR    18       24.396  10.203  18.417
1.00129.42           H
ATOM    216 HG23 THR    18       23.858   8.733  17.603
1.00156.21           H
ATOM    217  N   VAL    19       28.340   8.606  15.602  1.00
16.32           N
ATOM    218  CA  VAL    19       29.755   8.444  15.374  1.00
13.34           C
ATOM    219  C   VAL    19       30.268   9.621  14.572  1.00
25.88           C
ATOM    220  O   VAL    19       29.846   9.875  13.445  1.00
42.11           O
ATOM    221  CB  VAL    19       30.068   7.141  14.642  1.00
15.48           C
ATOM    222  CG1 VAL    19       29.181   7.004  13.399  1.00
29.06           C
ATOM    223  CG2 VAL    19       31.544   7.146  14.222  1.00
20.67           C
ATOM    224  HN  VAL    19       27.907   9.412  15.267  1.00
26.89           H
ATOM    225  HA  VAL    19       30.256   8.431  16.331  1.00
8.08           H
ATOM    226  HB  VAL    19       29.888   6.312  15.306  1.00
11.49           H
ATOM    227 HG11 VAL    19       29.253   5.995  13.017
1.00137.43           H
ATOM    228 HG12 VAL    19       29.511   7.698  12.642  1.00
93.29           H
ATOM    229 HG13 VAL    19       28.156   7.215  13.660
1.00108.69           H
ATOM    230 HG21 VAL    19       31.855   6.144  13.973  1.00
99.42           H
ATOM    231 HG22 VAL    19       32.149   7.521  15.040
1.00111.36           H
ATOM    232 HG23 VAL    19       31.670   7.789  13.364  1.00
77.11           H
ATOM    233  N   PHE    20       31.172  10.331  15.191  1.00
24.58           N
ATOM    234  CA  PHE    20       31.780  11.509  14.600  1.00
40.34           C
ATOM    235  C   PHE    20       33.133  11.174  13.985  1.00
```

40.37 C
ATOM 236 O PHE 20 33.325 11.312 12.777 1.00
66.02 O
ATOM 237 CB PHE 20 31.942 12.596 15.680 1.00
48.79 C
ATOM 238 CG PHE 20 32.118 11.976 17.060 1.00
35.75 C
ATOM 239 CD1 PHE 20 31.068 11.254 17.667 1.00
30.20 C
ATOM 240 CD2 PHE 20 33.329 12.140 17.747 1.00
40.95 C
ATOM 241 CE1 PHE 20 31.243 10.705 18.941 1.00
30.41 C
ATOM 242 CE2 PHE 20 33.498 11.586 19.021 1.00
45.84 C
ATOM 243 CZ PHE 20 32.455 10.869 19.617 1.00
40.81 C
ATOM 244 HN PHE 20 31.429 10.061 16.087 1.00
16.73 H
ATOM 245 HA PHE 20 31.135 11.892 13.818 1.00
59.91 H
ATOM 246 HB1 PHE 20 31.070 13.206 15.685 1.00
67.15 H
ATOM 247 HB2 PHE 20 32.798 13.217 15.453 1.00
58.17 H
ATOM 248 HD1 PHE 20 30.127 11.112 17.152 1.00
33.34 H
ATOM 249 HD2 PHE 20 34.137 12.692 17.290 1.00
49.35 H
ATOM 250 HE1 PHE 20 30.439 10.154 19.401 1.00
30.61 H
ATOM 251 HE2 PHE 20 34.434 11.712 19.545 1.00
61.27 H
ATOM 252 HZ PHE 20 32.587 10.443 20.602 1.00
52.66 H
ATOM 253 N GLU 21 34.077 10.769 14.825 1.00
41.31 N
ATOM 254 CA GLU 21 35.415 10.457 14.373 1.00
56.16 C
ATOM 255 C GLU 21 35.695 8.961 14.449 1.00
58.61 C
ATOM 256 O GLU 21 34.802 8.160 14.722
1.00201.19 O
ATOM 257 CB GLU 21 36.370 11.219 15.263 1.00
57.37 C
ATOM 258 CG GLU 21 36.278 10.677 16.687
1.00193.56 C
ATOM 259 CD GLU 21 37.025 11.601 17.644
1.00304.62 C
ATOM 260 OE1 GLU 21 37.664 12.524 17.167
1.00451.81 O
ATOM 261 OE2 GLU 21 36.942 11.376 18.840

```
1.00442.88           O1-
ATOM    262  HN  GLU    21       33.886  10.705  15.781  1.00
50.82          H
ATOM    263  HA  GLU    21       35.551  10.791  13.353  1.00
85.72          H
ATOM    264  HB1 GLU    21       36.087  12.260  15.262  1.00
46.37          H
ATOM    265  HB2 GLU    21       37.369  11.110  14.896
1.00131.81           H
ATOM    266  HG1 GLU    21       36.716   9.696  16.722
1.00314.91           H
ATOM    267  HG2 GLU    21       35.244  10.613  16.980
1.00266.92           H
ATOM    268  N   GLY    22       36.946   8.598  14.190  1.00
86.70          N
ATOM    269  CA  GLY    22       37.359   7.191  14.209
1.00104.66           C
ATOM    270  C   GLY    22       37.944   6.788  15.562  1.00
71.50          C
ATOM    271  O   GLY    22       38.152   5.603  15.824  1.00
89.87          O
ATOM    272  HN  GLY    22       37.606   9.292  13.973
1.00214.64           H
ATOM    273  HA1 GLY    22       38.107   7.036  13.446
1.00152.47           H
ATOM    274  HA2 GLY    22       36.504   6.564  13.995
1.00118.15           H
ATOM    275  N   ASP    23       38.216   7.770  16.411  1.00
44.68          N
ATOM    276  CA  ASP    23       38.789   7.489  17.726  1.00
43.33          C
ATOM    277  C   ASP    23       37.852   6.618  18.554  1.00
37.55          C
ATOM    278  O   ASP    23       38.296   5.717  19.266  1.00
61.37          O
ATOM    279  CB  ASP    23       39.058   8.796  18.475  1.00
40.74          C
ATOM    280  CG  ASP    23       40.222   9.540  17.827
1.00141.86           C
ATOM    281  OD1 ASP    23       40.920   8.931  17.032
1.00328.18           O
ATOM    282  OD2 ASP    23       40.398  10.706  18.135
1.00304.58           O1-
ATOM    283  HN  ASP    23       38.041   8.697  16.149  1.00
40.86          H
ATOM    284  HA  ASP    23       39.721   6.968  17.593  1.00
72.12          H
ATOM    285  HB1 ASP    23       39.306   8.573  19.501  1.00
94.15          H
ATOM    286  HB2 ASP    23       38.175   9.414  18.448  1.00
93.26          H
ATOM    287  N   MET    24       36.557   6.897  18.464  1.00
```

23.24 N
ATOM 288 CA MET 24 35.569 6.133 19.220 1.00
32.50 C
ATOM 289 C MET 24 34.182 6.265 18.597 1.00
25.13 C
ATOM 290 O MET 24 33.950 7.118 17.742 1.00
54.40 O
ATOM 291 CB MET 24 35.531 6.622 20.670 1.00
47.36 C
ATOM 292 CG MET 24 35.113 8.094 20.707
1.00151.73 C
ATOM 293 SD MET 24 35.199 8.710 22.409
1.00209.23 S
ATOM 294 CE MET 24 33.783 7.799 23.075
1.00243.29 C
ATOM 295 HN MET 24 36.264 7.629 17.885 1.00
17.32 H
ATOM 296 HA MET 24 35.853 5.090 19.214 1.00
48.94 H
ATOM 297 HB1 MET 24 36.512 6.519 21.110
1.00124.40 H
ATOM 298 HB2 MET 24 34.821 6.031 21.230
1.00166.96 H
ATOM 299 HG1 MET 24 34.102 8.194 20.339
1.00331.71 H
ATOM 300 HG2 MET 24 35.778 8.673 20.082
1.00302.30 H
ATOM 301 HE1 MET 24 33.432 8.289 23.972
1.00340.44 H
ATOM 302 HE2 MET 24 34.080 6.791 23.313
1.00373.88 H
ATOM 303 HE3 MET 24 32.991 7.773 22.338
1.00386.81 H
ATOM 304 N VAL 25 33.260 5.407 19.041 1.00
22.44 N
ATOM 305 CA VAL 25 31.884 5.417 18.537 1.00
15.37 C
ATOM 306 C VAL 25 30.895 5.600 19.683 1.00
17.47 C
ATOM 307 O VAL 25 31.096 5.093 20.786 1.00
29.52 O
ATOM 308 CB VAL 25 31.577 4.107 17.801 1.00
23.36 C
ATOM 309 CG1 VAL 25 30.078 4.046 17.451 1.00
58.39 C
ATOM 310 CG2 VAL 25 32.409 4.045 16.515 1.00
55.25 C
ATOM 311 HN VAL 25 33.511 4.753 19.726 1.00
48.11 H
ATOM 312 HA VAL 25 31.758 6.242 17.843 1.00
9.25 H
ATOM 313 HB VAL 25 31.831 3.271 18.436 1.00

```
52.45           H
ATOM    314 HG11 VAL    25      29.720   5.037  17.209
1.00171.07           H
ATOM    315 HG12 VAL    25      29.527   3.668  18.298
1.00166.22           H
ATOM    316 HG13 VAL    25      29.924   3.391  16.604
1.00134.76           H
ATOM    317 HG21 VAL    25      32.089   3.202  15.920
1.00141.88           H
ATOM    318 HG22 VAL    25      33.453   3.933  16.766
1.00184.50           H
ATOM    319 HG23 VAL    25      32.268   4.956  15.951
1.00133.90           H
ATOM    320  N   THR    26      29.827   6.343  19.405  1.00
13.60           N
ATOM    321  CA  THR    26      28.790   6.623  20.397  1.00
21.19           C
ATOM    322  C   THR    26      27.440   6.062  19.951  1.00
16.55           C
ATOM    323  O   THR    26      27.048   6.207  18.793  1.00
11.43           O
ATOM    324  CB  THR    26      28.682   8.135  20.580  1.00
27.07           C
ATOM    325  CG2 THR    26      27.555   8.464  21.563  1.00
44.39           C
ATOM    326  OG1 THR    26      29.914   8.629  21.084  1.00
34.75           O
ATOM    327  HN  THR    26      29.735   6.721  18.506  1.00
10.81           H
ATOM    328  HA  THR    26      29.058   6.177  21.344  1.00
33.16           H
ATOM    329  HB  THR    26      28.473   8.591  19.621  1.00
20.60           H
ATOM    330  HG1 THR    26      30.128   8.134  21.877  1.00
77.72           H
ATOM    331 HG21 THR    26      27.592   9.515  21.811
1.00126.99           H
ATOM    332 HG22 THR    26      27.681   7.878  22.462
1.00108.32           H
ATOM    333 HG23 THR    26      26.602   8.234  21.115
1.00151.99           H
ATOM    334  N   ARG    27      26.735   5.421  20.883  1.00
22.38           N
ATOM    335  CA  ARG    27      25.422   4.836  20.591  1.00
20.21           C
ATOM    336  C   ARG    27      24.492   5.016  21.787  1.00
16.96           C
ATOM    337  O   ARG    27      24.952   5.158  22.920  1.00
20.06           O
ATOM    338  CB  ARG    27      25.578   3.346  20.278  1.00
22.14           C
ATOM    339  CG  ARG    27      26.050   2.604  21.533
```

1.00124.30 C
ATOM 340 CD ARG 27 26.438 1.171 21.163
1.00109.38 C
ATOM 341 NE ARG 27 26.819 0.425 22.358
1.00227.73 N
ATOM 342 CZ ARG 27 28.036 0.536 22.880
1.00426.12 C
ATOM 343 NH1 ARG 27 28.352 -0.144 23.947
1.00767.09 N1+
ATOM 344 NH2 ARG 27 28.915 1.325 22.325
1.00581.78 N
ATOM 345 HN ARG 27 27.102 5.340 21.787 1.00
30.75 H
ATOM 346 HA ARG 27 24.989 5.332 19.732 1.00
23.13 H
ATOM 347 HB1 ARG 27 26.306 3.216 19.492 1.00
87.46 H
ATOM 348 HB2 ARG 27 24.628 2.944 19.958
1.00103.36 H
ATOM 349 HG1 ARG 27 25.252 2.578 22.261
1.00281.97 H
ATOM 350 HG2 ARG 27 26.905 3.113 21.952
1.00276.19 H
ATOM 351 HD1 ARG 27 27.269 1.194 20.473
1.00183.60 H
ATOM 352 HD2 ARG 27 25.599 0.684 20.692
1.00142.93 H
ATOM 353 HE ARG 27 26.165 -0.168 22.782
1.00372.53 H
ATOM 354 HH11 ARG 27 27.679 -0.749 24.372
1.00910.59 H
ATOM 355 HH12 ARG 27 29.268 -0.062 24.339
1.00999.99 H
ATOM 356 HH21 ARG 27 28.673 1.846 21.506
1.00532.54 H
ATOM 357 HH22 ARG 27 29.831 1.407 22.717
1.00948.84 H
ATOM 358 N SER 28 23.180 5.017 21.535 1.00
14.60 N
ATOM 359 CA SER 28 22.203 5.188 22.613 1.00
14.92 C
ATOM 360 C SER 28 20.991 4.286 22.401 1.00
12.12 C
ATOM 361 O SER 28 20.608 3.994 21.268 1.00
13.41 O
ATOM 362 CB SER 28 21.748 6.647 22.671 1.00
23.26 C
ATOM 363 OG SER 28 22.867 7.477 22.952
1.00146.32 O
ATOM 364 HN SER 28 22.864 4.903 20.612 1.00
15.23 H
ATOM 365 HA SER 28 22.661 4.932 23.558 1.00

```
15.44            H
ATOM    366  HB1 SER    28       20.999   6.759  23.445  1.00
88.73            H
ATOM    367  HB2 SER    28       21.323   6.932  21.724
1.00124.68            H
ATOM    368  HG  SER    28       23.650   7.046  22.601
1.00242.47            H
ATOM    369  N   CYS    29       20.385   3.861  23.505  1.00
10.48            N
ATOM    370  CA  CYS    29       19.207   3.005  23.446  1.00
9.97            C
ATOM    371  C   CYS    29       18.617   2.840  24.845  1.00
11.60            C
ATOM    372  O   CYS    29       19.266   2.293  25.738  1.00
15.64            O
ATOM    373  CB  CYS    29       19.567   1.633  22.850  1.00
9.52            C
ATOM    374  SG  CYS    29       18.152   0.968  21.957  1.00
10.40            S
ATOM    375  HN  CYS    29       20.731   4.139  24.378  1.00
11.10            H
ATOM    376  HA  CYS    29       18.471   3.479  22.814  1.00
11.08            H
ATOM    377  HB1 CYS    29       19.836   0.941  23.639  1.00
9.33            H
ATOM    378  HB2 CYS    29       20.397   1.739  22.171  1.00
10.78            H
ATOM    379  N   GLU    30       17.394   3.324  25.037  1.00
13.52            N
ATOM    380  CA  GLU    30       16.742   3.232  26.340  1.00
17.41            C
ATOM    381  C   GLU    30       16.104   1.860  26.533  1.00
10.81            C
ATOM    382  O   GLU    30       15.683   1.220  25.571  1.00
23.22            O
ATOM    383  CB  GLU    30       15.675   4.320  26.464  1.00
37.83            C
ATOM    384  CG  GLU    30       16.334   5.695  26.326
1.00108.13            C
ATOM    385  CD  GLU    30       17.256   5.951  27.513
1.00240.52            C
ATOM    386  OE1 GLU    30       17.084   5.294  28.526
1.00422.56            O
ATOM    387  OE2 GLU    30       18.122   6.803  27.390
1.00410.81            O1-
ATOM    388  HN  GLU    30       16.923   3.758  24.291  1.00
15.61            H
ATOM    389  HA  GLU    30       17.480   3.382  27.114  1.00
24.60            H
ATOM    390  HB1 GLU    30       15.196   4.247  27.428  1.00
72.67            H
ATOM    391  HB2 GLU    30       14.939   4.194  25.683  1.00
```

```
56.47           H
ATOM    392  HG1 GLU    30       15.569   6.453  26.296
1.00196.66           H
ATOM    393  HG2 GLU    30       16.907   5.726  25.411
1.00140.67           H
ATOM    394  N   LYS    31       16.036   1.417  27.787  1.00
11.96           N
ATOM    395  CA  LYS    31       15.447   0.117  28.116  1.00
11.40           C
ATOM    396  C   LYS    31       14.158   0.317  28.908  1.00
10.99           C
ATOM    397  O   LYS    31       14.070   1.210  29.750  1.00
12.86           O
ATOM    398  CB  LYS    31       16.430  -0.707  28.954  1.00
21.40           C
ATOM    399  CG  LYS    31       17.812  -0.737  28.284  1.00
57.81           C
ATOM    400  CD  LYS    31       17.749  -1.518  26.966
1.00115.52           C
ATOM    401  CE  LYS    31       19.171  -1.773  26.463
1.00250.23           C
ATOM    402  NZ  LYS    31       19.115  -2.525  25.177
1.00462.10           N1+
ATOM    403  HN  LYS    31       16.391   1.976  28.510  1.00
26.13           H
ATOM    404  HA  LYS    31       15.213  -0.423  27.209  1.00
13.87           H
ATOM    405  HB1 LYS    31       16.059  -1.717  29.049  1.00
37.63           H
ATOM    406  HB2 LYS    31       16.518  -0.266  29.935  1.00
47.19           H
ATOM    407  HG1 LYS    31       18.519  -1.214  28.946
1.00126.32           H
ATOM    408  HG2 LYS    31       18.136   0.274  28.085
1.00114.19           H
ATOM    409  HD1 LYS    31       17.214  -0.942  26.229
1.00198.45           H
ATOM    410  HD2 LYS    31       17.247  -2.460  27.127
1.00200.14           H
ATOM    411  HE1 LYS    31       19.714  -2.352  27.195
1.00374.64           H
ATOM    412  HE2 LYS    31       19.672  -0.830  26.306
1.00403.27           H
ATOM    413  HZ1 LYS    31       18.128  -2.591  24.857
1.00627.38           H
ATOM    414  HZ2 LYS    31       19.500  -3.480  25.318
1.00622.26           H
ATOM    415  HZ3 LYS    31       19.677  -2.026  24.458
1.00619.20           H
ATOM    416  N   THR    32       13.162  -0.517  28.632  1.00
15.51           N
ATOM    417  CA  THR    32       11.885  -0.417  29.325  1.00
```

```
22.44           C
ATOM    418  C   THR    32       12.035  -0.831  30.785  1.00
26.60           C
ATOM    419  O   THR    32       12.536  -1.915  31.088  1.00
57.43           O
ATOM    420  CB  THR    32       10.847  -1.309  28.640  1.00
56.28           C
ATOM    421  CG2 THR    32        9.506  -1.192  29.367
1.00102.70           C
ATOM    422  OG1 THR    32       10.691  -0.896  27.289
1.00111.17           O
ATOM    423  HN  THR    32       13.287  -1.209  27.951  1.00
18.79           H
ATOM    424  HA  THR    32       11.545   0.607  29.287  1.00
19.67           H
ATOM    425  HB  THR    32       11.178  -2.334  28.667  1.00
84.25           H
ATOM    426  HG1 THR    32       10.149  -1.549  26.841
1.00205.03           H
ATOM    427 HG21 THR    32        8.747  -1.719  28.809
1.00220.71           H
ATOM    428 HG22 THR    32        9.233  -0.151  29.453
1.00174.67           H
ATOM    429 HG23 THR    32        9.592  -1.625  30.353
1.00217.44           H
ATOM    430  N   THR    33       11.596   0.043  31.682  1.00
22.47           N
ATOM    431  CA  THR    33       11.676  -0.224  33.116  1.00
41.60           C
ATOM    432  C   THR    33       10.566   0.483  33.845  1.00
38.03           C
ATOM    433  O   THR    33       10.718   1.616  34.302  1.00
52.73           O
ATOM    434  CB  THR    33       13.030   0.221  33.670  1.00
63.25           C
ATOM    435  CG2 THR    33       14.131  -0.698  33.140
1.00121.65           C
ATOM    436  OG1 THR    33       13.289   1.558  33.264  1.00
96.20           O
ATOM    437  HN  THR    33       11.207   0.888  31.373  1.00
23.82           H
ATOM    438  HA  THR    33       11.548  -1.274  33.297  1.00
67.12           H
ATOM    439  HB  THR    33       13.011   0.169  34.748
1.00107.59           H
ATOM    440  HG1 THR    33       14.162   1.581  32.865
1.00176.27           H
ATOM    441 HG21 THR    33       15.051  -0.499  33.670
1.00255.23           H
ATOM    442 HG22 THR    33       14.277  -0.515  32.086
1.00245.37           H
ATOM    443 HG23 THR    33       13.845  -1.728  33.290
```

```
1.00185.78           H
ATOM    444  N   GLY    34        9.446  -0.211  33.966  1.00
40.93          N
ATOM    445  CA  GLY    34        8.313   0.341  34.661  1.00
45.59          C
ATOM    446  C   GLY    34        7.642   1.430  33.831  1.00
40.97          C
ATOM    447  O   GLY    34        6.423   1.436  33.664  1.00
82.17          O
ATOM    448  HN  GLY    34        9.394  -1.115  33.592  1.00
54.00          H
ATOM    449  HA1 GLY    34        8.666   0.758  35.582  1.00
54.41          H
ATOM    450  HA2 GLY    34        7.601  -0.446  34.870  1.00
62.09          H
ATOM    451  N   ASN    35        8.453   2.348  33.305  1.00
32.51          N
ATOM    452  CA  ASN    35        7.944   3.446  32.479  1.00
33.20          C
ATOM    453  C   ASN    35        8.767   3.565  31.200  1.00
23.17          C
ATOM    454  O   ASN    35        9.990   3.421  31.223  1.00
27.01          O
ATOM    455  CB  ASN    35        8.012   4.761  33.255  1.00
54.51          C
ATOM    456  CG  ASN    35        7.256   4.634  34.565  1.00
80.73          C
ATOM    457  ND2 ASN    35        5.993   4.337  34.547
1.00220.46           N
ATOM    458  OD1 ASN    35        7.835   4.810  35.637
1.00117.80           O
ATOM    459  HN  ASN    35        9.417   2.285  33.470  1.00
51.15          H
ATOM    460  HA  ASN    35        6.914   3.251  32.210  1.00
47.21          H
ATOM    461  HB1 ASN    35        7.574   5.551  32.664  1.00
68.31          H
ATOM    462  HB2 ASN    35        9.037   4.998  33.467  1.00
57.75          H
ATOM    463 HD21 ASN    35        5.533   4.196  33.693
1.00405.39           H
ATOM    464 HD22 ASN    35        5.506   4.254  35.384
1.00245.64           H
ATOM    465  N   PHE    36        8.091   3.824  30.088  1.00
35.72          N
ATOM    466  CA  PHE    36        8.762   3.954  28.803  1.00
33.41          C
ATOM    467  C   PHE    36        9.337   5.357  28.632  1.00
28.51          C
ATOM    468  O   PHE    36        8.754   6.338  29.093  1.00
45.23          O
ATOM    469  CB  PHE    36        7.760   3.670  27.690  1.00
```

67.53 C
ATOM 470 CG PHE 36 7.332 2.224 27.755 1.00
86.71 C
ATOM 471 CD1 PHE 36 6.226 1.860 28.532
1.00106.51 C
ATOM 472 CD2 PHE 36 8.039 1.249 27.041 1.00
99.48 C
ATOM 473 CE1 PHE 36 5.824 0.520 28.593
1.00134.48 C
ATOM 474 CE2 PHE 36 7.637 -0.090 27.101
1.00134.27 C
ATOM 475 CZ PHE 36 6.529 -0.455 27.878
1.00149.48 C
ATOM 476 HN PHE 36 7.119 3.924 30.126 1.00
62.87 H
ATOM 477 HA PHE 36 9.562 3.232 28.745 1.00
28.53 H
ATOM 478 HB1 PHE 36 8.218 3.866 26.739 1.00
73.56 H
ATOM 479 HB2 PHE 36 6.896 4.308 27.810 1.00
88.42 H
ATOM 480 HD1 PHE 36 5.683 2.613 29.085
1.00110.20 H
ATOM 481 HD2 PHE 36 8.892 1.530 26.443 1.00
93.07 H
ATOM 482 HE1 PHE 36 4.971 0.238 29.191
1.00155.06 H
ATOM 483 HE2 PHE 36 8.181 -0.842 26.550
1.00159.46 H
ATOM 484 HZ PHE 36 6.220 -1.488 27.924
1.00183.25 H
ATOM 485 N THR 37 10.486 5.443 27.961 1.00
20.06 N
ATOM 486 CA THR 37 11.147 6.724 27.722 1.00
27.18 C
ATOM 487 C THR 37 11.489 6.863 26.244 1.00
41.87 C
ATOM 488 O THR 37 10.756 6.387 25.377
1.00169.86 O
ATOM 489 CB THR 37 12.428 6.813 28.555 1.00
26.52 C
ATOM 490 CG2 THR 37 12.108 6.511 30.019 1.00
75.69 C
ATOM 491 OG1 THR 37 13.373 5.869 28.071 1.00
81.95 O
ATOM 492 HN THR 37 10.902 4.627 27.617 1.00
18.47 H
ATOM 493 HA THR 37 10.488 7.533 28.005 1.00
38.80 H
ATOM 494 HB THR 37 12.840 7.807 28.479 1.00
81.60 H
ATOM 495 HG1 THR 37 13.814 5.477 28.827

```
1.00188.80            H
ATOM    496 HG21 THR    37       11.717   5.509  30.103
1.00196.98            H
ATOM    497 HG22 THR    37       11.372   7.217  30.380
1.00196.23            H
ATOM    498 HG23 THR    37       13.008   6.597  30.609
1.00168.92            H
ATOM    499  N   GLU    38       12.607   7.518  25.964  1.00
26.53           N
ATOM    500  CA  GLU    38       13.041   7.717  24.585  1.00
30.29           C
ATOM    501  C   GLU    38       14.526   8.064  24.528  1.00
25.39           C
ATOM    502  O   GLU    38       15.085   8.604  25.482  1.00
51.60           O
ATOM    503  CB  GLU    38       12.224   8.838  23.939  1.00
54.82           C
ATOM    504  CG  GLU    38       12.438  10.142  24.711
1.00165.84            C
ATOM    505  CD  GLU    38       11.538  11.236  24.149
1.00292.92            C
ATOM    506  OE1 GLU    38       11.592  12.342  24.660
1.00414.19            O
ATOM    507  OE2 GLU    38       10.805  10.951  23.215
1.00537.65            O1-
ATOM    508  HN  GLU    38       13.149   7.872  26.698  1.00
84.50           H
ATOM    509  HA  GLU    38       12.875   6.805  24.032  1.00
32.15           H
ATOM    510  HB1 GLU    38       11.177   8.579  23.960
1.00144.82            H
ATOM    511  HB2 GLU    38       12.541   8.969  22.915
1.00106.49            H
ATOM    512  HG1 GLU    38       13.471  10.447  24.619
1.00303.31            H
ATOM    513  HG2 GLU    38       12.203   9.984  25.754
1.00323.99            H
ATOM    514  N   CYS    39       15.153   7.758  23.397  1.00
15.84           N
ATOM    515  CA  CYS    39       16.571   8.046  23.216  1.00
14.30           C
ATOM    516  C   CYS    39       16.799   9.561  23.179  1.00
22.31           C
ATOM    517  O   CYS    39       15.920  10.306  22.746  1.00
32.92           O
ATOM    518  CB  CYS    39       17.054   7.416  21.906  1.00
18.08           C
ATOM    519  SG  CYS    39       15.973   7.940  20.552  1.00
17.41           S
ATOM    520  HN  CYS    39       14.653   7.335  22.669  1.00
24.87           H
ATOM    521  HA  CYS    39       17.118   7.611  24.036  1.00
```

```
14.60            H
ATOM    522  HB1 CYS    39      17.028   6.339  21.991  1.00
27.71            H
ATOM    523  HB2 CYS    39      18.064   7.735  21.702  1.00
34.91            H
ATOM    524  N   PRO    40      17.942  10.038  23.618  1.00
27.27            N
ATOM    525  CA  PRO    40      18.244  11.497  23.619  1.00
47.66            C
ATOM    526  C   PRO    40      18.555  12.014  22.216  1.00
71.54            C
ATOM    527  O   PRO    40      19.289  11.383  21.456  1.00
87.70            O
ATOM    528  CB  PRO    40      19.465  11.607  24.538  1.00
53.85            C
ATOM    529  CG  PRO    40      20.170  10.301  24.375  1.00
46.04            C
ATOM    530  CD  PRO    40      19.073   9.255  24.154  1.00
25.70            C
ATOM    531  HA  PRO    40      17.420  12.048  24.043  1.00
58.04            H
ATOM    532  HB1 PRO    40      19.153  11.733  25.565  1.00
67.93            H
ATOM    533  HB2 PRO    40      20.102  12.429  24.234  1.00
70.99            H
ATOM    534  HG1 PRO    40      20.732  10.061  25.266  1.00
65.91            H
ATOM    535  HG2 PRO    40      20.831  10.341  23.516  1.00
54.99            H
ATOM    536  HD1 PRO    40      18.794   8.791  25.088  1.00
24.22            H
ATOM    537  HD2 PRO    40      19.396   8.515  23.439  1.00
26.94            H
ATOM    538  N   GLY    41      17.992  13.168  21.888
1.00105.24            N
ATOM    539  CA  GLY    41      18.210  13.773  20.580
1.00147.55            C
ATOM    540  C   GLY    41      19.679  14.134  20.383
1.00161.74            C
ATOM    541  O   GLY    41      20.234  13.957  19.298
1.00244.37            O
ATOM    542  HN  GLY    41      17.421  13.621  22.539
1.00121.79            H
ATOM    543  HA1 GLY    41      17.615  14.670  20.499
1.00184.40            H
ATOM    544  HA2 GLY    41      17.911  13.078  19.814
1.00157.34            H
ATOM    545  N   LEU    42      20.299  14.643  21.444
1.00162.48            N
ATOM    546  CA  LEU    42      21.710  15.039  21.400
1.00191.48            C
ATOM    547  C   LEU    42      22.556  14.092  22.244
```

1.00166.66 C
ATOM 548 O LEU 42 22.113 13.608 23.286
1.00251.38 O
ATOM 549 CB LEU 42 21.862 16.465 21.936
1.00307.99 C
ATOM 550 CG LEU 42 20.951 17.420 21.156
1.00452.28 C
ATOM 551 CD1 LEU 42 21.081 18.830 21.743
1.00681.24 C
ATOM 552 CD2 LEU 42 21.353 17.440 19.672
1.00527.46 C
ATOM 553 HN LEU 42 19.796 14.759 22.276
1.00195.47 H
ATOM 554 HA LEU 42 22.068 15.010 20.380
1.00229.77 H
ATOM 555 HB1 LEU 42 22.889 16.782 21.825
1.00342.49 H
ATOM 556 HB2 LEU 42 21.593 16.485 22.983
1.00335.33 H
ATOM 557 HG LEU 42 19.926 17.089 21.250
1.00427.79 H
ATOM 558 HD11 LEU 42 20.608 18.860 22.713
1.00909.71 H
ATOM 559 HD12 LEU 42 20.598 19.538 21.086
1.00706.80 H
ATOM 560 HD13 LEU 42 22.125 19.084 21.842
1.00858.82 H
ATOM 561 HD21 LEU 42 21.008 18.357 19.213
1.00631.69 H
ATOM 562 HD22 LEU 42 20.902 16.600 19.165
1.00628.06 H
ATOM 563 HD23 LEU 42 22.429 17.377 19.584
1.00656.75 H
ATOM 564 N THR 43 23.777 13.831 21.787
1.00178.52 N
ATOM 565 CA THR 43 24.679 12.940 22.509
1.00240.84 C
ATOM 566 C THR 43 25.145 13.598 23.814
1.00372.31 C
ATOM 567 O THR 43 25.250 14.822 23.886
1.00500.13 O
ATOM 568 CB THR 43 25.887 12.619 21.623
1.00354.13 C
ATOM 569 CG2 THR 43 25.417 11.899 20.359
1.00463.04 C
ATOM 570 OG1 THR 43 26.543 13.828 21.266
1.00508.50 O
ATOM 571 HN THR 43 24.075 14.246 20.951
1.00229.59 H
ATOM 572 HA THR 43 24.153 12.026 22.727
1.00249.90 H
ATOM 573 HB THR 43 26.572 11.984 22.159

```
1.00481.58           H
ATOM    574  HG1 THR    43       25.914  14.546  21.363
1.00624.31           H
ATOM    575 HG21 THR    43       25.105  10.897  20.610
1.00650.95           H
ATOM    576 HG22 THR    43       26.228  11.856  19.647
1.00580.73           H
ATOM    577 HG23 THR    43       24.586  12.437  19.925
1.00571.99           H
ATOM    578  N   PRO    44       25.423  12.825  24.845
1.00488.39           N
ATOM    579  CA  PRO    44       25.880  13.376  26.158
1.00760.13           C
ATOM    580  C   PRO    44       27.335  13.845  26.119
1.00735.36           C
ATOM    581  O   PRO    44       27.815  14.484  27.054
1.00999.99           O
ATOM    582  CB  PRO    44       25.702  12.193  27.116
1.00999.99           C
ATOM    583  CG  PRO    44       25.908  10.989  26.261
1.00856.94           C
ATOM    584  CD  PRO    44       25.336  11.348  24.886
1.00548.09           C
ATOM    585  HA  PRO    44       25.238  14.187  26.465
1.00939.00           H
ATOM    586  HB1 PRO    44       24.703  12.187  27.527
1.00999.99           H
ATOM    587  HB2 PRO    44       26.438  12.231  27.913
1.00999.99           H
ATOM    588  HG1 PRO    44       25.380  10.140  26.668
1.00999.99           H
ATOM    589  HG2 PRO    44       26.967  10.767  26.181
1.00853.53           H
ATOM    590  HD1 PRO    44       24.306  11.034  24.810
1.00602.45           H
ATOM    591  HD2 PRO    44       25.932  10.905  24.101
1.00463.32           H
ATOM    592  N   ILE    45       28.035  13.512  25.039
1.00518.33           N
ATOM    593  CA  ILE    45       29.436  13.898  24.906
1.00582.79           C
ATOM    594  C   ILE    45       29.603  15.400  25.119
1.00795.93           C
ATOM    595  O   ILE    45       30.638  15.855  25.609
1.00898.75           O
ATOM    596  CB  ILE    45       29.958  13.509  23.518
1.00474.41           C
ATOM    597  CG1 ILE    45       31.482  13.669  23.479
1.00516.90           C
ATOM    598  CG2 ILE    45       29.323  14.412  22.453
1.00760.75           C
ATOM    599  CD1 ILE    45       32.027  13.045  22.193
```

```
1.00566.81           C
ATOM    600  HN  ILE    45       27.607  12.995  24.326
1.00395.72           H
ATOM    601  HA  ILE    45       30.010  13.372  25.653
1.00711.06           H
ATOM    602  HB  ILE    45       29.697  12.481  23.314
1.00477.81           H
ATOM    603 HG11 ILE    45       31.919  13.170  24.331
1.00624.28           H
ATOM    604 HG12 ILE    45       31.734  14.719  23.506
1.00744.84           H
ATOM    605 HG21 ILE    45       29.813  15.375  22.453
1.00920.57           H
ATOM    606 HG22 ILE    45       28.275  14.545  22.671
1.00999.99           H
ATOM    607 HG23 ILE    45       29.432  13.953  21.481
1.00865.46           H
ATOM    608 HD11 ILE    45       31.788  11.991  22.175
1.00658.89           H
ATOM    609 HD12 ILE    45       33.099  13.171  22.159
1.00734.30           H
ATOM    610 HD13 ILE    45       31.579  13.530  21.338
1.00675.84           H
ATOM    611  N   ALA    46       28.582  16.164  24.752
1.00999.99           N
ATOM    612  CA  ALA    46       28.628  17.613  24.908
1.00999.99           C
ATOM    613  C   ALA    46       27.240  18.219  24.716
1.00999.99           C
ATOM    614  CB  ALA    46       29.596  18.219  23.891
1.00999.99           C
ATOM    615  OT1 ALA    46       26.302  17.458  24.545
1.00999.99           O
ATOM    616  OT2 ALA    46       27.137  19.433  24.743
1.00999.99           O
ATOM    617  HN  ALA    46       27.781  15.746  24.368
1.00999.99           H
ATOM    618  HA  ALA    46       28.976  17.847  25.903
1.00999.99           H
ATOM    619  HB1 ALA    46       30.502  17.632  23.864
1.00999.99           H
ATOM    620  HB2 ALA    46       29.832  19.233  24.177
1.00999.99           H
ATOM    621  HB3 ALA    46       29.137  18.217  22.914
1.00999.99           H
ENDMDL
TER
MODEL        3
ATOM      1  N   GLY     1       26.201  -3.594  19.145
1.00999.99           N
ATOM      2  CA  GLY     1       24.825  -3.158  19.516
1.00999.99           C
```

```
ATOM      3  C   GLY     1       24.307  -2.166  18.480
1.00999.99           C
ATOM      4  O   GLY     1       23.805  -1.097  18.828
1.00999.99           O
ATOM      5  HA1 GLY     1       24.845  -2.684  20.485
1.00999.99           H
ATOM      6  HA2 GLY     1       24.172  -4.020  19.549
1.00999.99           H
ATOM      7  HT1 GLY     1       26.784  -2.759  18.935
1.00999.99           H
ATOM      8  HT2 GLY     1       26.155  -4.206  18.305
1.00999.99           H
ATOM      9  HT3 GLY     1       26.623  -4.119  19.936
1.00999.99           H
ATOM     10  N   LEU     2       24.434  -2.527  17.208
1.00999.99           N
ATOM     11  CA  LEU     2       23.976  -1.659  16.128
1.00895.59           C
ATOM     12  C   LEU     2       22.451  -1.629  16.084
1.00532.27           C
ATOM     13  O   LEU     2       21.790  -2.600  16.451
1.00625.21           O
ATOM     14  CB  LEU     2       24.517  -2.161  14.787
1.00999.99           C
ATOM     15  CG  LEU     2       26.050  -2.195  14.820
1.00999.99           C
ATOM     16  CD1 LEU     2       26.567  -2.786  13.504
1.00999.99           C
ATOM     17  CD2 LEU     2       26.611  -0.771  15.003
1.00999.99           C
ATOM     18  HN  LEU     2       24.842  -3.391  16.990
1.00999.99           H
ATOM     19  HA  LEU     2       24.338  -0.660  16.303
1.00890.87           H
ATOM     20  HB1 LEU     2       24.192  -1.499  13.999
1.00924.07           H
ATOM     21  HB2 LEU     2       24.140  -3.156  14.598
1.00999.99           H
ATOM     22  HG  LEU     2       26.375  -2.818  15.642
1.00999.99           H
ATOM     23 HD11 LEU     2       26.220  -3.803  13.405
1.00999.99           H
ATOM     24 HD12 LEU     2       27.647  -2.773  13.504
1.00999.99           H
ATOM     25 HD13 LEU     2       26.200  -2.197  12.677
1.00999.99           H
ATOM     26 HD21 LEU     2       26.632  -0.525  16.055
1.00999.99           H
ATOM     27 HD22 LEU     2       25.985  -0.061  14.481
1.00999.99           H
ATOM     28 HD23 LEU     2       27.616  -0.719  14.606
1.00999.99           H
```

```
ATOM     29  N   CYS     3      21.898  -0.503  15.637
1.00271.28           N
ATOM     30  CA  CYS     3      20.447  -0.355  15.555
1.00104.50           C
ATOM     31  C   CYS     3      19.917  -0.937  14.249  1.00
90.98           C
ATOM     32  O   CYS     3      20.058  -0.335  13.186
1.00207.28           O
ATOM     33  CB  CYS     3      20.070   1.127  15.635  1.00
37.83           C
ATOM     34  SG  CYS     3      20.561   1.791  17.245  1.00
81.56           S
ATOM     35  HN  CYS     3      22.475   0.239  15.361
1.00296.55           H
ATOM     36  HA  CYS     3      19.990  -0.873  16.384
1.00166.45           H
ATOM     37  HB1 CYS     3      19.003   1.233  15.512  1.00
40.38           H
ATOM     38  HB2 CYS     3      20.578   1.670  14.853  1.00
98.45           H
ATOM     39  N   SER     4      19.294  -2.107  14.341  1.00
85.31           N
ATOM     40  CA  SER     4      18.733  -2.757  13.163  1.00
92.94           C
ATOM     41  C   SER     4      17.648  -1.881  12.564  1.00
67.37           C
ATOM     42  O   SER     4      17.543  -1.731  11.347
1.00102.28           O
ATOM     43  CB  SER     4      18.103  -4.092  13.549
1.00123.83           C
ATOM     44  OG  SER     4      17.674  -4.766  12.374
1.00178.89           O
ATOM     45  HN  SER     4      19.204  -2.538  15.217
1.00163.03           H
ATOM     46  HA  SER     4      19.509  -2.924  12.434
1.00129.78           H
ATOM     47  HB1 SER     4      17.251  -3.905  14.195
1.00105.26           H
ATOM     48  HB2 SER     4      18.823  -4.702  14.070
1.00156.87           H
ATOM     49  HG  SER     4      18.434  -5.213  11.992
1.00218.16           H
ATOM     50  N   GLU     5      16.826  -1.325  13.448  1.00
41.67           N
ATOM     51  CA  GLU     5      15.715  -0.476  13.046  1.00
41.21           C
ATOM     52  C   GLU     5      15.537   0.666  14.040  1.00
41.92           C
ATOM     53  O   GLU     5      16.369   0.864  14.924  1.00
74.72           O
ATOM     54  CB  GLU     5      14.442  -1.315  12.987  1.00
49.24           C
```

```
ATOM     55  CG  GLU     5      14.181  -1.911  14.362  1.00
55.48          C
ATOM     56  CD  GLU     5      13.071  -2.955  14.280  1.00
92.06          C
ATOM     57  OE1 GLU     5      12.569  -3.171  13.189
1.00206.49          O
ATOM     58  OE2 GLU     5      12.741  -3.522  15.308
1.00197.24          O1-
ATOM     59  HN  GLU     5      16.963  -1.507  14.402  1.00
45.52          H
ATOM     60  HA  GLU     5      15.907  -0.069  12.073  1.00
60.63          H
ATOM     61  HB1 GLU     5      14.566  -2.110  12.268  1.00
61.59          H
ATOM     62  HB2 GLU     5      13.615  -0.694  12.699  1.00
63.66          H
ATOM     63  HG1 GLU     5      13.884  -1.127  15.043  1.00
58.03          H
ATOM     64  HG2 GLU     5      15.086  -2.371  14.719  1.00
57.77          H
ATOM     65  N   ASN     6      14.449   1.415  13.893  1.00
50.73          N
ATOM     66  CA  ASN     6      14.183   2.533  14.790  1.00
71.04          C
ATOM     67  C   ASN     6      14.064   2.039  16.228  1.00
65.17          C
ATOM     68  O   ASN     6      14.548   2.684  17.160  1.00
94.86          O
ATOM     69  CB  ASN     6      12.889   3.238  14.380  1.00
94.83          C
ATOM     70  CG  ASN     6      13.103   4.003  13.079
1.00160.94          C
ATOM     71  ND2 ASN     6      12.071   4.337  12.353
1.00244.88          N
ATOM     72  OD1 ASN     6      14.238   4.307  12.716
1.00219.73          O
ATOM     73  HN  ASN     6      13.817   1.213  13.171  1.00
72.81          H
ATOM     74  HA  ASN     6      15.000   3.236  14.726  1.00
96.02          H
ATOM     75  HB1 ASN     6      12.596   3.927  15.157
1.00111.09          H
ATOM     76  HB2 ASN     6      12.110   2.502  14.241  1.00
98.13          H
ATOM     77 HD21 ASN     6      11.167   4.096  12.645
1.00272.43          H
ATOM     78 HD22 ASN     6      12.201   4.829  11.516
1.00336.78          H
ATOM     79  N   GLY     7      13.421   0.885  16.402  1.00
51.80          N
ATOM     80  CA  GLY     7      13.240   0.291  17.730  1.00
71.47          C
```

```
ATOM     81  C   GLY     7      13.916  -1.071  17.805  1.00
41.04           C
ATOM     82  O   GLY     7      13.260  -2.094  17.998  1.00
44.54           O
ATOM     83  HN  GLY     7      13.062   0.416  15.619  1.00
45.49           H
ATOM     84  HA1 GLY     7      12.191   0.168  17.920
1.00103.52           H
ATOM     85  HA2 GLY     7      13.665   0.940  18.485
1.00108.67           H
ATOM     86  N   ASP     8      15.232  -1.072  17.644  1.00
27.00           N
ATOM     87  CA  ASP     8      15.997  -2.313  17.687  1.00
14.15           C
ATOM     88  C   ASP     8      15.841  -2.997  19.039  1.00
9.37           C
ATOM     89  O   ASP     8      15.601  -4.203  19.114  1.00
14.61           O
ATOM     90  CB  ASP     8      17.476  -2.025  17.425  1.00
15.06           C
ATOM     91  CG  ASP     8      18.262  -3.331  17.391  1.00
21.22           C
ATOM     92  OD1 ASP     8      17.680  -4.357  17.697
1.00119.56           O
ATOM     93  OD2 ASP     8      19.436  -3.284  17.060
1.00133.27           O1-
ATOM     94  HN  ASP     8      15.693  -0.226  17.488  1.00
35.63           H
ATOM     95  HA  ASP     8      15.631  -2.975  16.921  1.00
20.92           H
ATOM     96  HB1 ASP     8      17.862  -1.396  18.212  1.00
29.39           H
ATOM     97  HB2 ASP     8      17.579  -1.519  16.476  1.00
44.14           H
ATOM     98  N   CYS     9      15.976  -2.220  20.104  1.00
5.93           N
ATOM     99  CA  CYS     9      15.843  -2.762  21.450  1.00
7.54           C
ATOM    100  C   CYS     9      14.431  -3.297  21.668  1.00
14.60           C
ATOM    101  O   CYS     9      14.237  -4.366  22.245  1.00
25.78           O
ATOM    102  CB  CYS     9      16.152  -1.680  22.476  1.00
6.63           C
ATOM    103  SG  CYS     9      17.917  -1.330  22.415  1.00
10.91           S
ATOM    104  HN  CYS     9      16.162  -1.268  19.980  1.00
6.51           H
ATOM    105  HA  CYS     9      16.556  -3.559  21.575  1.00
11.12           H
ATOM    106  HB1 CYS     9      15.891  -2.028  23.463  1.00
11.28           H
```

```
ATOM    107  HB2 CYS     9       15.593  -0.786  22.245  1.00
5.04          H
ATOM    108  N   ALA    10       13.456  -2.539  21.183  1.00
16.26          N
ATOM    109  CA  ALA    10       12.053  -2.919  21.301  1.00
32.04          C
ATOM    110  C   ALA    10       11.193  -1.966  20.473  1.00
45.47          C
ATOM    111  O   ALA    10       11.649  -0.890  20.087
1.00119.04          O
ATOM    112  CB  ALA    10       11.613  -2.889  22.772  1.00
30.10          C
ATOM    113  HN  ALA    10       13.686  -1.704  20.729  1.00
12.81          H
ATOM    114  HA  ALA    10       11.929  -3.922  20.918  1.00
47.14          H
ATOM    115  HB1 ALA    10       10.772  -3.555  22.909  1.00
84.37          H
ATOM    116  HB2 ALA    10       11.323  -1.886  23.048
1.00118.20          H
ATOM    117  HB3 ALA    10       12.430  -3.212  23.398
1.00117.32          H
ATOM    118  N   ALA    11        9.953  -2.360  20.198  1.00
30.62          N
ATOM    119  CA  ALA    11        9.066  -1.514  19.409  1.00
35.24          C
ATOM    120  C   ALA    11        8.855  -0.171  20.103  1.00
22.78          C
ATOM    121  O   ALA    11        8.935   0.884  19.472  1.00
53.33          O
ATOM    122  CB  ALA    11        7.717  -2.209  19.216  1.00
60.49          C
ATOM    123  HN  ALA    11        9.635  -3.227  20.526  1.00
50.37          H
ATOM    124  HA  ALA    11        9.511  -1.343  18.442  1.00
41.70          H
ATOM    125  HB1 ALA    11        7.837  -3.048  18.546
1.00157.06          H
ATOM    126  HB2 ALA    11        7.009  -1.510  18.795
1.00148.83          H
ATOM    127  HB3 ALA    11        7.352  -2.560  20.170
1.00137.06          H
ATOM    128  N   ASP    12        8.596  -0.214  21.406  1.00
18.98          N
ATOM    129  CA  ASP    12        8.389   1.006  22.177  1.00
31.05          C
ATOM    130  C   ASP    12        9.705   1.760  22.347  1.00
22.98          C
ATOM    131  O   ASP    12        9.742   2.991  22.299  1.00
37.14          O
ATOM    132  CB  ASP    12        7.810   0.664  23.552  1.00
48.35          C
```

```
ATOM    133  CG  ASP    12        6.366   0.193  23.406
1.00104.83           C
ATOM    134  OD1 ASP    12        5.844  -0.355  24.362
1.00258.73           O
ATOM    135  OD2 ASP    12        5.805   0.388  22.341
1.00213.46           O1-
ATOM    136  HN  ASP    12        8.551  -1.083  21.858  1.00
36.19          H
ATOM    137  HA  ASP    12        7.688   1.638  21.652  1.00
49.76          H
ATOM    138  HB1 ASP    12        7.837   1.543  24.179  1.00
95.71          H
ATOM    139  HB2 ASP    12        8.400  -0.118  24.004  1.00
54.37          H
ATOM    140  N   GLU    13       10.780   1.008  22.555  1.00
17.60          N
ATOM    141  CA  GLU    13       12.103   1.597  22.742  1.00
12.01          C
ATOM    142  C   GLU    13       12.686   2.038  21.403  1.00
10.07          C
ATOM    143  O   GLU    13       12.300   1.521  20.354  1.00
12.81          O
ATOM    144  CB  GLU    13       13.025   0.565  23.395  1.00
11.74          C
ATOM    145  CG  GLU    13       12.510   0.242  24.800  1.00
13.49          C
ATOM    146  CD  GLU    13       13.174  -1.026  25.327
1.00142.36           C
ATOM    147  OE1 GLU    13       12.985  -1.329  26.492
1.00339.06           O
ATOM    148  OE2 GLU    13       13.853  -1.678  24.553
1.00335.28           O1-
ATOM    149  HN  GLU    13       10.682   0.033  22.588  1.00
28.36          H
ATOM    150  HA  GLU    13       12.020   2.452  23.394  1.00
13.06          H
ATOM    151  HB1 GLU    13       14.024   0.968  23.463  1.00
13.76          H
ATOM    152  HB2 GLU    13       13.040  -0.334  22.797  1.00
9.94          H
ATOM    153  HG1 GLU    13       11.440   0.098  24.766  1.00
66.02          H
ATOM    154  HG2 GLU    13       12.738   1.065  25.462  1.00
56.23          H
ATOM    155  N   CYS    14       13.619   2.993  21.441  1.00
9.12          N
ATOM    156  CA  CYS    14       14.253   3.493  20.214  1.00
10.69          C
ATOM    157  C   CYS    14       15.695   3.003  20.129  1.00
9.56          C
ATOM    158  O   CYS    14       16.223   2.441  21.087  1.00
13.62          O
```

```
ATOM    159  CB  CYS    14      14.216   5.036  20.181  1.00
13.96          C
ATOM    160  SG  CYS    14      15.628   5.728  21.087  1.00
42.96          S
ATOM    161  HN  CYS    14      13.892   3.365  22.308  1.00
9.76           H
ATOM    162  HA  CYS    14      13.713   3.119  19.353  1.00
15.54          H
ATOM    163  HB1 CYS    14      13.297   5.378  20.637  1.00
50.72          H
ATOM    164  HB2 CYS    14      14.252   5.373  19.156  1.00
46.13          H
ATOM    165  N   CYS    15      16.329   3.231  18.984  1.00
9.60           N
ATOM    166  CA  CYS    15      17.714   2.820  18.789  1.00
9.28           C
ATOM    167  C   CYS    15      18.346   3.680  17.706  1.00
9.92           C
ATOM    168  O   CYS    15      17.800   3.812  16.611  1.00
13.31          O
ATOM    169  CB  CYS    15      17.777   1.346  18.382  1.00
12.90          C
ATOM    170  SG  CYS    15      19.471   0.731  18.576  1.00
39.11          S
ATOM    171  HN  CYS    15      15.859   3.693  18.258  1.00
13.12          H
ATOM    172  HA  CYS    15      18.260   2.956  19.712  1.00
8.26           H
ATOM    173  HB1 CYS    15      17.473   1.243  17.351  1.00
24.82          H
ATOM    174  HB2 CYS    15      17.113   0.771  19.011  1.00
27.31          H
ATOM    175  N   VAL    16      19.494   4.273  18.014  1.00
9.56           N
ATOM    176  CA  VAL    16      20.179   5.127  17.049  1.00
11.99          C
ATOM    177  C   VAL    16      21.681   5.100  17.290  1.00
7.73           C
ATOM    178  O   VAL    16      22.147   5.296  18.413  1.00
8.83           O
ATOM    179  CB  VAL    16      19.653   6.559  17.167  1.00
18.37          C
ATOM    180  CG1 VAL    16      19.852   7.065  18.597  1.00
39.61          C
ATOM    181  CG2 VAL    16      20.413   7.460  16.195
1.00115.21          C
ATOM    182  HN  VAL    16      19.886   4.141  18.904  1.00
9.76           H
ATOM    183  HA  VAL    16      19.982   4.764  16.050  1.00
17.27          H
ATOM    184  HB  VAL    16      18.600   6.573  16.925  1.00
52.63          H
```

```
ATOM    185 HG11 VAL    16       19.279   7.970  18.740
1.00128.07             H
ATOM    186 HG12 VAL    16       20.897   7.271  18.762
1.00154.13             H
ATOM    187 HG13 VAL    16       19.514   6.314  19.296
1.00135.74             H
ATOM    188 HG21 VAL    16       20.456   6.988  15.225
1.00229.05             H
ATOM    189 HG22 VAL    16       21.415   7.620  16.561
1.00261.82             H
ATOM    190 HG23 VAL    16       19.904   8.408  16.112
1.00210.07             H
ATOM    191  N   ASP    17       22.439   4.846  16.225  1.00
14.34           N
ATOM    192  CA  ASP    17       23.896   4.782  16.321  1.00
11.90           C
ATOM    193  C   ASP    17       24.525   6.123  15.963  1.00
13.15           C
ATOM    194  O   ASP    17       24.364   6.620  14.848  1.00
25.96           O
ATOM    195  CB  ASP    17       24.428   3.707  15.373  1.00
20.80           C
ATOM    196  CG  ASP    17       24.057   2.322  15.893  1.00
28.72           C
ATOM    197  OD1 ASP    17       24.166   1.376  15.130
1.00135.42             O
ATOM    198  OD2 ASP    17       23.668   2.228  17.046
1.00114.17             O1-
ATOM    199  HN  ASP    17       22.009   4.691  15.358  1.00
28.29           H
ATOM    200  HA  ASP    17       24.178   4.522  17.332  1.00
10.49           H
ATOM    201  HB1 ASP    17       25.505   3.787  15.310  1.00
22.42           H
ATOM    202  HB2 ASP    17       24.000   3.849  14.393  1.00
29.52           H
ATOM    203  N   THR    18       25.262   6.695  16.913  1.00
11.55           N
ATOM    204  CA  THR    18       25.941   7.972  16.701  1.00
17.10           C
ATOM    205  C   THR    18       27.430   7.732  16.494  1.00
10.63           C
ATOM    206  O   THR    18       27.937   6.654  16.799  1.00
6.49           O
ATOM    207  CB  THR    18       25.732   8.889  17.909  1.00
28.52           C
ATOM    208  CG2 THR    18       24.305   9.434  17.906  1.00
45.38           C
ATOM    209  OG1 THR    18       25.962   8.157  19.101  1.00
26.49           O
ATOM    210  HN  THR    18       25.361   6.240  17.776  1.00
13.96           H
```

```
ATOM    211  HA  THR    18       25.539   8.455  15.821  1.00
26.43           H
ATOM    212  HB  THR    18       26.425   9.715  17.856  1.00
37.43           H
ATOM    213  HG1 THR    18       25.276   7.491  19.178  1.00
71.62           H
ATOM    214 HG21 THR    18       24.193  10.149  18.707
1.00100.44           H
ATOM    215 HG22 THR    18       23.608   8.621  18.049
1.00129.42           H
ATOM    216 HG23 THR    18       24.106   9.917  16.962
1.00156.21           H
ATOM    217  N   VAL    19       28.124   8.748  15.985  1.00
16.32           N
ATOM    218  CA  VAL    19       29.543   8.657  15.745  1.00
13.34           C
ATOM    219  C   VAL    19       30.020   9.943  15.098  1.00
25.88           C
ATOM    220  O   VAL    19       29.578  10.328  14.016  1.00
42.11           O
ATOM    221  CB  VAL    19       29.894   7.461  14.853  1.00
15.48           C
ATOM    222  CG1 VAL    19       28.976   7.428  13.628  1.00
29.06           C
ATOM    223  CG2 VAL    19       31.354   7.588  14.393  1.00
20.67           C
ATOM    224  HN  VAL    19       27.675   9.585  15.780  1.00
26.89           H
ATOM    225  HA  VAL    19       30.047   8.540  16.694  1.00
8.08           H
ATOM    226  HB  VAL    19       29.777   6.549  15.420  1.00
11.49           H
ATOM    227 HG11 VAL    19       29.123   6.501  13.094
1.00137.43           H
ATOM    228 HG12 VAL    19       29.210   8.257  12.979  1.00
93.29           H
ATOM    229 HG13 VAL    19       27.946   7.499  13.946
1.00108.69           H
ATOM    230 HG21 VAL    19       31.428   8.359  13.639  1.00
99.42           H
ATOM    231 HG22 VAL    19       31.688   6.650  13.981
1.00111.36           H
ATOM    232 HG23 VAL    19       31.972   7.859  15.240  1.00
77.11           H
ATOM    233  N   PHE    20       30.922  10.591  15.788  1.00
24.58           N
ATOM    234  CA  PHE    20       31.500  11.848  15.338  1.00
40.34           C
ATOM    235  C   PHE    20       32.850  11.620  14.668  1.00
40.37           C
ATOM    236  O   PHE    20       33.009  11.878  13.474  1.00
66.02           O
```

```
ATOM    237  CB  PHE    20       31.662  12.803  16.539  1.00
48.79           C
ATOM    238  CG  PHE    20       31.848  12.018  17.826  1.00
35.75           C
ATOM    239  CD1 PHE    20       30.799  11.235  18.340  1.00
30.20           C
ATOM    240  CD2 PHE    20       33.069  12.076  18.511  1.00
40.95           C
ATOM    241  CE1 PHE    20       30.977  10.517  19.526  1.00
30.41           C
ATOM    242  CE2 PHE    20       33.244  11.356  19.698  1.00
45.84           C
ATOM    243  CZ  PHE    20       32.200  10.576  20.205  1.00
40.81           C
ATOM    244  HN  PHE    20       31.200  10.213  16.638  1.00
16.73           H
ATOM    245  HA  PHE    20       30.835  12.310  14.617  1.00
59.91           H
ATOM    246  HB1 PHE    20       30.784  13.403  16.623  1.00
67.15           H
ATOM    247  HB2 PHE    20       32.514  13.451  16.386  1.00
58.17           H
ATOM    248  HD1 PHE    20       29.851  11.183  17.821  1.00
33.34           H
ATOM    249  HD2 PHE    20       33.876  12.677  18.122  1.00
49.35           H
ATOM    250  HE1 PHE    20       30.173   9.915  19.914  1.00
30.61           H
ATOM    251  HE2 PHE    20       34.188  11.401  20.222  1.00
61.27           H
ATOM    252  HZ  PHE    20       32.336  10.020  21.120  1.00
52.66           H
ATOM    253  N   GLU    21       33.830  11.170  15.446  1.00
41.31           N
ATOM    254  CA  GLU    21       35.166  10.952  14.936  1.00
56.16           C
ATOM    255  C   GLU    21       35.490   9.467  14.831  1.00
58.61           C
ATOM    256  O   GLU    21       34.620   8.612  15.003
1.00201.19          O
ATOM    257  CB  GLU    21       36.119  11.631  15.895  1.00
57.37           C
ATOM    258  CG  GLU    21       36.082  10.913  17.241
1.00193.56          C
ATOM    259  CD  GLU    21       36.795  11.749  18.298
1.00304.62          C
ATOM    260  OE1 GLU    21       36.740  11.375  19.458
1.00451.81          O
ATOM    261  OE2 GLU    21       37.386  12.751  17.932
1.00442.88          O1-
ATOM    262  HN  GLU    21       33.665  11.009  16.396  1.00
50.82           H
```

```
ATOM    263  HA  GLU    21       35.272  11.408  13.962  1.00
85.72          H
ATOM    264  HB1 GLU    21       35.804  12.653  16.031  1.00
46.37          H
ATOM    265  HB2 GLU    21       37.111  11.603  15.495
1.00131.81          H
ATOM    266  HG1 GLU    21       36.570   9.960  17.148
1.00314.91          H
ATOM    267  HG2 GLU    21       35.058  10.755  17.536
1.00266.92          H
ATOM    268  N   GLY    22       36.751   9.172  14.530  1.00
86.70          N
ATOM    269  CA  GLY    22       37.205   7.786  14.383
1.00104.66          C
ATOM    270  C   GLY    22       37.822   7.247  15.672  1.00
71.50          C
ATOM    271  O   GLY    22       38.037   6.043  15.807  1.00
89.87          O
ATOM    272  HN  GLY    22       37.391   9.907  14.402
1.00214.64          H
ATOM    273  HA1 GLY    22       37.945   7.742  13.598
1.00152.47          H
ATOM    274  HA2 GLY    22       36.365   7.162  14.107
1.00118.15          H
ATOM    275  N   ASP    23       38.112   8.139  16.614  1.00
44.68          N
ATOM    276  CA  ASP    23       38.713   7.726  17.880  1.00
43.33          C
ATOM    277  C   ASP    23       37.798   6.758  18.621  1.00
37.55          C
ATOM    278  O   ASP    23       38.256   5.768  19.192  1.00
61.37          O
ATOM    279  CB  ASP    23       38.967   8.950  18.762  1.00
40.74          C
ATOM    280  CG  ASP    23       40.105   9.784  18.182
1.00141.86          C
ATOM    281  OD1 ASP    23       40.804   9.279  17.319
1.00328.18          O
ATOM    282  OD2 ASP    23       40.261  10.916  18.610
1.00304.58          O1-
ATOM    283  HN  ASP    23       37.928   9.086  16.453  1.00
40.86          H
ATOM    284  HA  ASP    23       39.652   7.238  17.680  1.00
72.12          H
ATOM    285  HB1 ASP    23       39.234   8.625  19.755  1.00
94.15          H
ATOM    286  HB2 ASP    23       38.071   9.548  18.811  1.00
93.26          H
ATOM    287  N   MET    24       36.508   7.054  18.605  1.00
23.24          N
ATOM    288  CA  MET    24       35.528   6.208  19.277  1.00
32.50          C
```

ATOM 289 C MET 24 34.134 6.476 18.736 1.00
25.13 C
ATOM 290 O MET 24 33.890 7.501 18.100 1.00
54.40 O
ATOM 291 CB MET 24 35.558 6.464 20.787 1.00
47.36 C
ATOM 292 CG MET 24 35.186 7.921 21.071
1.00151.73 C
ATOM 293 SD MET 24 35.351 8.253 22.845
1.00209.23 S
ATOM 294 CE MET 24 37.156 8.143 22.954
1.00243.29 C
ATOM 295 HN MET 24 36.206 7.856 18.131 1.00
17.32 H
ATOM 296 HA MET 24 35.771 5.172 19.094 1.00
48.94 H
ATOM 297 HB1 MET 24 36.551 6.268 21.163
1.00124.40 H
ATOM 298 HB2 MET 24 34.854 5.809 21.276
1.00166.96 H
ATOM 299 HG1 MET 24 34.164 8.095 20.769
1.00331.71 H
ATOM 300 HG2 MET 24 35.840 8.576 20.515
1.00302.30 H
ATOM 301 HE1 MET 24 37.597 8.464 22.021
1.00340.44 H
ATOM 302 HE2 MET 24 37.444 7.123 23.152
1.00373.88 H
ATOM 303 HE3 MET 24 37.505 8.775 23.759
1.00386.81 H
ATOM 304 N VAL 25 33.226 5.535 18.978 1.00
22.44 N
ATOM 305 CA VAL 25 31.861 5.645 18.506 1.00
15.37 C
ATOM 306 C VAL 25 30.880 5.628 19.669 1.00
17.47 C
ATOM 307 O VAL 25 31.214 5.201 20.776 1.00
29.52 O
ATOM 308 CB VAL 25 31.587 4.476 17.575 1.00
23.36 C
ATOM 309 CG1 VAL 25 32.415 4.631 16.297 1.00
58.39 C
ATOM 310 CG2 VAL 25 31.970 3.168 18.273 1.00
55.25 C
ATOM 311 HN VAL 25 33.482 4.734 19.470 1.00
48.11 H
ATOM 312 HA VAL 25 31.731 6.568 17.958 1.00
9.25 H
ATOM 313 HB VAL 25 30.551 4.460 17.335 1.00
52.45 H
ATOM 314 HG11 VAL 25 32.356 5.651 15.947
1.00171.07 H

ATOM   315 HG12 VAL   25   32.030   3.969 15.536
1.00166.22   H
ATOM   316 HG13 VAL   25   33.445   4.383 16.502
1.00134.76   H
ATOM   317 HG21 VAL   25   31.622   2.332 17.685
1.00141.88   H
ATOM   318 HG22 VAL   25   31.515   3.133 19.251
1.00184.50   H
ATOM   319 HG23 VAL   25   33.044   3.115 18.372
1.00133.90   H
ATOM   320 N   THR   26   29.670   6.116 19.411 1.00
13.60   N
ATOM   321 CA THR   26   28.628   6.187 20.433 1.00
21.19   C
ATOM   322 C   THR   26   27.320   5.580 19.932 1.00
16.55   C
ATOM   323 O   THR   26   26.921   5.793 18.786 1.00
11.43   O
ATOM   324 CB THR   26   28.403   7.651 20.808 1.00
27.07   C
ATOM   325 CG2 THR   26   27.259   7.761 21.819 1.00
44.39   C
ATOM   326 OG1 THR   26   29.595   8.175 21.375 1.00
34.75   O
ATOM   327 HN THR   26   29.476   6.453 18.510 1.00
10.81   H
ATOM   328 HA THR   26   28.945   5.649 21.315 1.00
33.16   H
ATOM   329 HB THR   26   28.153   8.210 19.917 1.00
20.60   H
ATOM   330 HG1 THR   26   29.562   8.027 22.323 1.00
77.72   H
ATOM   331 HG21 THR   26   27.225   8.766 22.213
1.00126.99   H
ATOM   332 HG22 THR   26   27.424   7.064 22.626
1.00108.32   H
ATOM   333 HG23 THR   26   26.322   7.535 21.334
1.00151.99   H
ATOM   334 N   ARG   27   26.655   4.824 20.807 1.00
22.38   N
ATOM   335 CA ARG   27   25.381   4.181 20.472 1.00
20.21   C
ATOM   336 C   ARG   27   24.338   4.517 21.532 1.00
16.96   C
ATOM   337 O   ARG   27   24.682   4.814 22.676 1.00
20.06   O
ATOM   338 CB ARG   27   25.569   2.663 20.402 1.00
22.14   C
ATOM   339 CG ARG   27   26.549   2.319 19.279
1.00124.30   C
ATOM   340 CD ARG   27   26.553   0.808 19.048
1.00109.38   C

ATOM 341 NE ARG 27 27.004 0.114 20.252
1.00227.73 N
ATOM 342 CZ ARG 27 28.298 -0.091 20.488
1.00426.12 C
ATOM 343 NH1 ARG 27 28.672 -0.715 21.571
1.00767.09 N1+
ATOM 344 NH2 ARG 27 29.191 0.332 19.637
1.00581.78 N
ATOM 345 HN ARG 27 27.029 4.698 21.704 1.00
30.75 H
ATOM 346 HA ARG 27 25.034 4.535 19.511 1.00
23.13 H
ATOM 347 HB1 ARG 27 24.619 2.192 20.205 1.00
87.46 H
ATOM 348 HB2 ARG 27 25.960 2.306 21.343
1.00103.36 H
ATOM 349 HG1 ARG 27 27.542 2.640 19.555
1.00281.97 H
ATOM 350 HG2 ARG 27 26.247 2.822 18.372
1.00276.19 H
ATOM 351 HD1 ARG 27 27.212 0.572 18.225
1.00183.60 H
ATOM 352 HD2 ARG 27 25.552 0.483 18.806
1.00142.93 H
ATOM 353 HE ARG 27 26.342 -0.207 20.898
1.00372.53 H
ATOM 354 HH11 ARG 27 27.986 -1.039 22.225
1.00910.59 H
ATOM 355 HH12 ARG 27 29.644 -0.869 21.749
1.00999.99 H
ATOM 356 HH21 ARG 27 28.905 0.811 18.807
1.00532.54 H
ATOM 357 HH22 ARG 27 30.163 0.177 19.815
1.00948.84 H
ATOM 358 N SER 28 23.058 4.478 21.154 1.00
14.60 N
ATOM 359 CA SER 28 21.980 4.792 22.095 1.00
14.92 C
ATOM 360 C SER 28 20.781 3.878 21.870 1.00
12.12 C
ATOM 361 O SER 28 20.511 3.449 20.747 1.00
13.41 O
ATOM 362 CB SER 28 21.549 6.250 21.925 1.00
23.26 C
ATOM 363 OG SER 28 20.498 6.536 22.838
1.00146.32 O
ATOM 364 HN SER 28 22.834 4.240 20.228 1.00
15.23 H
ATOM 365 HA SER 28 22.334 4.652 23.107 1.00
15.44 H
ATOM 366 HB1 SER 28 21.211 6.407 20.911 1.00
88.73 H

```
ATOM    367  HB2 SER    28       22.384   6.899  22.128
1.00124.68           H
ATOM    368  HG  SER    28       20.854   7.092  23.536
1.00242.47           H
ATOM    369  N   CYS    29       20.067   3.586  22.952  1.00
10.48           N
ATOM    370  CA  CYS    29       18.896   2.723  22.885  1.00
9.97           C
ATOM    371  C   CYS    29       18.178   2.738  24.235  1.00
11.60           C
ATOM    372  O   CYS    29       18.617   2.089  25.184  1.00
15.64           O
ATOM    373  CB  CYS    29       19.319   1.291  22.516  1.00
9.52           C
ATOM    374  SG  CYS    29       18.014   0.491  21.566  1.00
10.40           S
ATOM    375  HN  CYS    29       20.334   3.965  23.816  1.00
11.10           H
ATOM    376  HA  CYS    29       18.226   3.102  22.127  1.00
11.08           H
ATOM    377  HB1 CYS    29       19.500   0.709  23.412  1.00
9.33           H
ATOM    378  HB2 CYS    29       20.217   1.319  21.923  1.00
10.78           H
ATOM    379  N   GLU    30       17.090   3.496  24.322  1.00
13.52           N
ATOM    380  CA  GLU    30       16.349   3.596  25.566  1.00
17.41           C
ATOM    381  C   GLU    30       15.821   2.244  26.005  1.00
10.81           C
ATOM    382  O   GLU    30       15.749   1.299  25.220  1.00
23.22           O
ATOM    383  CB  GLU    30       15.181   4.562  25.403  1.00
37.83           C
ATOM    384  CG  GLU    30       14.417   4.220  24.126
1.00108.13           C
ATOM    385  CD  GLU    30       13.214   5.145  23.971
1.00240.52           C
ATOM    386  OE1 GLU    30       12.308   5.046  24.780
1.00422.56           O
ATOM    387  OE2 GLU    30       13.216   5.937  23.042
1.00410.81           O1-
ATOM    388  HN  GLU    30       16.788   4.003  23.543  1.00
15.61           H
ATOM    389  HA  GLU    30       17.006   3.979  26.333  1.00
24.60           H
ATOM    390  HB1 GLU    30       15.553   5.566  25.347  1.00
72.67           H
ATOM    391  HB2 GLU    30       14.523   4.472  26.250  1.00
56.47           H
ATOM    392  HG1 GLU    30       14.078   3.199  24.177
1.00196.66           H
```

```
ATOM    393  HG2 GLU    30       15.070   4.338  23.278
1.00140.67          H
ATOM    394  N   LYS    31       15.440   2.180  27.274  1.00
11.96          N
ATOM    395  CA  LYS    31       14.897   0.956  27.862  1.00
11.40          C
ATOM    396  C   LYS    31       13.682   1.283  28.721  1.00
10.99          C
ATOM    397  O   LYS    31       13.647   2.316  29.389  1.00
12.86          O
ATOM    398  CB  LYS    31       15.963   0.273  28.719  1.00
21.40          C
ATOM    399  CG  LYS    31       17.145  -0.133  27.831  1.00
57.81          C
ATOM    400  CD  LYS    31       18.115  -1.026  28.615
1.00115.52          C
ATOM    401  CE  LYS    31       18.887  -0.194  29.646
1.00250.23          C
ATOM    402  NZ  LYS    31       19.987  -1.018  30.222
1.00462.10          N1+
ATOM    403  HN  LYS    31       15.517   2.986  27.828  1.00
26.13          H
ATOM    404  HA  LYS    31       14.595   0.279  27.074  1.00
13.87          H
ATOM    405  HB1 LYS    31       15.543  -0.607  29.184  1.00
37.63          H
ATOM    406  HB2 LYS    31       16.298   0.960  29.481  1.00
47.19          H
ATOM    407  HG1 LYS    31       17.663   0.753  27.500
1.00126.32          H
ATOM    408  HG2 LYS    31       16.775  -0.674  26.972
1.00114.19          H
ATOM    409  HD1 LYS    31       18.815  -1.482  27.931
1.00198.45          H
ATOM    410  HD2 LYS    31       17.559  -1.799  29.126
1.00200.14          H
ATOM    411  HE1 LYS    31       18.220   0.114  30.436
1.00374.64          H
ATOM    412  HE2 LYS    31       19.305   0.680  29.166
1.00403.27          H
ATOM    413  HZ1 LYS    31       20.083  -1.899  29.678
1.00627.38          H
ATOM    414  HZ2 LYS    31       19.766  -1.245  31.213
1.00622.26          H
ATOM    415  HZ3 LYS    31       20.879  -0.487  30.177
1.00619.20          H
ATOM    416  N   THR    32       12.686   0.399  28.700  1.00
15.51          N
ATOM    417  CA  THR    32       11.464   0.601  29.482  1.00
22.44          C
ATOM    418  C   THR    32       11.364  -0.428  30.605  1.00
26.60          C
```

```
ATOM    419  O   THR    32       11.373  -1.634  30.360  1.00
57.43           O
ATOM    420  CB  THR    32       10.239   0.480  28.571  1.00
56.28           C
ATOM    421  CG2 THR    32        8.967   0.685  29.393
1.00102.70           C
ATOM    422  OG1 THR    32       10.312   1.468  27.552
1.00111.17           O
ATOM    423  HN  THR    32       12.773  -0.405  28.145  1.00
18.79           H
ATOM    424  HA  THR    32       11.471   1.591  29.918  1.00
19.67           H
ATOM    425  HB  THR    32       10.219  -0.499  28.122  1.00
84.25           H
ATOM    426  HG1 THR    32        9.941   1.095  26.750
1.00205.03           H
ATOM    427 HG21 THR    32        9.071   1.570  30.003
1.00220.71           H
ATOM    428 HG22 THR    32        8.807  -0.173  30.030
1.00174.67           H
ATOM    429 HG23 THR    32        8.123   0.801  28.730
1.00217.44           H
ATOM    430  N   THR    33       11.260   0.064  31.836  1.00
22.47           N
ATOM    431  CA  THR    33       11.147  -0.809  33.001  1.00
41.60           C
ATOM    432  C   THR    33       10.439  -0.098  34.119  1.00
38.03           C
ATOM    433  O   THR    33       11.059   0.466  35.019  1.00
52.73           O
ATOM    434  CB  THR    33       12.524  -1.271  33.475  1.00
63.25           C
ATOM    435  CG2 THR    33       13.128  -2.238  32.456
1.00121.65           C
ATOM    436  OG1 THR    33       13.374  -0.143  33.626  1.00
96.20           O
ATOM    437  HN  THR    33       11.252   1.034  31.964  1.00
23.82           H
ATOM    438  HA  THR    33       10.552  -1.667  32.751  1.00
67.12           H
ATOM    439  HB  THR    33       12.419  -1.774  34.423
1.00107.59           H
ATOM    440  HG1 THR    33       14.268  -0.463  33.768
1.00176.27           H
ATOM    441 HG21 THR    33       12.394  -2.981  32.183
1.00255.23           H
ATOM    442 HG22 THR    33       13.988  -2.726  32.890
1.00245.37           H
ATOM    443 HG23 THR    33       13.432  -1.690  31.575
1.00185.78           H
ATOM    444  N   GLY    34        9.123  -0.147  34.052  1.00
40.93           N
```

```
ATOM    445  CA  GLY    34        8.307   0.477  35.062  1.00
45.59           C
ATOM    446  C   GLY    34        8.226   1.979  34.827  1.00
40.97           C
ATOM    447  O   GLY    34        7.497   2.692  35.515  1.00
82.17           O
ATOM    448  HN  GLY    34        8.699  -0.626  33.313  1.00
54.00           H
ATOM    449  HA1 GLY    34        8.754   0.284  36.017  1.00
54.41           H
ATOM    450  HA2 GLY    34        7.313   0.049  35.035  1.00
62.09           H
ATOM    451  N   ASN    35        8.984   2.446  33.837  1.00
32.51           N
ATOM    452  CA  ASN    35        9.008   3.862  33.488  1.00
33.20           C
ATOM    453  C   ASN    35        9.414   4.031  32.026  1.00
23.17           C
ATOM    454  O   ASN    35       10.167   3.221  31.487  1.00
27.01           O
ATOM    455  CB  ASN    35        9.995   4.613  34.381  1.00
54.51           C
ATOM    456  CG  ASN    35        9.606   4.455  35.846  1.00
80.73           C
ATOM    457  ND2 ASN    35       10.490   4.025  36.704
1.00220.46           N
ATOM    458  OD1 ASN    35        8.465   4.729  36.220
1.00117.80           O
ATOM    459  HN  ASN    35        9.537   1.820  33.325  1.00
51.15           H
ATOM    460  HA  ASN    35        8.020   4.279  33.629  1.00
47.21           H
ATOM    461  HB1 ASN    35        9.985   5.661  34.120  1.00
68.31           H
ATOM    462  HB2 ASN    35       10.989   4.215  34.229  1.00
57.75           H
ATOM    463 HD21 ASN    35       11.398   3.806  36.405
1.00405.39           H
ATOM    464 HD22 ASN    35       10.247   3.920  37.647
1.00245.64           H
ATOM    465  N   PHE    36        8.908   5.083  31.387  1.00
35.72           N
ATOM    466  CA  PHE    36        9.211   5.351  29.989  1.00
33.41           C
ATOM    467  C   PHE    36       10.238   6.471  29.859  1.00
28.51           C
ATOM    468  O   PHE    36       10.222   7.435  30.625  1.00
45.23           O
ATOM    469  CB  PHE    36        7.924   5.752  29.282  1.00
67.53           C
ATOM    470  CG  PHE    36        6.985   4.572  29.252  1.00
86.71           C
```

```
ATOM    471  CD1 PHE    36        6.061   4.388  30.287
1.00106.51           C
ATOM    472  CD2 PHE    36        7.039   3.658  28.192  1.00
99.48           C
ATOM    473  CE1 PHE    36        5.189   3.294  30.262
1.00134.48           C
ATOM    474  CE2 PHE    36        6.168   2.562  28.167
1.00134.27           C
ATOM    475  CZ  PHE    36        5.242   2.381  29.201
1.00149.48           C
ATOM    476  HN  PHE    36        8.307   5.691  31.859  1.00
62.87           H
ATOM    477  HA  PHE    36        9.601   4.457  29.521  1.00
28.53           H
ATOM    478  HB1 PHE    36        8.150   6.059  28.281  1.00
73.56           H
ATOM    479  HB2 PHE    36        7.460   6.569  29.814  1.00
88.42           H
ATOM    480  HD1 PHE    36        6.021   5.094  31.105
1.00110.20           H
ATOM    481  HD2 PHE    36        7.755   3.796  27.394  1.00
93.07           H
ATOM    482  HE1 PHE    36        4.476   3.153  31.060
1.00155.06           H
ATOM    483  HE2 PHE    36        6.210   1.859  27.349
1.00159.46           H
ATOM    484  HZ  PHE    36        4.570   1.536  29.182
1.00183.25           H
ATOM    485  N   THR    37       11.127   6.339  28.879  1.00
20.06           N
ATOM    486  CA  THR    37       12.156   7.342  28.643  1.00
27.18           C
ATOM    487  C   THR    37       12.617   7.288  27.192  1.00
41.87           C
ATOM    488  O   THR    37       13.030   6.241  26.697
1.00169.86           O
ATOM    489  CB  THR    37       13.349   7.102  29.575  1.00
26.52           C
ATOM    490  CG2 THR    37       13.961   5.726  29.293  1.00
75.69           C
ATOM    491  OG1 THR    37       14.327   8.109  29.357  1.00
81.95           O
ATOM    492  HN  THR    37       11.087   5.556  28.296  1.00
18.47           H
ATOM    493  HA  THR    37       11.749   8.322  28.845  1.00
38.80           H
ATOM    494  HB  THR    37       13.017   7.138  30.601  1.00
81.60           H
ATOM    495  HG1 THR    37       14.699   8.350  30.209
1.00188.80           H
ATOM    496 HG21 THR    37       14.566   5.424  30.134
1.00196.98           H
```

```
ATOM    497 HG22 THR    37      14.577   5.781  28.409
1.00196.23          H
ATOM    498 HG23 THR    37      13.173   5.003  29.140
1.00168.92          H
ATOM    499  N   GLU    38      12.533   8.425  26.517  1.00
26.53          N
ATOM    500  CA  GLU    38      12.933   8.513  25.115  1.00
30.29          C
ATOM    501  C   GLU    38      14.445   8.678  24.992  1.00
25.39          C
ATOM    502  O   GLU    38      15.114   9.099  25.937  1.00
51.60          O
ATOM    503  CB  GLU    38      12.237   9.701  24.447  1.00
54.82          C
ATOM    504  CG  GLU    38      10.717   9.483  24.441
1.00165.84          C
ATOM    505  CD  GLU    38      10.124   9.834  25.803
1.00292.92          C
ATOM    506  OE1 GLU    38      10.876  10.253  26.668
1.00414.19          O
ATOM    507  OE2 GLU    38       8.924   9.678  25.962
1.00537.65          O1-
ATOM    508  HN  GLU    38      12.187   9.220  26.968  1.00
84.50          H
ATOM    509  HA  GLU    38      12.639   7.606  24.607  1.00
32.15          H
ATOM    510  HB1 GLU    38      12.586   9.793  23.429
1.00144.82          H
ATOM    511  HB2 GLU    38      12.471  10.607  24.987
1.00106.49          H
ATOM    512  HG1 GLU    38      10.500   8.449  24.213
1.00303.31          H
ATOM    513  HG2 GLU    38      10.271  10.113  23.685
1.00323.99          H
ATOM    514  N   CYS    39      14.977   8.349  23.814  1.00
15.84          N
ATOM    515  CA  CYS    39      16.414   8.470  23.565  1.00
14.30          C
ATOM    516  C   CYS    39      16.922   9.805  24.134  1.00
22.31          C
ATOM    517  O   CYS    39      16.130  10.718  24.366  1.00
32.92          O
ATOM    518  CB  CYS    39      16.693   8.386  22.042  1.00
18.08          C
ATOM    519  SG  CYS    39      15.249   7.709  21.181  1.00
17.41          S
ATOM    520  HN  CYS    39      14.391   8.026  23.102  1.00
24.87          H
ATOM    521  HA  CYS    39      16.924   7.656  24.065  1.00
14.60          H
ATOM    522  HB1 CYS    39      17.541   7.739  21.862  1.00
27.71          H
```

```
ATOM    523  HB2 CYS    39      16.910   9.369  21.648  1.00
34.91          H
ATOM    524  N   PRO    40      18.204   9.940  24.370  1.00
27.27          N
ATOM    525  CA  PRO    40      18.777  11.196  24.929  1.00
47.66          C
ATOM    526  C   PRO    40      18.846  12.307  23.881  1.00
71.54          C
ATOM    527  O   PRO    40      19.294  12.087  22.756  1.00
87.70          O
ATOM    528  CB  PRO    40      20.174  10.772  25.396  1.00
53.85          C
ATOM    529  CG  PRO    40      20.552   9.648  24.486  1.00
46.04          C
ATOM    530  CD  PRO    40      19.246   8.926  24.127  1.00
25.70          C
ATOM    531  HA  PRO    40      18.196  11.523  25.776  1.00
58.04          H
ATOM    532  HB1 PRO    40      20.137  10.422  26.419  1.00
67.93          H
ATOM    533  HB2 PRO    40      20.873  11.594  25.305  1.00
70.99          H
ATOM    534  HG1 PRO    40      21.222   8.964  24.988  1.00
65.91          H
ATOM    535  HG2 PRO    40      21.024  10.040  23.591  1.00
54.99          H
ATOM    536  HD1 PRO    40      19.092   8.072  24.766  1.00
24.22          H
ATOM    537  HD2 PRO    40      19.256   8.631  23.091  1.00
26.94          H
ATOM    538  N   GLY    41      18.396  13.493  24.264
1.00105.24          N
ATOM    539  CA  GLY    41      18.404  14.637  23.359
1.00147.55          C
ATOM    540  C   GLY    41      19.825  15.015  22.961
1.00161.74          C
ATOM    541  O   GLY    41      20.089  15.352  21.808
1.00244.37          O
ATOM    542  HN  GLY    41      18.050  13.603  25.174
1.00121.79          H
ATOM    543  HA1 GLY    41      17.939  15.480  23.847
1.00184.40          H
ATOM    544  HA2 GLY    41      17.844  14.388  22.472
1.00157.34          H
ATOM    545  N   LEU    42      20.735  14.960  23.930
1.00162.48          N
ATOM    546  CA  LEU    42      22.140  15.303  23.691
1.00191.48          C
ATOM    547  C   LEU    42      23.027  14.071  23.851
1.00166.66          C
ATOM    548  O   LEU    42      22.758  13.204  24.681
1.00251.38          O
```

ATOM 549 CB LEU 42 22.588 16.377 24.687
1.00307.99 C
ATOM 550 CG LEU 42 21.665 17.599 24.592
1.00452.28 C
ATOM 551 CD1 LEU 42 22.096 18.635 25.633
1.00681.24 C
ATOM 552 CD2 LEU 42 21.748 18.215 23.184
1.00527.46 C
ATOM 553 HN LEU 42 20.455 14.688 24.829
1.00195.47 H
ATOM 554 HA LEU 42 22.260 15.687 22.688
1.00229.77 H
ATOM 555 HB1 LEU 42 23.599 16.676 24.460
1.00342.49 H
ATOM 556 HB2 LEU 42 22.549 15.974 25.689
1.00335.33 H
ATOM 557 HG LEU 42 20.648 17.294 24.792
1.00427.79 H
ATOM 558 HD11 LEU 42 21.591 19.571 25.440
1.00909.71 H
ATOM 559 HD12 LEU 42 23.164 18.784 25.573
1.00706.80 H
ATOM 560 HD13 LEU 42 21.836 18.283 26.621
1.00858.82 H
ATOM 561 HD21 LEU 42 21.088 17.678 22.517
1.00631.69 H
ATOM 562 HD22 LEU 42 22.761 18.150 22.815
1.00628.06 H
ATOM 563 HD23 LEU 42 21.446 19.252 23.221
1.00656.75 H
ATOM 564 N THR 43 24.083 14.005 23.048
1.00178.52 N
ATOM 565 CA THR 43 25.008 12.878 23.105
1.00240.84 C
ATOM 566 C THR 43 25.923 13.023 24.327
1.00372.31 C
ATOM 567 O THR 43 25.869 14.043 25.014
1.00500.13 O
ATOM 568 CB THR 43 25.841 12.830 21.818
1.00354.13 C
ATOM 569 CG2 THR 43 24.997 13.328 20.641
1.00463.04 C
ATOM 570 OG1 THR 43 26.983 13.663 21.962
1.00508.50 O
ATOM 571 HN THR 43 24.247 14.729 22.407
1.00229.59 H
ATOM 572 HA THR 43 24.433 11.970 23.191
1.00249.90 H
ATOM 573 HB THR 43 26.153 11.816 21.620
1.00481.58 H
ATOM 574 HG1 THR 43 27.748 13.098 22.088
1.00624.31 H

```
ATOM    575 HG21 THR    43       25.488  13.073  19.714
1.00650.95          H
ATOM    576 HG22 THR    43       24.886  14.400  20.707
1.00580.73          H
ATOM    577 HG23 THR    43       24.025  12.861  20.674
1.00571.99          H
ATOM    578  N   PRO    44       26.750  12.043  24.630
1.00488.39          N
ATOM    579  CA  PRO    44       27.661  12.114  25.814
1.00760.13          C
ATOM    580  C   PRO    44       28.653  13.270  25.692
1.00735.36          C
ATOM    581  O   PRO    44       29.205  13.735  26.689
1.00999.99          O
ATOM    582  CB  PRO    44       28.379  10.747  25.814
1.00999.99          C
ATOM    583  CG  PRO    44       27.532   9.863  24.962
1.00856.94          C
ATOM    584  CD  PRO    44       26.937  10.777  23.903
1.00548.09          C
ATOM    585  HA  PRO    44       27.081  12.219  26.716
1.00939.00          H
ATOM    586  HB1 PRO    44       28.431  10.350  26.815
1.00999.99          H
ATOM    587  HB2 PRO    44       29.374  10.836  25.394
1.00999.99          H
ATOM    588  HG1 PRO    44       26.739   9.418  25.546
1.00999.99          H
ATOM    589  HG2 PRO    44       28.137   9.092  24.501
1.00853.53          H
ATOM    590  HD1 PRO    44       26.000  10.390  23.559
1.00602.45          H
ATOM    591  HD2 PRO    44       27.637  10.901  23.086
1.00463.32          H
ATOM    592  N   ILE    45       28.875  13.719  24.462
1.00518.33          N
ATOM    593  CA  ILE    45       29.803  14.813  24.216
1.00582.79          C
ATOM    594  C   ILE    45       29.353  16.069  24.954
1.00795.93          C
ATOM    595  O   ILE    45       30.157  16.750  25.591
1.00898.75          O
ATOM    596  CB  ILE    45       29.890  15.094  22.712
1.00474.41          C
ATOM    597  CG1 ILE    45       30.559  13.910  22.012
1.00516.90          C
ATOM    598  CG2 ILE    45       30.719  16.359  22.467
1.00760.75          C
ATOM    599  CD1 ILE    45       30.400  14.053  20.498
1.00566.81          C
ATOM    600  HN  ILE    45       28.408  13.305  23.707
1.00395.72          H
```

```
ATOM    601  HA  ILE    45       30.777  14.527  24.573
1.00711.06           H
ATOM    602  HB  ILE    45       28.894  15.234  22.315
1.00477.81           H
ATOM    603 HG11 ILE    45       30.091  12.991  22.334
1.00624.28           H
ATOM    604 HG12 ILE    45       31.609  13.886  22.266
1.00744.84           H
ATOM    605 HG21 ILE    45       31.620  16.319  23.060
1.00920.57           H
ATOM    606 HG22 ILE    45       30.141  17.227  22.747
1.00999.99           H
ATOM    607 HG23 ILE    45       30.978  16.424  21.421
1.00865.46           H
ATOM    608 HD11 ILE    45       30.803  15.005  20.182
1.00658.89           H
ATOM    609 HD12 ILE    45       29.353  14.001  20.238
1.00734.30           H
ATOM    610 HD13 ILE    45       30.934  13.256  20.003
1.00675.84           H
ATOM    611  N   ALA    46       28.065  16.367  24.862
1.00999.99           N
ATOM    612  CA  ALA    46       27.511  17.542  25.522
1.00999.99           C
ATOM    613  C   ALA    46       27.752  17.476  27.027
1.00999.99           C
ATOM    614  CB  ALA    46       26.009  17.637  25.249
1.00999.99           C
ATOM    615  OT1 ALA    46       27.025  18.133  27.753
1.00999.99           O
ATOM    616  OT2 ALA    46       28.661  16.769  27.430
1.00999.99           O
ATOM    617  HN  ALA    46       27.476  15.784  24.340
1.00999.99           H
ATOM    618  HA  ALA    46       27.993  18.425  25.129
1.00999.99           H
ATOM    619  HB1 ALA    46       25.659  18.627  25.498
1.00999.99           H
ATOM    620  HB2 ALA    46       25.487  16.908  25.850
1.00999.99           H
ATOM    621  HB3 ALA    46       25.821  17.441  24.204
1.00999.99           H
ENDMDL
TER
MODEL        4
ATOM      1  N   GLY     1       26.757  -0.733  13.713
1.00999.99           N
ATOM      2  CA  GLY     1       26.717   0.553  12.959
1.00999.99           C
ATOM      3  C   GLY     1       25.397   1.265  13.234
1.00999.99           C
ATOM      4  O   GLY     1       25.254   1.958  14.239
```

```
1.00999.99           O
ATOM      5  HA1 GLY     1      26.802   0.354  11.902
1.00999.99           H
ATOM      6  HA2 GLY     1      27.540   1.180  13.275
1.00999.99           H
ATOM      7  HT1 GLY     1      26.871  -1.522  13.047
1.00999.99           H
ATOM      8  HT2 GLY     1      25.869  -0.850  14.244
1.00999.99           H
ATOM      9  HT3 GLY     1      27.559  -0.721  14.376
1.00999.99           H
ATOM     10  N   LEU     2      24.436   1.087  12.330
1.00999.99           N
ATOM     11  CA  LEU     2      23.120   1.715  12.473
1.00895.59           C
ATOM     12  C   LEU     2      22.134   0.741  13.108
1.00532.27           C
ATOM     13  O   LEU     2      22.277  -0.475  12.979
1.00625.21           O
ATOM     14  CB  LEU     2      22.595   2.143  11.101
1.00999.99           C
ATOM     15  CG  LEU     2      23.610   3.066  10.414
1.00999.99           C
ATOM     16  CD1 LEU     2      23.091   3.432   9.021
1.00999.99           C
ATOM     17  CD2 LEU     2      23.807   4.346  11.245
1.00999.99           C
ATOM     18  HN  LEU     2      24.612   0.523  11.548
1.00999.99           H
ATOM     19  HA  LEU     2      23.203   2.588  13.104
1.00890.87           H
ATOM     20  HB1 LEU     2      21.661   2.669  11.223
1.00924.07           H
ATOM     21  HB2 LEU     2      22.437   1.266  10.489
1.00999.99           H
ATOM     22  HG  LEU     2      24.554   2.548  10.318
1.00999.99           H
ATOM     23 HD11 LEU     2      22.758   2.539   8.514
1.00999.99           H
ATOM     24 HD12 LEU     2      23.884   3.895   8.452
1.00999.99           H
ATOM     25 HD13 LEU     2      22.266   4.122   9.114
1.00999.99           H
ATOM     26 HD21 LEU     2      24.523   4.157  12.031
1.00999.99           H
ATOM     27 HD22 LEU     2      22.865   4.647  11.681
1.00999.99           H
ATOM     28 HD23 LEU     2      24.177   5.139  10.611
1.00999.99           H
ATOM     29  N   CYS     3      21.132   1.282  13.795
1.00271.28           N
ATOM     30  CA  CYS     3      20.128   0.446  14.444
```

1.00104.50 C
ATOM 31 C CYS 3 19.344 -0.344 13.401 1.00
90.98 C
ATOM 32 O CYS 3 19.132 0.125 12.283
1.00207.28 O
ATOM 33 CB CYS 3 19.165 1.317 15.257 1.00
37.83 C
ATOM 34 SG CYS 3 20.062 2.108 16.617 1.00
81.56 S
ATOM 35 HN CYS 3 21.066 2.257 13.864
1.00296.55 H
ATOM 36 HA CYS 3 20.623 -0.244 15.110
1.00166.45 H
ATOM 37 HB1 CYS 3 18.375 0.700 15.658 1.00
40.38 H
ATOM 38 HB2 CYS 3 18.740 2.076 14.617 1.00
98.45 H
ATOM 39 N SER 4 18.918 -1.546 13.773 1.00
85.31 N
ATOM 40 CA SER 4 18.160 -2.395 12.860 1.00
92.94 C
ATOM 41 C SER 4 16.876 -1.701 12.443 1.00
67.37 C
ATOM 42 O SER 4 16.482 -1.737 11.276
1.00102.28 O
ATOM 43 CB SER 4 17.786 -3.703 13.549
1.00123.83 C
ATOM 44 OG SER 4 17.181 -4.575 12.603
1.00178.89 O
ATOM 45 HN SER 4 19.118 -1.870 14.676
1.00163.03 H
ATOM 46 HA SER 4 18.756 -2.609 11.987
1.00129.78 H
ATOM 47 HB1 SER 4 17.085 -3.489 14.350
1.00105.26 H
ATOM 48 HB2 SER 4 18.667 -4.170 13.955
1.00156.87 H
ATOM 49 HG SER 4 17.142 -5.452 12.990
1.00218.16 H
ATOM 50 N GLU 5 16.216 -1.091 13.418 1.00
41.67 N
ATOM 51 CA GLU 5 14.954 -0.406 13.179 1.00
41.21 C
ATOM 52 C GLU 5 14.814 0.799 14.103 1.00
41.92 C
ATOM 53 O GLU 5 15.763 1.179 14.789 1.00
74.72 O
ATOM 54 CB GLU 5 13.806 -1.379 13.421 1.00
49.24 C
ATOM 55 CG GLU 5 13.881 -1.887 14.855 1.00
55.48 C
ATOM 56 CD GLU 5 12.907 -3.045 15.054 1.00

```
92.06           C
ATOM     57  OE1 GLU     5      12.851  -3.562  16.157
1.00206.49           O
ATOM     58  OE2 GLU     5      12.235  -3.398  14.099
1.00197.24           O1-
ATOM     59  HN  GLU     5      16.579  -1.119  14.328  1.00
45.52           H
ATOM     60  HA  GLU     5      14.915  -0.073  12.161  1.00
60.63           H
ATOM     61  HB1 GLU     5      13.887  -2.214  12.741  1.00
61.59           H
ATOM     62  HB2 GLU     5      12.869  -0.877  13.266  1.00
63.66           H
ATOM     63  HG1 GLU     5      13.627  -1.085  15.533  1.00
58.03           H
ATOM     64  HG2 GLU     5      14.885  -2.219  15.052  1.00
57.77           H
ATOM     65  N   ASN     6      13.629   1.397  14.117  1.00
50.73           N
ATOM     66  CA  ASN     6      13.384   2.558  14.963  1.00
71.04           C
ATOM     67  C   ASN     6      13.605   2.198  16.427  1.00
65.17           C
ATOM     68  O   ASN     6      14.156   2.986  17.195  1.00
94.86           O
ATOM     69  CB  ASN     6      11.951   3.058  14.767  1.00
94.83           C
ATOM     70  CG  ASN     6      11.808   3.710  13.397
1.00160.94           C
ATOM     71  ND2 ASN     6      10.623   3.843  12.867
1.00244.88           N
ATOM     72  OD1 ASN     6      12.803   4.111  12.793
1.00219.73           O
ATOM     73  HN  ASN     6      12.907   1.053  13.550  1.00
72.81           H
ATOM     74  HA  ASN     6      14.070   3.346  14.687  1.00
96.02           H
ATOM     75  HB1 ASN     6      11.716   3.780  15.534
1.00111.09           H
ATOM     76  HB2 ASN     6      11.268   2.223  14.840  1.00
98.13           H
ATOM     77 HD21 ASN     6       9.832   3.523  13.350
1.00272.43           H
ATOM     78 HD22 ASN     6      10.524   4.261  11.988
1.00336.78           H
ATOM     79  N   GLY     7      13.176   0.995  16.807  1.00
51.80           N
ATOM     80  CA  GLY     7      13.332   0.518  18.183  1.00
71.47           C
ATOM     81  C   GLY     7      14.015  -0.843  18.205  1.00
41.04           C
ATOM     82  O   GLY     7      13.376  -1.869  18.429  1.00
```

```
44.54           O
ATOM     83  HN  GLY     7       12.749   0.411  16.146  1.00
45.49           H
ATOM     84  HA1 GLY     7       12.361   0.429  18.639
1.00103.52           H
ATOM     85  HA2 GLY     7       13.928   1.222  18.751
1.00108.67           H
ATOM     86  N   ASP     8       15.321  -0.838  17.970  1.00
27.00           N
ATOM     87  CA  ASP     8       16.091  -2.077  17.962  1.00
14.15           C
ATOM     88  C   ASP     8       16.024  -2.759  19.322  1.00
9.37           C
ATOM     89  O   ASP     8       15.843  -3.973  19.412  1.00
14.61           O
ATOM     90  CB  ASP     8       17.550  -1.783  17.606  1.00
15.06           C
ATOM     91  CG  ASP     8       18.337  -3.085  17.517  1.00
21.22           C
ATOM     92  OD1 ASP     8       17.780  -4.116  17.858
1.00119.56           O
ATOM     93  OD2 ASP     8       19.486  -3.033  17.108
1.00133.27           O1-
ATOM     94  HN  ASP     8       15.772   0.012  17.799  1.00
35.63           H
ATOM     95  HA  ASP     8       15.679  -2.738  17.220  1.00
20.92           H
ATOM     96  HB1 ASP     8       17.985  -1.155  18.370  1.00
29.39           H
ATOM     97  HB2 ASP     8       17.591  -1.273  16.656  1.00
44.14           H
ATOM     98  N   CYS     9       16.167  -1.967  20.375  1.00
5.93           N
ATOM     99  CA  CYS     9       16.118  -2.500  21.731  1.00
7.54           C
ATOM    100  C   CYS     9       14.750  -3.111  22.007  1.00
14.60           C
ATOM    101  O   CYS     9       14.641  -4.180  22.609  1.00
25.78           O
ATOM    102  CB  CYS     9       16.402  -1.388  22.730  1.00
6.63           C
ATOM    103  SG  CYS     9       18.138  -0.936  22.590  1.00
10.91           S
ATOM    104  HN  CYS     9       16.306  -1.008  20.237  1.00
6.51           H
ATOM    105  HA  CYS     9       16.879  -3.255  21.837  1.00
11.12           H
ATOM    106  HB1 CYS     9       16.204  -1.734  23.732  1.00
11.28           H
ATOM    107  HB2 CYS     9       15.782  -0.531  22.510  1.00
5.04           H
ATOM    108  N   ALA    10       13.714  -2.420  21.553  1.00
```

16.26 N
ATOM 109 CA ALA 10 12.346 -2.884 21.734 1.00
32.04 C
ATOM 110 C ALA 10 11.390 -2.035 20.901 1.00
45.47 C
ATOM 111 O ALA 10 11.746 -0.945 20.454
1.00119.04 O
ATOM 112 CB ALA 10 11.954 -2.818 23.214 1.00
30.10 C
ATOM 113 HN ALA 10 13.875 -1.580 21.076 1.00
12.81 H
ATOM 114 HA ALA 10 12.279 -3.910 21.403 1.00
47.14 H
ATOM 115 HB1 ALA 10 11.128 -3.487 23.400 1.00
84.37 H
ATOM 116 HB2 ALA 10 11.662 -1.809 23.468
1.00118.20 H
ATOM 117 HB3 ALA 10 12.797 -3.111 23.822
1.00117.32 H
ATOM 118 N ALA 11 10.181 -2.540 20.691 1.00
30.62 N
ATOM 119 CA ALA 11 9.189 -1.815 19.904 1.00
35.24 C
ATOM 120 C ALA 11 8.859 -0.470 20.546 1.00
22.78 C
ATOM 121 O ALA 11 8.676 0.531 19.853 1.00
53.33 O
ATOM 122 CB ALA 11 7.911 -2.648 19.784 1.00
60.49 C
ATOM 123 HN ALA 11 9.952 -3.413 21.069 1.00
50.37 H
ATOM 124 HA ALA 11 9.585 -1.643 18.915 1.00
41.70 H
ATOM 125 HB1 ALA 11 8.169 -3.673 19.564
1.00157.06 H
ATOM 126 HB2 ALA 11 7.297 -2.251 18.990
1.00148.83 H
ATOM 127 HB3 ALA 11 7.365 -2.605 20.716
1.00137.06 H
ATOM 128 N ASP 12 8.772 -0.457 21.875 1.00
18.98 N
ATOM 129 CA ASP 12 8.448 0.766 22.610 1.00
31.05 C
ATOM 130 C ASP 12 9.712 1.524 23.019 1.00
22.98 C
ATOM 131 O ASP 12 9.650 2.458 23.819 1.00
37.14 O
ATOM 132 CB ASP 12 7.638 0.416 23.859 1.00
48.35 C
ATOM 133 CG ASP 12 8.451 -0.497 24.771
1.00104.83 C
ATOM 134 OD1 ASP 12 9.510 -0.929 24.350

```
1.00258.73           O
ATOM    135  OD2 ASP    12       8.001  -0.751  25.876
1.00213.46           O1-
ATOM    136  HN  ASP    12       8.920  -1.287  22.372  1.00
36.19          H
ATOM    137  HA  ASP    12       7.848   1.410  21.982  1.00
49.76          H
ATOM    138  HB1 ASP    12       6.730  -0.090  23.565  1.00
95.71          H
ATOM    139  HB2 ASP    12       7.387   1.322  24.390  1.00
54.37          H
ATOM    140  N   GLU    13      10.860   1.120  22.469  1.00
17.60          N
ATOM    141  CA  GLU    13      12.140   1.770  22.784  1.00
12.01          C
ATOM    142  C   GLU    13      12.772   2.370  21.533  1.00
10.07          C
ATOM    143  O   GLU    13      12.446   1.986  20.410  1.00
12.81          O
ATOM    144  CB  GLU    13      13.102   0.758  23.406  1.00
11.74          C
ATOM    145  CG  GLU    13      12.612   0.385  24.808  1.00
13.49          C
ATOM    146  CD  GLU    13      12.798   1.567  25.752
1.00142.36           C
ATOM    147  OE1 GLU    13      13.476   2.503  25.369
1.00339.06           O
ATOM    148  OE2 GLU    13      12.253   1.521  26.842
1.00335.28           O1-
ATOM    149  HN  GLU    13      10.848   0.369  21.839  1.00
28.36          H
ATOM    150  HA  GLU    13      11.973   2.566  23.498  1.00
13.06          H
ATOM    151  HB1 GLU    13      14.086   1.192  23.474  1.00
13.76          H
ATOM    152  HB2 GLU    13      13.141  -0.128  22.788  1.00
9.94          H
ATOM    153  HG1 GLU    13      13.181  -0.457  25.175  1.00
66.02          H
ATOM    154  HG2 GLU    13      11.567   0.124  24.765  1.00
56.23          H
ATOM    155  N   CYS    14      13.681   3.326  21.743  1.00
9.12          N
ATOM    156  CA  CYS    14      14.369   4.001  20.639  1.00
10.69          C
ATOM    157  C   CYS    14      15.799   3.489  20.503  1.00
9.56          C
ATOM    158  O   CYS    14      16.389   3.005  21.467  1.00
13.62          O
ATOM    159  CB  CYS    14      14.397   5.508  20.897  1.00
13.96          C
ATOM    160  SG  CYS    14      15.318   6.332  19.572  1.00
```

42.96 S
ATOM 161 HN CYS 14 13.893 3.588 22.664 1.00
9.76 H
ATOM 162 HA CYS 14 13.839 3.818 19.714 1.00
15.54 H
ATOM 163 HB1 CYS 14 14.881 5.698 21.843 1.00
50.72 H
ATOM 164 HB2 CYS 14 13.387 5.887 20.924 1.00
46.13 H
ATOM 165 N CYS 15 16.352 3.607 19.297 1.00
9.60 N
ATOM 166 CA CYS 15 17.719 3.164 19.031 1.00
9.28 C
ATOM 167 C CYS 15 18.358 4.083 18.001 1.00
9.92 C
ATOM 168 O CYS 15 17.790 4.314 16.933 1.00
13.31 O
ATOM 169 CB CYS 15 17.710 1.728 18.501 1.00
12.90 C
ATOM 170 SG CYS 15 19.414 1.149 18.275 1.00
39.11 S
ATOM 171 HN CYS 15 15.833 4.010 18.569 1.00
13.12 H
ATOM 172 HA CYS 15 18.297 3.201 19.944 1.00
8.26 H
ATOM 173 HB1 CYS 15 17.193 1.698 17.552 1.00
24.82 H
ATOM 174 HB2 CYS 15 17.205 1.086 19.207 1.00
27.31 H
ATOM 175 N VAL 16 19.536 4.612 18.316 1.00
9.56 N
ATOM 176 CA VAL 16 20.220 5.509 17.394 1.00
11.99 C
ATOM 177 C VAL 16 21.726 5.425 17.594 1.00
7.73 C
ATOM 178 O VAL 16 22.219 5.502 18.720 1.00
8.83 O
ATOM 179 CB VAL 16 19.732 6.942 17.614 1.00
18.37 C
ATOM 180 CG1 VAL 16 20.062 7.389 19.039 1.00
39.61 C
ATOM 181 CG2 VAL 16 20.415 7.872 16.613
1.00115.21 C
ATOM 182 HN VAL 16 19.954 4.400 19.184 1.00
9.76 H
ATOM 183 HA VAL 16 19.992 5.217 16.377 1.00
17.27 H
ATOM 184 HB VAL 16 18.663 6.981 17.467 1.00
52.63 H
ATOM 185 HG11 VAL 16 19.548 8.315 19.254
1.00128.07 H
ATOM 186 HG12 VAL 16 21.127 7.539 19.134

```
1.00154.13            H
ATOM    187 HG13 VAL    16      19.740   6.630  19.737
1.00135.74            H
ATOM    188 HG21 VAL    16      20.307   7.467  15.618
1.00229.05            H
ATOM    189 HG22 VAL    16      21.463   7.957  16.857
1.00261.82            H
ATOM    190 HG23 VAL    16      19.954   8.847  16.656
1.00210.07            H
ATOM    191  N   ASP    17      22.456   5.252  16.494  1.00
14.34            N
ATOM    192  CA  ASP    17      23.914   5.141  16.552  1.00
11.90            C
ATOM    193  C   ASP    17      24.581   6.404  16.023  1.00
13.15            C
ATOM    194  O   ASP    17      24.360   6.807  14.880  1.00
25.96            O
ATOM    195  CB  ASP    17      24.373   3.940  15.722  1.00
20.80            C
ATOM    196  CG  ASP    17      23.743   2.662  16.266  1.00
28.72            C
ATOM    197  OD1 ASP    17      23.230   1.894  15.471
1.00135.42            O
ATOM    198  OD2 ASP    17      23.786   2.470  17.470
1.00114.17            O1-
ATOM    199  HN  ASP    17      22.005   5.189  15.626  1.00
28.29            H
ATOM    200  HA  ASP    17      24.224   4.989  17.574  1.00
10.49            H
ATOM    201  HB1 ASP    17      25.449   3.860  15.775  1.00
22.42            H
ATOM    202  HB2 ASP    17      24.073   4.079  14.694  1.00
29.52            H
ATOM    203  N   THR    18      25.419   7.010  16.860  1.00
11.55            N
ATOM    204  CA  THR    18      26.152   8.218  16.486  1.00
17.10            C
ATOM    205  C   THR    18      27.629   7.891  16.337  1.00
10.63            C
ATOM    206  O   THR    18      28.097   6.872  16.845  1.00
6.49            O
ATOM    207  CB  THR    18      25.974   9.301  17.553  1.00
28.52            C
ATOM    208  CG2 THR    18      24.566   9.885  17.469  1.00
45.38            C
ATOM    209  OG1 THR    18      26.184   8.736  18.838  1.00
26.49            O
ATOM    210  HN  THR    18      25.561   6.625  17.750  1.00
13.96            H
ATOM    211  HA  THR    18      25.780   8.593  15.540  1.00
26.43            H
ATOM    212  HB  THR    18      26.692  10.089  17.387  1.00
```

```
37.43           H
ATOM    213  HG1 THR    18       26.187   7.780  18.744  1.00
71.62           H
ATOM    214 HG21 THR    18       24.352  10.438  18.371
1.00100.44           H
ATOM    215 HG22 THR    18       23.849   9.086  17.357
1.00129.42           H
ATOM    216 HG23 THR    18       24.502  10.548  16.617
1.00156.21           H
ATOM    217  N   VAL    19       28.360   8.763  15.657  1.00
16.32           N
ATOM    218  CA  VAL    19       29.773   8.570  15.456  1.00
13.34           C
ATOM    219  C   VAL    19       30.341   9.740  14.685  1.00
25.88           C
ATOM    220  O   VAL    19       29.983  10.000  13.537  1.00
42.11           O
ATOM    221  CB  VAL    19       30.068   7.271  14.708  1.00
15.48           C
ATOM    222  CG1 VAL    19       29.257   7.212  13.407  1.00
29.06           C
ATOM    223  CG2 VAL    19       31.567   7.216  14.385  1.00
20.67           C
ATOM    224  HN  VAL    19       27.945   9.566  15.291  1.00
26.89           H
ATOM    225  HA  VAL    19       30.253   8.526  16.423  1.00
8.08          H
ATOM    226  HB  VAL    19       29.803   6.435  15.334  1.00
11.49           H
ATOM    227 HG11 VAL    19       29.229   6.194  13.048
1.00137.43           H
ATOM    228 HG12 VAL    19       29.722   7.842  12.662  1.00
93.29           H
ATOM    229 HG13 VAL    19       28.251   7.556  13.591
1.00108.69           H
ATOM    230 HG21 VAL    19       31.843   6.210  14.107  1.00
99.42           H
ATOM    231 HG22 VAL    19       32.134   7.521  15.255
1.00111.36           H
ATOM    232 HG23 VAL    19       31.782   7.889  13.568  1.00
77.11           H
ATOM    233  N   PHE    20       31.227  10.437  15.347  1.00
24.58           N
ATOM    234  CA  PHE    20       31.885  11.601  14.782  1.00
40.34           C
ATOM    235  C   PHE    20       33.254  11.232  14.232  1.00
40.37           C
ATOM    236  O   PHE    20       33.545  11.464  13.058  1.00
66.02           O
ATOM    237  CB  PHE    20       32.025  12.682  15.864  1.00
48.79           C
ATOM    238  CG  PHE    20       32.212  12.056  17.237  1.00
```

35.75 C
ATOM 239 CD1 PHE 20 31.162 11.344 17.850 1.00
30.20 C
ATOM 240 CD2 PHE 20 33.431 12.206 17.911 1.00
40.95 C
ATOM 241 CE1 PHE 20 31.343 10.789 19.121 1.00
30.41 C
ATOM 242 CE2 PHE 20 33.607 11.646 19.181 1.00
45.84 C
ATOM 243 CZ PHE 20 32.564 10.939 19.785 1.00
40.81 C
ATOM 244 HN PHE 20 31.437 10.161 16.254 1.00
16.73 H
ATOM 245 HA PHE 20 31.284 11.995 13.971 1.00
59.91 H
ATOM 246 HB1 PHE 20 31.140 13.272 15.873 1.00
67.15 H
ATOM 247 HB2 PHE 20 32.868 13.321 15.640 1.00
58.17 H
ATOM 248 HD1 PHE 20 30.215 11.216 17.342 1.00
33.34 H
ATOM 249 HD2 PHE 20 34.240 12.751 17.446 1.00
49.35 H
ATOM 250 HE1 PHE 20 30.538 10.245 19.588 1.00
30.61 H
ATOM 251 HE2 PHE 20 34.550 11.762 19.695 1.00
61.27 H
ATOM 252 HZ PHE 20 32.700 10.510 20.767 1.00
52.66 H
ATOM 253 N GLU 21 34.105 10.687 15.096 1.00
41.31 N
ATOM 254 CA GLU 21 35.450 10.325 14.715 1.00
56.16 C
ATOM 255 C GLU 21 35.673 8.824 14.822 1.00
58.61 C
ATOM 256 O GLU 21 34.759 8.063 15.136
1.00201.19 O
ATOM 257 CB GLU 21 36.386 11.059 15.646 1.00
57.37 C
ATOM 258 CG GLU 21 36.239 10.513 17.067
1.00193.56 C
ATOM 259 CD GLU 21 36.927 11.449 18.056
1.00304.62 C
ATOM 260 OE1 GLU 21 36.795 11.223 19.247
1.00451.81 O
ATOM 261 OE2 GLU 21 37.576 12.380 17.606
1.00442.88 O1-
ATOM 262 HN GLU 21 33.837 10.554 16.023 1.00
50.82 H
ATOM 263 HA GLU 21 35.651 10.641 13.700 1.00
85.72 H
ATOM 264 HB1 GLU 21 36.128 12.103 15.638 1.00

```
46.37           H
ATOM    265  HB2 GLU    21       37.391  10.929  15.311
1.00131.81           H
ATOM    266  HG1 GLU    21       36.694   9.539  17.122
1.00314.91           H
ATOM    267  HG2 GLU    21       35.194  10.430  17.313
1.00266.92           H
ATOM    268  N   GLY    22       36.905   8.416  14.553  1.00
86.70           N
ATOM    269  CA  GLY    22       37.273   6.999  14.609
1.00104.66           C
ATOM    270  C   GLY    22       37.848   6.636  15.974  1.00
71.50           C
ATOM    271  O   GLY    22       38.014   5.460  16.296  1.00
89.87           O
ATOM    272  HN  GLY    22       37.581   9.086  14.311
1.00214.64           H
ATOM    273  HA1 GLY    22       38.018   6.799  13.852
1.00152.47           H
ATOM    274  HA2 GLY    22       36.400   6.390  14.417
1.00118.15           H
ATOM    275  N   ASP    23       38.156   7.655  16.767  1.00
44.68           N
ATOM    276  CA  ASP    23       38.719   7.439  18.094  1.00
43.33           C
ATOM    277  C   ASP    23       37.755   6.663  18.982  1.00
37.55           C
ATOM    278  O   ASP    23       38.152   5.740  19.693  1.00
61.37           O
ATOM    279  CB  ASP    23       39.034   8.786  18.746  1.00
40.74           C
ATOM    280  CG  ASP    23       39.790   8.571  20.052
1.00141.86           C
ATOM    281  OD1 ASP    23       39.862   7.436  20.491
1.00328.18           O
ATOM    282  OD2 ASP    23       40.285   9.545  20.594
1.00304.58           O1-
ATOM    283  HN  ASP    23       38.008   8.568  16.450  1.00
40.86           H
ATOM    284  HA  ASP    23       39.632   6.879  18.000  1.00
72.12           H
ATOM    285  HB1 ASP    23       38.109   9.305  18.950  1.00
94.15           H
ATOM    286  HB2 ASP    23       39.635   9.379  18.073  1.00
93.26           H
ATOM    287  N   MET    24       36.485   7.051  18.944  1.00
23.24           N
ATOM    288  CA  MET    24       35.464   6.394  19.758  1.00
32.50           C
ATOM    289  C   MET    24       34.085   6.540  19.124  1.00
25.13           C
ATOM    290  O   MET    24       33.772   7.566  18.521  1.00
```

54.40 O
ATOM 291 CB MET 24 35.452 7.006 21.162 1.00
47.36 C
ATOM 292 CG MET 24 34.404 6.297 22.025
1.00151.73 C
ATOM 293 SD MET 24 34.517 6.902 23.730
1.00209.23 S
ATOM 294 CE MET 24 33.888 8.575 23.434
1.00243.29 C
ATOM 295 HN MET 24 36.232 7.799 18.363 1.00
17.32 H
ATOM 296 HA MET 24 35.701 5.342 19.841 1.00
48.94 H
ATOM 297 HB1 MET 24 35.208 8.055 21.093
1.00124.40 H
ATOM 298 HB2 MET 24 36.427 6.892 21.613
1.00166.96 H
ATOM 299 HG1 MET 24 34.586 5.233 22.010
1.00331.71 H
ATOM 300 HG2 MET 24 33.417 6.499 21.637
1.00302.30 H
ATOM 301 HE1 MET 24 33.503 8.982 24.359
1.00340.44 H
ATOM 302 HE2 MET 24 33.099 8.544 22.701
1.00373.88 H
ATOM 303 HE3 MET 24 34.692 9.202 23.069
1.00386.81 H
ATOM 304 N VAL 25 33.259 5.502 19.274 1.00
22.44 N
ATOM 305 CA VAL 25 31.900 5.509 18.722 1.00
15.37 C
ATOM 306 C VAL 25 30.872 5.669 19.837 1.00
17.47 C
ATOM 307 O VAL 25 31.041 5.146 20.939 1.00
29.52 O
ATOM 308 CB VAL 25 31.627 4.207 17.963 1.00
23.36 C
ATOM 309 CG1 VAL 25 30.152 4.158 17.532 1.00
58.39 C
ATOM 310 CG2 VAL 25 32.527 4.148 16.724 1.00
55.25 C
ATOM 311 HN VAL 25 33.568 4.716 19.769 1.00
48.11 H
ATOM 312 HA VAL 25 31.794 6.340 18.034 1.00
9.25 H
ATOM 313 HB VAL 25 31.841 3.365 18.606 1.00
52.45 H
ATOM 314 HG11 VAL 25 29.551 3.807 18.357
1.00171.07 H
ATOM 315 HG12 VAL 25 30.035 3.487 16.693
1.00166.22 H
ATOM 316 HG13 VAL 25 29.823 5.149 17.247

```
1.00134.76            H
ATOM    317 HG21 VAL    25       32.154   4.830  15.975
1.00141.88            H
ATOM    318 HG22 VAL    25       32.528   3.144  16.327
1.00184.50            H
ATOM    319 HG23 VAL    25       33.535   4.428  16.996
1.00133.90            H
ATOM    320  N   THR    26       29.808   6.410  19.536  1.00
13.60           N
ATOM    321  CA  THR    26       28.739   6.667  20.504  1.00
21.19           C
ATOM    322  C   THR    26       27.421   6.041  20.047  1.00
16.55           C
ATOM    323  O   THR    26       27.014   6.195  18.896  1.00
11.43           O
ATOM    324  CB  THR    26       28.561   8.174  20.655  1.00
27.07           C
ATOM    325  CG2 THR    26       27.393   8.470  21.596  1.00
44.39           C
ATOM    326  OG1 THR    26       29.755   8.732  21.185  1.00
34.75           O
ATOM    327  HN  THR    26       29.741   6.803  18.641  1.00
10.81           H
ATOM    328  HA  THR    26       29.008   6.251  21.464  1.00
33.16           H
ATOM    329  HB  THR    26       28.364   8.606  19.683  1.00
20.60           H
ATOM    330  HG1 THR    26       30.483   8.469  20.617  1.00
77.72           H
ATOM    331 HG21 THR    26       27.497   7.882  22.496
1.00126.99            H
ATOM    332 HG22 THR    26       26.462   8.219  21.109
1.00108.32            H
ATOM    333 HG23 THR    26       27.393   9.519  21.850
1.00151.99            H
ATOM    334  N   ARG    27       26.758   5.337  20.967  1.00
22.38           N
ATOM    335  CA  ARG    27       25.476   4.687  20.674  1.00
20.21           C
ATOM    336  C   ARG    27       24.472   4.994  21.781  1.00
16.96           C
ATOM    337  O   ARG    27       24.861   5.284  22.912  1.00
20.06           O
ATOM    338  CB  ARG    27       25.676   3.172  20.574  1.00
22.14           C
ATOM    339  CG  ARG    27       26.646   2.856  19.434
1.00124.30            C
ATOM    340  CD  ARG    27       26.806   1.340  19.306
1.00109.38            C
ATOM    341  NE  ARG    27       27.792   1.021  18.280
1.00227.73            N
ATOM    342  CZ  ARG    27       27.466   1.004  16.992
```

```
1.00426.12           C
ATOM    343  NH1 ARG    27       28.367   0.714  16.094
1.00767.09           N1+
ATOM    344  NH2 ARG    27       26.244   1.279  16.625
1.00581.78           N
ATOM    345  HN  ARG    27       27.137   5.258  21.868  1.00
30.75           H
ATOM    346  HA  ARG    27       25.087   5.052  19.736  1.00
23.13           H
ATOM    347  HB1 ARG    27       24.727   2.696  20.380  1.00
87.46           H
ATOM    348  HB2 ARG    27       26.080   2.800  21.505
1.00103.36           H
ATOM    349  HG1 ARG    27       27.607   3.300  19.644
1.00281.97           H
ATOM    350  HG2 ARG    27       26.258   3.257  18.510
1.00276.19           H
ATOM    351  HD1 ARG    27       25.854   0.901  19.041
1.00183.60           H
ATOM    352  HD2 ARG    27       27.132   0.933  20.251
1.00142.93           H
ATOM    353  HE  ARG    27       28.713   0.814  18.546
1.00372.53           H
ATOM    354 HH11 ARG    27       29.304   0.505  16.375
1.00910.59           H
ATOM    355 HH12 ARG    27       28.121   0.703  15.125
1.00999.99           H
ATOM    356 HH21 ARG    27       25.554   1.501  17.314
1.00532.54           H
ATOM    357 HH22 ARG    27       25.998   1.267  15.656
1.00948.84           H
ATOM    358  N   SER    28       23.176   4.931  21.459  1.00
14.60           N
ATOM    359  CA  SER    28       22.140   5.210  22.455  1.00
14.92           C
ATOM    360  C   SER    28       20.926   4.316  22.235  1.00
12.12           C
ATOM    361  O   SER    28       20.588   3.974  21.100  1.00
13.41           O
ATOM    362  CB  SER    28       21.718   6.677  22.377  1.00
23.26           C
ATOM    363  OG  SER    28       20.679   6.917  23.317
1.00146.32           O
ATOM    364  HN  SER    28       22.910   4.695  20.542  1.00
15.23           H
ATOM    365  HA  SER    28       22.533   5.017  23.443  1.00
15.44           H
ATOM    366  HB1 SER    28       21.372   6.897  21.378  1.00
88.73           H
ATOM    367  HB2 SER    28       22.558   7.306  22.610
1.00124.68           H
ATOM    368  HG  SER    28       20.886   7.726  23.789
```

```
1.00242.47           H
ATOM    369  N   CYS    29       20.279   3.943  23.330  1.00
10.48          N
ATOM    370  CA  CYS    29       19.103   3.086  23.267  1.00
9.97          C
ATOM    371  C   CYS    29       18.483   2.955  24.657  1.00
11.60          C
ATOM    372  O   CYS    29       19.118   2.441  25.580  1.00
15.64          O
ATOM    373  CB  CYS    29       19.493   1.699  22.731  1.00
9.52          C
ATOM    374  SG  CYS    29       18.083   0.953  21.900  1.00
10.40          S
ATOM    375  HN  CYS    29       20.598   4.255  24.203  1.00
11.10          H
ATOM    376  HA  CYS    29       18.380   3.531  22.600  1.00
11.08          H
ATOM    377  HB1 CYS    29       19.794   1.053  23.546  1.00
9.33          H
ATOM    378  HB2 CYS    29       20.306   1.793  22.030  1.00
10.78          H
ATOM    379  N   GLU    30       17.251   3.430  24.806  1.00
13.52          N
ATOM    380  CA  GLU    30       16.570   3.365  26.098  1.00
17.41          C
ATOM    381  C   GLU    30       15.884   2.016  26.284  1.00
10.81          C
ATOM    382  O   GLU    30       15.537   1.343  25.315  1.00
23.22          O
ATOM    383  CB  GLU    30       15.539   4.487  26.197  1.00
37.83          C
ATOM    384  CG  GLU    30       16.254   5.838  26.123
1.00108.13           C
ATOM    385  CD  GLU    30       17.088   6.055  27.382
1.00240.52           C
ATOM    386  OE1 GLU    30       16.846   5.363  28.356
1.00422.56           O
ATOM    387  OE2 GLU    30       17.959   6.910  27.349
1.00410.81           O1-
ATOM    388  HN  GLU    30       16.791   3.834  24.040  1.00
15.61          H
ATOM    389  HA  GLU    30       17.298   3.495  26.885  1.00
24.60          H
ATOM    390  HB1 GLU    30       15.013   4.411  27.134  1.00
72.67          H
ATOM    391  HB2 GLU    30       14.838   4.407  25.379  1.00
56.47          H
ATOM    392  HG1 GLU    30       15.521   6.626  26.040
1.00196.66           H
ATOM    393  HG2 GLU    30       16.899   5.853  25.257
1.00140.67           H
ATOM    394  N   LYS    31       15.691   1.630  27.545  1.00
```

```
11.96           N
ATOM    395  CA  LYS    31       15.039   0.359  27.873  1.00
11.40           C
ATOM    396  C   LYS    31       13.809   0.615  28.737  1.00
10.99           C
ATOM    397  O   LYS    31       13.804   1.520  29.570  1.00
12.86           O
ATOM    398  CB  LYS    31       16.014  -0.544  28.631  1.00
21.40           C
ATOM    399  CG  LYS    31       17.205  -0.879  27.730  1.00
57.81           C
ATOM    400  CD  LYS    31       18.181  -1.782  28.486
1.00115.52           C
ATOM    401  CE  LYS    31       19.373  -2.111  27.586
1.00250.23           C
ATOM    402  NZ  LYS    31       20.328  -2.985  28.325
1.00462.10           N1+
ATOM    403  HN  LYS    31       15.990   2.213  28.272  1.00
26.13           H
ATOM    404  HA  LYS    31       14.732  -0.142  26.966  1.00
13.87           H
ATOM    405  HB1 LYS    31       15.513  -1.457  28.915  1.00
37.63           H
ATOM    406  HB2 LYS    31       16.362  -0.034  29.517  1.00
47.19           H
ATOM    407  HG1 LYS    31       17.708   0.033  27.446
1.00126.32           H
ATOM    408  HG2 LYS    31       16.853  -1.387  26.844
1.00114.19           H
ATOM    409  HD1 LYS    31       17.681  -2.697  28.766
1.00198.45           H
ATOM    410  HD2 LYS    31       18.528  -1.274  29.375
1.00200.14           H
ATOM    411  HE1 LYS    31       19.870  -1.196  27.299
1.00374.64           H
ATOM    412  HE2 LYS    31       19.025  -2.625  26.702
1.00403.27           H
ATOM    413  HZ1 LYS    31       20.029  -3.062  29.318
1.00627.38           H
ATOM    414  HZ2 LYS    31       21.281  -2.572  28.281
1.00622.26           H
ATOM    415  HZ3 LYS    31       20.338  -3.931  27.895
1.00619.20           H
ATOM    416  N   THR    32       12.762  -0.181  28.526  1.00
15.51           N
ATOM    417  CA  THR    32       11.525  -0.020  29.287  1.00
22.44           C
ATOM    418  C   THR    32       11.568  -0.845  30.569  1.00
26.60           C
ATOM    419  O   THR    32       11.685  -2.070  30.530  1.00
57.43           O
ATOM    420  CB  THR    32       10.329  -0.462  28.437  1.00
```

```
56.28           C
ATOM    421  CG2 THR    32        9.040  -0.295  29.243
1.00102.70           C
ATOM    422  OG1 THR    32       10.261   0.336  27.265
1.00111.17           O
ATOM    423  HN  THR    32       12.822  -0.881  27.844  1.00
18.79           H
ATOM    424  HA  THR    32       11.398   1.021  29.547  1.00
19.67           H
ATOM    425  HB  THR    32       10.444  -1.498  28.162  1.00
84.25           H
ATOM    426  HG1 THR    32        9.760   1.128  27.474
1.00205.03           H
ATOM    427 HG21 THR    32        8.190  -0.431  28.590
1.00220.71           H
ATOM    428 HG22 THR    32        9.010   0.695  29.672
1.00174.67           H
ATOM    429 HG23 THR    32        9.011  -1.033  30.031
1.00217.44           H
ATOM    430  N   THR    33       11.464  -0.161  31.704  1.00
22.47           N
ATOM    431  CA  THR    33       11.482  -0.827  33.003  1.00
41.60           C
ATOM    432  C   THR    33       10.729  -0.019  34.021  1.00
38.03           C
ATOM    433  O   THR    33       11.310   0.726  34.810  1.00
52.73           O
ATOM    434  CB  THR    33       12.912  -1.055  33.483  1.00
63.25           C
ATOM    435  CG2 THR    33       13.595  -2.103  32.605
1.00121.65           C
ATOM    436  OG1 THR    33       13.632   0.168  33.418  1.00
96.20           O
ATOM    437  HN  THR    33       11.367   0.815  31.669  1.00
23.82           H
ATOM    438  HA  THR    33       10.982  -1.776  32.926  1.00
67.12           H
ATOM    439  HB  THR    33       12.887  -1.407  34.501
1.00107.59           H
ATOM    440  HG1 THR    33       13.072   0.820  32.993
1.00176.27           H
ATOM    441 HG21 THR    33       14.522  -2.410  33.066
1.00255.23           H
ATOM    442 HG22 THR    33       13.800  -1.680  31.632
1.00245.37           H
ATOM    443 HG23 THR    33       12.947  -2.960  32.495
1.00185.78           H
ATOM    444  N   GLY    34        9.424  -0.197  34.003  1.00
40.93           N
ATOM    445  CA  GLY    34        8.567   0.494  34.935  1.00
45.59           C
ATOM    446  C   GLY    34        8.339   1.935  34.499  1.00
```

```
40.97           C
ATOM    447  O   GLY    34        7.564   2.668  35.115  1.00
82.17           O
ATOM    448  HN  GLY    34        9.037  -0.820  33.358  1.00
54.00           H
ATOM    449  HA1 GLY    34        9.045   0.479  35.895  1.00
54.41           H
ATOM    450  HA2 GLY    34        7.619  -0.021  34.995  1.00
62.09           H
ATOM    451  N   ASN    35        9.017   2.333  33.425  1.00
32.51           N
ATOM    452  CA  ASN    35        8.885   3.686  32.898  1.00
33.20           C
ATOM    453  C   ASN    35        9.324   3.733  31.437  1.00
23.17           C
ATOM    454  O   ASN    35       10.200   2.976  31.020  1.00
27.01           O
ATOM    455  CB  ASN    35        9.736   4.658  33.716  1.00
54.51           C
ATOM    456  CG  ASN    35        9.341   4.598  35.180  1.00
80.73           C
ATOM    457  ND2 ASN    35        8.152   4.973  35.538
1.00220.46           N
ATOM    458  OD1 ASN    35       10.145   4.203  36.025
1.00117.80           O
ATOM    459  HN  ASN    35        9.614   1.699  32.975  1.00
51.15           H
ATOM    460  HA  ASN    35        7.850   3.990  32.962  1.00
47.21           H
ATOM    461  HB1 ASN    35        9.588   5.662  33.347  1.00
68.31           H
ATOM    462  HB2 ASN    35       10.771   4.392  33.624  1.00
57.75           H
ATOM    463 HD21 ASN    35        7.515   5.294  34.865
1.00405.39           H
ATOM    464 HD22 ASN    35        7.897   4.938  36.474
1.00245.64           H
ATOM    465  N   PHE    36        8.706   4.622  30.663  1.00
35.72           N
ATOM    466  CA  PHE    36        9.024   4.766  29.251  1.00
33.41           C
ATOM    467  C   PHE    36        9.884   6.006  29.017  1.00
28.51           C
ATOM    468  O   PHE    36        9.661   7.051  29.628  1.00
45.23           O
ATOM    469  CB  PHE    36        7.722   4.892  28.472  1.00
67.53           C
ATOM    470  CG  PHE    36        6.925   3.619  28.624  1.00
86.71           C
ATOM    471  CD1 PHE    36        5.974   3.510  29.646
1.00106.51           C
ATOM    472  CD2 PHE    36        7.136   2.548  27.746  1.00
```

```
99.48           C
ATOM    473  CE1 PHE    36        5.233   2.331  29.790
1.00134.48           C
ATOM    474  CE2 PHE    36        6.394   1.370  27.890
1.00134.27           C
ATOM    475  CZ  PHE    36        5.444   1.261  28.911
1.00149.48           C
ATOM    476  HN  PHE    36        8.013   5.193  31.044  1.00
62.87           H
ATOM    477  HA  PHE    36        9.558   3.893  28.902  1.00
28.53           H
ATOM    478  HB1 PHE    36        7.942   5.059  27.439  1.00
73.56           H
ATOM    479  HB2 PHE    36        7.151   5.724  28.859  1.00
88.42           H
ATOM    480  HD1 PHE    36        5.814   4.336  30.324
1.00110.20           H
ATOM    481  HD2 PHE    36        7.871   2.630  26.958  1.00
93.07           H
ATOM    482  HE1 PHE    36        4.501   2.249  30.579
1.00155.06           H
ATOM    483  HE2 PHE    36        6.554   0.544  27.213
1.00159.46           H
ATOM    484  HZ  PHE    36        4.871   0.352  29.023
1.00183.25           H
ATOM    485  N   THR    37       10.865   5.883  28.128  1.00
20.06           N
ATOM    486  CA  THR    37       11.751   6.998  27.818  1.00
27.18           C
ATOM    487  C   THR    37       12.427   6.782  26.467  1.00
41.87           C
ATOM    488  O   THR    37       13.073   5.760  26.238
1.00169.86           O
ATOM    489  CB  THR    37       12.814   7.142  28.914  1.00
26.52           C
ATOM    490  CG2 THR    37       13.237   5.758  29.414  1.00
75.69           C
ATOM    491  OG1 THR    37       13.943   7.827  28.389  1.00
81.95           O
ATOM    492  HN  THR    37       10.995   5.029  27.668  1.00
18.47           H
ATOM    493  HA  THR    37       11.168   7.907  27.775  1.00
38.80           H
ATOM    494  HB  THR    37       12.405   7.706  29.740  1.00
81.60           H
ATOM    495  HG1 THR    37       14.342   7.269  27.717
1.00188.80           H
ATOM    496 HG21 THR    37       14.198   5.829  29.901
1.00196.98           H
ATOM    497 HG22 THR    37       13.307   5.076  28.581
1.00196.23           H
ATOM    498 HG23 THR    37       12.505   5.390  30.118
```

```
1.00168.92          H
ATOM    499  N   GLU    38       12.265   7.754  25.578  1.00
26.53           N
ATOM    500  CA  GLU    38       12.852   7.677  24.241  1.00
30.29           C
ATOM    501  C   GLU    38       14.314   8.112  24.259  1.00
25.39           C
ATOM    502  O   GLU    38       14.786   8.709  25.227  1.00
51.60           O
ATOM    503  CB  GLU    38       12.067   8.570  23.277  1.00
54.82           C
ATOM    504  CG  GLU    38       10.624   8.061  23.146
1.00165.84          C
ATOM    505  CD  GLU    38        9.788   8.523  24.337
1.00292.92          C
ATOM    506  OE1 GLU    38       10.324   9.226  25.179
1.00414.19          O
ATOM    507  OE2 GLU    38        8.623   8.166  24.391
1.00537.65          O1-
ATOM    508  HN  GLU    38       11.734   8.539  25.823  1.00
84.50           H
ATOM    509  HA  GLU    38       12.797   6.656  23.893  1.00
32.15           H
ATOM    510  HB1 GLU    38       12.539   8.549  22.307
1.00144.82          H
ATOM    511  HB2 GLU    38       12.062   9.585  23.650
1.00106.49          H
ATOM    512  HG1 GLU    38       10.623   6.980  23.108
1.00303.31          H
ATOM    513  HG2 GLU    38       10.192   8.450  22.237
1.00323.99          H
ATOM    514  N   CYS    39       15.023   7.813  23.174  1.00
15.84           N
ATOM    515  CA  CYS    39       16.433   8.176  23.056  1.00
14.30           C
ATOM    516  C   CYS    39       16.588   9.700  23.093  1.00
22.31           C
ATOM    517  O   CYS    39       15.683  10.422  22.678  1.00
32.92           O
ATOM    518  CB  CYS    39       16.989   7.629  21.738  1.00
18.08           C
ATOM    519  SG  CYS    39       15.889   8.096  20.379  1.00
17.41           S
ATOM    520  HN  CYS    39       14.588   7.343  22.434  1.00
24.87           H
ATOM    521  HA  CYS    39       16.975   7.732  23.876  1.00
14.60           H
ATOM    522  HB1 CYS    39       17.057   6.554  21.794  1.00
27.71           H
ATOM    523  HB2 CYS    39       17.970   8.042  21.561  1.00
34.91           H
ATOM    524  N   PRO    40       17.700  10.211  23.577  1.00
```

```
27.27           N
ATOM    525  CA  PRO    40      17.928  11.682  23.651  1.00
47.66           C
ATOM    526  C   PRO    40      18.265  12.277  22.286  1.00
71.54           C
ATOM    527  O   PRO    40      18.992  11.677  21.495  1.00
87.70           O
ATOM    528  CB  PRO    40      19.108  11.807  24.622  1.00
53.85           C
ATOM    529  CG  PRO    40      19.890  10.551  24.417  1.00
46.04           C
ATOM    530  CD  PRO    40      18.859   9.461  24.098  1.00
25.70           C
ATOM    531  HA  PRO    40      17.065  12.173  24.069  1.00
58.04           H
ATOM    532  HB1 PRO    40      18.755  11.860  25.640  1.00
67.93           H
ATOM    533  HB2 PRO    40      19.709  12.677  24.386  1.00
70.99           H
ATOM    534  HG1 PRO    40      20.433  10.294  25.315  1.00
65.91           H
ATOM    535  HG2 PRO    40      20.578  10.675  23.588  1.00
54.99           H
ATOM    536  HD1 PRO    40      18.586   8.925  24.993  1.00
24.22           H
ATOM    537  HD2 PRO    40      19.244   8.788  23.350  1.00
26.94           H
ATOM    538  N   GLY    41      17.733  13.462  22.024
1.00105.24           N
ATOM    539  CA  GLY    41      17.982  14.141  20.758
1.00147.55           C
ATOM    540  C   GLY    41      19.462  14.462  20.596
1.00161.74           C
ATOM    541  O   GLY    41      20.024  14.326  19.509
1.00244.37           O
ATOM    542  HN  GLY    41      17.168  13.889  22.697
1.00121.79           H
ATOM    543  HA1 GLY    41      17.416  15.060  20.730
1.00184.40           H
ATOM    544  HA2 GLY    41      17.667  13.504  19.947
1.00157.34           H
ATOM    545  N   LEU    42      20.087  14.890  21.690
1.00162.48           N
ATOM    546  CA  LEU    42      21.511  15.237  21.684
1.00191.48           C
ATOM    547  C   LEU    42      22.294  14.266  22.563
1.00166.66           C
ATOM    548  O   LEU    42      21.808  13.828  23.606
1.00251.38           O
ATOM    549  CB  LEU    42      21.702  16.662  22.212
1.00307.99           C
ATOM    550  CG  LEU    42      20.867  17.645  21.382
```

```
1.00452.28              C
ATOM    551  CD1 LEU    42      21.026  19.053  21.964
1.00681.24              C
ATOM    552  CD2 LEU    42      21.341  17.635  19.918
1.00527.46              C
ATOM    553  HN  LEU    42      19.579  14.975  22.522
1.00195.47              H
ATOM    554  HA  LEU    42      21.899  15.182  20.677
1.00229.77              H
ATOM    555  HB1 LEU    42      22.744  16.932  22.143
1.00342.49              H
ATOM    556  HB2 LEU    42      21.387  16.707  23.244
1.00335.33              H
ATOM    557  HG  LEU    42      19.827  17.355  21.426
1.00427.79              H
ATOM    558 HD11 LEU    42      22.073  19.316  21.991
1.00909.71              H
ATOM    559 HD12 LEU    42      20.624  19.076  22.965
1.00706.80              H
ATOM    560 HD13 LEU    42      20.493  19.761  21.346
1.00858.82              H
ATOM    561 HD21 LEU    42      20.873  16.813  19.395
1.00631.69              H
ATOM    562 HD22 LEU    42      22.415  17.521  19.882
1.00628.06              H
ATOM    563 HD23 LEU    42      21.063  18.564  19.439
1.00656.75              H
ATOM    564  N   THR    43      23.507  13.933  22.136
1.00178.52              N
ATOM    565  CA  THR    43      24.346  13.012  22.890
1.00240.84              C
ATOM    566  C   THR    43      24.836  13.674  24.184
1.00372.31              C
ATOM    567  O   THR    43      25.020  14.890  24.223
1.00500.13              O
ATOM    568  CB  THR    43      25.549  12.603  22.032
1.00354.13              C
ATOM    569  CG2 THR    43      25.054  12.017  20.709
1.00463.04              C
ATOM    570  OG1 THR    43      26.352  13.746  21.774
1.00508.50              O
ATOM    571  HN  THR    43      23.841  14.313  21.296
1.00229.59              H
ATOM    572  HA  THR    43      23.769  12.135  23.125
1.00249.90              H
ATOM    573  HB  THR    43      26.134  11.864  22.550
1.00481.58              H
ATOM    574  HG1 THR    43      26.899  13.905  22.547
1.00624.31              H
ATOM    575 HG21 THR    43      24.293  11.277  20.904
1.00650.95              H
ATOM    576 HG22 THR    43      25.881  11.554  20.190
```

1.00580.73 H
ATOM 577 HG23 THR 43 24.642 12.805 20.097
1.00571.99 H
ATOM 578 N PRO 44 25.057 12.914 25.237
1.00488.39 N
ATOM 579 CA PRO 44 25.542 13.473 26.536
1.00760.13 C
ATOM 580 C PRO 44 27.018 13.866 26.473
1.00735.36 C
ATOM 581 O PRO 44 27.578 14.370 27.447
1.00999.99 O
ATOM 582 CB PRO 44 25.311 12.323 27.526
1.00999.99 C
ATOM 583 CG PRO 44 25.430 11.087 26.696
1.00856.94 C
ATOM 584 CD PRO 44 24.869 11.451 25.320
1.00548.09 C
ATOM 585 HA PRO 44 24.944 14.322 26.825
1.00939.00 H
ATOM 586 HB1 PRO 44 24.320 12.387 27.951
1.00999.99 H
ATOM 587 HB2 PRO 44 26.060 12.335 28.308
1.00999.99 H
ATOM 588 HG1 PRO 44 24.851 10.285 27.129
1.00999.99 H
ATOM 589 HG2 PRO 44 26.470 10.796 26.614
1.00853.53 H
ATOM 590 HD1 PRO 44 23.819 11.207 25.261
1.00602.45 H
ATOM 591 HD2 PRO 44 25.422 10.944 24.543
1.00463.32 H
ATOM 592 N ILE 45 27.641 13.625 25.324
1.00518.33 N
ATOM 593 CA ILE 45 29.051 13.951 25.148
1.00582.79 C
ATOM 594 C ILE 45 29.316 15.411 25.502
1.00795.93 C
ATOM 595 O ILE 45 28.612 16.309 25.041
1.00898.75 O
ATOM 596 CB ILE 45 29.468 13.698 23.700
1.00474.41 C
ATOM 597 CG1 ILE 45 29.304 12.212 23.373
1.00516.90 C
ATOM 598 CG2 ILE 45 30.932 14.100 23.511
1.00760.75 C
ATOM 599 CD1 ILE 45 29.457 12.002 21.865
1.00566.81 C
ATOM 600 HN ILE 45 27.145 13.218 24.583
1.00395.72 H
ATOM 601 HA ILE 45 29.642 13.319 25.796
1.00711.06 H
ATOM 602 HB ILE 45 28.847 14.286 23.040

```
1.00477.81           H
ATOM    603 HG11 ILE    45       28.324  11.881  23.680
1.00624.28           H
ATOM    604 HG12 ILE    45       30.058  11.642  23.895
1.00744.84           H
ATOM    605 HG21 ILE    45       31.014  15.176  23.527
1.00920.57           H
ATOM    606 HG22 ILE    45       31.291  13.726  22.564
1.00999.99           H
ATOM    607 HG23 ILE    45       31.525  13.683  24.312
1.00865.46           H
ATOM    608 HD11 ILE    45       30.476  12.211  21.574
1.00658.89           H
ATOM    609 HD12 ILE    45       28.790  12.669  21.339
1.00734.30           H
ATOM    610 HD13 ILE    45       29.214  10.980  21.616
1.00675.84           H
ATOM    611  N   ALA    46       30.338  15.639  26.321
1.00999.99           N
ATOM    612  CA  ALA    46       30.690  16.993  26.732
1.00999.99           C
ATOM    613  C   ALA    46       30.974  17.865  25.512
1.00999.99           C
ATOM    614  CB  ALA    46       31.923  16.960  27.636
1.00999.99           C
ATOM    615  OT1 ALA    46       30.676  19.047  25.571
1.00999.99           O
ATOM    616  OT2 ALA    46       31.484  17.337  24.538
1.00999.99           O
ATOM    617  HN  ALA    46       30.864  14.882  26.656
1.00999.99           H
ATOM    618  HA  ALA    46       29.865  17.418  27.283
1.00999.99           H
ATOM    619  HB1 ALA    46       32.759  16.550  27.090
1.00999.99           H
ATOM    620  HB2 ALA    46       31.720  16.343  28.499
1.00999.99           H
ATOM    621  HB3 ALA    46       32.160  17.963  27.958
1.00999.99           H
ENDMDL
TER
MODEL        5
ATOM      1  N   GLY     1       25.247  -2.355  20.648
1.00999.99           N
ATOM      2  CA  GLY     1       24.976  -2.957  19.311
1.00999.99           C
ATOM      3  C   GLY     1       24.380  -1.901  18.387
1.00999.99           C
ATOM      4  O   GLY     1       23.826  -0.901  18.844
1.00999.99           O
ATOM      5  HA1 GLY     1       24.277  -3.772  19.418
1.00999.99           H
```

```
ATOM      6  HA2 GLY     1       25.900  -3.327  18.890
1.00999.99           H
ATOM      7  HT1 GLY     1       24.579  -2.743  21.343
1.00999.99           H
ATOM      8  HT2 GLY     1       25.130  -1.322  20.593
1.00999.99           H
ATOM      9  HT3 GLY     1       26.218  -2.581  20.940
1.00999.99           H
ATOM     10  N   LEU     2       24.496  -2.131  17.084
1.00999.99           N
ATOM     11  CA  LEU     2       23.964  -1.194  16.100
1.00895.59           C
ATOM     12  C   LEU     2       22.440  -1.230  16.107
1.00532.27           C
ATOM     13  O   LEU     2       21.835  -2.267  16.384
1.00625.21           O
ATOM     14  CB  LEU     2       24.476  -1.557  14.703
1.00999.99           C
ATOM     15  CG  LEU     2       26.010  -1.540  14.681
1.00999.99           C
ATOM     16  CD1 LEU     2       26.496  -2.003  13.304
1.00999.99           C
ATOM     17  CD2 LEU     2       26.529  -0.117  14.959
1.00999.99           C
ATOM     18  HN  LEU     2       24.946  -2.946  16.777
1.00999.99           H
ATOM     19  HA  LEU     2       24.290  -0.197  16.348
1.00890.87           H
ATOM     20  HB1 LEU     2       24.101  -0.840  13.987
1.00924.07           H
ATOM     21  HB2 LEU     2       24.124  -2.544  14.440
1.00999.99           H
ATOM     22  HG  LEU     2       26.385  -2.215  15.438
1.00999.99           H
ATOM     23 HD11 LEU     2       26.324  -1.220  12.580
1.00999.99           H
ATOM     24 HD12 LEU     2       25.954  -2.889  13.011
1.00999.99           H
ATOM     25 HD13 LEU     2       27.553  -2.224  13.351
1.00999.99           H
ATOM     26 HD21 LEU     2       26.564   0.052  16.025
1.00999.99           H
ATOM     27 HD22 LEU     2       25.870   0.609  14.503
1.00999.99           H
ATOM     28 HD23 LEU     2       27.523  -0.004  14.550
1.00999.99           H
ATOM     29  N   CYS     3       21.821  -0.093  15.797
1.00271.28           N
ATOM     30  CA  CYS     3       20.363  -0.013  15.773
1.00104.50           C
ATOM     31  C   CYS     3       19.822  -0.555  14.454  1.00
90.98           C
```

```
ATOM     32  O   CYS     3      19.977   0.070  13.404
1.00207.28           O
ATOM     33  CB  CYS     3      19.912   1.440  15.945  1.00
37.83           C
ATOM     34  SG  CYS     3      20.471   2.068  17.547  1.00
81.56           S
ATOM     35  HN  CYS     3      22.351   0.703  15.584
1.00296.55           H
ATOM     36  HA  CYS     3      19.962  -0.600  16.586
1.00166.45           H
ATOM     37  HB1 CYS     3      18.834   1.489  15.895  1.00
40.38           H
ATOM     38  HB2 CYS     3      20.335   2.043  15.156  1.00
98.45           H
ATOM     39  N   SER     4      19.180  -1.717  14.515  1.00
85.31           N
ATOM     40  CA  SER     4      18.614  -2.330  13.319  1.00
92.94           C
ATOM     41  C   SER     4      17.552  -1.422  12.727  1.00
67.37           C
ATOM     42  O   SER     4      17.462  -1.248  11.512
1.00102.28           O
ATOM     43  CB  SER     4      17.956  -3.661  13.673
1.00123.83           C
ATOM     44  OG  SER     4      17.535  -4.307  12.478
1.00178.89           O
ATOM     45  HN  SER     4      19.083  -2.168  15.381
1.00163.03           H
ATOM     46  HA  SER     4      19.393  -2.499  12.593
1.00129.78           H
ATOM     47  HB1 SER     4      17.096  -3.470  14.309
1.00105.26           H
ATOM     48  HB2 SER     4      18.656  -4.291  14.195
1.00156.87           H
ATOM     49  HG  SER     4      18.022  -5.130  12.398
1.00218.16           H
ATOM     50  N   GLU     5      16.733  -0.866  13.613  1.00
41.67           N
ATOM     51  CA  GLU     5      15.642   0.013  13.211  1.00
41.21           C
ATOM     52  C   GLU     5      15.475   1.144  14.218  1.00
41.92           C
ATOM     53  O   GLU     5      16.338   1.361  15.069  1.00
74.72           O
ATOM     54  CB  GLU     5      14.354  -0.801  13.123  1.00
49.24           C
ATOM     55  CG  GLU     5      14.078  -1.443  14.476  1.00
55.48           C
ATOM     56  CD  GLU     5      12.950  -2.462  14.355  1.00
92.06           C
ATOM     57  OE1 GLU     5      12.453  -2.639  13.255
1.00206.49           O
```

```
ATOM     58  OE2 GLU     5      12.599  -3.051  15.364
1.00197.24           O1-
ATOM     59  HN  GLU     5      16.855  -1.065  14.564  1.00
45.52          H
ATOM     60  HA  GLU     5      15.854   0.430  12.247  1.00
60.63          H
ATOM     61  HB1 GLU     5      14.462  -1.572  12.377  1.00
61.59          H
ATOM     62  HB2 GLU     5      13.538  -0.155  12.859  1.00
63.66          H
ATOM     63  HG1 GLU     5      13.795  -0.679  15.185  1.00
58.03          H
ATOM     64  HG2 GLU     5      14.974  -1.933  14.817  1.00
57.77          H
ATOM     65  N   ASN     6      14.363   1.864  14.120  1.00
50.73          N
ATOM     66  CA  ASN     6      14.105   2.972  15.033  1.00
71.04          C
ATOM     67  C   ASN     6      14.059   2.469  16.473  1.00
65.17          C
ATOM     68  O   ASN     6      14.563   3.125  17.385  1.00
94.86          O
ATOM     69  CB  ASN     6      12.776   3.641  14.681  1.00
94.83          C
ATOM     70  CG  ASN     6      12.914   4.418  13.375
1.00160.94           C
ATOM     71  ND2 ASN     6      11.844   4.725  12.693
1.00244.88           N
ATOM     72  OD1 ASN     6      14.026   4.754  12.965
1.00219.73           O
ATOM     73  HN  ASN     6      13.708   1.649  13.423  1.00
72.81          H
ATOM     74  HA  ASN     6      14.899   3.697  14.939  1.00
96.02          H
ATOM     75  HB1 ASN     6      12.496   4.321  15.472
1.00111.09           H
ATOM     76  HB2 ASN     6      12.013   2.886  14.569  1.00
98.13          H
ATOM     77 HD21 ASN     6      10.961   4.457  13.021
1.00272.43           H
ATOM     78 HD22 ASN     6      11.925   5.224  11.853
1.00336.78           H
ATOM     79  N   GLY     7      13.456   1.297  16.669  1.00
51.80          N
ATOM     80  CA  GLY     7      13.347   0.696  18.001  1.00
71.47          C
ATOM     81  C   GLY     7      13.985  -0.686  18.019  1.00
41.04          C
ATOM     82  O   GLY     7      13.307  -1.694  18.214  1.00
44.54          O
ATOM     83  HN  GLY     7      13.079   0.822  15.899  1.00
45.49          H
```

```
ATOM     84  HA1 GLY     7      12.309   0.602  18.261
1.00103.52           H
ATOM     85  HA2 GLY     7      13.841   1.325  18.732
1.00108.67           H
ATOM     86  N   ASP     8      15.294  -0.723  17.813  1.00
27.00           N
ATOM     87  CA  ASP     8      16.023  -1.987  17.804  1.00
14.15           C
ATOM     88  C   ASP     8      15.896  -2.690  19.149  1.00
9.37           C
ATOM     89  O   ASP     8      15.642  -3.892  19.210  1.00
14.61           O
ATOM     90  CB  ASP     8      17.500  -1.738  17.493  1.00
15.06           C
ATOM     91  CG  ASP     8      18.243  -3.066  17.394  1.00
21.22           C
ATOM     92  OD1 ASP     8      17.640  -4.083  17.695
1.00119.56           O
ATOM     93  OD2 ASP     8      19.403  -3.048  17.019
1.00133.27           O1-
ATOM     94  HN  ASP     8      15.775   0.115  17.662  1.00
35.63           H
ATOM     95  HA  ASP     8      15.610  -2.622  17.040  1.00
20.92           H
ATOM     96  HB1 ASP     8      17.936  -1.144  18.282  1.00
29.39           H
ATOM     97  HB2 ASP     8      17.584  -1.210  16.556  1.00
44.14           H
ATOM     98  N   CYS     9      16.067  -1.930  20.222  1.00
5.93           N
ATOM     99  CA  CYS     9      15.962  -2.489  21.564  1.00
7.54           C
ATOM    100  C   CYS     9      14.556  -3.022  21.802  1.00
14.60           C
ATOM    101  O   CYS     9      14.369  -4.094  22.377  1.00
25.78           O
ATOM    102  CB  CYS     9      16.292  -1.423  22.598  1.00
6.63           C
ATOM    103  SG  CYS     9      18.059  -1.084  22.515  1.00
10.91           S
ATOM    104  HN  CYS     9      16.262  -0.978  20.107  1.00
6.51           H
ATOM    105  HA  CYS     9      16.674  -3.291  21.663  1.00
11.12           H
ATOM    106  HB1 CYS     9      16.042  -1.777  23.585  1.00
11.28           H
ATOM    107  HB2 CYS     9      15.737  -0.521  22.382  1.00
5.04           H
ATOM    108  N   ALA    10      13.573  -2.259  21.342  1.00
16.26           N
ATOM    109  CA  ALA    10      12.174  -2.639  21.485  1.00
32.04           C
```

ATOM 110 C ALA 10 11.292 -1.689 20.677 1.00
45.47 C
ATOM 111 O ALA 10 11.745 -0.631 20.244
1.00119.04 O
ATOM 112 CB ALA 10 11.765 -2.609 22.963 1.00
30.10 C
ATOM 113 HN ALA 10 13.796 -1.420 20.887 1.00
12.81 H
ATOM 114 HA ALA 10 12.045 -3.642 21.106 1.00
47.14 H
ATOM 115 HB1 ALA 10 12.591 -2.946 23.571 1.00
84.37 H
ATOM 116 HB2 ALA 10 10.918 -3.263 23.117
1.00118.20 H
ATOM 117 HB3 ALA 10 11.497 -1.603 23.246
1.00117.32 H
ATOM 118 N ALA 11 10.039 -2.073 20.472 1.00
30.62 N
ATOM 119 CA ALA 11 9.117 -1.239 19.710 1.00
35.24 C
ATOM 120 C ALA 11 8.928 0.118 20.385 1.00
22.78 C
ATOM 121 O ALA 11 8.882 1.150 19.716 1.00
53.33 O
ATOM 122 CB ALA 11 7.763 -1.943 19.584 1.00
60.49 C
ATOM 123 HN ALA 11 9.730 -2.929 20.837 1.00
50.37 H
ATOM 124 HA ALA 11 9.521 -1.086 18.721 1.00
41.70 H
ATOM 125 HB1 ALA 11 7.899 -2.903 19.110
1.00157.06 H
ATOM 126 HB2 ALA 11 7.097 -1.337 18.988
1.00148.83 H
ATOM 127 HB3 ALA 11 7.338 -2.082 20.567
1.00137.06 H
ATOM 128 N ASP 12 8.814 0.108 21.711 1.00
18.98 N
ATOM 129 CA ASP 12 8.625 1.346 22.465 1.00
31.05 C
ATOM 130 C ASP 12 9.958 2.038 22.737 1.00
22.98 C
ATOM 131 O ASP 12 10.027 3.266 22.805 1.00
37.14 O
ATOM 132 CB ASP 12 7.930 1.041 23.794 1.00
48.35 C
ATOM 133 CG ASP 12 6.488 0.613 23.540
1.00104.83 C
ATOM 134 OD1 ASP 12 6.013 0.828 22.437
1.00258.73 O
ATOM 135 OD2 ASP 12 5.881 0.077 24.453
1.00213.46 O1-

```
ATOM    136  HN  ASP    12        8.855  -0.745  22.191  1.00
36.19           H
ATOM    137  HA  ASP    12        7.997   2.012  21.894  1.00
49.76           H
ATOM    138  HB1 ASP    12        7.937   1.925  24.412  1.00
95.71           H
ATOM    139  HB2 ASP    12        8.456   0.246  24.299  1.00
54.37           H
ATOM    140  N   GLU    13       11.013   1.248  22.902  1.00
17.60           N
ATOM    141  CA  GLU    13       12.337   1.800  23.178  1.00
12.01           C
ATOM    142  C   GLU    13       12.946   2.427  21.925  1.00
10.07           C
ATOM    143  O   GLU    13       12.618   2.044  20.802  1.00
12.81           O
ATOM    144  CB  GLU    13       13.262   0.701  23.702  1.00
11.74           C
ATOM    145  CG  GLU    13       12.740   0.200  25.050  1.00
13.49           C
ATOM    146  CD  GLU    13       12.921   1.279  26.111
1.00142.36           C
ATOM    147  OE1 GLU    13       13.681   2.201  25.866
1.00339.06           O
ATOM    148  OE2 GLU    13       12.295   1.170  27.151
1.00335.28           O1-
ATOM    149  HN  GLU    13       10.899   0.275  22.845  1.00
28.36           H
ATOM    150  HA  GLU    13       12.242   2.560  23.938  1.00
13.06           H
ATOM    151  HB1 GLU    13       14.257   1.099  23.829  1.00
13.76           H
ATOM    152  HB2 GLU    13       13.287  -0.116  22.996  1.00
9.94           H
ATOM    153  HG1 GLU    13       13.289  -0.684  25.343  1.00
66.02           H
ATOM    154  HG2 GLU    13       11.691  -0.042  24.963  1.00
56.23           H
ATOM    155  N   CYS    14       13.842   3.395  22.133  1.00
9.12           N
ATOM    156  CA  CYS    14       14.511   4.083  21.025  1.00
10.69           C
ATOM    157  C   CYS    14       15.916   3.529  20.831  1.00
9.56           C
ATOM    158  O   CYS    14       16.506   2.977  21.759  1.00
13.62           O
ATOM    159  CB  CYS    14       14.596   5.583  21.317  1.00
13.96           C
ATOM    160  SG  CYS    14       15.403   6.424  19.928  1.00
42.96           S
ATOM    161  HN  CYS    14       14.064   3.650  23.053  1.00
9.76           H
```

```
ATOM    162  HA  CYS    14      13.946  3.936  20.114  1.00
15.54          H
ATOM    163  HB1 CYS    14      15.172  5.741  22.216  1.00
50.72          H
ATOM    164  HB2 CYS    14      13.601  5.981  21.450  1.00
46.13          H
ATOM    165  N   CYS    15      16.454  3.682  19.623  1.00
9.60          N
ATOM    166  CA  CYS    15      17.797  3.198  19.320  1.00
9.28          C
ATOM    167  C   CYS    15      18.409  4.062  18.227  1.00
9.92          C
ATOM    168  O   CYS    15      17.783  4.298  17.193  1.00
13.31          O
ATOM    169  CB  CYS    15      17.741  1.741  18.852  1.00
12.90          C
ATOM    170  SG  CYS    15      19.402  1.017  18.903  1.00
39.11          S
ATOM    171  HN  CYS    15      15.939  4.136  18.923  1.00
13.12          H
ATOM    172  HA  CYS    15      18.412  3.262  20.206  1.00
8.26          H
ATOM    173  HB1 CYS    15      17.363  1.703  17.839  1.00
24.82          H
ATOM    174  HB2 CYS    15      17.085  1.181  19.500  1.00
27.31          H
ATOM    175  N   VAL    16      19.628  4.542  18.454  1.00
9.56          N
ATOM    176  CA  VAL    16      20.292  5.387  17.468  1.00
11.99          C
ATOM    177  C   VAL    16      21.806  5.233  17.561  1.00
7.73          C
ATOM    178  O   VAL    16      22.382  5.291  18.648  1.00
8.83          O
ATOM    179  CB  VAL    16      19.896  6.847  17.698  1.00
18.37          C
ATOM    180  CG1 VAL    16      20.327  7.284  19.100  1.00
39.61          C
ATOM    181  CG2 VAL    16      20.575  7.730  16.652
1.00115.21          C
ATOM    182  HN  VAL    16      20.086  4.331  19.298  1.00
9.76          H
ATOM    183  HA  VAL    16      19.976  5.095  16.477  1.00
17.27          H
ATOM    184  HB  VAL    16      18.824  6.943  17.608  1.00
52.63          H
ATOM    185 HG11 VAL    16      19.879  8.239  19.333
1.00128.07          H
ATOM    186 HG12 VAL    16      21.403  7.374  19.135
1.00154.13          H
ATOM    187 HG13 VAL    16      20.002  6.549  19.821
1.00135.74          H
```

```
ATOM    188 HG21 VAL    16       21.641   7.730  16.818
1.00229.05           H
ATOM    189 HG22 VAL    16       20.198   8.738  16.733
1.00261.82           H
ATOM    190 HG23 VAL    16       20.363   7.345  15.667
1.00210.07           H
ATOM    191  N   ASP    17       22.444   5.028  16.411  1.00
14.34          N
ATOM    192  CA  ASP    17       23.894   4.859  16.362  1.00
11.90          C
ATOM    193  C   ASP    17       24.579   6.183  16.045  1.00
13.15          C
ATOM    194  O   ASP    17       24.347   6.779  14.992  1.00
25.96          O
ATOM    195  CB  ASP    17       24.259   3.831  15.288  1.00
20.80          C
ATOM    196  CG  ASP    17       23.814   2.440  15.725  1.00
28.72          C
ATOM    197  OD1 ASP    17       23.538   2.271  16.902
1.00135.42           O
ATOM    198  OD2 ASP    17       23.752   1.566  14.877
1.00114.17           O1-
ATOM    199  HN  ASP    17       21.928   4.986  15.578  1.00
28.29          H
ATOM    200  HA  ASP    17       24.249   4.500  17.319  1.00
10.49          H
ATOM    201  HB1 ASP    17       25.328   3.833  15.138  1.00
22.42          H
ATOM    202  HB2 ASP    17       23.767   4.092  14.362  1.00
29.52          H
ATOM    203  N   THR    18       25.434   6.632  16.960  1.00
11.55          N
ATOM    204  CA  THR    18       26.171   7.885  16.782  1.00
17.10          C
ATOM    205  C   THR    18       27.634   7.592  16.477  1.00
10.63          C
ATOM    206  O   THR    18       28.121   6.495  16.740  1.00
6.49          O
ATOM    207  CB  THR    18       26.074   8.738  18.051  1.00
28.52          C
ATOM    208  CG2 THR    18       24.697   9.391  18.138  1.00
45.38          C
ATOM    209  OG1 THR    18       26.285   7.912  19.185  1.00
26.49          O
ATOM    210  HN  THR    18       25.581   6.106  17.774  1.00
13.96          H
ATOM    211  HA  THR    18       25.748   8.441  15.955  1.00
26.43          H
ATOM    212  HB  THR    18       26.831   9.509  18.026  1.00
37.43          H
ATOM    213  HG1 THR    18       25.439   7.788  19.622  1.00
71.62          H
```

```
ATOM    214 HG21 THR    18       23.938   8.664  17.893
1.00100.44           H
ATOM    215 HG22 THR    18       24.648  10.215  17.440
1.00129.42           H
ATOM    216 HG23 THR    18       24.536   9.757  19.140
1.00156.21           H
ATOM    217  N   VAL    19       28.323   8.591  15.935  1.00
16.32           N
ATOM    218  CA  VAL    19       29.721   8.468  15.600  1.00
13.34           C
ATOM    219  C   VAL    19       30.184   9.751  14.936  1.00
25.88           C
ATOM    220  O   VAL    19       29.671  10.170  13.898  1.00
42.11           O
ATOM    221  CB  VAL    19       29.985   7.273  14.687  1.00
15.48           C
ATOM    222  CG1 VAL    19       28.996   7.277  13.517  1.00
29.06           C
ATOM    223  CG2 VAL    19       31.420   7.362  14.148  1.00
20.67           C
ATOM    224  HN  VAL    19       27.884   9.444  15.770  1.00
26.89           H
ATOM    225  HA  VAL    19       30.284   8.336  16.515  1.00
8.08           H
ATOM    226  HB  VAL    19       29.870   6.366  15.257  1.00
11.49           H
ATOM    227 HG11 VAL    19       27.988   7.362  13.897
1.00137.43           H
ATOM    228 HG12 VAL    19       29.094   6.356  12.961  1.00
93.29           H
ATOM    229 HG13 VAL    19       29.208   8.112  12.868
1.00108.69           H
ATOM    230 HG21 VAL    19       31.464   8.103  13.364  1.00
99.42           H
ATOM    231 HG22 VAL    19       31.721   6.402  13.757
1.00111.36           H
ATOM    232 HG23 VAL    19       32.086   7.652  14.950  1.00
77.11           H
ATOM    233  N   PHE    20       31.139  10.364  15.581  1.00
24.58           N
ATOM    234  CA  PHE    20       31.709  11.624  15.136  1.00
40.34           C
ATOM    235  C   PHE    20       32.950  11.402  14.275  1.00
40.37           C
ATOM    236  O   PHE    20       32.862  11.232  13.060  1.00
66.02           O
ATOM    237  CB  PHE    20       32.067  12.477  16.375  1.00
48.79           C
ATOM    238  CG  PHE    20       32.368  11.590  17.577  1.00
35.75           C
ATOM    239  CD1 PHE    20       31.344  10.833  18.180  1.00
30.20           C
```

ATOM 240 CD2 PHE 20 33.666 11.538 18.100 1.00
40.95 C
ATOM 241 CE1 PHE 20 31.629 10.033 19.290 1.00
30.41 C
ATOM 242 CE2 PHE 20 33.946 10.736 19.211 1.00
45.84 C
ATOM 243 CZ PHE 20 32.929 9.982 19.805 1.00
40.81 C
ATOM 244 HN PHE 20 31.456 9.963 16.405 1.00
16.73 H
ATOM 245 HA PHE 20 30.975 12.159 14.549 1.00
59.91 H
ATOM 246 HB1 PHE 20 31.241 13.104 16.613 1.00
67.15 H
ATOM 247 HB2 PHE 20 32.925 13.100 16.165 1.00
58.17 H
ATOM 248 HD1 PHE 20 30.337 10.858 17.782 1.00
33.34 H
ATOM 249 HD2 PHE 20 34.450 12.120 17.647 1.00
49.35 H
ATOM 250 HE1 PHE 20 30.845 9.456 19.750 1.00
30.61 H
ATOM 251 HE2 PHE 20 34.950 10.695 19.609 1.00
61.27 H
ATOM 252 HZ PHE 20 33.146 9.363 20.663 1.00
52.66 H
ATOM 253 N GLU 21 34.099 11.442 14.924 1.00
41.31 N
ATOM 254 CA GLU 21 35.377 11.285 14.246 1.00
56.16 C
ATOM 255 C GLU 21 35.686 9.808 13.979 1.00
58.61 C
ATOM 256 O GLU 21 36.523 9.489 13.136
1.00201.19 O
ATOM 257 CB GLU 21 36.457 11.946 15.123 1.00
57.37 C
ATOM 258 CG GLU 21 37.842 11.332 14.873
1.00193.56 C
ATOM 259 CD GLU 21 38.914 12.178 15.550
1.00304.62 C
ATOM 260 OE1 GLU 21 40.025 12.206 15.047
1.00451.81 O
ATOM 261 OE2 GLU 21 38.610 12.785 16.563
1.00442.88 O1-
ATOM 262 HN GLU 21 34.091 11.603 15.890 1.00
50.82 H
ATOM 263 HA GLU 21 35.335 11.805 13.299 1.00
85.72 H
ATOM 264 HB1 GLU 21 36.182 11.819 16.159 1.00
46.37 H
ATOM 265 HB2 GLU 21 36.496 13.005 14.899
1.00131.81 H

```
ATOM    266  HG1 GLU    21      38.032  11.296  13.810
1.00314.91           H
ATOM    267  HG2 GLU    21      37.869  10.331  15.278
1.00266.92           H
ATOM    268  N   GLY    22      35.011   8.916  14.696  1.00
86.70           N
ATOM    269  CA  GLY    22      35.230   7.480  14.519
1.00104.66           C
ATOM    270  C   GLY    22      36.271   6.956  15.501  1.00
71.50           C
ATOM    271  O   GLY    22      36.469   5.747  15.619  1.00
89.87           O
ATOM    272  HN  GLY    22      34.354   9.226  15.356
1.00214.64           H
ATOM    273  HA1 GLY    22      35.568   7.287  13.511
1.00152.47           H
ATOM    274  HA2 GLY    22      34.298   6.960  14.683
1.00118.15           H
ATOM    275  N   ASP    23      36.926   7.867  16.215  1.00
44.68           N
ATOM    276  CA  ASP    23      37.931   7.466  17.192  1.00
43.33           C
ATOM    277  C   ASP    23      37.287   6.571  18.241  1.00
37.55           C
ATOM    278  O   ASP    23      37.850   5.553  18.643  1.00
61.37           O
ATOM    279  CB  ASP    23      38.533   8.703  17.863  1.00
40.74           C
ATOM    280  CG  ASP    23      39.624   8.289  18.844
1.00141.86           C
ATOM    281  OD1 ASP    23      40.299   9.168  19.353
1.00328.18           O
ATOM    282  OD2 ASP    23      39.769   7.099  19.072
1.00304.58           O1-
ATOM    283  HN  ASP    23      36.724   8.818  16.089  1.00
40.86           H
ATOM    284  HA  ASP    23      38.714   6.920  16.691  1.00
72.12           H
ATOM    285  HB1 ASP    23      37.757   9.235  18.395  1.00
94.15           H
ATOM    286  HB2 ASP    23      38.956   9.349  17.109  1.00
93.26           H
ATOM    287  N   MET    24      36.089   6.960  18.661  1.00
23.24           N
ATOM    288  CA  MET    24      35.328   6.201  19.650  1.00
32.50           C
ATOM    289  C   MET    24      33.849   6.247  19.290  1.00
25.13           C
ATOM    290  O   MET    24      33.189   7.268  19.483  1.00
54.40           O
ATOM    291  CB  MET    24      35.542   6.795  21.044  1.00
47.36           C
```

ATOM 292 CG MET 24 34.742 5.992 22.073
1.00151.73 C
ATOM 293 SD MET 24 35.146 6.574 23.740
1.00209.23 S
ATOM 294 CE MET 24 34.346 8.197 23.633
1.00243.29 C
ATOM 295 HN MET 24 35.698 7.775 18.285 1.00
17.32 H
ATOM 296 HA MET 24 35.658 5.170 19.650 1.00
48.94 H
ATOM 297 HB1 MET 24 35.207 7.820 21.051
1.00124.40 H
ATOM 298 HB2 MET 24 36.592 6.757 21.294
1.00166.96 H
ATOM 299 HG1 MET 24 34.995 4.946 21.986
1.00331.71 H
ATOM 300 HG2 MET 24 33.687 6.123 21.891
1.00302.30 H
ATOM 301 HE1 MET 24 35.005 8.887 23.126
1.00340.44 H
ATOM 302 HE2 MET 24 34.142 8.563 24.626
1.00373.88 H
ATOM 303 HE3 MET 24 33.419 8.106 23.087
1.00386.81 H
ATOM 304 N VAL 25 33.333 5.146 18.755 1.00
22.44 N
ATOM 305 CA VAL 25 31.930 5.099 18.362 1.00
15.37 C
ATOM 306 C VAL 25 31.021 5.169 19.580 1.00
17.47 C
ATOM 307 O VAL 25 31.360 4.680 20.658 1.00
29.52 O
ATOM 308 CB VAL 25 31.623 3.830 17.565 1.00
23.36 C
ATOM 309 CG1 VAL 25 30.125 3.794 17.238 1.00
58.39 C
ATOM 310 CG2 VAL 25 32.431 3.836 16.266 1.00
55.25 C
ATOM 311 HN VAL 25 33.903 4.363 18.612 1.00
48.11 H
ATOM 312 HA VAL 25 31.724 5.953 17.733 1.00
9.25 H
ATOM 313 HB VAL 25 31.884 2.962 18.155 1.00
52.45 H
ATOM 314 HG11 VAL 25 29.574 3.474 18.109
1.00171.07 H
ATOM 315 HG12 VAL 25 29.946 3.105 16.426
1.00166.22 H
ATOM 316 HG13 VAL 25 29.793 4.785 16.950
1.00134.76 H
ATOM 317 HG21 VAL 25 33.465 4.058 16.484
1.00141.88 H

```
ATOM    318 HG22 VAL    25       32.034   4.588  15.599
1.00184.50            H
ATOM    319 HG23 VAL    25       32.362   2.866  15.795
1.00133.90            H
ATOM    320  N   THR    26       29.865   5.797  19.392  1.00
13.60            N
ATOM    321  CA  THR    26       28.884   5.964  20.464  1.00
21.19            C
ATOM    322  C   THR    26       27.520   5.421  20.045  1.00
16.55            C
ATOM    323  O   THR    26       27.048   5.682  18.939  1.00
11.43            O
ATOM    324  CB  THR    26       28.771   7.449  20.792  1.00
27.07            C
ATOM    325  CG2 THR    26       27.691   7.673  21.856  1.00
44.39            C
ATOM    326  OG1 THR    26       30.021   7.911  21.281  1.00
34.75            O
ATOM    327  HN  THR    26       29.666   6.170  18.507  1.00
10.81            H
ATOM    328  HA  THR    26       29.215   5.437  21.348  1.00
33.16            H
ATOM    329  HB  THR    26       28.514   7.990  19.890  1.00
20.60            H
ATOM    330  HG1 THR    26       30.715   7.411  20.842  1.00
77.72            H
ATOM    331 HG21 THR    26       27.872   7.016  22.694
1.00126.99            H
ATOM    332 HG22 THR    26       26.720   7.464  21.438
1.00108.32            H
ATOM    333 HG23 THR    26       27.725   8.700  22.190
1.00151.99            H
ATOM    334  N   ARG    27       26.890   4.665  20.945  1.00
22.38            N
ATOM    335  CA  ARG    27       25.571   4.080  20.683  1.00
20.21            C
ATOM    336  C   ARG    27       24.627   4.388  21.841  1.00
16.96            C
ATOM    337  O   ARG    27       25.054   4.455  22.994  1.00
20.06            O
ATOM    338  CB  ARG    27       25.703   2.564  20.527  1.00
22.14            C
ATOM    339  CG  ARG    27       26.589   2.247  19.320
1.00124.30            C
ATOM    340  CD  ARG    27       26.818   0.738  19.238
1.00109.38            C
ATOM    341  NE  ARG    27       27.709   0.420  18.130
1.00227.73            N
ATOM    342  CZ  ARG    27       28.129  -0.824  17.921
1.00426.12            C
ATOM    343  NH1 ARG    27       28.928  -1.085  16.922
1.00767.09            N1+
```

```
ATOM    344  NH2 ARG    27      27.745  -1.784  18.716
1.00581.78           N
ATOM    345  HN  ARG    27      27.320   4.498  21.811  1.00
30.75          H
ATOM    346  HA  ARG    27      25.161   4.494  19.773  1.00
23.13          H
ATOM    347  HB1 ARG    27      24.726   2.131  20.376  1.00
87.46          H
ATOM    348  HB2 ARG    27      26.148   2.149  21.419
1.00103.36           H
ATOM    349  HG1 ARG    27      27.539   2.749  19.430
1.00281.97           H
ATOM    350  HG2 ARG    27      26.103   2.589  18.418
1.00276.19           H
ATOM    351  HD1 ARG    27      25.870   0.242  19.092
1.00183.60           H
ATOM    352  HD2 ARG    27      27.261   0.393  20.161
1.00142.93           H
ATOM    353  HE  ARG    27      28.004   1.135  17.528
1.00372.53           H
ATOM    354 HH11 ARG    27      29.223  -0.348  16.313
1.00910.59           H
ATOM    355 HH12 ARG    27      29.244  -2.020  16.766
1.00999.99           H
ATOM    356 HH21 ARG    27      27.134  -1.585  19.482
1.00532.54           H
ATOM    357 HH22 ARG    27      28.060  -2.719  18.559
1.00948.84           H
ATOM    358  N   SER    28      23.340   4.576  21.536  1.00
14.60          N
ATOM    359  CA  SER    28      22.349   4.878  22.573  1.00
14.92          C
ATOM    360  C   SER    28      21.100   4.027  22.386  1.00
12.12          C
ATOM    361  O   SER    28      20.700   3.732  21.261  1.00
13.41          O
ATOM    362  CB  SER    28      21.972   6.358  22.518  1.00
23.26          C
ATOM    363  OG  SER    28      23.124   7.147  22.780
1.00146.32           O
ATOM    364  HN  SER    28      23.049   4.512  20.600  1.00
15.23          H
ATOM    365  HA  SER    28      22.769   4.665  23.549  1.00
15.44          H
ATOM    366  HB1 SER    28      21.206   6.561  23.255  1.00
88.73          H
ATOM    367  HB2 SER    28      21.595   6.598  21.537
1.00124.68           H
ATOM    368  HG  SER    28      23.897   6.591  22.663
1.00242.47           H
ATOM    369  N   CYS    29      20.483   3.643  23.500  1.00
10.48          N
```

```
ATOM    370  CA  CYS    29      19.272   2.833  23.452  1.00
9.97          C
ATOM    371  C   CYS    29      18.684   2.690  24.853  1.00
11.60          C
ATOM    372  O   CYS    29      19.310   2.115  25.743  1.00
15.64          O
ATOM    373  CB  CYS    29      19.583   1.448  22.856  1.00
9.52          C
ATOM    374  SG  CYS    29      18.151   0.843  21.946  1.00
10.40          S
ATOM    375  HN  CYS    29      20.844   3.917  24.368  1.00
11.10          H
ATOM    376  HA  CYS    29      18.552   3.333  22.822  1.00
11.08          H
ATOM    377  HB1 CYS    29      19.813   0.743  23.646  1.00
9.33          H
ATOM    378  HB2 CYS    29      20.423   1.518  22.186  1.00
10.78          H
ATOM    379  N   GLU    30      17.482   3.223  25.045  1.00
13.52          N
ATOM    380  CA  GLU    30      16.826   3.152  26.345  1.00
17.41          C
ATOM    381  C   GLU    30      16.178   1.789  26.552  1.00
10.81          C
ATOM    382  O   GLU    30      15.811   1.112  25.592  1.00
23.22          O
ATOM    383  CB  GLU    30      15.764   4.247  26.451  1.00
37.83          C
ATOM    384  CG  GLU    30      16.433   5.614  26.303
1.00108.13          C
ATOM    385  CD  GLU    30      17.329   5.887  27.504
1.00240.52          C
ATOM    386  OE1 GLU    30      17.151   5.227  28.516
1.00422.56          O
ATOM    387  OE2 GLU    30      18.182   6.754  27.398
1.00410.81          O1-
ATOM    388  HN  GLU    30      17.028   3.673  24.300  1.00
15.61          H
ATOM    389  HA  GLU    30      17.562   3.310  27.118  1.00
24.60          H
ATOM    390  HB1 GLU    30      15.279   4.188  27.414  1.00
72.67          H
ATOM    391  HB2 GLU    30      15.032   4.117  25.668  1.00
56.47          H
ATOM    392  HG1 GLU    30      15.674   6.378  26.241
1.00196.66          H
ATOM    393  HG2 GLU    30      17.027   5.624  25.401
1.00140.67          H
ATOM    394  N   LYS    31      16.038   1.394  27.816  1.00
11.96          N
ATOM    395  CA  LYS    31      15.429   0.107  28.160  1.00
11.40          C
```

```
ATOM    396  C   LYS    31       14.144   0.332  28.949  1.00
10.99          C
ATOM    397  O   LYS    31       14.061   1.246  29.769  1.00
12.86          O
ATOM    398  CB  LYS    31       16.402  -0.721  29.003  1.00
21.40          C
ATOM    399  CG  LYS    31       17.661  -1.015  28.186  1.00
57.81          C
ATOM    400  CD  LYS    31       18.634  -1.842  29.027
1.00115.52          C
ATOM    401  CE  LYS    31       19.898  -2.130  28.213
1.00250.23          C
ATOM    402  NZ  LYS    31       20.849  -2.928  29.037
1.00462.10          N1+
ATOM    403  HN  LYS    31       16.350   1.980  28.536  1.00
26.13          H
ATOM    404  HA  LYS    31       15.195  -0.441  27.257  1.00
13.87          H
ATOM    405  HB1 LYS    31       15.932  -1.651  29.284  1.00
37.63          H
ATOM    406  HB2 LYS    31       16.666  -0.167  29.891  1.00
47.19          H
ATOM    407  HG1 LYS    31       18.134  -0.086  27.903
1.00126.32          H
ATOM    408  HG2 LYS    31       17.392  -1.568  27.296
1.00114.19          H
ATOM    409  HD1 LYS    31       18.168  -2.777  29.304
1.00198.45          H
ATOM    410  HD2 LYS    31       18.897  -1.293  29.919
1.00200.14          H
ATOM    411  HE1 LYS    31       20.363  -1.196  27.930
1.00374.64          H
ATOM    412  HE2 LYS    31       19.636  -2.686  27.325
1.00403.27          H
ATOM    413  HZ1 LYS    31       21.740  -2.404  29.144
1.00627.38          H
ATOM    414  HZ2 LYS    31       21.035  -3.838  28.568
1.00622.26          H
ATOM    415  HZ3 LYS    31       20.436  -3.099  29.975
1.00619.20          H
ATOM    416  N   THR    32       13.145  -0.503  28.694  1.00
15.51          N
ATOM    417  CA  THR    32       11.870  -0.380  29.385  1.00
22.44          C
ATOM    418  C   THR    32       12.030  -0.729  30.860  1.00
26.60          C
ATOM    419  O   THR    32       12.530  -1.798  31.209  1.00
57.43          O
ATOM    420  CB  THR    32       10.835  -1.305  28.737  1.00
56.28          C
ATOM    421  CG2 THR    32        9.484  -1.139  29.435
1.00102.70          C
```

```
ATOM    422  OG1 THR    32       10.700  -0.970  27.363
1.00111.17           O
ATOM    423  HN  THR    32       13.268  -1.210  28.029  1.00
18.79           H
ATOM    424  HA  THR    32       11.524   0.639  29.304  1.00
19.67           H
ATOM    425  HB  THR    32       11.158  -2.328  28.829  1.00
84.25           H
ATOM    426  HG1 THR    32       11.578  -0.932  26.979
1.00205.03           H
ATOM    427 HG21 THR    32        9.566  -1.454  30.464
1.00220.71           H
ATOM    428 HG22 THR    32        8.744  -1.745  28.932
1.00174.67           H
ATOM    429 HG23 THR    32        9.186  -0.101  29.398
1.00217.44           H
ATOM    430  N   THR    33       11.597   0.186  31.722  1.00
22.47           N
ATOM    431  CA  THR    33       11.687  -0.017  33.165  1.00
41.60           C
ATOM    432  C   THR    33       10.588   0.727  33.872  1.00
38.03           C
ATOM    433  O   THR    33       10.756   1.873  34.290  1.00
52.73           O
ATOM    434  CB  THR    33       13.047   0.450  33.687  1.00
63.25           C
ATOM    435  CG2 THR    33       14.144  -0.487  33.179
1.00121.65           C
ATOM    436  OG1 THR    33       13.301   1.771  33.232  1.00
96.20           O
ATOM    437  HN  THR    33       11.207   1.017  31.377  1.00
23.82           H
ATOM    438  HA  THR    33       11.560  -1.057  33.393  1.00
67.12           H
ATOM    439  HB  THR    33       13.039   0.435  34.765
1.00107.59           H
ATOM    440  HG1 THR    33       13.865   2.204  33.877
1.00176.27           H
ATOM    441 HG21 THR    33       13.857  -1.512  33.363
1.00255.23           H
ATOM    442 HG22 THR    33       15.067  -0.274  33.697
1.00245.37           H
ATOM    443 HG23 THR    33       14.282  -0.337  32.119
1.00185.78           H
ATOM    444  N   GLY    34        9.465   0.049  34.021  1.00
40.93           N
ATOM    445  CA  GLY    34        8.339   0.638  34.701  1.00
45.59           C
ATOM    446  C   GLY    34        7.680   1.709  33.839  1.00
40.97           C
ATOM    447  O   GLY    34        6.460   1.729  33.681  1.00
82.17           O
```

```
ATOM    448  HN  GLY    34       9.402  -0.866  33.679  1.00
54.00          H
ATOM    449  HA1 GLY    34       8.701   1.079  35.609  1.00
54.41          H
ATOM    450  HA2 GLY    34       7.619  -0.133  34.936  1.00
62.09          H
ATOM    451  N   ASN    35       8.502   2.597  33.278  1.00
32.51          N
ATOM    452  CA  ASN    35       8.003   3.674  32.419  1.00
33.20          C
ATOM    453  C   ASN    35       8.843   3.765  31.148  1.00
23.17          C
ATOM    454  O   ASN    35      10.067   3.642  31.193  1.00
27.01          O
ATOM    455  CB  ASN    35       8.064   5.007  33.166  1.00
54.51          C
ATOM    456  CG  ASN    35       7.212   4.937  34.428  1.00
80.73          C
ATOM    457  ND2 ASN    35       7.670   5.437  35.543
1.00220.46          N
ATOM    458  OD1 ASN    35       6.099   4.412  34.398
1.00117.80          O
ATOM    459  HN  ASN    35       9.464   2.523  33.439  1.00
51.15          H
ATOM    460  HA  ASN    35       6.976   3.474  32.145  1.00
47.21          H
ATOM    461  HB1 ASN    35       7.691   5.792  32.526  1.00
68.31          H
ATOM    462  HB2 ASN    35       9.088   5.219  33.436  1.00
57.75          H
ATOM    463 HD21 ASN    35       8.555   5.854  35.565
1.00405.39          H
ATOM    464 HD22 ASN    35       7.127   5.396  36.358
1.00245.64          H
ATOM    465  N   PHE    36       8.176   3.974  30.018  1.00
35.72          N
ATOM    466  CA  PHE    36       8.861   4.073  28.739  1.00
33.41          C
ATOM    467  C   PHE    36       9.397   5.488  28.525  1.00
28.51          C
ATOM    468  O   PHE    36       8.786   6.464  28.958  1.00
45.23          O
ATOM    469  CB  PHE    36       7.882   3.722  27.623  1.00
67.53          C
ATOM    470  CG  PHE    36       7.479   2.271  27.745  1.00
86.71          C
ATOM    471  CD1 PHE    36       6.382   1.919  28.541
1.00106.51          C
ATOM    472  CD2 PHE    36       8.194   1.280  27.061  1.00
99.48          C
ATOM    473  CE1 PHE    36       6.000   0.578  28.654
1.00134.48          C
```

```
ATOM    474  CE2 PHE    36       7.813  -0.061  27.174
1.00134.27           C
ATOM    475  CZ  PHE    36       6.716  -0.413  27.971
1.00149.48           C
ATOM    476  HN  PHE    36       7.203   4.058  30.040  1.00
62.87            H
ATOM    477  HA  PHE    36       9.683   3.374  28.718  1.00
28.53            H
ATOM    478  HB1 PHE    36       8.354   3.884  26.672  1.00
73.56            H
ATOM    479  HB2 PHE    36       7.006   4.346  27.702  1.00
88.42            H
ATOM    480  HD1 PHE    36       5.832   2.685  29.069
1.00110.20           H
ATOM    481  HD2 PHE    36       9.042   1.550  26.449  1.00
93.07            H
ATOM    482  HE1 PHE    36       5.155   0.307  29.268
1.00155.06           H
ATOM    483  HE2 PHE    36       8.364  -0.825  26.647
1.00159.46           H
ATOM    484  HZ  PHE    36       6.421  -1.448  28.057
1.00183.25           H
ATOM    485  N   THR    37      10.543   5.589  27.850  1.00
20.06            N
ATOM    486  CA  THR    37      11.162   6.885  27.576  1.00
27.18            C
ATOM    487  C   THR    37      11.588   6.964  26.115  1.00
41.87            C
ATOM    488  O   THR    37      10.918   6.429  25.232
1.00169.86           O
ATOM    489  CB  THR    37      12.383   7.081  28.478  1.00
26.52            C
ATOM    490  CG2 THR    37      11.987   6.832  29.933  1.00
75.69            C
ATOM    491  OG1 THR    37      13.400   6.164  28.098  1.00
81.95            O
ATOM    492  HN  THR    37      10.983   4.778  27.529  1.00
18.47            H
ATOM    493  HA  THR    37      10.452   7.677  27.775  1.00
38.80            H
ATOM    494  HB  THR    37      12.750   8.090  28.375  1.00
81.60            H
ATOM    495  HG1 THR    37      13.453   6.160  27.140
1.00188.80           H
ATOM    496 HG21 THR    37      11.098   7.398  30.167
1.00196.98           H
ATOM    497 HG22 THR    37      12.792   7.143  30.584
1.00196.23           H
ATOM    498 HG23 THR    37      11.793   5.781  30.081
1.00168.92           H
ATOM    499  N   GLU    38      12.704   7.637  25.870  1.00
26.53            N
```

```
ATOM    500  CA  GLU    38       13.219   7.787  24.512  1.00
30.29          C
ATOM    501  C   GLU    38       14.712   8.102  24.532  1.00
25.39          C
ATOM    502  O   GLU    38       15.244   8.575  25.536  1.00
51.60          O
ATOM    503  CB  GLU    38       12.465   8.907  23.790  1.00
54.82          C
ATOM    504  CG  GLU    38       12.671  10.228  24.534
1.00165.84          C
ATOM    505  CD  GLU    38       11.820  11.322  23.898
1.00292.92          C
ATOM    506  OE1 GLU    38       11.872  12.441  24.380
1.00414.19          O
ATOM    507  OE2 GLU    38       11.130  11.025  22.936
1.00537.65          O1-
ATOM    508  HN  GLU    38       13.193   8.040  26.618  1.00
84.50          H
ATOM    509  HA  GLU    38       13.065   6.863  23.975  1.00
32.15          H
ATOM    510  HB1 GLU    38       11.412   8.672  23.762
1.00144.82          H
ATOM    511  HB2 GLU    38       12.841   9.000  22.780
1.00106.49          H
ATOM    512  HG1 GLU    38       13.711  10.511  24.482
1.00303.31          H
ATOM    513  HG2 GLU    38       12.382  10.106  25.568
1.00323.99          H
ATOM    514  N   CYS    39       15.380   7.842  23.411  1.00
15.84          N
ATOM    515  CA  CYS    39       16.811   8.102  23.306  1.00
14.30          C
ATOM    516  C   CYS    39       17.099   9.581  23.602  1.00
22.31          C
ATOM    517  O   CYS    39       16.240  10.435  23.384  1.00
32.92          O
ATOM    518  CB  CYS    39       17.292   7.736  21.897  1.00
18.08          C
ATOM    519  SG  CYS    39       16.018   8.191  20.693  1.00
17.41          S
ATOM    520  HN  CYS    39       14.902   7.470  22.642  1.00
24.87          H
ATOM    521  HA  CYS    39       17.326   7.484  24.023  1.00
14.60          H
ATOM    522  HB1 CYS    39       17.474   6.673  21.840  1.00
27.71          H
ATOM    523  HB2 CYS    39       18.207   8.268  21.671  1.00
34.91          H
ATOM    524  N   PRO    40       18.274   9.902  24.099  1.00
27.27          N
ATOM    525  CA  PRO    40       18.643  11.309  24.428  1.00
47.66          C
```

```
ATOM    526  C   PRO    40      18.929  12.139  23.179  1.00
71.54           C
ATOM    527  O   PRO    40      19.626  11.694  22.267  1.00
87.70           O
ATOM    528  CB  PRO    40      19.899  11.151  25.293  1.00
53.85           C
ATOM    529  CG  PRO    40      20.534   9.894  24.798  1.00
46.04           C
ATOM    530  CD  PRO    40      19.379   8.973  24.395  1.00
25.70           C
ATOM    531  HA  PRO    40      17.860  11.772  25.010  1.00
58.04           H
ATOM    532  HB1 PRO    40      19.630  11.045  26.334  1.00
67.93           H
ATOM    533  HB2 PRO    40      20.565  11.996  25.160  1.00
70.99           H
ATOM    534  HG1 PRO    40      21.117   9.431  25.581  1.00
65.91           H
ATOM    535  HG2 PRO    40      21.164  10.108  23.942  1.00
54.99           H
ATOM    536  HD1 PRO    40      19.111   8.320  25.211  1.00
24.22           H
ATOM    537  HD2 PRO    40      19.639   8.403  23.517  1.00
26.94           H
ATOM    538  N   GLY    41      18.391  13.349  23.158
1.00105.24           N
ATOM    539  CA  GLY    41      18.592  14.253  22.032
1.00147.55           C
ATOM    540  C   GLY    41      20.061  14.637  21.905
1.00161.74           C
ATOM    541  O   GLY    41      20.594  14.742  20.801
1.00244.37           O
ATOM    542  HN  GLY    41      17.852  13.642  23.920
1.00121.79           H
ATOM    543  HA1 GLY    41      18.004  15.147  22.182
1.00184.40           H
ATOM    544  HA2 GLY    41      18.272  13.766  21.125
1.00157.34           H
ATOM    545  N   LEU    42      20.703  14.852  23.049
1.00162.48           N
ATOM    546  CA  LEU    42      22.117  15.233  23.083
1.00191.48           C
ATOM    547  C   LEU    42      22.977  14.040  23.488
1.00166.66           C
ATOM    548  O   LEU    42      22.574  13.223  24.314
1.00251.38           O
ATOM    549  CB  LEU    42      22.324  16.368  24.089
1.00307.99           C
ATOM    550  CG  LEU    42      21.389  17.538  23.757
1.00452.28           C
ATOM    551  CD1 LEU    42      21.586  18.648  24.794
1.00681.24           C
```

ATOM 552 CD2 LEU 42 21.704 18.081 22.351
1.00527.46 C
ATOM 553 HN LEU 42 20.215 14.755 23.893
1.00195.47 H
ATOM 554 HA LEU 42 22.428 15.571 22.104
1.00229.77 H
ATOM 555 HB1 LEU 42 23.348 16.704 24.044
1.00342.49 H
ATOM 556 HB2 LEU 42 22.108 16.008 25.085
1.00335.33 H
ATOM 557 HG LEU 42 20.365 17.196 23.790
1.00427.79 H
ATOM 558 HD11 LEU 42 20.823 19.400 24.665
1.00909.71 H
ATOM 559 HD12 LEU 42 22.560 19.097 24.661
1.00706.80 H
ATOM 560 HD13 LEU 42 21.515 18.229 25.787
1.00858.82 H
ATOM 561 HD21 LEU 42 21.357 19.102 22.269
1.00631.69 H
ATOM 562 HD22 LEU 42 21.200 17.477 21.611
1.00628.06 H
ATOM 563 HD23 LEU 42 22.771 18.049 22.177
1.00656.75 H
ATOM 564 N THR 43 24.166 13.949 22.899
1.00178.52 N
ATOM 565 CA THR 43 25.078 12.852 23.203
1.00240.84 C
ATOM 566 C THR 43 25.449 12.864 24.693
1.00372.31 C
ATOM 567 O THR 43 25.614 13.935 25.277
1.00500.13 O
ATOM 568 CB THR 43 26.347 12.988 22.358
1.00354.13 C
ATOM 569 CG2 THR 43 27.113 14.240 22.783
1.00463.04 C
ATOM 570 OG1 THR 43 27.164 11.841 22.544
1.00508.50 O
ATOM 571 HN THR 43 24.433 14.630 22.249
1.00229.59 H
ATOM 572 HA THR 43 24.590 11.927 22.953
1.00249.90 H
ATOM 573 HB THR 43 26.078 13.073 21.316
1.00481.58 H
ATOM 574 HG1 THR 43 26.880 11.169 21.918
1.00624.31 H
ATOM 575 HG21 THR 43 27.883 14.455 22.056
1.00650.95 H
ATOM 576 HG22 THR 43 27.567 14.076 23.749
1.00580.73 H
ATOM 577 HG23 THR 43 26.432 15.077 22.843
1.00571.99 H

```
ATOM    578  N   PRO    44       25.581  11.716  25.325
1.00488.39          N
ATOM    579  CA  PRO    44       25.938  11.642  26.774
1.00760.13          C
ATOM    580  C   PRO    44       27.406  11.983  27.004
1.00735.36          C
ATOM    581  O   PRO    44       27.844  12.169  28.140
1.00999.99          O
ATOM    582  CB  PRO    44       25.634  10.186  27.143
1.00999.99          C
ATOM    583  CG  PRO    44       25.855   9.428  25.876
1.00856.94          C
ATOM    584  CD  PRO    44       25.425  10.365  24.745
1.00548.09          C
ATOM    585  HA  PRO    44       25.307  12.302  27.348
1.00939.00          H
ATOM    586  HB1 PRO    44       24.606  10.085  27.461
1.00999.99          H
ATOM    587  HB2 PRO    44       26.305   9.841  27.921
1.00999.99          H
ATOM    588  HG1 PRO    44       25.250   8.532  25.862
1.00999.99          H
ATOM    589  HG2 PRO    44       26.904   9.172  25.777
1.00853.53          H
ATOM    590  HD1 PRO    44       24.394  10.191  24.478
1.00602.45          H
ATOM    591  HD2 PRO    44       26.071  10.243  23.885
1.00463.32          H
ATOM    592  N   ILE    45       28.162  12.061  25.914
1.00518.33          N
ATOM    593  CA  ILE    45       29.582  12.374  26.000
1.00582.79          C
ATOM    594  C   ILE    45       29.782  13.747  26.632
1.00795.93          C
ATOM    595  O   ILE    45       30.635  13.925  27.501
1.00898.75          O
ATOM    596  CB  ILE    45       30.209  12.346  24.602
1.00474.41          C
ATOM    597  CG1 ILE    45       30.211  10.908  24.076
1.00516.90          C
ATOM    598  CG2 ILE    45       31.649  12.861  24.667
1.00760.75          C
ATOM    599  CD1 ILE    45       30.567  10.906  22.589
1.00566.81          C
ATOM    600  HN  ILE    45       27.758  11.899  25.036
1.00395.72          H
ATOM    601  HA  ILE    45       30.062  11.633  26.613
1.00711.06          H
ATOM    602  HB  ILE    45       29.633  12.973  23.936
1.00477.81          H
ATOM    603 HG11 ILE    45       29.233  10.473  24.213
1.00624.28          H
```

```
ATOM    604 HG12 ILE    45       30.942  10.329  24.624
1.00744.84          H
ATOM    605 HG21 ILE    45       32.159  12.400  25.501
1.00920.57          H
ATOM    606 HG22 ILE    45       31.643  13.933  24.800
1.00999.99          H
ATOM    607 HG23 ILE    45       32.163  12.616  23.751
1.00865.46          H
ATOM    608 HD11 ILE    45       29.724  11.267  22.018
1.00658.89          H
ATOM    609 HD12 ILE    45       30.808   9.900  22.278
1.00734.30          H
ATOM    610 HD13 ILE    45       31.419  11.548  22.422
1.00675.84          H
ATOM    611  N   ALA    46       28.989  14.711  26.188
1.00999.99          N
ATOM    612  CA  ALA    46       29.080  16.070  26.711
1.00999.99          C
ATOM    613  C   ALA    46       27.937  16.927  26.176
1.00999.99          C
ATOM    614  CB  ALA    46       30.418  16.695  26.313
1.00999.99          C
ATOM    615  OT1 ALA    46       26.953  17.074  26.881
1.00999.99          O
ATOM    616  OT2 ALA    46       28.065  17.425  25.070
1.00999.99          O
ATOM    617  HN  ALA    46       28.329  14.504  25.495
1.00999.99          H
ATOM    618  HA  ALA    46       29.019  16.035  27.788
1.00999.99          H
ATOM    619  HB1 ALA    46       31.226  16.084  26.689
1.00999.99          H
ATOM    620  HB2 ALA    46       30.491  17.687  26.732
1.00999.99          H
ATOM    621  HB3 ALA    46       30.482  16.752  25.237
1.00999.99          H
ENDMDL
TER
MODEL        6
ATOM      1  N   GLY     1       25.876  -2.630  12.500
1.00999.99          N
ATOM      2  CA  GLY     1       25.932  -2.082  13.885
1.00999.99          C
ATOM      3  C   GLY     1       24.884  -0.985  14.042
1.00999.99          C
ATOM      4  O   GLY     1       24.421  -0.710  15.148
1.00999.99          O
ATOM      5  HA1 GLY     1       26.912  -1.668  14.070
1.00999.99          H
ATOM      6  HA2 GLY     1       25.736  -2.874  14.594
1.00999.99          H
ATOM      7  HT1 GLY     1       25.209  -3.426  12.470
```

```
1.00999.99           H
ATOM      8  HT2 GLY     1       26.822  -2.959  12.219
1.00999.99           H
ATOM      9  HT3 GLY     1       25.559  -1.888  11.846
1.00999.99           H
ATOM     10  N   LEU     2       24.517  -0.361  12.927
1.00999.99           N
ATOM     11  CA  LEU     2       23.523   0.706  12.952
1.00895.59           C
ATOM     12  C   LEU     2       22.134   0.130  13.210
1.00532.27           C
ATOM     13  O   LEU     2       21.787  -0.930  12.689
1.00625.21           O
ATOM     14  CB  LEU     2       23.520   1.443  11.608
1.00999.99           C
ATOM     15  CG  LEU     2       24.920   1.996  11.306
1.00999.99           C
ATOM     16  CD1 LEU     2       24.916   2.630   9.912
1.00999.99           C
ATOM     17  CD2 LEU     2       25.307   3.056  12.352
1.00999.99           C
ATOM     18  HN  LEU     2       24.921  -0.623  12.074
1.00999.99           H
ATOM     19  HA  LEU     2       23.766   1.402  13.736
1.00890.87           H
ATOM     20  HB1 LEU     2       22.814   2.258  11.648
1.00924.07           H
ATOM     21  HB2 LEU     2       23.231   0.757  10.825
1.00999.99           H
ATOM     22  HG  LEU     2       25.636   1.187  11.327
1.00999.99           H
ATOM     23 HD11 LEU     2       24.227   3.463   9.896
1.00999.99           H
ATOM     24 HD12 LEU     2       24.609   1.897   9.182
1.00999.99           H
ATOM     25 HD13 LEU     2       25.909   2.982   9.675
1.00999.99           H
ATOM     26 HD21 LEU     2       26.068   3.707  11.945
1.00999.99           H
ATOM     27 HD22 LEU     2       25.696   2.567  13.233
1.00999.99           H
ATOM     28 HD23 LEU     2       24.439   3.642  12.618
1.00999.99           H
ATOM     29  N   CYS     3       21.338   0.835  14.011
1.00271.28           N
ATOM     30  CA  CYS     3       19.987   0.373  14.315
1.00104.50           C
ATOM     31  C   CYS     3       19.060   0.640  13.131  1.00
90.98           C
ATOM     32  O   CYS     3       18.835   1.788  12.750
1.00207.28           O
ATOM     33  CB  CYS     3       19.457   1.072  15.582  1.00
```

```
37.83           C
ATOM     34  SG  CYS     3       19.910   0.100  17.043  1.00
81.56           S
ATOM     35  HN  CYS     3       21.660   1.675  14.397
1.00296.55           H
ATOM     36  HA  CYS     3       20.018  -0.691  14.494
1.00166.45           H
ATOM     37  HB1 CYS     3       18.380   1.161  15.532  1.00
40.38           H
ATOM     38  HB2 CYS     3       19.894   2.057  15.658  1.00
98.45           H
ATOM     39  N   SER     4       18.530  -0.434  12.557  1.00
85.31           N
ATOM     40  CA  SER     4       17.630  -0.316  11.416  1.00
92.94           C
ATOM     41  C   SER     4       16.397   0.478  11.800  1.00
67.37           C
ATOM     42  O   SER     4       15.923   1.328  11.047
1.00102.28           O
ATOM     43  CB  SER     4       17.175  -1.702  10.968
1.00123.83           C
ATOM     44  OG  SER     4       16.394  -1.579   9.787
1.00178.89           O
ATOM     45  HN  SER     4       18.748  -1.323  12.907
1.00163.03           H
ATOM     46  HA  SER     4       18.138   0.174  10.600
1.00129.78           H
ATOM     47  HB1 SER     4       16.577  -2.146  11.758
1.00105.26           H
ATOM     48  HB2 SER     4       18.032  -2.324  10.770
1.00156.87           H
ATOM     49  HG  SER     4       15.572  -2.056   9.924
1.00218.16           H
ATOM     50  N   GLU     5       15.867   0.165  12.976  1.00
41.67           N
ATOM     51  CA  GLU     5       14.663   0.816  13.469  1.00
41.21           C
ATOM     52  C   GLU     5       14.659   0.864  14.993  1.00
41.92           C
ATOM     53  O   GLU     5       15.521   0.278  15.646  1.00
74.72           O
ATOM     54  CB  GLU     5       13.442   0.051  12.970  1.00
49.24           C
ATOM     55  CG  GLU     5       13.537  -1.396  13.435  1.00
55.48           C
ATOM     56  CD  GLU     5       12.461  -2.238  12.758  1.00
92.06           C
ATOM     57  OE1 GLU     5       12.419  -3.430  13.018
1.00206.49           O
ATOM     58  OE2 GLU     5       11.695  -1.679  11.991
1.00197.24           O1-
ATOM     59  HN  GLU     5       16.285  -0.538  13.516  1.00
```

```
45.52           H
ATOM     60  HA  GLU     5      14.620   1.817  13.086  1.00
60.63           H
ATOM     61  HB1 GLU     5      13.411   0.078  11.891  1.00
61.59           H
ATOM     62  HB2 GLU     5      12.551   0.498  13.368  1.00
63.66           H
ATOM     63  HG1 GLU     5      13.405  -1.438  14.505  1.00
58.03           H
ATOM     64  HG2 GLU     5      14.511  -1.778  13.179  1.00
57.77           H
ATOM     65  N   ASN     6      13.681   1.570  15.550  1.00
50.73           N
ATOM     66  CA  ASN     6      13.569   1.693  16.998  1.00
71.04           C
ATOM     67  C   ASN     6      13.259   0.337  17.625  1.00
65.17           C
ATOM     68  O   ASN     6      13.388   0.156  18.835  1.00
94.86           O
ATOM     69  CB  ASN     6      12.457   2.685  17.346  1.00
94.83           C
ATOM     70  CG  ASN     6      12.900   4.105  17.005
1.00160.94           C
ATOM     71  ND2 ASN     6      12.003   5.038  16.841
1.00244.88           N
ATOM     72  OD1 ASN     6      14.097   4.370  16.883
1.00219.73           O
ATOM     73  HN  ASN     6      13.024   2.015  14.976  1.00
72.81           H
ATOM     74  HA  ASN     6      14.502   2.061  17.394  1.00
96.02           H
ATOM     75  HB1 ASN     6      12.239   2.625  18.402
1.00111.09           H
ATOM     76  HB2 ASN     6      11.570   2.442  16.781  1.00
98.13           H
ATOM     77 HD21 ASN     6      11.052   4.825  16.937
1.00272.43           H
ATOM     78 HD22 ASN     6      12.281   5.952  16.621
1.00336.78           H
ATOM     79  N   GLY     7      12.848  -0.609  16.790  1.00
51.80           N
ATOM     80  CA  GLY     7      12.518  -1.948  17.262  1.00
71.47           C
ATOM     81  C   GLY     7      13.761  -2.694  17.738  1.00
41.04           C
ATOM     82  O   GLY     7      13.661  -3.771  18.325  1.00
44.54           O
ATOM     83  HN  GLY     7      12.764  -0.403  15.835  1.00
45.49           H
ATOM     84  HA1 GLY     7      12.063  -2.505  16.455
1.00103.52           H
ATOM     85  HA2 GLY     7      11.817  -1.872  18.078
```

```
1.00108.67          H
ATOM     86  N   ASP     8      14.936  -2.121  17.480  1.00
27.00           N
ATOM     87  CA  ASP     8      16.183  -2.756  17.891  1.00
14.15           C
ATOM     88  C   ASP     8      16.103  -3.197  19.348  1.00
9.37            C
ATOM     89  O   ASP     8      15.917  -4.379  19.636  1.00
14.61           O
ATOM     90  CB  ASP     8      17.351  -1.786  17.706  1.00
15.06           C
ATOM     91  CG  ASP     8      18.639  -2.416  18.224  1.00
21.22           C
ATOM     92  OD1 ASP     8      19.104  -3.358  17.603
1.00119.56          O
ATOM     93  OD2 ASP     8      19.141  -1.947  19.231
1.00133.27          O1-
ATOM     94  HN  ASP     8      14.961  -1.263  17.006  1.00
35.63           H
ATOM     95  HA  ASP     8      16.351  -3.623  17.277  1.00
20.92           H
ATOM     96  HB1 ASP     8      17.151  -0.878  18.254  1.00
29.39           H
ATOM     97  HB2 ASP     8      17.460  -1.555  16.655  1.00
44.14           H
ATOM     98  N   CYS     9      16.227  -2.244  20.262  1.00
5.93            N
ATOM     99  CA  CYS     9      16.148  -2.562  21.684  1.00
7.54            C
ATOM    100  C   CYS     9      14.770  -3.122  22.013  1.00
14.60           C
ATOM    101  O   CYS     9      14.639  -4.111  22.736  1.00
25.78           O
ATOM    102  CB  CYS     9      16.407  -1.318  22.523  1.00
6.63            C
ATOM    103  SG  CYS     9      18.160  -0.910  22.419  1.00
10.91           S
ATOM    104  HN  CYS     9      16.363  -1.319  19.976  1.00
6.51            H
ATOM    105  HA  CYS     9      16.901  -3.295  21.922  1.00
11.12           H
ATOM    106  HB1 CYS     9      16.142  -1.511  23.551  1.00
11.28           H
ATOM    107  HB2 CYS     9      15.820  -0.494  22.145  1.00
5.04            H
ATOM    108  N   ALA    10      13.750  -2.478  21.460  1.00
16.26           N
ATOM    109  CA  ALA    10      12.369  -2.897  21.669  1.00
32.04           C
ATOM    110  C   ALA    10      11.434  -2.055  20.801  1.00
45.47           C
ATOM    111  O   ALA    10      11.835  -1.019  20.272
```

```
1.00119.04           O
ATOM    112  CB  ALA    10      11.985  -2.756  23.146  1.00
30.10          C
ATOM    113  HN  ALA    10      13.932  -1.703  20.890  1.00
12.81          H
ATOM    114  HA  ALA    10      12.273  -3.934  21.380  1.00
47.14          H
ATOM    115  HB1 ALA    10      11.167  -3.423  23.372  1.00
84.37          H
ATOM    116  HB2 ALA    10      11.683  -1.739  23.347
1.00118.20           H
ATOM    117  HB3 ALA    10      12.834  -3.006  23.764
1.00117.32           H
ATOM    118  N   ALA    11      10.193  -2.505  20.655  1.00
30.62          N
ATOM    119  CA  ALA    11       9.223  -1.779  19.844  1.00
35.24          C
ATOM    120  C   ALA    11       8.977  -0.378  20.404  1.00
22.78          C
ATOM    121  O   ALA    11       8.872   0.589  19.650  1.00
53.33          O
ATOM    122  CB  ALA    11       7.902  -2.549  19.802  1.00
60.49          C
ATOM    123  HN  ALA    11       9.927  -3.338  21.097  1.00
50.37          H
ATOM    124  HA  ALA    11       9.605  -1.691  18.838  1.00
41.70          H
ATOM    125  HB1 ALA    11       8.091  -3.572  19.508
1.00157.06           H
ATOM    126  HB2 ALA    11       7.238  -2.086  19.086
1.00148.83           H
ATOM    127  HB3 ALA    11       7.444  -2.534  20.779
1.00137.06           H
ATOM    128  N   ASP    12       8.877  -0.278  21.726  1.00
18.98          N
ATOM    129  CA  ASP    12       8.633   1.011  22.374  1.00
31.05          C
ATOM    130  C   ASP    12       9.933   1.790  22.572  1.00
22.98          C
ATOM    131  O   ASP    12       9.936   3.020  22.555  1.00
37.14          O
ATOM    132  CB  ASP    12       7.964   0.790  23.733  1.00
48.35          C
ATOM    133  CG  ASP    12       6.546   0.264  23.535
1.00104.83           C
ATOM    134  OD1 ASP    12       5.978  -0.225  24.498
1.00258.73           O
ATOM    135  OD2 ASP    12       6.049   0.358  22.425
1.00213.46           O1-
ATOM    136  HN  ASP    12       8.963  -1.083  22.279  1.00
36.19          H
ATOM    137  HA  ASP    12       7.968   1.596  21.755  1.00
```

49.76 H
ATOM 138 HB1 ASP 12 7.926 1.726 24.270 1.00
95.71 H
ATOM 139 HB2 ASP 12 8.537 0.072 24.301 1.00
54.37 H
ATOM 140 N GLU 13 11.031 1.068 22.768 1.00
17.60 N
ATOM 141 CA GLU 13 12.327 1.705 22.983 1.00
12.01 C
ATOM 142 C GLU 13 12.895 2.264 21.680 1.00
10.07 C
ATOM 143 O GLU 13 12.538 1.815 20.590 1.00
12.81 O
ATOM 144 CB GLU 13 13.306 0.699 23.586 1.00
11.74 C
ATOM 145 CG GLU 13 12.810 0.277 24.972 1.00
13.49 C
ATOM 146 CD GLU 13 12.916 1.447 25.942
1.00142.36 C
ATOM 147 OE1 GLU 13 12.296 1.380 26.992
1.00339.06 O
ATOM 148 OE2 GLU 13 13.607 2.396 25.620
1.00335.28 O1-
ATOM 149 HN GLU 13 10.967 0.090 22.781 1.00
28.36 H
ATOM 150 HA GLU 13 12.199 2.520 23.678 1.00
13.06 H
ATOM 151 HB1 GLU 13 14.280 1.155 23.676 1.00
13.76 H
ATOM 152 HB2 GLU 13 13.372 -0.167 22.945 1.00
9.94 H
ATOM 153 HG1 GLU 13 13.411 -0.543 25.334 1.00
66.02 H
ATOM 154 HG2 GLU 13 11.779 -0.034 24.904 1.00
56.23 H
ATOM 155 N CYS 14 13.784 3.254 21.807 1.00
9.12 N
ATOM 156 CA CYS 14 14.412 3.894 20.645 1.00
10.69 C
ATOM 157 C CYS 14 15.880 3.497 20.549 1.00
9.56 C
ATOM 158 O CYS 14 16.489 3.106 21.542 1.00
13.62 O
ATOM 159 CB CYS 14 14.305 5.416 20.775 1.00
13.96 C
ATOM 160 SG CYS 14 15.334 6.209 19.515 1.00
42.96 S
ATOM 161 HN CYS 14 14.024 3.563 22.708 1.00
9.76 H
ATOM 162 HA CYS 14 13.905 3.585 19.741 1.00
15.54 H
ATOM 163 HB1 CYS 14 14.640 5.714 21.757 1.00

```
50.72           H
ATOM    164  HB2 CYS    14       13.275   5.714  20.640  1.00
46.13           H
ATOM    165  N   CYS    15       16.448   3.610  19.347  1.00
9.60           N
ATOM    166  CA  CYS    15       17.851   3.269  19.130  1.00
9.28           C
ATOM    167  C   CYS    15       18.447   4.185  18.076  1.00
9.92           C
ATOM    168  O   CYS    15       17.844   4.404  17.025  1.00
13.31           O
ATOM    169  CB  CYS    15       17.988   1.822  18.654  1.00
12.90           C
ATOM    170  SG  CYS    15       19.747   1.389  18.590  1.00
39.11           S
ATOM    171  HN  CYS    15       15.915   3.939  18.592  1.00
13.12           H
ATOM    172  HA  CYS    15       18.402   3.388  20.053  1.00
8.26           H
ATOM    173  HB1 CYS    15       17.558   1.726  17.667  1.00
24.82           H
ATOM    174  HB2 CYS    15       17.473   1.165  19.335  1.00
27.31           H
ATOM    175  N   VAL    16       19.631   4.718  18.350  1.00
9.56           N
ATOM    176  CA  VAL    16       20.283   5.602  17.398  1.00
11.99           C
ATOM    177  C   VAL    16       21.791   5.563  17.605  1.00
7.73           C
ATOM    178  O   VAL    16       22.293   5.911  18.673  1.00
8.83           O
ATOM    179  CB  VAL    16       19.745   7.026  17.568  1.00
18.37           C
ATOM    180  CG1 VAL    16       20.073   7.555  18.969  1.00
39.61           C
ATOM    181  CG2 VAL    16       20.370   7.933  16.509
1.00115.21           C
ATOM    182  HN  VAL    16       20.077   4.509  19.203  1.00
9.76           H
ATOM    183  HA  VAL    16       20.065   5.266  16.394  1.00
17.27           H
ATOM    184  HB  VAL    16       18.672   7.015  17.438  1.00
52.63           H
ATOM    185 HG11 VAL    16       19.923   6.770  19.694
1.00128.07           H
ATOM    186 HG12 VAL    16       19.422   8.385  19.200
1.00154.13           H
ATOM    187 HG13 VAL    16       21.100   7.887  19.002
1.00135.74           H
ATOM    188 HG21 VAL    16       20.157   7.535  15.529
1.00229.05           H
ATOM    189 HG22 VAL    16       21.438   7.976  16.658
```

```
1.00261.82          H
ATOM    190 HG23 VAL    16      19.953   8.924  16.595
1.00210.07          H
ATOM    191  N   ASP    17      22.510   5.114  16.579  1.00
14.34           N
ATOM    192  CA  ASP    17      23.965   5.010  16.660  1.00
11.90           C
ATOM    193  C   ASP    17      24.637   6.244  16.077  1.00
13.15           C
ATOM    194  O   ASP    17      24.497   6.542  14.892  1.00
25.96           O
ATOM    195  CB  ASP    17      24.440   3.770  15.902  1.00
20.80           C
ATOM    196  CG  ASP    17      24.021   2.511  16.654  1.00
28.72           C
ATOM    197  OD1 ASP    17      23.649   2.632  17.810
1.00135.42          O
ATOM    198  OD2 ASP    17      24.077   1.446  16.064
1.00114.17          O1-
ATOM    199  HN  ASP    17      22.053   4.832  15.757  1.00
28.29           H
ATOM    200  HA  ASP    17      24.259   4.912  17.695  1.00
10.49           H
ATOM    201  HB1 ASP    17      25.516   3.793  15.817  1.00
22.42           H
ATOM    202  HB2 ASP    17      24.002   3.764  14.915  1.00
29.52           H
ATOM    203  N   THR    18      25.387   6.946  16.921  1.00
11.55           N
ATOM    204  CA  THR    18      26.115   8.140  16.497  1.00
17.10           C
ATOM    205  C   THR    18      27.590   7.807  16.338  1.00
10.63           C
ATOM    206  O   THR    18      28.057   6.787  16.843  1.00
6.49           O
ATOM    207  CB  THR    18      25.949   9.256  17.531  1.00
28.52           C
ATOM    208  CG2 THR    18      24.536   9.832  17.448  1.00
45.38           C
ATOM    209  OG1 THR    18      26.177   8.733  18.830  1.00
26.49           O
ATOM    210  HN  THR    18      25.470   6.643  17.849  1.00
13.96           H
ATOM    211  HA  THR    18      25.728   8.484  15.547  1.00
26.43           H
ATOM    212  HB  THR    18      26.663  10.039  17.331  1.00
37.43           H
ATOM    213  HG1 THR    18      26.568   7.861  18.734  1.00
71.62           H
ATOM    214 HG21 THR    18      24.441  10.647  18.150
1.00100.44          H
ATOM    215 HG22 THR    18      23.819   9.063  17.689
```

```
1.00129.42           H
ATOM    216 HG23 THR    18      24.356  10.196  16.448
1.00156.21           H
ATOM    217  N   VAL    19      28.318   8.673  15.646  1.00
16.32           N
ATOM    218  CA  VAL    19      29.731   8.473  15.433  1.00
13.34           C
ATOM    219  C   VAL    19      30.288   9.631  14.634  1.00
25.88           C
ATOM    220  O   VAL    19      29.903   9.877  13.490  1.00
42.11           O
ATOM    221  CB  VAL    19      30.012   7.157  14.708  1.00
15.48           C
ATOM    222  CG1 VAL    19      29.152   7.053  13.443  1.00
29.06           C
ATOM    223  CG2 VAL    19      31.498   7.109  14.327  1.00
20.67           C
ATOM    224  HN  VAL    19      27.904   9.476  15.283  1.00
26.89           H
ATOM    225  HA  VAL    19      30.222   8.448  16.395  1.00
8.08           H
ATOM    226  HB  VAL    19      29.783   6.334  15.365  1.00
11.49           H
ATOM    227 HG11 VAL    19      29.163   6.033  13.085
1.00137.43           H
ATOM    228 HG12 VAL    19      29.550   7.704  12.680  1.00
93.29           H
ATOM    229 HG13 VAL    19      28.136   7.341  13.670
1.00108.69           H
ATOM    230 HG21 VAL    19      31.776   6.096  14.079  1.00
99.42           H
ATOM    231 HG22 VAL    19      32.094   7.455  15.162
1.00111.36           H
ATOM    232 HG23 VAL    19      31.670   7.752  13.476  1.00
77.11           H
ATOM    233  N   PHE    20      31.188  10.338  15.268  1.00
24.58           N
ATOM    234  CA  PHE    20      31.831  11.499  14.677  1.00
40.34           C
ATOM    235  C   PHE    20      33.222  11.153  14.164  1.00
40.37           C
ATOM    236  O   PHE    20      33.494  11.259  12.968  1.00
66.02           O
ATOM    237  CB  PHE    20      31.924  12.615  15.730  1.00
48.79           C
ATOM    238  CG  PHE    20      32.137  12.031  17.118  1.00
35.75           C
ATOM    239  CD1 PHE    20      31.117  11.294  17.749  1.00
30.20           C
ATOM    240  CD2 PHE    20      33.350  12.245  17.784  1.00
40.95           C
ATOM    241  CE1 PHE    20      31.321  10.773  19.031  1.00
```

```
30.41           C
ATOM    242  CE2 PHE    20       33.549  11.722  19.067  1.00
45.84           C
ATOM    243  CZ  PHE    20       32.536  10.988  19.690  1.00
40.81           C
ATOM    244  HN  PHE    20       31.412  10.076  16.176  1.00
16.73           H
ATOM    245  HA  PHE    20       31.236  11.857  13.846  1.00
59.91           H
ATOM    246  HB1 PHE    20       31.016  13.168  15.724  1.00
67.15           H
ATOM    247  HB2 PHE    20       32.740  13.281  15.488  1.00
58.17           H
ATOM    248  HD1 PHE    20       30.175  11.119  17.248  1.00
33.34           H
ATOM    249  HD2 PHE    20       34.136  12.812  17.306  1.00
49.35           H
ATOM    250  HE1 PHE    20       30.538  10.207  19.511  1.00
30.61           H
ATOM    251  HE2 PHE    20       34.486  11.887  19.577  1.00
61.27           H
ATOM    252  HZ  PHE    20       32.691  10.585  20.680  1.00
52.66           H
ATOM    253  N   GLU    21       34.109  10.779  15.078  1.00
41.31           N
ATOM    254  CA  GLU    21       35.476  10.465  14.726  1.00
56.16           C
ATOM    255  C   GLU    21       35.751   8.970  14.801  1.00
58.61           C
ATOM    256  O   GLU    21       34.858   8.168  15.072
1.00201.19           O
ATOM    257  CB  GLU    21       36.369  11.212  15.692  1.00
57.37           C
ATOM    258  CG  GLU    21       36.236  10.620  17.097
1.00193.56           C
ATOM    259  CD  GLU    21       36.899  11.544  18.113
1.00304.62           C
ATOM    260  OE1 GLU    21       36.744  11.300  19.297
1.00451.81           O
ATOM    261  OE2 GLU    21       37.555  12.482  17.691
1.00442.88           O1-
ATOM    262  HN  GLU    21       33.851  10.744  16.019  1.00
50.82           H
ATOM    263  HA  GLU    21       35.688  10.812  13.726  1.00
85.72           H
ATOM    264  HB1 GLU    21       36.063  12.243  15.712  1.00
46.37           H
ATOM    265  HB2 GLU    21       37.385  11.142  15.365
1.00131.81           H
ATOM    266  HG1 GLU    21       36.713   9.657  17.125
1.00314.91           H
ATOM    267  HG2 GLU    21       35.192  10.505  17.340
```

```
1.00266.92           H
ATOM    268  N   GLY    22       37.003   8.617  14.546  1.00
86.70        N
ATOM    269  CA  GLY    22       37.429   7.214  14.569
1.00104.66           C
ATOM    270  C   GLY    22       37.995   6.828  15.931  1.00
71.50        C
ATOM    271  O   GLY    22       38.206   5.650  16.215  1.00
89.87        O
ATOM    272  HN  GLY    22       37.657   9.318  14.338
1.00214.64           H
ATOM    273  HA1 GLY    22       38.191   7.066  13.819
1.00152.47           H
ATOM    274  HA2 GLY    22       36.583   6.577  14.342
1.00118.15           H
ATOM    275  N   ASP    23       38.244   7.830  16.765  1.00
44.68        N
ATOM    276  CA  ASP    23       38.791   7.590  18.095  1.00
43.33        C
ATOM    277  C   ASP    23       37.856   6.712  18.917  1.00
37.55        C
ATOM    278  O   ASP    23       38.295   5.800  19.615  1.00
61.37        O
ATOM    279  CB  ASP    23       39.002   8.921  18.816  1.00
40.74        C
ATOM    280  CG  ASP    23       39.727   8.690  20.137
1.00141.86           C
ATOM    281  OD1 ASP    23       40.139   9.666  20.743
1.00328.18           O
ATOM    282  OD2 ASP    23       39.860   7.540  20.525
1.00304.58           O1-
ATOM    283  HN  ASP    23       38.062   8.747  16.478  1.00
40.86        H
ATOM    284  HA  ASP    23       39.741   7.092  17.997  1.00
72.12        H
ATOM    285  HB1 ASP    23       38.042   9.373  19.011  1.00
94.15        H
ATOM    286  HB2 ASP    23       39.588   9.578  18.191  1.00
93.26        H
ATOM    287  N   MET    24       36.563   7.001  18.832  1.00
23.24        N
ATOM    288  CA  MET    24       35.565   6.238  19.576  1.00
32.50        C
ATOM    289  C   MET    24       34.197   6.343  18.910  1.00
25.13        C
ATOM    290  O   MET    24       33.982   7.186  18.039  1.00
54.40        O
ATOM    291  CB  MET    24       35.476   6.760  21.013  1.00
47.36        C
ATOM    292  CG  MET    24       34.978   8.208  21.006
1.00151.73           C
ATOM    293  SD  MET    24       35.116   8.908  22.670
```

```
1.00209.23            S
ATOM    294  CE  MET    24       36.855   9.411  22.584
1.00243.29            C
ATOM    295  HN  MET    24       36.276   7.743  18.262  1.00
17.32          H
ATOM    296  HA  MET    24       35.860   5.200  19.603  1.00
48.94          H
ATOM    297  HB1 MET    24       36.452   6.718  21.472
1.00124.40            H
ATOM    298  HB2 MET    24       34.787   6.144  21.576
1.00166.96            H
ATOM    299  HG1 MET    24       33.945   8.230  20.691
1.00331.71            H
ATOM    300  HG2 MET    24       35.574   8.789  20.319
1.00302.30            H
ATOM    301  HE1 MET    24       37.366   8.831  21.828
1.00340.44            H
ATOM    302  HE2 MET    24       37.326   9.243  23.539
1.00373.88            H
ATOM    303  HE3 MET    24       36.913  10.462  22.339
1.00386.81            H
ATOM    304  N   VAL    25       33.272   5.477  19.329  1.00
22.44          N
ATOM    305  CA  VAL    25       31.915   5.464  18.775  1.00
15.37          C
ATOM    306  C   VAL    25       30.879   5.620  19.883  1.00
17.47          C
ATOM    307  O   VAL    25       31.025   5.064  20.973  1.00
29.52          O
ATOM    308  CB  VAL    25       31.664   4.154  18.021  1.00
23.36          C
ATOM    309  CG1 VAL    25       30.193   4.083  17.579  1.00
58.39          C
ATOM    310  CG2 VAL    25       32.576   4.100  16.790  1.00
55.25          C
ATOM    311  HN  VAL    25       33.508   4.831  20.027  1.00
48.11          H
ATOM    312  HA  VAL    25       31.799   6.291  18.081  1.00
9.25          H
ATOM    313  HB  VAL    25       31.884   3.319  18.671  1.00
52.45          H
ATOM    314 HG11 VAL    25       29.854   5.067  17.286
1.00171.07            H
ATOM    315 HG12 VAL    25       29.590   3.731  18.401
1.00166.22            H
ATOM    316 HG13 VAL    25       30.094   3.404  16.744
1.00134.76            H
ATOM    317 HG21 VAL    25       33.600   3.972  17.107
1.00141.88            H
ATOM    318 HG22 VAL    25       32.484   5.021  16.233
1.00184.50            H
ATOM    319 HG23 VAL    25       32.287   3.270  16.164
```

```
1.00133.90          H
ATOM    320  N   THR    26       29.838   6.396  19.593  1.00
13.60          N
ATOM    321  CA  THR    26       28.766   6.655  20.554  1.00
21.19          C
ATOM    322  C   THR    26       27.464   5.985  20.120  1.00
16.55          C
ATOM    323  O   THR    26       27.040   6.111  18.971  1.00
11.43          O
ATOM    324  CB  THR    26       28.553   8.163  20.658  1.00
27.07          C
ATOM    325  CG2 THR    26       27.372   8.460  21.584  1.00
44.39          C
ATOM    326  OG1 THR    26       29.730   8.768  21.176  1.00
34.75          O
ATOM    327  HN  THR    26       29.793   6.815  18.708  1.00
10.81          H
ATOM    328  HA  THR    26       29.049   6.277  21.527  1.00
33.16          H
ATOM    329  HB  THR    26       28.348   8.560  19.674  1.00
20.60          H
ATOM    330  HG1 THR    26       29.722   8.660  22.130  1.00
77.72          H
ATOM    331 HG21 THR    26       27.349   9.515  21.811
1.00126.99          H
ATOM    332 HG22 THR    26       27.481   7.896  22.499
1.00108.32          H
ATOM    333 HG23 THR    26       26.450   8.178  21.097
1.00151.99          H
ATOM    334  N   ARG    27       26.830   5.274  21.056  1.00
22.38          N
ATOM    335  CA  ARG    27       25.567   4.581  20.785  1.00
20.21          C
ATOM    336  C   ARG    27       24.560   4.878  21.894  1.00
16.96          C
ATOM    337  O   ARG    27       24.942   5.057  23.050  1.00
20.06          O
ATOM    338  CB  ARG    27       25.808   3.071  20.715  1.00
22.14          C
ATOM    339  CG  ARG    27       26.780   2.759  19.575
1.00124.30          C
ATOM    340  CD  ARG    27       27.093   1.261  19.571
1.00109.38          C
ATOM    341  NE  ARG    27       25.893   0.495  19.254
1.00227.73          N
ATOM    342  CZ  ARG    27       25.505   0.311  17.996
1.00426.12          C
ATOM    343  NH1 ARG    27       24.423  -0.371  17.739
1.00767.09          N1+
ATOM    344  NH2 ARG    27       26.208   0.812  17.017
1.00581.78          N
ATOM    345  HN  ARG    27       27.220   5.217  21.954  1.00
```

```
30.75           H
ATOM    346  HA  ARG    27       25.161   4.917  19.842  1.00
23.13           H
ATOM    347  HB1 ARG    27       24.872   2.565  20.536  1.00
87.46           H
ATOM    348  HB2 ARG    27       26.228   2.730  21.650
1.00103.36           H
ATOM    349  HG1 ARG    27       27.693   3.316  19.718
1.00281.97           H
ATOM    350  HG2 ARG    27       26.330   3.036  18.633
1.00276.19           H
ATOM    351  HD1 ARG    27       27.458   0.971  20.547
1.00183.60           H
ATOM    352  HD2 ARG    27       27.854   1.058  18.833
1.00142.93           H
ATOM    353  HE  ARG    27       25.361   0.109  19.981
1.00372.53           H
ATOM    354 HH11 ARG    27       23.886  -0.757  18.488
1.00910.59           H
ATOM    355 HH12 ARG    27       24.131  -0.510  16.792
1.00999.99           H
ATOM    356 HH21 ARG    27       27.040   1.333  17.213
1.00532.54           H
ATOM    357 HH22 ARG    27       25.917   0.674  16.070
1.00948.84           H
ATOM    358  N   SER    28       23.270   4.935  21.544  1.00
14.60           N
ATOM    359  CA  SER    28       22.230   5.216  22.538  1.00
14.92           C
ATOM    360  C   SER    28       21.025   4.305  22.331  1.00
12.12           C
ATOM    361  O   SER    28       20.691   3.943  21.204  1.00
13.41           O
ATOM    362  CB  SER    28       21.796   6.678  22.435  1.00
23.26           C
ATOM    363  OG  SER    28       22.896   7.519  22.751
1.00146.32           O
ATOM    364  HN  SER    28       23.010   4.786  20.606  1.00
15.23           H
ATOM    365  HA  SER    28       22.625   5.044  23.530  1.00
15.44           H
ATOM    366  HB1 SER    28       20.980   6.860  23.123  1.00
88.73           H
ATOM    367  HB2 SER    28       21.467   6.888  21.432
1.00124.68           H
ATOM    368  HG  SER    28       22.550   8.361  23.051
1.00242.47           H
ATOM    369  N   CYS    29       20.373   3.948  23.433  1.00
10.48           N
ATOM    370  CA  CYS    29       19.197   3.089  23.380  1.00
9.97           C
ATOM    371  C   CYS    29       18.597   2.956  24.777  1.00
```

```
11.60           C
ATOM    372  O   CYS    29       19.248   2.456  25.695  1.00
15.64           O
ATOM    373  CB  CYS    29       19.564   1.701  22.819  1.00
9.52            C
ATOM    374  SG  CYS    29       18.163   1.033  21.904  1.00
10.40           S
ATOM    375  HN  CYS    29       20.683   4.282  24.301  1.00
11.10           H
ATOM    376  HA  CYS    29       18.466   3.548  22.732  1.00
11.08           H
ATOM    377  HB1 CYS    29       19.807   1.022  23.625  1.00
9.33            H
ATOM    378  HB2 CYS    29       20.410   1.786  22.158  1.00
10.78           H
ATOM    379  N   GLU    30       17.364   3.418  24.937  1.00
13.52           N
ATOM    380  CA  GLU    30       16.694   3.358  26.233  1.00
17.41           C
ATOM    381  C   GLU    30       16.055   1.994  26.460  1.00
10.81           C
ATOM    382  O   GLU    30       15.735   1.279  25.511  1.00
23.22           O
ATOM    383  CB  GLU    30       15.626   4.446  26.304  1.00
37.83           C
ATOM    384  CG  GLU    30       16.296   5.817  26.202
1.00108.13           C
ATOM    385  CD  GLU    30       17.091   6.105  27.471
1.00240.52           C
ATOM    386  OE1 GLU    30       16.873   5.415  28.451
1.00422.56           O
ATOM    387  OE2 GLU    30       17.906   7.012  27.442
1.00410.81           O1-
ATOM    388  HN  GLU    30       16.894   3.816  24.171  1.00
15.61           H
ATOM    389  HA  GLU    30       17.420   3.536  27.012  1.00
24.60           H
ATOM    390  HB1 GLU    30       15.095   4.374  27.241  1.00
72.67           H
ATOM    391  HB2 GLU    30       14.935   4.321  25.484  1.00
56.47           H
ATOM    392  HG1 GLU    30       15.541   6.578  26.071
1.00196.66           H
ATOM    393  HG2 GLU    30       16.963   5.823  25.352
1.00140.67           H
ATOM    394  N   LYS    31       15.866   1.643  27.732  1.00
11.96           N
ATOM    395  CA  LYS    31       15.254   0.365  28.101  1.00
11.40           C
ATOM    396  C   LYS    31       13.962   0.612  28.868  1.00
10.99           C
ATOM    397  O   LYS    31       13.861   1.567  29.638  1.00
```

```
12.86           O
ATOM    398  CB  LYS    31       16.218  -0.439  28.975  1.00
21.40           C
ATOM    399  CG  LYS    31       17.479  -0.775  28.175  1.00
57.81           C
ATOM    400  CD  LYS    31       18.440  -1.579  29.050
1.00115.52           C
ATOM    401  CE  LYS    31       19.702  -1.911  28.253
1.00250.23           C
ATOM    402  NZ  LYS    31       20.644  -2.685  29.111
1.00462.10           N1+
ATOM    403  HN  LYS    31       16.140   2.262  28.441  1.00
26.13           H
ATOM    404  HA  LYS    31       15.030  -0.206  27.210  1.00
13.87           H
ATOM    405  HB1 LYS    31       15.741  -1.354  29.290  1.00
37.63           H
ATOM    406  HB2 LYS    31       16.488   0.144  29.844  1.00
47.19           H
ATOM    407  HG1 LYS    31       17.960   0.139  27.859
1.00126.32           H
ATOM    408  HG2 LYS    31       17.207  -1.358  27.305
1.00114.19           H
ATOM    409  HD1 LYS    31       17.964  -2.494  29.363
1.00198.45           H
ATOM    410  HD2 LYS    31       18.707  -0.995  29.921
1.00200.14           H
ATOM    411  HE1 LYS    31       20.177  -0.995  27.933
1.00374.64           H
ATOM    412  HE2 LYS    31       19.437  -2.501  27.387
1.00403.27           H
ATOM    413  HZ1 LYS    31       20.554  -3.698  28.897
1.00627.38           H
ATOM    414  HZ2 LYS    31       20.416  -2.519  30.113
1.00622.26           H
ATOM    415  HZ3 LYS    31       21.618  -2.379  28.922
1.00619.20           H
ATOM    416  N   THR    32       12.971  -0.247  28.651  1.00
15.51           N
ATOM    417  CA  THR    32       11.687  -0.099  29.326  1.00
22.44           C
ATOM    418  C   THR    32       11.759  -0.646  30.748  1.00
26.60           C
ATOM    419  O   THR    32       12.105  -1.807  30.964  1.00
57.43           O
ATOM    420  CB  THR    32       10.597  -0.837  28.546  1.00
56.28           C
ATOM    421  CG2 THR    32        9.257  -0.685  29.270
1.00102.70           C
ATOM    422  OG1 THR    32       10.497  -0.288  27.240
1.00111.17           O
ATOM    423  HN  THR    32       13.105  -0.987  28.022  1.00
```

```
18.79            H
ATOM    424  HA  THR    32       11.432   0.950  29.369  1.00
19.67            H
ATOM    425  HB  THR    32       10.848  -1.883  28.479  1.00
84.25            H
ATOM    426  HG1 THR    32       11.076  -0.791  26.662
1.00205.03                 H
ATOM    427 HG21 THR    32        9.093   0.355  29.508
1.00220.71                 H
ATOM    428 HG22 THR    32        9.273  -1.265  30.181
1.00174.67                 H
ATOM    429 HG23 THR    32        8.461  -1.040  28.632
1.00217.44                 H
ATOM    430  N   THR    33       11.423   0.204  31.715  1.00
22.47            N
ATOM    431  CA  THR    33       11.444  -0.188  33.120  1.00
41.60            C
ATOM    432  C   THR    33       10.458   0.630  33.907  1.00
38.03            C
ATOM    433  O   THR    33       10.789   1.686  34.449  1.00
52.73            O
ATOM    434  CB  THR    33       12.848  -0.018  33.706  1.00
63.25            C
ATOM    435  CG2 THR    33       13.795  -1.050  33.091
1.00121.65                 C
ATOM    436  OG1 THR    33       13.322   1.290  33.424  1.00
96.20            O
ATOM    437  HN  THR    33       11.154   1.116  31.477  1.00
23.82            H
ATOM    438  HA  THR    33       11.145  -1.214  33.214  1.00
67.12            H
ATOM    439  HB  THR    33       12.810  -0.163  34.775
1.00107.59                 H
ATOM    440  HG1 THR    33       13.960   1.227  32.707
1.00176.27                 H
ATOM    441 HG21 THR    33       13.334  -2.026  33.121
1.00255.23                 H
ATOM    442 HG22 THR    33       14.718  -1.072  33.653
1.00245.37                 H
ATOM    443 HG23 THR    33       14.005  -0.782  32.067
1.00185.78                 H
ATOM    444  N   GLY    34        9.242   0.121  33.979  1.00
40.93            N
ATOM    445  CA  GLY    34        8.207   0.798  34.717  1.00
45.59            C
ATOM    446  C   GLY    34        7.745   2.048  33.978  1.00
40.97            C
ATOM    447  O   GLY    34        6.550   2.333  33.906  1.00
82.17            O
ATOM    448  HN  GLY    34        9.043  -0.730  33.535  1.00
54.00            H
ATOM    449  HA1 GLY    34        8.613   1.074  35.670  1.00
```

```
54.41           H
ATOM    450  HA2 GLY    34        7.370   0.129  34.861  1.00
62.09           H
ATOM    451  N   ASN    35        8.704   2.785  33.421  1.00
32.51           N
ATOM    452  CA  ASN    35        8.405   4.007  32.670  1.00
33.20           C
ATOM    453  C   ASN    35        9.197   4.028  31.368  1.00
23.17           C
ATOM    454  O   ASN    35       10.364   3.638  31.335  1.00
27.01           O
ATOM    455  CB  ASN    35        8.763   5.236  33.504  1.00
54.51           C
ATOM    456  CG  ASN    35        7.955   5.243  34.797  1.00
80.73           C
ATOM    457  ND2 ASN    35        8.543   5.551  35.921
1.00220.46           N
ATOM    458  OD1 ASN    35        6.757   4.959  34.783
1.00117.80           O
ATOM    459  HN  ASN    35        9.638   2.498  33.509  1.00
51.15           H
ATOM    460  HA  ASN    35        7.350   4.038  32.435  1.00
47.21           H
ATOM    461  HB1 ASN    35        8.539   6.128  32.939  1.00
68.31           H
ATOM    462  HB2 ASN    35        9.816   5.215  33.738  1.00
57.75           H
ATOM    463 HD21 ASN    35        9.495   5.774  35.930
1.00405.39           H
ATOM    464 HD22 ASN    35        8.030   5.556  36.757
1.00245.64           H
ATOM    465  N   PHE    36        8.555   4.477  30.295  1.00
35.72           N
ATOM    466  CA  PHE    36        9.199   4.537  28.993  1.00
33.41           C
ATOM    467  C   PHE    36       10.018   5.818  28.850  1.00
28.51           C
ATOM    468  O   PHE    36        9.662   6.860  29.400  1.00
45.23           O
ATOM    469  CB  PHE    36        8.129   4.485  27.907  1.00
67.53           C
ATOM    470  CG  PHE    36        7.459   3.132  27.935  1.00
86.71           C
ATOM    471  CD1 PHE    36        6.344   2.925  28.757
1.00106.51           C
ATOM    472  CD2 PHE    36        7.947   2.087  27.142  1.00
99.48           C
ATOM    473  CE1 PHE    36        5.719   1.672  28.785
1.00134.48           C
ATOM    474  CE2 PHE    36        7.322   0.835  27.171
1.00134.27           C
ATOM    475  CZ  PHE    36        6.208   0.627  27.992
```

```
1.00149.48           C
ATOM    476  HN  PHE    36       7.625   4.768  30.376  1.00
62.87           H
ATOM    477  HA  PHE    36       9.853   3.685  28.879  1.00
28.53           H
ATOM    478  HB1 PHE    36       8.586   4.639  26.948  1.00
73.56           H
ATOM    479  HB2 PHE    36       7.395   5.256  28.087  1.00
88.42           H
ATOM    480  HD1 PHE    36       5.967   3.731  29.371
1.00110.20           H
ATOM    481  HD2 PHE    36       8.809   2.247  26.510  1.00
93.07           H
ATOM    482  HE1 PHE    36       4.859   1.512  29.420
1.00155.06           H
ATOM    483  HE2 PHE    36       7.699   0.029  26.559
1.00159.46           H
ATOM    484  HZ  PHE    36       5.726  -0.338  28.015
1.00183.25           H
ATOM    485  N   THR    37      11.117   5.729  28.103  1.00
20.06           N
ATOM    486  CA  THR    37      11.989   6.877  27.878  1.00
27.18           C
ATOM    487  C   THR    37      12.604   6.796  26.486  1.00
41.87           C
ATOM    488  O   THR    37      13.304   5.841  26.161
1.00169.86           O
ATOM    489  CB  THR    37      13.101   6.904  28.929  1.00
26.52           C
ATOM    490  CG2 THR    37      12.485   7.026  30.324  1.00
75.69           C
ATOM    491  OG1 THR    37      13.857   5.703  28.847  1.00
81.95           O
ATOM    492  HN  THR    37      11.344   4.875  27.686  1.00
18.47           H
ATOM    493  HA  THR    37      11.412   7.788  27.955  1.00
38.80           H
ATOM    494  HB  THR    37      13.746   7.749  28.752  1.00
81.60           H
ATOM    495  HG1 THR    37      14.764   5.939  28.637
1.00188.80           H
ATOM    496 HG21 THR    37      12.047   6.079  30.609
1.00196.98           H
ATOM    497 HG22 THR    37      11.719   7.786  30.315
1.00196.23           H
ATOM    498 HG23 THR    37      13.251   7.295  31.035
1.00168.92           H
ATOM    499  N   GLU    38      12.326   7.801  25.670  1.00
26.53           N
ATOM    500  CA  GLU    38      12.846   7.837  24.305  1.00
30.29           C
ATOM    501  C   GLU    38      14.336   8.173  24.292  1.00
```

```
25.39           C
ATOM    502  O   GLU    38       14.860   8.759  25.239  1.00
51.60           O
ATOM    503  CB  GLU    38       12.080   8.877  23.484  1.00
54.82           C
ATOM    504  CG  GLU    38       10.635   8.409  23.290
1.00165.84           C
ATOM    505  CD  GLU    38        9.837   9.475  22.548
1.00292.92           C
ATOM    506  OE1 GLU    38       10.361  10.561  22.367
1.00414.19           O
ATOM    507  OE2 GLU    38        8.712   9.191  22.173
1.00537.65           O1-
ATOM    508  HN  GLU    38       11.754   8.528  25.986  1.00
84.50           H
ATOM    509  HA  GLU    38       12.704   6.867  23.853  1.00
32.15           H
ATOM    510  HB1 GLU    38       12.551   8.993  22.520
1.00144.82           H
ATOM    511  HB2 GLU    38       12.086   9.823  24.006
1.00106.49           H
ATOM    512  HG1 GLU    38       10.184   8.230  24.253
1.00303.31           H
ATOM    513  HG2 GLU    38       10.630   7.494  22.716
1.00323.99           H
ATOM    514  N   CYS    39       15.008   7.801  23.205  1.00
15.84           N
ATOM    515  CA  CYS    39       16.436   8.067  23.062  1.00
14.30           C
ATOM    516  C   CYS    39       16.686   9.579  23.002  1.00
22.31           C
ATOM    517  O   CYS    39       15.830  10.325  22.526  1.00
32.92           O
ATOM    518  CB  CYS    39       16.953   7.403  21.782  1.00
18.08           C
ATOM    519  SG  CYS    39       15.942   7.935  20.377  1.00
17.41           S
ATOM    520  HN  CYS    39       14.533   7.343  22.482  1.00
24.87           H
ATOM    521  HA  CYS    39       16.954   7.645  23.908  1.00
14.60           H
ATOM    522  HB1 CYS    39       16.893   6.330  21.883  1.00
27.71           H
ATOM    523  HB2 CYS    39       17.979   7.691  21.614  1.00
34.91           H
ATOM    524  N   PRO    40       17.822  10.052  23.467  1.00
27.27           N
ATOM    525  CA  PRO    40       18.142  11.509  23.446  1.00
47.66           C
ATOM    526  C   PRO    40       18.523  11.992  22.049  1.00
71.54           C
ATOM    527  O   PRO    40       19.250  11.315  21.321  1.00
```

```
87.70           O
ATOM    528  CB  PRO    40      19.320  11.625  24.418  1.00
53.85           C
ATOM    529  CG  PRO    40      20.022  10.312  24.308  1.00
46.04           C
ATOM    530  CD  PRO    40      18.926   9.270  24.055  1.00
25.70           C
ATOM    531  HA  PRO    40      17.306  12.078  23.821  1.00
58.04           H
ATOM    532  HB1 PRO    40      18.962  11.769  25.429  1.00
67.93           H
ATOM    533  HB2 PRO    40      19.976  12.438  24.131  1.00
70.99           H
ATOM    534  HG1 PRO    40      20.542  10.083  25.227  1.00
65.91           H
ATOM    535  HG2 PRO    40      20.721  10.335  23.479  1.00
54.99           H
ATOM    536  HD1 PRO    40      18.612   8.817  24.981  1.00
24.22           H
ATOM    537  HD2 PRO    40      19.273   8.521  23.359  1.00
26.94           H
ATOM    538  N   GLY    41      18.034  13.169  21.691
1.00105.24           N
ATOM    539  CA  GLY    41      18.329  13.748  20.386
1.00147.55           C
ATOM    540  C   GLY    41      19.821  14.022  20.242
1.00161.74           C
ATOM    541  O   GLY    41      20.408  13.802  19.183
1.00244.37           O
ATOM    542  HN  GLY    41      17.467  13.660  22.319
1.00121.79           H
ATOM    543  HA1 GLY    41      17.789  14.676  20.277
1.00184.40           H
ATOM    544  HA2 GLY    41      18.020  13.061  19.615
1.00157.34           H
ATOM    545  N   LEU    42      20.429  14.507  21.324
1.00162.48           N
ATOM    546  CA  LEU    42      21.860  14.821  21.336
1.00191.48           C
ATOM    547  C   LEU    42      22.600  13.875  22.275
1.00166.66           C
ATOM    548  O   LEU    42      22.069  13.469  23.309
1.00251.38           O
ATOM    549  CB  LEU    42      22.072  16.263  21.804
1.00307.99           C
ATOM    550  CG  LEU    42      21.275  17.226  20.917
1.00452.28           C
ATOM    551  CD1 LEU    42      21.449  18.654  21.442
1.00681.24           C
ATOM    552  CD2 LEU    42      21.777  17.145  19.465
1.00527.46           C
ATOM    553  HN  LEU    42      19.901  14.658  22.135
```

```
1.00195.47           H
ATOM    554  HA  LEU    42       22.268  14.712  20.342
1.00229.77           H
ATOM    555  HB1 LEU    42       23.121  16.510  21.743
1.00342.49           H
ATOM    556  HB2 LEU    42       21.739  16.360  22.828
1.00335.33           H
ATOM    557  HG  LEU    42       20.227  16.958  20.952
1.00427.79           H
ATOM    558 HD11 LEU    42       20.766  19.314  20.926
1.00909.71           H
ATOM    559 HD12 LEU    42       22.463  18.980  21.269
1.00706.80           H
ATOM    560 HD13 LEU    42       21.239  18.676  22.501
1.00858.82           H
ATOM    561 HD21 LEU    42       21.523  18.055  18.939
1.00631.69           H
ATOM    562 HD22 LEU    42       21.311  16.308  18.968
1.00628.06           H
ATOM    563 HD23 LEU    42       22.851  17.016  19.457
1.00656.75           H
ATOM    564  N   THR    43       23.830  13.526  21.909
1.00178.52           N
ATOM    565  CA  THR    43       24.632  12.624  22.727
1.00240.84           C
ATOM    566  C   THR    43       25.043  13.314  24.036
1.00372.31           C
ATOM    567  O   THR    43       25.211  14.532  24.066
1.00500.13           O
ATOM    568  CB  THR    43       25.880  12.206  21.944
1.00354.13           C
ATOM    569  CG2 THR    43       25.462  11.451  20.682
1.00463.04           C
ATOM    570  OG1 THR    43       26.618  13.365  21.581
1.00508.50           O
ATOM    571  HN  THR    43       24.202  13.880  21.074
1.00229.59           H
ATOM    572  HA  THR    43       24.046  11.747  22.945
1.00249.90           H
ATOM    573  HB  THR    43       26.496  11.566  22.554
1.00481.58           H
ATOM    574  HG1 THR    43       26.511  14.015  22.280
1.00624.31           H
ATOM    575 HG21 THR    43       24.949  12.124  20.011
1.00650.95           H
ATOM    576 HG22 THR    43       24.803  10.638  20.950
1.00580.73           H
ATOM    577 HG23 THR    43       26.339  11.055  20.191
1.00571.99           H
ATOM    578  N   PRO    44       25.207  12.574  25.114
1.00488.39           N
ATOM    579  CA  PRO    44       25.604  13.156  26.432
```

```
1.00760.13           C
ATOM    580  C   PRO    44       27.082  13.549  26.471
1.00735.36           C
ATOM    581  O   PRO    44       27.532  14.209  27.409
1.00999.99           O
ATOM    582  CB  PRO    44       25.304  12.025  27.422
1.00999.99           C
ATOM    583  CG  PRO    44       25.499  10.776  26.629
1.00856.94           C
ATOM    584  CD  PRO    44       25.036  11.107  25.207
1.00548.09           C
ATOM    585  HA  PRO    44       24.987  14.010  26.663
1.00939.00           H
ATOM    586  HB1 PRO    44       24.282  12.087  27.767
1.00999.99           H
ATOM    587  HB2 PRO    44       25.988  12.058  28.262
1.00999.99           H
ATOM    588  HG1 PRO    44       24.900   9.973  27.033
1.00999.99           H
ATOM    589  HG2 PRO    44       26.546  10.496  26.629
1.00853.53           H
ATOM    590  HD1 PRO    44       23.996  10.843  25.075
1.00602.45           H
ATOM    591  HD2 PRO    44       25.655  10.599  24.481
1.00463.32           H
ATOM    592  N   ILE    45       27.830  13.135  25.455
1.00518.33           N
ATOM    593  CA  ILE    45       29.255  13.444  25.398
1.00582.79           C
ATOM    594  C   ILE    45       29.488  14.942  25.571
1.00795.93           C
ATOM    595  O   ILE    45       30.514  15.363  26.107
1.00898.75           O
ATOM    596  CB  ILE    45       29.838  12.978  24.059
1.00474.41           C
ATOM    597  CG1 ILE    45       31.368  13.049  24.113
1.00516.90           C
ATOM    598  CG2 ILE    45       29.325  13.876  22.928
1.00760.75           C
ATOM    599  CD1 ILE    45       31.954  12.360  22.880
1.00566.81           C
ATOM    600  HN  ILE    45       27.423  12.606  24.740
1.00395.72           H
ATOM    601  HA  ILE    45       29.754  12.917  26.197
1.00711.06           H
ATOM    602  HB  ILE    45       29.533  11.958  23.872
1.00477.81           H
ATOM    603 HG11 ILE    45       31.723  12.551  25.003
1.00624.28           H
ATOM    604 HG12 ILE    45       31.680  14.083  24.131
1.00744.84           H
ATOM    605 HG21 ILE    45       28.274  14.077  23.074
```

```
1.00920.57           H
ATOM    606 HG22 ILE    45       29.466  13.376  21.980
1.00999.99           H
ATOM    607 HG23 ILE    45       29.871  14.808  22.928
1.00865.46           H
ATOM    608 HD11 ILE    45       31.706  11.309  22.902
1.00658.89           H
ATOM    609 HD12 ILE    45       33.028  12.476  22.879
1.00734.30           H
ATOM    610 HD13 ILE    45       31.544  12.808  21.987
1.00675.84           H
ATOM    611  N   ALA    46       28.530  15.742  25.113
1.00999.99           N
ATOM    612  CA  ALA    46       28.642  17.192  25.224
1.00999.99           C
ATOM    613  C   ALA    46       27.318  17.861  24.875
1.00999.99           C
ATOM    614  CB  ALA    46       29.737  17.704  24.286
1.00999.99           C
ATOM    615  OT1 ALA    46       27.215  19.063  25.062
1.00999.99           O
ATOM    616  OT2 ALA    46       26.423  17.163  24.427
1.00999.99           O
ATOM    617  HN  ALA    46       27.735  15.351  24.696
1.00999.99           H
ATOM    618  HA  ALA    46       28.907  17.447  26.239
1.00999.99           H
ATOM    619  HB1 ALA    46       29.427  17.563  23.261
1.00999.99           H
ATOM    620  HB2 ALA    46       30.650  17.156  24.464
1.00999.99           H
ATOM    621  HB3 ALA    46       29.907  18.755  24.469
1.00999.99           H
ENDMDL
TER
MODEL        7
ATOM      1  N   GLY     1       23.879  -4.071  19.872
1.00999.99           N
ATOM      2  CA  GLY     1       24.684  -2.819  19.959
1.00999.99           C
ATOM      3  C   GLY     1       24.188  -1.822  18.918
1.00999.99           C
ATOM      4  O   GLY     1       23.674  -0.757  19.260
1.00999.99           O
ATOM      5  HA1 GLY     1       25.722  -3.046  19.770
1.00999.99           H
ATOM      6  HA2 GLY     1       24.582  -2.393  20.946
1.00999.99           H
ATOM      7  HT1 GLY     1       24.057  -4.657  20.712
1.00999.99           H
ATOM      8  HT2 GLY     1       24.149  -4.598  19.016
1.00999.99           H
```

```
ATOM      9  HT3 GLY     1       22.869  -3.832  19.828
1.00999.99           H
ATOM     10  N   LEU     2       24.343  -2.174  17.646
1.00999.99           N
ATOM     11  CA  LEU     2       23.904  -1.301  16.564
1.00895.59           C
ATOM     12  C   LEU     2       22.382  -1.293  16.474
1.00532.27           C
ATOM     13  O   LEU     2       21.727  -2.284  16.794
1.00625.21           O
ATOM     14  CB  LEU     2       24.492  -1.784  15.234
1.00999.99           C
ATOM     15  CG  LEU     2       26.023  -1.817  15.319
1.00999.99           C
ATOM     16  CD1 LEU     2       26.584  -2.388  14.014
1.00999.99           C
ATOM     17  CD2 LEU     2       26.573  -0.396  15.542
1.00999.99           C
ATOM     18  HN  LEU     2       24.758  -3.037  17.432
1.00999.99           H
ATOM     19  HA  LEU     2       24.249  -0.298  16.756
1.00890.87           H
ATOM     20  HB1 LEU     2       24.192  -1.109  14.445
1.00924.07           H
ATOM     21  HB2 LEU     2       24.121  -2.775  15.018
1.00999.99           H
ATOM     22  HG  LEU     2       26.320  -2.453  16.142
1.00999.99           H
ATOM     23 HD11 LEU     2       26.049  -3.290  13.754
1.00999.99           H
ATOM     24 HD12 LEU     2       27.632  -2.616  14.140
1.00999.99           H
ATOM     25 HD13 LEU     2       26.467  -1.662  13.222
1.00999.99           H
ATOM     26 HD21 LEU     2       25.972   0.319  15.000
1.00999.99           H
ATOM     27 HD22 LEU     2       27.596  -0.342  15.190
1.00999.99           H
ATOM     28 HD23 LEU     2       26.550  -0.162  16.595
1.00999.99           H
ATOM     29  N   CYS     3       21.824  -0.164  16.042
1.00271.28           N
ATOM     30  CA  CYS     3       20.373  -0.036  15.921
1.00104.50           C
ATOM     31  C   CYS     3       19.896  -0.562  14.572  1.00
90.98           C
ATOM     32  O   CYS     3       20.106   0.071  13.537
1.00207.28           O
ATOM     33  CB  CYS     3       19.966   1.432  16.060  1.00
37.83           C
ATOM     34  SG  CYS     3       20.465   2.048  17.687  1.00
81.56           S
```

```
ATOM     35  HN  CYS     3       22.396   0.596  15.803
1.00296.55          H
ATOM     36  HA  CYS     3       19.899  -0.604  16.707
1.00166.45          H
ATOM     37  HB1 CYS     3       18.895   1.519  15.956  1.00
40.38           H
ATOM     38  HB2 CYS     3       20.450   2.015  15.291  1.00
98.45           H
ATOM     39  N   SER     4       19.242  -1.719  14.593  1.00
85.31           N
ATOM     40  CA  SER     4       18.726  -2.316  13.365  1.00
92.94           C
ATOM     41  C   SER     4       17.696  -1.392  12.742  1.00
67.37           C
ATOM     42  O   SER     4       17.659  -1.200  11.527
1.00102.28          O
ATOM     43  CB  SER     4       18.046  -3.646  13.675
1.00123.83          C
ATOM     44  OG  SER     4       17.672  -4.277  12.458
1.00178.89          O
ATOM     45  HN  SER     4       19.097  -2.175  15.447
1.00163.03          H
ATOM     46  HA  SER     4       19.536  -2.479  12.672
1.00129.78          H
ATOM     47  HB1 SER     4       17.161  -3.457  14.276
1.00105.26          H
ATOM     48  HB2 SER     4       18.722  -4.286  14.219
1.00156.87          H
ATOM     49  HG  SER     4       17.603  -3.598  11.781
1.00218.16          H
ATOM     50  N   GLU     5       16.843  -0.844  13.599  1.00
41.67           N
ATOM     51  CA  GLU     5       15.776   0.047  13.168  1.00
41.21           C
ATOM     52  C   GLU     5       15.546   1.138  14.210  1.00
41.92           C
ATOM     53  O   GLU     5       16.356   1.318  15.118  1.00
74.72           O
ATOM     54  CB  GLU     5       14.497  -0.761  12.972  1.00
49.24           C
ATOM     55  CG  GLU     5       14.144  -1.458  14.278  1.00
55.48           C
ATOM     56  CD  GLU     5       13.025  -2.467  14.050  1.00
92.06           C
ATOM     57  OE1 GLU     5       12.576  -2.578  12.921
1.00206.49          O
ATOM     58  OE2 GLU     5       12.631  -3.114  15.008
1.00197.24          O1-
ATOM     59  HN  GLU     5       16.926  -1.058  14.552  1.00
45.52           H
ATOM     60  HA  GLU     5       16.044   0.502  12.235  1.00
60.63           H
```

```
ATOM     61  HB1 GLU     5       14.653  -1.503  12.202  1.00
61.59           H
ATOM     62  HB2 GLU     5       13.697  -0.106  12.685  1.00
63.66           H
ATOM     63  HG1 GLU     5       13.823  -0.723  15.001  1.00
58.03           H
ATOM     64  HG2 GLU     5       15.018  -1.965  14.649  1.00
57.77           H
ATOM     65  N   ASN     6       14.440   1.861  14.076  1.00
50.73           N
ATOM     66  CA  ASN     6       14.128   2.929  15.020  1.00
71.04           C
ATOM     67  C   ASN     6       14.001   2.362  16.431  1.00
65.17           C
ATOM     68  O   ASN     6       14.454   2.972  17.400  1.00
94.86           O
ATOM     69  CB  ASN     6       12.820   3.616  14.623  1.00
94.83           C
ATOM     70  CG  ASN     6       13.030   4.448  13.362
1.00160.94           C
ATOM     71  ND2 ASN     6       12.001   4.785  12.634
1.00244.88           N
ATOM     72  OD1 ASN     6       14.163   4.798  13.030
1.00219.73           O
ATOM     73  HN  ASN     6       13.827   1.677  13.335  1.00
72.81           H
ATOM     74  HA  ASN     6       14.925   3.657  15.005  1.00
96.02           H
ATOM     75  HB1 ASN     6       12.495   4.261  15.424
1.00111.09           H
ATOM     76  HB2 ASN     6       12.064   2.866  14.436  1.00
98.13           H
ATOM     77 HD21 ASN     6       11.101   4.505  12.900
1.00272.43           H
ATOM     78 HD22 ASN     6       12.128   5.320  11.822
1.00336.78           H
ATOM     79  N   GLY     7       13.386   1.184  16.535  1.00
51.80           N
ATOM     80  CA  GLY     7       13.204   0.518  17.828  1.00
71.47           C
ATOM     81  C   GLY     7       13.900  -0.836  17.830  1.00
41.04           C
ATOM     82  O   GLY     7       13.260  -1.877  17.974  1.00
44.54           O
ATOM     83  HN  GLY     7       13.054   0.747  15.724  1.00
45.49           H
ATOM     84  HA1 GLY     7       12.155   0.368  18.002
1.00103.52           H
ATOM     85  HA2 GLY     7       13.613   1.130  18.621
1.00108.67           H
ATOM     86  N   ASP     8       15.214  -0.810  17.662  1.00
27.00           N
```

```
ATOM     87  CA  ASP     8       16.001  -2.039  17.635  1.00
14.15          C
ATOM     88  C   ASP     8       15.864  -2.798  18.947  1.00
9.37          C
ATOM     89  O   ASP     8       15.663  -4.013  18.954  1.00
14.61          O
ATOM     90  CB  ASP     8       17.473  -1.713  17.382  1.00
15.06          C
ATOM     91  CG  ASP     8       18.281  -3.001  17.277  1.00
21.22          C
ATOM     92  OD1 ASP     8       19.446  -2.919  16.927
1.00119.56          O
ATOM     93  OD2 ASP     8       17.722  -4.051  17.549
1.00133.27          O1-
ATOM     94  HN  ASP     8       15.662   0.053  17.547  1.00
35.63          H
ATOM     95  HA  ASP     8       15.642  -2.662  16.836  1.00
20.92          H
ATOM     96  HB1 ASP     8       17.854  -1.118  18.200  1.00
29.39          H
ATOM     97  HB2 ASP     8       17.564  -1.155  16.461  1.00
44.14          H
ATOM     98  N   CYS     9       15.974  -2.078  20.054  1.00
5.93          N
ATOM     99  CA  CYS     9       15.858  -2.700  21.367  1.00
7.54          C
ATOM    100  C   CYS     9       14.471  -3.304  21.543  1.00
14.60          C
ATOM    101  O   CYS     9       14.316  -4.410  22.059  1.00
25.78          O
ATOM    102  CB  CYS     9       16.105  -1.670  22.457  1.00
6.63          C
ATOM    103  SG  CYS     9       17.849  -1.223  22.436  1.00
10.91          S
ATOM    104  HN  CYS     9       16.131  -1.114  19.985  1.00
6.51          H
ATOM    105  HA  CYS     9       16.603  -3.471  21.455  1.00
11.12          H
ATOM    106  HB1 CYS     9       15.855  -2.089  23.417  1.00
11.28          H
ATOM    107  HB2 CYS     9       15.502  -0.792  22.273  1.00
5.04          H
ATOM    108  N   ALA    10       13.467  -2.558  21.098  1.00
16.26          N
ATOM    109  CA  ALA    10       12.082  -3.001  21.189  1.00
32.04          C
ATOM    110  C   ALA    10       11.181  -2.054  20.399  1.00
45.47          C
ATOM    111  O   ALA    10       11.611  -0.976  19.990
1.00119.04          O
ATOM    112  CB  ALA    10       11.636  -3.048  22.654  1.00
30.10          C
```

```
ATOM    113  HN  ALA    10       13.666  -1.688  20.694  1.00
12.81           H
ATOM    114  HA  ALA    10       12.002  -3.991  20.767  1.00
47.14           H
ATOM    115  HB1 ALA    10       12.445  -3.420  23.263  1.00
84.37           H
ATOM    116  HB2 ALA    10       10.782  -3.704  22.749
1.00118.20           H
ATOM    117  HB3 ALA    10       11.364  -2.056  22.983
1.00117.32           H
ATOM    118  N   ALA    11        9.936  -2.461  20.182  1.00
30.62           N
ATOM    119  CA  ALA    11        8.997  -1.632  19.434  1.00
35.24           C
ATOM    120  C   ALA    11        8.778  -0.293  20.136  1.00
22.78           C
ATOM    121  O   ALA    11        8.727   0.753  19.491  1.00
53.33           O
ATOM    122  CB  ALA    11        7.660  -2.359  19.291  1.00
60.49           C
ATOM    123  HN  ALA    11        9.644  -3.330  20.527  1.00
50.37           H
ATOM    124  HA  ALA    11        9.400  -1.449  18.450  1.00
41.70           H
ATOM    125  HB1 ALA    11        6.952  -1.717  18.787
1.00157.06           H
ATOM    126  HB2 ALA    11        7.281  -2.614  20.269
1.00148.83           H
ATOM    127  HB3 ALA    11        7.800  -3.262  18.715
1.00137.06           H
ATOM    128  N   ASP    12        8.648  -0.334  21.460  1.00
18.98           N
ATOM    129  CA  ASP    12        8.431   0.883  22.238  1.00
31.05           C
ATOM    130  C   ASP    12        9.749   1.604  22.509  1.00
22.98           C
ATOM    131  O   ASP    12        9.799   2.833  22.550  1.00
37.14           O
ATOM    132  CB  ASP    12        7.757   0.537  23.568  1.00
48.35           C
ATOM    133  CG  ASP    12        6.325   0.073  23.322
1.00104.83           C
ATOM    134  OD1 ASP    12        5.743  -0.496  24.230
1.00258.73           O
ATOM    135  OD2 ASP    12        5.833   0.292  22.227
1.00213.46           O1-
ATOM    136  HN  ASP    12        8.696  -1.198  21.921  1.00
36.19           H
ATOM    137  HA  ASP    12        7.780   1.544  21.683  1.00
49.76           H
ATOM    138  HB1 ASP    12        7.745   1.410  24.202  1.00
95.71           H
```

```
ATOM    139  HB2 ASP    12        8.311  -0.253  24.054  1.00
54.37           H
ATOM    140  N   GLU    13       10.813   0.830  22.701  1.00
17.60           N
ATOM    141  CA  GLU    13       12.128   1.403  22.976  1.00
12.01           C
ATOM    142  C   GLU    13       12.715   2.046  21.721  1.00
10.07           C
ATOM    143  O   GLU    13       12.411   1.637  20.601  1.00
12.81           O
ATOM    144  CB  GLU    13       13.071   0.311  23.484  1.00
11.74           C
ATOM    145  CG  GLU    13       12.566  -0.206  24.833  1.00
13.49           C
ATOM    146  CD  GLU    13       13.416  -1.384  25.294
1.00142.36           C
ATOM    147  OE1 GLU    13       12.842  -2.378  25.706
1.00339.06           O
ATOM    148  OE2 GLU    13       14.628  -1.276  25.226
1.00335.28           O1-
ATOM    149  HN  GLU    13       10.712  -0.144  22.662  1.00
28.36           H
ATOM    150  HA  GLU    13       12.026   2.154  23.744  1.00
13.06           H
ATOM    151  HB1 GLU    13       14.063   0.719  23.604  1.00
13.76           H
ATOM    152  HB2 GLU    13       13.098  -0.502  22.771  1.00
9.94           H
ATOM    153  HG1 GLU    13       11.539  -0.522  24.736  1.00
66.02           H
ATOM    154  HG2 GLU    13       12.628   0.586  25.565  1.00
56.23           H
ATOM    155  N   CYS    14       13.569   3.054  21.919  1.00
9.12           N
ATOM    156  CA  CYS    14       14.212   3.753  20.805  1.00
10.69           C
ATOM    157  C   CYS    14       15.666   3.318  20.692  1.00
9.56           C
ATOM    158  O   CYS    14       16.262   2.897  21.677  1.00
13.62           O
ATOM    159  CB  CYS    14       14.154   5.267  21.026  1.00
13.96           C
ATOM    160  SG  CYS    14       15.151   6.096  19.761  1.00
42.96           S
ATOM    161  HN  CYS    14       13.779   3.328  22.836  1.00
9.76           H
ATOM    162  HA  CYS    14       13.702   3.516  19.881  1.00
15.54           H
ATOM    163  HB1 CYS    14       14.544   5.504  22.004  1.00
50.72           H
ATOM    164  HB2 CYS    14       13.130   5.602  20.955  1.00
46.13           H
```

```
ATOM    165  N   CYS    15       16.229   3.418  19.491  1.00
9.60           N
ATOM    166  CA  CYS    15       17.622   3.027  19.270  1.00
9.28           C
ATOM    167  C   CYS    15       18.239   3.913  18.195  1.00
9.92           C
ATOM    168  O   CYS    15       17.660   4.093  17.124  1.00
13.31           O
ATOM    169  CB  CYS    15       17.688   1.562  18.827  1.00
12.90           C
ATOM    170  SG  CYS    15       19.386   0.948  18.993  1.00
39.11           S
ATOM    171  HN  CYS    15       15.704   3.762  18.740  1.00
13.12           H
ATOM    172  HA  CYS    15       18.182   3.144  20.189  1.00
8.26           H
ATOM    173  HB1 CYS    15       17.379   1.485  17.795  1.00
24.82           H
ATOM    174  HB2 CYS    15       17.031   0.969  19.444  1.00
27.31           H
ATOM    175  N   VAL    16       19.414   4.470  18.482  1.00
9.56           N
ATOM    176  CA  VAL    16       20.088   5.340  17.520  1.00
11.99           C
ATOM    177  C   VAL    16       21.601   5.221  17.662  1.00
7.73           C
ATOM    178  O   VAL    16       22.138   5.288  18.767  1.00
8.83           O
ATOM    179  CB  VAL    16       19.658   6.790  17.747  1.00
18.37           C
ATOM    180  CG1 VAL    16       20.206   7.287  19.086  1.00
39.61           C
ATOM    181  CG2 VAL    16       20.201   7.663  16.615
1.00115.21           C
ATOM    182  HN  VAL    16       19.836   4.295  19.352  1.00
9.76           H
ATOM    183  HA  VAL    16       19.811   5.047  16.517  1.00
17.27           H
ATOM    184  HB  VAL    16       18.580   6.845  17.759  1.00
52.63           H
ATOM    185 HG11 VAL    16       19.706   8.203  19.361
1.00128.07           H
ATOM    186 HG12 VAL    16       21.268   7.470  18.995
1.00154.13           H
ATOM    187 HG13 VAL    16       20.034   6.539  19.845
1.00135.74           H
ATOM    188 HG21 VAL    16       19.848   8.675  16.742
1.00229.05           H
ATOM    189 HG22 VAL    16       19.855   7.277  15.667
1.00261.82           H
ATOM    190 HG23 VAL    16       21.280   7.651  16.637
1.00210.07           H
```

```
ATOM    191  N   ASP    17       22.284   5.044  16.532  1.00
14.34           N
ATOM    192  CA  ASP    17       23.741   4.915  16.532  1.00
11.90           C
ATOM    193  C   ASP    17       24.394   6.231  16.127  1.00
13.15           C
ATOM    194  O   ASP    17       24.163   6.739  15.029  1.00
25.96           O
ATOM    195  CB  ASP    17       24.166   3.822  15.551  1.00
20.80           C
ATOM    196  CG  ASP    17       23.754   2.453  16.083  1.00
28.72           C
ATOM    197  OD1 ASP    17       23.420   2.372  17.254
1.00135.42           O
ATOM    198  OD2 ASP    17       23.779   1.508  15.312
1.00114.17           O1-
ATOM    199  HN  ASP    17       21.799   4.998  15.682  1.00
28.29           H
ATOM    200  HA  ASP    17       24.082   4.645  17.522  1.00
10.49           H
ATOM    201  HB1 ASP    17       25.238   3.849  15.427  1.00
22.42           H
ATOM    202  HB2 ASP    17       23.692   3.994  14.595  1.00
29.52           H
ATOM    203  N   THR    18       25.225   6.773  17.016  1.00
11.55           N
ATOM    204  CA  THR    18       25.930   8.027  16.746  1.00
17.10           C
ATOM    205  C   THR    18       27.403   7.749  16.491  1.00
10.63           C
ATOM    206  O   THR    18       27.901   6.671  16.808  1.00
6.49           O
ATOM    207  CB  THR    18       25.800   8.978  17.936  1.00
28.52           C
ATOM    208  CG2 THR    18       24.361   9.480  18.046  1.00
45.38           C
ATOM    209  OG1 THR    18       26.163   8.297  19.126  1.00
26.49           O
ATOM    210  HN  THR    18       25.376   6.314  17.868  1.00
13.96           H
ATOM    211  HA  THR    18       25.508   8.502  15.871  1.00
26.43           H
ATOM    212  HB  THR    18       26.458   9.822  17.793  1.00
37.43           H
ATOM    213  HG1 THR    18       25.607   8.622  19.837  1.00
71.62           H
ATOM    214 HG21 THR    18       24.315  10.275  18.774
1.00100.44           H
ATOM    215 HG22 THR    18       23.720   8.669  18.355
1.00129.42           H
ATOM    216 HG23 THR    18       24.036   9.852  17.086
1.00156.21           H
```

```
ATOM    217  N   VAL    19      28.091   8.736  15.935  1.00
16.32           N
ATOM    218  CA  VAL    19      29.500   8.618  15.649  1.00
13.34           C
ATOM    219  C   VAL    19      29.984   9.884  14.977  1.00
25.88           C
ATOM    220  O   VAL    19      29.548  10.249  13.885  1.00
42.11           O
ATOM    221  CB  VAL    19      29.806   7.409  14.763  1.00
15.48           C
ATOM    222  CG1 VAL    19      28.884   7.402  13.539  1.00
29.06           C
ATOM    223  CG2 VAL    19      31.270   7.484  14.308  1.00
20.67           C
ATOM    224  HN  VAL    19      27.646   9.578  15.726  1.00
26.89           H
ATOM    225  HA  VAL    19      30.028   8.500  16.586  1.00
8.08           H
ATOM    226  HB  VAL    19      29.655   6.507  15.332  1.00
11.49           H
ATOM    227 HG11 VAL    19      28.944   6.439  13.051
1.00137.43           H
ATOM    228 HG12 VAL    19      29.194   8.172  12.849  1.00
93.29           H
ATOM    229 HG13 VAL    19      27.866   7.584  13.848
1.00108.69           H
ATOM    230 HG21 VAL    19      31.363   8.220  13.522  1.00
99.42           H
ATOM    231 HG22 VAL    19      31.585   6.520  13.941
1.00111.36           H
ATOM    232 HG23 VAL    19      31.892   7.776  15.146  1.00
77.11           H
ATOM    233  N   PHE    20      30.884  10.544  15.660  1.00
24.58           N
ATOM    234  CA  PHE    20      31.467  11.787  15.187  1.00
40.34           C
ATOM    235  C   PHE    20      32.816  11.530  14.532  1.00
40.37           C
ATOM    236  O   PHE    20      33.047  11.929  13.390  1.00
66.02           O
ATOM    237  CB  PHE    20      31.632  12.755  16.371  1.00
48.79           C
ATOM    238  CG  PHE    20      31.907  11.992  17.659  1.00
35.75           C
ATOM    239  CD1 PHE    20      30.913  11.180  18.238  1.00
30.20           C
ATOM    240  CD2 PHE    20      33.155  12.112  18.285  1.00
40.95           C
ATOM    241  CE1 PHE    20      31.176  10.498  19.428  1.00
30.41           C
ATOM    242  CE2 PHE    20      33.413  11.424  19.476  1.00
45.84           C
```

```
ATOM    243  CZ  PHE    20      32.424  10.618  20.047  1.00
40.81           C
ATOM    244  HN  PHE    20      31.158  10.183  16.518  1.00
16.73           H
ATOM    245  HA  PHE    20      30.809  12.239  14.456  1.00
59.91           H
ATOM    246  HB1 PHE    20      30.733  13.311  16.485  1.00
67.15           H
ATOM    247  HB2 PHE    20      32.445  13.441  16.176  1.00
58.17           H
ATOM    248  HD1 PHE    20      29.944  11.075  17.767  1.00
33.34           H
ATOM    249  HD2 PHE    20      33.921  12.733  17.847  1.00
49.35           H
ATOM    250  HE1 PHE    20      30.414   9.877  19.868  1.00
30.61           H
ATOM    251  HE2 PHE    20      34.375  11.516  19.955  1.00
61.27           H
ATOM    252  HZ  PHE    20      32.623  10.088  20.967  1.00
52.66           H
ATOM    253  N   GLU    21      33.715  10.894  15.275  1.00
41.31           N
ATOM    254  CA  GLU    21      35.046  10.618  14.793  1.00
56.16           C
ATOM    255  C   GLU    21      35.314   9.124  14.728  1.00
58.61           C
ATOM    256  O   GLU    21      34.438   8.305  15.004
1.00201.19           O
ATOM    257  CB  GLU    21      36.010  11.280  15.750  1.00
57.37           C
ATOM    258  CG  GLU    21      35.948  10.585  17.112
1.00193.56           C
ATOM    259  CD  GLU    21      36.671  11.429  18.156
1.00304.62           C
ATOM    260  OE1 GLU    21      36.600  11.081  19.323
1.00451.81           O
ATOM    261  OE2 GLU    21      37.286  12.411  17.774
1.00442.88           O1-
ATOM    262  HN  GLU    21      33.493  10.629  16.186  1.00
50.82           H
ATOM    263  HA  GLU    21      35.185  11.046  13.810  1.00
85.72           H
ATOM    264  HB1 GLU    21      35.725  12.310  15.865  1.00
46.37           H
ATOM    265  HB2 GLU    21      36.999  11.215  15.357
1.00131.81           H
ATOM    266  HG1 GLU    21      36.421   9.621  17.041
1.00314.91           H
ATOM    267  HG2 GLU    21      34.918  10.455  17.401
1.00266.92           H
ATOM    268  N   GLY    22      36.538   8.784  14.353  1.00
86.70           N
```

```
ATOM    269  CA  GLY    22       36.945   7.382  14.239
1.00104.66          C
ATOM    270  C   GLY    22       37.592   6.891  15.529  1.00
71.50           C
ATOM    271  O   GLY    22       37.805   5.692  15.713  1.00
89.87           O
ATOM    272  HN  GLY    22       37.182   9.496  14.149
1.00214.64          H
ATOM    273  HA1 GLY    22       37.657   7.285  13.431
1.00152.47          H
ATOM    274  HA2 GLY    22       36.079   6.770  14.021
1.00118.15          H
ATOM    275  N   ASP    23       37.911   7.825  16.414  1.00
44.68           N
ATOM    276  CA  ASP    23       38.542   7.485  17.683  1.00
43.33           C
ATOM    277  C   ASP    23       37.640   6.590  18.523  1.00
37.55           C
ATOM    278  O   ASP    23       38.094   5.607  19.108  1.00
61.37           O
ATOM    279  CB  ASP    23       38.858   8.761  18.463  1.00
40.74           C
ATOM    280  CG  ASP    23       39.679   8.425  19.704
1.00141.86          C
ATOM    281  OD1 ASP    23       40.168   9.348  20.334
1.00328.18          O
ATOM    282  OD2 ASP    23       39.806   7.250  20.005
1.00304.58          O1-
ATOM    283  HN  ASP    23       37.724   8.765  16.208  1.00
40.86           H
ATOM    284  HA  ASP    23       39.462   6.964  17.485  1.00
72.12           H
ATOM    285  HB1 ASP    23       37.933   9.231  18.764  1.00
94.15           H
ATOM    286  HB2 ASP    23       39.415   9.438  17.833  1.00
93.26           H
ATOM    287  N   MET    24       36.362   6.944  18.589  1.00
23.24           N
ATOM    288  CA  MET    24       35.400   6.173  19.374  1.00
32.50           C
ATOM    289  C   MET    24       33.985   6.356  18.835  1.00
25.13           C
ATOM    290  O   MET    24       33.617   7.439  18.380  1.00
54.40           O
ATOM    291  CB  MET    24       35.451   6.622  20.836  1.00
47.36           C
ATOM    292  CG  MET    24       34.467   5.797  21.667
1.00151.73          C
ATOM    293  SD  MET    24       34.653   6.219  23.419
1.00209.23          S
ATOM    294  CE  MET    24       33.971   7.895  23.338
1.00243.29          C
```

```
ATOM    295  HN  MET    24       36.063   7.742  18.107  1.00
17.32           H
ATOM    296  HA  MET    24       35.660   5.125  19.324  1.00
48.94           H
ATOM    297  HB1 MET    24       35.186   7.666  20.899
1.00124.40           H
ATOM    298  HB2 MET    24       36.451   6.482  21.219
1.00166.96           H
ATOM    299  HG1 MET    24       34.672   4.745  21.528
1.00331.71           H
ATOM    300  HG2 MET    24       33.456   6.009  21.352
1.00302.30           H
ATOM    301  HE1 MET    24       33.622   8.188  24.319
1.00340.44           H
ATOM    302  HE2 MET    24       33.147   7.920  22.645
1.00373.88           H
ATOM    303  HE3 MET    24       34.740   8.581  23.007
1.00386.81           H
ATOM    304  N   VAL    25       33.190   5.287  18.900  1.00
22.44           N
ATOM    305  CA  VAL    25       31.804   5.323  18.428  1.00
15.37           C
ATOM    306  C   VAL    25       30.844   5.396  19.610  1.00
17.47           C
ATOM    307  O   VAL    25       31.118   4.865  20.686  1.00
29.52           O
ATOM    308  CB  VAL    25       31.494   4.078  17.591  1.00
23.36           C
ATOM    309  CG1 VAL    25       29.990   4.035  17.258  1.00
58.39           C
ATOM    310  CG2 VAL    25       32.312   4.128  16.297  1.00
55.25           C
ATOM    311  HN  VAL    25       33.542   4.455  19.280  1.00
48.11           H
ATOM    312  HA  VAL    25       31.656   6.201  17.809  1.00
9.25           H
ATOM    313  HB  VAL    25       31.762   3.194  18.153  1.00
52.45           H
ATOM    314 HG11 VAL    25       29.623   5.040  17.105
1.00171.07           H
ATOM    315 HG12 VAL    25       29.455   3.582  18.078
1.00166.22           H
ATOM    316 HG13 VAL    25       29.831   3.454  16.361
1.00134.76           H
ATOM    317 HG21 VAL    25       31.951   4.933  15.674
1.00141.88           H
ATOM    318 HG22 VAL    25       32.212   3.191  15.770
1.00184.50           H
ATOM    319 HG23 VAL    25       33.353   4.299  16.534
1.00133.90           H
ATOM    320  N   THR    26       29.719   6.079  19.402  1.00
13.60           N
```

```
ATOM    321  CA  THR    26      28.708   6.251  20.447  1.00
21.19           C
ATOM    322  C   THR    26      27.343   5.749  19.987  1.00
16.55           C
ATOM    323  O   THR    26      26.942   5.959  18.841  1.00
11.43           O
ATOM    324  CB  THR    26      28.611   7.732  20.802  1.00
27.07           C
ATOM    325  CG2 THR    26      27.507   7.950  21.840  1.00
44.39           C
ATOM    326  OG1 THR    26      29.856   8.162  21.335  1.00
34.75           O
ATOM    327  HN  THR    26      29.567   6.488  18.524  1.00
10.81           H
ATOM    328  HA  THR    26      29.000   5.703  21.332  1.00
33.16           H
ATOM    329  HB  THR    26      28.385   8.296  19.907  1.00
20.60           H
ATOM    330  HG1 THR    26      30.221   7.442  21.855  1.00
77.72           H
ATOM    331 HG21 THR    26      27.565   8.960  22.219
1.00126.99           H
ATOM    332 HG22 THR    26      27.636   7.254  22.654
1.00108.32           H
ATOM    333 HG23 THR    26      26.542   7.793  21.382
1.00151.99           H
ATOM    334  N   ARG    27      26.629   5.088  20.897  1.00
22.38           N
ATOM    335  CA  ARG    27      25.299   4.557  20.600  1.00
20.21           C
ATOM    336  C   ARG    27      24.421   4.636  21.846  1.00
16.96           C
ATOM    337  O   ARG    27      24.930   4.758  22.960  1.00
20.06           O
ATOM    338  CB  ARG    27      25.408   3.102  20.137  1.00
22.14           C
ATOM    339  CG  ARG    27      25.925   2.237  21.288
1.00124.30           C
ATOM    340  CD  ARG    27      26.299   0.852  20.758
1.00109.38           C
ATOM    341  NE  ARG    27      27.454   0.945  19.873
1.00227.73           N
ATOM    342  CZ  ARG    27      27.841  -0.092  19.138
1.00426.12           C
ATOM    343  NH1 ARG    27      28.878   0.015  18.352
1.00767.09           N1+
ATOM    344  NH2 ARG    27      27.184  -1.216  19.199
1.00581.78           N
ATOM    345  HN  ARG    27      27.004   4.960  21.793  1.00
30.75           H
ATOM    346  HA  ARG    27      24.844   5.142  19.813  1.00
23.13           H
```

```
ATOM    347  HB1 ARG    27       26.095   3.040  19.306  1.00
87.46           H
ATOM    348  HB2 ARG    27       24.436   2.750  19.828
1.00103.36           H
ATOM    349  HG1 ARG    27       25.154   2.137  22.037
1.00281.97           H
ATOM    350  HG2 ARG    27       26.796   2.703  21.726
1.00276.19           H
ATOM    351  HD1 ARG    27       25.461   0.440  20.215
1.00183.60           H
ATOM    352  HD2 ARG    27       26.536   0.204  21.588
1.00142.93           H
ATOM    353  HE  ARG    27       27.953   1.787  19.819
1.00372.53           H
ATOM    354 HH11 ARG    27       29.382   0.879  18.305
1.00910.59           H
ATOM    355 HH12 ARG    27       29.169  -0.764  17.799
1.00999.99           H
ATOM    356 HH21 ARG    27       26.388  -1.300  19.800
1.00532.54           H
ATOM    357 HH22 ARG    27       27.474  -1.998  18.645
1.00948.84           H
ATOM    358  N   SER    28       23.103   4.574  21.658  1.00
14.60           N
ATOM    359  CA  SER    28       22.177   4.650  22.788  1.00
14.92           C
ATOM    360  C   SER    28       20.904   3.868  22.498  1.00
12.12           C
ATOM    361  O   SER    28       20.597   3.563  21.346  1.00
13.41           O
ATOM    362  CB  SER    28       21.826   6.110  23.073  1.00
23.26           C
ATOM    363  OG  SER    28       21.094   6.639  21.975
1.00146.32           O
ATOM    364  HN  SER    28       22.746   4.482  20.747  1.00
15.23           H
ATOM    365  HA  SER    28       22.650   4.231  23.665  1.00
15.44           H
ATOM    366  HB1 SER    28       22.736   6.677  23.216  1.00
88.73           H
ATOM    367  HB2 SER    28       21.222   6.171  23.963
1.00124.68           H
ATOM    368  HG  SER    28       20.159   6.596  22.193
1.00242.47           H
ATOM    369  N   CYS    29       20.163   3.550  23.555  1.00
10.48           N
ATOM    370  CA  CYS    29       18.919   2.808  23.407  1.00
9.97           C
ATOM    371  C   CYS    29       18.157   2.798  24.731  1.00
11.60           C
ATOM    372  O   CYS    29       18.709   2.449  25.774  1.00
15.64           O
```

```
ATOM    373  CB  CYS    29      19.216   1.370  22.949  1.00
9.52          C
ATOM    374  SG  CYS    29      17.828   0.738  21.996  1.00
10.40          S
ATOM    375  HN  CYS    29      20.454   3.826  24.448  1.00
11.10          H
ATOM    376  HA  CYS    29      18.312   3.295  22.662  1.00
11.08          H
ATOM    377  HB1 CYS    29      19.372   0.729  23.805  1.00
9.33          H
ATOM    378  HB2 CYS    29      20.100   1.361  22.330  1.00
10.78          H
ATOM    379  N   GLU    30      16.890   3.195  24.682  1.00
13.52          N
ATOM    380  CA  GLU    30      16.067   3.240  25.885  1.00
17.41          C
ATOM    381  C   GLU    30      15.627   1.842  26.303  1.00
10.81          C
ATOM    382  O   GLU    30      15.482   0.949  25.470  1.00
23.22          O
ATOM    383  CB  GLU    30      14.830   4.102  25.643  1.00
37.83          C
ATOM    384  CG  GLU    30      15.257   5.523  25.283
1.00108.13          C
ATOM    385  CD  GLU    30      15.909   6.192  26.488
1.00240.52          C
ATOM    386  OE1 GLU    30      15.760   5.672  27.582
1.00422.56          O
ATOM    387  OE2 GLU    30      16.545   7.216  26.302
1.00410.81          O1-
ATOM    388  HN  GLU    30      16.504   3.470  23.824  1.00
15.61          H
ATOM    389  HA  GLU    30      16.642   3.678  26.686  1.00
24.60          H
ATOM    390  HB1 GLU    30      14.226   4.125  26.538  1.00
72.67          H
ATOM    391  HB2 GLU    30      14.252   3.682  24.831  1.00
56.47          H
ATOM    392  HG1 GLU    30      14.390   6.090  24.984
1.00196.66          H
ATOM    393  HG2 GLU    30      15.963   5.489  24.466
1.00140.67          H
ATOM    394  N   LYS    31      15.403   1.671  27.605  1.00
11.96          N
ATOM    395  CA  LYS    31      14.961   0.387  28.149  1.00
11.40          C
ATOM    396  C   LYS    31      13.720   0.587  29.008  1.00
10.99          C
ATOM    397  O   LYS    31      13.629   1.548  29.770  1.00
12.86          O
ATOM    398  CB  LYS    31      16.067  -0.235  29.001  1.00
21.40          C
```

```
ATOM    399  CG  LYS    31      17.314  -0.457  28.140  1.00
57.81           C
ATOM    400  CD  LYS    31      18.338  -1.311  28.899
1.00115.52          C
ATOM    401  CE  LYS    31      18.968  -0.494  30.031
1.00250.23          C
ATOM    402  NZ  LYS    31      20.118  -1.250  30.604
1.00462.10          N1+
ATOM    403  HN  LYS    31      15.528   2.428  28.214  1.00
26.13           H
ATOM    404  HA  LYS    31      14.720  -0.290  27.343  1.00
13.87           H
ATOM    405  HB1 LYS    31      15.728  -1.181  29.396  1.00
37.63           H
ATOM    406  HB2 LYS    31      16.299   0.432  29.815  1.00
47.19           H
ATOM    407  HG1 LYS    31      17.754   0.497  27.895
1.00126.32          H
ATOM    408  HG2 LYS    31      17.032  -0.965  27.227
1.00114.19          H
ATOM    409  HD1 LYS    31      19.111  -1.631  28.218
1.00198.45          H
ATOM    410  HD2 LYS    31      17.845  -2.177  29.313
1.00200.14          H
ATOM    411  HE1 LYS    31      18.235  -0.322  30.806
1.00374.64          H
ATOM    412  HE2 LYS    31      19.316   0.453  29.645
1.00403.27          H
ATOM    413  HZ1 LYS    31      19.917  -1.491  31.594
1.00627.38          H
ATOM    414  HZ2 LYS    31      20.976  -0.662  30.557
1.00622.26          H
ATOM    415  HZ3 LYS    31      20.265  -2.123  30.060
1.00619.20          H
ATOM    416  N   THR    32      12.769  -0.326  28.880  1.00
15.51           N
ATOM    417  CA  THR    32      11.534  -0.239  29.651  1.00
22.44           C
ATOM    418  C   THR    32      11.801  -0.555  31.119  1.00
26.60           C
ATOM    419  O   THR    32      11.378  -1.592  31.630  1.00
57.43           O
ATOM    420  CB  THR    32      10.496  -1.214  29.092  1.00
56.28           C
ATOM    421  CG2 THR    32      10.397  -1.045  27.576
1.00102.70          C
ATOM    422  OG1 THR    32      10.884  -2.546  29.400
1.00111.17          O
ATOM    423  HN  THR    32      12.898  -1.068  28.255  1.00
18.79           H
ATOM    424  HA  THR    32      11.147   0.767  29.575  1.00
19.67           H
```

```
ATOM    425  HB  THR    32       9.534  -1.008  29.536  1.00
84.25           H
ATOM    426  HG1 THR    32      10.258  -3.141  28.982
1.00205.03           H
ATOM    427 HG21 THR    32      11.366  -1.219  27.131
1.00220.71           H
ATOM    428 HG22 THR    32      10.071  -0.042  27.346
1.00174.67           H
ATOM    429 HG23 THR    32       9.686  -1.755  27.180
1.00217.44           H
ATOM    430  N   THR    33      12.502   0.352  31.792  1.00
22.47           N
ATOM    431  CA  THR    33      12.819   0.172  33.206  1.00
41.60           C
ATOM    432  C   THR    33      11.626   0.508  34.050  1.00
38.03           C
ATOM    433  O   THR    33      11.721   1.195  35.066  1.00
52.73           O
ATOM    434  CB  THR    33      13.992   1.062  33.592  1.00
63.25           C
ATOM    435  CG2 THR    33      14.624   0.572  34.899
1.00121.65           C
ATOM    436  OG1 THR    33      14.964   1.038  32.555  1.00
96.20           O
ATOM    437  HN  THR    33      12.807   1.162  31.332  1.00
23.82           H
ATOM    438  HA  THR    33      13.064  -0.854  33.394  1.00
67.12           H
ATOM    439  HB  THR    33      13.630   2.066  33.723
1.00107.59           H
ATOM    440  HG1 THR    33      15.136   0.122  32.330
1.00176.27           H
ATOM    441 HG21 THR    33      13.915   0.680  35.706
1.00255.23           H
ATOM    442 HG22 THR    33      15.505   1.158  35.114
1.00245.37           H
ATOM    443 HG23 THR    33      14.899  -0.468  34.798
1.00185.78           H
ATOM    444  N   GLY    34      10.502   0.003  33.614  1.00
40.93           N
ATOM    445  CA  GLY    34       9.266   0.223  34.323  1.00
45.59           C
ATOM    446  C   GLY    34       8.677   1.574  33.943  1.00
40.97           C
ATOM    447  O   GLY    34       7.603   1.956  34.410  1.00
82.17           O
ATOM    448  HN  GLY    34      10.513  -0.531  32.803  1.00
54.00           H
ATOM    449  HA1 GLY    34       9.477   0.201  35.376  1.00
54.41           H
ATOM    450  HA2 GLY    34       8.565  -0.563  34.076  1.00
62.09           H
```

```
ATOM    451  N   ASN    35       9.394   2.284  33.075  1.00
32.51           N
ATOM    452  CA  ASN    35       8.957   3.593  32.602  1.00
33.20           C
ATOM    453  C   ASN    35       9.463   3.823  31.183  1.00
23.17           C
ATOM    454  O   ASN    35      10.575   3.419  30.838  1.00
27.01           O
ATOM    455  CB  ASN    35       9.490   4.693  33.521  1.00
54.51           C
ATOM    456  CG  ASN    35       8.988   4.472  34.944  1.00
80.73           C
ATOM    457  ND2 ASN    35       9.825   4.561  35.942
1.00220.46           N
ATOM    458  OD1 ASN    35       7.804   4.208  35.152
1.00117.80           O
ATOM    459  HN  ASN    35      10.235   1.913  32.739  1.00
51.15           H
ATOM    460  HA  ASN    35       7.877   3.631  32.600  1.00
47.21           H
ATOM    461  HB1 ASN    35       9.143   5.652  33.165  1.00
68.31           H
ATOM    462  HB2 ASN    35      10.569   4.674  33.512  1.00
57.75           H
ATOM    463 HD21 ASN    35      10.768   4.769  35.773
1.00405.39           H
ATOM    464 HD22 ASN    35       9.511   4.420  36.859
1.00245.64           H
ATOM    465  N   PHE    36       8.639   4.467  30.359  1.00
35.72           N
ATOM    466  CA  PHE    36       9.003   4.740  28.976  1.00
33.41           C
ATOM    467  C   PHE    36       9.627   6.126  28.848  1.00
28.51           C
ATOM    468  O   PHE    36       9.144   7.092  29.439  1.00
45.23           O
ATOM    469  CB  PHE    36       7.746   4.664  28.117  1.00
67.53           C
ATOM    470  CG  PHE    36       7.226   3.248  28.115  1.00
86.71           C
ATOM    471  CD1 PHE    36       6.330   2.834  29.106
1.00106.51           C
ATOM    472  CD2 PHE    36       7.638   2.350  27.123  1.00
99.48           C
ATOM    473  CE1 PHE    36       5.844   1.521  29.107
1.00134.48           C
ATOM    474  CE2 PHE    36       7.152   1.038  27.124
1.00134.27           C
ATOM    475  CZ  PHE    36       6.255   0.623  28.115
1.00149.48           C
ATOM    476  HN  PHE    36       7.765   4.759  30.683  1.00
62.87           H
```

```
ATOM    477  HA  PHE    36        9.708   3.998  28.630  1.00
28.53          H
ATOM    478  HB1 PHE    36        7.982   4.963  27.115  1.00
73.56          H
ATOM    479  HB2 PHE    36        6.994   5.325  28.523  1.00
88.42          H
ATOM    480  HD1 PHE    36        6.013   3.528  29.873
1.00110.20          H
ATOM    481  HD2 PHE    36        8.330   2.670  26.359  1.00
93.07          H
ATOM    482  HE1 PHE    36        5.150   1.201  29.871
1.00155.06          H
ATOM    483  HE2 PHE    36        7.469   0.344  26.359
1.00159.46          H
ATOM    484  HZ  PHE    36        5.880  -0.390  28.114
1.00183.25          H
ATOM    485  N   THR    37       10.706   6.216  28.070  1.00
20.06          N
ATOM    486  CA  THR    37       11.399   7.480  27.859  1.00
27.18          C
ATOM    487  C   THR    37       11.694   7.662  26.377  1.00
41.87          C
ATOM    488  O   THR    37       10.958   7.174  25.521
1.00169.86          O
ATOM    489  CB  THR    37       12.710   7.491  28.649  1.00
26.52          C
ATOM    490  CG2 THR    37       12.451   7.007  30.076  1.00
75.69          C
ATOM    491  OG1 THR    37       13.646   6.629  28.018  1.00
81.95          O
ATOM    492  HN  THR    37       11.046   5.417  27.625  1.00
18.47          H
ATOM    493  HA  THR    37       10.779   8.299  28.196  1.00
38.80          H
ATOM    494  HB  THR    37       13.104   8.494  28.678  1.00
81.60          H
ATOM    495  HG1 THR    37       13.454   6.621  27.078
1.00188.80          H
ATOM    496 HG21 THR    37       13.332   7.174  30.678
1.00196.98          H
ATOM    497 HG22 THR    37       12.219   5.953  30.062
1.00196.23          H
ATOM    498 HG23 THR    37       11.620   7.554  30.498
1.00168.92          H
ATOM    499  N   GLU    38       12.777   8.368  26.086  1.00
26.53          N
ATOM    500  CA  GLU    38       13.181   8.621  24.701  1.00
30.29          C
ATOM    501  C   GLU    38       14.702   8.630  24.574  1.00
25.39          C
ATOM    502  O   GLU    38       15.407   9.119  25.457  1.00
51.60          O
```

```
ATOM    503  CB  GLU    38      12.630   9.970  24.229  1.00
54.82        C
ATOM    504  CG  GLU    38      11.102   9.912  24.157
1.00165.84         C
ATOM    505  CD  GLU    38      10.555  11.242  23.650
1.00292.92         C
ATOM    506  OE1 GLU    38      11.342  12.160  23.485
1.00414.19         O
ATOM    507  OE2 GLU    38       9.356  11.324  23.436
1.00537.65         O1-
ATOM    508  HN  GLU    38      13.318   8.720  26.817  1.00
84.50        H
ATOM    509  HA  GLU    38      12.784   7.843  24.063  1.00
32.15        H
ATOM    510  HB1 GLU    38      13.025  10.198  23.252
1.00144.82         H
ATOM    511  HB2 GLU    38      12.927  10.741  24.926
1.00106.49         H
ATOM    512  HG1 GLU    38      10.704   9.715  25.140
1.00303.31         H
ATOM    513  HG2 GLU    38      10.804   9.121  23.484
1.00323.99         H
ATOM    514  N   CYS    39      15.199   8.098  23.460  1.00
15.84        N
ATOM    515  CA  CYS    39      16.635   8.060  23.214  1.00
14.30        C
ATOM    516  C   CYS    39      17.170   9.488  23.071  1.00
22.31        C
ATOM    517  O   CYS    39      16.441  10.379  22.636  1.00
32.92        O
ATOM    518  CB  CYS    39      16.911   7.267  21.934  1.00
18.08        C
ATOM    519  SG  CYS    39      15.772   7.810  20.636  1.00
17.41        S
ATOM    520  HN  CYS    39      14.589   7.733  22.789  1.00
24.87        H
ATOM    521  HA  CYS    39      17.118   7.567  24.045  1.00
14.60        H
ATOM    522  HB1 CYS    39      16.768   6.215  22.124  1.00
27.71        H
ATOM    523  HB2 CYS    39      17.927   7.441  21.615  1.00
34.91        H
ATOM    524  N   PRO    40      18.409   9.735  23.426  1.00
27.27        N
ATOM    525  CA  PRO    40      19.003  11.100  23.325  1.00
47.66        C
ATOM    526  C   PRO    40      19.290  11.494  21.879  1.00
71.54        C
ATOM    527  O   PRO    40      19.910  10.741  21.126  1.00
87.70        O
ATOM    528  CB  PRO    40      20.294  10.992  24.145  1.00
53.85        C
```

```
ATOM    529  CG  PRO    40       20.689   9.554  24.038  1.00
46.04           C
ATOM    530  CD  PRO    40       19.380   8.761  23.957  1.00
25.70           C
ATOM    531  HA  PRO    40       18.346  11.825  23.781  1.00
58.04           H
ATOM    532  HB1 PRO    40       20.106  11.248  25.177  1.00
67.93           H
ATOM    533  HB2 PRO    40       21.064  11.634  23.732  1.00
70.99           H
ATOM    534  HG1 PRO    40       21.247   9.250  24.911  1.00
65.91           H
ATOM    535  HG2 PRO    40       21.281   9.398  23.144  1.00
54.99           H
ATOM    536  HD1 PRO    40       19.073   8.426  24.935  1.00
24.22           H
ATOM    537  HD2 PRO    40       19.485   7.925  23.281  1.00
26.94           H
ATOM    538  N   GLY    41       18.838  12.680  21.508
1.00105.24           N
ATOM    539  CA  GLY    41       19.046  13.186  20.156
1.00147.55           C
ATOM    540  C   GLY    41       20.525  13.428  19.886
1.00161.74           C
ATOM    541  O   GLY    41       21.024  13.143  18.798
1.00244.37           O
ATOM    542  HN  GLY    41       18.358  13.230  22.159
1.00121.79           H
ATOM    543  HA1 GLY    41       18.510  14.116  20.039
1.00184.40           H
ATOM    544  HA2 GLY    41       18.669  12.467  19.448
1.00157.34           H
ATOM    545  N   LEU    42       21.219  13.964  20.888
1.00162.48           N
ATOM    546  CA  LEU    42       22.649  14.261  20.771
1.00191.48           C
ATOM    547  C   LEU    42       23.449  13.467  21.801
1.00166.66           C
ATOM    548  O   LEU    42       22.956  13.164  22.886
1.00251.38           O
ATOM    549  CB  LEU    42       22.887  15.758  20.994
1.00307.99           C
ATOM    550  CG  LEU    42       22.048  16.576  20.005
1.00452.28           C
ATOM    551  CD1 LEU    42       22.245  18.067  20.294
1.00681.24           C
ATOM    552  CD2 LEU    42       22.481  16.268  18.562
1.00527.46           C
ATOM    553  HN  LEU    42       20.756  14.170  21.727
1.00195.47           H
ATOM    554  HA  LEU    42       23.000  13.996  19.783
1.00229.77           H
```

```
ATOM    555  HB1 LEU    42       23.933  15.981  20.844
1.00342.49           H
ATOM    556  HB2 LEU    42       22.606  16.017  22.005
1.00335.33           H
ATOM    557  HG  LEU    42       21.003  16.325  20.130
1.00427.79           H
ATOM    558 HD11 LEU    42       21.851  18.649  19.474
1.00909.71           H
ATOM    559 HD12 LEU    42       23.299  18.275  20.407
1.00706.80           H
ATOM    560 HD13 LEU    42       21.727  18.329  21.204
1.00858.82           H
ATOM    561 HD21 LEU    42       22.206  17.090  17.915
1.00631.69           H
ATOM    562 HD22 LEU    42       21.988  15.370  18.221
1.00628.06           H
ATOM    563 HD23 LEU    42       23.552  16.127  18.524
1.00656.75           H
ATOM    564  N   THR    43       24.687  13.137  21.446
1.00178.52           N
ATOM    565  CA  THR    43       25.557  12.380  22.338
1.00240.84           C
ATOM    566  C   THR    43       25.957  13.245  23.540
1.00372.31           C
ATOM    567  O   THR    43       26.035  14.468  23.419
1.00500.13           O
ATOM    568  CB  THR    43       26.810  11.951  21.568
1.00354.13           C
ATOM    569  CG2 THR    43       26.398  11.191  20.308
1.00463.04           C
ATOM    570  OG1 THR    43       27.556  13.103  21.203
1.00508.50           O
ATOM    571  HN  THR    43       25.022  13.409  20.566
1.00229.59           H
ATOM    572  HA  THR    43       25.032  11.503  22.678
1.00249.90           H
ATOM    573  HB  THR    43       27.418  11.311  22.183
1.00481.58           H
ATOM    574  HG1 THR    43       27.324  13.332  20.300
1.00624.31           H
ATOM    575 HG21 THR    43       27.280  10.863  19.780
1.00650.95           H
ATOM    576 HG22 THR    43       25.817  11.840  19.669
1.00580.73           H
ATOM    577 HG23 THR    43       25.804  10.332  20.583
1.00571.99           H
ATOM    578  N   PRO    44       26.221  12.654  24.686
1.00488.39           N
ATOM    579  CA  PRO    44       26.627  13.423  25.900
1.00760.13           C
ATOM    580  C   PRO    44       28.064  13.926  25.791
1.00735.36           C
```

```
ATOM    581  O   PRO    44       28.537  14.681  26.641
1.00999.99           O
ATOM    582  CB  PRO    44       26.468  12.407  27.036
1.00999.99           C
ATOM    583  CG  PRO    44       26.708  11.082  26.393
1.00856.94           C
ATOM    584  CD  PRO    44       26.167  11.201  24.963
1.00548.09           C
ATOM    585  HA  PRO    44       25.955  14.252  26.057
1.00939.00           H
ATOM    586  HB1 PRO    44       25.467  12.447  27.439
1.00999.99           H
ATOM    587  HB2 PRO    44       27.196  12.591  27.818
1.00999.99           H
ATOM    588  HG1 PRO    44       26.177  10.303  26.920
1.00999.99           H
ATOM    589  HG2 PRO    44       27.770  10.864  26.376
1.00853.53           H
ATOM    590  HD1 PRO    44       25.148  10.850  24.909
1.00602.45           H
ATOM    591  HD2 PRO    44       26.797  10.654  24.278
1.00463.32           H
ATOM    592  N   ILE    45       28.753  13.496  24.738
1.00518.33           N
ATOM    593  CA  ILE    45       30.135  13.902  24.524
1.00582.79           C
ATOM    594  C   ILE    45       30.230  15.420  24.391
1.00795.93           C
ATOM    595  O   ILE    45       29.509  16.030  23.602
1.00898.75           O
ATOM    596  CB  ILE    45       30.681  13.250  23.250
1.00474.41           C
ATOM    597  CG1 ILE    45       30.499  11.724  23.322
1.00516.90           C
ATOM    598  CG2 ILE    45       32.164  13.591  23.086
1.00760.75           C
ATOM    599  CD1 ILE    45       31.163  11.152  24.582
1.00566.81           C
ATOM    600  HN  ILE    45       28.324  12.893  24.096
1.00395.72           H
ATOM    601  HA  ILE    45       30.730  13.585  25.365
1.00711.06           H
ATOM    602  HB  ILE    45       30.136  13.633  22.399
1.00477.81           H
ATOM    603 HG11 ILE    45       30.947  11.272  22.448
1.00624.28           H
ATOM    604 HG12 ILE    45       29.445  11.493  23.341
1.00744.84           H
ATOM    605 HG21 ILE    45       32.669  13.466  24.032
1.00920.57           H
ATOM    606 HG22 ILE    45       32.263  14.616  22.758
1.00999.99           H
```

```
ATOM    607 HG23 ILE    45       32.606  12.934  22.351
1.00865.46           H
ATOM    608 HD11 ILE    45       30.490  11.259  25.421
1.00658.89           H
ATOM    609 HD12 ILE    45       32.081  11.680  24.788
1.00734.30           H
ATOM    610 HD13 ILE    45       31.378  10.106  24.429
1.00675.84           H
ATOM    611  N   ALA    46       31.125  16.021  25.167
1.00999.99           N
ATOM    612  CA  ALA    46       31.307  17.467  25.129
1.00999.99           C
ATOM    613  C   ALA    46       32.053  17.880  23.864
1.00999.99           C
ATOM    614  CB  ALA    46       32.090  17.926  26.359
1.00999.99           C
ATOM    615  OT1 ALA    46       31.833  17.255  22.840
1.00999.99           O
ATOM    616  OT2 ALA    46       32.831  18.816  23.940
1.00999.99           O
ATOM    617  HN  ALA    46       31.673  15.483  25.776
1.00999.99           H
ATOM    618  HA  ALA    46       30.338  17.942  25.136
1.00999.99           H
ATOM    619  HB1 ALA    46       31.579  17.599  27.253
1.00999.99           H
ATOM    620  HB2 ALA    46       32.163  19.003  26.359
1.00999.99           H
ATOM    621  HB3 ALA    46       33.082  17.498  26.336
1.00999.99           H
ENDMDL
TER
MODEL        8
ATOM      1  N   GLY     1       26.106  -2.123  20.562
1.00999.99           N
ATOM      2  CA  GLY     1       25.055  -2.779  19.732
1.00999.99           C
ATOM      3  C   GLY     1       24.440  -1.755  18.786
1.00999.99           C
ATOM      4  O   GLY     1       23.801  -0.797  19.222
1.00999.99           O
ATOM      5  HA1 GLY     1       24.286  -3.179  20.375
1.00999.99           H
ATOM      6  HA2 GLY     1       25.498  -3.580  19.157
1.00999.99           H
ATOM      7  HT1 GLY     1       26.529  -1.338  20.029
1.00999.99           H
ATOM      8  HT2 GLY     1       26.844  -2.819  20.800
1.00999.99           H
ATOM      9  HT3 GLY     1       25.679  -1.757  21.435
1.00999.99           H
ATOM     10  N   LEU     2       24.636  -1.963  17.488
```

```
1.00999.99           N
ATOM     11  CA  LEU     2       24.094  -1.049  16.489
1.00895.59           C
ATOM     12  C   LEU     2       22.574  -1.161  16.441
1.00532.27           C
ATOM     13  O   LEU     2       22.014  -2.230  16.681
1.00625.21           O
ATOM     14  CB  LEU     2       24.675  -1.379  15.111
1.00999.99           C
ATOM     15  CG  LEU     2       26.207  -1.296  15.154
1.00999.99           C
ATOM     16  CD1 LEU     2       26.770  -1.710  13.791
1.00999.99           C
ATOM     17  CD2 LEU     2       26.650   0.143  15.481
1.00999.99           C
ATOM     18  HN  LEU     2       25.152  -2.743  17.198
1.00999.99           H
ATOM     19  HA  LEU     2       24.360  -0.039  16.752
1.00890.87           H
ATOM     20  HB1 LEU     2       24.299  -0.673  14.386
1.00924.07           H
ATOM     21  HB2 LEU     2       24.378  -2.379  14.827
1.00999.99           H
ATOM     22  HG  LEU     2       26.579  -1.968  15.913
1.00999.99           H
ATOM     23 HD11 LEU     2       27.845  -1.799  13.857
1.00999.99           H
ATOM     24 HD12 LEU     2       26.516  -0.964  13.053
1.00999.99           H
ATOM     25 HD13 LEU     2       26.347  -2.661  13.501
1.00999.99           H
ATOM     26 HD21 LEU     2       26.639   0.287  16.551
1.00999.99           H
ATOM     27 HD22 LEU     2       25.975   0.848  15.015
1.00999.99           H
ATOM     28 HD23 LEU     2       27.651   0.309  15.110
1.00999.99           H
ATOM     29  N   CYS     3       21.911  -0.050  16.135
1.00271.28           N
ATOM     30  CA  CYS     3       20.453  -0.036  16.066
1.00104.50           C
ATOM     31  C   CYS     3       19.974  -0.574  14.721  1.00
90.98           C
ATOM     32  O   CYS     3       20.199   0.042  13.679
1.00207.28           O
ATOM     33  CB  CYS     3       19.932   1.391  16.250  1.00
37.83           C
ATOM     34  SG  CYS     3       20.461   2.028  17.860  1.00
81.56           S
ATOM     35  HN  CYS     3       22.409   0.777  15.957
1.00296.55           H
ATOM     36  HA  CYS     3       20.056  -0.657  16.855
```

```
1.00166.45           H
ATOM     37  HB1 CYS     3       18.854   1.388  16.201  1.00
40.38           H
ATOM     38  HB2 CYS     3       20.326   2.022  15.468  1.00
98.45           H
ATOM     39  N   SER     4       19.302  -1.720  14.754  1.00
85.31           N
ATOM     40  CA  SER     4       18.784  -2.326  13.532  1.00
92.94           C
ATOM     41  C   SER     4       17.772  -1.396  12.890  1.00
67.37           C
ATOM     42  O   SER     4       17.745  -1.218  11.673
1.00102.28           O
ATOM     43  CB  SER     4       18.084  -3.642  13.855
1.00123.83           C
ATOM     44  OG  SER     4       17.707  -4.281  12.642
1.00178.89           O
ATOM     45  HN  SER     4       19.145  -2.162  15.614
1.00163.03           H
ATOM     46  HA  SER     4       19.596  -2.509  12.845
1.00129.78           H
ATOM     47  HB1 SER     4       17.199  -3.432  14.448
1.00105.26           H
ATOM     48  HB2 SER     4       18.746  -4.285  14.411
1.00156.87           H
ATOM     49  HG  SER     4       16.885  -3.887  12.341
1.00218.16           H
ATOM     50  N   GLU     5       16.923  -0.824  13.737  1.00
41.67           N
ATOM     51  CA  GLU     5       15.871   0.078  13.290  1.00
41.21           C
ATOM     52  C   GLU     5       15.693   1.218  14.287  1.00
41.92           C
ATOM     53  O   GLU     5       16.563   1.460  15.125  1.00
74.72           O
ATOM     54  CB  GLU     5       14.568  -0.702  13.150  1.00
49.24           C
ATOM     55  CG  GLU     5       14.237  -1.365  14.481  1.00
55.48           C
ATOM     56  CD  GLU     5       13.081  -2.345  14.307  1.00
92.06           C
ATOM     57  OE1 GLU     5       12.705  -2.967  15.286
1.00206.49           O
ATOM     58  OE2 GLU     5       12.590  -2.460  13.194
1.00197.24           O1-
ATOM     59  HN  GLU     5       16.997  -1.028  14.691  1.00
45.52           H
ATOM     60  HA  GLU     5       16.133   0.487  12.334  1.00
60.63           H
ATOM     61  HB1 GLU     5       14.679  -1.461  12.390  1.00
61.59           H
ATOM     62  HB2 GLU     5       13.774  -0.031  12.876  1.00
```

```
63.66          H
ATOM     63  HG1 GLU     5      13.959  -0.609  15.199  1.00
58.03          H
ATOM     64  HG2 GLU     5      15.107  -1.890  14.832  1.00
57.77          H
ATOM     65  N   ASN     6      14.568   1.917  14.197  1.00
50.73          N
ATOM     66  CA  ASN     6      14.303   3.029  15.102  1.00
71.04          C
ATOM     67  C   ASN     6      14.282   2.544  16.548  1.00
65.17          C
ATOM     68  O   ASN     6      14.785   3.218  17.447  1.00
94.86          O
ATOM     69  CB  ASN     6      12.961   3.675  14.759  1.00
94.83          C
ATOM     70  CG  ASN     6      13.072   4.444  13.446
1.00160.94          C
ATOM     71  ND2 ASN     6      11.990   4.730  12.774
1.00244.88          N
ATOM     72  OD1 ASN     6      14.175   4.794  13.024
1.00219.73          O
ATOM     73  HN  ASN     6      13.909   1.684  13.510  1.00
72.81          H
ATOM     74  HA  ASN     6      15.085   3.766  14.991  1.00
96.02          H
ATOM     75  HB1 ASN     6      12.678   4.357  15.548
1.00111.09          H
ATOM     76  HB2 ASN     6      12.207   2.907  14.662  1.00
98.13          H
ATOM     77 HD21 ASN     6      11.115   4.451  13.112
1.00272.43          H
ATOM     78 HD22 ASN     6      12.055   5.223  11.930
1.00336.78          H
ATOM     79  N   GLY     7      13.699   1.365  16.763  1.00
51.80          N
ATOM     80  CA  GLY     7      13.614   0.779  18.105  1.00
71.47          C
ATOM     81  C   GLY     7      14.209  -0.622  18.121  1.00
41.04          C
ATOM     82  O   GLY     7      13.501  -1.607  18.333  1.00
44.54          O
ATOM     83  HN  GLY     7      13.319   0.875  16.005  1.00
45.49          H
ATOM     84  HA1 GLY     7      12.581   0.722  18.400
1.00103.52          H
ATOM     85  HA2 GLY     7      14.150   1.399  18.812
1.00108.67          H
ATOM     86  N   ASP     8      15.511  -0.700  17.895  1.00
27.00          N
ATOM     87  CA  ASP     8      16.201  -1.986  17.883  1.00
14.15          C
ATOM     88  C   ASP     8      16.082  -2.671  19.239  1.00
```

```
9.37           C
ATOM     89  O   ASP     8       15.853  -3.878  19.319  1.00
14.61           O
ATOM     90  CB  ASP     8       17.675  -1.794  17.532  1.00
15.06           C
ATOM     91  CG  ASP     8       18.359  -3.150  17.391  1.00
21.22           C
ATOM     92  OD1 ASP     8       17.733  -4.144  17.719
1.00119.56           O
ATOM     93  OD2 ASP     8       19.500  -3.174  16.959
1.00133.27           O1-
ATOM     94  HN  ASP     8       16.017   0.121  17.733  1.00
35.63           H
ATOM     95  HA  ASP     8       15.745  -2.616  17.137  1.00
20.92           H
ATOM     96  HB1 ASP     8       18.158  -1.232  18.316  1.00
29.39           H
ATOM     97  HB2 ASP     8       17.756  -1.252  16.601  1.00
44.14           H
ATOM     98  N   CYS     9       16.235  -1.893  20.303  1.00
5.93           N
ATOM     99  CA  CYS     9       16.136  -2.438  21.651  1.00
7.54           C
ATOM    100  C   CYS     9       14.748  -3.024  21.873  1.00
14.60           C
ATOM    101  O   CYS     9       14.595  -4.107  22.440  1.00
25.78           O
ATOM    102  CB  CYS     9       16.399  -1.346  22.677  1.00
6.63           C
ATOM    103  SG  CYS     9       18.144  -0.903  22.610  1.00
10.91           S
ATOM    104  HN  CYS     9       16.411  -0.937  20.177  1.00
6.51           H
ATOM    105  HA  CYS     9       16.881  -3.206  21.773  1.00
11.12           H
ATOM    106  HB1 CYS     9       16.161  -1.712  23.664  1.00
11.28           H
ATOM    107  HB2 CYS     9       15.794  -0.481  22.453  1.00
5.04           H
ATOM    108  N   ALA    10       13.742  -2.295  21.405  1.00
16.26           N
ATOM    109  CA  ALA    10       12.355  -2.725  21.525  1.00
32.04           C
ATOM    110  C   ALA    10       11.467  -1.861  20.629  1.00
45.47           C
ATOM    111  O   ALA    10       11.885  -0.798  20.173
1.00119.04           O
ATOM    112  CB  ALA    10       11.889  -2.635  22.989  1.00
30.10           C
ATOM    113  HN  ALA    10       13.941  -1.447  20.956  1.00
12.81           H
ATOM    114  HA  ALA    10       12.282  -3.753  21.197  1.00
```

```
47.14           H
ATOM    115  HB1 ALA    10       12.728  -2.823  23.642  1.00
84.37           H
ATOM    116  HB2 ALA    10       11.121  -3.373  23.170
1.00118.20           H
ATOM    117  HB3 ALA    10       11.492  -1.651  23.189
1.00117.32           H
ATOM    118  N   ALA    11       10.250  -2.325  20.374  1.00
30.62           N
ATOM    119  CA  ALA    11        9.326  -1.584  19.522  1.00
35.24           C
ATOM    120  C   ALA    11        9.016  -0.207  20.111  1.00
22.78           C
ATOM    121  O   ALA    11        8.939   0.783  19.383  1.00
53.33           O
ATOM    122  CB  ALA    11        8.027  -2.372  19.356  1.00
60.49           C
ATOM    123  HN  ALA    11        9.969  -3.181  20.759  1.00
50.37           H
ATOM    124  HA  ALA    11        9.778  -1.453  18.550  1.00
41.70           H
ATOM    125  HB1 ALA    11        7.316  -1.782  18.796
1.00157.06           H
ATOM    126  HB2 ALA    11        7.616  -2.600  20.329
1.00148.83           H
ATOM    127  HB3 ALA    11        8.228  -3.290  18.827
1.00137.06           H
ATOM    128  N   ASP    12        8.828  -0.152  21.427  1.00
18.98           N
ATOM    129  CA  ASP    12        8.514   1.111  22.097  1.00
31.05           C
ATOM    130  C   ASP    12        9.783   1.880  22.461  1.00
22.98           C
ATOM    131  O   ASP    12        9.775   3.110  22.523  1.00
37.14           O
ATOM    132  CB  ASP    12        7.709   0.833  23.369  1.00
48.35           C
ATOM    133  CG  ASP    12        6.329   0.299  23.004
1.00104.83           C
ATOM    134  OD1 ASP    12        5.678  -0.252  23.878
1.00258.73           O
ATOM    135  OD2 ASP    12        5.942   0.449  21.857
1.00213.46           O1-
ATOM    136  HN  ASP    12        8.893  -0.975  21.957  1.00
36.19           H
ATOM    137  HA  ASP    12        7.913   1.719  21.438  1.00
49.76           H
ATOM    138  HB1 ASP    12        7.602   1.746  23.933  1.00
95.71           H
ATOM    139  HB2 ASP    12        8.229   0.100  23.970  1.00
54.37           H
ATOM    140  N   GLU    13       10.866   1.155  22.709  1.00
```

```
17.60           N
ATOM    141  CA  GLU    13       12.131   1.784  23.077  1.00
12.01           C
ATOM    142  C   GLU    13       12.790   2.440  21.865  1.00
10.07           C
ATOM    143  O   GLU    13       12.484   2.100  20.722  1.00
12.81           O
ATOM    144  CB  GLU    13       13.068   0.741  23.680  1.00
11.74           C
ATOM    145  CG  GLU    13       12.469   0.236  24.996  1.00
13.49           C
ATOM    146  CD  GLU    13       13.201  -1.017  25.464
1.00142.36           C
ATOM    147  OE1 GLU    13       12.770  -1.598  26.446
1.00339.06           O
ATOM    148  OE2 GLU    13       14.179  -1.377  24.835
1.00335.28           O1-
ATOM    149  HN  GLU    13       10.814   0.178  22.651  1.00
28.36           H
ATOM    150  HA  GLU    13       11.939   2.544  23.820  1.00
13.06           H
ATOM    151  HB1 GLU    13       14.031   1.188  23.874  1.00
13.76           H
ATOM    152  HB2 GLU    13       13.187  -0.082  22.991  1.00
9.94           H
ATOM    153  HG1 GLU    13       11.424   0.003  24.848  1.00
66.02           H
ATOM    154  HG2 GLU    13       12.561   1.005  25.749  1.00
56.23           H
ATOM    155  N   CYS    14       13.691   3.387  22.127  1.00
9.12           N
ATOM    156  CA  CYS    14       14.395   4.100  21.057  1.00
10.69           C
ATOM    157  C   CYS    14       15.810   3.563  20.908  1.00
9.56           C
ATOM    158  O   CYS    14       16.411   3.112  21.879  1.00
13.62           O
ATOM    159  CB  CYS    14       14.461   5.593  21.381  1.00
13.96           C
ATOM    160  SG  CYS    14       15.308   6.465  20.036  1.00
42.96           S
ATOM    161  HN  CYS    14       13.893   3.613  23.060  1.00
9.76           H
ATOM    162  HA  CYS    14       13.867   3.968  20.123  1.00
15.54           H
ATOM    163  HB1 CYS    14       15.008   5.734  22.301  1.00
50.72           H
ATOM    164  HB2 CYS    14       13.460   5.983  21.491  1.00
46.13           H
ATOM    165  N   CYS    15       16.339   3.627  19.691  1.00
9.60           N
ATOM    166  CA  CYS    15       17.694   3.158  19.427  1.00
```

```
9.28            C
ATOM    167  C   CYS    15      18.292   3.976  18.296  1.00
9.92            C
ATOM    168  O   CYS    15      17.712   4.072  17.215  1.00
13.31           O
ATOM    169  CB  CYS    15      17.681   1.674  19.043  1.00
12.90           C
ATOM    170  SG  CYS    15      19.354   0.995  19.197  1.00
39.11           S
ATOM    171  HN  CYS    15      15.815   4.010  18.957  1.00
13.12           H
ATOM    172  HA  CYS    15      18.300   3.287  20.314  1.00
8.26            H
ATOM    173  HB1 CYS    15      17.342   1.569  18.023  1.00
24.82           H
ATOM    174  HB2 CYS    15      17.012   1.139  19.700  1.00
27.31           H
ATOM    175  N   VAL    16      19.448   4.577  18.551  1.00
9.56            N
ATOM    176  CA  VAL    16      20.106   5.400  17.542  1.00
11.99           C
ATOM    177  C   VAL    16      21.619   5.280  17.663  1.00
7.73            C
ATOM    178  O   VAL    16      22.177   5.380  18.755  1.00
8.83            O
ATOM    179  CB  VAL    16      19.672   6.855  17.719  1.00
18.37           C
ATOM    180  CG1 VAL    16      20.055   7.341  19.119  1.00
39.61           C
ATOM    181  CG2 VAL    16      20.359   7.721  16.668
1.00115.21           C
ATOM    182  HN  VAL    16      19.864   4.473  19.434  1.00
9.76            H
ATOM    183  HA  VAL    16      19.810   5.067  16.557  1.00
17.27           H
ATOM    184  HB  VAL    16      18.600   6.924  17.600  1.00
52.63           H
ATOM    185 HG11 VAL    16      21.129   7.438  19.188
1.00128.07           H
ATOM    186 HG12 VAL    16      19.709   6.630  19.856
1.00154.13           H
ATOM    187 HG13 VAL    16      19.595   8.301  19.305
1.00135.74           H
ATOM    188 HG21 VAL    16      19.947   8.719  16.699
1.00229.05           H
ATOM    189 HG22 VAL    16      20.194   7.295  15.689
1.00261.82           H
ATOM    190 HG23 VAL    16      21.418   7.763  16.870
1.00210.07           H
ATOM    191  N   ASP    17      22.278   5.053  16.528  1.00
14.34           N
ATOM    192  CA  ASP    17      23.733   4.910  16.506  1.00
```

```
11.90           C
ATOM    193  C   ASP    17       24.401   6.236  16.159  1.00
13.15           C
ATOM    194  O   ASP    17       24.165   6.801  15.093  1.00
25.96           O
ATOM    195  CB  ASP    17       24.131   3.858  15.470  1.00
20.80           C
ATOM    196  CG  ASP    17       23.724   2.469  15.953  1.00
28.72           C
ATOM    197  OD1 ASP    17       23.419   2.337  17.127
1.00135.42           O
ATOM    198  OD2 ASP    17       23.722   1.559  15.140
1.00114.17           O1-
ATOM    199  HN  ASP    17       21.777   4.976  15.690  1.00
28.29           H
ATOM    200  HA  ASP    17       24.077   4.586  17.478  1.00
10.49           H
ATOM    201  HB1 ASP    17       25.201   3.885  15.325  1.00
22.42           H
ATOM    202  HB2 ASP    17       23.636   4.071  14.535  1.00
29.52           H
ATOM    203  N   THR    18       25.248   6.716  17.066  1.00
11.55           N
ATOM    204  CA  THR    18       25.971   7.972  16.857  1.00
17.10           C
ATOM    205  C   THR    18       27.424   7.683  16.510  1.00
10.63           C
ATOM    206  O   THR    18       27.905   6.572  16.714  1.00
6.49           O
ATOM    207  CB  THR    18       25.921   8.829  18.123  1.00
28.52           C
ATOM    208  CG2 THR    18       24.495   9.316  18.373  1.00
45.38           C
ATOM    209  OG1 THR    18       26.364   8.058  19.228  1.00
26.49           O
ATOM    210  HN  THR    18       25.399   6.211  17.893  1.00
13.96           H
ATOM    211  HA  THR    18       25.516   8.524  16.044  1.00
26.43           H
ATOM    212  HB  THR    18       26.570   9.683  18.002  1.00
37.43           H
ATOM    213  HG1 THR    18       25.730   8.171  19.940  1.00
71.62           H
ATOM    214 HG21 THR    18       24.398   9.627  19.402
1.00100.44           H
ATOM    215 HG22 THR    18       23.797   8.516  18.170
1.00129.42           H
ATOM    216 HG23 THR    18       24.285  10.152  17.724
1.00156.21           H
ATOM    217  N   VAL    19       28.113   8.699  16.004  1.00
16.32           N
ATOM    218  CA  VAL    19       29.506   8.577  15.644  1.00
```

13.34   C
ATOM   219 C   VAL   19   29.973   9.872 15.013 1.00
25.88   C
ATOM   220 O   VAL   19   29.468 10.308 13.978 1.00
42.11   O
ATOM   221 CB VAL   19   29.752   7.417 14.681 1.00
15.48   C
ATOM   222 CG1 VAL   19   28.784   7.497 13.496 1.00
29.06   C
ATOM   223 CG2 VAL   19   31.198   7.493 14.169 1.00
20.67   C
ATOM   224 HN VAL   19   27.681   9.566 15.889 1.00
26.89   H
ATOM   225 HA VAL   19   30.078   8.406 16.544 1.00
8.08   H
ATOM   226 HB VAL   19   29.610   6.488 15.204 1.00
11.49   H
ATOM   227 HG11 VAL   19   27.784   7.690 13.858
1.00137.43   H
ATOM   228 HG12 VAL   19   28.795   6.560 12.958 1.00
93.29   H
ATOM   229 HG13 VAL   19   29.087   8.294 12.833
1.00108.69   H
ATOM   230 HG21 VAL   19   31.480   6.547 13.733 1.00
99.42   H
ATOM   231 HG22 VAL   19   31.860   7.726 14.994
1.00111.36   H
ATOM   232 HG23 VAL   19   31.274   8.273 13.423 1.00
77.11   H
ATOM   233 N   PHE   20   30.932 10.473 15.666 1.00
24.58   N
ATOM   234 CA PHE   20   31.503 11.734 15.226 1.00
40.34   C
ATOM   235 C   PHE   20   32.798 11.503 14.455 1.00
40.37   C
ATOM   236 O   PHE   20   32.896 11.848 13.278 1.00
66.02   O
ATOM   237 CB PHE   20   31.767 12.634 16.450 1.00
48.79   C
ATOM   238 CG PHE   20   32.031 11.799 17.697 1.00
35.75   C
ATOM   239 CD1 PHE   20   31.015 10.997 18.255 1.00
30.20   C
ATOM   240 CD2 PHE   20   33.291 11.844 18.309 1.00
40.95   C
ATOM   241 CE1 PHE   20   31.270 10.249 19.408 1.00
30.41   C
ATOM   242 CE2 PHE   20   33.541 11.091 19.462 1.00
45.84   C
ATOM   243 CZ PHE   20   32.531 10.294 20.011 1.00
40.81   C
ATOM   244 HN PHE   20   31.256 10.060 16.484 1.00

```
16.73           H
ATOM    245  HA  PHE    20       30.802  12.236  14.572  1.00
59.91           H
ATOM    246  HB1 PHE    20       30.909  13.242  16.618  1.00
67.15           H
ATOM    247  HB2 PHE    20       32.616  13.276  16.261  1.00
58.17           H
ATOM    248  HD1 PHE    20       30.036  10.948  17.795  1.00
33.34           H
ATOM    249  HD2 PHE    20       34.074  12.456  17.889  1.00
49.35           H
ATOM    250  HE1 PHE    20       30.491   9.636  19.831  1.00
30.61           H
ATOM    251  HE2 PHE    20       34.514  11.125  19.929  1.00
61.27           H
ATOM    252  HZ  PHE    20       32.724   9.715  20.902  1.00
52.66           H
ATOM    253  N   GLU    21       33.798  10.950  15.134  1.00
41.31           N
ATOM    254  CA  GLU    21       35.089  10.714  14.527  1.00
56.16           C
ATOM    255  C   GLU    21       35.366   9.225  14.369  1.00
58.61           C
ATOM    256  O   GLU    21       34.495   8.388  14.606
1.00201.19           O
ATOM    257  CB  GLU    21       36.123  11.349  15.428  1.00
57.37           C
ATOM    258  CG  GLU    21       36.150  10.617  16.765
1.00193.56           C
ATOM    259  CD  GLU    21       36.983  11.401  17.774
1.00304.62           C
ATOM    260  OE1 GLU    21       37.593  12.379  17.375
1.00451.81           O
ATOM    261  OE2 GLU    21       36.996  11.015  18.930
1.00442.88           O1-
ATOM    262  HN  GLU    21       33.682  10.723  16.075  1.00
50.82           H
ATOM    263  HA  GLU    21       35.137  11.188  13.556  1.00
85.72           H
ATOM    264  HB1 GLU    21       35.851  12.380  15.595  1.00
46.37           H
ATOM    265  HB2 GLU    21       37.084  11.295  14.963
1.00131.81           H
ATOM    266  HG1 GLU    21       36.579   9.641  16.626
1.00314.91           H
ATOM    267  HG2 GLU    21       35.143  10.508  17.135
1.00266.92           H
ATOM    268  N   GLY    22       36.587   8.908  13.953  1.00
86.70           N
ATOM    269  CA  GLY    22       36.989   7.514  13.742
1.00104.66           C
ATOM    270  C   GLY    22       37.678   6.927  14.972  1.00
```

```
71.50           C
ATOM    271  O   GLY    22       37.891   5.717  15.052  1.00
89.87           O
ATOM    272  HN  GLY    22       37.227   9.630  13.777
1.00214.64           H
ATOM    273  HA1 GLY    22       37.674   7.472  12.907
1.00152.47           H
ATOM    274  HA2 GLY    22       36.117   6.919  13.509
1.00118.15           H
ATOM    275  N   ASP    23       38.033   7.783  15.923  1.00
44.68           N
ATOM    276  CA  ASP    23       38.706   7.323  17.134  1.00
43.33           C
ATOM    277  C   ASP    23       37.827   6.350  17.913  1.00
37.55           C
ATOM    278  O   ASP    23       38.313   5.362  18.460  1.00
61.37           O
ATOM    279  CB  ASP    23       39.056   8.513  18.031  1.00
40.74           C
ATOM    280  CG  ASP    23       40.172   9.335  17.397
1.00141.86           C
ATOM    281  OD1 ASP    23       40.791   8.841  16.468
1.00328.18           O
ATOM    282  OD2 ASP    23       40.392  10.448  17.848
1.00304.58           O1-
ATOM    283  HN  ASP    23       37.849   8.738  15.806  1.00
40.86           H
ATOM    284  HA  ASP    23       39.615   6.821  16.856  1.00
72.12           H
ATOM    285  HB1 ASP    23       39.385   8.150  18.993  1.00
94.15           H
ATOM    286  HB2 ASP    23       38.181   9.131  18.164  1.00
93.26           H
ATOM    287  N   MET    24       36.531   6.641  17.963  1.00
23.24           N
ATOM    288  CA  MET    24       35.594   5.785  18.685  1.00
32.50           C
ATOM    289  C   MET    24       34.170   5.978  18.174  1.00
25.13           C
ATOM    290  O   MET    24       33.886   6.917  17.432  1.00
54.40           O
ATOM    291  CB  MET    24       35.651   6.102  20.183  1.00
47.36           C
ATOM    292  CG  MET    24       35.217   7.552  20.422
1.00151.73           C
ATOM    293  SD  MET    24       35.420   7.969  22.175
1.00209.23           S
ATOM    294  CE  MET    24       37.217   8.208  22.178
1.00243.29           C
ATOM    295  HN  MET    24       36.203   7.445  17.511  1.00
17.32           H
ATOM    296  HA  MET    24       35.877   4.753  18.540  1.00
```

```
48.94           H
ATOM    297  HB1 MET    24       36.659   5.966  20.539
1.00124.40           H
ATOM    298  HB2 MET    24       34.988   5.436  20.716
1.00166.96           H
ATOM    299  HG1 MET    24       34.179   7.667  20.147
1.00331.71           H
ATOM    300  HG2 MET    24       35.821   8.213  19.820
1.00302.30           H
ATOM    301  HE1 MET    24       37.501   8.759  23.064
1.00340.44           H
ATOM    302  HE2 MET    24       37.512   8.769  21.304
1.00373.88           H
ATOM    303  HE3 MET    24       37.710   7.247  22.171
1.00386.81           H
ATOM    304  N   VAL    25       33.275   5.077  18.583  1.00
22.44           N
ATOM    305  CA  VAL    25       31.869   5.136  18.175  1.00
15.37           C
ATOM    306  C   VAL    25       30.960   5.172  19.398  1.00
17.47           C
ATOM    307  O   VAL    25       31.271   4.590  20.438  1.00
29.52           O
ATOM    308  CB  VAL    25       31.513   3.925  17.306  1.00
23.36           C
ATOM    309  CG1 VAL    25       30.000   3.922  17.019  1.00
58.39           C
ATOM    310  CG2 VAL    25       32.291   4.011  15.989  1.00
55.25           C
ATOM    311  HN  VAL    25       33.568   4.354  19.177  1.00
48.11           H
ATOM    312  HA  VAL    25       31.698   6.040  17.599  1.00
9.25           H
ATOM    313  HB  VAL    25       31.781   3.019  17.828  1.00
52.45           H
ATOM    314 HG11 VAL    25       29.801   3.369  16.113
1.00171.07           H
ATOM    315 HG12 VAL    25       29.648   4.938  16.904
1.00166.22           H
ATOM    316 HG13 VAL    25       29.482   3.457  17.843
1.00134.76           H
ATOM    317 HG21 VAL    25       33.351   4.000  16.194
1.00141.88           H
ATOM    318 HG22 VAL    25       32.033   4.926  15.476
1.00184.50           H
ATOM    319 HG23 VAL    25       32.037   3.166  15.365
1.00133.90           H
ATOM    320  N   THR    26       29.840   5.879  19.265  1.00
13.60           N
ATOM    321  CA  THR    26       28.878   6.027  20.356  1.00
21.19           C
ATOM    322  C   THR    26       27.479   5.594  19.922  1.00
```

```
16.55           C
ATOM    323  O   THR    26       27.063   5.839  18.790  1.00
11.43           O
ATOM    324  CB  THR    26       28.851   7.491  20.784  1.00
27.07           C
ATOM    325  CG2 THR    26       27.805   7.698  21.880  1.00
44.39           C
ATOM    326  OG1 THR    26       30.134   7.851  21.277  1.00
34.75           O
ATOM    327  HN  THR    26       29.665   6.328  18.411  1.00
10.81           H
ATOM    328  HA  THR    26       29.185   5.425  21.199  1.00
33.16           H
ATOM    329  HB  THR    26       28.608   8.104  19.925  1.00
20.60           H
ATOM    330  HG1 THR    26       30.198   7.547  22.185  1.00
77.72           H
ATOM    331 HG21 THR    26       27.906   8.693  22.288
1.00126.99           H
ATOM    332 HG22 THR    26       27.958   6.971  22.665
1.00108.32           H
ATOM    333 HG23 THR    26       26.816   7.580  21.468
1.00151.99           H
ATOM    334  N   ARG    27       26.755   4.947  20.838  1.00
22.38           N
ATOM    335  CA  ARG    27       25.395   4.479  20.557  1.00
20.21           C
ATOM    336  C   ARG    27       24.482   4.764  21.748  1.00
16.96           C
ATOM    337  O   ARG    27       24.948   4.876  22.881  1.00
20.06           O
ATOM    338  CB  ARG    27       25.415   2.974  20.271  1.00
22.14           C
ATOM    339  CG  ARG    27       25.853   2.215  21.527
1.00124.30           C
ATOM    340  CD  ARG    27       26.068   0.741  21.184
1.00109.38           C
ATOM    341  NE  ARG    27       26.423  -0.010  22.383
1.00227.73           N
ATOM    342  CZ  ARG    27       27.670  -0.020  22.846
1.00426.12           C
ATOM    343  NH1 ARG    27       27.960  -0.700  23.921
1.00767.09           N1+
ATOM    344  NH2 ARG    27       28.602   0.650  22.226
1.00581.78           N
ATOM    345  HN  ARG    27       27.142   4.784  21.722  1.00
30.75           H
ATOM    346  HA  ARG    27       25.005   4.994  19.688  1.00
23.13           H
ATOM    347  HB1 ARG    27       26.109   2.771  19.470  1.00
87.46           H
ATOM    348  HB2 ARG    27       24.426   2.650  19.980
```

```
1.00103.36            H
ATOM    349  HG1 ARG    27      25.088   2.297  22.285
1.00281.97            H
ATOM    350  HG2 ARG    27      26.775   2.638  21.899
1.00276.19            H
ATOM    351  HD1 ARG    27      26.864   0.657  20.456
1.00183.60            H
ATOM    352  HD2 ARG    27      25.161   0.337  20.767
1.00142.93            H
ATOM    353  HE  ARG    27      25.731  -0.516  22.856
1.00372.53            H
ATOM    354 HH11 ARG    27      27.245  -1.214  24.397
1.00910.59            H
ATOM    355 HH12 ARG    27      28.897  -0.709  24.269
1.00999.99            H
ATOM    356 HH21 ARG    27      28.380   1.171  21.403
1.00532.54            H
ATOM    357 HH22 ARG    27      29.540   0.642  22.575
1.00948.84            H
ATOM    358  N   SER    28      23.178   4.889  21.486  1.00
14.60         N
ATOM    359  CA  SER    28      22.207   5.171  22.549  1.00
14.92         C
ATOM    360  C   SER    28      20.984   4.265  22.427  1.00
12.12         C
ATOM    361  O   SER    28      20.609   3.850  21.331  1.00
13.41         O
ATOM    362  CB  SER    28      21.767   6.633  22.474  1.00
23.26         C
ATOM    363  OG  SER    28      22.893   7.474  22.687
1.00146.32            O
ATOM    364  HN  SER    28      22.861   4.795  20.562  1.00
15.23         H
ATOM    365  HA  SER    28      22.668   4.999  23.512  1.00
15.44         H
ATOM    366  HB1 SER    28      21.017   6.821  23.231  1.00
88.73         H
ATOM    367  HB2 SER    28      21.352   6.838  21.503
1.00124.68            H
ATOM    368  HG  SER    28      22.693   8.060  23.420
1.00242.47            H
ATOM    369  N   CYS    29      20.365   3.972  23.568  1.00
10.48         N
ATOM    370  CA  CYS    29      19.178   3.123  23.604  1.00
9.97         C
ATOM    371  C   CYS    29      18.653   3.054  25.038  1.00
11.60         C
ATOM    372  O   CYS    29      19.411   2.776  25.968  1.00
15.64         O
ATOM    373  CB  CYS    29      19.513   1.707  23.081  1.00
9.52         C
ATOM    374  SG  CYS    29      18.115   1.053  22.150  1.00
```

```
10.40           S
ATOM    375  HN  CYS    29       20.709   4.343  24.407  1.00
11.10           H
ATOM    376  HA  CYS    29       18.417   3.564  22.978  1.00
11.08           H
ATOM    377  HB1 CYS    29       19.722   1.038  23.907  1.00
9.33           H
ATOM    378  HB2 CYS    29       20.373   1.750  22.435  1.00
10.78           H
ATOM    379  N   GLU    30       17.360   3.329  25.222  1.00
13.52           N
ATOM    380  CA  GLU    30       16.760   3.311  26.561  1.00
17.41           C
ATOM    381  C   GLU    30       15.854   2.100  26.746  1.00
10.81           C
ATOM    382  O   GLU    30       14.993   1.822  25.914  1.00
23.22           O
ATOM    383  CB  GLU    30       15.951   4.590  26.770  1.00
37.83           C
ATOM    384  CG  GLU    30       16.884   5.798  26.673
1.00108.13           C
ATOM    385  CD  GLU    30       16.094   7.085  26.879
1.00240.52           C
ATOM    386  OE1 GLU    30       14.895   6.994  27.088
1.00422.56           O
ATOM    387  OE2 GLU    30       16.698   8.144  26.825
1.00410.81           O1-
ATOM    388  HN  GLU    30       16.800   3.557  24.447  1.00
15.61           H
ATOM    389  HA  GLU    30       17.543   3.276  27.308  1.00
24.60           H
ATOM    390  HB1 GLU    30       15.490   4.570  27.747  1.00
72.67           H
ATOM    391  HB2 GLU    30       15.185   4.661  26.010  1.00
56.47           H
ATOM    392  HG1 GLU    30       17.347   5.815  25.697
1.00196.66           H
ATOM    393  HG2 GLU    30       17.648   5.721  27.432
1.00140.67           H
ATOM    394  N   LYS    31       16.053   1.388  27.852  1.00
11.96           N
ATOM    395  CA  LYS    31       15.249   0.206  28.153  1.00
11.40           C
ATOM    396  C   LYS    31       13.976   0.602  28.895  1.00
10.99           C
ATOM    397  O   LYS    31       13.956   1.590  29.629  1.00
12.86           O
ATOM    398  CB  LYS    31       16.059  -0.768  29.011  1.00
21.40           C
ATOM    399  CG  LYS    31       17.263  -1.271  28.213  1.00
57.81           C
ATOM    400  CD  LYS    31       18.075  -2.244  29.069
```

```
1.00115.52          C
ATOM    401  CE  LYS    31      19.301  -2.715  28.283
1.00250.23          C
ATOM    402  NZ  LYS    31      18.856  -3.466  27.075
1.00462.10          N1+
ATOM    403  HN  LYS    31      16.752   1.665  28.479  1.00
26.13          H
ATOM    404  HA  LYS    31      14.979  -0.286  27.231  1.00
13.87          H
ATOM    405  HB1 LYS    31      15.437  -1.607  29.287  1.00
37.63          H
ATOM    406  HB2 LYS    31      16.401  -0.264  29.903  1.00
47.19          H
ATOM    407  HG1 LYS    31      17.886  -0.434  27.934
1.00126.32          H
ATOM    408  HG2 LYS    31      16.919  -1.776  27.322
1.00114.19          H
ATOM    409  HD1 LYS    31      17.464  -3.097  29.325
1.00198.45          H
ATOM    410  HD2 LYS    31      18.397  -1.747  29.974
1.00200.14          H
ATOM    411  HE1 LYS    31      19.903  -3.359  28.908
1.00374.64          H
ATOM    412  HE2 LYS    31      19.884  -1.859  27.980
1.00403.27          H
ATOM    413  HZ1 LYS    31      19.470  -4.293  26.935
1.00627.38          H
ATOM    414  HZ2 LYS    31      18.913  -2.845  26.242
1.00622.26          H
ATOM    415  HZ3 LYS    31      17.874  -3.782  27.205
1.00619.20          H
ATOM    416  N   THR    32      12.915  -0.178  28.701  1.00
15.51          N
ATOM    417  CA  THR    32      11.635   0.092  29.356  1.00
22.44          C
ATOM    418  C   THR    32      11.509  -0.727  30.637  1.00
26.60          C
ATOM    419  O   THR    32      11.599  -1.954  30.611  1.00
57.43          O
ATOM    420  CB  THR    32      10.484  -0.264  28.412  1.00
56.28          C
ATOM    421  CG2 THR    32       9.149  -0.010  29.112
1.00102.70          C
ATOM    422  OG1 THR    32      10.569   0.538  27.242
1.00111.17          O
ATOM    423  HN  THR    32      12.994  -0.952  28.105  1.00
18.79          H
ATOM    424  HA  THR    32      11.568   1.144  29.601  1.00
19.67          H
ATOM    425  HB  THR    32      10.550  -1.305  28.140  1.00
84.25          H
ATOM    426  HG1 THR    32      10.302   1.431  27.474
```

```
1.00205.03            H
ATOM    427 HG21 THR    32        8.999  -0.752  29.882
1.00220.71            H
ATOM    428 HG22 THR    32        8.347  -0.074  28.392
1.00174.67            H
ATOM    429 HG23 THR    32        9.156   0.973  29.557
1.00217.44            H
ATOM    430  N   THR    33       11.297  -0.040  31.759  1.00
22.47           N
ATOM    431  CA  THR    33       11.156  -0.711  33.051  1.00
41.60           C
ATOM    432  C   THR    33       10.179   0.018  33.929  1.00
38.03           C
ATOM    433  O   THR    33       10.554   0.821  34.784  1.00
52.73           O
ATOM    434  CB  THR    33       12.506  -0.813  33.756  1.00
63.25           C
ATOM    435  CG2 THR    33       13.403  -1.798  33.006
1.00121.65            C
ATOM    436  OG1 THR    33       13.121   0.468  33.785  1.00
96.20           O
ATOM    437  HN  THR    33       11.233   0.937  31.718  1.00
23.82           H
ATOM    438  HA  THR    33       10.763  -1.700  32.904  1.00
67.12           H
ATOM    439  HB  THR    33       12.352  -1.166  34.762
1.00107.59            H
ATOM    440  HG1 THR    33       13.674   0.550  33.005
1.00176.27            H
ATOM    441 HG21 THR    33       12.887  -2.741  32.895
1.00255.23            H
ATOM    442 HG22 THR    33       14.316  -1.949  33.563
1.00245.37            H
ATOM    443 HG23 THR    33       13.638  -1.400  32.030
1.00185.78            H
ATOM    444  N   GLY    34        8.916  -0.293  33.720  1.00
40.93           N
ATOM    445  CA  GLY    34        7.862   0.305  34.502  1.00
45.59           C
ATOM    446  C   GLY    34        7.554   1.714  34.015  1.00
40.97           C
ATOM    447  O   GLY    34        6.610   2.352  34.479  1.00
82.17           O
ATOM    448  HN  GLY    34        8.693  -0.956  33.035  1.00
54.00           H
ATOM    449  HA1 GLY    34        8.189   0.341  35.523  1.00
54.41           H
ATOM    450  HA2 GLY    34        6.974  -0.305  34.431  1.00
62.09           H
ATOM    451  N   ASN    35        8.357   2.189  33.066  1.00
32.51           N
ATOM    452  CA  ASN    35        8.171   3.523  32.504  1.00
```

```
33.20           C
ATOM    453  C   ASN    35        8.779   3.597  31.108  1.00
23.17           C
ATOM    454  O   ASN    35        9.761   2.917  30.812  1.00
27.01           O
ATOM    455  CB  ASN    35        8.830   4.574  33.400  1.00
54.51           C
ATOM    456  CG  ASN    35        8.222   4.529  34.798  1.00
80.73           C
ATOM    457  ND2 ASN    35        9.002   4.389  35.834
1.00220.46           N
ATOM    458  OD1 ASN    35        7.005   4.629  34.950
1.00117.80           O
ATOM    459  HN  ASN    35        9.090   1.628  32.735  1.00
51.15           H
ATOM    460  HA  ASN    35        7.114   3.734  32.437  1.00
47.21           H
ATOM    461  HB1 ASN    35        8.674   5.553  32.973  1.00
68.31           H
ATOM    462  HB2 ASN    35        9.890   4.374  33.463  1.00
57.75           H
ATOM    463 HD21 ASN    35        9.971   4.311  35.711
1.00405.39           H
ATOM    464 HD22 ASN    35        8.619   4.361  36.736
1.00245.64           H
ATOM    465  N   PHE    36        8.189   4.426  30.251  1.00
35.72           N
ATOM    466  CA  PHE    36        8.669   4.589  28.888  1.00
33.41           C
ATOM    467  C   PHE    36        9.517   5.851  28.767  1.00
28.51           C
ATOM    468  O   PHE    36        9.170   6.897  29.314  1.00
45.23           O
ATOM    469  CB  PHE    36        7.467   4.688  27.959  1.00
67.53           C
ATOM    470  CG  PHE    36        6.712   3.382  27.984  1.00
86.71           C
ATOM    471  CD1 PHE    36        5.643   3.212  28.872
1.00106.51           C
ATOM    472  CD2 PHE    36        7.080   2.342  27.125  1.00
99.48           C
ATOM    473  CE1 PHE    36        4.941   2.002  28.898
1.00134.48           C
ATOM    474  CE2 PHE    36        6.378   1.131  27.151
1.00134.27           C
ATOM    475  CZ  PHE    36        5.309   0.961  28.037
1.00149.48           C
ATOM    476  HN  PHE    36        7.409   4.940  30.538  1.00
62.87           H
ATOM    477  HA  PHE    36        9.261   3.732  28.602  1.00
28.53           H
ATOM    478  HB1 PHE    36        7.802   4.891  26.963  1.00
```

73.56 H
ATOM 479 HB2 PHE 36 6.818 5.487 28.291 1.00
88.42 H
ATOM 480 HD1 PHE 36 5.361 4.016 29.537
1.00110.20 H
ATOM 481 HD2 PHE 36 7.909 2.471 26.443 1.00
93.07 H
ATOM 482 HE1 PHE 36 4.115 1.871 29.582
1.00155.06 H
ATOM 483 HE2 PHE 36 6.663 0.328 26.487
1.00159.46 H
ATOM 484 HZ PHE 36 4.767 0.027 28.056
1.00183.25 H
ATOM 485 N THR 37 10.630 5.746 28.043 1.00
20.06 N
ATOM 486 CA THR 37 11.522 6.884 27.857 1.00
27.18 C
ATOM 487 C THR 37 12.258 6.772 26.525 1.00
41.87 C
ATOM 488 O THR 37 13.001 5.818 26.292
1.00169.86 O
ATOM 489 CB THR 37 12.539 6.944 28.999 1.00
26.52 C
ATOM 490 CG2 THR 37 11.811 7.193 30.321 1.00
75.69 C
ATOM 491 OG1 THR 37 13.244 5.713 29.068 1.00
81.95 O
ATOM 492 HN THR 37 10.855 4.889 27.629 1.00
18.47 H
ATOM 493 HA THR 37 10.943 7.797 27.857 1.00
38.80 H
ATOM 494 HB THR 37 13.236 7.749 28.819 1.00
81.60 H
ATOM 495 HG1 THR 37 14.049 5.803 28.553
1.00188.80 H
ATOM 496 HG21 THR 37 11.095 7.991 30.193
1.00196.98 H
ATOM 497 HG22 THR 37 12.528 7.472 31.079
1.00196.23 H
ATOM 498 HG23 THR 37 11.298 6.293 30.625
1.00168.92 H
ATOM 499 N GLU 38 12.046 7.755 25.662 1.00
26.53 N
ATOM 500 CA GLU 38 12.694 7.769 24.355 1.00
30.29 C
ATOM 501 C GLU 38 14.161 8.155 24.494 1.00
25.39 C
ATOM 502 O GLU 38 14.569 8.724 25.509 1.00
51.60 O
ATOM 503 CB GLU 38 11.987 8.762 23.431 1.00
54.82 C
ATOM 504 CG GLU 38 10.581 8.251 23.114

```
1.00165.84          C
ATOM    505  CD  GLU    38       9.836   9.270  22.257
1.00292.92          C
ATOM    506  OE1 GLU    38      10.353  10.361  22.086
1.00414.19          O
ATOM    507  OE2 GLU    38       8.758   8.943  21.786
1.00537.65          O1-
ATOM    508  HN  GLU    38      11.445   8.486  25.908  1.00
84.50          H
ATOM    509  HA  GLU    38      12.631   6.782  23.921  1.00
32.15          H
ATOM    510  HB1 GLU    38      12.547   8.861  22.513
1.00144.82          H
ATOM    511  HB2 GLU    38      11.920   9.722  23.919
1.00106.49          H
ATOM    512  HG1 GLU    38      10.040   8.093  24.034
1.00303.31          H
ATOM    513  HG2 GLU    38      10.652   7.315  22.576
1.00323.99          H
ATOM    514  N   CYS    39      14.952   7.850  23.472  1.00
15.84          N
ATOM    515  CA  CYS    39      16.371   8.177  23.496  1.00
14.30          C
ATOM    516  C   CYS    39      16.548   9.663  23.837  1.00
22.31          C
ATOM    517  O   CYS    39      15.622  10.449  23.639  1.00
32.92          O
ATOM    518  CB  CYS    39      16.993   7.864  22.129  1.00
18.08          C
ATOM    519  SG  CYS    39      15.801   8.260  20.825  1.00
17.41          S
ATOM    520  HN  CYS    39      14.573   7.402  22.687  1.00
24.87          H
ATOM    521  HA  CYS    39      16.845   7.569  24.246  1.00
14.60          H
ATOM    522  HB1 CYS    39      17.243   6.813  22.077  1.00
27.71          H
ATOM    523  HB2 CYS    39      17.888   8.452  21.990  1.00
34.91          H
ATOM    524  N   PRO    40      17.692  10.080  24.344  1.00
27.27          N
ATOM    525  CA  PRO    40      17.915  11.514  24.697  1.00
47.66          C
ATOM    526  C   PRO    40      17.669  12.452  23.525  1.00
71.54          C
ATOM    527  O   PRO    40      17.643  12.039  22.365  1.00
87.70          O
ATOM    528  CB  PRO    40      19.385  11.582  25.136  1.00
53.85          C
ATOM    529  CG  PRO    40      19.739  10.191  25.520  1.00
46.04          C
ATOM    530  CD  PRO    40      18.890   9.266  24.640  1.00
```

```
25.70           C
ATOM    531  HA  PRO    40      17.280  11.789  25.526  1.00
58.04           H
ATOM    532  HB1 PRO    40      19.497  12.244  25.985  1.00
67.93           H
ATOM    533  HB2 PRO    40      20.012  11.919  24.316  1.00
70.99           H
ATOM    534  HG1 PRO    40      19.509  10.016  26.562  1.00
65.91           H
ATOM    535  HG2 PRO    40      20.788  10.024  25.340  1.00
54.99           H
ATOM    536  HD1 PRO    40      18.625   8.380  25.188  1.00
24.22           H
ATOM    537  HD2 PRO    40      19.421   9.015  23.731  1.00
26.94           H
ATOM    538  N   GLY    41      17.508  13.721  23.854
1.00105.24           N
ATOM    539  CA  GLY    41      17.283  14.746  22.842
1.00147.55           C
ATOM    540  C   GLY    41      18.551  14.987  22.037
1.00161.74           C
ATOM    541  O   GLY    41      18.510  15.155  20.818
1.00244.37           O
ATOM    542  HN  GLY    41      17.565  13.972  24.798
1.00121.79           H
ATOM    543  HA1 GLY    41      16.989  15.665  23.324
1.00184.40           H
ATOM    544  HA2 GLY    41      16.495  14.422  22.178
1.00157.34           H
ATOM    545  N   LEU    42      19.682  14.999  22.739
1.00162.48           N
ATOM    546  CA  LEU    42      20.988  15.218  22.115
1.00191.48           C
ATOM    547  C   LEU    42      21.914  14.043  22.415
1.00166.66           C
ATOM    548  O   LEU    42      21.700  13.306  23.376
1.00251.38           O
ATOM    549  CB  LEU    42      21.613  16.506  22.655
1.00307.99           C
ATOM    550  CG  LEU    42      20.652  17.683  22.450
1.00452.28           C
ATOM    551  CD1 LEU    42      21.270  18.947  23.054
1.00681.24           C
ATOM    552  CD2 LEU    42      20.396  17.900  20.950
1.00527.46           C
ATOM    553  HN  LEU    42      19.637  14.856  23.707
1.00195.47           H
ATOM    554  HA  LEU    42      20.873  15.306  21.043
1.00229.77           H
ATOM    555  HB1 LEU    42      22.536  16.704  22.132
1.00342.49           H
ATOM    556  HB2 LEU    42      21.818  16.389  23.709
```

```
1.00335.33           H
ATOM    557  HG  LEU    42      19.717  17.469  22.949
1.00427.79           H
ATOM    558 HD11 LEU    42      22.280  19.064  22.690
1.00909.71           H
ATOM    559 HD12 LEU    42      21.283  18.863  24.131
1.00706.80           H
ATOM    560 HD13 LEU    42      20.682  19.807  22.768
1.00858.82           H
ATOM    561 HD21 LEU    42      21.307  17.726  20.395
1.00631.69           H
ATOM    562 HD22 LEU    42      20.061  18.914  20.780
1.00628.06           H
ATOM    563 HD23 LEU    42      19.633  17.216  20.611
1.00656.75           H
ATOM    564  N   THR    43      22.938  13.871  21.587
1.00178.52           N
ATOM    565  CA  THR    43      23.882  12.778  21.780
1.00240.84           C
ATOM    566  C   THR    43      24.591  12.921  23.137
1.00372.31           C
ATOM    567  O   THR    43      24.795  14.038  23.613
1.00500.13           O
ATOM    568  CB  THR    43      24.906  12.785  20.642
1.00354.13           C
ATOM    569  CG2 THR    43      24.194  12.552  19.306
1.00463.04           C
ATOM    570  OG1 THR    43      25.563  14.043  20.611
1.00508.50           O
ATOM    571  HN  THR    43      23.060  14.488  20.834
1.00229.59           H
ATOM    572  HA  THR    43      23.335  11.851  21.754
1.00249.90           H
ATOM    573  HB  THR    43      25.630  12.002  20.800
1.00481.58           H
ATOM    574  HG1 THR    43      26.395  13.931  20.146
1.00624.31           H
ATOM    575 HG21 THR    43      23.708  13.463  18.992
1.00650.95           H
ATOM    576 HG22 THR    43      23.454  11.772  19.419
1.00580.73           H
ATOM    577 HG23 THR    43      24.917  12.256  18.560
1.00571.99           H
ATOM    578  N   PRO    44      24.956  11.826  23.778
1.00488.39           N
ATOM    579  CA  PRO    44      25.635  11.863  25.114
1.00760.13           C
ATOM    580  C   PRO    44      27.104  12.285  25.031
1.00735.36           C
ATOM    581  O   PRO    44      27.710  12.639  26.042
1.00999.99           O
ATOM    582  CB  PRO    44      25.505  10.417  25.608
```

```
1.00999.99           C
ATOM    583  CG  PRO    44      25.507   9.599  24.361
1.00856.94           C
ATOM    584  CD  PRO    44      24.768  10.436  23.312
1.00548.09           C
ATOM    585  HA  PRO    44      25.101  12.519  25.782
1.00939.00           H
ATOM    586  HB1 PRO    44      24.571  10.282  26.137
1.00999.99           H
ATOM    587  HB2 PRO    44      26.341  10.151  26.244
1.00999.99           H
ATOM    588  HG1 PRO    44      24.987   8.667  24.520
1.00999.99           H
ATOM    589  HG2 PRO    44      26.526   9.410  24.044
1.00853.53           H
ATOM    590  HD1 PRO    44      23.719  10.185  23.300
1.00602.45           H
ATOM    591  HD2 PRO    44      25.210  10.294  22.335
1.00463.32           H
ATOM    592  N   ILE    45      27.677  12.234  23.833
1.00518.33           N
ATOM    593  CA  ILE    45      29.074  12.597  23.651
1.00582.79           C
ATOM    594  C   ILE    45      29.415  13.867  24.429
1.00795.93           C
ATOM    595  O   ILE    45      28.546  14.698  24.692
1.00898.75           O
ATOM    596  CB  ILE    45      29.358  12.798  22.159
1.00474.41           C
ATOM    597  CG1 ILE    45      28.593  14.019  21.636
1.00516.90           C
ATOM    598  CG2 ILE    45      28.915  11.552  21.390
1.00760.75           C
ATOM    599  CD1 ILE    45      28.846  14.175  20.135
1.00566.81           C
ATOM    600  HN  ILE    45      27.158  11.935  23.059
1.00395.72           H
ATOM    601  HA  ILE    45      29.691  11.792  24.014
1.00711.06           H
ATOM    602  HB  ILE    45      30.413  12.944  22.020
1.00477.81           H
ATOM    603 HG11 ILE    45      28.933  14.908  22.144
1.00624.28           H
ATOM    604 HG12 ILE    45      27.539  13.883  21.815
1.00744.84           H
ATOM    605 HG21 ILE    45      27.838  11.489  21.398
1.00920.57           H
ATOM    606 HG22 ILE    45      29.331  10.673  21.858
1.00999.99           H
ATOM    607 HG23 ILE    45      29.265  11.614  20.370
1.00865.46           H
ATOM    608 HD11 ILE    45      29.902  14.065  19.935
```

```
1.00658.89          H
ATOM    609 HD12 ILE    45      28.518  15.152  19.814
1.00734.30          H
ATOM    610 HD13 ILE    45      28.298  13.415  19.596
1.00675.84          H
ATOM    611  N   ALA    46      30.686  14.007  24.794
1.00999.99          N
ATOM    612  CA  ALA    46      31.130  15.179  25.541
1.00999.99          C
ATOM    613  C   ALA    46      32.649  15.180  25.683
1.00999.99          C
ATOM    614  CB  ALA    46      30.485  15.192  26.928
1.00999.99          C
ATOM    615  OT1 ALA    46      33.319  15.089  24.667
1.00999.99          O
ATOM    616  OT2 ALA    46      33.120  15.271  26.805
1.00999.99          O
ATOM    617  HN  ALA    46      31.334  13.312  24.556
1.00999.99          H
ATOM    618  HA  ALA    46      30.828  16.068  25.010
1.00999.99          H
ATOM    619  HB1 ALA    46      30.960  15.946  27.538
1.00999.99          H
ATOM    620  HB2 ALA    46      30.609  14.224  27.392
1.00999.99          H
ATOM    621  HB3 ALA    46      29.434  15.414  26.834
1.00999.99          H
ENDMDL
TER
MODEL        9
ATOM      1  N   GLY     1      24.545  -4.470  18.883
1.00999.99          N
ATOM      2  CA  GLY     1      24.924  -3.104  19.343
1.00999.99          C
ATOM      3  C   GLY     1      24.376  -2.066  18.367
1.00999.99          C
ATOM      4  O   GLY     1      23.873  -1.021  18.776
1.00999.99          O
ATOM      5  HA1 GLY     1      25.999  -3.022  19.383
1.00999.99          H
ATOM      6  HA2 GLY     1      24.512  -2.927  20.325
1.00999.99          H
ATOM      7  HT1 GLY     1      24.693  -4.544  17.857
1.00999.99          H
ATOM      8  HT2 GLY     1      23.542  -4.643  19.103
1.00999.99          H
ATOM      9  HT3 GLY     1      25.134  -5.175  19.369
1.00999.99          H
ATOM     10  N   LEU     2      24.477  -2.366  17.077
1.00999.99          N
ATOM     11  CA  LEU     2      23.990  -1.450  16.052
1.00895.59          C
```

```
ATOM     12  C   LEU     2       22.466  -1.406  16.060
1.00532.27          C
ATOM     13  O   LEU     2       21.808  -2.400  16.368
1.00625.21          O
ATOM     14  CB  LEU     2       24.476  -1.902  14.671
1.00999.99          C
ATOM     15  CG  LEU     2       26.008  -1.956  14.645
1.00999.99          C
ATOM     16  CD1 LEU     2       26.465  -2.490  13.283
1.00999.99          C
ATOM     17  CD2 LEU     2       26.595  -0.551  14.872
1.00999.99          C
ATOM     18  HN  LEU     2       24.887  -3.214  16.809
1.00999.99          H
ATOM     19  HA  LEU     2       24.367  -0.461  16.252
1.00890.87          H
ATOM     20  HB1 LEU     2       24.132  -1.202  13.925
1.00924.07          H
ATOM     21  HB2 LEU     2       24.077  -2.882  14.455
1.00999.99          H
ATOM     22  HG  LEU     2       26.355  -2.622  15.423
1.00999.99          H
ATOM     23 HD11 LEU     2       27.544  -2.494  13.241
1.00999.99          H
ATOM     24 HD12 LEU     2       26.077  -1.856  12.500
1.00999.99          H
ATOM     25 HD13 LEU     2       26.095  -3.496  13.149
1.00999.99          H
ATOM     26 HD21 LEU     2       26.655  -0.354  15.933
1.00999.99          H
ATOM     27 HD22 LEU     2       25.963   0.190  14.404
1.00999.99          H
ATOM     28 HD23 LEU     2       27.587  -0.496  14.446
1.00999.99          H
ATOM     29  N   CYS     3       21.907  -0.247  15.721
1.00271.28          N
ATOM     30  CA  CYS     3       20.456  -0.085  15.694
1.00104.50          C
ATOM     31  C   CYS     3       19.894  -0.550  14.354  1.00
90.98          C
ATOM     32  O   CYS     3       20.072   0.114  13.332
1.00207.28          O
ATOM     33  CB  CYS     3       20.087   1.383  15.914  1.00
37.83          C
ATOM     34  SG  CYS     3       20.711   1.936  17.521  1.00
81.56          S
ATOM     35  HN  CYS     3       22.480   0.513  15.486
1.00296.55          H
ATOM     36  HA  CYS     3       20.018  -0.675  16.484
1.00166.45          H
ATOM     37  HB1 CYS     3       19.013   1.492  15.889  1.00
40.38          H
```

```
ATOM     38  HB2 CYS     3      20.528   1.986  15.132  1.00
98.45           H
ATOM     39  N   SER     4      19.211  -1.688  14.367  1.00
85.31           N
ATOM     40  CA  SER     4      18.621  -2.229  13.147  1.00
92.94           C
ATOM     41  C   SER     4      17.600  -1.253  12.594  1.00
67.37           C
ATOM     42  O   SER     4      17.524  -1.017  11.389
1.00102.28           O
ATOM     43  CB  SER     4      17.906  -3.543  13.446
1.00123.83           C
ATOM     44  OG  SER     4      17.445  -4.114  12.228
1.00178.89           O
ATOM     45  HN  SER     4      19.097  -2.174  15.212
1.00163.03           H
ATOM     46  HA  SER     4      19.393  -2.400  12.414
1.00129.78           H
ATOM     47  HB1 SER     4      17.060  -3.342  14.098
1.00105.26           H
ATOM     48  HB2 SER     4      18.583  -4.228  13.931
1.00156.87           H
ATOM     49  HG  SER     4      17.558  -5.066  12.286
1.00218.16           H
ATOM     50  N   GLU     5      16.801  -0.706  13.501  1.00
41.67           N
ATOM     51  CA  GLU     5      15.750   0.232  13.143  1.00
41.21           C
ATOM     52  C   GLU     5      15.610   1.304  14.218  1.00
41.92           C
ATOM     53  O   GLU     5      16.473   1.437  15.085  1.00
74.72           O
ATOM     54  CB  GLU     5      14.434  -0.523  12.988  1.00
49.24           C
ATOM     55  CG  GLU     5      14.123  -1.251  14.288  1.00
55.48           C
ATOM     56  CD  GLU     5      12.952  -2.208  14.089  1.00
92.06           C
ATOM     57  OE1 GLU     5      12.589  -2.877  15.044
1.00206.49           O
ATOM     58  OE2 GLU     5      12.432  -2.256  12.987
1.00197.24           O1-
ATOM     59  HN  GLU     5      16.912  -0.955  14.443  1.00
45.52           H
ATOM     60  HA  GLU     5      15.991   0.702  12.208  1.00
60.63           H
ATOM     61  HB1 GLU     5      14.522  -1.243  12.187  1.00
61.59           H
ATOM     62  HB2 GLU     5      13.644   0.169  12.764  1.00
63.66           H
ATOM     63  HG1 GLU     5      13.872  -0.531  15.052  1.00
58.03           H
```

```
ATOM     64  HG2 GLU     5       14.994  -1.806  14.593  1.00
57.77           H
ATOM     65  N   ASN     6       14.523   2.064  14.162  1.00
50.73           N
ATOM     66  CA  ASN     6       14.296   3.115  15.144  1.00
71.04           C
ATOM     67  C   ASN     6       14.227   2.514  16.545  1.00
65.17           C
ATOM     68  O   ASN     6       14.748   3.086  17.504  1.00
94.86           O
ATOM     69  CB  ASN     6       12.991   3.852  14.836  1.00
94.83           C
ATOM     70  CG  ASN     6       13.161   4.709  13.586
1.00160.94           C
ATOM     71  ND2 ASN     6       12.105   5.107  12.930
1.00244.88           N
ATOM     72  OD1 ASN     6       14.285   5.027  13.198
1.00219.73           O
ATOM     73  HN  ASN     6       13.864   1.916  13.451  1.00
72.81           H
ATOM     74  HA  ASN     6       15.114   3.818  15.103  1.00
96.02           H
ATOM     75  HB1 ASN     6       12.731   4.485  15.671
1.00111.09           H
ATOM     76  HB2 ASN     6       12.204   3.131  14.672  1.00
98.13           H
ATOM     77 HD21 ASN     6       11.211   4.853  13.241
1.00272.43           H
ATOM     78 HD22 ASN     6       12.207   5.659  12.127
1.00336.78           H
ATOM     79  N   GLY     7       13.587   1.351  16.651  1.00
51.80           N
ATOM     80  CA  GLY     7       13.454   0.656  17.936  1.00
71.47           C
ATOM     81  C   GLY     7       14.106  -0.719  17.874  1.00
41.04           C
ATOM     82  O   GLY     7       13.441  -1.744  18.028  1.00
44.54           O
ATOM     83  HN  GLY     7       13.200   0.945  15.849  1.00
45.49           H
ATOM     84  HA1 GLY     7       12.412   0.535  18.164
1.00103.52           H
ATOM     85  HA2 GLY     7       13.925   1.236  18.719
1.00108.67           H
ATOM     86  N   ASP     8       15.411  -0.730  17.642  1.00
27.00           N
ATOM     87  CA  ASP     8       16.153  -1.984  17.553  1.00
14.15           C
ATOM     88  C   ASP     8       16.054  -2.757  18.862  1.00
9.37           C
ATOM     89  O   ASP     8       15.843  -3.970  18.864  1.00
14.61           O
```

```
ATOM     90  CB  ASP     8       17.623  -1.705  17.233  1.00
15.06           C
ATOM     91  CG  ASP     8       18.374  -3.021  17.055  1.00
21.22           C
ATOM     92  OD1 ASP     8       17.791  -4.056  17.335
1.00119.56           O
ATOM     93  OD2 ASP     8       19.520  -2.974  16.643
1.00133.27           O1-
ATOM     94  HN  ASP     8       15.881   0.120  17.524  1.00
35.63           H
ATOM     95  HA  ASP     8       15.733  -2.583  16.765  1.00
20.92           H
ATOM     96  HB1 ASP     8       18.067  -1.147  18.043  1.00
29.39           H
ATOM     97  HB2 ASP     8       17.687  -1.129  16.321  1.00
44.14           H
ATOM     98  N   CYS     9       16.199  -2.047  19.972  1.00
5.93           N
ATOM     99  CA  CYS     9       16.116  -2.677  21.282  1.00
7.54           C
ATOM    100  C   CYS     9       14.733  -3.285  21.480  1.00
14.60           C
ATOM    101  O   CYS     9       14.590  -4.392  22.001  1.00
25.78           O
ATOM    102  CB  CYS     9       16.392  -1.652  22.373  1.00
6.63           C
ATOM    103  SG  CYS     9       18.142  -1.228  22.326  1.00
10.91           S
ATOM    104  HN  CYS     9       16.359  -1.083  19.907  1.00
6.51           H
ATOM    105  HA  CYS     9       16.863  -3.450  21.347  1.00
11.12           H
ATOM    106  HB1 CYS     9       16.149  -2.070  23.337  1.00
11.28           H
ATOM    107  HB2 CYS     9       15.798  -0.767  22.200  1.00
5.04           H
ATOM    108  N   ALA    10       13.718  -2.546  21.047  1.00
16.26           N
ATOM    109  CA  ALA    10       12.336  -2.997  21.155  1.00
32.04           C
ATOM    110  C   ALA    10       11.424  -2.070  20.356  1.00
45.47           C
ATOM    111  O   ALA    10       11.841  -0.992  19.933
1.00119.04           O
ATOM    112  CB  ALA    10       11.901  -3.030  22.627  1.00
30.10           C
ATOM    113  HN  ALA    10       13.906  -1.678  20.636  1.00
12.81           H
ATOM    114  HA  ALA    10       12.263  -3.995  20.747  1.00
47.14           H
ATOM    115  HB1 ALA    10       12.736  -3.329  23.240  1.00
84.37           H
```

ATOM   116 HB2 ALA   10   11.094 -3.738 22.749
1.00118.20   H
ATOM   117 HB3 ALA   10   11.566 -2.050 22.930
1.00117.32   H
ATOM   118 N   ALA   11   10.181 -2.493 20.146 1.00
30.62   N
ATOM   119 CA ALA   11   9.233 -1.681 19.390 1.00
35.24   C
ATOM   120 C   ALA   11   9.001 -0.337 20.076 1.00
22.78   C
ATOM   121 O   ALA   11   8.929   0.700 19.417 1.00
53.33   O
ATOM   122 CB ALA   11   7.902 -2.424 19.258 1.00
60.49   C
ATOM   123 HN ALA   11   9.900 -3.361 20.502 1.00
50.37   H
ATOM   124 HA ALA   11   9.633 -1.507 18.403 1.00
41.70   H
ATOM   125 HB1 ALA   11   7.281 -1.922 18.532
1.00157.06   H
ATOM   126 HB2 ALA   11   7.401 -2.437 20.214
1.00148.83   H
ATOM   127 HB3 ALA   11   8.087 -3.438 18.934
1.00137.06   H
ATOM   128 N   ASP   12   8.882 -0.361 21.401 1.00
18.98   N
ATOM   129 CA ASP   12   8.654   0.866 22.165 1.00
31.05   C
ATOM   130 C   ASP   12   9.969   1.582 22.457 1.00
22.98   C
ATOM   131 O   ASP   12   10.015   2.810 22.531 1.00
37.14   O
ATOM   132 CB ASP   12   7.955   0.530 23.485 1.00
48.35   C
ATOM   133 CG ASP   12   6.529   0.060 23.213
1.00104.83   C
ATOM   134 OD1 ASP   12   5.932 -0.508 24.113
1.00258.73   O
ATOM   135 OD2 ASP   12   6.056   0.275 22.110
1.00213.46   O1-
ATOM   136 HN ASP   12   8.946 -1.218 21.874 1.00
36.19   H
ATOM   137 HA ASP   12   8.016   1.523 21.594 1.00
49.76   H
ATOM   138 HB1 ASP   12   7.928   1.410 24.109 1.00
95.71   H
ATOM   139 HB2 ASP   12   8.501 -0.252 23.990 1.00
54.37   H
ATOM   140 N   GLU   13   11.036   0.808 22.631 1.00
17.60   N
ATOM   141 CA GLU   13   12.347   1.379 22.924 1.00
12.01   C

```
ATOM    142  C   GLU    13       12.938   2.048  21.687  1.00
10.07           C
ATOM    143  O   GLU    13       12.550   1.745  20.559  1.00
12.81           O
ATOM    144  CB  GLU    13       13.290   0.282  23.419  1.00
11.74           C
ATOM    145  CG  GLU    13       12.785  -0.253  24.761  1.00
13.49           C
ATOM    146  CD  GLU    13       13.596  -1.478  25.173
1.00142.36           C
ATOM    147  OE1 GLU    13       12.987  -2.480  25.510
1.00339.06           O
ATOM    148  OE2 GLU    13       14.812  -1.393  25.149
1.00335.28           O1-
ATOM    149  HN  GLU    13       10.937  -0.166  22.566  1.00
28.36           H
ATOM    150  HA  GLU    13       12.237   2.118  23.702  1.00
13.06           H
ATOM    151  HB1 GLU    13       14.281   0.688  23.546  1.00
13.76           H
ATOM    152  HB2 GLU    13       13.319  -0.521  22.697  1.00
9.94           H
ATOM    153  HG1 GLU    13       11.745  -0.524  24.674  1.00
66.02           H
ATOM    154  HG2 GLU    13       12.893   0.515  25.514  1.00
56.23           H
ATOM    155  N   CYS    14       13.883   2.965  21.909  1.00
9.12           N
ATOM    156  CA  CYS    14       14.539   3.688  20.816  1.00
10.69           C
ATOM    157  C   CYS    14       15.998   3.264  20.707  1.00
9.56           C
ATOM    158  O   CYS    14       16.635   2.960  21.711  1.00
13.62           O
ATOM    159  CB  CYS    14       14.470   5.195  21.070  1.00
13.96           C
ATOM    160  SG  CYS    14       15.336   6.070  19.742  1.00
42.96           S
ATOM    161  HN  CYS    14       14.148   3.160  22.833  1.00
9.76           H
ATOM    162  HA  CYS    14       14.038   3.469  19.882  1.00
15.54           H
ATOM    163  HB1 CYS    14       14.939   5.420  22.016  1.00
50.72           H
ATOM    164  HB2 CYS    14       13.437   5.510  21.095  1.00
46.13           H
ATOM    165  N   CYS    15       16.522   3.251  19.482  1.00
9.60           N
ATOM    166  CA  CYS    15       17.915   2.867  19.250  1.00
9.28           C
ATOM    167  C   CYS    15       18.505   3.766  18.176  1.00
9.92           C
```

```
ATOM    168  O   CYS    15      17.920   3.930  17.105  1.00
13.31           O
ATOM    169  CB  CYS    15      17.989   1.407  18.793  1.00
12.90           C
ATOM    170  SG  CYS    15      19.704   0.820  18.873  1.00
39.11           S
ATOM    171  HN  CYS    15      15.967   3.512  18.718  1.00
13.12           H
ATOM    172  HA  CYS    15      18.484   2.984  20.161  1.00
8.26           H
ATOM    173  HB1 CYS    15      17.630   1.331  17.777  1.00
24.82           H
ATOM    174  HB2 CYS    15      17.372   0.797  19.436  1.00
27.31           H
ATOM    175  N   VAL    16      19.662   4.355  18.460  1.00
9.56           N
ATOM    176  CA  VAL    16      20.305   5.240  17.499  1.00
11.99           C
ATOM    177  C   VAL    16      21.820   5.168  17.641  1.00
7.73           C
ATOM    178  O   VAL    16      22.357   5.248  18.746  1.00
8.83           O
ATOM    179  CB  VAL    16      19.816   6.673  17.719  1.00
18.37           C
ATOM    180  CG1 VAL    16      20.280   7.180  19.086  1.00
39.61           C
ATOM    181  CG2 VAL    16      20.371   7.575  16.618
1.00115.21           C
ATOM    182  HN  VAL    16      20.089   4.194  19.332  1.00
9.76           H
ATOM    183  HA  VAL    16      20.040   4.933  16.496  1.00
17.27           H
ATOM    184  HB  VAL    16      18.736   6.689  17.682  1.00
52.63           H
ATOM    185 HG11 VAL    16      21.347   7.340  19.069
1.00128.07           H
ATOM    186 HG12 VAL    16      20.037   6.447  19.840
1.00154.13           H
ATOM    187 HG13 VAL    16      19.781   8.110  19.313
1.00135.74           H
ATOM    188 HG21 VAL    16      20.027   8.586  16.776
1.00229.05           H
ATOM    189 HG22 VAL    16      20.025   7.222  15.659
1.00261.82           H
ATOM    190 HG23 VAL    16      21.450   7.552  16.644
1.00210.07           H
ATOM    191  N   ASP    17      22.505   5.001  16.511  1.00
14.34           N
ATOM    192  CA  ASP    17      23.963   4.902  16.509  1.00
11.90           C
ATOM    193  C   ASP    17      24.600   6.249  16.188  1.00
13.15           C
```

```
ATOM    194  O   ASP    17      24.362   6.824  15.127  1.00
25.96          O
ATOM    195  CB  ASP    17      24.409   3.875  15.467  1.00
20.80          C
ATOM    196  CG  ASP    17      24.034   2.471  15.925  1.00
28.72          C
ATOM    197  OD1 ASP    17      23.719   2.315  17.093
1.00135.42          O
ATOM    198  OD2 ASP    17      24.063   1.574  15.099
1.00114.17          O1-
ATOM    199  HN  ASP    17      22.018   4.932  15.663  1.00
28.29          H
ATOM    200  HA  ASP    17      24.305   4.575  17.482  1.00
10.49          H
ATOM    201  HB1 ASP    17      25.479   3.936  15.339  1.00
22.42          H
ATOM    202  HB2 ASP    17      23.924   4.088  14.525  1.00
29.52          H
ATOM    203  N   THR    18      25.426   6.737  17.111  1.00
11.55          N
ATOM    204  CA  THR    18      26.123   8.012  16.927  1.00
17.10          C
ATOM    205  C   THR    18      27.579   7.757  16.562  1.00
10.63          C
ATOM    206  O   THR    18      28.077   6.644  16.718  1.00
6.49          O
ATOM    207  CB  THR    18      26.054   8.843  18.214  1.00
28.52          C
ATOM    208  CG2 THR    18      24.661   9.452  18.367  1.00
45.38          C
ATOM    209  OG1 THR    18      26.338   8.009  19.326  1.00
26.49          O
ATOM    210  HN  THR    18      25.583   6.223  17.930  1.00
13.96          H
ATOM    211  HA  THR    18      25.656   8.569  16.126  1.00
26.43          H
ATOM    212  HB  THR    18      26.783   9.638  18.167  1.00
37.43          H
ATOM    213  HG1 THR    18      25.632   7.361  19.396  1.00
71.62          H
ATOM    214 HG21 THR    18      24.525   9.780  19.388
1.00100.44          H
ATOM    215 HG22 THR    18      23.914   8.711  18.123
1.00129.42          H
ATOM    216 HG23 THR    18      24.565  10.296  17.702
1.00156.21          H
ATOM    217  N   VAL    19      28.253   8.801  16.094  1.00
16.32          N
ATOM    218  CA  VAL    19      29.643   8.711  15.716  1.00
13.34          C
ATOM    219  C   VAL    19      30.099  10.052  15.182  1.00
25.88          C
```

ATOM 220 O VAL 19 29.569 10.579 14.203 1.00
42.11 O
ATOM 221 CB VAL 19 29.881 7.621 14.671 1.00
15.48 C
ATOM 222 CG1 VAL 19 28.897 7.782 13.509 1.00
29.06 C
ATOM 223 CG2 VAL 19 31.320 7.738 14.152 1.00
20.67 C
ATOM 224 HN VAL 19 27.812 9.665 16.012 1.00
26.89 H
ATOM 225 HA VAL 19 30.226 8.479 16.598 1.00
8.08 H
ATOM 226 HB VAL 19 29.744 6.657 15.133 1.00
11.49 H
ATOM 227 HG11 VAL 19 29.168 8.649 12.924
1.00137.43 H
ATOM 228 HG12 VAL 19 27.897 7.906 13.895 1.00
93.29 H
ATOM 229 HG13 VAL 19 28.935 6.903 12.883
1.00108.69 H
ATOM 230 HG21 VAL 19 31.597 6.826 13.645 1.00
99.42 H
ATOM 231 HG22 VAL 19 31.989 7.911 14.983
1.00111.36 H
ATOM 232 HG23 VAL 19 31.387 8.569 13.464 1.00
77.11 H
ATOM 233 N PHE 20 31.072 10.591 15.866 1.00
24.58 N
ATOM 234 CA PHE 20 31.641 11.887 15.540 1.00
40.34 C
ATOM 235 C PHE 20 32.872 11.743 14.651 1.00
40.37 C
ATOM 236 O PHE 20 32.774 11.665 13.426 1.00
66.02 O
ATOM 237 CB PHE 20 32.016 12.615 16.852 1.00
48.79 C
ATOM 238 CG PHE 20 32.324 11.616 17.961 1.00
35.75 C
ATOM 239 CD1 PHE 20 31.304 10.805 18.495 1.00
30.20 C
ATOM 240 CD2 PHE 20 33.625 11.517 18.468 1.00
40.95 C
ATOM 241 CE1 PHE 20 31.596 9.902 19.523 1.00
30.41 C
ATOM 242 CE2 PHE 20 33.912 10.612 19.497 1.00
45.84 C
ATOM 243 CZ PHE 20 32.899 9.804 20.024 1.00
40.81 C
ATOM 244 HN PHE 20 31.405 10.104 16.637 1.00
16.73 H
ATOM 245 HA PHE 20 30.903 12.478 15.014 1.00
59.91 H

```
ATOM    246  HB1 PHE    20       31.196  13.217  17.158  1.00
67.15          H
ATOM    247  HB2 PHE    20       32.873  13.254  16.695  1.00
58.17          H
ATOM    248  HD1 PHE    20       30.296  10.866  18.109  1.00
33.34          H
ATOM    249  HD2 PHE    20       34.407  12.140  18.069  1.00
49.35          H
ATOM    250  HE1 PHE    20       30.814   9.283  19.930  1.00
30.61          H
ATOM    251  HE2 PHE    20       34.919  10.535  19.883  1.00
61.27          H
ATOM    252  HZ  PHE    20       33.121   9.107  20.817  1.00
52.66          H
ATOM    253  N   GLU    21       34.024  11.734  15.293  1.00
41.31          N
ATOM    254  CA  GLU    21       35.296  11.634  14.600  1.00
56.16          C
ATOM    255  C   GLU    21       35.545  10.205  14.109  1.00
58.61          C
ATOM    256  O   GLU    21       36.238   9.998  13.113
1.00201.19          O
ATOM    257  CB  GLU    21       36.390  12.109  15.568  1.00
57.37          C
ATOM    258  CG  GLU    21       37.758  11.517  15.202
1.00193.56          C
ATOM    259  CD  GLU    21       38.857  12.218  15.994
1.00304.62          C
ATOM    260  OE1 GLU    21       38.595  12.592  17.126
1.00451.81          O
ATOM    261  OE2 GLU    21       39.942  12.368  15.458
1.00442.88          O1-
ATOM    262  HN  GLU    21       34.020  11.815  16.269  1.00
50.82          H
ATOM    263  HA  GLU    21       35.279  12.294  13.745  1.00
85.72          H
ATOM    264  HB1 GLU    21       36.116  11.810  16.569  1.00
46.37          H
ATOM    265  HB2 GLU    21       36.453  13.189  15.530
1.00131.81          H
ATOM    266  HG1 GLU    21       37.937  11.653  14.147
1.00314.91          H
ATOM    267  HG2 GLU    21       37.766  10.462  15.436
1.00266.92          H
ATOM    268  N   GLY    22       34.977   9.226  14.808  1.00
86.70          N
ATOM    269  CA  GLY    22       35.144   7.820  14.428
1.00104.66          C
ATOM    270  C   GLY    22       36.188   7.131  15.301  1.00
71.50          C
ATOM    271  O   GLY    22       36.279   5.903  15.314  1.00
89.87          O
```

```
ATOM    272  HN  GLY    22       34.433   9.449  15.593
1.00214.64           H
ATOM    273  HA1 GLY    22       35.455   7.756  13.394
1.00152.47           H
ATOM    274  HA2 GLY    22       34.201   7.312  14.543
1.00118.15           H
ATOM    275  N   ASP    23       36.965   7.917  16.038  1.00
44.68          N
ATOM    276  CA  ASP    23       37.981   7.346  16.914  1.00
43.33          C
ATOM    277  C   ASP    23       37.317   6.451  17.952  1.00
37.55          C
ATOM    278  O   ASP    23       37.825   5.382  18.289  1.00
61.37          O
ATOM    279  CB  ASP    23       38.764   8.458  17.617  1.00
40.74          C
ATOM    280  CG  ASP    23       39.687   9.155  16.624
1.00141.86           C
ATOM    281  OD1 ASP    23       39.854   8.636  15.533
1.00328.18           O
ATOM    282  OD2 ASP    23       40.215  10.200  16.970
1.00304.58           O1-
ATOM    283  HN  ASP    23       36.848   8.890  16.000  1.00
40.86          H
ATOM    284  HA  ASP    23       38.664   6.754  16.323  1.00
72.12          H
ATOM    285  HB1 ASP    23       39.354   8.032  18.414  1.00
94.15          H
ATOM    286  HB2 ASP    23       38.070   9.177  18.028  1.00
93.26          H
ATOM    287  N   MET    24       36.165   6.900  18.441  1.00
23.24          N
ATOM    288  CA  MET    24       35.398   6.153  19.435  1.00
32.50          C
ATOM    289  C   MET    24       33.917   6.201  19.080  1.00
25.13          C
ATOM    290  O   MET    24       33.248   7.207  19.322  1.00
54.40          O
ATOM    291  CB  MET    24       35.607   6.766  20.820  1.00
47.36          C
ATOM    292  CG  MET    24       37.099   6.774  21.155
1.00151.73           C
ATOM    293  SD  MET    24       37.347   7.508  22.792
1.00209.23           S
ATOM    294  CE  MET    24       36.890   9.209  22.368
1.00243.29           C
ATOM    295  HN  MET    24       35.816   7.758  18.119  1.00
17.32          H
ATOM    296  HA  MET    24       35.724   5.121  19.452  1.00
48.94          H
ATOM    297  HB1 MET    24       35.078   6.181  21.557
1.00124.40           H
```

```
ATOM    298  HB2 MET    24       35.230   7.778  20.825
1.00166.96            H
ATOM    299  HG1 MET    24       37.633   7.351  20.414
1.00331.71            H
ATOM    300  HG2 MET    24       37.471   5.761  21.156
1.00302.30            H
ATOM    301  HE1 MET    24       35.821   9.332  22.472
1.00340.44            H
ATOM    302  HE2 MET    24       37.176   9.421  21.350
1.00373.88            H
ATOM    303  HE3 MET    24       37.403   9.893  23.030
1.00386.81            H
ATOM    304  N   VAL    25       33.407   5.123  18.497  1.00
22.44          N
ATOM    305  CA  VAL    25       32.003   5.083  18.112  1.00
15.37          C
ATOM    306  C   VAL    25       31.107   5.027  19.340  1.00
17.47          C
ATOM    307  O   VAL    25       31.476   4.461  20.370  1.00
29.52          O
ATOM    308  CB  VAL    25       31.715   3.885  17.211  1.00
23.36          C
ATOM    309  CG1 VAL    25       30.223   3.881  16.853  1.00
58.39          C
ATOM    310  CG2 VAL    25       32.551   3.996  15.932  1.00
55.25          C
ATOM    311  HN  VAL    25       33.982   4.350  18.319  1.00
48.11          H
ATOM    312  HA  VAL    25       31.775   5.985  17.563  1.00
9.25          H
ATOM    313  HB  VAL    25       31.965   2.972  17.733  1.00
52.45          H
ATOM    314 HG11 VAL    25       29.657   3.497  17.687
1.00171.07            H
ATOM    315 HG12 VAL    25       30.060   3.257  15.989
1.00166.22            H
ATOM    316 HG13 VAL    25       29.900   4.892  16.635
1.00134.76            H
ATOM    317 HG21 VAL    25       32.301   3.184  15.266
1.00141.88            H
ATOM    318 HG22 VAL    25       33.600   3.944  16.183
1.00184.50            H
ATOM    319 HG23 VAL    25       32.343   4.937  15.446
1.00133.90            H
ATOM    320  N   THR    26       29.930   5.638  19.227  1.00
13.60          N
ATOM    321  CA  THR    26       28.970   5.687  20.330  1.00
21.19          C
ATOM    322  C   THR    26       27.600   5.161  19.905  1.00
16.55          C
ATOM    323  O   THR    26       27.119   5.451  18.810  1.00
11.43          O
```

```
ATOM    324  CB  THR    26      28.840   7.134  20.797  1.00
27.07           C
ATOM    325  CG2 THR    26      27.762   7.243  21.880  1.00
44.39           C
ATOM    326  OG1 THR    26      30.088   7.564  21.324  1.00
34.75           O
ATOM    327  HN  THR    26      29.705   6.081  18.382  1.00
10.81           H
ATOM    328  HA  THR    26      29.332   5.089  21.155  1.00
33.16           H
ATOM    329  HB  THR    26      28.574   7.756  19.953  1.00
20.60           H
ATOM    330  HG1 THR    26      30.783   7.217  20.759  1.00
77.72           H
ATOM    331 HG21 THR    26      27.930   6.487  22.632
1.00126.99           H
ATOM    332 HG22 THR    26      26.789   7.102  21.438
1.00108.32           H
ATOM    333 HG23 THR    26      27.811   8.221  22.339
1.00151.99           H
ATOM    334  N   ARG    27      26.975   4.390  20.797  1.00
22.38           N
ATOM    335  CA  ARG    27      25.648   3.816  20.543  1.00
20.21           C
ATOM    336  C   ARG    27      24.721   4.139  21.712  1.00
16.96           C
ATOM    337  O   ARG    27      25.169   4.227  22.855  1.00
20.06           O
ATOM    338  CB  ARG    27      25.765   2.299  20.394  1.00
22.14           C
ATOM    339  CG  ARG    27      26.631   1.967  19.177
1.00124.30           C
ATOM    340  CD  ARG    27      26.937   0.469  19.163
1.00109.38           C
ATOM    341  NE  ARG    27      27.768   0.131  18.015
1.00227.73           N
ATOM    342  CZ  ARG    27      28.317  -1.074  17.897
1.00426.12           C
ATOM    343  NH1 ARG    27      29.058  -1.356  16.860
1.00767.09           N1+
ATOM    344  NH2 ARG    27      28.117  -1.974  18.820
1.00581.78           N
ATOM    345  HN  ARG    27      27.414   4.206  21.654  1.00
30.75           H
ATOM    346  HA  ARG    27      25.234   4.233  19.634  1.00
23.13           H
ATOM    347  HB1 ARG    27      24.782   1.873  20.259  1.00
87.46           H
ATOM    348  HB2 ARG    27      26.218   1.883  21.283
1.00103.36           H
ATOM    349  HG1 ARG    27      27.556   2.521  19.231
1.00281.97           H
```

ATOM 350 HG2 ARG 27 26.101 2.237 18.274
1.00276.19 H
ATOM 351 HD1 ARG 27 26.009 -0.083 19.110
1.00183.60 H
ATOM 352 HD2 ARG 27 27.458 0.203 20.071
1.00142.93 H
ATOM 353 HE ARG 27 27.926 0.803 17.319
1.00372.53 H
ATOM 354 HH11 ARG 27 29.213 -0.665 16.154
1.00910.59 H
ATOM 355 HH12 ARG 27 29.471 -2.261 16.772
1.00999.99 H
ATOM 356 HH21 ARG 27 27.550 -1.758 19.615
1.00532.54 H
ATOM 357 HH22 ARG 27 28.530 -2.881 18.731
1.00948.84 H
ATOM 358 N SER 28 23.427 4.325 21.430 1.00
14.60 N
ATOM 359 CA SER 28 22.461 4.647 22.487 1.00
14.92 C
ATOM 360 C SER 28 21.194 3.809 22.351 1.00
12.12 C
ATOM 361 O SER 28 20.778 3.461 21.244 1.00
13.41 O
ATOM 362 CB SER 28 22.100 6.132 22.424 1.00
23.26 C
ATOM 363 OG SER 28 23.264 6.908 22.679
1.00146.32 O
ATOM 364 HN SER 28 23.116 4.250 20.502 1.00
15.23 H
ATOM 365 HA SER 28 22.902 4.443 23.454 1.00
15.44 H
ATOM 366 HB1 SER 28 21.340 6.349 23.163 1.00
88.73 H
ATOM 367 HB2 SER 28 21.725 6.371 21.444
1.00124.68 H
ATOM 368 HG SER 28 22.981 7.785 22.950
1.00242.47 H
ATOM 369 N CYS 29 20.581 3.499 23.490 1.00
10.48 N
ATOM 370 CA CYS 29 19.351 2.714 23.513 1.00
9.97 C
ATOM 371 C CYS 29 18.837 2.626 24.950 1.00
11.60 C
ATOM 372 O CYS 29 19.598 2.322 25.869 1.00
15.64 O
ATOM 373 CB CYS 29 19.599 1.302 22.937 1.00
9.52 C
ATOM 374 SG CYS 29 18.149 0.758 22.015 1.00
10.40 S
ATOM 375 HN CYS 29 20.957 3.819 24.336 1.00
11.10 H

ATOM 376 HA CYS 29 18.614 3.219 22.911 1.00
11.08 H
ATOM 377 HB1 CYS 29 19.787 0.596 23.736 1.00
9.33 H
ATOM 378 HB2 CYS 29 20.448 1.322 22.273 1.00
10.78 H
ATOM 379 N GLU 30 17.549 2.914 25.146 1.00
13.52 N
ATOM 380 CA GLU 30 16.953 2.885 26.482 1.00
17.41 C
ATOM 381 C GLU 30 16.021 1.684 26.636 1.00
10.81 C
ATOM 382 O GLU 30 15.663 1.030 25.655 1.00
23.22 O
ATOM 383 CB GLU 30 16.175 4.190 26.718 1.00
37.83 C
ATOM 384 CG GLU 30 15.564 4.669 25.399
1.00108.13 C
ATOM 385 CD GLU 30 14.714 3.561 24.790
1.00240.52 C
ATOM 386 OE1 GLU 30 15.258 2.777 24.030
1.00422.56 O
ATOM 387 OE2 GLU 30 13.533 3.512 25.092
1.00410.81 O1-
ATOM 388 HN GLU 30 16.988 3.166 24.383 1.00
15.61 H
ATOM 389 HA GLU 30 17.736 2.809 27.227 1.00
24.60 H
ATOM 390 HB1 GLU 30 16.847 4.948 27.093 1.00
72.67 H
ATOM 391 HB2 GLU 30 15.390 4.022 27.437 1.00
56.47 H
ATOM 392 HG1 GLU 30 16.351 4.936 24.712
1.00196.66 H
ATOM 393 HG2 GLU 30 14.946 5.534 25.587
1.00140.67 H
ATOM 394 N LYS 31 15.634 1.405 27.882 1.00
11.96 N
ATOM 395 CA LYS 31 14.742 0.284 28.184 1.00
11.40 C
ATOM 396 C LYS 31 13.561 0.758 29.026 1.00
10.99 C
ATOM 397 O LYS 31 13.691 1.685 29.827 1.00
12.86 O
ATOM 398 CB LYS 31 15.510 -0.797 28.947 1.00
21.40 C
ATOM 399 CG LYS 31 16.625 -1.351 28.058 1.00
57.81 C
ATOM 400 CD LYS 31 17.398 -2.429 28.818
1.00115.52 C
ATOM 401 CE LYS 31 18.517 -2.975 27.930
1.00250.23 C

```
ATOM    402  NZ  LYS    31      19.279  -4.018  28.673
1.00462.10           N1+
ATOM    403  HN  LYS    31      15.957   1.967  28.617  1.00
26.13          H
ATOM    404  HA  LYS    31      14.364  -0.139  27.264  1.00
13.87          H
ATOM    405  HB1 LYS    31      14.837  -1.596  29.216  1.00
37.63          H
ATOM    406  HB2 LYS    31      15.939  -0.369  29.841  1.00
47.19          H
ATOM    407  HG1 LYS    31      17.299  -0.552  27.786
1.00126.32           H
ATOM    408  HG2 LYS    31      16.194  -1.778  27.164
1.00114.19           H
ATOM    409  HD1 LYS    31      16.729  -3.233  29.084
1.00198.45           H
ATOM    410  HD2 LYS    31      17.824  -2.002  29.715
1.00200.14           H
ATOM    411  HE1 LYS    31      19.184  -2.170  27.656
1.00374.64           H
ATOM    412  HE2 LYS    31      18.090  -3.409  27.038
1.00403.27           H
ATOM    413  HZ1 LYS    31      19.229  -4.919  28.157
1.00627.38           H
ATOM    414  HZ2 LYS    31      18.866  -4.139  29.621
1.00622.26           H
ATOM    415  HZ3 LYS    31      20.272  -3.726  28.762
1.00619.20           H
ATOM    416  N   THR    32      12.411   0.118  28.840  1.00
15.51          N
ATOM    417  CA  THR    32      11.206   0.481  29.586  1.00
22.44          C
ATOM    418  C   THR    32      11.121  -0.315  30.885  1.00
26.60          C
ATOM    419  O   THR    32      11.117  -1.545  30.871  1.00
57.43          O
ATOM    420  CB  THR    32       9.967   0.201  28.735  1.00
56.28          C
ATOM    421  CG2 THR    32       8.709   0.566  29.525
1.00102.70           C
ATOM    422  OG1 THR    32      10.024   0.979  27.547
1.00111.17           O
ATOM    423  HN  THR    32      12.367  -0.612  28.187  1.00
18.79          H
ATOM    424  HA  THR    32      11.234   1.536  29.819  1.00
19.67          H
ATOM    425  HB  THR    32       9.934  -0.847  28.480  1.00
84.25          H
ATOM    426  HG1 THR    32      10.076   1.903  27.800
1.00205.03           H
ATOM    427 HG21 THR    32       8.572  -0.142  30.329
1.00220.71           H
```

```
ATOM    428 HG22 THR    32       7.852   0.538  28.870
1.00174.67           H
ATOM    429 HG23 THR    32       8.819   1.559  29.935
1.00217.44           H
ATOM    430  N   THR    33      11.048   0.397  32.008  1.00
22.47          N
ATOM    431  CA  THR    33      10.954  -0.249  33.318  1.00
41.60          C
ATOM    432  C   THR    33      10.131   0.580  34.263  1.00
38.03          C
ATOM    433  O   THR    33      10.653   1.351  35.069  1.00
52.73          O
ATOM    434  CB  THR    33      12.343  -0.475  33.908  1.00
63.25          C
ATOM    435  CG2 THR    33      13.075  -1.549  33.102
1.00121.65           C
ATOM    436  OG1 THR    33      13.080   0.739  33.868  1.00
96.20          O
ATOM    437  HN  THR    33      11.050   1.375  31.956  1.00
23.82          H
ATOM    438  HA  THR    33      10.457  -1.195  33.219  1.00
67.12          H
ATOM    439  HB  THR    33      12.242  -0.804  34.929
1.00107.59           H
ATOM    440  HG1 THR    33      12.484   1.438  33.590
1.00176.27           H
ATOM    441 HG21 THR    33      13.276  -1.178  32.108
1.00255.23           H
ATOM    442 HG22 THR    33      12.460  -2.435  33.040
1.00245.37           H
ATOM    443 HG23 THR    33      14.008  -1.792  33.590
1.00185.78           H
ATOM    444  N   GLY    34       8.830   0.389  34.169  1.00
40.93          N
ATOM    445  CA  GLY    34       7.910   1.093  35.028  1.00
45.59          C
ATOM    446  C   GLY    34       7.696   2.521  34.541  1.00
40.97          C
ATOM    447  O   GLY    34       6.863   3.254  35.074  1.00
82.17          O
ATOM    448  HN  GLY    34       8.487  -0.255  33.518  1.00
54.00          H
ATOM    449  HA1 GLY    34       8.327   1.109  36.016  1.00
54.41          H
ATOM    450  HA2 GLY    34       6.964   0.571  35.044  1.00
62.09          H
ATOM    451  N   ASN    35       8.454   2.903  33.517  1.00
32.51          N
ATOM    452  CA  ASN    35       8.349   4.240  32.944  1.00
33.20          C
ATOM    453  C   ASN    35       8.836   4.231  31.498  1.00
23.17          C
```

```
ATOM    454  O   ASN    35       9.685   3.421  31.127  1.00
27.01          O
ATOM    455  CB  ASN    35       9.186   5.231  33.755  1.00
54.51          C
ATOM    456  CG  ASN    35       8.710   5.256  35.203  1.00
80.73          C
ATOM    457  ND2 ASN    35       9.565   5.034  36.163
1.00220.46          N
ATOM    458  OD1 ASN    35       7.528   5.483  35.467
1.00117.80          O
ATOM    459  HN  ASN    35       9.095   2.268  33.134  1.00
51.15          H
ATOM    460  HA  ASN    35       7.316   4.555  32.962  1.00
47.21          H
ATOM    461  HB1 ASN    35       9.082   6.218  33.329  1.00
68.31          H
ATOM    462  HB2 ASN    35      10.223   4.934  33.723  1.00
57.75          H
ATOM    463 HD21 ASN    35      10.503   4.853  35.950
1.00405.39          H
ATOM    464 HD22 ASN    35       9.267   5.047  37.097
1.00245.64          H
ATOM    465  N   PHE    36       8.290   5.130  30.684  1.00
35.72          N
ATOM    466  CA  PHE    36       8.663   5.221  29.282  1.00
33.41          C
ATOM    467  C   PHE    36       9.649   6.361  29.052  1.00
28.51          C
ATOM    468  O   PHE    36       9.530   7.430  29.653  1.00
45.23          O
ATOM    469  CB  PHE    36       7.405   5.457  28.458  1.00
67.53          C
ATOM    470  CG  PHE    36       6.515   4.242  28.552  1.00
86.71          C
ATOM    471  CD1 PHE    36       5.503   4.192  29.517
1.00106.51          C
ATOM    472  CD2 PHE    36       6.702   3.166  27.677  1.00
99.48          C
ATOM    473  CE1 PHE    36       4.676   3.066  29.606
1.00134.48          C
ATOM    474  CE2 PHE    36       5.875   2.040  27.765
1.00134.27          C
ATOM    475  CZ  PHE    36       4.860   1.990  28.730
1.00149.48          C
ATOM    476  HN  PHE    36       7.614   5.745  31.028  1.00
62.87          H
ATOM    477  HA  PHE    36       9.117   4.292  28.962  1.00
28.53          H
ATOM    478  HB1 PHE    36       7.675   5.628  27.437  1.00
73.56          H
ATOM    479  HB2 PHE    36       6.880   6.319  28.844  1.00
88.42          H
```

```
ATOM    480  HD1 PHE    36        5.361   5.022  30.195
1.00110.20           H
ATOM    481  HD2 PHE    36        7.486   3.204  26.934  1.00
93.07          H
ATOM    482  HE1 PHE    36        3.895   3.028  30.351
1.00155.06           H
ATOM    483  HE2 PHE    36        6.018   1.210  27.089
1.00159.46           H
ATOM    484  HZ  PHE    36        4.223   1.122  28.798
1.00183.25           H
ATOM    485  N   THR    37       10.619   6.128  28.173  1.00
20.06          N
ATOM    486  CA  THR    37       11.618   7.133  27.862  1.00
27.18          C
ATOM    487  C   THR    37       12.299   6.794  26.541  1.00
41.87          C
ATOM    488  O   THR    37       12.729   5.661  26.326
1.00169.86           O
ATOM    489  CB  THR    37       12.661   7.204  28.979  1.00
26.52          C
ATOM    490  CG2 THR    37       13.420   5.880  29.057  1.00
75.69          C
ATOM    491  OG1 THR    37       13.570   8.261  28.708  1.00
81.95          O
ATOM    492  HN  THR    37       10.659   5.263  27.720  1.00
18.47          H
ATOM    493  HA  THR    37       11.135   8.094  27.771  1.00
38.80          H
ATOM    494  HB  THR    37       12.167   7.387  29.921  1.00
81.60          H
ATOM    495  HG1 THR    37       13.071   9.080  28.668
1.00188.80           H
ATOM    496 HG21 THR    37       12.722   5.059  29.001
1.00196.98           H
ATOM    497 HG22 THR    37       13.961   5.829  29.991
1.00196.23           H
ATOM    498 HG23 THR    37       14.118   5.817  28.236
1.00168.92           H
ATOM    499  N   GLU    38       12.378   7.781  25.663  1.00
26.53          N
ATOM    500  CA  GLU    38       12.999   7.597  24.351  1.00
30.29          C
ATOM    501  C   GLU    38       14.508   7.824  24.422  1.00
25.39          C
ATOM    502  O   GLU    38       15.029   8.306  25.427  1.00
51.60          O
ATOM    503  CB  GLU    38       12.387   8.573  23.346  1.00
54.82          C
ATOM    504  CG  GLU    38       10.934   8.183  23.074
1.00165.84           C
ATOM    505  CD  GLU    38       10.275   9.219  22.170
1.00292.92           C
```

ATOM 506 OE1 GLU 38 9.174 8.963 21.712
1.00414.19 O
ATOM 507 OE2 GLU 38 10.881 10.254 21.950
1.00537.65 O1-
ATOM 508 HN GLU 38 12.004 8.651 25.898 1.00
84.50 H
ATOM 509 HA GLU 38 12.814 6.588 24.010 1.00
32.15 H
ATOM 510 HB1 GLU 38 12.945 8.538 22.422
1.00144.82 H
ATOM 511 HB2 GLU 38 12.422 9.575 23.749
1.00106.49 H
ATOM 512 HG1 GLU 38 10.396 8.130 24.009
1.00303.31 H
ATOM 513 HG2 GLU 38 10.907 7.216 22.590
1.00323.99 H
ATOM 514 N CYS 39 15.201 7.475 23.340 1.00
15.84 N
ATOM 515 CA CYS 39 16.649 7.644 23.272 1.00
14.30 C
ATOM 516 C CYS 39 17.014 9.118 23.477 1.00
22.31 C
ATOM 517 O CYS 39 16.213 10.001 23.166 1.00
32.92 O
ATOM 518 CB CYS 39 17.150 7.166 21.906 1.00
18.08 C
ATOM 519 SG CYS 39 16.028 7.758 20.614 1.00
17.41 S
ATOM 520 HN CYS 39 14.727 7.101 22.569 1.00
24.87 H
ATOM 521 HA CYS 39 17.106 7.045 24.042 1.00
14.60 H
ATOM 522 HB1 CYS 39 17.180 6.087 21.890 1.00
27.71 H
ATOM 523 HB2 CYS 39 18.141 7.555 21.724 1.00
34.91 H
ATOM 524 N PRO 40 18.187 9.412 23.993 1.00
27.27 N
ATOM 525 CA PRO 40 18.617 10.820 24.233 1.00
47.66 C
ATOM 526 C PRO 40 19.024 11.524 22.941 1.00
71.54 C
ATOM 527 O PRO 40 19.752 10.967 22.119 1.00
87.70 O
ATOM 528 CB PRO 40 19.808 10.664 25.186 1.00
53.85 C
ATOM 529 CG PRO 40 20.407 9.346 24.819 1.00
46.04 C
ATOM 530 CD PRO 40 19.233 8.453 24.401 1.00
25.70 C
ATOM 531 HA PRO 40 17.832 11.371 24.725 1.00
58.04 H

```
ATOM    532  HB1 PRO    40       19.471  10.645  26.212  1.00
67.93           H
ATOM    533  HB2 PRO    40       20.524  11.466  25.039  1.00
70.99           H
ATOM    534  HG1 PRO    40       20.914   8.912  25.669  1.00
65.91           H
ATOM    535  HG2 PRO    40       21.099   9.471  23.995  1.00
54.99           H
ATOM    536  HD1 PRO    40       18.892   7.858  25.233  1.00
24.22           H
ATOM    537  HD2 PRO    40       19.516   7.823  23.572  1.00
26.94           H
ATOM    538  N   GLY    41       18.553  12.753  22.778
1.00105.24           N
ATOM    539  CA  GLY    41       18.875  13.536  21.589
1.00147.55           C
ATOM    540  C   GLY    41       20.368  13.829  21.519
1.00161.74           C
ATOM    541  O   GLY    41       20.970  13.795  20.447
1.00244.37           O
ATOM    542  HN  GLY    41       17.983  13.141  23.471
1.00121.79           H
ATOM    543  HA1 GLY    41       18.333  14.469  21.621
1.00184.40           H
ATOM    544  HA2 GLY    41       18.581  12.983  20.712
1.00157.34           H
ATOM    545  N   LEU    42       20.956  14.126  22.676
1.00162.48           N
ATOM    546  CA  LEU    42       22.386  14.436  22.760
1.00191.48           C
ATOM    547  C   LEU    42       23.132  13.319  23.482
1.00166.66           C
ATOM    548  O   LEU    42       22.630  12.743  24.448
1.00251.38           O
ATOM    549  CB  LEU    42       22.587  15.746  23.526
1.00307.99           C
ATOM    550  CG  LEU    42       21.784  16.871  22.861
1.00452.28           C
ATOM    551  CD1 LEU    42       21.937  18.152  23.686
1.00681.24           C
ATOM    552  CD2 LEU    42       22.301  17.115  21.433
1.00527.46           C
ATOM    553  HN  LEU    42       20.415  14.139  23.492
1.00195.47           H
ATOM    554  HA  LEU    42       22.798  14.543  21.767
1.00229.77           H
ATOM    555  HB1 LEU    42       23.635  16.005  23.521
1.00342.49           H
ATOM    556  HB2 LEU    42       22.254  15.621  24.546
1.00335.33           H
ATOM    557  HG  LEU    42       20.741  16.592  22.825
1.00427.79           H
```

```
ATOM    558 HD11 LEU    42      21.665  17.954  24.712
1.00909.71            H
ATOM    559 HD12 LEU    42      21.291  18.918  23.284
1.00706.80            H
ATOM    560 HD13 LEU    42      22.962  18.487  23.645
1.00858.82            H
ATOM    561 HD21 LEU    42      21.854  16.398  20.761
1.00631.69            H
ATOM    562 HD22 LEU    42      23.376  17.008  21.410
1.00628.06            H
ATOM    563 HD23 LEU    42      22.035  18.113  21.115
1.00656.75            H
ATOM    564  N   THR    43      24.338  13.026  23.010
1.00178.52            N
ATOM    565  CA  THR    43      25.155  11.983  23.617
1.00240.84            C
ATOM    566  C   THR    43      25.644  12.433  25.000
1.00372.31            C
ATOM    567  O   THR    43      25.812  13.629  25.237
1.00500.13            O
ATOM    568  CB  THR    43      26.346  11.681  22.703
1.00354.13            C
ATOM    569  CG2 THR    43      25.859  10.941  21.457
1.00463.04            C
ATOM    570  OG1 THR    43      26.964  12.902  22.319
1.00508.50            O
ATOM    571  HN  THR    43      24.687  13.522  22.241
1.00229.59            H
ATOM    572  HA  THR    43      24.557  11.092  23.717
1.00249.90            H
ATOM    573  HB  THR    43      27.058  11.066  23.227
1.00481.58            H
ATOM    574  HG1 THR    43      27.439  12.749  21.501
1.00624.31            H
ATOM    575 HG21 THR    43      26.690  10.778  20.787
1.00650.95            H
ATOM    576 HG22 THR    43      25.104  11.531  20.958
1.00580.73            H
ATOM    577 HG23 THR    43      25.438   9.988  21.746
1.00571.99            H
ATOM    578  N   PRO    44      25.863  11.517  25.923
1.00488.39            N
ATOM    579  CA  PRO    44      26.324  11.863  27.303
1.00760.13            C
ATOM    580  C   PRO    44      27.802  12.251  27.353
1.00735.36            C
ATOM    581  O   PRO    44      28.275  12.781  28.359
1.00999.99            O
ATOM    582  CB  PRO    44      26.061  10.574  28.093
1.00999.99            C
ATOM    583  CG  PRO    44      26.224   9.485  27.086
1.00856.94            C
```

```
ATOM    584  CD  PRO    44      25.706  10.055  25.761
1.00548.09           C
ATOM    585  HA  PRO    44      25.719  12.660  27.706
1.00939.00           H
ATOM    586  HB1 PRO    44      25.053  10.570  28.480
1.00999.99           H
ATOM    587  HB2 PRO    44      26.777  10.466  28.898
1.00999.99           H
ATOM    588  HG1 PRO    44      25.642   8.620  27.367
1.00999.99           H
ATOM    589  HG2 PRO    44      27.271   9.216  26.998
1.00853.53           H
ATOM    590  HD1 PRO    44      24.665   9.804  25.623
1.00602.45           H
ATOM    591  HD2 PRO    44      26.301   9.693  24.937
1.00463.32           H
ATOM    592  N   ILE    45      28.529  11.977  26.276
1.00518.33           N
ATOM    593  CA  ILE    45      29.945  12.295  26.231
1.00582.79           C
ATOM    594  C   ILE    45      30.179  13.764  26.571
1.00795.93           C
ATOM    595  O   ILE    45      31.268  14.146  26.998
1.00898.75           O
ATOM    596  CB  ILE    45      30.495  11.987  24.839
1.00474.41           C
ATOM    597  CG1 ILE    45      29.768  12.836  23.794
1.00516.90           C
ATOM    598  CG2 ILE    45      30.286  10.504  24.525
1.00760.75           C
ATOM    599  CD1 ILE    45      30.333  12.537  22.405
1.00566.81           C
ATOM    600  HN  ILE    45      28.111  11.548  25.502
1.00395.72           H
ATOM    601  HA  ILE    45      30.460  11.682  26.951
1.00711.06           H
ATOM    602  HB  ILE    45      31.547  12.210  24.818
1.00477.81           H
ATOM    603 HG11 ILE    45      29.909  13.884  24.014
1.00624.28           H
ATOM    604 HG12 ILE    45      28.717  12.604  23.815
1.00744.84           H
ATOM    605 HG21 ILE    45      30.815  10.251  23.619
1.00920.57           H
ATOM    606 HG22 ILE    45      29.232  10.310  24.394
1.00999.99           H
ATOM    607 HG23 ILE    45      30.663   9.906  25.342
1.00865.46           H
ATOM    608 HD11 ILE    45      29.950  13.258  21.698
1.00658.89           H
ATOM    609 HD12 ILE    45      30.038  11.543  22.101
1.00734.30           H
```

```
ATOM    610  HD13 ILE    45       31.411  12.599  22.433
1.00675.84          H
ATOM    611  N   ALA    46       29.148  14.581  26.377
1.00999.99          N
ATOM    612  CA  ALA    46       29.248  16.006  26.667
1.00999.99          C
ATOM    613  C   ALA    46       29.791  16.232  28.074
1.00999.99          C
ATOM    614  CB  ALA    46       27.874  16.662  26.538
1.00999.99          C
ATOM    615  OT1 ALA    46       30.099  15.254  28.733
1.00999.99          O
ATOM    616  OT2 ALA    46       29.886  17.382  28.474
1.00999.99          O
ATOM    617  HN  ALA    46       28.305  14.217  26.034
1.00999.99          H
ATOM    618  HA  ALA    46       29.919  16.462  25.953
1.00999.99          H
ATOM    619  HB1 ALA    46       27.582  16.692  25.499
1.00999.99          H
ATOM    620  HB2 ALA    46       27.916  17.668  26.930
1.00999.99          H
ATOM    621  HB3 ALA    46       27.149  16.089  27.099
1.00999.99          H
ENDMDL
TER
MODEL       10
ATOM      1  N   GLY     1       27.510  -3.058  16.368
1.00999.99          N
ATOM      2  CA  GLY     1       26.501  -2.714  17.410
1.00999.99          C
ATOM      3  C   GLY     1       25.561  -1.641  16.875
1.00999.99          C
ATOM      4  O   GLY     1       25.109  -0.770  17.619
1.00999.99          O
ATOM      5  HA1 GLY     1       27.005  -2.340  18.289
1.00999.99          H
ATOM      6  HA2 GLY     1       25.934  -3.597  17.666
1.00999.99          H
ATOM      7  HT1 GLY     1       28.021  -3.919  16.650
1.00999.99          H
ATOM      8  HT2 GLY     1       28.184  -2.273  16.263
1.00999.99          H
ATOM      9  HT3 GLY     1       27.030  -3.226  15.462
1.00999.99          H
ATOM     10  N   LEU     2       25.268  -1.710  15.580
1.00999.99          N
ATOM     11  CA  LEU     2       24.377  -0.738  14.956
1.00895.59          C
ATOM     12  C   LEU     2       22.926  -1.041  15.313
1.00532.27          C
ATOM     13  O   LEU     2       22.542  -2.202  15.454
```

```
1.00625.21           O
ATOM     14  CB  LEU     2      24.543  -0.778  13.434
1.00999.99           C
ATOM     15  CG  LEU     2      26.003  -0.497  13.054
1.00999.99           C
ATOM     16  CD1 LEU     2      26.158  -0.625  11.536
1.00999.99           C
ATOM     17  CD2 LEU     2      26.402   0.922  13.496
1.00999.99           C
ATOM     18  HN  LEU     2      25.657  -2.426  15.035
1.00999.99           H
ATOM     19  HA  LEU     2      24.626   0.250  15.311
1.00890.87           H
ATOM     20  HB1 LEU     2      23.909  -0.029  12.984
1.00924.07           H
ATOM     21  HB2 LEU     2      24.259  -1.753  13.068
1.00999.99           H
ATOM     22  HG  LEU     2      26.643  -1.221  13.540
1.00999.99           H
ATOM     23 HD11 LEU     2      25.531   0.105  11.046
1.00999.99           H
ATOM     24 HD12 LEU     2      25.863  -1.618  11.227
1.00999.99           H
ATOM     25 HD13 LEU     2      27.189  -0.456  11.264
1.00999.99           H
ATOM     26 HD21 LEU     2      26.704   0.905  14.533
1.00999.99           H
ATOM     27 HD22 LEU     2      25.563   1.593  13.377
1.00999.99           H
ATOM     28 HD23 LEU     2      27.228   1.274  12.893
1.00999.99           H
ATOM     29  N   CYS     3      22.123   0.008  15.460
1.00271.28           N
ATOM     30  CA  CYS     3      20.717  -0.163  15.802
1.00104.50           C
ATOM     31  C   CYS     3      19.988  -0.933  14.709  1.00
90.98           C
ATOM     32  O   CYS     3      19.152  -1.792  14.991
1.00207.28           O
ATOM     33  CB  CYS     3      20.048   1.201  15.992  1.00
37.83           C
ATOM     34  SG  CYS     3      20.654   1.969  17.516  1.00
81.56           S
ATOM     35  HN  CYS     3      22.482   0.912  15.336
1.00296.55           H
ATOM     36  HA  CYS     3      20.647  -0.713  16.723
1.00166.45           H
ATOM     37  HB1 CYS     3      18.979   1.071  16.056  1.00
40.38           H
ATOM     38  HB2 CYS     3      20.283   1.837  15.152  1.00
98.45           H
ATOM     39  N   SER     4      20.306  -0.617  13.460  1.00
```

```
85.31           N
ATOM     40  CA  SER     4       19.671  -1.280  12.323  1.00
92.94           C
ATOM     41  C   SER     4       18.260  -0.739  12.120  1.00
67.37           C
ATOM     42  O   SER     4       17.767  -0.659  10.995
1.00102.28           O
ATOM     43  CB  SER     4       19.614  -2.796  12.559
1.00123.83           C
ATOM     44  OG  SER     4       19.690  -3.467  11.308
1.00178.89           O
ATOM     45  HN  SER     4       20.977   0.077  13.300
1.00163.03           H
ATOM     46  HA  SER     4       20.253  -1.086  11.435
1.00129.78           H
ATOM     47  HB1 SER     4       18.687  -3.056  13.065
1.00105.26           H
ATOM     48  HB2 SER     4       20.447  -3.097  13.172
1.00156.87           H
ATOM     49  HG  SER     4       18.823  -3.832  11.115
1.00218.16           H
ATOM     50  N   GLU     5       17.616  -0.381  13.226  1.00
41.67           N
ATOM     51  CA  GLU     5       16.256   0.138  13.182  1.00
41.21           C
ATOM     52  C   GLU     5       15.992   1.042  14.383  1.00
41.92           C
ATOM     53  O   GLU     5       16.724   1.004  15.372  1.00
74.72           O
ATOM     54  CB  GLU     5       15.266  -1.023  13.172  1.00
49.24           C
ATOM     55  CG  GLU     5       15.489  -1.874  14.414  1.00
55.48           C
ATOM     56  CD  GLU     5       14.697  -3.173  14.309  1.00
92.06           C
ATOM     57  OE1 GLU     5       14.024  -3.355  13.309
1.00206.49           O
ATOM     58  OE2 GLU     5       14.776  -3.968  15.233
1.00197.24           O1-
ATOM     59  HN  GLU     5       18.063  -0.480  14.092  1.00
45.52           H
ATOM     60  HA  GLU     5       16.127   0.707  12.283  1.00
60.63           H
ATOM     61  HB1 GLU     5       15.424  -1.626  12.291  1.00
61.59           H
ATOM     62  HB2 GLU     5       14.260  -0.640  13.172  1.00
63.66           H
ATOM     63  HG1 GLU     5       15.166  -1.327  15.286  1.00
58.03           H
ATOM     64  HG2 GLU     5       16.539  -2.096  14.498  1.00
57.77           H
ATOM     65  N   ASN     6       14.950   1.859  14.288  1.00
```

```
50.73           N
ATOM     66  CA  ASN     6       14.608   2.776  15.371  1.00
71.04           C
ATOM     67  C   ASN     6       14.279   2.014  16.651  1.00
65.17           C
ATOM     68  O   ASN     6       14.663   2.428  17.746  1.00
94.86           O
ATOM     69  CB  ASN     6       13.409   3.635  14.966  1.00
94.83           C
ATOM     70  CG  ASN     6       13.823   4.641  13.897
1.00160.94           C
ATOM     71  ND2 ASN     6       12.914   5.176  13.130
1.00244.88           N
ATOM     72  OD1 ASN     6       15.008   4.945  13.758
1.00219.73           O
ATOM     73  HN  ASN     6       14.405   1.852  13.473  1.00
72.81           H
ATOM     74  HA  ASN     6       15.450   3.425  15.558  1.00
96.02           H
ATOM     75  HB1 ASN     6       13.038   4.164  15.831
1.00111.09           H
ATOM     76  HB2 ASN     6       12.630   2.997  14.575  1.00
98.13           H
ATOM     77 HD21 ASN     6       11.973   4.931  13.242
1.00272.43           H
ATOM     78 HD22 ASN     6       13.172   5.823  12.441
1.00336.78           H
ATOM     79  N   GLY     7       13.561   0.901  16.509  1.00
51.80           N
ATOM     80  CA  GLY     7       13.177   0.081  17.663  1.00
71.47           C
ATOM     81  C   GLY     7       14.029  -1.177  17.745  1.00
41.04           C
ATOM     82  O   GLY     7       13.509  -2.285  17.879  1.00
44.54           O
ATOM     83  HN  GLY     7       13.282   0.623  15.611  1.00
45.49           H
ATOM     84  HA1 GLY     7       12.146  -0.205  17.560
1.00103.52           H
ATOM     85  HA2 GLY     7       13.296   0.652  18.574
1.00108.67           H
ATOM     86  N   ASP     8       15.339  -1.001  17.661  1.00
27.00           N
ATOM     87  CA  ASP     8       16.255  -2.134  17.721  1.00
14.15           C
ATOM     88  C   ASP     8       16.122  -2.859  19.052  1.00
9.37           C
ATOM     89  O   ASP     8       15.970  -4.081  19.094  1.00
14.61           O
ATOM     90  CB  ASP     8       17.695  -1.653  17.549  1.00
15.06           C
ATOM     91  CG  ASP     8       18.619  -2.849  17.337  1.00
```

```
21.22           C
ATOM     92  OD1 ASP     8       19.799  -2.717  17.615
1.00119.56           O
ATOM     93  OD2 ASP     8       18.131  -3.877  16.900
1.00133.27           O1-
ATOM     94  HN  ASP     8       15.693  -0.095  17.550  1.00
35.63           H
ATOM     95  HA  ASP     8       16.018  -2.817  16.926  1.00
20.92           H
ATOM     96  HB1 ASP     8       18.003  -1.117  18.433  1.00
29.39           H
ATOM     97  HB2 ASP     8       17.754  -0.995  16.694  1.00
44.14           H
ATOM     98  N   CYS     9       16.174  -2.099  20.136  1.00
5.93           N
ATOM     99  CA  CYS     9       16.051  -2.679  21.465  1.00
7.54           C
ATOM    100  C   CYS     9       14.666  -3.285  21.646  1.00
14.60           C
ATOM    101  O   CYS     9       14.511  -4.371  22.203  1.00
25.78           O
ATOM    102  CB  CYS     9       16.284  -1.610  22.524  1.00
6.63           C
ATOM    103  SG  CYS     9       18.020  -1.140  22.488  1.00
10.91           S
ATOM    104  HN  CYS     9       16.292  -1.133  20.036  1.00
6.51           H
ATOM    105  HA  CYS     9       16.800  -3.444  21.584  1.00
11.12           H
ATOM    106  HB1 CYS     9       16.040  -2.005  23.495  1.00
11.28           H
ATOM    107  HB2 CYS     9       15.671  -0.747  22.314  1.00
5.04           H
ATOM    108  N   ALA    10       13.665  -2.562  21.158  1.00
16.26           N
ATOM    109  CA  ALA    10       12.279  -3.006  21.244  1.00
32.04           C
ATOM    110  C   ALA    10       11.387  -2.086  20.414  1.00
45.47           C
ATOM    111  O   ALA    10       11.819  -1.018  19.980
1.00119.04           O
ATOM    112  CB  ALA    10       11.814  -3.013  22.705  1.00
30.10           C
ATOM    113  HN  ALA    10       13.864  -1.708  20.722  1.00
12.81           H
ATOM    114  HA  ALA    10       12.209  -4.010  20.851  1.00
47.14           H
ATOM    115  HB1 ALA    10       12.621  -3.350  23.338  1.00
84.37           H
ATOM    116  HB2 ALA    10       10.972  -3.681  22.811
1.00118.20           H
ATOM    117  HB3 ALA    10       11.521  -2.017  22.998
```

```
1.00117.32           H
ATOM    118  N   ALA    11      10.148  -2.503  20.192  1.00
30.62           N
ATOM    119  CA  ALA    11       9.216  -1.702  19.404  1.00
35.24           C
ATOM    120  C   ALA    11       8.969  -0.345  20.061  1.00
22.78           C
ATOM    121  O   ALA    11       8.908   0.680  19.380  1.00
53.33           O
ATOM    122  CB  ALA    11       7.888  -2.446  19.255  1.00
60.49           C
ATOM    123  HN  ALA    11       9.854  -3.364  20.557  1.00
50.37           H
ATOM    124  HA  ALA    11       9.636  -1.543  18.423  1.00
41.70           H
ATOM    125  HB1 ALA    11       7.408  -2.521  20.220
1.00157.06           H
ATOM    126  HB2 ALA    11       8.072  -3.436  18.865
1.00148.83           H
ATOM    127  HB3 ALA    11       7.246  -1.905  18.575
1.00137.06           H
ATOM    128  N   ASP    12       8.820  -0.341  21.383  1.00
18.98           N
ATOM    129  CA  ASP    12       8.570   0.898  22.118  1.00
31.05           C
ATOM    130  C   ASP    12       9.875   1.624  22.439  1.00
22.98           C
ATOM    131  O   ASP    12       9.908   2.853  22.524  1.00
37.14           O
ATOM    132  CB  ASP    12       7.827   0.590  23.418  1.00
48.35           C
ATOM    133  CG  ASP    12       6.409   0.120  23.111
1.00104.83           C
ATOM    134  OD1 ASP    12       5.974   0.313  21.987
1.00258.73           O
ATOM    135  OD2 ASP    12       5.779  -0.425  24.002
1.00213.46           O1-
ATOM    136  HN  ASP    12       8.873  -1.188  21.874  1.00
36.19           H
ATOM    137  HA  ASP    12       7.951   1.548  21.514  1.00
49.76           H
ATOM    138  HB1 ASP    12       7.783   1.482  24.027  1.00
95.71           H
ATOM    139  HB2 ASP    12       8.353  -0.185  23.956  1.00
54.37           H
ATOM    140  N   GLU    13      10.944   0.859  22.625  1.00
17.60           N
ATOM    141  CA  GLU    13      12.244   1.441  22.946  1.00
12.01           C
ATOM    142  C   GLU    13      12.871   2.090  21.716  1.00
10.07           C
ATOM    143  O   GLU    13      12.510   1.771  20.582  1.00
```

```
12.81           O
ATOM    144  CB  GLU    13       13.171   0.355  23.497  1.00
11.74           C
ATOM    145  CG  GLU    13       12.598  -0.170  24.816  1.00
13.49           C
ATOM    146  CD  GLU    13       13.349  -1.422  25.255
1.00142.36           C
ATOM    147  OE1 GLU    13       14.566  -1.409  25.197
1.00339.06           O
ATOM    148  OE2 GLU    13       12.695  -2.374  25.644
1.00335.28           O1-
ATOM    149  HN  GLU    13       10.858  -0.114  22.551  1.00
28.36           H
ATOM    150  HA  GLU    13       12.108   2.196  23.705  1.00
13.06           H
ATOM    151  HB1 GLU    13       14.151   0.775  23.673  1.00
13.76           H
ATOM    152  HB2 GLU    13       13.246  -0.452  22.784  1.00
9.94           H
ATOM    153  HG1 GLU    13       11.553  -0.406  24.684  1.00
66.02           H
ATOM    154  HG2 GLU    13       12.699   0.591  25.576  1.00
56.23           H
ATOM    155  N   CYS    14       13.806   3.013  21.948  1.00
9.12           N
ATOM    156  CA  CYS    14       14.485   3.723  20.858  1.00
10.69           C
ATOM    157  C   CYS    14       15.956   3.337  20.802  1.00
9.56           C
ATOM    158  O   CYS    14       16.588   3.127  21.833  1.00
13.62           O
ATOM    159  CB  CYS    14       14.370   5.231  21.071  1.00
13.96           C
ATOM    160  SG  CYS    14       15.335   6.094  19.803  1.00
42.96           S
ATOM    161  HN  CYS    14       14.050   3.226  22.876  1.00
9.76           H
ATOM    162  HA  CYS    14       14.022   3.469  19.914  1.00
15.54           H
ATOM    163  HB1 CYS    14       14.750   5.485  22.048  1.00
50.72           H
ATOM    164  HB2 CYS    14       13.334   5.528  20.999  1.00
46.13           H
ATOM    165  N   CYS    15       16.496   3.258  19.588  1.00
9.60           N
ATOM    166  CA  CYS    15       17.903   2.906  19.389  1.00
9.28           C
ATOM    167  C   CYS    15       18.495   3.802  18.313  1.00
9.92           C
ATOM    168  O   CYS    15       17.935   3.927  17.223  1.00
13.31           O
ATOM    169  CB  CYS    15       18.023   1.441  18.960  1.00
```

12.90 C
ATOM 170 SG CYS 15 19.761 0.926 18.999 1.00
39.11 S
ATOM 171 HN CYS 15 15.940 3.449 18.803 1.00
13.12 H
ATOM 172 HA CYS 15 18.451 3.052 20.312 1.00
8.26 H
ATOM 173 HB1 CYS 15 17.636 1.329 17.958 1.00
24.82 H
ATOM 174 HB2 CYS 15 17.451 0.822 19.636 1.00
27.31 H
ATOM 175 N VAL 16 19.624 4.434 18.619 1.00
9.56 N
ATOM 176 CA VAL 16 20.270 5.327 17.661 1.00
11.99 C
ATOM 177 C VAL 16 21.788 5.209 17.758 1.00
7.73 C
ATOM 178 O VAL 16 22.354 5.221 18.851 1.00
8.83 O
ATOM 179 CB VAL 16 19.837 6.768 17.940 1.00
18.37 C
ATOM 180 CG1 VAL 16 20.462 7.260 19.248 1.00
39.61 C
ATOM 181 CG2 VAL 16 20.282 7.665 16.786
1.00115.21 C
ATOM 182 HN VAL 16 20.025 4.303 19.504 1.00
9.76 H
ATOM 183 HA VAL 16 19.967 5.061 16.658 1.00
17.27 H
ATOM 184 HB VAL 16 18.760 6.804 18.026 1.00
52.63 H
ATOM 185 HG11 VAL 16 19.986 8.179 19.552
1.00128.07 H
ATOM 186 HG12 VAL 16 21.518 7.432 19.101
1.00154.13 H
ATOM 187 HG13 VAL 16 20.323 6.512 20.017
1.00135.74 H
ATOM 188 HG21 VAL 16 20.135 8.700 17.058
1.00229.05 H
ATOM 189 HG22 VAL 16 19.695 7.436 15.909
1.00261.82 H
ATOM 190 HG23 VAL 16 21.327 7.491 16.578
1.00210.07 H
ATOM 191 N ASP 17 22.439 5.088 16.604 1.00
14.34 N
ATOM 192 CA ASP 17 23.894 4.959 16.559 1.00
11.90 C
ATOM 193 C ASP 17 24.555 6.326 16.401 1.00
13.15 C
ATOM 194 O ASP 17 24.302 7.041 15.432 1.00
25.96 O
ATOM 195 CB ASP 17 24.299 4.071 15.380 1.00

```
20.80           C
ATOM    196  CG  ASP    17      23.695   2.681  15.542  1.00
28.72           C
ATOM    197  OD1 ASP    17      23.574   2.236  16.671
1.00135.42           O
ATOM    198  OD2 ASP    17      23.361   2.083  14.532
1.00114.17           O1-
ATOM    199  HN  ASP    17      21.931   5.079  15.766  1.00
28.29           H
ATOM    200  HA  ASP    17      24.244   4.503  17.473  1.00
10.49           H
ATOM    201  HB1 ASP    17      25.375   3.991  15.345  1.00
22.42           H
ATOM    202  HB2 ASP    17      23.941   4.513  14.461  1.00
29.52           H
ATOM    203  N   THR    18      25.418   6.671  17.354  1.00
11.55           N
ATOM    204  CA  THR    18      26.138   7.945  17.316  1.00
17.10           C
ATOM    205  C   THR    18      27.558   7.724  16.816  1.00
10.63           C
ATOM    206  O   THR    18      28.046   6.596  16.794  1.00
6.49           O
ATOM    207  CB  THR    18      26.185   8.572  18.711  1.00
28.52           C
ATOM    208  CG2 THR    18      24.817   9.150  19.067  1.00
45.38           C
ATOM    209  OG1 THR    18      26.547   7.582  19.659  1.00
26.49           O
ATOM    210  HN  THR    18      25.587   6.050  18.094  1.00
13.96           H
ATOM    211  HA  THR    18      25.633   8.626  16.644  1.00
26.43           H
ATOM    212  HB  THR    18      26.918   9.366  18.726  1.00
37.43           H
ATOM    213  HG1 THR    18      25.997   7.697  20.435  1.00
71.62           H
ATOM    214 HG21 THR    18      24.046   8.445  18.801
1.00100.44           H
ATOM    215 HG22 THR    18      24.668  10.073  18.526
1.00129.42           H
ATOM    216 HG23 THR    18      24.777   9.347  20.128
1.00156.21           H
ATOM    217  N   VAL    19      28.215   8.815  16.435  1.00
16.32           N
ATOM    218  CA  VAL    19      29.575   8.766  15.950  1.00
13.34           C
ATOM    219  C   VAL    19      29.993  10.157  15.519  1.00
25.88           C
ATOM    220  O   VAL    19      29.389  10.780  14.646  1.00
42.11           O
ATOM    221  CB  VAL    19      29.731   7.782  14.792  1.00
```

15.48   C
ATOM   222 CG1 VAL   19   28.636   8.021 13.747 1.00
29.06   C
ATOM   223 CG2 VAL   19   31.110   7.981 14.147 1.00
20.67   C
ATOM   224 HN VAL   19   27.781   9.685 16.497 1.00
26.89   H
ATOM   225 HA VAL   19   30.218   8.450 16.760 1.00
8.08   H
ATOM   226 HB VAL   19   29.655   6.776 15.173 1.00
11.49   H
ATOM   227 HG11 VAL   19   27.670   8.028 14.228
1.00137.43   H
ATOM   228 HG12 VAL   19   28.665   7.233 13.010 1.00
93.29   H
ATOM   229 HG13 VAL   19   28.804   8.972 13.261
1.00108.69   H
ATOM   230 HG21 VAL   19   31.854   8.099 14.923 1.00
99.42   H
ATOM   231 HG22 VAL   19   31.094   8.871 13.533
1.00111.36   H
ATOM   232 HG23 VAL   19   31.353   7.124 13.538 1.00
77.11   H
ATOM   233 N   PHE   20   31.019 10.626 16.178 1.00
24.58   N
ATOM   234 CA PHE   20   31.566 11.951 15.945 1.00
40.34   C
ATOM   235 C   PHE   20   32.738 11.908 14.968 1.00
40.37   C
ATOM   236 O   PHE   20   32.560 11.972 13.751 1.00
66.02   O
ATOM   237 CB PHE   20   32.023 12.548 17.297 1.00
48.79   C
ATOM   238 CG PHE   20   32.420 11.448 18.272 1.00
35.75   C
ATOM   239 CD1 PHE   20   31.452 10.563 18.791 1.00
30.20   C
ATOM   240 CD2 PHE   20   33.755 11.321 18.678 1.00
40.95   C
ATOM   241 CE1 PHE   20   31.827   9.568 19.694 1.00
30.41   C
ATOM   242 CE2 PHE   20   34.126 10.322 19.584 1.00
45.84   C
ATOM   243 CZ PHE   20   33.163   9.444 20.091 1.00
40.81   C
ATOM   244 HN PHE   20   31.409 10.065 16.867 1.00
16.73   H
ATOM   245 HA PHE   20   30.795 12.588 15.530 1.00
59.91   H
ATOM   246 HB1 PHE   20   31.222 13.103 17.718 1.00
67.15   H
ATOM   247 HB2 PHE   20   32.862 13.213 17.145 1.00

```
58.17          H
ATOM    248  HD1 PHE    20       30.415  10.642  18.484  1.00
33.34          H
ATOM    249  HD2 PHE    20       34.498  11.998  18.293  1.00
49.35          H
ATOM    250  HE1 PHE    20       31.084   8.893  20.088  1.00
30.61          H
ATOM    251  HE2 PHE    20       35.157  10.226  19.890  1.00
61.27          H
ATOM    252  HZ  PHE    20       33.450   8.674  20.791  1.00
52.66          H
ATOM    253  N   GLU    21       33.931  11.832  15.526  1.00
41.31          N
ATOM    254  CA  GLU    21       35.155  11.816  14.740  1.00
56.16          C
ATOM    255  C   GLU    21       35.398  10.436  14.122  1.00
58.61          C
ATOM    256  O   GLU    21       36.127  10.314  13.137
1.00201.19           O
ATOM    257  CB  GLU    21       36.308  12.241  15.666  1.00
57.37          C
ATOM    258  CG  GLU    21       37.656  11.687  15.179
1.00193.56           C
ATOM    259  CD  GLU    21       38.796  12.342  15.950
1.00304.62           C
ATOM    260  OE1 GLU    21       39.846  12.543  15.362
1.00451.81           O
ATOM    261  OE2 GLU    21       38.602  12.636  17.119
1.00442.88           O1-
ATOM    262  HN  GLU    21       33.993  11.805  16.504  1.00
50.82          H
ATOM    263  HA  GLU    21       35.065  12.540  13.944  1.00
85.72          H
ATOM    264  HB1 GLU    21       36.102  11.875  16.660  1.00
46.37          H
ATOM    265  HB2 GLU    21       36.362  13.322  15.693
1.00131.81           H
ATOM    266  HG1 GLU    21       37.770  11.896  14.126
1.00314.91           H
ATOM    267  HG2 GLU    21       37.686  10.619  15.340
1.00266.92           H
ATOM    268  N   GLY    22       34.787   9.405  14.697  1.00
86.70          N
ATOM    269  CA  GLY    22       34.951   8.044  14.183
1.00104.66           C
ATOM    270  C   GLY    22       36.060   7.302  14.923  1.00
71.50          C
ATOM    271  O   GLY    22       36.233   6.097  14.745  1.00
89.87          O
ATOM    272  HN  GLY    22       34.217   9.559  15.479
1.00214.64           H
ATOM    273  HA1 GLY    22       35.194   8.080  13.130
```

```
1.00152.47          H
ATOM    274  HA2 GLY    22       34.024   7.506  14.311
1.00118.15          H
ATOM    275  N   ASP    23       36.803   8.021  15.759  1.00
44.68           N
ATOM    276  CA  ASP    23       37.877   7.396  16.522  1.00
43.33           C
ATOM    277  C   ASP    23       37.298   6.293  17.396  1.00
37.55           C
ATOM    278  O   ASP    23       37.853   5.198  17.494  1.00
61.37           O
ATOM    279  CB  ASP    23       38.580   8.437  17.397  1.00
40.74           C
ATOM    280  CG  ASP    23       39.759   7.796  18.121
1.00141.86          C
ATOM    281  OD1 ASP    23       39.896   6.587  18.035
1.00328.18          O
ATOM    282  OD2 ASP    23       40.507   8.525  18.753
1.00304.58          O1-
ATOM    283  HN  ASP    23       36.619   8.977  15.873  1.00
40.86           H
ATOM    284  HA  ASP    23       38.593   6.967  15.838  1.00
72.12           H
ATOM    285  HB1 ASP    23       37.881   8.824  18.123  1.00
94.15           H
ATOM    286  HB2 ASP    23       38.936   9.245  16.775  1.00
93.26           H
ATOM    287  N   MET    24       36.160   6.594  18.011  1.00
23.24           N
ATOM    288  CA  MET    24       35.459   5.641  18.868  1.00
32.50           C
ATOM    289  C   MET    24       33.958   5.790  18.656  1.00
25.13           C
ATOM    290  O   MET    24       33.372   6.809  19.021  1.00
54.40           O
ATOM    291  CB  MET    24       35.805   5.901  20.337  1.00
47.36           C
ATOM    292  CG  MET    24       35.075   4.889  21.224
1.00151.73          C
ATOM    293  SD  MET    24       35.610   5.091  22.943
1.00209.23          S
ATOM    294  CE  MET    24       34.818   6.689  23.258
1.00243.29          C
ATOM    295  HN  MET    24       35.769   7.481  17.872  1.00
17.32           H
ATOM    296  HA  MET    24       35.755   4.633  18.607  1.00
48.94           H
ATOM    297  HB1 MET    24       35.496   6.900  20.607
1.00124.40          H
ATOM    298  HB2 MET    24       36.870   5.804  20.480
1.00166.96          H
ATOM    299  HG1 MET    24       35.306   3.887  20.891
```

```
1.00331.71          H
ATOM    300  HG2 MET    24       34.009   5.051  21.156
1.00302.30          H
ATOM    301  HE1 MET    24       35.443   7.482  22.875
1.00340.44          H
ATOM    302  HE2 MET    24       34.686   6.823  24.321
1.00373.88          H
ATOM    303  HE3 MET    24       33.852   6.716  22.773
1.00386.81          H
ATOM    304  N   VAL    25       33.339   4.780  18.053  1.00
22.44           N
ATOM    305  CA  VAL    25       31.907   4.840  17.792  1.00
15.37           C
ATOM    306  C   VAL    25       31.111   4.743  19.084  1.00
17.47           C
ATOM    307  O   VAL    25       31.548   4.129  20.057  1.00
29.52           O
ATOM    308  CB  VAL    25       31.476   3.725  16.843  1.00
23.36           C
ATOM    309  CG1 VAL    25       29.962   3.826  16.614  1.00
58.39           C
ATOM    310  CG2 VAL    25       32.211   3.878  15.508  1.00
55.25           C
ATOM    311  HN  VAL    25       33.852   3.995  17.771  1.00
48.11           H
ATOM    312  HA  VAL    25       31.685   5.789  17.325  1.00
9.25           H
ATOM    313  HB  VAL    25       31.711   2.766  17.282  1.00
52.45           H
ATOM    314 HG11 VAL    25       29.443   3.412  17.464
1.00171.07          H
ATOM    315 HG12 VAL    25       29.691   3.277  15.724
1.00166.22          H
ATOM    316 HG13 VAL    25       29.682   4.866  16.494
1.00134.76          H
ATOM    317 HG21 VAL    25       31.880   4.781  15.016
1.00141.88          H
ATOM    318 HG22 VAL    25       31.997   3.027  14.880
1.00184.50          H
ATOM    319 HG23 VAL    25       33.275   3.935  15.687
1.00133.90          H
ATOM    320  N   THR    26       29.941   5.375  19.084  1.00
13.60           N
ATOM    321  CA  THR    26       29.068   5.393  20.255  1.00
21.19           C
ATOM    322  C   THR    26       27.627   5.067  19.870  1.00
16.55           C
ATOM    323  O   THR    26       27.154   5.457  18.802  1.00
11.43           O
ATOM    324  CB  THR    26       29.131   6.779  20.889  1.00
27.07           C
ATOM    325  CG2 THR    26       28.185   6.852  22.093  1.00
```

44.39 C
ATOM 326 OG1 THR 26 30.461 7.029 21.321 1.00
34.75 O
ATOM 327 HN THR 26 29.662 5.857 18.277 1.00
10.81 H
ATOM 328 HA THR 26 29.410 4.665 20.977 1.00
33.16 H
ATOM 329 HB THR 26 28.844 7.518 20.151 1.00
20.60 H
ATOM 330 HG1 THR 26 30.433 7.249 22.254 1.00
77.72 H
ATOM 331 HG21 THR 26 28.333 7.789 22.607
1.00126.99 H
ATOM 332 HG22 THR 26 28.397 6.035 22.766
1.00108.32 H
ATOM 333 HG23 THR 26 27.161 6.786 21.758
1.00151.99 H
ATOM 334 N ARG 27 26.934 4.348 20.756 1.00
22.38 N
ATOM 335 CA ARG 27 25.537 3.966 20.523 1.00
20.21 C
ATOM 336 C ARG 27 24.697 4.270 21.759 1.00
16.96 C
ATOM 337 O ARG 27 25.236 4.488 22.844 1.00
20.06 O
ATOM 338 CB ARG 27 25.456 2.470 20.194 1.00
22.14 C
ATOM 339 CG ARG 27 25.939 1.647 21.391
1.00124.30 C
ATOM 340 CD ARG 27 25.972 0.166 21.011
1.00109.38 C
ATOM 341 NE ARG 27 26.411 -0.637 22.146
1.00227.73 N
ATOM 342 CZ ARG 27 26.557 -1.953 22.041
1.00426.12 C
ATOM 343 NH1 ARG 27 26.953 -2.651 23.071
1.00767.09 N1+
ATOM 344 NH2 ARG 27 26.308 -2.549 20.908
1.00581.78 N
ATOM 345 HN ARG 27 27.370 4.072 21.589 1.00
30.75 H
ATOM 346 HA ARG 27 25.142 4.528 19.687 1.00
23.13 H
ATOM 347 HB1 ARG 27 26.079 2.257 19.338 1.00
87.46 H
ATOM 348 HB2 ARG 27 24.432 2.209 19.967
1.00103.36 H
ATOM 349 HG1 ARG 27 25.265 1.787 22.223
1.00281.97 H
ATOM 350 HG2 ARG 27 26.930 1.970 21.674
1.00276.19 H
ATOM 351 HD1 ARG 27 26.652 0.025 20.185

1.00183.60 H
ATOM 352 HD2 ARG 27 24.982 -0.148 20.715
1.00142.93 H
ATOM 353 HE ARG 27 26.601 -0.198 23.002
1.00372.53 H
ATOM 354 HH11 ARG 27 27.144 -2.194 23.939
1.00910.59 H
ATOM 355 HH12 ARG 27 27.062 -3.641 22.990
1.00999.99 H
ATOM 356 HH21 ARG 27 26.005 -2.015 20.118
1.00532.54 H
ATOM 357 HH22 ARG 27 26.418 -3.540 20.827
1.00948.84 H
ATOM 358 N SER 28 23.374 4.292 21.594 1.00
14.60 N
ATOM 359 CA SER 28 22.476 4.583 22.712 1.00
14.92 C
ATOM 360 C SER 28 21.149 3.847 22.556 1.00
12.12 C
ATOM 361 O SER 28 20.696 3.586 21.441 1.00
13.41 O
ATOM 362 CB SER 28 22.216 6.087 22.793 1.00
23.26 C
ATOM 363 OG SER 28 21.359 6.356 23.894
1.00146.32 O
ATOM 364 HN SER 28 22.995 4.117 20.706 1.00
15.23 H
ATOM 365 HA SER 28 22.941 4.263 23.634 1.00
15.44 H
ATOM 366 HB1 SER 28 21.755 6.422 21.874 1.00
88.73 H
ATOM 367 HB2 SER 28 23.149 6.609 22.934
1.00124.68 H
ATOM 368 HG SER 28 21.830 6.929 24.504
1.00242.47 H
ATOM 369 N CYS 29 20.530 3.524 23.687 1.00
10.48 N
ATOM 370 CA CYS 29 19.251 2.823 23.688 1.00
9.97 C
ATOM 371 C CYS 29 18.687 2.803 25.108 1.00
11.60 C
ATOM 372 O CYS 29 19.415 2.540 26.064 1.00
15.64 O
ATOM 373 CB CYS 29 19.438 1.389 23.157 1.00
9.52 C
ATOM 374 SG CYS 29 17.959 0.860 22.282 1.00
10.40 S
ATOM 375 HN CYS 29 20.939 3.767 24.543 1.00
11.10 H
ATOM 376 HA CYS 29 18.564 3.351 23.045 1.00
11.08 H
ATOM 377 HB1 CYS 29 19.620 0.705 23.976 1.00

```
9.33           H
ATOM    378  HB2 CYS    29       20.276   1.359  22.480  1.00
10.78          H
ATOM    379  N   GLU    30       17.393   3.100  25.247  1.00
13.52          N
ATOM    380  CA  GLU    30       16.753   3.130  26.569  1.00
17.41          C
ATOM    381  C   GLU    30       15.733   2.007  26.717  1.00
10.81          C
ATOM    382  O   GLU    30       14.933   1.754  25.815  1.00
23.22          O
ATOM    383  CB  GLU    30       16.055   4.477  26.768  1.00
37.83          C
ATOM    384  CG  GLU    30       17.091   5.599  26.690
1.00108.13          C
ATOM    385  CD  GLU    30       17.997   5.561  27.916
1.00240.52          C
ATOM    386  OE1 GLU    30       19.033   6.203  27.883
1.00422.56          O
ATOM    387  OE2 GLU    30       17.640   4.892  28.871
1.00410.81          O1-
ATOM    388  HN  GLU    30       16.859   3.314  24.451  1.00
15.61          H
ATOM    389  HA  GLU    30       17.504   3.016  27.339  1.00
24.60          H
ATOM    390  HB1 GLU    30       15.581   4.495  27.738  1.00
72.67          H
ATOM    391  HB2 GLU    30       15.312   4.615  25.998  1.00
56.47          H
ATOM    392  HG1 GLU    30       16.585   6.552  26.646
1.00196.66          H
ATOM    393  HG2 GLU    30       17.690   5.470  25.800
1.00140.67          H
ATOM    394  N   LYS    31       15.765   1.342  27.870  1.00
11.96          N
ATOM    395  CA  LYS    31       14.838   0.251  28.152  1.00
11.40          C
ATOM    396  C   LYS    31       13.563   0.794  28.781  1.00
10.99          C
ATOM    397  O   LYS    31       13.600   1.760  29.545  1.00
12.86          O
ATOM    398  CB  LYS    31       15.490  -0.755  29.102  1.00
21.40          C
ATOM    399  CG  LYS    31       16.696  -1.398  28.417  1.00
57.81          C
ATOM    400  CD  LYS    31       17.348  -2.404  29.369
1.00115.52          C
ATOM    401  CE  LYS    31       18.557  -3.044  28.684
1.00250.23          C
ATOM    402  NZ  LYS    31       19.197  -4.016  29.615
1.00462.10          N1+
ATOM    403  HN  LYS    31       16.423   1.599  28.551  1.00
```

```
26.13           H
ATOM    404  HA  LYS    31      14.589  -0.253  27.230  1.00
13.87           H
ATOM    405  HB1 LYS    31      14.774  -1.520  29.362  1.00
37.63           H
ATOM    406  HB2 LYS    31      15.813  -0.244  29.998  1.00
47.19           H
ATOM    407  HG1 LYS    31      17.413  -0.634  28.158
1.00126.32           H
ATOM    408  HG2 LYS    31      16.372  -1.906  27.521
1.00114.19           H
ATOM    409  HD1 LYS    31      16.634  -3.172  29.625
1.00198.45           H
ATOM    410  HD2 LYS    31      17.669  -1.895  30.265
1.00200.14           H
ATOM    411  HE1 LYS    31      19.269  -2.276  28.420
1.00374.64           H
ATOM    412  HE2 LYS    31      18.233  -3.559  27.791
1.00403.27           H
ATOM    413  HZ1 LYS    31      19.400  -4.900  29.106
1.00627.38           H
ATOM    414  HZ2 LYS    31      18.553  -4.211  30.407
1.00622.26           H
ATOM    415  HZ3 LYS    31      20.084  -3.616  29.977
1.00619.20           H
ATOM    416  N   THR    32      12.430   0.175  28.458  1.00
15.51           N
ATOM    417  CA  THR    32      11.144   0.612  29.001  1.00
22.44           C
ATOM    418  C   THR    32      10.797  -0.176  30.259  1.00
26.60           C
ATOM    419  O   THR    32      10.361  -1.326  30.185  1.00
57.43           O
ATOM    420  CB  THR    32      10.045   0.415  27.955  1.00
56.28           C
ATOM    421  CG2 THR    32       8.704   0.868  28.531
1.00102.70           C
ATOM    422  OG1 THR    32      10.351   1.182  26.799
1.00111.17           O
ATOM    423  HN  THR    32      12.457  -0.589  27.843  1.00
18.79           H
ATOM    424  HA  THR    32      11.198   1.663  29.251  1.00
19.67           H
ATOM    425  HB  THR    32       9.982  -0.629  27.688  1.00
84.25           H
ATOM    426  HG1 THR    32      10.233   2.110  27.019
1.00205.03           H
ATOM    427 HG21 THR    32       8.816   1.841  28.987
1.00220.71           H
ATOM    428 HG22 THR    32       8.372   0.158  29.275
1.00174.67           H
ATOM    429 HG23 THR    32       7.971   0.925  27.738
```

```
1.00217.44           H
ATOM    430  N   THR    33       10.982   0.459  31.413  1.00
22.47           N
ATOM    431  CA  THR    33       10.677  -0.173  32.698  1.00
41.60           C
ATOM    432  C   THR    33       10.047   0.822  33.625  1.00
38.03           C
ATOM    433  O   THR    33       10.594   1.892  33.887  1.00
52.73           O
ATOM    434  CB  THR    33       11.943  -0.733  33.337  1.00
63.25           C
ATOM    435  CG2 THR    33       11.567  -1.615  34.528
1.00121.65           C
ATOM    436  OG1 THR    33       12.656  -1.508  32.382  1.00
96.20           O
ATOM    437  HN  THR    33       11.323   1.376  31.403  1.00
23.82           H
ATOM    438  HA  THR    33        9.971  -0.968  32.564  1.00
67.12           H
ATOM    439  HB  THR    33       12.556   0.080  33.680
1.00107.59           H
ATOM    440  HG1 THR    33       12.549  -2.433  32.610
1.00176.27           H
ATOM    441 HG21 THR    33       10.969  -2.446  34.187
1.00255.23           H
ATOM    442 HG22 THR    33       11.000  -1.034  35.242
1.00245.37           H
ATOM    443 HG23 THR    33       12.465  -1.986  34.999
1.00185.78           H
ATOM    444  N   GLY    34        8.892   0.446  34.131  1.00
40.93           N
ATOM    445  CA  GLY    34        8.183   1.295  35.044  1.00
45.59           C
ATOM    446  C   GLY    34        7.660   2.539  34.333  1.00
40.97           C
ATOM    447  O   GLY    34        6.451   2.737  34.209  1.00
82.17           O
ATOM    448  HN  GLY    34        8.523  -0.426  33.889  1.00
54.00           H
ATOM    449  HA1 GLY    34        8.870   1.583  35.806  1.00
54.41           H
ATOM    450  HA2 GLY    34        7.359   0.750  35.482  1.00
62.09           H
ATOM    451  N   ASN    35        8.586   3.373  33.855  1.00
32.51           N
ATOM    452  CA  ASN    35        8.231   4.601  33.140  1.00
33.20           C
ATOM    453  C   ASN    35        8.860   4.595  31.749  1.00
23.17           C
ATOM    454  O   ASN    35        9.950   4.056  31.555  1.00
27.01           O
ATOM    455  CB  ASN    35        8.727   5.820  33.918  1.00
```

```
54.51            C
ATOM    456  CG  ASN    35        8.077   5.858  35.298  1.00
80.73            C
ATOM    457  ND2 ASN    35        8.804   6.145  36.343
1.00220.46            N
ATOM    458  OD1 ASN    35        6.878   5.618  35.426
1.00117.80            O
ATOM    459  HN  ASN    35        9.533   3.155  33.981  1.00
51.15            H
ATOM    460  HA  ASN    35        7.157   4.666  33.036  1.00
47.21            H
ATOM    461  HB1 ASN    35        8.469   6.720  33.377  1.00
68.31            H
ATOM    462  HB2 ASN    35        9.800   5.762  34.028  1.00
57.75            H
ATOM    463 HD21 ASN    35        9.759   6.335  36.239
1.00405.39            H
ATOM    464 HD22 ASN    35        8.393   6.171  37.232
1.00245.64            H
ATOM    465  N   PHE    36        8.168   5.191  30.785  1.00
35.72            N
ATOM    466  CA  PHE    36        8.666   5.245  29.420  1.00
33.41            C
ATOM    467  C   PHE    36        9.818   6.237  29.300  1.00
28.51            C
ATOM    468  O   PHE    36        9.841   7.262  29.981  1.00
45.23            O
ATOM    469  CB  PHE    36        7.531   5.653  28.483  1.00
67.53            C
ATOM    470  CG  PHE    36        6.535   4.522  28.373  1.00
86.71            C
ATOM    471  CD1 PHE    36        5.462   4.447  29.268
1.00106.51            C
ATOM    472  CD2 PHE    36        6.686   3.549  27.378  1.00
99.48            C
ATOM    473  CE1 PHE    36        4.539   3.399  29.169
1.00134.48            C
ATOM    474  CE2 PHE    36        5.764   2.502  27.277
1.00134.27            C
ATOM    475  CZ  PHE    36        4.690   2.427  28.173
1.00149.48            C
ATOM    476  HN  PHE    36        7.304   5.602  30.993  1.00
62.87            H
ATOM    477  HA  PHE    36        9.016   4.263  29.135  1.00
28.53            H
ATOM    478  HB1 PHE    36        7.933   5.873  27.510  1.00
73.56            H
ATOM    479  HB2 PHE    36        7.039   6.530  28.875  1.00
88.42            H
ATOM    480  HD1 PHE    36        5.346   5.197  30.038
1.00110.20            H
ATOM    481  HD2 PHE    36        7.515   3.607  26.687  1.00
```

```
93.07          H
ATOM    482  HE1 PHE    36        3.710   3.341  29.860
1.00155.06          H
ATOM    483  HE2 PHE    36        5.879   1.752  26.509
1.00159.46          H
ATOM    484  HZ  PHE    36        3.977   1.619  28.096
1.00183.25          H
ATOM    485  N   THR    37       10.771   5.926  28.424  1.00
20.06          N
ATOM    486  CA  THR    37       11.924   6.794  28.212  1.00
27.18          C
ATOM    487  C   THR    37       12.505   6.575  26.819  1.00
41.87          C
ATOM    488  O   THR    37       12.887   5.462  26.456
1.00169.86          O
ATOM    489  CB  THR    37       12.994   6.512  29.271  1.00
26.52          C
ATOM    490  CG2 THR    37       13.131   5.003  29.483  1.00
75.69          C
ATOM    491  OG1 THR    37       14.236   7.052  28.845  1.00
81.95          O
ATOM    492  HN  THR    37       10.696   5.099  27.907  1.00
18.47          H
ATOM    493  HA  THR    37       11.611   7.824  28.300  1.00
38.80          H
ATOM    494  HB  THR    37       12.704   6.975  30.204  1.00
81.60          H
ATOM    495  HG1 THR    37       14.065   7.640  28.104
1.00188.80          H
ATOM    496 HG21 THR    37       13.381   4.527  28.547
1.00196.98          H
ATOM    497 HG22 THR    37       12.197   4.604  29.851
1.00196.23          H
ATOM    498 HG23 THR    37       13.913   4.811  30.204
1.00168.92          H
ATOM    499  N   GLU    38       12.562   7.650  26.039  1.00
26.53          N
ATOM    500  CA  GLU    38       13.090   7.583  24.678  1.00
30.29          C
ATOM    501  C   GLU    38       14.602   7.799  24.669  1.00
25.39          C
ATOM    502  O   GLU    38       15.179   8.266  25.651  1.00
51.60          O
ATOM    503  CB  GLU    38       12.420   8.651  23.810  1.00
54.82          C
ATOM    504  CG  GLU    38       10.908   8.422  23.793
1.00165.84          C
ATOM    505  CD  GLU    38       10.587   7.108  23.090
1.00292.92          C
ATOM    506  OE1 GLU    38        9.503   6.591  23.309
1.00414.19          O
ATOM    507  OE2 GLU    38       11.430   6.637  22.343
```

```
1.00537.65          O1-
ATOM    508  HN  GLU    38      12.234   8.504  26.386  1.00
84.50          H
ATOM    509  HA  GLU    38      12.872   6.612  24.259  1.00
32.15          H
ATOM    510  HB1 GLU    38      12.804   8.588  22.802
1.00144.82          H
ATOM    511  HB2 GLU    38      12.633   9.630  24.215
1.00106.49          H
ATOM    512  HG1 GLU    38      10.428   9.234  23.266
1.00303.31          H
ATOM    513  HG2 GLU    38      10.540   8.387  24.808
1.00323.99          H
ATOM    514  N   CYS    39      15.231   7.461  23.549  1.00
15.84          N
ATOM    515  CA  CYS    39      16.675   7.625  23.407  1.00
14.30          C
ATOM    516  C   CYS    39      17.043   9.111  23.489  1.00
22.31          C
ATOM    517  O   CYS    39      16.242   9.966  23.113  1.00
32.92          O
ATOM    518  CB  CYS    39      17.121   7.054  22.058  1.00
18.08          C
ATOM    519  SG  CYS    39      16.069   7.725  20.746  1.00
17.41          S
ATOM    520  HN  CYS    39      14.716   7.102  22.799  1.00
24.87          H
ATOM    521  HA  CYS    39      17.165   7.081  24.198  1.00
14.60          H
ATOM    522  HB1 CYS    39      17.035   5.979  22.075  1.00
27.71          H
ATOM    523  HB2 CYS    39      18.148   7.329  21.871  1.00
34.91          H
ATOM    524  N   PRO    40      18.222   9.445  23.970  1.00
27.27          N
ATOM    525  CA  PRO    40      18.655  10.867  24.084  1.00
47.66          C
ATOM    526  C   PRO    40      19.034  11.456  22.727  1.00
71.54          C
ATOM    527  O   PRO    40      19.686  10.805  21.912  1.00
87.70          O
ATOM    528  CB  PRO    40      19.866  10.794  25.021  1.00
53.85          C
ATOM    529  CG  PRO    40      20.452   9.444  24.765  1.00
46.04          C
ATOM    530  CD  PRO    40      19.268   8.523  24.447  1.00
25.70          C
ATOM    531  HA  PRO    40      17.878  11.458  24.541  1.00
58.04          H
ATOM    532  HB1 PRO    40      19.551  10.870  26.051  1.00
67.93          H
ATOM    533  HB2 PRO    40      20.580  11.574  24.787  1.00
```

```
70.99           H
ATOM    534  HG1 PRO    40       20.971   9.085  25.642  1.00
65.91           H
ATOM    535  HG2 PRO    40       21.133   9.490  23.923  1.00
54.99           H
ATOM    536  HD1 PRO    40       18.937   8.010  25.337  1.00
24.22           H
ATOM    537  HD2 PRO    40       19.534   7.820  23.674  1.00
26.94           H
ATOM    538  N   GLY    41       18.622  12.697  22.501
1.00105.24           N
ATOM    539  CA  GLY    41       18.917  13.390  21.247
1.00147.55           C
ATOM    540  C   GLY    41       20.184  14.227  21.372
1.00161.74           C
ATOM    541  O   GLY    41       20.594  14.894  20.422
1.00244.37           O
ATOM    542  HN  GLY    41       18.111  13.159  23.195
1.00121.79           H
ATOM    543  HA1 GLY    41       18.091  14.040  21.000
1.00184.40           H
ATOM    544  HA2 GLY    41       19.045  12.664  20.455
1.00157.34           H
ATOM    545  N   LEU    42       20.802  14.189  22.553
1.00162.48           N
ATOM    546  CA  LEU    42       22.027  14.954  22.807
1.00191.48           C
ATOM    547  C   LEU    42       23.206  14.014  23.036
1.00166.66           C
ATOM    548  O   LEU    42       23.054  12.938  23.614
1.00251.38           O
ATOM    549  CB  LEU    42       21.838  15.840  24.040
1.00307.99           C
ATOM    550  CG  LEU    42       20.586  16.710  23.873
1.00452.28           C
ATOM    551  CD1 LEU    42       20.416  17.592  25.111
1.00681.24           C
ATOM    552  CD2 LEU    42       20.720  17.593  22.620
1.00527.46           C
ATOM    553  HN  LEU    42       20.424  13.642  23.273
1.00195.47           H
ATOM    554  HA  LEU    42       22.250  15.584  21.956
1.00229.77           H
ATOM    555  HB1 LEU    42       22.703  16.479  24.158
1.00342.49           H
ATOM    556  HB2 LEU    42       21.729  15.218  24.915
1.00335.33           H
ATOM    557  HG  LEU    42       19.721  16.069  23.771
1.00427.79           H
ATOM    558 HD11 LEU    42       19.517  18.182  25.012
1.00909.71           H
ATOM    559 HD12 LEU    42       21.269  18.248  25.207
```

1.00706.80 H
ATOM 560 HD13 LEU 42 20.342 16.969 25.991
1.00858.82 H
ATOM 561 HD21 LEU 42 21.742 17.930 22.518
1.00631.69 H
ATOM 562 HD22 LEU 42 20.068 18.449 22.708
1.00628.06 H
ATOM 563 HD23 LEU 42 20.442 17.021 21.746
1.00656.75 H
ATOM 564 N THR 43 24.386 14.434 22.579
1.00178.52 N
ATOM 565 CA THR 43 25.599 13.631 22.736
1.00240.84 C
ATOM 566 C THR 43 26.815 14.527 22.959
1.00372.31 C
ATOM 567 O THR 43 27.738 14.556 22.144
1.00500.13 O
ATOM 568 CB THR 43 25.808 12.770 21.496
1.00354.13 C
ATOM 569 CG2 THR 43 25.682 13.625 20.233
1.00463.04 C
ATOM 570 OG1 THR 43 27.094 12.167 21.548
1.00508.50 O
ATOM 571 HN THR 43 24.442 15.301 22.130
1.00229.59 H
ATOM 572 HA THR 43 25.487 12.976 23.586
1.00249.90 H
ATOM 573 HB THR 43 25.056 12.002 21.482
1.00481.58 H
ATOM 574 HG1 THR 43 27.044 11.412 22.138
1.00624.31 H
ATOM 575 HG21 THR 43 26.193 14.566 20.378
1.00650.95 H
ATOM 576 HG22 THR 43 24.639 13.813 20.028
1.00580.73 H
ATOM 577 HG23 THR 43 26.124 13.101 19.397
1.00571.99 H
ATOM 578 N PRO 44 26.828 15.249 24.048
1.00488.39 N
ATOM 579 CA PRO 44 27.950 16.167 24.400
1.00760.13 C
ATOM 580 C PRO 44 29.177 15.393 24.875
1.00735.36 C
ATOM 581 O PRO 44 30.277 15.937 24.956
1.00999.99 O
ATOM 582 CB PRO 44 27.360 17.030 25.523
1.00999.99 C
ATOM 583 CG PRO 44 26.351 16.148 26.183
1.00856.94 C
ATOM 584 CD PRO 44 25.770 15.265 25.073
1.00548.09 C
ATOM 585 HA PRO 44 28.203 16.788 23.556

```
1.00939.00            H
ATOM    586  HB1 PRO    44       26.875  17.902  25.110
1.00999.99            H
ATOM    587  HB2 PRO    44       28.132  17.323  26.224
1.00999.99            H
ATOM    588  HG1 PRO    44       25.567  16.740  26.630
1.00999.99            H
ATOM    589  HG2 PRO    44       26.832  15.536  26.939
1.00853.53            H
ATOM    590  HD1 PRO    44       24.867  15.698  24.670
1.00602.45            H
ATOM    591  HD2 PRO    44       25.584  14.264  25.441
1.00463.32            H
ATOM    592  N   ILE    45       28.971  14.119  25.191
1.00518.33            N
ATOM    593  CA  ILE    45       30.052  13.272  25.661
1.00582.79            C
ATOM    594  C   ILE    45       31.168  13.191  24.624
1.00795.93            C
ATOM    595  O   ILE    45       32.310  12.866  24.949
1.00898.75            O
ATOM    596  CB  ILE    45       29.506  11.877  25.958
1.00474.41            C
ATOM    597  CG1 ILE    45       28.816  11.313  24.709
1.00516.90            C
ATOM    598  CG2 ILE    45       28.495  11.961  27.101
1.00760.75            C
ATOM    599  CD1 ILE    45       28.472   9.838  24.934
1.00566.81            C
ATOM    600  HN  ILE    45       28.073  13.740  25.109
1.00395.72            H
ATOM    601  HA  ILE    45       30.448  13.690  26.573
1.00711.06            H
ATOM    602  HB  ILE    45       30.317  11.230  26.245
1.00477.81            H
ATOM    603 HG11 ILE    45       29.474  11.397  23.858
1.00624.28            H
ATOM    604 HG12 ILE    45       27.910  11.869  24.518
1.00744.84            H
ATOM    605 HG21 ILE    45       28.996  12.279  28.002
1.00920.57            H
ATOM    606 HG22 ILE    45       28.046  10.992  27.259
1.00999.99            H
ATOM    607 HG23 ILE    45       27.726  12.676  26.847
1.00865.46            H
ATOM    608 HD11 ILE    45       27.836   9.492  24.134
1.00658.89            H
ATOM    609 HD12 ILE    45       27.957   9.727  25.877
1.00734.30            H
ATOM    610 HD13 ILE    45       29.381   9.256  24.950
1.00675.84            H
ATOM    611  N   ALA    46       30.826  13.487  23.374
```

```
1.00999.99           N
ATOM    612  CA  ALA    46      31.804  13.445  22.295
1.00999.99           C
ATOM    613  C   ALA    46      31.276  14.175  21.063
1.00999.99           C
ATOM    614  CB  ALA    46      32.120  11.992  21.932
1.00999.99           C
ATOM    615  OT1 ALA    46      31.639  15.325  20.881
1.00999.99           O
ATOM    616  OT2 ALA    46      30.516  13.574  20.323
1.00999.99           O
ATOM    617  HN  ALA    46      29.900  13.739  23.176
1.00999.99           H
ATOM    618  HA  ALA    46      32.713  13.926  22.625
1.00999.99           H
ATOM    619  HB1 ALA    46      31.197  11.441  21.819
1.00999.99           H
ATOM    620  HB2 ALA    46      32.713  11.546  22.715
1.00999.99           H
ATOM    621  HB3 ALA    46      32.670  11.965  21.003
1.00999.99           H
ENDMDL
TER
MODEL       11
ATOM      1  N   GLY     1      27.745  -2.434  17.602
1.00999.99           N
ATOM      2  CA  GLY     1      26.306  -2.800  17.742
1.00999.99           C
ATOM      3  C   GLY     1      25.439  -1.671  17.200
1.00999.99           C
ATOM      4  O   GLY     1      24.973  -0.818  17.957
1.00999.99           O
ATOM      5  HA1 GLY     1      26.076  -2.961  18.783
1.00999.99           H
ATOM      6  HA2 GLY     1      26.111  -3.705  17.184
1.00999.99           H
ATOM      7  HT1 GLY     1      28.177  -2.356  18.543
1.00999.99           H
ATOM      8  HT2 GLY     1      27.823  -1.521  17.107
1.00999.99           H
ATOM      9  HT3 GLY     1      28.239  -3.169  17.057
1.00999.99           H
ATOM     10  N   LEU     2      25.222  -1.672  15.890
1.00999.99           N
ATOM     11  CA  LEU     2      24.406  -0.640  15.260
1.00895.59           C
ATOM     12  C   LEU     2      22.928  -0.886  15.544
1.00532.27           C
ATOM     13  O   LEU     2      22.490  -2.030  15.657
1.00625.21           O
ATOM     14  CB  LEU     2      24.641  -0.639  13.747
1.00999.99           C
```

```
ATOM     15  CG  LEU     2      26.127  -0.405  13.445
1.00999.99           C
ATOM     16  CD1 LEU     2      26.350  -0.489  11.931
1.00999.99           C
ATOM     17  CD2 LEU     2      26.562   0.981  13.956
1.00999.99           C
ATOM     18  HN  LEU     2      25.619  -2.378  15.337
1.00999.99           H
ATOM     19  HA  LEU     2      24.683   0.321  15.660
1.00890.87           H
ATOM     20  HB1 LEU     2      24.058   0.151  13.295
1.00924.07           H
ATOM     21  HB2 LEU     2      24.336  -1.590  13.335
1.00999.99           H
ATOM     22  HG  LEU     2      26.713  -1.171  13.934
1.00999.99           H
ATOM     23 HD11 LEU     2      25.900  -1.395  11.552
1.00999.99           H
ATOM     24 HD12 LEU     2      27.410  -0.499  11.724
1.00999.99           H
ATOM     25 HD13 LEU     2      25.897   0.366  11.453
1.00999.99           H
ATOM     26 HD21 LEU     2      25.753   1.688  13.829
1.00999.99           H
ATOM     27 HD22 LEU     2      27.424   1.321  13.400
1.00999.99           H
ATOM     28 HD23 LEU     2      26.819   0.913  15.002
1.00999.99           H
ATOM     29  N   CYS     3      22.164   0.196  15.665
1.00271.28           N
ATOM     30  CA  CYS     3      20.737   0.082  15.942
1.00104.50           C
ATOM     31  C   CYS     3      20.025  -0.661  14.817  1.00
90.98           C
ATOM     32  O   CYS     3      19.166  -1.506  15.064
1.00207.28           O
ATOM     33  CB  CYS     3      20.116   1.472  16.099  1.00
37.83           C
ATOM     34  SG  CYS     3      20.690   2.229  17.640  1.00
81.56           S
ATOM     35  HN  CYS     3      22.566   1.084  15.569
1.00296.55           H
ATOM     36  HA  CYS     3      20.603  -0.463  16.859
1.00166.45           H
ATOM     37  HB1 CYS     3      19.040   1.385  16.120  1.00
40.38           H
ATOM     38  HB2 CYS     3      20.410   2.092  15.264  1.00
98.45           H
ATOM     39  N   SER     4      20.385  -0.335  13.584  1.00
85.31           N
ATOM     40  CA  SER     4      19.765  -0.971  12.424  1.00
92.94           C
```

```
ATOM     41  C   SER     4      18.351  -0.432  12.221  1.00
67.37           C
ATOM     42  O   SER     4      17.891  -0.266  11.092
1.00102.28           O
ATOM     43  CB  SER     4      19.716  -2.493  12.621
1.00123.83           C
ATOM     44  OG  SER     4      19.793  -3.130  11.352
1.00178.89           O
ATOM     45  HN  SER     4      21.072   0.348  13.450
1.00163.03           H
ATOM     46  HA  SER     4      20.355  -0.751  11.546
1.00129.78           H
ATOM     47  HB1 SER     4      18.789  -2.769  13.119
1.00105.26           H
ATOM     48  HB2 SER     4      20.551  -2.806  13.225
1.00156.87           H
ATOM     49  HG  SER     4      20.567  -2.790  10.896
1.00218.16           H
ATOM     50  N   GLU     5      17.669  -0.171  13.332  1.00
41.67           N
ATOM     51  CA  GLU     5      16.305   0.339  13.295  1.00
41.21           C
ATOM     52  C   GLU     5      16.026   1.204  14.521  1.00
41.92           C
ATOM     53  O   GLU     5      16.747   1.132  15.517  1.00
74.72           O
ATOM     54  CB  GLU     5      15.323  -0.830  13.244  1.00
49.24           C
ATOM     55  CG  GLU     5      15.543  -1.712  14.465  1.00
55.48           C
ATOM     56  CD  GLU     5      14.762  -3.013  14.319  1.00
92.06           C
ATOM     57  OE1 GLU     5      14.844  -3.835  15.217
1.00206.49           O
ATOM     58  OE2 GLU     5      14.092  -3.171  13.310
1.00197.24           O1-
ATOM     59  HN  GLU     5      18.093  -0.333  14.202  1.00
45.52           H
ATOM     60  HA  GLU     5      16.176   0.934  12.412  1.00
60.63           H
ATOM     61  HB1 GLU     5      15.494  -1.407  12.349  1.00
61.59           H
ATOM     62  HB2 GLU     5      14.314  -0.454  13.245  1.00
63.66           H
ATOM     63  HG1 GLU     5      15.208  -1.191  15.348  1.00
58.03           H
ATOM     64  HG2 GLU     5      16.594  -1.928  14.552  1.00
57.77           H
ATOM     65  N   ASN     6      14.987   2.027  14.440  1.00
50.73           N
ATOM     66  CA  ASN     6      14.635   2.910  15.549  1.00
71.04           C
```

```
ATOM     67  C   ASN     6      14.285   2.109  16.800  1.00
65.17           C
ATOM     68  O   ASN     6      14.663   2.482  17.911  1.00
94.86           O
ATOM     69  CB  ASN     6      13.446   3.788  15.156  1.00
94.83           C
ATOM     70  CG  ASN     6      13.872   4.806  14.105
1.00160.94           C
ATOM     71  ND2 ASN     6      14.801   5.676  14.392
1.00244.88           N
ATOM     72  OD1 ASN     6      13.346   4.807  12.992
1.00219.73           O
ATOM     73  HN  ASN     6      14.452   2.049  13.620  1.00
72.81           H
ATOM     74  HA  ASN     6      15.478   3.548  15.768  1.00
96.02           H
ATOM     75  HB1 ASN     6      13.081   4.308  16.030
1.00111.09           H
ATOM     76  HB2 ASN     6      12.659   3.165  14.755  1.00
98.13           H
ATOM     77 HD21 ASN     6      15.218   5.673  15.278
1.00272.43           H
ATOM     78 HD22 ASN     6      15.081   6.334  13.721
1.00336.78           H
ATOM     79  N   GLY     7      13.559   1.008  16.615  1.00
51.80           N
ATOM     80  CA  GLY     7      13.157   0.156  17.738  1.00
71.47           C
ATOM     81  C   GLY     7      14.029  -1.089  17.816  1.00
41.04           C
ATOM     82  O   GLY     7      13.527  -2.206  17.945  1.00
44.54           O
ATOM     83  HN  GLY     7      13.287   0.763  15.706  1.00
45.49           H
ATOM     84  HA1 GLY     7      12.134  -0.145  17.598
1.00103.52           H
ATOM     85  HA2 GLY     7      13.239   0.708  18.665
1.00108.67           H
ATOM     86  N   ASP     8      15.335  -0.890  17.731  1.00
27.00           N
ATOM     87  CA  ASP     8      16.272  -2.007  17.784  1.00
14.15           C
ATOM     88  C   ASP     8      16.156  -2.743  19.114  1.00
9.37           C
ATOM     89  O   ASP     8      16.025  -3.966  19.150  1.00
14.61           O
ATOM     90  CB  ASP     8      17.703  -1.499  17.612  1.00
15.06           C
ATOM     91  CG  ASP     8      18.646  -2.672  17.361  1.00
21.22           C
ATOM     92  OD1 ASP     8      18.177  -3.691  16.882
1.00119.56           O
```

```
ATOM     93  OD2 ASP     8       19.821  -2.534  17.655
1.00133.27           O1-
ATOM     94  HN  ASP     8       15.675   0.021  17.623  1.00
35.63           H
ATOM     95  HA  ASP     8       16.046  -2.691  16.988  1.00
20.92           H
ATOM     96  HB1 ASP     8       18.009  -0.983  18.509  1.00
29.39           H
ATOM     97  HB2 ASP     8       17.741  -0.816  16.776  1.00
44.14           H
ATOM     98  N   CYS     9       16.202  -1.987  20.199  1.00
5.93           N
ATOM     99  CA  CYS     9       16.095  -2.575  21.528  1.00
7.54           C
ATOM    100  C   CYS     9       14.723  -3.205  21.715  1.00
14.60           C
ATOM    101  O   CYS     9       14.591  -4.296  22.272  1.00
25.78           O
ATOM    102  CB  CYS     9       16.318  -1.508  22.590  1.00
6.63           C
ATOM    103  SG  CYS     9       18.048  -1.016  22.554  1.00
10.91           S
ATOM    104  HN  CYS     9       16.304  -1.018  20.105  1.00
6.51           H
ATOM    105  HA  CYS     9       16.859  -3.327  21.639  1.00
11.12           H
ATOM    106  HB1 CYS     9       16.080  -1.907  23.561  1.00
11.28           H
ATOM    107  HB2 CYS     9       15.695  -0.650  22.385  1.00
5.04           H
ATOM    108  N   ALA    10       13.706  -2.502  21.234  1.00
16.26           N
ATOM    109  CA  ALA    10       12.329  -2.975  21.330  1.00
32.04           C
ATOM    110  C   ALA    10       11.408  -2.056  20.531  1.00
45.47           C
ATOM    111  O   ALA    10       11.818  -0.979  20.098
1.00119.04           O
ATOM    112  CB  ALA    10       11.886  -3.020  22.796  1.00
30.10           C
ATOM    113  HN  ALA    10       13.885  -1.644  20.799  1.00
12.81           H
ATOM    114  HA  ALA    10       12.272  -3.972  20.917  1.00
47.14           H
ATOM    115  HB1 ALA    10       11.589  -2.032  23.115  1.00
84.37           H
ATOM    116  HB2 ALA    10       12.705  -3.363  23.409
1.00118.20           H
ATOM    117  HB3 ALA    10       11.052  -3.697  22.898
1.00117.32           H
ATOM    118  N   ALA    11       10.167  -2.486  20.334  1.00
30.62           N
```

```
ATOM    119  CA  ALA    11        9.207  -1.685  19.580  1.00
35.24           C
ATOM    120  C   ALA    11        8.967  -0.339  20.259  1.00
22.78           C
ATOM    121  O   ALA    11        8.877   0.693  19.594  1.00
53.33           O
ATOM    122  CB  ALA    11        7.883  -2.440  19.457  1.00
60.49           C
ATOM    123  HN  ALA    11        9.893  -3.353  20.697  1.00
50.37           H
ATOM    124  HA  ALA    11        9.601  -1.511  18.589  1.00
41.70           H
ATOM    125  HB1 ALA    11        7.248  -1.936  18.743
1.00157.06           H
ATOM    126  HB2 ALA    11        7.395  -2.468  20.419
1.00148.83           H
ATOM    127  HB3 ALA    11        8.075  -3.449  19.119
1.00137.06           H
ATOM    128  N   ASP    12        8.854  -0.355  21.585  1.00
18.98           N
ATOM    129  CA  ASP    12        8.613   0.872  22.343  1.00
31.05           C
ATOM    130  C   ASP    12        9.922   1.604  22.636  1.00
22.98           C
ATOM    131  O   ASP    12        9.949   2.831  22.728  1.00
37.14           O
ATOM    132  CB  ASP    12        7.912   0.538  23.662  1.00
48.35           C
ATOM    133  CG  ASP    12        6.493   0.054  23.388
1.00104.83           C
ATOM    134  OD1 ASP    12        6.021   0.258  22.283
1.00258.73           O
ATOM    135  OD2 ASP    12        5.898  -0.514  24.291
1.00213.46           O1-
ATOM    136  HN  ASP    12        8.929  -1.208  22.063  1.00
36.19           H
ATOM    137  HA  ASP    12        7.971   1.523  21.767  1.00
49.76           H
ATOM    138  HB1 ASP    12        7.877   1.421  24.283  1.00
95.71           H
ATOM    139  HB2 ASP    12        8.464  -0.237  24.173  1.00
54.37           H
ATOM    140  N   GLU    13       11.001   0.844  22.789  1.00
17.60           N
ATOM    141  CA  GLU    13       12.306   1.431  23.081  1.00
12.01           C
ATOM    142  C   GLU    13       12.901   2.087  21.839  1.00
10.07           C
ATOM    143  O   GLU    13       12.553   1.736  20.711  1.00
12.81           O
ATOM    144  CB  GLU    13       13.249   0.349  23.607  1.00
11.74           C
```

ATOM 145 CG GLU 13 12.710 -0.184 24.937 1.00
13.49 C
ATOM 146 CD GLU 13 13.502 -1.414 25.370
1.00142.36 C
ATOM 147 OE1 GLU 13 12.878 -2.410 25.697
1.00339.06 O
ATOM 148 OE2 GLU 13 14.718 -1.341 25.371
1.00335.28 O1-
ATOM 149 HN GLU 13 10.918 -0.129 22.711 1.00
28.36 H
ATOM 150 HA GLU 13 12.184 2.184 23.847 1.00
13.06 H
ATOM 151 HB1 GLU 13 14.231 0.770 23.761 1.00
13.76 H
ATOM 152 HB2 GLU 13 13.309 -0.457 22.890 1.00
9.94 H
ATOM 153 HG1 GLU 13 11.670 -0.449 24.823 1.00
66.02 H
ATOM 154 HG2 GLU 13 12.803 0.584 25.691 1.00
56.23 H
ATOM 155 N CYS 14 13.803 3.047 22.056 1.00
9.12 N
ATOM 156 CA CYS 14 14.452 3.766 20.954 1.00
10.69 C
ATOM 157 C CYS 14 15.937 3.432 20.900 1.00
9.56 C
ATOM 158 O CYS 14 16.578 3.251 21.933 1.00
13.62 O
ATOM 159 CB CYS 14 14.286 5.275 21.147 1.00
13.96 C
ATOM 160 SG CYS 14 15.138 6.148 19.807 1.00
42.96 S
ATOM 161 HN CYS 14 14.039 3.281 22.979 1.00
9.76 H
ATOM 162 HA CYS 14 13.997 3.483 20.015 1.00
15.54 H
ATOM 163 HB1 CYS 14 14.712 5.564 22.094 1.00
50.72 H
ATOM 164 HB2 CYS 14 13.237 5.527 21.131 1.00
46.13 H
ATOM 165 N CYS 15 16.480 3.360 19.686 1.00
9.60 N
ATOM 166 CA CYS 15 17.897 3.056 19.491 1.00
9.28 C
ATOM 167 C CYS 15 18.456 3.985 18.426 1.00
9.92 C
ATOM 168 O CYS 15 17.898 4.094 17.334 1.00
13.31 O
ATOM 169 CB CYS 15 18.064 1.601 19.045 1.00
12.90 C
ATOM 170 SG CYS 15 19.816 1.136 19.097 1.00
39.11 S

ATOM 171 HN CYS 15 15.917 3.526 18.900 1.00
13.12 H
ATOM 172 HA CYS 15 18.437 3.209 20.415 1.00
8.26 H
ATOM 173 HB1 CYS 15 17.692 1.488 18.037 1.00
24.82 H
ATOM 174 HB2 CYS 15 17.503 0.958 19.708 1.00
27.31 H
ATOM 175 N VAL 16 19.548 4.673 18.747 1.00
9.56 N
ATOM 176 CA VAL 16 20.152 5.609 17.802 1.00
11.99 C
ATOM 177 C VAL 16 21.671 5.480 17.807 1.00
7.73 C
ATOM 178 O VAL 16 22.305 5.486 18.862 1.00
8.83 O
ATOM 179 CB VAL 16 19.742 7.035 18.176 1.00
18.37 C
ATOM 180 CG1 VAL 16 20.444 7.461 19.468 1.00
39.61 C
ATOM 181 CG2 VAL 16 20.119 7.985 17.042
1.00115.21 C
ATOM 182 HN VAL 16 19.947 4.560 19.637 1.00
9.76 H
ATOM 183 HA VAL 16 19.793 5.398 16.803 1.00
17.27 H
ATOM 184 HB VAL 16 18.671 7.067 18.328 1.00
52.63 H
ATOM 185 HG11 VAL 16 21.485 7.661 19.263
1.00128.07 H
ATOM 186 HG12 VAL 16 20.367 6.669 20.199
1.00154.13 H
ATOM 187 HG13 VAL 16 19.976 8.354 19.855
1.00135.74 H
ATOM 188 HG21 VAL 16 19.471 7.807 16.196
1.00229.05 H
ATOM 189 HG22 VAL 16 21.144 7.812 16.753
1.00261.82 H
ATOM 190 HG23 VAL 16 20.005 9.005 17.374
1.00210.07 H
ATOM 191 N ASP 17 22.247 5.357 16.612 1.00
14.34 N
ATOM 192 CA ASP 17 23.694 5.220 16.470 1.00
11.90 C
ATOM 193 C ASP 17 24.350 6.579 16.248 1.00
13.15 C
ATOM 194 O ASP 17 23.847 7.408 15.491 1.00
25.96 O
ATOM 195 CB ASP 17 24.013 4.316 15.277 1.00
20.80 C
ATOM 196 CG ASP 17 23.611 2.879 15.583 1.00
28.72 C

```
ATOM    197  OD1 ASP    17       23.392   2.579  16.746
1.00135.42             O
ATOM    198  OD2 ASP    17       23.524   2.099  14.649
1.00114.17             O1-
ATOM    199  HN  ASP    17       21.684   5.353  15.810  1.00
28.29           H
ATOM    200  HA  ASP    17       24.105   4.772  17.364  1.00
10.49           H
ATOM    201  HB1 ASP    17       25.075   4.354  15.074  1.00
22.42           H
ATOM    202  HB2 ASP    17       23.470   4.662  14.410  1.00
29.52           H
ATOM    203  N   THR    18       25.490   6.788  16.903  1.00
11.55           N
ATOM    204  CA  THR    18       26.244   8.035  16.771  1.00
17.10           C
ATOM    205  C   THR    18       27.692   7.728  16.424  1.00
10.63           C
ATOM    206  O   THR    18       28.174   6.625  16.673  1.00
6.49           O
ATOM    207  CB  THR    18       26.191   8.832  18.076  1.00
28.52           C
ATOM    208  CG2 THR    18       24.803   9.438  18.259  1.00
45.38           C
ATOM    209  OG1 THR    18       26.488   7.975  19.164  1.00
26.49           O
ATOM    210  HN  THR    18       25.843   6.077  17.479  1.00
13.96           H
ATOM    211  HA  THR    18       25.817   8.635  15.979  1.00
26.43           H
ATOM    212  HB  THR    18       26.920   9.627  18.038  1.00
37.43           H
ATOM    213  HG1 THR    18       25.686   7.859  19.679  1.00
71.62           H
ATOM    214 HG21 THR    18       24.052   8.697  18.032
1.00100.44             H
ATOM    215 HG22 THR    18       24.692  10.282  17.594
1.00129.42             H
ATOM    216 HG23 THR    18       24.689   9.768  19.281
1.00156.21             H
ATOM    217  N   VAL    19       28.377   8.715  15.865  1.00
16.32           N
ATOM    218  CA  VAL    19       29.763   8.573  15.489  1.00
13.34           C
ATOM    219  C   VAL    19       30.228   9.866  14.853  1.00
25.88           C
ATOM    220  O   VAL    19       29.687  10.328  13.848  1.00
42.11           O
ATOM    221  CB  VAL    19       29.975   7.401  14.534  1.00
15.48           C
ATOM    222  CG1 VAL    19       28.973   7.477  13.378  1.00
29.06           C
```

```
ATOM    223  CG2 VAL    19       31.404   7.460  13.981  1.00
20.67           C
ATOM    224  HN  VAL    19       27.947   9.575  15.712  1.00
26.89           H
ATOM    225  HA  VAL    19       30.348   8.402  16.385  1.00
8.08            H
ATOM    226  HB  VAL    19       29.836   6.478  15.075  1.00
11.49           H
ATOM    227 HG11 VAL    19       27.976   7.613  13.771
1.00137.43           H
ATOM    228 HG12 VAL    19       29.012   6.561  12.808  1.00
93.29           H
ATOM    229 HG13 VAL    19       29.224   8.309  12.738
1.00108.69           H
ATOM    230 HG21 VAL    19       31.661   6.510  13.538  1.00
99.42           H
ATOM    231 HG22 VAL    19       32.091   7.684  14.787
1.00111.36           H
ATOM    232 HG23 VAL    19       31.467   8.238  13.233  1.00
77.11           H
ATOM    233  N   PHE    20       31.207  10.449  15.491  1.00
24.58           N
ATOM    234  CA  PHE    20       31.773  11.723  15.076  1.00
40.34           C
ATOM    235  C   PHE    20       33.057  11.547  14.264  1.00
40.37           C
ATOM    236  O   PHE    20       33.018  11.430  13.039  1.00
66.02           O
ATOM    237  CB  PHE    20       32.044  12.566  16.335  1.00
48.79           C
ATOM    238  CG  PHE    20       32.406  11.671  17.513  1.00
35.75           C
ATOM    239  CD1 PHE    20       31.433  10.841  18.106  1.00
30.20           C
ATOM    240  CD2 PHE    20       33.709  11.686  18.027  1.00
40.95           C
ATOM    241  CE1 PHE    20       31.773  10.035  19.196  1.00
30.41           C
ATOM    242  CE2 PHE    20       34.045  10.875  19.117  1.00
45.84           C
ATOM    243  CZ  PHE    20       33.077  10.050  19.702  1.00
40.81           C
ATOM    244  HN  PHE    20       31.539  10.022  16.297  1.00
16.73           H
ATOM    245  HA  PHE    20       31.052  12.247  14.461  1.00
59.91           H
ATOM    246  HB1 PHE    20       31.157  13.117  16.580  1.00
67.15           H
ATOM    247  HB2 PHE    20       32.850  13.261  16.148  1.00
58.17           H
ATOM    248  HD1 PHE    20       30.423  10.813  17.717  1.00
33.34           H
```

ATOM 249 HD2 PHE 20 34.458 12.320 17.580 1.00
49.35 H
ATOM 250 HE1 PHE 20 31.025 9.402 19.648 1.00
30.61 H
ATOM 251 HE2 PHE 20 35.052 10.886 19.508 1.00
61.27 H
ATOM 252 HZ PHE 20 33.336 9.426 20.545 1.00
52.66 H
ATOM 253 N GLU 21 34.189 11.565 14.953 1.00
41.31 N
ATOM 254 CA GLU 21 35.483 11.446 14.291 1.00
56.16 C
ATOM 255 C GLU 21 35.694 10.030 13.760 1.00
58.61 C
ATOM 256 O GLU 21 36.304 9.829 12.710
1.00201.19 O
ATOM 257 CB GLU 21 36.575 11.856 15.291 1.00
57.37 C
ATOM 258 CG GLU 21 37.102 10.653 16.077
1.00193.56 C
ATOM 259 CD GLU 21 37.879 11.130 17.300
1.00304.62 C
ATOM 260 OE1 GLU 21 37.248 11.599 18.233
1.00451.81 O
ATOM 261 OE2 GLU 21 39.094 11.021 17.286
1.00442.88 O1-
ATOM 262 HN GLU 21 34.156 11.682 15.925 1.00
50.82 H
ATOM 263 HA GLU 21 35.504 12.132 13.456 1.00
85.72 H
ATOM 264 HB1 GLU 21 36.137 12.554 15.984 1.00
46.37 H
ATOM 265 HB2 GLU 21 37.393 12.331 14.769
1.00131.81 H
ATOM 266 HG1 GLU 21 37.758 10.075 15.444
1.00314.91 H
ATOM 267 HG2 GLU 21 36.271 10.043 16.398
1.00266.92 H
ATOM 268 N GLY 22 35.162 9.060 14.490 1.00
86.70 N
ATOM 269 CA GLY 22 35.265 7.646 14.095
1.00104.66 C
ATOM 270 C GLY 22 36.272 6.888 14.953 1.00
71.50 C
ATOM 271 O GLY 22 36.328 5.659 14.915 1.00
89.87 O
ATOM 272 HN GLY 22 34.683 9.302 15.313
1.00214.64 H
ATOM 273 HA1 GLY 22 35.570 7.581 13.059
1.00152.47 H
ATOM 274 HA2 GLY 22 34.296 7.183 14.204
1.00118.15 H

```
ATOM    275  N   ASP    23       37.053   7.619  15.732  1.00
44.68          N
ATOM    276  CA  ASP    23       38.043   6.999  16.603  1.00
43.33          C
ATOM    277  C   ASP    23       37.343   6.074  17.589  1.00
37.55          C
ATOM    278  O   ASP    23       37.793   4.958  17.844  1.00
61.37          O
ATOM    279  CB  ASP    23       38.813   8.078  17.364  1.00
40.74          C
ATOM    280  CG  ASP    23       39.895   7.436  18.226
1.00141.86          C
ATOM    281  OD1 ASP    23       40.828   6.889  17.661
1.00328.18          O
ATOM    282  OD2 ASP    23       39.776   7.501  19.437
1.00304.58          O1-
ATOM    283  HN  ASP    23       36.958   8.588  15.728  1.00
40.86          H
ATOM    284  HA  ASP    23       38.732   6.425  16.005  1.00
72.12          H
ATOM    285  HB1 ASP    23       38.131   8.627  17.997  1.00
94.15          H
ATOM    286  HB2 ASP    23       39.272   8.756  16.658  1.00
93.26          H
ATOM    287  N   MET    24       36.226   6.554  18.123  1.00
23.24          N
ATOM    288  CA  MET    24       35.427   5.785  19.075  1.00
32.50          C
ATOM    289  C   MET    24       33.948   5.965  18.753  1.00
25.13          C
ATOM    290  O   MET    24       33.420   7.074  18.832  1.00
54.40          O
ATOM    291  CB  MET    24       35.707   6.268  20.500  1.00
47.36          C
ATOM    292  CG  MET    24       34.871   5.461  21.495
1.00151.73          C
ATOM    293  SD  MET    24       35.311   5.942  23.185
1.00209.23          S
ATOM    294  CE  MET    24       34.599   7.607  23.157
1.00243.29          C
ATOM    295  HN  MET    24       35.924   7.448  17.863  1.00
17.32          H
ATOM    296  HA  MET    24       35.679   4.736  19.001  1.00
48.94          H
ATOM    297  HB1 MET    24       35.449   7.314  20.581
1.00124.40          H
ATOM    298  HB2 MET    24       36.756   6.140  20.724
1.00166.96          H
ATOM    299  HG1 MET    24       35.069   4.408  21.360
1.00331.71          H
ATOM    300  HG2 MET    24       33.821   5.651  21.328
1.00302.30          H
```

```
ATOM    301  HE1 MET    24       35.301   8.292  22.702
1.00340.44            H
ATOM    302  HE2 MET    24       34.392   7.928  24.166
1.00373.88            H
ATOM    303  HE3 MET    24       33.678   7.599  22.592
1.00386.81            H
ATOM    304  N   VAL    25       33.282   4.877  18.383  1.00
22.44           N
ATOM    305  CA  VAL    25       31.865   4.948  18.047  1.00
15.37           C
ATOM    306  C   VAL    25       31.017   5.056  19.306  1.00
17.47           C
ATOM    307  O   VAL    25       31.387   4.560  20.370  1.00
29.52           O
ATOM    308  CB  VAL    25       31.432   3.724  17.242  1.00
23.36           C
ATOM    309  CG1 VAL    25       29.926   3.806  16.975  1.00
58.39           C
ATOM    310  CG2 VAL    25       32.186   3.699  15.909  1.00
55.25           C
ATOM    311  HN  VAL    25       33.750   4.017  18.331  1.00
48.11           H
ATOM    312  HA  VAL    25       31.699   5.833  17.446  1.00
9.25           H
ATOM    313  HB  VAL    25       31.653   2.827  17.802  1.00
52.45           H
ATOM    314 HG11 VAL    25       29.665   4.818  16.694
1.00171.07            H
ATOM    315 HG12 VAL    25       29.388   3.533  17.870
1.00166.22            H
ATOM    316 HG13 VAL    25       29.661   3.130  16.176
1.00134.76            H
ATOM    317 HG21 VAL    25       32.146   4.677  15.455
1.00141.88            H
ATOM    318 HG22 VAL    25       31.725   2.977  15.250
1.00184.50            H
ATOM    319 HG23 VAL    25       33.215   3.422  16.082
1.00133.90            H
ATOM    320  N   THR    26       29.877   5.728  19.170  1.00
13.60           N
ATOM    321  CA  THR    26       28.958   5.935  20.286  1.00
21.19           C
ATOM    322  C   THR    26       27.538   5.518  19.908  1.00
16.55           C
ATOM    323  O   THR    26       27.097   5.730  18.779  1.00
11.43           O
ATOM    324  CB  THR    26       28.981   7.413  20.671  1.00
27.07           C
ATOM    325  CG2 THR    26       27.970   7.682  21.789  1.00
44.39           C
ATOM    326  OG1 THR    26       30.285   7.753  21.120  1.00
34.75           O
```

ATOM 327 HN THR 26 29.652 6.106 18.295 1.00
10.81 H
ATOM 328 HA THR 26 29.279 5.350 21.138 1.00
33.16 H
ATOM 329 HB THR 26 28.733 8.007 19.802 1.00
20.60 H
ATOM 330 HG1 THR 26 30.790 6.940 21.207 1.00
77.72 H
ATOM 331 HG21 THR 26 28.131 6.981 22.595
1.00126.99 H
ATOM 332 HG22 THR 26 26.967 7.566 21.409
1.00108.32 H
ATOM 333 HG23 THR 26 28.101 8.688 22.156
1.00151.99 H
ATOM 334 N ARG 27 26.828 4.923 20.867 1.00
22.38 N
ATOM 335 CA ARG 27 25.452 4.473 20.642 1.00
20.21 C
ATOM 336 C ARG 27 24.630 4.645 21.917 1.00
16.96 C
ATOM 337 O ARG 27 25.187 4.795 23.005 1.00
20.06 O
ATOM 338 CB ARG 27 25.445 3.003 20.212 1.00
22.14 C
ATOM 339 CG ARG 27 25.954 2.126 21.358
1.00124.30 C
ATOM 340 CD ARG 27 26.133 0.691 20.862
1.00109.38 C
ATOM 341 NE ARG 27 26.564 -0.172 21.958
1.00227.73 N
ATOM 342 CZ ARG 27 27.839 -0.225 22.332
1.00426.12 C
ATOM 343 NH1 ARG 27 28.198 -1.004 23.314
1.00767.09 N1+
ATOM 344 NH2 ARG 27 28.730 0.504 21.717
1.00581.78 N
ATOM 345 HN ARG 27 27.237 4.783 21.747 1.00
30.75 H
ATOM 346 HA ARG 27 25.005 5.069 19.858 1.00
23.13 H
ATOM 347 HB1 ARG 27 26.088 2.877 19.354 1.00
87.46 H
ATOM 348 HB2 ARG 27 24.438 2.709 19.954
1.00103.36 H
ATOM 349 HG1 ARG 27 25.238 2.136 22.167
1.00281.97 H
ATOM 350 HG2 ARG 27 26.902 2.507 21.711
1.00276.19 H
ATOM 351 HD1 ARG 27 26.877 0.675 20.078
1.00183.60 H
ATOM 352 HD2 ARG 27 25.195 0.328 20.472
1.00142.93 H

```
ATOM    353  HE  ARG    27      25.902  -0.723  22.425
1.00372.53            H
ATOM    354 HH11 ARG    27      27.515  -1.563  23.786
1.00910.59            H
ATOM    355 HH12 ARG    27      29.157  -1.044  23.597
1.00999.99            H
ATOM    356 HH21 ARG    27      28.454   1.100  20.964
1.00532.54            H
ATOM    357 HH22 ARG    27      29.689   0.463  22.000
1.00948.84            H
ATOM    358  N   SER    28      23.304   4.632  21.783  1.00
14.60           N
ATOM    359  CA  SER    28      22.425   4.799  22.941  1.00
14.92           C
ATOM    360  C   SER    28      21.110   4.052  22.741  1.00
12.12           C
ATOM    361  O   SER    28      20.663   3.848  21.613  1.00
13.41           O
ATOM    362  CB  SER    28      22.140   6.283  23.169  1.00
23.26           C
ATOM    363  OG  SER    28      21.317   6.429  24.318
1.00146.32            O
ATOM    364  HN  SER    28      22.909   4.517  20.892  1.00
15.23           H
ATOM    365  HA  SER    28      22.916   4.403  23.819  1.00
15.44           H
ATOM    366  HB1 SER    28      21.641   6.690  22.300  1.00
88.73           H
ATOM    367  HB2 SER    28      23.066   6.810  23.326
1.00124.68            H
ATOM    368  HG  SER    28      21.121   5.553  24.657
1.00242.47            H
ATOM    369  N   CYS    29      20.495   3.653  23.851  1.00
10.48           N
ATOM    370  CA  CYS    29      19.228   2.934  23.805  1.00
9.97           C
ATOM    371  C   CYS    29      18.633   2.862  25.211  1.00
11.60           C
ATOM    372  O   CYS    29      19.328   2.524  26.170  1.00
15.64           O
ATOM    373  CB  CYS    29      19.443   1.522  23.236  1.00
9.52           C
ATOM    374  SG  CYS    29      17.965   0.985  22.362  1.00
10.40           S
ATOM    375  HN  CYS    29      20.898   3.856  24.720  1.00
11.10           H
ATOM    376  HA  CYS    29      18.546   3.474  23.165  1.00
11.08           H
ATOM    377  HB1 CYS    29      19.646   0.821  24.036  1.00
9.33           H
ATOM    378  HB2 CYS    29      20.275   1.529  22.552  1.00
10.78           H
```

ATOM 379 N GLU 30 17.349 3.196 25.333 1.00
13.52 N
ATOM 380 CA GLU 30 16.676 3.182 26.635 1.00
17.41 C
ATOM 381 C GLU 30 15.789 1.951 26.788 1.00
10.81 C
ATOM 382 O GLU 30 15.245 1.437 25.810 1.00
23.22 O
ATOM 383 CB GLU 30 15.829 4.445 26.783 1.00
37.83 C
ATOM 384 CG GLU 30 16.739 5.672 26.717
1.00108.13 C
ATOM 385 CD GLU 30 17.586 5.760 27.982
1.00240.52 C
ATOM 386 OE1 GLU 30 17.270 5.064 28.933
1.00422.56 O
ATOM 387 OE2 GLU 30 18.538 6.523 27.982
1.00410.81 O1-
ATOM 388 HN GLU 30 16.846 3.469 24.537 1.00
15.61 H
ATOM 389 HA GLU 30 17.416 3.174 27.424 1.00
24.60 H
ATOM 390 HB1 GLU 30 15.317 4.428 27.733 1.00
72.67 H
ATOM 391 HB2 GLU 30 15.104 4.487 25.982 1.00
56.47 H
ATOM 392 HG1 GLU 30 16.137 6.563 26.627
1.00196.66 H
ATOM 393 HG2 GLU 30 17.390 5.586 25.859
1.00140.67 H
ATOM 394 N LYS 31 15.650 1.487 28.030 1.00
11.96 N
ATOM 395 CA LYS 31 14.826 0.312 28.330 1.00
11.40 C
ATOM 396 C LYS 31 13.544 0.732 29.042 1.00
10.99 C
ATOM 397 O LYS 31 13.538 1.691 29.814 1.00
12.86 O
ATOM 398 CB LYS 31 15.609 -0.653 29.222 1.00
21.40 C
ATOM 399 CG LYS 31 16.832 -1.171 28.464 1.00
57.81 C
ATOM 400 CD LYS 31 17.617 -2.137 29.354
1.00115.52 C
ATOM 401 CE LYS 31 18.841 -2.652 28.596
1.00250.23 C
ATOM 402 NZ LYS 31 19.608 -3.586 29.468
1.00462.10 N1+
ATOM 403 HN LYS 31 16.110 1.945 28.764 1.00
26.13 H
ATOM 404 HA LYS 31 14.567 -0.195 27.413 1.00
13.87 H

```
ATOM    405  HB1 LYS    31      14.979  -1.486  29.493  1.00
37.63           H
ATOM    406  HB2 LYS    31      15.930  -0.138  30.116  1.00
47.19           H
ATOM    407  HG1 LYS    31      17.468  -0.340  28.192
1.00126.32           H
ATOM    408  HG2 LYS    31      16.511  -1.685  27.570
1.00114.19           H
ATOM    409  HD1 LYS    31      16.985  -2.970  29.622
1.00198.45           H
ATOM    410  HD2 LYS    31      17.935  -1.624  30.249
1.00200.14           H
ATOM    411  HE1 LYS    31      19.470  -1.818  28.321
1.00374.64           H
ATOM    412  HE2 LYS    31      18.522  -3.171  27.705
1.00403.27           H
ATOM    413  HZ1 LYS    31      20.627  -3.419  29.344
1.00627.38           H
ATOM    414  HZ2 LYS    31      19.383  -4.568  29.205
1.00622.26           H
ATOM    415  HZ3 LYS    31      19.352  -3.425  30.462
1.00619.20           H
ATOM    416  N   THR    32      12.459   0.009  28.775  1.00
15.51           N
ATOM    417  CA  THR    32      11.167   0.312  29.391  1.00
22.44           C
ATOM    418  C   THR    32      10.941  -0.560  30.624  1.00
26.60           C
ATOM    419  O   THR    32      10.953  -1.788  30.538  1.00
57.43           O
ATOM    420  CB  THR    32      10.042   0.066  28.384  1.00
56.28           C
ATOM    421  CG2 THR    32       8.692   0.369  29.038
1.00102.70           C
ATOM    422  OG1 THR    32      10.225   0.912  27.258
1.00111.17           O
ATOM    423  HN  THR    32      12.526  -0.742  28.150  1.00
18.79           H
ATOM    424  HA  THR    32      11.143   1.352  29.689  1.00
19.67           H
ATOM    425  HB  THR    32      10.060  -0.965  28.067  1.00
84.25           H
ATOM    426  HG1 THR    32      10.449   0.360  26.505
1.00205.03           H
ATOM    427 HG21 THR    32       7.923   0.395  28.281
1.00220.71           H
ATOM    428 HG22 THR    32       8.741   1.326  29.535
1.00174.67           H
ATOM    429 HG23 THR    32       8.462  -0.401  29.759
1.00217.44           H
ATOM    430  N   THR    33      10.733   0.086  31.768  1.00
22.47           N
```

ATOM 431 CA THR 33 10.498 -0.632 33.020 1.00
41.60 C
ATOM 432 C THR 33 9.622 0.175 33.936 1.00
38.03 C
ATOM 433 O THR 33 10.098 0.889 34.819 1.00
52.73 O
ATOM 434 CB THR 33 11.818 -0.953 33.716 1.00
63.25 C
ATOM 435 CG2 THR 33 12.578 -2.012 32.917
1.00121.65 C
ATOM 436 OG1 THR 33 12.601 0.228 33.813 1.00
96.20 O
ATOM 437 HN THR 33 10.731 1.067 31.772 1.00
23.82 H
ATOM 438 HA THR 33 9.976 -1.547 32.818 1.00
67.12 H
ATOM 439 HB THR 33 11.612 -1.333 34.704
1.00107.59 H
ATOM 440 HG1 THR 33 13.210 0.243 33.070
1.00176.27 H
ATOM 441 HG21 THR 33 13.428 -2.354 33.490
1.00255.23 H
ATOM 442 HG22 THR 33 12.921 -1.584 31.987
1.00245.37 H
ATOM 443 HG23 THR 33 11.925 -2.846 32.711
1.00185.78 H
ATOM 444 N GLY 34 8.329 0.036 33.723 1.00
40.93 N
ATOM 445 CA GLY 34 7.363 0.732 34.536 1.00
45.59 C
ATOM 446 C GLY 34 7.245 2.188 34.105 1.00
40.97 C
ATOM 447 O GLY 34 6.416 2.937 34.619 1.00
82.17 O
ATOM 448 HN GLY 34 8.022 -0.562 33.012 1.00
54.00 H
ATOM 449 HA1 GLY 34 7.692 0.684 35.556 1.00
54.41 H
ATOM 450 HA2 GLY 34 6.400 0.247 34.445 1.00
62.09 H
ATOM 451 N ASN 35 8.082 2.574 33.147 1.00
32.51 N
ATOM 452 CA ASN 35 8.077 3.937 32.629 1.00
33.20 C
ATOM 453 C ASN 35 8.704 3.972 31.241 1.00
23.17 C
ATOM 454 O ASN 35 9.582 3.167 30.928 1.00
27.01 O
ATOM 455 CB ASN 35 8.858 4.862 33.565 1.00
54.51 C
ATOM 456 CG ASN 35 8.295 4.784 34.973 1.00
80.73 C

```
ATOM    457  ND2 ASN    35       7.083   5.190  35.198
1.00220.46           N
ATOM    458  OD1 ASN    35       8.982   4.345  35.895
1.00117.80           O
ATOM    459  HN  ASN    35       8.715   1.925  32.776  1.00
51.15           H
ATOM    460  HA  ASN    35       7.057   4.287  32.564  1.00
47.21           H
ATOM    461  HB1 ASN    35       8.786   5.878  33.206  1.00
68.31           H
ATOM    462  HB2 ASN    35       9.889   4.562  33.586  1.00
57.75           H
ATOM    463 HD21 ASN    35       6.538   5.542  34.464
1.00405.39           H
ATOM    464 HD22 ASN    35       6.719   5.142  36.099
1.00245.64           H
ATOM    465  N   PHE    36       8.248   4.902  30.407  1.00
35.72           N
ATOM    466  CA  PHE    36       8.760   5.033  29.052  1.00
33.41           C
ATOM    467  C   PHE    36       9.815   6.131  28.976  1.00
28.51           C
ATOM    468  O   PHE    36       9.670   7.191  29.584  1.00
45.23           O
ATOM    469  CB  PHE    36       7.601   5.369  28.123  1.00
67.53           C
ATOM    470  CG  PHE    36       6.645   4.203  28.083  1.00
86.71           C
ATOM    471  CD1 PHE    36       5.543   4.174  28.947
1.00106.51           C
ATOM    472  CD2 PHE    36       6.858   3.150  27.186  1.00
99.48           C
ATOM    473  CE1 PHE    36       4.654   3.093  28.913
1.00134.48           C
ATOM    474  CE2 PHE    36       5.971   2.069  27.152
1.00134.27           C
ATOM    475  CZ  PHE    36       4.869   2.040  28.015
1.00149.48           C
ATOM    476  HN  PHE    36       7.543   5.513  30.704  1.00
62.87           H
ATOM    477  HA  PHE    36       9.199   4.097  28.735  1.00
28.53           H
ATOM    478  HB1 PHE    36       7.978   5.560  27.139  1.00
73.56           H
ATOM    479  HB2 PHE    36       7.087   6.246  28.490  1.00
88.42           H
ATOM    480  HD1 PHE    36       5.381   4.987  29.641
1.00110.20           H
ATOM    481  HD2 PHE    36       7.711   3.172  26.522  1.00
93.07           H
ATOM    482  HE1 PHE    36       3.805   3.071  29.578
1.00155.06           H
```

```
ATOM    483  HE2 PHE    36       6.135   1.256  26.460
1.00159.46          H
ATOM    484  HZ  PHE    36       4.183   1.206  27.988
1.00183.25          H
ATOM    485  N   THR    37      10.880   5.868  28.219  1.00
20.06           N
ATOM    486  CA  THR    37      11.963   6.835  28.056  1.00
27.18           C
ATOM    487  C   THR    37      12.563   6.718  26.661  1.00
41.87           C
ATOM    488  O   THR    37      13.097   5.675  26.286
1.00169.86          O
ATOM    489  CB  THR    37      13.049   6.585  29.106  1.00
26.52           C
ATOM    490  CG2 THR    37      12.475   6.824  30.503  1.00
75.69           C
ATOM    491  OG1 THR    37      13.505   5.243  29.001  1.00
81.95           O
ATOM    492  HN  THR    37      10.936   5.009  27.756  1.00
18.47           H
ATOM    493  HA  THR    37      11.574   7.835  28.187  1.00
38.80           H
ATOM    494  HB  THR    37      13.873   7.260  28.939  1.00
81.60           H
ATOM    495  HG1 THR    37      13.195   4.764  29.774
1.00188.80          H
ATOM    496 HG21 THR    37      11.953   7.769  30.522
1.00196.98          H
ATOM    497 HG22 THR    37      13.280   6.843  31.224
1.00196.23          H
ATOM    498 HG23 THR    37      11.789   6.028  30.751
1.00168.92          H
ATOM    499  N   GLU    38      12.466   7.797  25.896  1.00
26.53           N
ATOM    500  CA  GLU    38      12.998   7.814  24.538  1.00
30.29           C
ATOM    501  C   GLU    38      14.517   7.951  24.551  1.00
25.39           C
ATOM    502  O   GLU    38      15.104   8.388  25.541  1.00
51.60           O
ATOM    503  CB  GLU    38      12.386   8.980  23.755  1.00
54.82           C
ATOM    504  CG  GLU    38      10.895   8.720  23.536
1.00165.84          C
ATOM    505  CD  GLU    38      10.255   9.917  22.840
1.00292.92          C
ATOM    506  OE1 GLU    38       9.108   9.802  22.443
1.00414.19          O
ATOM    507  OE2 GLU    38      10.922  10.931  22.714
1.00537.65          O1-
ATOM    508  HN  GLU    38      12.025   8.594  26.251  1.00
84.50           H
```

```
ATOM    509  HA  GLU    38      12.734   6.891  24.047  1.00
32.15          H
ATOM    510  HB1 GLU    38      12.879   9.068  22.800
1.00144.82          H
ATOM    511  HB2 GLU    38      12.516   9.895  24.314
1.00106.49          H
ATOM    512  HG1 GLU    38      10.414   8.563  24.490
1.00303.31          H
ATOM    513  HG2 GLU    38      10.771   7.841  22.923
1.00323.99          H
ATOM    514  N   CYS    39      15.147   7.577  23.441  1.00
15.84          N
ATOM    515  CA  CYS    39      16.598   7.665  23.326  1.00
14.30          C
ATOM    516  C   CYS    39      17.051   9.119  23.504  1.00
22.31          C
ATOM    517  O   CYS    39      16.304  10.043  23.183  1.00
32.92          O
ATOM    518  CB  CYS    39      17.034   7.147  21.952  1.00
18.08          C
ATOM    519  SG  CYS    39      15.915   7.797  20.685  1.00
17.41          S
ATOM    520  HN  CYS    39      14.625   7.244  22.684  1.00
24.87          H
ATOM    521  HA  CYS    39      17.044   7.047  24.088  1.00
14.60          H
ATOM    522  HB1 CYS    39      16.995   6.067  21.946  1.00
27.71          H
ATOM    523  HB2 CYS    39      18.041   7.472  21.744  1.00
34.91          H
ATOM    524  N   PRO    40      18.244   9.348  24.007  1.00
27.27          N
ATOM    525  CA  PRO    40      18.765  10.730  24.222  1.00
47.66          C
ATOM    526  C   PRO    40      19.185  11.391  22.911  1.00
71.54          C
ATOM    527  O   PRO    40      19.870  10.787  22.087  1.00
87.70          O
ATOM    528  CB  PRO    40      19.967  10.513  25.147  1.00
53.85          C
ATOM    529  CG  PRO    40      20.470   9.154  24.788  1.00
46.04          C
ATOM    530  CD  PRO    40      19.230   8.333  24.422  1.00
25.70          C
ATOM    531  HA  PRO    40      18.025  11.333  24.723  1.00
58.04          H
ATOM    532  HB1 PRO    40      19.654  10.530  26.181  1.00
67.93          H
ATOM    533  HB2 PRO    40      20.728  11.264  24.969  1.00
70.99          H
ATOM    534  HG1 PRO    40      20.973   8.702  25.630  1.00
65.91          H
```

```
ATOM    535  HG2 PRO    40       21.143   9.219  23.941  1.00
54.99           H
ATOM    536  HD1 PRO    40       18.868   7.789  25.281  1.00
24.22           H
ATOM    537  HD2 PRO    40       19.448   7.661  23.605  1.00
26.94           H
ATOM    538  N   GLY    41       18.775  12.640  22.736
1.00105.24           N
ATOM    539  CA  GLY    41       19.117  13.387  21.532
1.00147.55           C
ATOM    540  C   GLY    41       20.622  13.594  21.432
1.00161.74           C
ATOM    541  O   GLY    41       21.204  13.499  20.351
1.00244.37           O
ATOM    542  HN  GLY    41       18.237  13.067  23.433
1.00121.79           H
ATOM    543  HA1 GLY    41       18.630  14.350  21.560
1.00184.40           H
ATOM    544  HA2 GLY    41       18.774  12.840  20.668
1.00157.34           H
ATOM    545  N   LEU    42       21.246  13.878  22.573
1.00162.48           N
ATOM    546  CA  LEU    42       22.694  14.105  22.632
1.00191.48           C
ATOM    547  C   LEU    42       23.361  13.039  23.494
1.00166.66           C
ATOM    548  O   LEU    42       22.777  12.555  24.464
1.00251.38           O
ATOM    549  CB  LEU    42       22.976  15.486  23.229
1.00307.99           C
ATOM    550  CG  LEU    42       22.252  16.568  22.419
1.00452.28           C
ATOM    551  CD1 LEU    42       22.498  17.932  23.071
1.00681.24           C
ATOM    552  CD2 LEU    42       22.777  16.585  20.973
1.00527.46           C
ATOM    553  HN  LEU    42       20.719  13.938  23.396
1.00195.47           H
ATOM    554  HA  LEU    42       23.113  14.060  21.637
1.00229.77           H
ATOM    555  HB1 LEU    42       24.041  15.674  23.204
1.00342.49           H
ATOM    556  HB2 LEU    42       22.633  15.512  24.253
1.00335.33           H
ATOM    557  HG  LEU    42       21.191  16.359  22.416
1.00427.79           H
ATOM    558 HD11 LEU    42       22.276  17.872  24.126
1.00909.71           H
ATOM    559 HD12 LEU    42       21.859  18.671  22.611
1.00706.80           H
ATOM    560 HD13 LEU    42       23.532  18.214  22.937
1.00858.82           H
```

ATOM 561 HD21 LEU 42 22.270 15.825 20.397
1.00631.69 H
ATOM 562 HD22 LEU 42 23.840 16.391 20.967
1.00628.06 H
ATOM 563 HD23 LEU 42 22.588 17.552 20.528
1.00656.75 H
ATOM 564 N THR 43 24.589 12.679 23.135
1.00178.52 N
ATOM 565 CA THR 43 25.327 11.670 23.886
1.00240.84 C
ATOM 566 C THR 43 25.695 12.207 25.275
1.00372.31 C
ATOM 567 O THR 43 25.899 13.411 25.436
1.00500.13 O
ATOM 568 CB THR 43 26.599 11.296 23.117
1.00354.13 C
ATOM 569 CG2 THR 43 26.221 10.735 21.745
1.00463.04 C
ATOM 570 OG1 THR 43 27.405 12.454 22.949
1.00508.50 O
ATOM 571 HN THR 43 25.006 13.099 22.355
1.00229.59 H
ATOM 572 HA THR 43 24.708 10.794 23.987
1.00249.90 H
ATOM 573 HB THR 43 27.152 10.552 23.666
1.00481.58 H
ATOM 574 HG1 THR 43 27.050 12.959 22.213
1.00624.31 H
ATOM 575 HG21 THR 43 25.805 9.746 21.864
1.00650.95 H
ATOM 576 HG22 THR 43 27.102 10.682 21.122
1.00580.73 H
ATOM 577 HG23 THR 43 25.490 11.380 21.282
1.00571.99 H
ATOM 578 N PRO 44 25.787 11.359 26.276
1.00488.39 N
ATOM 579 CA PRO 44 26.138 11.794 27.663
1.00760.13 C
ATOM 580 C PRO 44 27.613 12.181 27.789
1.00735.36 C
ATOM 581 O PRO 44 28.024 12.775 28.786
1.00999.99 O
ATOM 582 CB PRO 44 25.806 10.564 28.518
1.00999.99 C
ATOM 583 CG PRO 44 25.993 9.405 27.597
1.00856.94 C
ATOM 584 CD PRO 44 25.569 9.897 26.211
1.00548.09 C
ATOM 585 HA PRO 44 25.513 12.620 27.961
1.00939.00 H
ATOM 586 HB1 PRO 44 24.779 10.606 28.855
1.00999.99 H

```
ATOM    587  HB2 PRO    44       26.477  10.493  29.365
1.00999.99           H
ATOM    588  HG1 PRO    44       25.369   8.577  27.899
1.00999.99           H
ATOM    589  HG2 PRO    44       27.034   9.103  27.587
1.00853.53           H
ATOM    590  HD1 PRO    44       24.525   9.684  26.034
1.00602.45           H
ATOM    591  HD2 PRO    44       26.186   9.449  25.445
1.00463.32           H
ATOM    592  N   ILE    45       28.402  11.836  26.776
1.00518.33           N
ATOM    593  CA  ILE    45       29.829  12.146  26.788
1.00582.79           C
ATOM    594  C   ILE    45       30.077  13.556  27.321
1.00795.93           C
ATOM    595  O   ILE    45       29.337  14.488  27.004
1.00898.75           O
ATOM    596  CB  ILE    45       30.399  12.027  25.372
1.00474.41           C
ATOM    597  CG1 ILE    45       31.926  12.135  25.424
1.00516.90           C
ATOM    598  CG2 ILE    45       29.839  13.148  24.492
1.00760.75           C
ATOM    599  CD1 ILE    45       32.513  11.751  24.064
1.00566.81           C
ATOM    600  HN  ILE    45       28.020  11.362  26.009
1.00395.72           H
ATOM    601  HA  ILE    45       30.333  11.437  27.427
1.00711.06           H
ATOM    602  HB  ILE    45       30.120  11.070  24.953
1.00477.81           H
ATOM    603 HG11 ILE    45       32.311  11.466  26.179
1.00624.28           H
ATOM    604 HG12 ILE    45       32.207  13.150  25.666
1.00744.84           H
ATOM    605 HG21 ILE    45       30.001  12.906  23.453
1.00920.57           H
ATOM    606 HG22 ILE    45       30.339  14.077  24.728
1.00999.99           H
ATOM    607 HG23 ILE    45       28.780  13.256  24.677
1.00865.46           H
ATOM    608 HD11 ILE    45       33.592  11.749  24.126
1.00658.89           H
ATOM    609 HD12 ILE    45       32.198  12.469  23.322
1.00734.30           H
ATOM    610 HD13 ILE    45       32.166  10.767  23.786
1.00675.84           H
ATOM    611  N   ALA    46       31.121  13.703  28.129
1.00999.99           N
ATOM    612  CA  ALA    46       31.457  15.002  28.699
1.00999.99           C
```

```
ATOM    613  C   ALA    46       31.765  16.009  27.596
1.00999.99           C
ATOM    614  CB  ALA    46       32.667  14.869  29.626
1.00999.99           C
ATOM    615  OT1 ALA    46       32.931  16.173  27.279
1.00999.99           O
ATOM    616  OT2 ALA    46       30.829  16.601  27.084
1.00999.99           O
ATOM    617  HN  ALA    46       31.675  12.925  28.345
1.00999.99           H
ATOM    618  HA  ALA    46       30.616  15.360  29.275
1.00999.99           H
ATOM    619  HB1 ALA    46       33.502  14.467  29.071
1.00999.99           H
ATOM    620  HB2 ALA    46       32.425  14.206  30.443
1.00999.99           H
ATOM    621  HB3 ALA    46       32.930  15.842  30.016
1.00999.99           H
ENDMDL
TER
MODEL       12
ATOM      1  N   GLY     1       26.359  -2.764  18.853
1.00999.99           N
ATOM      2  CA  GLY     1       26.472  -2.687  17.369
1.00999.99           C
ATOM      3  C   GLY     1       25.533  -1.607  16.842
1.00999.99           C
ATOM      4  O   GLY     1       25.083  -0.742  17.592
1.00999.99           O
ATOM      5  HA1 GLY     1       26.199  -3.638  16.937
1.00999.99           H
ATOM      6  HA2 GLY     1       27.490  -2.445  17.098
1.00999.99           H
ATOM      7  HT1 GLY     1       27.308  -2.729  19.276
1.00999.99           H
ATOM      8  HT2 GLY     1       25.893  -3.656  19.119
1.00999.99           H
ATOM      9  HT3 GLY     1       25.798  -1.961  19.200
1.00999.99           H
ATOM     10  N   LEU     2       25.244  -1.665  15.545
1.00999.99           N
ATOM     11  CA  LEU     2       24.355  -0.686  14.928
1.00895.59           C
ATOM     12  C   LEU     2       22.905  -0.968  15.307
1.00532.27           C
ATOM     13  O   LEU     2       22.506  -2.123  15.452
1.00625.21           O
ATOM     14  CB  LEU     2       24.502  -0.736  13.405
1.00999.99           C
ATOM     15  CG  LEU     2       25.959  -0.469  13.006
1.00999.99           C
ATOM     16  CD1 LEU     2       26.098  -0.611  11.487
```

```
1.00999.99           C
ATOM     17  CD2 LEU     2       26.374   0.951  13.433
1.00999.99           C
ATOM     18  HN  LEU     2       25.632  -2.377  14.997
1.00999.99           H
ATOM     19  HA  LEU     2       24.621   0.299  15.274
1.00890.87           H
ATOM     20  HB1 LEU     2       23.868   0.014  12.958
1.00924.07           H
ATOM     21  HB2 LEU     2       24.208  -1.713  13.050
1.00999.99           H
ATOM     22  HG  LEU     2       26.599  -1.193  13.490
1.00999.99           H
ATOM     23 HD11 LEU     2       25.902  -1.633  11.202
1.00999.99           H
ATOM     24 HD12 LEU     2       27.098  -0.338  11.189
1.00999.99           H
ATOM     25 HD13 LEU     2       25.387   0.040  10.998
1.00999.99           H
ATOM     26 HD21 LEU     2       27.194   1.294  12.815
1.00999.99           H
ATOM     27 HD22 LEU     2       26.693   0.938  14.464
1.00999.99           H
ATOM     28 HD23 LEU     2       25.536   1.626  13.324
1.00999.99           H
ATOM     29  N   CYS     3       22.122   0.093  15.466
1.00271.28           N
ATOM     30  CA  CYS     3       20.717  -0.056  15.828
1.00104.50           C
ATOM     31  C   CYS     3       19.961  -0.816  14.744  1.00
90.98           C
ATOM     32  O   CYS     3       19.114  -1.658  15.036
1.00207.28           O
ATOM     33  CB  CYS     3       20.071   1.316  16.027  1.00
37.83           C
ATOM     34  SG  CYS     3       20.703   2.077  17.543  1.00
81.56           S
ATOM     35  HN  CYS     3       22.492   0.990  15.339
1.00296.55           H
ATOM     36  HA  CYS     3       20.652  -0.607  16.749
1.00166.45           H
ATOM     37  HB1 CYS     3       19.001   1.202  16.101  1.00
40.38           H
ATOM     38  HB2 CYS     3       20.306   1.949  15.184  1.00
98.45           H
ATOM     39  N   SER     4       20.269  -0.505  13.491  1.00
85.31           N
ATOM     40  CA  SER     4       19.607  -1.154  12.362  1.00
92.94           C
ATOM     41  C   SER     4       18.198  -0.595  12.190  1.00
67.37           C
ATOM     42  O   SER     4       17.695  -0.475  11.073
```

```
1.00102.28          O
ATOM     43  CB  SER     4      19.538  -2.670  12.591
1.00123.83          C
ATOM     44  OG  SER     4      19.567  -3.333  11.334
1.00178.89          O
ATOM     45  HN  SER     4      20.950   0.179  13.322
1.00163.03          H
ATOM     46  HA  SER     4      20.174  -0.960  11.465
1.00129.78          H
ATOM     47  HB1 SER     4      18.623  -2.923  13.121
1.00105.26          H
ATOM     48  HB2 SER     4      20.385  -2.986  13.176
1.00156.87          H
ATOM     49  HG  SER     4      18.813  -3.032  10.821
1.00218.16          H
ATOM     50  N   GLU     5      17.567  -0.267  13.313  1.00
41.67           N
ATOM     51  CA  GLU     5      16.213   0.268  13.299  1.00
41.21           C
ATOM     52  C   GLU     5      15.971   1.132  14.533  1.00
41.92           C
ATOM     53  O   GLU     5      16.702   1.038  15.519  1.00
74.72           O
ATOM     54  CB  GLU     5      15.211  -0.884  13.265  1.00
49.24           C
ATOM     55  CG  GLU     5      15.427  -1.762  14.488  1.00
55.48           C
ATOM     56  CD  GLU     5      14.628  -3.054  14.355  1.00
92.06           C
ATOM     57  OE1 GLU     5      13.955  -3.212  13.350
1.00206.49          O
ATOM     58  OE2 GLU     5      14.700  -3.868  15.263
1.00197.24          O1-
ATOM     59  HN  GLU     5      18.022  -0.395  14.171  1.00
45.52           H
ATOM     60  HA  GLU     5      16.080   0.867  12.421  1.00
60.63           H
ATOM     61  HB1 GLU     5      15.362  -1.469  12.371  1.00
61.59           H
ATOM     62  HB2 GLU     5      14.209  -0.490  13.274  1.00
63.66           H
ATOM     63  HG1 GLU     5      15.108  -1.232  15.372  1.00
58.03           H
ATOM     64  HG2 GLU     5      16.477  -1.992  14.569  1.00
57.77           H
ATOM     65  N   ASN     6      14.950   1.980  14.469  1.00
50.73           N
ATOM     66  CA  ASN     6      14.633   2.862  15.589  1.00
71.04           C
ATOM     67  C   ASN     6      14.284   2.053  16.834  1.00
65.17           C
ATOM     68  O   ASN     6      14.675   2.408  17.946  1.00
```

```
94.86           O
ATOM     69  CB  ASN     6       13.457   3.767  15.220  1.00
94.83           C
ATOM     70  CG  ASN     6       13.899   4.805  14.195
1.00160.94           C
ATOM     71  ND2 ASN     6       13.004   5.402  13.457
1.00244.88           N
ATOM     72  OD1 ASN     6       15.092   5.080  14.065
1.00219.73           O
ATOM     73  HN  ASN     6       14.405   2.017  13.657  1.00
72.81           H
ATOM     74  HA  ASN     6       15.492   3.479  15.803  1.00
96.02           H
ATOM     75  HB1 ASN     6       13.099   4.269  16.107
1.00111.09           H
ATOM     76  HB2 ASN     6       12.660   3.167  14.803  1.00
98.13           H
ATOM     77 HD21 ASN     6       12.056   5.181  13.562
1.00272.43           H
ATOM     78 HD22 ASN     6       13.281   6.071  12.795
1.00336.78           H
ATOM     79  N   GLY     7       13.540   0.965  16.642  1.00
51.80           N
ATOM     80  CA  GLY     7       13.136   0.105  17.757  1.00
71.47           C
ATOM     81  C   GLY     7       14.016  -1.134  17.839  1.00
41.04           C
ATOM     82  O   GLY     7       13.522  -2.252  17.976  1.00
44.54           O
ATOM     83  HN  GLY     7       13.257   0.734  15.733  1.00
45.49           H
ATOM     84  HA1 GLY     7       12.116  -0.204  17.608
1.00103.52           H
ATOM     85  HA2 GLY     7       13.207   0.654  18.686
1.00108.67           H
ATOM     86  N   ASP     8       15.322  -0.929  17.748  1.00
27.00           N
ATOM     87  CA  ASP     8       16.264  -2.040  17.806  1.00
14.15           C
ATOM     88  C   ASP     8       16.148  -2.771  19.137  1.00
9.37           C
ATOM     89  O   ASP     8       16.020  -3.995  19.177  1.00
14.61           O
ATOM     90  CB  ASP     8       17.693  -1.531  17.630  1.00
15.06           C
ATOM     91  CG  ASP     8       18.642  -2.708  17.430  1.00
21.22           C
ATOM     92  OD1 ASP     8       19.811  -2.563  17.749
1.00119.56           O
ATOM     93  OD2 ASP     8       18.185  -3.738  16.960
1.00133.27           O1-
ATOM     94  HN  ASP     8       15.657  -0.016  17.634  1.00
```

```
35.63           H
ATOM     95  HA  ASP     8       16.039  -2.729  17.012  1.00
20.92           H
ATOM     96  HB1 ASP     8       17.989  -0.980  18.509  1.00
29.39           H
ATOM     97  HB2 ASP     8       17.736  -0.881  16.768  1.00
44.14           H
ATOM     98  N   CYS     9       16.189  -2.012  20.222  1.00
5.93           N
ATOM     99  CA  CYS     9       16.082  -2.599  21.551  1.00
7.54           C
ATOM    100  C   CYS     9       14.704  -3.226  21.731  1.00
14.60           C
ATOM    101  O   CYS     9       14.569  -4.326  22.269  1.00
25.78           O
ATOM    102  CB  CYS     9       16.314  -1.532  22.612  1.00
6.63           C
ATOM    103  SG  CYS     9       18.046  -1.045  22.558  1.00
10.91           S
ATOM    104  HN  CYS     9       16.289  -1.044  20.125  1.00
6.51           H
ATOM    105  HA  CYS     9       16.840  -3.356  21.658  1.00
11.12           H
ATOM    106  HB1 CYS     9       16.090  -1.934  23.586  1.00
11.28           H
ATOM    107  HB2 CYS     9       15.690  -0.674  22.416  1.00
5.04           H
ATOM    108  N   ALA    10       13.687  -2.512  21.255  1.00
16.26           N
ATOM    109  CA  ALA    10       12.304  -2.980  21.334  1.00
32.04           C
ATOM    110  C   ALA    10       11.382  -2.011  20.598  1.00
45.47           C
ATOM    111  O   ALA    10       11.784  -0.900  20.254
1.00119.04           O
ATOM    112  CB  ALA    10       11.856  -3.109  22.791  1.00
30.10           C
ATOM    113  HN  ALA    10       13.873  -1.651  20.829  1.00
12.81           H
ATOM    114  HA  ALA    10       12.238  -3.950  20.863  1.00
47.14           H
ATOM    115  HB1 ALA    10       11.693  -2.126  23.206  1.00
84.37           H
ATOM    116  HB2 ALA    10       12.616  -3.620  23.361
1.00118.20           H
ATOM    117  HB3 ALA    10       10.936  -3.673  22.835
1.00117.32           H
ATOM    118  N   ALA    11       10.148  -2.440  20.356  1.00
30.62           N
ATOM    119  CA  ALA    11        9.181  -1.600  19.654  1.00
35.24           C
ATOM    120  C   ALA    11        8.898  -0.315  20.432  1.00
```

```
22.78           C
ATOM    121  O   ALA    11        8.753   0.756  19.844  1.00
53.33           O
ATOM    122  CB  ALA    11        7.876  -2.371  19.454  1.00
60.49           C
ATOM    123  HN  ALA    11        9.884  -3.336  20.650  1.00
50.37           H
ATOM    124  HA  ALA    11        9.583  -1.341  18.687  1.00
41.70           H
ATOM    125  HB1 ALA    11        7.280  -1.879  18.700
1.00157.06           H
ATOM    126  HB2 ALA    11        7.328  -2.402  20.385
1.00148.83           H
ATOM    127  HB3 ALA    11        8.099  -3.379  19.134
1.00137.06           H
ATOM    128  N   ASP    12        8.804  -0.433  21.755  1.00
18.98           N
ATOM    129  CA  ASP    12        8.520   0.723  22.610  1.00
31.05           C
ATOM    130  C   ASP    12        9.807   1.380  23.106  1.00
22.98           C
ATOM    131  O   ASP    12        9.779   2.197  24.026  1.00
37.14           O
ATOM    132  CB  ASP    12        7.676   0.282  23.807  1.00
48.35           C
ATOM    133  CG  ASP    12        8.433  -0.753  24.630
1.00104.83           C
ATOM    134  OD1 ASP    12        9.468  -1.205  24.169
1.00258.73           O
ATOM    135  OD2 ASP    12        7.969  -1.081  25.710
1.00213.46           O1-
ATOM    136  HN  ASP    12        8.918  -1.315  22.166  1.00
36.19           H
ATOM    137  HA  ASP    12        7.955   1.451  22.043  1.00
49.76           H
ATOM    138  HB1 ASP    12        6.751  -0.150  23.455  1.00
95.71           H
ATOM    139  HB2 ASP    12        7.456   1.140  24.426  1.00
54.37           H
ATOM    140  N   GLU    13       10.935   1.020  22.494  1.00
17.60           N
ATOM    141  CA  GLU    13       12.238   1.578  22.879  1.00
12.01           C
ATOM    142  C   GLU    13       12.903   2.253  21.679  1.00
10.07           C
ATOM    143  O   GLU    13       12.565   1.965  20.533  1.00
12.81           O
ATOM    144  CB  GLU    13       13.130   0.458  23.415  1.00
11.74           C
ATOM    145  CG  GLU    13       12.575  -0.021  24.761  1.00
13.49           C
ATOM    146  CD  GLU    13       13.220  -1.342  25.160
```

```
1.00142.36           C
ATOM    147  OE1 GLU    13       13.024  -1.758  26.290
1.00339.06           O
ATOM    148  OE2 GLU    13       13.897  -1.920  24.330
1.00335.28           O1-
ATOM    149  HN  GLU    13       10.894   0.362  21.767  1.00
28.36          H
ATOM    150  HA  GLU    13       12.102   2.314  23.659  1.00
13.06          H
ATOM    151  HB1 GLU    13       14.134   0.830  23.554  1.00
13.76          H
ATOM    152  HB2 GLU    13       13.142  -0.363  22.712  1.00
9.94          H
ATOM    153  HG1 GLU    13       11.506  -0.157  24.679  1.00
66.02          H
ATOM    154  HG2 GLU    13       12.783   0.722  25.516  1.00
56.23          H
ATOM    155  N   CYS    14       13.843   3.162  21.952  1.00
9.12          N
ATOM    156  CA  CYS    14       14.546   3.889  20.889  1.00
10.69          C
ATOM    157  C   CYS    14       16.012   3.483  20.839  1.00
9.56          C
ATOM    158  O   CYS    14       16.622   3.226  21.873  1.00
13.62          O
ATOM    159  CB  CYS    14       14.454   5.395  21.140  1.00
13.96          C
ATOM    160  SG  CYS    14       15.363   6.278  19.843  1.00
42.96          S
ATOM    161  HN  CYS    14       14.068   3.355  22.888  1.00
9.76          H
ATOM    162  HA  CYS    14       14.090   3.670  19.933  1.00
15.54          H
ATOM    163  HB1 CYS    14       14.885   5.624  22.102  1.00
50.72          H
ATOM    164  HB2 CYS    14       13.417   5.701  21.126  1.00
46.13          H
ATOM    165  N   CYS    15       16.571   3.429  19.633  1.00
9.60          N
ATOM    166  CA  CYS    15       17.974   3.059  19.449  1.00
9.28          C
ATOM    167  C   CYS    15       18.600   3.952  18.388  1.00
9.92          C
ATOM    168  O   CYS    15       18.052   4.109  17.298  1.00
13.31          O
ATOM    169  CB  CYS    15       18.074   1.594  19.011  1.00
12.90          C
ATOM    170  SG  CYS    15       19.806   1.054  19.040  1.00
39.11          S
ATOM    171  HN  CYS    15       16.033   3.654  18.844  1.00
13.12          H
ATOM    172  HA  CYS    15       18.511   3.186  20.379  1.00
```

```
8.26           H
ATOM    173  HB1 CYS    15      17.681   1.492  18.010  1.00
24.82          H
ATOM    174  HB2 CYS    15      17.496   0.980  19.686  1.00
27.31          H
ATOM    175  N   VAL    16      19.745   4.547  18.713  1.00
9.56           N
ATOM    176  CA  VAL    16      20.425   5.435  17.774  1.00
11.99          C
ATOM    177  C   VAL    16      21.938   5.302  17.912  1.00
7.73           C
ATOM    178  O   VAL    16      22.474   5.313  19.021  1.00
8.83           O
ATOM    179  CB  VAL    16      19.997   6.880  18.044  1.00
18.37          C
ATOM    180  CG1 VAL    16      20.588   7.358  19.373  1.00
39.61          C
ATOM    181  CG2 VAL    16      20.490   7.774  16.908
1.00115.21          C
ATOM    182  HN  VAL    16      20.134   4.392  19.599  1.00
9.76           H
ATOM    183  HA  VAL    16      20.146   5.173  16.763  1.00
17.27          H
ATOM    184  HB  VAL    16      18.920   6.927  18.096  1.00
52.63          H
ATOM    185 HG11 VAL    16      20.103   8.276  19.671
1.00128.07          H
ATOM    186 HG12 VAL    16      21.647   7.532  19.254
1.00154.13          H
ATOM    187 HG13 VAL    16      20.429   6.604  20.130
1.00135.74          H
ATOM    188 HG21 VAL    16      21.550   7.622  16.763
1.00229.05          H
ATOM    189 HG22 VAL    16      20.303   8.806  17.156
1.00261.82          H
ATOM    190 HG23 VAL    16      19.964   7.520  16.000
1.00210.07          H
ATOM    191  N   ASP    17      22.623   5.168  16.776  1.00
14.34          N
ATOM    192  CA  ASP    17      24.079   5.026  16.776  1.00
11.90          C
ATOM    193  C   ASP    17      24.759   6.383  16.633  1.00
13.15          C
ATOM    194  O   ASP    17      24.529   7.106  15.663  1.00
25.96          O
ATOM    195  CB  ASP    17      24.512   4.126  15.617  1.00
20.80          C
ATOM    196  CG  ASP    17      23.858   2.757  15.744  1.00
28.72          C
ATOM    197  OD1 ASP    17      23.816   2.240  16.849
1.00135.42          O
ATOM    198  OD2 ASP    17      23.410   2.243  14.733
```

```
1.00114.17           O1-
ATOM    199  HN  ASP    17       22.139   5.160  15.925  1.00
28.29           H
ATOM    200  HA  ASP    17       24.393   4.570  17.703  1.00
10.49           H
ATOM    201  HB1 ASP    17       25.587   4.012  15.637  1.00
22.42           H
ATOM    202  HB2 ASP    17       24.216   4.579  14.683  1.00
29.52           H
ATOM    203  N   THR    18       25.618   6.710  17.596  1.00
11.55           N
ATOM    204  CA  THR    18       26.360   7.973  17.573  1.00
17.10           C
ATOM    205  C   THR    18       27.781   7.724  17.084  1.00
10.63           C
ATOM    206  O   THR    18       28.287   6.606  17.169  1.00
6.49           O
ATOM    207  CB  THR    18       26.398   8.588  18.989  1.00
28.52           C
ATOM    208  CG2 THR    18       25.195   9.513  19.205  1.00
45.38           C
ATOM    209  OG1 THR    18       26.370   7.546  19.950  1.00
26.49           O
ATOM    210  HN  THR    18       25.768   6.084  18.335  1.00
13.96           H
ATOM    211  HA  THR    18       25.876   8.666  16.896  1.00
26.43           H
ATOM    212  HB  THR    18       27.305   9.163  19.116  1.00
37.43           H
ATOM    213  HG1 THR    18       25.549   7.060  19.838  1.00
71.62           H
ATOM    214 HG21 THR    18       24.317   9.080  18.751
1.00100.44           H
ATOM    215 HG22 THR    18       25.399  10.474  18.755
1.00129.42           H
ATOM    216 HG23 THR    18       25.029   9.643  20.263
1.00156.21           H
ATOM    217  N   VAL    19       28.420   8.780  16.596  1.00
16.32           N
ATOM    218  CA  VAL    19       29.780   8.699  16.117  1.00
13.34           C
ATOM    219  C   VAL    19       30.222  10.058  15.610  1.00
25.88           C
ATOM    220  O   VAL    19       29.673  10.608  14.654  1.00
42.11           O
ATOM    221  CB  VAL    19       29.940   7.658  15.009  1.00
15.48           C
ATOM    222  CG1 VAL    19       28.925   7.917  13.892  1.00
29.06           C
ATOM    223  CG2 VAL    19       31.367   7.756  14.447  1.00
20.67           C
ATOM    224  HN  VAL    19       27.977   9.648  16.580  1.00
```

```
26.89           H
ATOM    225  HA  VAL    19       30.418   8.418  16.945  1.00
8.08           H
ATOM    226  HB  VAL    19       29.784   6.672  15.419  1.00
11.49           H
ATOM    227 HG11 VAL    19       29.257   8.747  13.284
1.00137.43           H
ATOM    228 HG12 VAL    19       27.962   8.150  14.323  1.00
93.29           H
ATOM    229 HG13 VAL    19       28.837   7.034  13.275
1.00108.69           H
ATOM    230 HG21 VAL    19       31.445   8.631  13.817  1.00
99.42           H
ATOM    231 HG22 VAL    19       31.591   6.876  13.867
1.00111.36           H
ATOM    232 HG23 VAL    19       32.071   7.843  15.265  1.00
77.11           H
ATOM    233  N   PHE    20       31.216  10.574  16.278  1.00
24.58           N
ATOM    234  CA  PHE    20       31.785  11.869  15.957  1.00
40.34           C
ATOM    235  C   PHE    20       32.628  11.801  14.680  1.00
40.37           C
ATOM    236  O   PHE    20       32.120  11.980  13.574  1.00
66.02           O
ATOM    237  CB  PHE    20       32.646  12.357  17.151  1.00
48.79           C
ATOM    238  CG  PHE    20       32.962  11.196  18.075  1.00
35.75           C
ATOM    239  CD1 PHE    20       31.964  10.683  18.919  1.00
30.20           C
ATOM    240  CD2 PHE    20       34.248  10.641  18.096  1.00
40.95           C
ATOM    241  CE1 PHE    20       32.255   9.621  19.777  1.00
30.41           C
ATOM    242  CE2 PHE    20       34.535   9.574  18.958  1.00
45.84           C
ATOM    243  CZ  PHE    20       33.539   9.067  19.799  1.00
40.81           C
ATOM    244  HN  PHE    20       31.574  10.066  17.026  1.00
16.73           H
ATOM    245  HA  PHE    20       30.978  12.571  15.796  1.00
59.91           H
ATOM    246  HB1 PHE    20       32.098  13.091  17.700  1.00
67.15           H
ATOM    247  HB2 PHE    20       33.567  12.801  16.805  1.00
58.17           H
ATOM    248  HD1 PHE    20       30.966  11.104  18.903  1.00
33.34           H
ATOM    249  HD2 PHE    20       35.014  11.028  17.445  1.00
49.35           H
ATOM    250  HE1 PHE    20       31.487   9.225  20.422  1.00
```

```
30.61           H
ATOM    251  HE2 PHE    20       35.526   9.146  18.974  1.00
61.27           H
ATOM    252  HZ  PHE    20       33.761   8.245  20.463  1.00
52.66           H
ATOM    253  N   GLU    21       33.922  11.566  14.857  1.00
41.31           N
ATOM    254  CA  GLU    21       34.857  11.501  13.740  1.00
56.16           C
ATOM    255  C   GLU    21       34.973  10.074  13.207  1.00
58.61           C
ATOM    256  O   GLU    21       34.595   9.794  12.070
1.00201.19           O
ATOM    257  CB  GLU    21       36.217  12.034  14.230  1.00
57.37           C
ATOM    258  CG  GLU    21       37.374  11.488  13.380
1.00193.56           C
ATOM    259  CD  GLU    21       38.659  12.244  13.700
1.00304.62           C
ATOM    260  OE1 GLU    21       39.491  11.689  14.399
1.00451.81           O
ATOM    261  OE2 GLU    21       38.790  13.366  13.243
1.00442.88           O1-
ATOM    262  HN  GLU    21       34.264  11.451  15.768  1.00
50.82           H
ATOM    263  HA  GLU    21       34.505  12.135  12.945  1.00
85.72           H
ATOM    264  HB1 GLU    21       36.353  11.741  15.263  1.00
46.37           H
ATOM    265  HB2 GLU    21       36.217  13.115  14.171
1.00131.81           H
ATOM    266  HG1 GLU    21       37.137  11.608  12.334
1.00314.91           H
ATOM    267  HG2 GLU    21       37.514  10.438  13.598
1.00266.92           H
ATOM    268  N   GLY    22       35.499   9.183  14.032  1.00
86.70           N
ATOM    269  CA  GLY    22       35.659   7.790  13.625
1.00104.66           C
ATOM    270  C   GLY    22       36.661   7.062  14.517  1.00
71.50           C
ATOM    271  O   GLY    22       36.778   5.837  14.458  1.00
89.87           O
ATOM    272  HN  GLY    22       35.784   9.465  14.923
1.00214.64           H
ATOM    273  HA1 GLY    22       36.010   7.756  12.605
1.00152.47           H
ATOM    274  HA2 GLY    22       34.704   7.291  13.689
1.00118.15           H
ATOM    275  N   ASP    23       37.376   7.814  15.348  1.00
44.68           N
ATOM    276  CA  ASP    23       38.352   7.208  16.246  1.00
```

43.33 C
ATOM 277 C ASP 23 37.652 6.214 17.164 1.00
37.55 C
ATOM 278 O ASP 23 38.150 5.117 17.413 1.00
61.37 O
ATOM 279 CB ASP 23 39.039 8.288 17.084 1.00
40.74 C
ATOM 280 CG ASP 23 40.123 7.662 17.954
1.00141.86 C
ATOM 281 OD1 ASP 23 40.859 8.409 18.578
1.00328.18 O
ATOM 282 OD2 ASP 23 40.201 6.445 17.985
1.00304.58 O1-
ATOM 283 HN ASP 23 37.241 8.784 15.362 1.00
40.86 H
ATOM 284 HA ASP 23 39.095 6.687 15.663 1.00
72.12 H
ATOM 285 HB1 ASP 23 38.309 8.772 17.716 1.00
94.15 H
ATOM 286 HB2 ASP 23 39.486 9.021 16.427 1.00
93.26 H
ATOM 287 N MET 24 36.482 6.614 17.647 1.00
23.24 N
ATOM 288 CA MET 24 35.679 5.770 18.526 1.00
32.50 C
ATOM 289 C MET 24 34.206 6.029 18.267 1.00
25.13 C
ATOM 290 O MET 24 33.795 7.165 18.037 1.00
54.40 O
ATOM 291 CB MET 24 36.014 6.058 19.992 1.00
47.36 C
ATOM 292 CG MET 24 35.186 5.141 20.893
1.00151.73 C
ATOM 293 SD MET 24 35.731 5.322 22.612
1.00209.23 S
ATOM 294 CE MET 24 35.068 6.980 22.906
1.00243.29 C
ATOM 295 HN MET 24 36.143 7.498 17.394 1.00
17.32 H
ATOM 296 HA MET 24 35.882 4.730 18.314 1.00
48.94 H
ATOM 297 HB1 MET 24 35.784 7.088 20.218
1.00124.40 H
ATOM 298 HB2 MET 24 37.067 5.879 20.162
1.00166.96 H
ATOM 299 HG1 MET 24 35.319 4.115 20.581
1.00331.71 H
ATOM 300 HG2 MET 24 34.142 5.407 20.816
1.00302.30 H
ATOM 301 HE1 MET 24 35.730 7.716 22.471
1.00340.44 H
ATOM 302 HE2 MET 24 34.990 7.155 23.968

1.00373.88 H
ATOM 303 HE3 MET 24 34.087 7.062 22.460
1.00386.81 H
ATOM 304 N VAL 25 33.418 4.963 18.277 1.00
22.44 N
ATOM 305 CA VAL 25 31.992 5.067 18.017 1.00
15.37 C
ATOM 306 C VAL 25 31.194 5.005 19.314 1.00
17.47 C
ATOM 307 O VAL 25 31.679 4.521 20.336 1.00
29.52 O
ATOM 308 CB VAL 25 31.585 3.931 17.087 1.00
23.36 C
ATOM 309 CG1 VAL 25 32.058 4.246 15.665 1.00
58.39 C
ATOM 310 CG2 VAL 25 32.230 2.628 17.563 1.00
55.25 C
ATOM 311 HN VAL 25 33.805 4.081 18.444 1.00
48.11 H
ATOM 312 HA VAL 25 31.779 6.009 17.528 1.00
9.25 H
ATOM 313 HB VAL 25 30.521 3.829 17.097 1.00
52.45 H
ATOM 314 HG11 VAL 25 32.025 3.349 15.065
1.00171.07 H
ATOM 315 HG12 VAL 25 33.071 4.623 15.695
1.00166.22 H
ATOM 316 HG13 VAL 25 31.412 4.994 15.231
1.00134.76 H
ATOM 317 HG21 VAL 25 33.286 2.644 17.333
1.00141.88 H
ATOM 318 HG22 VAL 25 31.765 1.792 17.060
1.00184.50 H
ATOM 319 HG23 VAL 25 32.095 2.528 18.630
1.00133.90 H
ATOM 320 N THR 26 29.971 5.526 19.262 1.00
13.60 N
ATOM 321 CA THR 26 29.096 5.567 20.430 1.00
21.19 C
ATOM 322 C THR 26 27.670 5.166 20.054 1.00
16.55 C
ATOM 323 O THR 26 27.202 5.461 18.955 1.00
11.43 O
ATOM 324 CB THR 26 29.100 6.983 20.999 1.00
27.07 C
ATOM 325 CG2 THR 26 28.231 7.048 22.258 1.00
44.39 C
ATOM 326 OG1 THR 26 30.432 7.346 21.330 1.00
34.75 O
ATOM 327 HN THR 26 29.654 5.912 18.417 1.00
10.81 H
ATOM 328 HA THR 26 29.462 4.885 21.185 1.00

```
33.16           H
ATOM    329  HB  THR    26       28.712   7.668  20.257  1.00
20.60           H
ATOM    330  HG1 THR    26       30.411   8.213  21.741  1.00
77.72           H
ATOM    331 HG21 THR    26       28.285   8.043  22.677
1.00126.99           H
ATOM    332 HG22 THR    26       28.596   6.333  22.982
1.00108.32           H
ATOM    333 HG23 THR    26       27.209   6.817  22.008
1.00151.99           H
ATOM    334  N   ARG    27       26.985   4.494  20.980  1.00
22.38           N
ATOM    335  CA  ARG    27       25.605   4.053  20.751  1.00
20.21           C
ATOM    336  C   ARG    27       24.748   4.355  21.975  1.00
16.96           C
ATOM    337  O   ARG    27       25.268   4.565  23.072  1.00
20.06           O
ATOM    338  CB  ARG    27       25.580   2.549  20.465  1.00
22.14           C
ATOM    339  CG  ARG    27       26.051   1.783  21.704
1.00124.30           C
ATOM    340  CD  ARG    27       26.267   0.312  21.343
1.00109.38           C
ATOM    341  NE  ARG    27       25.011  -0.289  20.903
1.00227.73           N
ATOM    342  CZ  ARG    27       24.149  -0.804  21.776
1.00426.12           C
ATOM    343  NH1 ARG    27       23.035  -1.340  21.354
1.00767.09           N1+
ATOM    344  NH2 ARG    27       24.415  -0.774  23.053
1.00581.78           N
ATOM    345  HN  ARG    27       27.414   4.291  21.837  1.00
30.75           H
ATOM    346  HA  ARG    27       25.195   4.577  19.900  1.00
23.13           H
ATOM    347  HB1 ARG    27       26.237   2.328  19.637  1.00
87.46           H
ATOM    348  HB2 ARG    27       24.572   2.248  20.216
1.00103.36           H
ATOM    349  HG1 ARG    27       25.302   1.855  22.478
1.00281.97           H
ATOM    350  HG2 ARG    27       26.979   2.208  22.058
1.00276.19           H
ATOM    351  HD1 ARG    27       26.640  -0.217  22.208
1.00183.60           H
ATOM    352  HD2 ARG    27       26.990   0.245  20.544
1.00142.93           H
ATOM    353  HE  ARG    27       24.799  -0.315  19.947
1.00372.53           H
ATOM    354 HH11 ARG    27       22.831  -1.361  20.376
```

```
1.00910.59              H
ATOM    355 HH12 ARG    27       22.388  -1.728  22.008
1.00999.99              H
ATOM    356 HH21 ARG    27       25.266  -0.363  23.376
1.00532.54              H
ATOM    357 HH22 ARG    27       23.767  -1.163  23.708
1.00948.84              H
ATOM    358  N   SER    28       23.429   4.387  21.784  1.00
14.60           N
ATOM    359  CA  SER    28       22.508   4.678  22.882  1.00
14.92           C
ATOM    360  C   SER    28       21.183   3.947  22.687  1.00
12.12           C
ATOM    361  O   SER    28       20.764   3.687  21.560  1.00
13.41           O
ATOM    362  CB  SER    28       22.252   6.183  22.963  1.00
23.26           C
ATOM    363  OG  SER    28       21.363   6.450  24.039
1.00146.32              O
ATOM    364  HN  SER    28       23.068   4.220  20.887  1.00
15.23           H
ATOM    365  HA  SER    28       22.949   4.350  23.814  1.00
15.44           H
ATOM    366  HB1 SER    28       21.820   6.523  22.032  1.00
88.73           H
ATOM    367  HB2 SER    28       23.183   6.698  23.134
1.00124.68              H
ATOM    368  HG  SER    28       20.478   6.205  23.760
1.00242.47              H
ATOM    369  N   CYS    29       20.528   3.622  23.797  1.00
10.48           N
ATOM    370  CA  CYS    29       19.249   2.925  23.749  1.00
9.97           C
ATOM    371  C   CYS    29       18.627   2.894  25.144  1.00
11.60           C
ATOM    372  O   CYS    29       19.287   2.525  26.116  1.00
15.64           O
ATOM    373  CB  CYS    29       19.450   1.497  23.216  1.00
9.52           C
ATOM    374  SG  CYS    29       17.969   0.955  22.350  1.00
10.40           S
ATOM    375  HN  CYS    29       20.908   3.863  24.667  1.00
11.10           H
ATOM    376  HA  CYS    29       18.586   3.459  23.085  1.00
11.08           H
ATOM    377  HB1 CYS    29       19.643   0.814  24.033  1.00
9.33           H
ATOM    378  HB2 CYS    29       20.283   1.477  22.532  1.00
10.78           H
ATOM    379  N   GLU    30       17.358   3.290  25.241  1.00
13.52           N
ATOM    380  CA  GLU    30       16.660   3.311  26.531  1.00
```

17.41   C
ATOM   381 C   GLU   30   15.644   2.181 26.615 1.00
10.81   C
ATOM   382 O   GLU   30   14.932   1.898 25.651 1.00
23.22   O
ATOM   383 CB GLU   30   15.949   4.653 26.706 1.00
37.83   C
ATOM   384 CG GLU   30   16.986   5.777 26.724
1.00108.13   C
ATOM   385 CD GLU   30   17.795   5.720 28.015
1.00240.52   C
ATOM   386 OE1 GLU   30   18.825   6.373 28.076
1.00422.56   O
ATOM   387 OE2 GLU   30   17.376   5.024 28.924
1.00410.81   O1-
ATOM   388 HN GLU   30   16.881   3.579 24.434 1.00
15.61   H
ATOM   389 HA GLU   30   17.377   3.195 27.334 1.00
24.60   H
ATOM   390 HB1 GLU   30   15.404   4.654 27.638 1.00
72.67   H
ATOM   391 HB2 GLU   30   15.263   4.808 25.886 1.00
56.47   H
ATOM   392 HG1 GLU   30   16.485   6.731 26.659
1.00196.66   H
ATOM   393 HG2 GLU   30   17.652   5.663 25.880
1.00140.67   H
ATOM   394 N   LYS   31   15.584   1.535 27.779 1.00
11.96   N
ATOM   395 CA LYS   31   14.658   0.431 27.996 1.00
11.40   C
ATOM   396 C   LYS   31   13.379   0.923 28.665 1.00
10.99   C
ATOM   397 O   LYS   31   13.314   2.049 29.159 1.00
12.86   O
ATOM   398 CB LYS   31   15.318 -0.638 28.870 1.00
21.40   C
ATOM   399 CG LYS   31   16.074   0.022 30.028 1.00
57.81   C
ATOM   400 CD LYS   31   16.688 -1.060 30.917
1.00115.52   C
ATOM   401 CE LYS   31   17.447 -0.403 32.071
1.00250.23   C
ATOM   402 NZ LYS   31   18.037 -1.459 32.943
1.00462.10   N1+
ATOM   403 HN LYS   31   16.176   1.804 28.507 1.00
26.13   H
ATOM   404 HA LYS   31   14.404 -0.012 27.046 1.00
13.87   H
ATOM   405 HB1 LYS   31   16.008 -1.211 28.273 1.00
37.63   H
ATOM   406 HB2 LYS   31   14.563 -1.290 29.266 1.00

```
47.19           H
ATOM    407  HG1 LYS    31       15.391   0.621  30.611
1.00126.32           H
ATOM    408  HG2 LYS    31       16.861   0.649  29.635
1.00114.19           H
ATOM    409  HD1 LYS    31       17.372  -1.660  30.335
1.00198.45           H
ATOM    410  HD2 LYS    31       15.904  -1.689  31.312
1.00200.14           H
ATOM    411  HE1 LYS    31       16.767   0.203  32.650
1.00374.64           H
ATOM    412  HE2 LYS    31       18.237   0.218  31.676
1.00403.27           H
ATOM    413  HZ1 LYS    31       18.483  -2.188  32.351
1.00627.38           H
ATOM    414  HZ2 LYS    31       17.285  -1.891  33.519
1.00622.26           H
ATOM    415  HZ3 LYS    31       18.752  -1.035  33.567
1.00619.20           H
ATOM    416  N   THR    32       12.368   0.060  28.677  1.00
15.51           N
ATOM    417  CA  THR    32       11.077   0.388  29.290  1.00
22.44           C
ATOM    418  C   THR    32       10.837  -0.483  30.520  1.00
26.60           C
ATOM    419  O   THR    32       10.867  -1.711  30.439  1.00
57.43           O
ATOM    420  CB  THR    32        9.953   0.155  28.277  1.00
56.28           C
ATOM    421  CG2 THR    32        8.606   0.490  28.921
1.00102.70           C
ATOM    422  OG1 THR    32       10.159   0.987  27.144
1.00111.17           O
ATOM    423  HN  THR    32       12.493  -0.823  28.268  1.00
18.79           H
ATOM    424  HA  THR    32       11.065   1.429  29.587  1.00
19.67           H
ATOM    425  HB  THR    32        9.953  -0.880  27.970  1.00
84.25           H
ATOM    426  HG1 THR    32        9.381   0.925  26.585
1.00205.03           H
ATOM    427 HG21 THR    32        7.847   0.552  28.155
1.00220.71           H
ATOM    428 HG22 THR    32        8.679   1.439  29.434
1.00174.67           H
ATOM    429 HG23 THR    32        8.344  -0.283  29.627
1.00217.44           H
ATOM    430  N   THR    33       10.594   0.160  31.659  1.00
22.47           N
ATOM    431  CA  THR    33       10.344  -0.563  32.905  1.00
41.60           C
ATOM    432  C   THR    33        9.460   0.239  33.817  1.00
```

```
38.03           C
ATOM    433  O   THR    33        9.927   0.948  34.710  1.00
52.73           O
ATOM    434  CB  THR    33       11.656  -0.890  33.615  1.00
63.25           C
ATOM    435  CG2 THR    33       12.428  -1.943  32.818
1.00121.65           C
ATOM    436  OG1 THR    33       12.440   0.290  33.728  1.00
96.20           O
ATOM    437  HN  THR    33       10.581   1.141  31.662  1.00
23.82           H
ATOM    438  HA  THR    33        9.823  -1.476  32.692  1.00
67.12           H
ATOM    439  HB  THR    33       11.441  -1.275  34.597
1.00107.59           H
ATOM    440  HG1 THR    33       13.037   0.180  34.472
1.00176.27           H
ATOM    441 HG21 THR    33       12.799  -1.505  31.905
1.00255.23           H
ATOM    442 HG22 THR    33       11.772  -2.769  32.581
1.00245.37           H
ATOM    443 HG23 THR    33       13.258  -2.303  33.408
1.00185.78           H
ATOM    444  N   GLY    34        8.168   0.101  33.594  1.00
40.93           N
ATOM    445  CA  GLY    34        7.195   0.790  34.403  1.00
45.59           C
ATOM    446  C   GLY    34        7.076   2.247  33.976  1.00
40.97           C
ATOM    447  O   GLY    34        6.216   2.982  34.458  1.00
82.17           O
ATOM    448  HN  GLY    34        7.867  -0.495  32.876  1.00
54.00           H
ATOM    449  HA1 GLY    34        7.518   0.739  35.423  1.00
54.41           H
ATOM    450  HA2 GLY    34        6.234   0.303  34.303  1.00
62.09           H
ATOM    451  N   ASN    35        7.949   2.648  33.056  1.00
32.51           N
ATOM    452  CA  ASN    35        7.952   4.014  32.542  1.00
33.20           C
ATOM    453  C   ASN    35        8.595   4.047  31.162  1.00
23.17           C
ATOM    454  O   ASN    35        9.473   3.239  30.860  1.00
27.01           O
ATOM    455  CB  ASN    35        8.726   4.933  33.487  1.00
54.51           C
ATOM    456  CG  ASN    35        8.099   4.911  34.876  1.00
80.73           C
ATOM    457  ND2 ASN    35        8.836   4.613  35.911
1.00220.46           N
ATOM    458  OD1 ASN    35        6.904   5.170  35.023
```

```
1.00117.80           O
ATOM    459  HN  ASN    35       8.604   2.008  32.709  1.00
51.15          H
ATOM    460  HA  ASN    35       6.933   4.365  32.466  1.00
47.21          H
ATOM    461  HB1 ASN    35       8.703   5.942  33.101  1.00
68.31          H
ATOM    462  HB2 ASN    35       9.751   4.599  33.550  1.00
57.75          H
ATOM    463 HD21 ASN    35       9.786   4.405  35.791
1.00405.39           H
ATOM    464 HD22 ASN    35       8.439   4.595  36.807
1.00245.64           H
ATOM    465  N   PHE    36       8.150   4.978  30.324  1.00
35.72          N
ATOM    466  CA  PHE    36       8.680   5.107  28.976  1.00
33.41          C
ATOM    467  C   PHE    36       9.749   6.193  28.915  1.00
28.51          C
ATOM    468  O   PHE    36       9.591   7.270  29.492  1.00
45.23          O
ATOM    469  CB  PHE    36       7.538   5.456  28.034  1.00
67.53          C
ATOM    470  CG  PHE    36       6.572   4.299  27.976  1.00
86.71          C
ATOM    471  CD1 PHE    36       5.464   4.272  28.832
1.00106.51           C
ATOM    472  CD2 PHE    36       6.784   3.253  27.071  1.00
99.48          C
ATOM    473  CE1 PHE    36       4.567   3.199  28.779
1.00134.48           C
ATOM    474  CE2 PHE    36       5.888   2.179  27.020
1.00134.27           C
ATOM    475  CZ  PHE    36       4.779   2.153  27.874
1.00149.48           C
ATOM    476  HN  PHE    36       7.446   5.591  30.612  1.00
62.87          H
ATOM    477  HA  PHE    36       9.113   4.167  28.662  1.00
28.53          H
ATOM    478  HB1 PHE    36       7.928   5.650  27.056  1.00
73.56          H
ATOM    479  HB2 PHE    36       7.026   6.336  28.399  1.00
88.42          H
ATOM    480  HD1 PHE    36       5.302   5.080  29.531
1.00110.20           H
ATOM    481  HD2 PHE    36       7.642   3.273  26.415  1.00
93.07          H
ATOM    482  HE1 PHE    36       3.711   3.178  29.439
1.00155.06           H
ATOM    483  HE2 PHE    36       6.052   1.372  26.322
1.00159.46           H
ATOM    484  HZ  PHE    36       4.086   1.324  27.834
```

```
1.00183.25           H
ATOM    485  N   THR    37      10.839   5.903  28.206  1.00
20.06          N
ATOM    486  CA  THR    37      11.936   6.854  28.062  1.00
27.18          C
ATOM    487  C   THR    37      12.573   6.713  26.686  1.00
41.87          C
ATOM    488  O   THR    37      13.120   5.665  26.346
1.00169.86           O
ATOM    489  CB  THR    37      12.989   6.604  29.146  1.00
26.52          C
ATOM    490  CG2 THR    37      12.373   6.848  30.524  1.00
75.69          C
ATOM    491  OG1 THR    37      13.446   5.260  29.057  1.00
81.95          O
ATOM    492  HN  THR    37      10.904   5.032  27.765  1.00
18.47          H
ATOM    493  HA  THR    37      11.557   7.861  28.169  1.00
38.80          H
ATOM    494  HB  THR    37      13.820   7.278  29.003  1.00
81.60          H
ATOM    495  HG1 THR    37      12.832   4.775  28.502
1.00188.80           H
ATOM    496 HG21 THR    37      11.806   7.767  30.508
1.00196.98           H
ATOM    497 HG22 THR    37      13.160   6.923  31.261
1.00196.23           H
ATOM    498 HG23 THR    37      11.720   6.025  30.777
1.00168.92           H
ATOM    499  N   GLU    38      12.492   7.777  25.898  1.00
26.53          N
ATOM    500  CA  GLU    38      13.060   7.771  24.553  1.00
30.29          C
ATOM    501  C   GLU    38      14.571   7.971  24.600  1.00
25.39          C
ATOM    502  O   GLU    38      15.117   8.425  25.605  1.00
51.60          O
ATOM    503  CB  GLU    38      12.422   8.880  23.714  1.00
54.82          C
ATOM    504  CG  GLU    38      10.939   8.571  23.499
1.00165.84           C
ATOM    505  CD  GLU    38      10.283   9.692  22.700
1.00292.92           C
ATOM    506  OE1 GLU    38      10.944  10.690  22.466
1.00414.19           O
ATOM    507  OE2 GLU    38       9.130   9.535  22.335
1.00537.65           O1-
ATOM    508  HN  GLU    38      12.039   8.579  26.224  1.00
84.50          H
ATOM    509  HA  GLU    38      12.846   6.820  24.089  1.00
32.15          H
ATOM    510  HB1 GLU    38      12.917   8.938  22.757
```

1.00144.82 H
ATOM 511 HB2 GLU 38 12.522 9.824 24.231
1.00106.49 H
ATOM 512 HG1 GLU 38 10.449 8.482 24.457
1.00303.31 H
ATOM 513 HG2 GLU 38 10.842 7.641 22.959
1.00323.99 H
ATOM 514 N CYS 39 15.239 7.631 23.501 1.00
15.84 N
ATOM 515 CA CYS 39 16.687 7.781 23.418 1.00
14.30 C
ATOM 516 C CYS 39 17.072 9.253 23.605 1.00
22.31 C
ATOM 517 O CYS 39 16.291 10.142 23.267 1.00
32.92 O
ATOM 518 CB CYS 39 17.172 7.284 22.054 1.00
18.08 C
ATOM 519 SG CYS 39 16.084 7.929 20.760 1.00
17.41 S
ATOM 520 HN CYS 39 14.748 7.279 22.733 1.00
24.87 H
ATOM 521 HA CYS 39 17.143 7.182 24.191 1.00
14.60 H
ATOM 522 HB1 CYS 39 17.154 6.205 22.036 1.00
27.71 H
ATOM 523 HB2 CYS 39 18.179 7.629 21.882 1.00
34.91 H
ATOM 524 N PRO 40 18.240 9.535 24.135 1.00
27.27 N
ATOM 525 CA PRO 40 18.691 10.940 24.359 1.00
47.66 C
ATOM 526 C PRO 40 19.105 11.624 23.057 1.00
71.54 C
ATOM 527 O PRO 40 19.845 11.058 22.252 1.00
87.70 O
ATOM 528 CB PRO 40 19.883 10.781 25.310 1.00
53.85 C
ATOM 529 CG PRO 40 20.455 9.445 24.969 1.00
46.04 C
ATOM 530 CD PRO 40 19.261 8.567 24.575 1.00
25.70 C
ATOM 531 HA PRO 40 17.912 11.508 24.845 1.00
58.04 H
ATOM 532 HB1 PRO 40 19.547 10.789 26.337 1.00
67.93 H
ATOM 533 HB2 PRO 40 20.611 11.565 25.144 1.00
70.99 H
ATOM 534 HG1 PRO 40 20.958 9.019 25.824 1.00
65.91 H
ATOM 535 HG2 PRO 40 21.144 9.539 24.138 1.00
54.99 H
ATOM 536 HD1 PRO 40 18.905 8.008 25.426 1.00

24.22 H
ATOM 537 HD2 PRO 40 19.530 7.905 23.766 1.00
26.94 H
ATOM 538 N GLY 41 18.628 12.846 22.870
1.00105.24 N
ATOM 539 CA GLY 41 18.955 13.614 21.674
1.00147.55 C
ATOM 540 C GLY 41 20.447 13.910 21.612
1.00161.74 C
ATOM 541 O GLY 41 21.059 13.861 20.545
1.00244.37 O
ATOM 542 HN GLY 41 18.050 13.240 23.553
1.00121.79 H
ATOM 543 HA1 GLY 41 18.411 14.546 21.691
1.00184.40 H
ATOM 544 HA2 GLY 41 18.669 13.050 20.802
1.00157.34 H
ATOM 545 N LEU 42 21.023 14.222 22.767
1.00162.48 N
ATOM 546 CA LEU 42 22.448 14.534 22.855
1.00191.48 C
ATOM 547 C LEU 42 23.258 13.272 23.132
1.00166.66 C
ATOM 548 O LEU 42 22.724 12.271 23.610
1.00251.38 O
ATOM 549 CB LEU 42 22.688 15.549 23.975
1.00307.99 C
ATOM 550 CG LEU 42 21.729 16.732 23.817
1.00452.28 C
ATOM 551 CD1 LEU 42 21.993 17.750 24.929
1.00681.24 C
ATOM 552 CD2 LEU 42 21.935 17.395 22.446
1.00527.46 C
ATOM 553 HN LEU 42 20.478 14.245 23.581
1.00195.47 H
ATOM 554 HA LEU 42 22.780 14.963 21.919
1.00229.77 H
ATOM 555 HB1 LEU 42 23.707 15.906 23.924
1.00342.49 H
ATOM 556 HB2 LEU 42 22.521 15.074 24.931
1.00335.33 H
ATOM 557 HG LEU 42 20.710 16.378 23.894
1.00427.79 H
ATOM 558 HD11 LEU 42 23.054 17.935 25.002
1.00909.71 H
ATOM 559 HD12 LEU 42 21.630 17.361 25.869
1.00706.80 H
ATOM 560 HD13 LEU 42 21.481 18.674 24.701
1.00858.82 H
ATOM 561 HD21 LEU 42 21.412 16.828 21.690
1.00631.69 H
ATOM 562 HD22 LEU 42 22.988 17.425 22.211

```
1.00628.06           H
ATOM    563 HD23 LEU    42       21.544  18.403  22.468
1.00656.75           H
ATOM    564  N   THR    43       24.551  13.328  22.829
1.00178.52           N
ATOM    565  CA  THR    43       25.428  12.186  23.050
1.00240.84           C
ATOM    566  C   THR    43       25.550  11.894  24.550
1.00372.31           C
ATOM    567  O   THR    43       25.455  12.809  25.367
1.00500.13           O
ATOM    568  CB  THR    43       26.813  12.496  22.469
1.00354.13           C
ATOM    569  CG2 THR    43       26.676  12.866  20.991
1.00463.04           C
ATOM    570  OG1 THR    43       27.388  13.584  23.179
1.00508.50           O
ATOM    571  HN  THR    43       24.920  14.154  22.451
1.00229.59           H
ATOM    572  HA  THR    43       25.017  11.329  22.542
1.00249.90           H
ATOM    573  HB  THR    43       27.450  11.632  22.560
1.00481.58           H
ATOM    574  HG1 THR    43       28.342  13.514  23.109
1.00624.31           H
ATOM    575 HG21 THR    43       27.656  13.005  20.561
1.00650.95           H
ATOM    576 HG22 THR    43       26.109  13.782  20.901
1.00580.73           H
ATOM    577 HG23 THR    43       26.163  12.072  20.467
1.00571.99           H
ATOM    578  N   PRO    44       25.759  10.653  24.937
1.00488.39           N
ATOM    579  CA  PRO    44       25.894  10.280  26.377
1.00760.13           C
ATOM    580  C   PRO    44       27.244  10.711  26.943
1.00735.36           C
ATOM    581  O   PRO    44       27.491  10.600  28.144
1.00999.99           O
ATOM    582  CB  PRO    44       25.750   8.755  26.365
1.00999.99           C
ATOM    583  CG  PRO    44       26.269   8.339  25.029
1.00856.94           C
ATOM    584  CD  PRO    44       25.892   9.468  24.063
1.00548.09           C
ATOM    585  HA  PRO    44       25.093  10.717  26.952
1.00939.00           H
ATOM    586  HB1 PRO    44       24.711   8.474  26.462
1.00999.99           H
ATOM    587  HB2 PRO    44       26.335   8.309  27.160
1.00999.99           H
ATOM    588  HG1 PRO    44       25.808   7.417  24.713
```

```
1.00999.99           H
ATOM    589  HG2 PRO    44       27.346   8.222  25.071
1.00853.53           H
ATOM    590  HD1 PRO    44       24.951   9.254  23.578
1.00602.45           H
ATOM    591  HD2 PRO    44       26.674   9.614  23.333
1.00463.32           H
ATOM    592  N   ILE    45       28.116  11.197  26.066
1.00518.33           N
ATOM    593  CA  ILE    45       29.441  11.634  26.481
1.00582.79           C
ATOM    594  C   ILE    45       29.337  12.784  27.477
1.00795.93           C
ATOM    595  O   ILE    45       28.635  13.767  27.236
1.00898.75           O
ATOM    596  CB  ILE    45       30.245  12.088  25.262
1.00474.41           C
ATOM    597  CG1 ILE    45       30.452  10.900  24.318
1.00516.90           C
ATOM    598  CG2 ILE    45       31.605  12.621  25.716
1.00760.75           C
ATOM    599  CD1 ILE    45       31.014  11.397  22.985
1.00566.81           C
ATOM    600  HN  ILE    45       27.864  11.256  25.120
1.00395.72           H
ATOM    601  HA  ILE    45       29.952  10.808  26.947
1.00711.06           H
ATOM    602  HB  ILE    45       29.706  12.870  24.747
1.00477.81           H
ATOM    603 HG11 ILE    45       29.506  10.409  24.146
1.00624.28           H
ATOM    604 HG12 ILE    45       31.144  10.203  24.766
1.00744.84           H
ATOM    605 HG21 ILE    45       32.050  11.924  26.413
1.00920.57           H
ATOM    606 HG22 ILE    45       31.474  13.578  26.197
1.00999.99           H
ATOM    607 HG23 ILE    45       32.252  12.735  24.858
1.00865.46           H
ATOM    608 HD11 ILE    45       31.029  10.585  22.276
1.00658.89           H
ATOM    609 HD12 ILE    45       32.019  11.766  23.134
1.00734.30           H
ATOM    610 HD13 ILE    45       30.392  12.195  22.607
1.00675.84           H
ATOM    611  N   ALA    46       30.040  12.655  28.597
1.00999.99           N
ATOM    612  CA  ALA    46       30.019  13.691  29.625
1.00999.99           C
ATOM    613  C   ALA    46       28.586  14.107  29.937
1.00999.99           C
ATOM    614  CB  ALA    46       30.817  14.908  29.155
```

```
1.00999.99           C
ATOM    615  OT1 ALA    46       27.681  13.405  29.516
1.00999.99           O
ATOM    616  OT2 ALA    46       28.414  15.121  30.590
1.00999.99           O
ATOM    617  HN  ALA    46       30.582  11.851  28.735
1.00999.99           H
ATOM    618  HA  ALA    46       30.475  13.301  30.522
1.00999.99           H
ATOM    619  HB1 ALA    46       30.293  15.391  28.344
1.00999.99           H
ATOM    620  HB2 ALA    46       31.791  14.591  28.814
1.00999.99           H
ATOM    621  HB3 ALA    46       30.931  15.602  29.974
1.00999.99           H
ENDMDL
TER
MODEL       13
ATOM      1  N   GLY     1       24.379  -4.581  19.094
1.00999.99           N
ATOM      2  CA  GLY     1       24.678  -3.266  19.730
1.00999.99           C
ATOM      3  C   GLY     1       24.198  -2.140  18.823
1.00999.99           C
ATOM      4  O   GLY     1       23.713  -1.112  19.295
1.00999.99           O
ATOM      5  HA1 GLY     1       25.742  -3.174  19.882
1.00999.99           H
ATOM      6  HA2 GLY     1       24.171  -3.205  20.683
1.00999.99           H
ATOM      7  HT1 GLY     1       24.185  -4.441  18.083
1.00999.99           H
ATOM      8  HT2 GLY     1       23.547  -5.006  19.552
1.00999.99           H
ATOM      9  HT3 GLY     1       25.196  -5.214  19.204
1.00999.99           H
ATOM     10  N   LEU     2       24.335  -2.341  17.516
1.00999.99           N
ATOM     11  CA  LEU     2       23.911  -1.336  16.548
1.00895.59           C
ATOM     12  C   LEU     2       22.389  -1.299  16.457
1.00532.27           C
ATOM     13  O   LEU     2       21.721  -2.310  16.679
1.00625.21           O
ATOM     14  CB  LEU     2       24.498  -1.652  15.170
1.00999.99           C
ATOM     15  CG  LEU     2       26.031  -1.707  15.254
1.00999.99           C
ATOM     16  CD1 LEU     2       26.593  -2.106  13.886
1.00999.99           C
ATOM     17  CD2 LEU     2       26.589  -0.333  15.664
1.00999.99           C
```

```
ATOM     18  HN  LEU     2       24.728  -3.181  17.198
1.00999.99           H
ATOM     19  HA  LEU     2       24.263  -0.367  16.867
1.00890.87           H
ATOM     20  HB1 LEU     2       24.207  -0.884  14.470
1.00924.07           H
ATOM     21  HB2 LEU     2       24.123  -2.607  14.833
1.00999.99           H
ATOM     22  HG  LEU     2       26.320  -2.449  15.987
1.00999.99           H
ATOM     23 HD11 LEU     2       26.489  -1.279  13.199
1.00999.99           H
ATOM     24 HD12 LEU     2       26.049  -2.959  13.507
1.00999.99           H
ATOM     25 HD13 LEU     2       27.638  -2.360  13.986
1.00999.99           H
ATOM     26 HD21 LEU     2       25.988   0.452  15.225
1.00999.99           H
ATOM     27 HD22 LEU     2       27.610  -0.236  15.320
1.00999.99           H
ATOM     28 HD23 LEU     2       26.568  -0.242  16.739
1.00999.99           H
ATOM     29  N   CYS     3       21.844  -0.129  16.134
1.00271.28           N
ATOM     30  CA  CYS     3       20.397   0.025  16.023
1.00104.50           C
ATOM     31  C   CYS     3       19.906  -0.459  14.662  1.00
90.98          C
ATOM     32  O   CYS     3       20.109   0.206  13.647
1.00207.28           O
ATOM     33  CB  CYS     3       20.011   1.494  16.206  1.00
37.83          C
ATOM     34  SG  CYS     3       20.523   2.059  17.848  1.00
81.56          S
ATOM     35  HN  CYS     3       22.425   0.643  15.972
1.00296.55           H
ATOM     36  HA  CYS     3       19.920  -0.560  16.797
1.00166.45           H
ATOM     37  HB1 CYS     3       18.941   1.600  16.107  1.00
40.38          H
ATOM     38  HB2 CYS     3       20.502   2.090  15.452  1.00
98.45          H
ATOM     39  N   SER     4       19.251  -1.614  14.653  1.00
85.31          N
ATOM     40  CA  SER     4       18.725  -2.174  13.413  1.00
92.94          C
ATOM     41  C   SER     4       17.683  -1.239  12.827  1.00
67.37          C
ATOM     42  O   SER     4       17.637  -1.007  11.619
1.00102.28           O
ATOM     43  CB  SER     4       18.056  -3.518  13.687
1.00123.83           C
```

```
ATOM     44  OG  SER     4       17.673  -4.111  12.453
1.00178.89          O
ATOM     45  HN  SER     4       19.114  -2.097  15.496
1.00163.03          H
ATOM     46  HA  SER     4       19.529  -2.312  12.707
1.00129.78          H
ATOM     47  HB1 SER     4       17.176  -3.353  14.301
1.00105.26          H
ATOM     48  HB2 SER     4       18.739  -4.171  14.202
1.00156.87          H
ATOM     49  HG  SER     4       18.067  -3.597  11.742
1.00218.16          H
ATOM     50  N   GLU     5       16.833  -0.724  13.707  1.00
41.67          N
ATOM     51  CA  GLU     5       15.755   0.173  13.311  1.00
41.21          C
ATOM     52  C   GLU     5       15.564   1.269  14.355  1.00
41.92          C
ATOM     53  O   GLU     5       16.422   1.472  15.215  1.00
74.72          O
ATOM     54  CB  GLU     5       14.467  -0.630  13.158  1.00
49.24          C
ATOM     55  CG  GLU     5       14.160  -1.336  14.474  1.00
55.48          C
ATOM     56  CD  GLU     5       13.027  -2.338  14.281  1.00
92.06          C
ATOM     57  OE1 GLU     5       12.670  -2.991  15.248
1.00206.49          O
ATOM     58  OE2 GLU     5       12.532  -2.435  13.171
1.00197.24          O1-
ATOM     59  HN  GLU     5       16.923  -0.969  14.652  1.00
45.52          H
ATOM     60  HA  GLU     5       15.994   0.623  12.369  1.00
60.63          H
ATOM     61  HB1 GLU     5       14.589  -1.367  12.379  1.00
61.59          H
ATOM     62  HB2 GLU     5       13.660   0.029  12.907  1.00
63.66          H
ATOM     63  HG1 GLU     5       13.872  -0.604  15.213  1.00
58.03          H
ATOM     64  HG2 GLU     5       15.045  -1.849  14.806  1.00
57.77          H
ATOM     65  N   ASN     6       14.440   1.972  14.276  1.00
50.73          N
ATOM     66  CA  ASN     6       14.160   3.043  15.225  1.00
71.04          C
ATOM     67  C   ASN     6       14.120   2.493  16.647  1.00
65.17          C
ATOM     68  O   ASN     6       14.613   3.124  17.583  1.00
94.86          O
ATOM     69  CB  ASN     6       12.820   3.701  14.891  1.00
94.83          C
```

```
ATOM     70  CG  ASN     6      12.946   4.520  13.611
1.00160.94           C
ATOM     71  ND2 ASN     6      11.873   4.831  12.938
1.00244.88           N
ATOM     72  OD1 ASN     6      14.052   4.888  13.214
1.00219.73           O
ATOM     73  HN  ASN     6      13.790   1.769  13.571  1.00
72.81           H
ATOM     74  HA  ASN     6      14.942   3.786  15.157  1.00
96.02           H
ATOM     75  HB1 ASN     6      12.527   4.351  15.703
1.00111.09           H
ATOM     76  HB2 ASN     6      12.069   2.936  14.755  1.00
98.13           H
ATOM     77 HD21 ASN     6      10.993   4.538  13.256
1.00272.43           H
ATOM     78 HD22 ASN     6      11.945   5.356  12.114
1.00336.78           H
ATOM     79  N   GLY     7      13.532   1.308  16.800  1.00
51.80           N
ATOM     80  CA  GLY     7      13.429   0.658  18.109  1.00
71.47           C
ATOM     81  C   GLY     7      14.064  -0.726  18.070  1.00
41.04           C
ATOM     82  O   GLY     7      13.381  -1.739  18.214  1.00
44.54           O
ATOM     83  HN  GLY     7      13.162   0.855  16.014  1.00
45.49           H
ATOM     84  HA1 GLY     7      12.392   0.557  18.374
1.00103.52           H
ATOM     85  HA2 GLY     7      13.930   1.257  18.860
1.00108.67           H
ATOM     86  N   ASP     8      15.373  -0.755  17.869  1.00
27.00           N
ATOM     87  CA  ASP     8      16.100  -2.017  17.806  1.00
14.15           C
ATOM     88  C   ASP     8      15.962  -2.786  19.112  1.00
9.37           C
ATOM     89  O   ASP     8      15.716  -3.992  19.112  1.00
14.61           O
ATOM     90  CB  ASP     8      17.580  -1.757  17.518  1.00
15.06           C
ATOM     91  CG  ASP     8      18.323  -3.080  17.373  1.00
21.22           C
ATOM     92  OD1 ASP     8      19.488  -3.045  17.008
1.00119.56           O
ATOM     93  OD2 ASP     8      17.719  -4.109  17.628
1.00133.27           O1-
ATOM     94  HN  ASP     8      15.856   0.088  17.760  1.00
35.63           H
ATOM     95  HA  ASP     8      15.692  -2.614  17.008  1.00
20.92           H
```

ATOM 96 HB1 ASP 8 18.007 -1.195 18.335 1.00
29.39 H
ATOM 97 HB2 ASP 8 17.672 -1.190 16.604 1.00
44.14 H
ATOM 98 N CYS 9 16.117 -2.080 20.224 1.00
5.93 N
ATOM 99 CA CYS 9 15.998 -2.711 21.533 1.00
7.54 C
ATOM 100 C CYS 9 14.592 -3.270 21.707 1.00
14.60 C
ATOM 101 O CYS 9 14.401 -4.385 22.192 1.00
25.78 O
ATOM 102 CB CYS 9 16.281 -1.694 22.627 1.00
6.63 C
ATOM 103 SG CYS 9 18.029 -1.274 22.580 1.00
10.91 S
ATOM 104 HN CYS 9 16.307 -1.123 20.161 1.00
6.51 H
ATOM 105 HA CYS 9 16.721 -3.504 21.608 1.00
11.12 H
ATOM 106 HB1 CYS 9 16.041 -2.119 23.588 1.00
11.28 H
ATOM 107 HB2 CYS 9 15.686 -0.806 22.463 1.00
5.04 H
ATOM 108 N ALA 10 13.616 -2.472 21.292 1.00
16.26 N
ATOM 109 CA ALA 10 12.212 -2.856 21.374 1.00
32.04 C
ATOM 110 C ALA 10 11.363 -1.837 20.618 1.00
45.47 C
ATOM 111 O ALA 10 11.821 -0.729 20.338
1.00119.04 O
ATOM 112 CB ALA 10 11.768 -2.929 22.838 1.00
30.10 C
ATOM 113 HN ALA 10 13.848 -1.602 20.910 1.00
12.81 H
ATOM 114 HA ALA 10 12.086 -3.826 20.918 1.00
47.14 H
ATOM 115 HB1 ALA 10 11.545 -1.938 23.201 1.00
84.37 H
ATOM 116 HB2 ALA 10 12.557 -3.362 23.433
1.00118.20 H
ATOM 117 HB3 ALA 10 10.883 -3.545 22.914
1.00117.32 H
ATOM 118 N ALA 11 10.135 -2.209 20.283 1.00
30.62 N
ATOM 119 CA ALA 11 9.260 -1.297 19.553 1.00
35.24 C
ATOM 120 C ALA 11 9.021 -0.022 20.358 1.00
22.78 C
ATOM 121 O ALA 11 9.098 1.083 19.823 1.00
53.33 O

```
ATOM    122  CB  ALA    11       7.921  -1.979  19.264  1.00
60.49          C
ATOM    123  HN  ALA    11       9.814  -3.102  20.525  1.00
50.37          H
ATOM    124  HA  ALA    11       9.728  -1.039  18.616  1.00
41.70          H
ATOM    125  HB1 ALA    11       7.527  -2.400  20.175
1.00157.06          H
ATOM    126  HB2 ALA    11       8.067  -2.765  18.537
1.00148.83          H
ATOM    127  HB3 ALA    11       7.224  -1.253  18.872
1.00137.06          H
ATOM    128  N   ASP    12       8.736  -0.183  21.646  1.00
18.98          N
ATOM    129  CA  ASP    12       8.492   0.962  22.515  1.00
31.05          C
ATOM    130  C   ASP    12       9.788   1.722  22.800  1.00
22.98          C
ATOM    131  O   ASP    12       9.798   2.951  22.864  1.00
37.14          O
ATOM    132  CB  ASP    12       7.876   0.494  23.835  1.00
48.35          C
ATOM    133  CG  ASP    12       6.446   0.019  23.604
1.00104.83          C
ATOM    134  OD1 ASP    12       5.911   0.305  22.546
1.00258.73          O
ATOM    135  OD2 ASP    12       5.906  -0.626  24.489
1.00213.46          O1-
ATOM    136  HN  ASP    12       8.691  -1.089  22.019  1.00
36.19          H
ATOM    137  HA  ASP    12       7.797   1.631  22.028  1.00
49.76          H
ATOM    138  HB1 ASP    12       7.871   1.313  24.537  1.00
95.71          H
ATOM    139  HB2 ASP    12       8.465  -0.320  24.236  1.00
54.37          H
ATOM    140  N   GLU    13      10.873   0.976  22.985  1.00
17.60          N
ATOM    141  CA  GLU    13      12.172   1.577  23.278  1.00
12.01          C
ATOM    142  C   GLU    13      12.771   2.229  22.033  1.00
10.07          C
ATOM    143  O   GLU    13      12.367   1.927  20.910  1.00
12.81          O
ATOM    144  CB  GLU    13      13.118   0.505  23.818  1.00
11.74          C
ATOM    145  CG  GLU    13      12.550  -0.042  25.130  1.00
13.49          C
ATOM    146  CD  GLU    13      13.418  -1.183  25.645
1.00142.36          C
ATOM    147  OE1 GLU    13      14.533  -1.314  25.172
1.00339.06          O
```

ATOM 148 OE2 GLU 13 12.953 -1.914 26.504
1.00335.28 O1-
ATOM 149 HN GLU 13 10.798 0.000 22.930 1.00
28.36 H
ATOM 150 HA GLU 13 12.041 2.332 24.040 1.00
13.06 H
ATOM 151 HB1 GLU 13 14.090 0.938 23.999 1.00
13.76 H
ATOM 152 HB2 GLU 13 13.207 -0.294 23.096 1.00
9.94 H
ATOM 153 HG1 GLU 13 11.546 -0.403 24.965 1.00
66.02 H
ATOM 154 HG2 GLU 13 12.529 0.748 25.866 1.00
56.23 H
ATOM 155 N CYS 14 13.737 3.130 22.241 1.00
9.12 N
ATOM 156 CA CYS 14 14.392 3.833 21.134 1.00
10.69 C
ATOM 157 C CYS 14 15.825 3.344 20.970 1.00
9.56 C
ATOM 158 O CYS 14 16.428 2.849 21.918 1.00
13.62 O
ATOM 159 CB CYS 14 14.402 5.340 21.404 1.00
13.96 C
ATOM 160 SG CYS 14 15.288 6.185 20.066 1.00
42.96 S
ATOM 161 HN CYS 14 14.017 3.328 23.160 1.00
9.76 H
ATOM 162 HA CYS 14 13.853 3.648 20.215 1.00
15.54 H
ATOM 163 HB1 CYS 14 14.897 5.535 22.343 1.00
50.72 H
ATOM 164 HB2 CYS 14 13.387 5.704 21.450 1.00
46.13 H
ATOM 165 N CYS 15 16.364 3.490 19.761 1.00
9.60 N
ATOM 166 CA CYS 15 17.735 3.065 19.479 1.00
9.28 C
ATOM 167 C CYS 15 18.332 3.956 18.395 1.00
9.92 C
ATOM 168 O CYS 15 17.724 4.153 17.343 1.00
13.31 O
ATOM 169 CB CYS 15 17.747 1.607 19.011 1.00
12.90 C
ATOM 170 SG CYS 15 19.430 0.941 19.128 1.00
39.11 S
ATOM 171 HN CYS 15 15.832 3.896 19.045 1.00
13.12 H
ATOM 172 HA CYS 15 18.332 3.155 20.376 1.00
8.26 H
ATOM 173 HB1 CYS 15 17.412 1.555 17.984 1.00
24.82 H

```
ATOM    174  HB2 CYS    15      17.085   1.024  19.632  1.00
27.31           H
ATOM    175  N   VAL    16      19.522   4.497  18.651  1.00
9.56           N
ATOM    176  CA  VAL    16      20.176   5.366  17.677  1.00
11.99           C
ATOM    177  C   VAL    16      21.690   5.230  17.773  1.00
7.73           C
ATOM    178  O   VAL    16      22.260   5.278  18.863  1.00
8.83           O
ATOM    179  CB  VAL    16      19.775   6.820  17.931  1.00
18.37           C
ATOM    180  CG1 VAL    16      20.342   7.283  19.275  1.00
39.61           C
ATOM    181  CG2 VAL    16      20.325   7.705  16.811
1.00115.21           C
ATOM    182  HN  VAL    16      19.966   4.309  19.507  1.00
9.76           H
ATOM    183  HA  VAL    16      19.865   5.090  16.680  1.00
17.27           H
ATOM    184  HB  VAL    16      18.696   6.896  17.953  1.00
52.63           H
ATOM    185 HG11 VAL    16      19.855   8.198  19.575
1.00128.07           H
ATOM    186 HG12 VAL    16      21.405   7.455  19.177
1.00154.13           H
ATOM    187 HG13 VAL    16      20.169   6.521  20.021
1.00135.74           H
ATOM    188 HG21 VAL    16      20.031   8.730  16.985
1.00229.05           H
ATOM    189 HG22 VAL    16      19.930   7.372  15.863
1.00261.82           H
ATOM    190 HG23 VAL    16      21.402   7.637  16.795
1.00210.07           H
ATOM    191  N   ASP    17      22.338   5.061  16.620  1.00
14.34           N
ATOM    192  CA  ASP    17      23.793   4.917  16.576  1.00
11.90           C
ATOM    193  C   ASP    17      24.444   6.217  16.119  1.00
13.15           C
ATOM    194  O   ASP    17      24.202   6.687  15.008  1.00
25.96           O
ATOM    195  CB  ASP    17      24.178   3.796  15.610  1.00
20.80           C
ATOM    196  CG  ASP    17      23.791   2.443  16.200  1.00
28.72           C
ATOM    197  OD1 ASP    17      23.799   1.473  15.460
1.00135.42           O
ATOM    198  OD2 ASP    17      23.492   2.399  17.381
1.00114.17           O1-
ATOM    199  HN  ASP    17      21.828   5.031  15.785  1.00
28.29           H
```

```
ATOM    200  HA  ASP    17       24.164   4.667  17.562  1.00
10.49          H
ATOM    201  HB1 ASP    17       25.243   3.819  15.441  1.00
22.42          H
ATOM    202  HB2 ASP    17       23.662   3.939  14.671  1.00
29.52          H
ATOM    203  N   THR    18       25.282   6.786  16.982  1.00
11.55          N
ATOM    204  CA  THR    18       25.986   8.030  16.664  1.00
17.10          C
ATOM    205  C   THR    18       27.447   7.736  16.362  1.00
10.63          C
ATOM    206  O   THR    18       27.953   6.667  16.698  1.00
6.49          O
ATOM    207  CB  THR    18       25.899   9.000  17.844  1.00
28.52          C
ATOM    208  CG2 THR    18       24.475   9.538  17.967  1.00
45.38          C
ATOM    209  OG1 THR    18       26.263   8.324  19.037  1.00
26.49          O
ATOM    210  HN  THR    18       25.439   6.356  17.848  1.00
13.96          H
ATOM    211  HA  THR    18       25.533   8.494  15.798  1.00
26.43          H
ATOM    212  HB  THR    18       26.576   9.824  17.679  1.00
37.43          H
ATOM    213  HG1 THR    18       25.891   8.809  19.777  1.00
71.62          H
ATOM    214 HG21 THR    18       24.397  10.143  18.859
1.00100.44          H
ATOM    215 HG22 THR    18       23.782   8.712  18.030
1.00129.42          H
ATOM    216 HG23 THR    18       24.242  10.140  17.102
1.00156.21          H
ATOM    217  N   VAL    19       28.119   8.698  15.740  1.00
16.32          N
ATOM    218  CA  VAL    19       29.515   8.559  15.402  1.00
13.34          C
ATOM    219  C   VAL    19       29.975   9.803  14.669  1.00
25.88          C
ATOM    220  O   VAL    19       29.484  10.141  13.591  1.00
42.11          O
ATOM    221  CB  VAL    19       29.773   7.322  14.543  1.00
15.48          C
ATOM    222  CG1 VAL    19       28.816   7.297  13.347  1.00
29.06          C
ATOM    223  CG2 VAL    19       31.223   7.359  14.041  1.00
20.67          C
ATOM    224  HN  VAL    19       27.669   9.534  15.517  1.00
26.89          H
ATOM    225  HA  VAL    19       30.083   8.469  16.317  1.00
8.08          H
```

```
ATOM    226  HB  VAL    19       29.627   6.441  15.142  1.00
11.49           H
ATOM    227 HG11 VAL    19       29.125   8.035  12.622
1.00137.43           H
ATOM    228 HG12 VAL    19       27.813   7.515  13.681  1.00
93.29           H
ATOM    229 HG13 VAL    19       28.837   6.317  12.892
1.00108.69           H
ATOM    230 HG21 VAL    19       31.307   8.080  13.240  1.00
99.42           H
ATOM    231 HG22 VAL    19       31.506   6.383  13.680
1.00111.36           H
ATOM    232 HG23 VAL    19       31.877   7.650  14.853  1.00
77.11           H
ATOM    233  N   PHE    20       30.909  10.478  15.290  1.00
24.58           N
ATOM    234  CA  PHE    20       31.468  11.711  14.760  1.00
40.34           C
ATOM    235  C   PHE    20       32.847  11.477  14.169  1.00
40.37           C
ATOM    236  O   PHE    20       33.073  11.708  12.981  1.00
66.02           O
ATOM    237  CB  PHE    20       31.559  12.746  15.891  1.00
48.79           C
ATOM    238  CG  PHE    20       31.912  12.076  17.211  1.00
35.75           C
ATOM    239  CD1 PHE    20       31.000  11.209  17.846  1.00
30.20           C
ATOM    240  CD2 PHE    20       33.150  12.339  17.814  1.00
40.95           C
ATOM    241  CE1 PHE    20       31.333  10.616  19.066  1.00
30.41           C
ATOM    242  CE2 PHE    20       33.477  11.740  19.036  1.00
45.84           C
ATOM    243  CZ  PHE    20       32.571  10.879  19.661  1.00
40.81           C
ATOM    244  HN  PHE    20       31.219  10.144  16.149  1.00
16.73           H
ATOM    245  HA  PHE    20       30.820  12.100  13.985  1.00
59.91           H
ATOM    246  HB1 PHE    20       30.616  13.227  15.991  1.00
67.15           H
ATOM    247  HB2 PHE    20       32.306  13.488  15.651  1.00
58.17           H
ATOM    248  HD1 PHE    20       30.040  10.994  17.396  1.00
33.34           H
ATOM    249  HD2 PHE    20       33.853  13.001  17.334  1.00
49.35           H
ATOM    250  HE1 PHE    20       30.632   9.951  19.548  1.00
30.61           H
ATOM    251  HE2 PHE    20       34.432  11.943  19.496  1.00
61.27           H
```

```
ATOM    252  HZ  PHE    20      32.824  10.419  20.603  1.00
52.66          H
ATOM    253  N   GLU    21      33.778  11.057  15.016  1.00
41.31          N
ATOM    254  CA  GLU    21      35.143  10.839  14.595  1.00
56.16          C
ATOM    255  C   GLU    21      35.462   9.360  14.448  1.00
58.61          C
ATOM    256  O   GLU    21      34.606   8.497  14.645
1.00201.19          O
ATOM    257  CB  GLU    21      36.040  11.472  15.635  1.00
57.37          C
ATOM    258  CG  GLU    21      35.957  10.693  16.950
1.00193.56          C
ATOM    259  CD  GLU    21      36.664  11.472  18.055
1.00304.62          C
ATOM    260  OE1 GLU    21      36.577  11.055  19.198
1.00451.81          O
ATOM    261  OE2 GLU    21      37.284  12.476  17.741
1.00442.88          O1-
ATOM    262  HN  GLU    21      33.553  10.923  15.955  1.00
50.82          H
ATOM    263  HA  GLU    21      35.319  11.330  13.647  1.00
85.72          H
ATOM    264  HB1 GLU    21      35.705  12.480  15.800  1.00
46.37          H
ATOM    265  HB2 GLU    21      37.051  11.477  15.280
1.00131.81          H
ATOM    266  HG1 GLU    21      36.430   9.734  16.831
1.00314.91          H
ATOM    267  HG2 GLU    21      34.922  10.549  17.216
1.00266.92          H
ATOM    268  N   GLY    22      36.707   9.088  14.089  1.00
86.70          N
ATOM    269  CA  GLY    22      37.172   7.713  13.893
1.00104.66          C
ATOM    270  C   GLY    22      37.805   7.154  15.163  1.00
71.50          C
ATOM    271  O   GLY    22      38.088   5.959  15.253  1.00
89.87          O
ATOM    272  HN  GLY    22      37.328   9.836  13.949
1.00214.64          H
ATOM    273  HA1 GLY    22      37.906   7.699  13.102
1.00152.47          H
ATOM    274  HA2 GLY    22      36.337   7.087  13.609
1.00118.15          H
ATOM    275  N   ASP    23      38.029   8.024  16.139  1.00
44.68          N
ATOM    276  CA  ASP    23      38.634   7.608  17.398  1.00
43.33          C
ATOM    277  C   ASP    23      37.770   6.564  18.096  1.00
37.55          C
```

```
ATOM    278  O   ASP    23       38.278   5.565  18.608  1.00
61.37           O
ATOM    279  CB  ASP    23       38.807   8.818  18.313  1.00
40.74           C
ATOM    280  CG  ASP    23       39.589   8.421  19.561
1.00141.86           C
ATOM    281  OD1 ASP    23       39.968   9.309  20.307
1.00328.18           O
ATOM    282  OD2 ASP    23       39.799   7.235  19.754
1.00304.58           O1-
ATOM    283  HN  ASP    23       37.788   8.964  16.008  1.00
40.86           H
ATOM    284  HA  ASP    23       39.601   7.183  17.195  1.00
72.12           H
ATOM    285  HB1 ASP    23       37.835   9.184  18.604  1.00
94.15           H
ATOM    286  HB2 ASP    23       39.340   9.594  17.786  1.00
93.26           H
ATOM    287  N   MET    24       36.468   6.806  18.116  1.00
23.24           N
ATOM    288  CA  MET    24       35.537   5.882  18.759  1.00
32.50           C
ATOM    289  C   MET    24       34.117   6.093  18.245  1.00
25.13           C
ATOM    290  O   MET    24       33.832   7.074  17.559  1.00
54.40           O
ATOM    291  CB  MET    24       35.572   6.082  20.276  1.00
47.36           C
ATOM    292  CG  MET    24       35.140   7.510  20.617
1.00151.73           C
ATOM    293  SD  MET    24       35.317   7.783  22.398
1.00209.23           S
ATOM    294  CE  MET    24       34.729   9.494  22.427
1.00243.29           C
ATOM    295  HN  MET    24       36.125   7.619  17.692  1.00
17.32           H
ATOM    296  HA  MET    24       35.841   4.869  18.538  1.00
48.94           H
ATOM    297  HB1 MET    24       36.575   5.916  20.639
1.00124.40           H
ATOM    298  HB2 MET    24       34.898   5.380  20.745
1.00166.96           H
ATOM    299  HG1 MET    24       34.108   7.652  20.333
1.00331.71           H
ATOM    300  HG2 MET    24       35.762   8.212  20.081
1.00302.30           H
ATOM    301  HE1 MET    24       34.954   9.968  21.482
1.00340.44           H
ATOM    302  HE2 MET    24       35.219  10.032  23.223
1.00373.88           H
ATOM    303  HE3 MET    24       33.659   9.502  22.594
1.00386.81           H
```

```
ATOM    304  N   VAL    25      33.227   5.159  18.584  1.00
22.44          N
ATOM    305  CA  VAL    25      31.824   5.229  18.162  1.00
15.37          C
ATOM    306  C   VAL    25      30.905   5.324  19.374  1.00
17.47          C
ATOM    307  O   VAL    25      31.164   4.723  20.416  1.00
29.52          O
ATOM    308  CB  VAL    25      31.462   3.993  17.334  1.00
23.36          C
ATOM    309  CG1 VAL    25      29.945   3.974  17.059  1.00
58.39          C
ATOM    310  CG2 VAL    25      32.227   4.035  16.007  1.00
55.25          C
ATOM    311  HN  VAL    25      33.520   4.403  19.134  1.00
48.11          H
ATOM    312  HA  VAL    25      31.676   6.114  17.549  1.00
9.25          H
ATOM    313  HB  VAL    25      31.736   3.103  17.880  1.00
52.45          H
ATOM    314 HG11 VAL    25      29.740   3.396  16.169
1.00171.07          H
ATOM    315 HG12 VAL    25      29.587   4.985  16.923
1.00166.22          H
ATOM    316 HG13 VAL    25      29.435   3.528  17.900
1.00134.76          H
ATOM    317 HG21 VAL    25      31.948   3.184  15.403
1.00141.88          H
ATOM    318 HG22 VAL    25      33.289   4.004  16.203
1.00184.50          H
ATOM    319 HG23 VAL    25      31.985   4.945  15.480
1.00133.90          H
ATOM    320  N   THR    26      29.835   6.101  19.224  1.00
13.60          N
ATOM    321  CA  THR    26      28.866   6.308  20.300  1.00
21.19          C
ATOM    322  C   THR    26      27.480   5.805  19.897  1.00
16.55          C
ATOM    323  O   THR    26      27.030   6.026  18.772  1.00
11.43          O
ATOM    324  CB  THR    26      28.792   7.797  20.617  1.00
27.07          C
ATOM    325  CG2 THR    26      27.757   8.043  21.715  1.00
44.39          C
ATOM    326  OG1 THR    26      30.068   8.245  21.054  1.00
34.75          O
ATOM    327  HN  THR    26      29.698   6.558  18.368  1.00
10.81          H
ATOM    328  HA  THR    26      29.185   5.780  21.188  1.00
33.16          H
ATOM    329  HB  THR    26      28.508   8.335  19.722  1.00
20.60          H
```

```
ATOM    330  HG1 THR    26       30.415   7.594  21.669  1.00
77.72           H
ATOM    331 HG21 THR    26       27.908   9.024  22.137
1.00126.99           H
ATOM    332 HG22 THR    26       27.869   7.300  22.490
1.00108.32           H
ATOM    333 HG23 THR    26       26.763   7.980  21.298
1.00151.99           H
ATOM    334  N   ARG    27       26.805   5.130  20.827  1.00
22.38           N
ATOM    335  CA  ARG    27       25.465   4.595  20.572  1.00
20.21           C
ATOM    336  C   ARG    27       24.631   4.635  21.851  1.00
16.96           C
ATOM    337  O   ARG    27       25.175   4.745  22.949  1.00
20.06           O
ATOM    338  CB  ARG    27       25.568   3.155  20.065  1.00
22.14           C
ATOM    339  CG  ARG    27       26.139   2.261  21.169
1.00124.30           C
ATOM    340  CD  ARG    27       26.465   0.882  20.592
1.00109.38           C
ATOM    341  NE  ARG    27       26.995   0.011  21.637
1.00227.73           N
ATOM    342  CZ  ARG    27       27.504  -1.181  21.345
1.00426.12           C
ATOM    343  NH1 ARG    27       27.965  -1.943  22.297
1.00767.09           N1+
ATOM    344  NH2 ARG    27       27.543  -1.587  20.105
1.00581.78           N
ATOM    345  HN  ARG    27       27.216   4.988  21.705  1.00
30.75           H
ATOM    346  HA  ARG    27       24.977   5.198  19.819  1.00
23.13           H
ATOM    347  HB1 ARG    27       26.220   3.122  19.205  1.00
87.46           H
ATOM    348  HB2 ARG    27       24.586   2.800  19.787
1.00103.36           H
ATOM    349  HG1 ARG    27       25.413   2.154  21.960
1.00281.97           H
ATOM    350  HG2 ARG    27       27.038   2.708  21.565
1.00276.19           H
ATOM    351  HD1 ARG    27       27.198   0.989  19.806
1.00183.60           H
ATOM    352  HD2 ARG    27       25.567   0.444  20.185
1.00142.93           H
ATOM    353  HE  ARG    27       26.973   0.311  22.569
1.00372.53           H
ATOM    354 HH11 ARG    27       27.937  -1.630  23.247
1.00910.59           H
ATOM    355 HH12 ARG    27       28.349  -2.841  22.078
1.00999.99           H
```

```
ATOM    356 HH21 ARG    27       27.191  -1.003  19.375
1.00532.54            H
ATOM    357 HH22 ARG    27       27.926  -2.485  19.886
1.00948.84            H
ATOM    358  N   SER    28       23.307   4.549  21.707  1.00
14.60           N
ATOM    359  CA  SER    28       22.419   4.582  22.869  1.00
14.92           C
ATOM    360  C   SER    28       21.131   3.815  22.590  1.00
12.12           C
ATOM    361  O   SER    28       20.795   3.543  21.438  1.00
13.41           O
ATOM    362  CB  SER    28       22.080   6.031  23.222  1.00
23.26           C
ATOM    363  OG  SER    28       21.310   6.602  22.173
1.00146.32            O
ATOM    364  HN  SER    28       22.921   4.465  20.808  1.00
15.23           H
ATOM    365  HA  SER    28       22.918   4.128  23.711  1.00
15.44           H
ATOM    366  HB1 SER    28       22.998   6.591  23.354  1.00
88.73           H
ATOM    367  HB2 SER    28       21.511   6.058  24.135
1.00124.68            H
ATOM    368  HG  SER    28       20.401   6.311  22.281
1.00242.47            H
ATOM    369  N   CYS    29       20.412   3.472  23.655  1.00
10.48           N
ATOM    370  CA  CYS    29       19.160   2.740  23.517  1.00
9.97           C
ATOM    371  C   CYS    29       18.411   2.729  24.848  1.00
11.60           C
ATOM    372  O   CYS    29       18.987   2.429  25.894  1.00
15.64           O
ATOM    373  CB  CYS    29       19.439   1.303  23.050  1.00
9.52           C
ATOM    374  SG  CYS    29       18.027   0.685  22.122  1.00
10.40           S
ATOM    375  HN  CYS    29       20.728   3.722  24.548  1.00
11.10           H
ATOM    376  HA  CYS    29       18.546   3.236  22.781  1.00
11.08           H
ATOM    377  HB1 CYS    29       19.605   0.657  23.901  1.00
9.33           H
ATOM    378  HB2 CYS    29       20.310   1.289  22.414  1.00
10.78           H
ATOM    379  N   GLU    30       17.128   3.066  24.801  1.00
13.52           N
ATOM    380  CA  GLU    30       16.311   3.100  26.008  1.00
17.41           C
ATOM    381  C   GLU    30       15.895   1.699  26.433  1.00
10.81           C
```

```
ATOM    382  O   GLU    30       15.803   0.788  25.610  1.00
23.22          O
ATOM    383  CB  GLU    30       15.057   3.938  25.768  1.00
37.83          C
ATOM    384  CG  GLU    30       15.457   5.371  25.430
1.00108.13          C
ATOM    385  CD  GLU    30       16.042   6.049  26.665
1.00240.52          C
ATOM    386  OE1 GLU    30       15.900   5.496  27.744
1.00422.56          O
ATOM    387  OE2 GLU    30       16.623   7.112  26.516
1.00410.81          O1-
ATOM    388  HN  GLU    30       16.723   3.301  23.940  1.00
15.61          H
ATOM    389  HA  GLU    30       16.882   3.551  26.804  1.00
24.60          H
ATOM    390  HB1 GLU    30       14.447   3.938  26.660  1.00
72.67          H
ATOM    391  HB2 GLU    30       14.494   3.519  24.948  1.00
56.47          H
ATOM    392  HG1 GLU    30       14.587   5.914  25.102
1.00196.66          H
ATOM    393  HG2 GLU    30       16.195   5.359  24.642
1.00140.67          H
ATOM    394  N   LYS    31       15.628   1.543  27.727  1.00
11.96          N
ATOM    395  CA  LYS    31       15.196   0.259  28.278  1.00
11.40          C
ATOM    396  C   LYS    31       13.840   0.422  28.945  1.00
10.99          C
ATOM    397  O   LYS    31       13.587   1.413  29.630  1.00
12.86          O
ATOM    398  CB  LYS    31       16.205  -0.242  29.309  1.00
21.40          C
ATOM    399  CG  LYS    31       17.563  -0.453  28.640  1.00
57.81          C
ATOM    400  CD  LYS    31       18.563  -0.981  29.668
1.00115.52          C
ATOM    401  CE  LYS    31       19.917  -1.211  28.993
1.00250.23          C
ATOM    402  NZ  LYS    31       20.447   0.089  28.492
1.00462.10          N1+
ATOM    403  HN  LYS    31       15.710   2.314  28.326  1.00
26.13          H
ATOM    404  HA  LYS    31       15.114  -0.473  27.485  1.00
13.87          H
ATOM    405  HB1 LYS    31       15.861  -1.178  29.722  1.00
37.63          H
ATOM    406  HB2 LYS    31       16.298   0.486  30.099  1.00
47.19          H
ATOM    407  HG1 LYS    31       17.920   0.487  28.244
1.00126.32          H
```

```
ATOM    408  HG2 LYS    31       17.461  -1.168  27.835
1.00114.19            H
ATOM    409  HD1 LYS    31       18.203  -1.916  30.073
1.00198.45            H
ATOM    410  HD2 LYS    31       18.674  -0.261  30.466
1.00200.14            H
ATOM    411  HE1 LYS    31       19.795  -1.894  28.166
1.00374.64            H
ATOM    412  HE2 LYS    31       20.609  -1.631  29.708
1.00403.27            H
ATOM    413  HZ1 LYS    31       21.474   0.125  28.641
1.00627.38            H
ATOM    414  HZ2 LYS    31       19.991   0.869  29.008
1.00622.26            H
ATOM    415  HZ3 LYS    31       20.243   0.177  27.477
1.00619.20            H
ATOM    416  N   THR    32       12.968  -0.553  28.740  1.00
15.51           N
ATOM    417  CA  THR    32       11.633  -0.505  29.325  1.00
22.44           C
ATOM    418  C   THR    32       11.673  -0.933  30.789  1.00
26.60           C
ATOM    419  O   THR    32       12.120  -2.033  31.114  1.00
57.43           O
ATOM    420  CB  THR    32       10.687  -1.420  28.548  1.00
56.28           C
ATOM    421  CG2 THR    32        9.305  -1.404  29.200
1.00102.70            C
ATOM    422  OG1 THR    32       10.587  -0.962  27.206
1.00111.17            O
ATOM    423  HN  THR    32       13.226  -1.315  28.184  1.00
18.79           H
ATOM    424  HA  THR    32       11.261   0.508  29.269  1.00
19.67           H
ATOM    425  HB  THR    32       11.072  -2.426  28.555  1.00
84.25           H
ATOM    426  HG1 THR    32       10.548  -1.730  26.633
1.00205.03            H
ATOM    427 HG21 THR    32        8.593  -1.890  28.549
1.00220.71            H
ATOM    428 HG22 THR    32        8.997  -0.383  29.370
1.00174.67            H
ATOM    429 HG23 THR    32        9.345  -1.929  30.143
1.00217.44            H
ATOM    430  N   THR    33       11.198  -0.054  31.665  1.00
22.47           N
ATOM    431  CA  THR    33       11.176  -0.341  33.095  1.00
41.60           C
ATOM    432  C   THR    33       10.098   0.457  33.777  1.00
38.03           C
ATOM    433  O   THR    33       10.332   1.555  34.280  1.00
52.73           O
```

```
ATOM    434  CB  THR    33      12.531  -0.025  33.730  1.00
63.25          C
ATOM    435  CG2 THR    33      13.583  -1.020  33.233
1.00121.65          C
ATOM    436  OG1 THR    33      12.921   1.294  33.378  1.00
96.20          O
ATOM    437  HN  THR    33      10.854   0.805  31.343  1.00
23.82          H
ATOM    438  HA  THR    33      10.945  -1.377  33.253  1.00
67.12          H
ATOM    439  HB  THR    33      12.450  -0.102  34.804
1.00107.59          H
ATOM    440  HG1 THR    33      12.589   1.892  34.053
1.00176.27          H
ATOM    441 HG21 THR    33      13.853  -0.778  32.216
1.00255.23          H
ATOM    442 HG22 THR    33      13.183  -2.020  33.271
1.00245.37          H
ATOM    443 HG23 THR    33      14.459  -0.958  33.861
1.00185.78          H
ATOM    444  N   GLY    34       8.911  -0.125  33.806  1.00
40.93          N
ATOM    445  CA  GLY    34       7.795   0.519  34.447  1.00
45.59          C
ATOM    446  C   GLY    34       7.291   1.697  33.619  1.00
40.97          C
ATOM    447  O   GLY    34       6.096   1.807  33.341  1.00
82.17          O
ATOM    448  HN  GLY    34       8.796  -1.008  33.401  1.00
54.00          H
ATOM    449  HA1 GLY    34       8.127   0.872  35.404  1.00
54.41          H
ATOM    450  HA2 GLY    34       6.997  -0.196  34.582  1.00
62.09          H
ATOM    451  N   ASN    35       8.213   2.575  33.223  1.00
32.51          N
ATOM    452  CA  ASN    35       7.866   3.748  32.416  1.00
33.20          C
ATOM    453  C   ASN    35       8.811   3.872  31.225  1.00
23.17          C
ATOM    454  O   ASN    35      10.021   3.690  31.360  1.00
27.01          O
ATOM    455  CB  ASN    35       7.958   5.012  33.272  1.00
54.51          C
ATOM    456  CG  ASN    35       6.993   4.917  34.448  1.00
80.73          C
ATOM    457  ND2 ASN    35       7.386   5.294  35.633
1.00220.46          N
ATOM    458  OD1 ASN    35       5.851   4.488  34.283
1.00117.80          O
ATOM    459  HN  ASN    35       9.148   2.429  33.472  1.00
51.15          H
```

```
ATOM    460  HA  ASN    35       6.853   3.649  32.047  1.00
47.21          H
ATOM    461  HB1 ASN    35       7.702   5.871  32.669  1.00
68.31          H
ATOM    462  HB2 ASN    35       8.966   5.121  33.640  1.00
57.75          H
ATOM    463 HD21 ASN    35       8.295   5.636  35.762
1.00405.39          H
ATOM    464 HD22 ASN    35       6.772   5.237  36.395
1.00245.64          H
ATOM    465  N   PHE    36       8.249   4.175  30.061  1.00
35.72          N
ATOM    466  CA  PHE    36       9.036   4.318  28.848  1.00
33.41          C
ATOM    467  C   PHE    36       9.629   5.722  28.758  1.00
28.51          C
ATOM    468  O   PHE    36       9.049   6.682  29.267  1.00
45.23          O
ATOM    469  CB  PHE    36       8.137   4.062  27.644  1.00
67.53          C
ATOM    470  CG  PHE    36       7.604   2.650  27.708  1.00
86.71          C
ATOM    471  CD1 PHE    36       6.406   2.388  28.383
1.00106.51          C
ATOM    472  CD2 PHE    36       8.303   1.603  27.092  1.00
99.48          C
ATOM    473  CE1 PHE    36       5.904   1.083  28.440
1.00134.48          C
ATOM    474  CE2 PHE    36       7.801   0.298  27.151
1.00134.27          C
ATOM    475  CZ  PHE    36       6.603   0.038  27.823
1.00149.48          C
ATOM    476  HN  PHE    36       7.281   4.303  30.012  1.00
62.87          H
ATOM    477  HA  PHE    36       9.836   3.593  28.851  1.00
28.53          H
ATOM    478  HB1 PHE    36       8.702   4.192  26.742  1.00
73.56          H
ATOM    479  HB2 PHE    36       7.312   4.758  27.657  1.00
88.42          H
ATOM    480  HD1 PHE    36       5.869   3.196  28.859
1.00110.20          H
ATOM    481  HD2 PHE    36       9.230   1.802  26.573  1.00
93.07          H
ATOM    482  HE1 PHE    36       4.979   0.882  28.960
1.00155.06          H
ATOM    483  HE2 PHE    36       8.339  -0.509  26.674
1.00159.46          H
ATOM    484  HZ  PHE    36       6.215  -0.969  27.868
1.00183.25          H
ATOM    485  N   THR    37      10.788   5.839  28.108  1.00
20.06          N
```

ATOM 486 CA THR 37 11.461 7.128 27.955 1.00
27.18 C
ATOM 487 C THR 37 11.770 7.392 26.486 1.00
41.87 C
ATOM 488 O THR 37 11.007 7.009 25.600
1.00169.86 O
ATOM 489 CB THR 37 12.762 7.134 28.759 1.00
26.52 C
ATOM 490 CG2 THR 37 12.481 6.680 30.191 1.00
75.69 C
ATOM 491 OG1 THR 37 13.694 6.250 28.153 1.00
81.95 O
ATOM 492 HN THR 37 11.207 5.042 27.725 1.00
18.47 H
ATOM 493 HA THR 37 10.821 7.921 28.323 1.00
38.80 H
ATOM 494 HB THR 37 13.172 8.133 28.777 1.00
81.60 H
ATOM 495 HG1 THR 37 13.787 6.501 27.231
1.00188.80 H
ATOM 496 HG21 THR 37 11.683 7.275 30.608
1.00196.98 H
ATOM 497 HG22 THR 37 13.372 6.802 30.790
1.00196.23 H
ATOM 498 HG23 THR 37 12.190 5.641 30.189
1.00168.92 H
ATOM 499 N GLU 38 12.895 8.054 26.239 1.00
26.53 N
ATOM 500 CA GLU 38 13.307 8.378 24.873 1.00
30.29 C
ATOM 501 C GLU 38 14.830 8.471 24.786 1.00
25.39 C
ATOM 502 O GLU 38 15.482 9.012 25.679 1.00
51.60 O
ATOM 503 CB GLU 38 12.677 9.709 24.445 1.00
54.82 C
ATOM 504 CG GLU 38 13.043 10.029 22.989
1.00165.84 C
ATOM 505 CD GLU 38 12.475 8.965 22.056
1.00292.92 C
ATOM 506 OE1 GLU 38 11.545 8.286 22.459
1.00414.19 O
ATOM 507 OE2 GLU 38 12.979 8.844 20.951
1.00537.65 O1-
ATOM 508 HN GLU 38 13.456 8.331 26.990 1.00
84.50 H
ATOM 509 HA GLU 38 12.968 7.598 24.208 1.00
32.15 H
ATOM 510 HB1 GLU 38 13.039 10.498 25.085
1.00144.82 H
ATOM 511 HB2 GLU 38 11.602 9.639 24.535
1.00106.49 H

```
ATOM    512  HG1 GLU    38       14.117  10.062  22.884
1.00303.31           H
ATOM    513  HG2 GLU    38       12.631  10.991  22.723
1.00323.99           H
ATOM    514  N   CYS    39       15.389   7.938  23.700  1.00
15.84          N
ATOM    515  CA  CYS    39       16.834   7.965  23.500  1.00
14.30          C
ATOM    516  C   CYS    39       17.347   9.410  23.545  1.00
22.31          C
ATOM    517  O   CYS    39       16.615  10.338  23.203  1.00
32.92          O
ATOM    518  CB  CYS    39       17.173   7.328  22.148  1.00
18.08          C
ATOM    519  SG  CYS    39       15.981   7.889  20.906  1.00
17.41          S
ATOM    520  HN  CYS    39       14.817   7.521  23.023  1.00
24.87          H
ATOM    521  HA  CYS    39       17.300   7.388  24.283  1.00
14.60          H
ATOM    522  HB1 CYS    39       17.123   6.253  22.235  1.00
27.71          H
ATOM    523  HB2 CYS    39       18.169   7.618  21.850  1.00
34.91          H
ATOM    524  N   PRO    40       18.577   9.625  23.960  1.00
27.27          N
ATOM    525  CA  PRO    40       19.165  10.994  24.047  1.00
47.66          C
ATOM    526  C   PRO    40       19.537  11.550  22.673  1.00
71.54          C
ATOM    527  O   PRO    40       19.743  10.802  21.720  1.00
87.70          O
ATOM    528  CB  PRO    40       20.407  10.790  24.920  1.00
53.85          C
ATOM    529  CG  PRO    40       20.838   9.395  24.623  1.00
46.04          C
ATOM    530  CD  PRO    40       19.549   8.600  24.391  1.00
25.70          C
ATOM    531  HA  PRO    40       18.479  11.663  24.544  1.00
58.04          H
ATOM    532  HB1 PRO    40       20.154  10.884  25.966  1.00
67.93          H
ATOM    533  HB2 PRO    40       21.183  11.499  24.655  1.00
70.99          H
ATOM    534  HG1 PRO    40       21.379   8.979  25.460  1.00
65.91          H
ATOM    535  HG2 PRO    40       21.458   9.378  23.733  1.00
54.99          H
ATOM    536  HD1 PRO    40       19.221   8.133  25.306  1.00
24.22          H
ATOM    537  HD2 PRO    40       19.693   7.866  23.612  1.00
26.94          H
```

```
ATOM    538  N   GLY    41       19.625  12.873  22.594
1.00105.24           N
ATOM    539  CA  GLY    41       19.981  13.547  21.346
1.00147.55           C
ATOM    540  C   GLY    41       21.480  13.822  21.291
1.00161.74           C
ATOM    541  O   GLY    41       22.259  12.973  20.858
1.00244.37           O
ATOM    542  HN  GLY    41       19.453  13.407  23.394
1.00121.79           H
ATOM    543  HA1 GLY    41       19.451  14.486  21.288
1.00184.40           H
ATOM    544  HA2 GLY    41       19.697  12.928  20.506
1.00157.34           H
ATOM    545  N   LEU    42       21.878  15.012  21.733
1.00162.48           N
ATOM    546  CA  LEU    42       23.289  15.387  21.730
1.00191.48           C
ATOM    547  C   LEU    42       23.993  14.802  22.952
1.00166.66           C
ATOM    548  O   LEU    42       23.468  14.847  24.064
1.00251.38           O
ATOM    549  CB  LEU    42       23.427  16.911  21.746
1.00307.99           C
ATOM    550  CG  LEU    42       22.686  17.519  20.547
1.00452.28           C
ATOM    551  CD1 LEU    42       22.771  19.046  20.630
1.00681.24           C
ATOM    552  CD2 LEU    42       23.322  17.037  19.231
1.00527.46           C
ATOM    553  HN  LEU    42       21.212  15.647  22.068
1.00195.47           H
ATOM    554  HA  LEU    42       23.760  14.999  20.840
1.00229.77           H
ATOM    555  HB1 LEU    42       24.472  17.176  21.690
1.00342.49           H
ATOM    556  HB2 LEU    42       23.006  17.298  22.662
1.00335.33           H
ATOM    557  HG  LEU    42       21.648  17.218  20.581
1.00427.79           H
ATOM    558 HD11 LEU    42       23.803  19.355  20.535
1.00909.71           H
ATOM    559 HD12 LEU    42       22.381  19.379  21.580
1.00706.80           H
ATOM    560 HD13 LEU    42       22.191  19.483  19.830
1.00858.82           H
ATOM    561 HD21 LEU    42       22.919  16.068  18.972
1.00631.69           H
ATOM    562 HD22 LEU    42       24.393  16.962  19.349
1.00628.06           H
ATOM    563 HD23 LEU    42       23.097  17.739  18.440
1.00656.75           H
```

```
ATOM    564  N   THR    43       25.184  14.253  22.736
1.00178.52          N
ATOM    565  CA  THR    43       25.957  13.657  23.825
1.00240.84          C
ATOM    566  C   THR    43       26.699  14.754  24.606
1.00372.31          C
ATOM    567  O   THR    43       26.882  15.854  24.083
1.00500.13          O
ATOM    568  CB  THR    43       26.958  12.648  23.245
1.00354.13          C
ATOM    569  CG2 THR    43       26.215  11.589  22.422
1.00463.04          C
ATOM    570  OG1 THR    43       27.885  13.327  22.412
1.00508.50          O
ATOM    571  HN  THR    43       25.553  14.244  21.827
1.00229.59          H
ATOM    572  HA  THR    43       25.276  13.140  24.478
1.00249.90          H
ATOM    573  HB  THR    43       27.488  12.163  24.045
1.00481.58          H
ATOM    574  HG1 THR    43       28.642  12.751  22.279
1.00624.31          H
ATOM    575 HG21 THR    43       25.778  10.858  23.087
1.00650.95          H
ATOM    576 HG22 THR    43       26.909  11.100  21.756
1.00580.73          H
ATOM    577 HG23 THR    43       25.435  12.058  21.841
1.00571.99          H
ATOM    578  N   PRO    44       27.135  14.507  25.831
1.00488.39          N
ATOM    579  CA  PRO    44       27.865  15.543  26.630
1.00760.13          C
ATOM    580  C   PRO    44       29.124  16.025  25.914
1.00735.36          C
ATOM    581  O   PRO    44       29.628  17.115  26.185
1.00999.99          O
ATOM    582  CB  PRO    44       28.217  14.823  27.947
1.00999.99          C
ATOM    583  CG  PRO    44       27.256  13.687  28.037
1.00856.94          C
ATOM    584  CD  PRO    44       26.999  13.249  26.599
1.00548.09          C
ATOM    585  HA  PRO    44       27.215  16.377  26.834
1.00939.00          H
ATOM    586  HB1 PRO    44       28.081  15.486  28.786
1.00999.99          H
ATOM    587  HB2 PRO    44       29.235  14.455  27.921
1.00999.99          H
ATOM    588  HG1 PRO    44       26.331  14.011  28.491
1.00999.99          H
ATOM    589  HG2 PRO    44       27.689  12.874  28.608
1.00853.53          H
```

```
ATOM    590  HD1 PRO    44       26.008  12.851  26.512
1.00602.45           H
ATOM    591  HD2 PRO    44       27.741  12.526  26.293
1.00463.32           H
ATOM    592  N   ILE    45       29.633  15.192  25.010
1.00518.33           N
ATOM    593  CA  ILE    45       30.842  15.519  24.261
1.00582.79           C
ATOM    594  C   ILE    45       30.883  17.003  23.894
1.00795.93           C
ATOM    595  O   ILE    45       29.849  17.611  23.617
1.00898.75           O
ATOM    596  CB  ILE    45       30.906  14.666  22.987
1.00474.41           C
ATOM    597  CG1 ILE    45       32.308  14.760  22.371
1.00516.90           C
ATOM    598  CG2 ILE    45       29.867  15.166  21.972
1.00760.75           C
ATOM    599  CD1 ILE    45       32.438  13.734  21.244
1.00566.81           C
ATOM    600  HN  ILE    45       29.188  14.334  24.848
1.00395.72           H
ATOM    601  HA  ILE    45       31.696  15.287  24.875
1.00711.06           H
ATOM    602  HB  ILE    45       30.692  13.637  23.236
1.00477.81           H
ATOM    603 HG11 ILE    45       33.050  14.556  23.128
1.00624.28           H
ATOM    604 HG12 ILE    45       32.460  15.754  21.974
1.00744.84           H
ATOM    605 HG21 ILE    45       30.243  16.046  21.470
1.00920.57           H
ATOM    606 HG22 ILE    45       28.948  15.411  22.485
1.00999.99           H
ATOM    607 HG23 ILE    45       29.674  14.391  21.243
1.00865.46           H
ATOM    608 HD11 ILE    45       32.243  12.746  21.634
1.00658.89           H
ATOM    609 HD12 ILE    45       33.438  13.770  20.838
1.00734.30           H
ATOM    610 HD13 ILE    45       31.726  13.961  20.465
1.00675.84           H
ATOM    611  N   ALA    46       32.081  17.576  23.894
1.00999.99           N
ATOM    612  CA  ALA    46       32.241  18.987  23.558
1.00999.99           C
ATOM    613  C   ALA    46       33.690  19.290  23.189
1.00999.99           C
ATOM    614  CB  ALA    46       31.822  19.856  24.745
1.00999.99           C
ATOM    615  OT1 ALA    46       34.111  18.862  22.128
1.00999.99           O
```

```
ATOM    616  OT2 ALA    46       34.355  19.946  23.973
1.00999.99           O
ATOM    617  HN  ALA    46       32.871  17.043  24.122
1.00999.99           H
ATOM    618  HA  ALA    46       31.608  19.222  22.715
1.00999.99           H
ATOM    619  HB1 ALA    46       31.970  20.897  24.499
1.00999.99           H
ATOM    620  HB2 ALA    46       32.421  19.601  25.608
1.00999.99           H
ATOM    621  HB3 ALA    46       30.779  19.684  24.967
1.00999.99           H
ENDMDL
TER
MODEL       14
ATOM      1  N   GLY     1       28.026   1.545  12.408
1.00999.99           N
ATOM      2  CA  GLY     1       27.030   0.736  13.167
1.00999.99           C
ATOM      3  C   GLY     1       25.707   1.489  13.236
1.00999.99           C
ATOM      4  O   GLY     1       25.623   2.563  13.831
1.00999.99           O
ATOM      5  HA1 GLY     1       27.393   0.563  14.168
1.00999.99           H
ATOM      6  HA2 GLY     1       26.882  -0.211  12.668
1.00999.99           H
ATOM      7  HT1 GLY     1       28.282   2.388  12.961
1.00999.99           H
ATOM      8  HT2 GLY     1       27.614   1.838  11.499
1.00999.99           H
ATOM      9  HT3 GLY     1       28.877   0.975  12.237
1.00999.99           H
ATOM     10  N   LEU     2       24.671   0.917  12.622
1.00999.99           N
ATOM     11  CA  LEU     2       23.340   1.532  12.609
1.00895.59           C
ATOM     12  C   LEU     2       22.318   0.588  13.235
1.00532.27           C
ATOM     13  O   LEU     2       22.456  -0.632  13.152
1.00625.21           O
ATOM     14  CB  LEU     2       22.924   1.845  11.170
1.00999.99           C
ATOM     15  CG  LEU     2       23.982   2.728  10.498
1.00999.99           C
ATOM     16  CD1 LEU     2       23.572   2.983   9.044
1.00999.99           C
ATOM     17  CD2 LEU     2       24.103   4.069  11.243
1.00999.99           C
ATOM     18  HN  LEU     2       24.803   0.060  12.165
1.00999.99           H
ATOM     19  HA  LEU     2       23.356   2.451  13.178
```

```
1.00890.87           H
ATOM     20  HB1 LEU     2      21.977   2.365  11.176
1.00924.07           H
ATOM     21  HB2 LEU     2      22.822   0.921  10.617
1.00999.99           H
ATOM     22  HG  LEU     2      24.935   2.218  10.514
1.00999.99           H
ATOM     23 HD11 LEU     2      23.345   2.042   8.564
1.00999.99           H
ATOM     24 HD12 LEU     2      24.384   3.467   8.521
1.00999.99           H
ATOM     25 HD13 LEU     2      22.700   3.619   9.022
1.00999.99           H
ATOM     26 HD21 LEU     2      24.518   4.817  10.582
1.00999.99           H
ATOM     27 HD22 LEU     2      24.754   3.948  12.096
1.00999.99           H
ATOM     28 HD23 LEU     2      23.126   4.388  11.578
1.00999.99           H
ATOM     29  N   CYS     3      21.293   1.159  13.860
1.00271.28           N
ATOM     30  CA  CYS     3      20.255   0.351  14.492
1.00104.50           C
ATOM     31  C   CYS     3      19.477  -0.430  13.438  1.00
90.98           C
ATOM     32  O   CYS     3      19.280   0.048  12.321
1.00207.28           O
ATOM     33  CB  CYS     3      19.293   1.247  15.277  1.00
37.83           C
ATOM     34  SG  CYS     3      20.176   2.037  16.648  1.00
81.56           S
ATOM     35  HN  CYS     3      21.232   2.138  13.895
1.00296.55           H
ATOM     36  HA  CYS     3      20.720  -0.345  15.175
1.00166.45           H
ATOM     37  HB1 CYS     3      18.483   0.650  15.667  1.00
40.38           H
ATOM     38  HB2 CYS     3      18.895   2.009  14.621  1.00
98.45           H
ATOM     39  N   SER     4      19.038  -1.630  13.800  1.00
85.31           N
ATOM     40  CA  SER     4      18.281  -2.465  12.875  1.00
92.94           C
ATOM     41  C   SER     4      17.009  -1.754  12.452  1.00
67.37           C
ATOM     42  O   SER     4      16.621  -1.778  11.283
1.00102.28           O
ATOM     43  CB  SER     4      17.888  -3.774  13.552
1.00123.83           C
ATOM     44  OG  SER     4      17.277  -4.632  12.596
1.00178.89           O
ATOM     45  HN  SER     4      19.224  -1.958  14.704
```

```
1.00163.03            H
ATOM     46  HA  SER     4       18.883  -2.681  12.006
1.00129.78            H
ATOM     47  HB1 SER     4       17.183  -3.558  14.351
1.00105.26            H
ATOM     48  HB2 SER     4       18.760  -4.254  13.961
1.00156.87            H
ATOM     49  HG  SER     4       17.824  -5.417  12.510
1.00218.16            H
ATOM     50  N   GLU     5       16.354  -1.142  13.429  1.00
41.67           N
ATOM     51  CA  GLU     5       15.102  -0.435  13.192  1.00
41.21           C
ATOM     52  C   GLU     5       14.984   0.767  14.119  1.00
41.92           C
ATOM     53  O   GLU     5       15.928   1.105  14.834  1.00
74.72           O
ATOM     54  CB  GLU     5       13.938  -1.392  13.430  1.00
49.24           C
ATOM     55  CG  GLU     5       14.006  -1.912  14.859  1.00
55.48           C
ATOM     56  CD  GLU     5       13.019  -3.056  15.050  1.00
92.06           C
ATOM     57  OE1 GLU     5       12.338  -3.390  14.094
1.00206.49            O
ATOM     58  OE2 GLU     5       12.956  -3.584  16.147
1.00197.24            O1-
ATOM     59  HN  GLU     5       16.713  -1.178  14.340  1.00
45.52           H
ATOM     60  HA  GLU     5       15.069  -0.100  12.175  1.00
60.63           H
ATOM     61  HB1 GLU     5       14.004  -2.222  12.742  1.00
61.59           H
ATOM     62  HB2 GLU     5       13.010  -0.873  13.279  1.00
63.66           H
ATOM     63  HG1 GLU     5       13.767  -1.112  15.544  1.00
58.03           H
ATOM     64  HG2 GLU     5       15.007  -2.260  15.053  1.00
57.77           H
ATOM     65  N   ASN     6       13.823   1.411  14.107  1.00
50.73           N
ATOM     66  CA  ASN     6       13.603   2.576  14.956  1.00
71.04           C
ATOM     67  C   ASN     6       13.774   2.198  16.422  1.00
65.17           C
ATOM     68  O   ASN     6       14.338   2.958  17.209  1.00
94.86           O
ATOM     69  CB  ASN     6       12.196   3.131  14.728  1.00
94.83           C
ATOM     70  CG  ASN     6       12.114   3.798  13.359
1.00160.94            C
ATOM     71  ND2 ASN     6       10.947   3.980  12.803
```

```
1.00244.88           N
ATOM     72  OD1 ASN     6      13.137   4.163  12.782
1.00219.73           O
ATOM     73  HN  ASN     6      13.104   1.100  13.519  1.00
72.81           H
ATOM     74  HA  ASN     6      14.326   3.336  14.702  1.00
96.02           H
ATOM     75  HB1 ASN     6      11.971   3.859  15.494
1.00111.09           H
ATOM     76  HB2 ASN     6      11.480   2.325  14.778  1.00
98.13           H
ATOM     77 HD21 ASN     6      10.134   3.688  13.264
1.00272.43           H
ATOM     78 HD22 ASN     6      10.885   4.409  11.923
1.00336.78           H
ATOM     79  N   GLY     7      13.287   1.009  16.783  1.00
51.80           N
ATOM     80  CA  GLY     7      13.391   0.518  18.159  1.00
71.47           C
ATOM     81  C   GLY     7      14.059  -0.850  18.192  1.00
41.04           C
ATOM     82  O   GLY     7      13.406  -1.871  18.400  1.00
44.54           O
ATOM     83  HN  GLY     7      12.854   0.448  16.108  1.00
45.49           H
ATOM     84  HA1 GLY     7      12.404   0.432  18.579
1.00103.52           H
ATOM     85  HA2 GLY     7      13.972   1.210  18.755
1.00108.67           H
ATOM     86  N   ASP     8      15.369  -0.858  17.981  1.00
27.00           N
ATOM     87  CA  ASP     8      16.129  -2.102  17.983  1.00
14.15           C
ATOM     88  C   ASP     8      16.032  -2.787  19.340  1.00
9.37           C
ATOM     89  O   ASP     8      15.836  -4.000  19.423  1.00
14.61           O
ATOM     90  CB  ASP     8      17.596  -1.821  17.654  1.00
15.06           C
ATOM     91  CG  ASP     8      18.373  -3.130  17.578  1.00
21.22           C
ATOM     92  OD1 ASP     8      19.528  -3.089  17.185
1.00119.56           O
ATOM     93  OD2 ASP     8      17.803  -4.156  17.912
1.00133.27           O1-
ATOM     94  HN  ASP     8      15.831  -0.011  17.818  1.00
35.63           H
ATOM     95  HA  ASP     8      15.724  -2.759  17.232  1.00
20.92           H
ATOM     96  HB1 ASP     8      18.023  -1.197  18.426  1.00
29.39           H
ATOM     97  HB2 ASP     8      17.659  -1.310  16.705  1.00
```

```
44.14           H
ATOM     98  N   CYS     9      16.169  -2.001  20.397  1.00
5.93           N
ATOM     99  CA  CYS     9      16.095  -2.537  21.751  1.00
7.54           C
ATOM    100  C   CYS     9      14.717  -3.131  22.012  1.00
14.60           C
ATOM    101  O   CYS     9      14.589  -4.199  22.611  1.00
25.78           O
ATOM    102  CB  CYS     9      16.383  -1.432  22.757  1.00
6.63           C
ATOM    103  SG  CYS     9      18.127  -1.008  22.639  1.00
10.91           S
ATOM    104  HN  CYS     9      16.321  -1.044  20.264  1.00
6.51           H
ATOM    105  HA  CYS     9      16.844  -3.301  21.863  1.00
11.12           H
ATOM    106  HB1 CYS     9      16.165  -1.774  23.755  1.00
11.28           H
ATOM    107  HB2 CYS     9      15.780  -0.564  22.528  1.00
5.04           H
ATOM    108  N   ALA    10      13.694  -2.428  21.550  1.00
16.26           N
ATOM    109  CA  ALA    10      12.318  -2.880  21.719  1.00
32.04           C
ATOM    110  C   ALA    10      11.377  -2.024  20.874  1.00
45.47           C
ATOM    111  O   ALA    10      11.748  -0.942  20.420
1.00119.04           O
ATOM    112  CB  ALA    10      11.914  -2.806  23.194  1.00
30.10           C
ATOM    113  HN  ALA    10      13.867  -1.589  21.077  1.00
12.81           H
ATOM    114  HA  ALA    10      12.246  -3.906  21.388  1.00
47.14           H
ATOM    115  HB1 ALA    10      11.633  -1.794  23.443  1.00
84.37           H
ATOM    116  HB2 ALA    10      12.747  -3.110  23.810
1.00118.20           H
ATOM    117  HB3 ALA    10      11.077  -3.464  23.371
1.00117.32           H
ATOM    118  N   ALA    11      10.163  -2.516  20.663  1.00
30.62           N
ATOM    119  CA  ALA    11       9.182  -1.788  19.866  1.00
35.24           C
ATOM    120  C   ALA    11       8.864  -0.434  20.499  1.00
22.78           C
ATOM    121  O   ALA    11       8.702   0.564  19.797  1.00
53.33           O
ATOM    122  CB  ALA    11       7.897  -2.607  19.747  1.00
60.49           C
ATOM    123  HN  ALA    11       9.922  -3.386  21.047  1.00
```

```
50.37           H
ATOM    124  HA  ALA    11        9.585  -1.626  18.879  1.00
41.70           H
ATOM    125  HB1 ALA    11        7.349  -2.555  20.676
1.00157.06           H
ATOM    126  HB2 ALA    11        8.145  -3.637  19.534
1.00148.83           H
ATOM    127  HB3 ALA    11        7.289  -2.211  18.948
1.00137.06           H
ATOM    128  N   ASP    12        8.765  -0.411  21.825  1.00
18.98           N
ATOM    129  CA  ASP    12        8.452   0.823  22.549  1.00
31.05           C
ATOM    130  C   ASP    12        9.721   1.567  22.958  1.00
22.98           C
ATOM    131  O   ASP    12        9.662   2.522  23.734  1.00
37.14           O
ATOM    132  CB  ASP    12        7.628   0.493  23.796  1.00
48.35           C
ATOM    133  CG  ASP    12        8.428  -0.410  24.727
1.00104.83           C
ATOM    134  OD1 ASP    12        7.972  -0.637  25.837
1.00258.73           O
ATOM    135  OD2 ASP    12        9.481  -0.868  24.317
1.00213.46           O1-
ATOM    136  HN  ASP    12        8.897  -1.241  22.330  1.00
36.19           H
ATOM    137  HA  ASP    12        7.863   1.466  21.911  1.00
49.76           H
ATOM    138  HB1 ASP    12        6.720  -0.012  23.502  1.00
95.71           H
ATOM    139  HB2 ASP    12        7.378   1.408  24.311  1.00
54.37           H
ATOM    140  N   GLU    13       10.868   1.133  22.432  1.00
17.60           N
ATOM    141  CA  GLU    13       12.154   1.772  22.748  1.00
12.01           C
ATOM    142  C   GLU    13       12.795   2.353  21.492  1.00
10.07           C
ATOM    143  O   GLU    13       12.449   1.978  20.373  1.00
12.81           O
ATOM    144  CB  GLU    13       13.103   0.757  23.385  1.00
11.74           C
ATOM    145  CG  GLU    13       12.602   0.400  24.786  1.00
13.49           C
ATOM    146  CD  GLU    13       12.786   1.589  25.721
1.00142.36           C
ATOM    147  OE1 GLU    13       13.471   2.520  25.332
1.00339.06           O
ATOM    148  OE2 GLU    13       12.236   1.556  26.807
1.00335.28           O1-
ATOM    149  HN  GLU    13       10.853   0.370  21.817  1.00
```

28.36 H
ATOM 150 HA GLU 13 11.993 2.578 23.452 1.00
13.06 H
ATOM 151 HB1 GLU 13 14.092 1.183 23.456 1.00
13.76 H
ATOM 152 HB2 GLU 13 13.140 -0.134 22.774 1.00
9.94 H
ATOM 153 HG1 GLU 13 13.160 -0.442 25.165 1.00
66.02 H
ATOM 154 HG2 GLU 13 11.555 0.144 24.738 1.00
56.23 H
ATOM 155 N CYS 14 13.729 3.284 21.694 1.00
9.12 N
ATOM 156 CA CYS 14 14.426 3.940 20.584 1.00
10.69 C
ATOM 157 C CYS 14 15.856 3.424 20.460 1.00
9.56 C
ATOM 158 O CYS 14 16.431 2.922 21.425 1.00
13.62 O
ATOM 159 CB CYS 14 14.455 5.449 20.821 1.00
13.96 C
ATOM 160 SG CYS 14 15.427 6.251 19.520 1.00
42.96 S
ATOM 161 HN CYS 14 13.954 3.541 22.614 1.00
9.76 H
ATOM 162 HA CYS 14 13.901 3.745 19.658 1.00
15.54 H
ATOM 163 HB1 CYS 14 14.903 5.650 21.783 1.00
50.72 H
ATOM 164 HB2 CYS 14 13.446 5.836 20.808 1.00
46.13 H
ATOM 165 N CYS 15 16.425 3.559 19.265 1.00
9.60 N
ATOM 166 CA CYS 15 17.793 3.114 19.010 1.00
9.28 C
ATOM 167 C CYS 15 18.439 4.024 17.976 1.00
9.92 C
ATOM 168 O CYS 15 17.891 4.227 16.892 1.00
13.31 O
ATOM 169 CB CYS 15 17.789 1.673 18.493 1.00
12.90 C
ATOM 170 SG CYS 15 19.494 1.089 18.298 1.00
39.11 S
ATOM 171 HN CYS 15 15.918 3.974 18.535 1.00
13.12 H
ATOM 172 HA CYS 15 18.365 3.159 19.928 1.00
8.26 H
ATOM 173 HB1 CYS 15 17.287 1.636 17.537 1.00
24.82 H
ATOM 174 HB2 CYS 15 17.271 1.040 19.196 1.00
27.31 H
ATOM 175 N VAL 16 19.603 4.576 18.308 1.00

9.56 N
ATOM 176 CA VAL 16 20.299 5.465 17.386 1.00
11.99 C
ATOM 177 C VAL 16 21.801 5.388 17.622 1.00
7.73 C
ATOM 178 O VAL 16 22.264 5.450 18.760 1.00
8.83 O
ATOM 179 CB VAL 16 19.803 6.900 17.579 1.00
18.37 C
ATOM 180 CG1 VAL 16 20.101 7.364 19.005 1.00
39.61 C
ATOM 181 CG2 VAL 16 20.507 7.818 16.583
1.00115.21 C
ATOM 182 HN VAL 16 20.002 4.385 19.187 1.00
9.76 H
ATOM 183 HA VAL 16 20.093 5.158 16.370 1.00
17.27 H
ATOM 184 HB VAL 16 18.736 6.935 17.408 1.00
52.63 H
ATOM 185 HG11 VAL 16 19.599 8.302 19.191
1.00128.07 H
ATOM 186 HG12 VAL 16 21.166 7.497 19.126
1.00154.13 H
ATOM 187 HG13 VAL 16 19.749 6.622 19.705
1.00135.74 H
ATOM 188 HG21 VAL 16 20.382 7.427 15.584
1.00229.05 H
ATOM 189 HG22 VAL 16 21.561 7.869 16.819
1.00261.82 H
ATOM 190 HG23 VAL 16 20.077 8.806 16.639
1.00210.07 H
ATOM 191 N ASP 17 22.559 5.232 16.539 1.00
14.34 N
ATOM 192 CA ASP 17 24.014 5.126 16.639 1.00
11.90 C
ATOM 193 C ASP 17 24.693 6.439 16.276 1.00
13.15 C
ATOM 194 O ASP 17 24.585 6.919 15.148 1.00
25.96 O
ATOM 195 CB ASP 17 24.518 4.022 15.708 1.00
20.80 C
ATOM 196 CG ASP 17 24.106 2.657 16.249 1.00
28.72 C
ATOM 197 OD1 ASP 17 24.193 1.695 15.504
1.00135.42 O
ATOM 198 OD2 ASP 17 23.711 2.593 17.401
1.00114.17 O1-
ATOM 199 HN ASP 17 22.133 5.174 15.658 1.00
28.29 H
ATOM 200 HA ASP 17 24.284 4.866 17.651 1.00
10.49 H
ATOM 201 HB1 ASP 17 25.596 4.071 15.644 1.00

22.42 H
ATOM 202 HB2 ASP 17 24.093 4.162 14.724 1.00
29.52 H
ATOM 203 N THR 18 25.417 6.999 17.242 1.00
11.55 N
ATOM 204 CA THR 18 26.151 8.245 17.031 1.00
17.10 C
ATOM 205 C THR 18 27.619 7.925 16.791 1.00
10.63 C
ATOM 206 O THR 18 28.081 6.837 17.128 1.00
6.49 O
ATOM 207 CB THR 18 26.016 9.154 18.263 1.00
28.52 C
ATOM 208 CG2 THR 18 24.688 9.908 18.219 1.00
45.38 C
ATOM 209 OG1 THR 18 26.072 8.360 19.436 1.00
26.49 O
ATOM 210 HN THR 18 25.475 6.553 18.111 1.00
13.96 H
ATOM 211 HA THR 18 25.757 8.760 16.164 1.00
26.43 H
ATOM 212 HB THR 18 26.826 9.870 18.278 1.00
37.43 H
ATOM 213 HG1 THR 18 25.173 8.211 19.737 1.00
71.62 H
ATOM 214 HG21 THR 18 24.761 10.720 17.511
1.00100.44 H
ATOM 215 HG22 THR 18 24.468 10.304 19.200
1.00129.42 H
ATOM 216 HG23 THR 18 23.900 9.235 17.916
1.00156.21 H
ATOM 217 N VAL 19 28.343 8.882 16.226 1.00
16.32 N
ATOM 218 CA VAL 19 29.748 8.710 15.954 1.00
13.34 C
ATOM 219 C VAL 19 30.282 9.951 15.276 1.00
25.88 C
ATOM 220 O VAL 19 29.859 10.326 14.184 1.00
42.11 O
ATOM 221 CB VAL 19 30.016 7.493 15.071 1.00
15.48 C
ATOM 222 CG1 VAL 19 29.145 7.552 13.811 1.00
29.06 C
ATOM 223 CG2 VAL 19 31.500 7.485 14.670 1.00
20.67 C
ATOM 224 HN VAL 19 27.930 9.735 16.001 1.00
26.89 H
ATOM 225 HA VAL 19 30.267 8.577 16.893 1.00
8.08 H
ATOM 226 HB VAL 19 29.791 6.599 15.623 1.00
11.49 H
ATOM 227 HG11 VAL 19 29.562 8.263 13.113

```
1.00137.43             H
ATOM    228 HG12 VAL    19       28.143   7.852  14.076  1.00
93.29          H
ATOM    229 HG13 VAL    19       29.116   6.574  13.350
1.00108.69             H
ATOM    230 HG21 VAL    19       32.107   7.725  15.534  1.00
99.42          H
ATOM    231 HG22 VAL    19       31.668   8.228  13.903
1.00111.36             H
ATOM    232 HG23 VAL    19       31.769   6.511  14.296  1.00
77.11          H
ATOM    233  N   PHE    20       31.215  10.571  15.949  1.00
24.58          N
ATOM    234  CA  PHE    20       31.848  11.778  15.453  1.00
40.34          C
ATOM    235  C   PHE    20       33.073  11.425  14.622  1.00
40.37          C
ATOM    236  O   PHE    20       33.135  11.733  13.432  1.00
66.02          O
ATOM    237  CB  PHE    20       32.239  12.690  16.632  1.00
48.79          C
ATOM    238  CG  PHE    20       32.454  11.886  17.909  1.00
35.75          C
ATOM    239  CD1 PHE    20       31.384  11.200  18.519  1.00
30.20          C
ATOM    240  CD2 PHE    20       33.726  11.851  18.501  1.00
40.95          C
ATOM    241  CE1 PHE    20       31.597  10.484  19.700  1.00
30.41          C
ATOM    242  CE2 PHE    20       33.931  11.131  19.682  1.00
45.84          C
ATOM    243  CZ  PHE    20       32.869  10.448  20.281  1.00
40.81          C
ATOM    244  HN  PHE    20       31.483  10.202  16.806  1.00
16.73          H
ATOM    245  HA  PHE    20       31.149  12.309  14.818  1.00
59.91          H
ATOM    246  HB1 PHE    20       31.453  13.391  16.796  1.00
67.15          H
ATOM    247  HB2 PHE    20       33.142  13.234  16.393  1.00
58.17          H
ATOM    248  HD1 PHE    20       30.396  11.211  18.075  1.00
33.34          H
ATOM    249  HD2 PHE    20       34.547  12.381  18.045  1.00
49.35          H
ATOM    250  HE1 PHE    20       30.776   9.962  20.163  1.00
30.61          H
ATOM    251  HE2 PHE    20       34.913  11.102  20.133  1.00
61.27          H
ATOM    252  HZ  PHE    20       33.028   9.894  21.195  1.00
52.66          H
ATOM    253  N   GLU    21       34.057  10.796  15.259  1.00
```

```
41.31           N
ATOM    254  CA  GLU    21       35.281  10.427  14.594  1.00
56.16           C
ATOM    255  C   GLU    21       35.462   8.916  14.598  1.00
58.61           C
ATOM    256  O   GLU    21       34.568   8.172  15.000
1.00201.19           O
ATOM    257  CB  GLU    21       36.409  11.089  15.351  1.00
57.37           C
ATOM    258  CG  GLU    21       36.485  10.503  16.759
1.00193.56           C
ATOM    259  CD  GLU    21       37.379  11.372  17.634
1.00304.62           C
ATOM    260  OE1 GLU    21       37.504  11.066  18.809
1.00451.81           O
ATOM    261  OE2 GLU    21       37.929  12.332  17.119
1.00442.88           O1-
ATOM    262  HN  GLU    21       33.973  10.594  16.211  1.00
50.82           H
ATOM    263  HA  GLU    21       35.283  10.787  13.574  1.00
85.72           H
ATOM    264  HB1 GLU    21       36.211  12.145  15.417  1.00
46.37           H
ATOM    265  HB2 GLU    21       37.329  10.920  14.837
1.00131.81           H
ATOM    266  HG1 GLU    21       36.890   9.507  16.703
1.00314.91           H
ATOM    267  HG2 GLU    21       35.495  10.459  17.185
1.00266.92           H
ATOM    268  N   GLY    22       36.625   8.474  14.142  1.00
86.70           N
ATOM    269  CA  GLY    22       36.925   7.044  14.088
1.00104.66           C
ATOM    270  C   GLY    22       37.556   6.559  15.390  1.00
71.50           C
ATOM    271  O   GLY    22       37.553   5.363  15.684  1.00
89.87           O
ATOM    272  HN  GLY    22       37.294   9.124  13.838
1.00214.64           H
ATOM    273  HA1 GLY    22       37.612   6.860  13.276
1.00152.47           H
ATOM    274  HA2 GLY    22       36.012   6.491  13.909
1.00118.15           H
ATOM    275  N   ASP    23       38.107   7.491  16.159  1.00
44.68           N
ATOM    276  CA  ASP    23       38.753   7.144  17.421  1.00
43.33           C
ATOM    277  C   ASP    23       37.754   6.577  18.424  1.00
37.55           C
ATOM    278  O   ASP    23       38.068   5.641  19.161  1.00
61.37           O
ATOM    279  CB  ASP    23       39.417   8.383  18.022  1.00
```

```
40.74           C
ATOM    280  CG  ASP    23      40.637   8.775  17.193
1.00141.86           C
ATOM    281  OD1 ASP    23      41.063   7.969  16.384
1.00328.18           O
ATOM    282  OD2 ASP    23      41.127   9.877  17.382
1.00304.58           O1-
ATOM    283  HN  ASP    23      38.087   8.427  15.870  1.00
40.86           H
ATOM    284  HA  ASP    23      39.511   6.405  17.232  1.00
72.12           H
ATOM    285  HB1 ASP    23      39.727   8.170  19.033  1.00
94.15           H
ATOM    286  HB2 ASP    23      38.711   9.198  18.031  1.00
93.26           H
ATOM    287  N   MET    24      36.562   7.163  18.468  1.00
23.24           N
ATOM    288  CA  MET    24      35.527   6.724  19.411  1.00
32.50           C
ATOM    289  C   MET    24      34.157   6.655  18.748  1.00
25.13           C
ATOM    290  O   MET    24      33.876   7.377  17.791  1.00
54.40           O
ATOM    291  CB  MET    24      35.457   7.700  20.583  1.00
47.36           C
ATOM    292  CG  MET    24      36.729   7.596  21.424
1.00151.73           C
ATOM    293  SD  MET    24      36.748   8.928  22.648
1.00209.23           S
ATOM    294  CE  MET    24      35.313   8.380  23.606
1.00243.29           C
ATOM    295  HN  MET    24      36.379   7.915  17.869  1.00
17.32           H
ATOM    296  HA  MET    24      35.777   5.743  19.793  1.00
48.94           H
ATOM    297  HB1 MET    24      34.601   7.462  21.199
1.00124.40           H
ATOM    298  HB2 MET    24      35.357   8.708  20.206
1.00166.96           H
ATOM    299  HG1 MET    24      37.591   7.686  20.782
1.00331.71           H
ATOM    300  HG2 MET    24      36.751   6.640  21.926
1.00302.30           H
ATOM    301  HE1 MET    24      34.409   8.756  23.144
1.00340.44           H
ATOM    302  HE2 MET    24      35.383   8.759  24.612
1.00373.88           H
ATOM    303  HE3 MET    24      35.288   7.300  23.631
1.00386.81           H
ATOM    304  N   VAL    25      33.297   5.790  19.289  1.00
22.44           N
ATOM    305  CA  VAL    25      31.934   5.627  18.782  1.00
```

15.37 C
ATOM 306 C VAL 25 30.939 5.664 19.935 1.00
17.47 C
ATOM 307 O VAL 25 31.212 5.155 21.023 1.00
29.52 O
ATOM 308 CB VAL 25 31.784 4.310 18.020 1.00
23.36 C
ATOM 309 CG1 VAL 25 30.313 4.132 17.607 1.00
58.39 C
ATOM 310 CG2 VAL 25 32.670 4.343 16.772 1.00
55.25 C
ATOM 311 HN VAL 25 33.583 5.258 20.060 1.00
48.11 H
ATOM 312 HA VAL 25 31.704 6.442 18.107 1.00
9.25 H
ATOM 313 HB VAL 25 32.080 3.488 18.659 1.00
52.45 H
ATOM 314 HG11 VAL 25 29.758 3.712 18.431
1.00171.07 H
ATOM 315 HG12 VAL 25 30.247 3.470 16.757
1.00166.22 H
ATOM 316 HG13 VAL 25 29.889 5.094 17.348
1.00134.76 H
ATOM 317 HG21 VAL 25 32.710 3.355 16.334
1.00141.88 H
ATOM 318 HG22 VAL 25 33.667 4.654 17.046
1.00184.50 H
ATOM 319 HG23 VAL 25 32.258 5.038 16.056
1.00133.90 H
ATOM 320 N THR 26 29.789 6.284 19.691 1.00
13.60 N
ATOM 321 CA THR 26 28.745 6.413 20.709 1.00
21.19 C
ATOM 322 C THR 26 27.420 5.831 20.220 1.00
16.55 C
ATOM 323 O THR 26 26.994 6.088 19.094 1.00
11.43 O
ATOM 324 CB THR 26 28.567 7.895 21.036 1.00
27.07 C
ATOM 325 CG2 THR 26 27.456 8.081 22.073 1.00
44.39 C
ATOM 326 OG1 THR 26 29.786 8.400 21.560 1.00
34.75 O
ATOM 327 HN THR 26 29.640 6.678 18.805 1.00
10.81 H
ATOM 328 HA THR 26 29.044 5.893 21.608 1.00
33.16 H
ATOM 329 HB THR 26 28.314 8.428 20.130 1.00
20.60 H
ATOM 330 HG1 THR 26 30.201 7.701 22.071 1.00
77.72 H
ATOM 331 HG21 THR 26 27.670 7.472 22.941

```
1.00126.99          H
ATOM    332 HG22 THR    26      26.508   7.785  21.653
1.00108.32          H
ATOM    333 HG23 THR    26      27.411   9.118  22.365
1.00151.99          H
ATOM    334  N   ARG    27      26.769   5.051  21.085  1.00
22.38         N
ATOM    335  CA  ARG    27      25.482   4.431  20.758  1.00
20.21         C
ATOM    336  C   ARG    27      24.468   4.726  21.860  1.00
16.96         C
ATOM    337  O   ARG    27      24.836   4.838  23.030  1.00
20.06         O
ATOM    338  CB  ARG    27      25.654   2.917  20.616  1.00
22.14         C
ATOM    339  CG  ARG    27      26.624   2.614  19.472
1.00124.30          C
ATOM    340  CD  ARG    27      26.681   1.104  19.238
1.00109.38          C
ATOM    341  NE  ARG    27      27.597   0.797  18.144
1.00227.73          N
ATOM    342  CZ  ARG    27      28.908   0.707  18.346
1.00426.12          C
ATOM    343  NH1 ARG    27      29.704   0.433  17.348
1.00767.09          N1+
ATOM    344  NH2 ARG    27      29.398   0.892  19.541
1.00581.78          N
ATOM    345  HN  ARG    27      27.161   4.893  21.970  1.00
30.75         H
ATOM    346  HA  ARG    27      25.110   4.830  19.825  1.00
23.13         H
ATOM    347  HB1 ARG    27      24.697   2.463  20.405  1.00
87.46         H
ATOM    348  HB2 ARG    27      26.047   2.512  21.538
1.00103.36          H
ATOM    349  HG1 ARG    27      27.609   2.973  19.731
1.00281.97          H
ATOM    350  HG2 ARG    27      26.284   3.105  18.573
1.00276.19          H
ATOM    351  HD1 ARG    27      25.693   0.743  18.991
1.00183.60          H
ATOM    352  HD2 ARG    27      27.024   0.614  20.138
1.00142.93          H
ATOM    353  HE  ARG    27      27.238   0.657  17.243
1.00372.53          H
ATOM    354 HH11 ARG    27      29.329   0.291  16.434
1.00910.59          H
ATOM    355 HH12 ARG    27      30.690   0.364  17.501
1.00999.99          H
ATOM    356 HH21 ARG    27      28.787   1.102  20.304
1.00532.54          H
ATOM    357 HH22 ARG    27      30.384   0.823  19.693
```

1.00948.84 H
ATOM 358 N SER 28 23.189 4.855 21.491 1.00
14.60 N
ATOM 359 CA SER 28 22.140 5.142 22.475 1.00
14.92 C
ATOM 360 C SER 28 20.936 4.233 22.260 1.00
12.12 C
ATOM 361 O SER 28 20.605 3.877 21.129 1.00
13.41 O
ATOM 362 CB SER 28 21.708 6.603 22.362 1.00
23.26 C
ATOM 363 OG SER 28 22.810 7.445 22.674
1.00146.32 O
ATOM 364 HN SER 28 22.943 4.757 20.544 1.00
15.23 H
ATOM 365 HA SER 28 22.524 4.972 23.472 1.00
15.44 H
ATOM 366 HB1 SER 28 20.892 6.791 23.047 1.00
88.73 H
ATOM 367 HB2 SER 28 21.383 6.808 21.356
1.00124.68 H
ATOM 368 HG SER 28 22.497 8.141 23.258
1.00242.47 H
ATOM 369 N CYS 29 20.285 3.865 23.358 1.00
10.48 N
ATOM 370 CA CYS 29 19.114 3.002 23.294 1.00
9.97 C
ATOM 371 C CYS 29 18.484 2.883 24.681 1.00
11.60 C
ATOM 372 O CYS 29 19.101 2.355 25.606 1.00
15.64 O
ATOM 373 CB CYS 29 19.510 1.612 22.770 1.00
9.52 C
ATOM 374 SG CYS 29 18.116 0.867 21.912 1.00
10.40 S
ATOM 375 HN CYS 29 20.595 4.188 24.230 1.00
11.10 H
ATOM 376 HA CYS 29 18.394 3.439 22.619 1.00
11.08 H
ATOM 377 HB1 CYS 29 19.793 0.968 23.594 1.00
9.33 H
ATOM 378 HB2 CYS 29 20.339 1.703 22.087 1.00
10.78 H
ATOM 379 N GLU 30 17.262 3.386 24.825 1.00
13.52 N
ATOM 380 CA GLU 30 16.574 3.338 26.111 1.00
17.41 C
ATOM 381 C GLU 30 15.860 2.003 26.300 1.00
10.81 C
ATOM 382 O GLU 30 15.509 1.330 25.331 1.00
23.22 O
ATOM 383 CB GLU 30 15.565 4.482 26.200 1.00

```
37.83           C
ATOM    384  CG  GLU    30       16.303   5.819  26.105
1.00108.13           C
ATOM    385  CD  GLU    30       17.167   6.027  27.344
1.00240.52           C
ATOM    386  OE1 GLU    30       18.070   6.846  27.281
1.00422.56           O
ATOM    387  OE2 GLU    30       16.914   5.367  28.338
1.00410.81           O1-
ATOM    388  HN  GLU    30       16.817   3.803  24.055  1.00
15.61           H
ATOM    389  HA  GLU    30       17.299   3.457  26.903  1.00
24.60           H
ATOM    390  HB1 GLU    30       15.042   4.428  27.142  1.00
72.67           H
ATOM    391  HB2 GLU    30       14.857   4.404  25.389  1.00
56.47           H
ATOM    392  HG1 GLU    30       15.584   6.620  26.032
1.00196.66           H
ATOM    393  HG2 GLU    30       16.931   5.818  25.226
1.00140.67           H
ATOM    394  N   LYS    31       15.650   1.628  27.563  1.00
11.96           N
ATOM    395  CA  LYS    31       14.972   0.371  27.897  1.00
11.40           C
ATOM    396  C   LYS    31       13.739   0.659  28.747  1.00
10.99           C
ATOM    397  O   LYS    31       13.745   1.569  29.575  1.00
12.86           O
ATOM    398  CB  LYS    31       15.923  -0.542  28.672  1.00
21.40           C
ATOM    399  CG  LYS    31       17.125  -0.895  27.793  1.00
57.81           C
ATOM    400  CD  LYS    31       18.074  -1.812  28.567
1.00115.52           C
ATOM    401  CE  LYS    31       19.302  -2.118  27.708
1.00250.23           C
ATOM    402  NZ  LYS    31       18.881  -2.853  26.482
1.00462.10           N1+
ATOM    403  HN  LYS    31       15.955   2.212  28.289  1.00
26.13           H
ATOM    404  HA  LYS    31       14.665  -0.131  26.990  1.00
13.87           H
ATOM    405  HB1 LYS    31       15.406  -1.448  28.951  1.00
37.63           H
ATOM    406  HB2 LYS    31       16.264  -0.034  29.562  1.00
47.19           H
ATOM    407  HG1 LYS    31       17.646   0.009  27.516
1.00126.32           H
ATOM    408  HG2 LYS    31       16.783  -1.400  26.902
1.00114.19           H
ATOM    409  HD1 LYS    31       17.568  -2.734  28.809
```

```
1.00198.45          H
ATOM    410  HD2 LYS    31      18.385  -1.321  29.479
1.00200.14          H
ATOM    411  HE1 LYS    31      19.994  -2.726  28.272
1.00374.64          H
ATOM    412  HE2 LYS    31      19.784  -1.193  27.426
1.00403.27          H
ATOM    413  HZ1 LYS    31      18.562  -2.175  25.762
1.00627.38          H
ATOM    414  HZ2 LYS    31      18.102  -3.502  26.718
1.00622.26          H
ATOM    415  HZ3 LYS    31      19.684  -3.398  26.110
1.00619.20          H
ATOM    416  N   THR    32      12.678  -0.116  28.531  1.00
15.51           N
ATOM    417  CA  THR    32      11.437   0.074  29.283  1.00
22.44           C
ATOM    418  C   THR    32      11.448  -0.754  30.564  1.00
26.60           C
ATOM    419  O   THR    32      11.538  -1.982  30.522  1.00
57.43           O
ATOM    420  CB  THR    32      10.238  -0.335  28.422  1.00
56.28           C
ATOM    421  CG2 THR    32       8.946  -0.130  29.213
1.00102.70          C
ATOM    422  OG1 THR    32      10.202   0.462  27.247
1.00111.17          O
ATOM    423  HN  THR    32      12.727  -0.822  27.855  1.00
18.79           H
ATOM    424  HA  THR    32      11.334   1.118  29.542  1.00
19.67           H
ATOM    425  HB  THR    32      10.326  -1.374  28.150  1.00
84.25           H
ATOM    426  HG1 THR    32      10.534  -0.071  26.518
1.00205.03          H
ATOM    427 HG21 THR    32       8.933   0.866  29.630
1.00220.71          H
ATOM    428 HG22 THR    32       8.893  -0.857  30.011
1.00174.67          H
ATOM    429 HG23 THR    32       8.099  -0.255  28.556
1.00217.44          H
ATOM    430  N   THR    33      11.346  -0.070  31.701  1.00
22.47           N
ATOM    431  CA  THR    33      11.335  -0.741  32.997  1.00
41.60           C
ATOM    432  C   THR    33      10.571   0.072  34.003  1.00
38.03           C
ATOM    433  O   THR    33      11.143   0.808  34.807  1.00
52.73           O
ATOM    434  CB  THR    33      12.754  -0.980  33.501  1.00
63.25           C
ATOM    435  CG2 THR    33      13.442  -2.038  32.637
```

```
1.00121.65           C
ATOM    436  OG1 THR    33       13.487   0.235  33.443  1.00
96.20           O
ATOM    437  HN  THR    33       11.272   0.906  31.665  1.00
23.82           H
ATOM    438  HA  THR    33       10.829  -1.684  32.908  1.00
67.12           H
ATOM    439  HB  THR    33       12.708  -1.328  34.520
1.00107.59           H
ATOM    440  HG1 THR    33       13.682   0.508  34.344
1.00176.27           H
ATOM    441 HG21 THR    33       12.795  -2.897  32.537
1.00255.23           H
ATOM    442 HG22 THR    33       14.366  -2.338  33.105
1.00245.37           H
ATOM    443 HG23 THR    33       13.647  -1.627  31.661
1.00185.78           H
ATOM    444  N   GLY    34        9.265  -0.091  33.957  1.00
40.93           N
ATOM    445  CA  GLY    34        8.397   0.604  34.876  1.00
45.59           C
ATOM    446  C   GLY    34        8.194   2.050  34.438  1.00
40.97           C
ATOM    447  O   GLY    34        7.398   2.782  35.025  1.00
82.17           O
ATOM    448  HN  GLY    34        8.883  -0.708  33.303  1.00
54.00           H
ATOM    449  HA1 GLY    34        8.854   0.583  35.844  1.00
54.41           H
ATOM    450  HA2 GLY    34        7.440   0.100  34.916  1.00
62.09           H
ATOM    451  N   ASN    35        8.918   2.447  33.395  1.00
32.51           N
ATOM    452  CA  ASN    35        8.818   3.804  32.866  1.00
33.20           C
ATOM    453  C   ASN    35        9.262   3.836  31.407  1.00
23.17           C
ATOM    454  O   ASN    35       10.116   3.051  30.992  1.00
27.01           O
ATOM    455  CB  ASN    35        9.693   4.757  33.683  1.00
54.51           C
ATOM    456  CG  ASN    35        9.252   4.753  35.143  1.00
80.73           C
ATOM    457  ND2 ASN    35       10.123   4.484  36.075
1.00220.46           N
ATOM    458  OD1 ASN    35        8.084   5.003  35.439
1.00117.80           O
ATOM    459  HN  ASN    35        9.529   1.811  32.967  1.00
51.15           H
ATOM    460  HA  ASN    35        7.792   4.133  32.926  1.00
47.21           H
ATOM    461  HB1 ASN    35        9.600   5.755  33.283  1.00
```

```
68.31           H
ATOM    462  HB2 ASN    35      10.725   4.439  33.618  1.00
57.75           H
ATOM    463 HD21 ASN    35      11.053   4.286  35.837
1.00405.39           H
ATOM    464 HD22 ASN    35       9.848   4.480  37.017
1.00245.64           H
ATOM    465  N   PHE    36       8.674   4.742  30.627  1.00
35.72           N
ATOM    466  CA  PHE    36       9.002   4.874  29.215  1.00
33.41           C
ATOM    467  C   PHE    36       9.885   6.095  28.978  1.00
28.51           C
ATOM    468  O   PHE    36       9.671   7.153  29.570  1.00
45.23           O
ATOM    469  CB  PHE    36       7.706   5.019  28.432  1.00
67.53           C
ATOM    470  CG  PHE    36       6.895   3.752  28.566  1.00
86.71           C
ATOM    471  CD1 PHE    36       5.948   3.639  29.591
1.00106.51           C
ATOM    472  CD2 PHE    36       7.090   2.694  27.671  1.00
99.48           C
ATOM    473  CE1 PHE    36       5.194   2.465  29.718
1.00134.48           C
ATOM    474  CE2 PHE    36       6.336   1.522  27.800
1.00134.27           C
ATOM    475  CZ  PHE    36       5.388   1.408  28.823
1.00149.48           C
ATOM    476  HN  PHE    36       7.997   5.334  31.006  1.00
62.87           H
ATOM    477  HA  PHE    36       9.519   3.987  28.874  1.00
28.53           H
ATOM    478  HB1 PHE    36       7.934   5.193  27.401  1.00
73.56           H
ATOM    479  HB2 PHE    36       7.140   5.854  28.822  1.00
88.42           H
ATOM    480  HD1 PHE    36       5.800   4.455  30.283
1.00110.20           H
ATOM    481  HD2 PHE    36       7.822   2.781  26.882  1.00
93.07           H
ATOM    482  HE1 PHE    36       4.464   2.378  30.510
1.00155.06           H
ATOM    483  HE2 PHE    36       6.486   0.704  27.108
1.00159.46           H
ATOM    484  HZ  PHE    36       4.806   0.503  28.922
1.00183.25           H
ATOM    485  N   THR    37      10.879   5.940  28.107  1.00
20.06           N
ATOM    486  CA  THR    37      11.789   7.035  27.792  1.00
27.18           C
ATOM    487  C   THR    37      12.469   6.791  26.448  1.00
```

```
41.87           C
ATOM    488  O   THR    37      13.100   5.755  26.236
1.00169.86           O
ATOM    489  CB  THR    37      12.848   7.168  28.894  1.00
26.52           C
ATOM    490  CG2 THR    37      13.251   5.778  29.397  1.00
75.69           C
ATOM    491  OG1 THR    37      13.987   7.838  28.373  1.00
81.95           O
ATOM    492  HN  THR    37      10.999   5.077  27.663  1.00
18.47           H
ATOM    493  HA  THR    37      11.226   7.955  27.737  1.00
38.80           H
ATOM    494  HB  THR    37      12.442   7.737  29.716  1.00
81.60           H
ATOM    495  HG1 THR    37      14.556   8.073  29.110
1.00188.80           H
ATOM    496 HG21 THR    37      14.211   5.840  29.890
1.00196.98           H
ATOM    497 HG22 THR    37      13.319   5.094  28.565
1.00196.23           H
ATOM    498 HG23 THR    37      12.510   5.420  30.096
1.00168.92           H
ATOM    499  N   GLU    38      12.325   7.752  25.544  1.00
26.53           N
ATOM    500  CA  GLU    38      12.916   7.647  24.212  1.00
30.29           C
ATOM    501  C   GLU    38      14.379   8.083  24.223  1.00
25.39           C
ATOM    502  O   GLU    38      14.851   8.692  25.184  1.00
51.60           O
ATOM    503  CB  GLU    38      12.133   8.520  23.227  1.00
54.82           C
ATOM    504  CG  GLU    38      10.689   8.011  23.112
1.00165.84           C
ATOM    505  CD  GLU    38       9.856   8.507  24.292
1.00292.92           C
ATOM    506  OE1 GLU    38       8.690   8.155  24.356
1.00414.19           O
ATOM    507  OE2 GLU    38      10.396   9.226  25.115
1.00537.65           O1-
ATOM    508  HN  GLU    38      11.804   8.548  25.776  1.00
84.50           H
ATOM    509  HA  GLU    38      12.861   6.619  23.884  1.00
32.15           H
ATOM    510  HB1 GLU    38      12.604   8.473  22.256
1.00144.82           H
ATOM    511  HB2 GLU    38      12.132   9.543  23.575
1.00106.49           H
ATOM    512  HG1 GLU    38      10.684   6.931  23.099
1.00303.31           H
ATOM    513  HG2 GLU    38      10.256   8.380  22.193
```

```
1.00323.99           H
ATOM    514  N   CYS    39      15.088   7.767  23.143  1.00
15.84        N
ATOM    515  CA  CYS    39      16.496   8.128  23.021  1.00
14.30        C
ATOM    516  C   CYS    39      16.651   9.652  23.009  1.00
22.31        C
ATOM    517  O   CYS    39      15.746  10.361  22.566  1.00
32.92        O
ATOM    518  CB  CYS    39      17.058   7.539  21.721  1.00
18.08        C
ATOM    519  SG  CYS    39      15.994   8.012  20.334  1.00
17.41        S
ATOM    520  HN  CYS    39      14.652   7.287  22.411  1.00
24.87        H
ATOM    521  HA  CYS    39      17.036   7.711  23.856  1.00
14.60        H
ATOM    522  HB1 CYS    39      17.093   6.464  21.799  1.00
27.71        H
ATOM    523  HB2 CYS    39      18.053   7.918  21.551  1.00
34.91        H
ATOM    524  N   PRO    40      17.760  10.179  23.480  1.00
27.27        N
ATOM    525  CA  PRO    40      17.988  11.652  23.509  1.00
47.66        C
ATOM    526  C   PRO    40      18.305  12.205  22.122  1.00
71.54        C
ATOM    527  O   PRO    40      18.994  11.566  21.326  1.00
87.70        O
ATOM    528  CB  PRO    40      19.178  11.809  24.463  1.00
53.85        C
ATOM    529  CG  PRO    40      19.953  10.545  24.302  1.00
46.04        C
ATOM    530  CD  PRO    40      18.918   9.447  24.030  1.00
25.70        C
ATOM    531  HA  PRO    40      17.127  12.153  23.920  1.00
58.04        H
ATOM    532  HB1 PRO    40      18.833  11.906  25.483  1.00
67.93        H
ATOM    533  HB2 PRO    40      19.779  12.668  24.186  1.00
70.99        H
ATOM    534  HG1 PRO    40      20.499  10.318  25.207  1.00
65.91        H
ATOM    535  HG2 PRO    40      20.638  10.633  23.467  1.00
54.99        H
ATOM    536  HD1 PRO    40      18.644   8.947  24.946  1.00
24.22        H
ATOM    537  HD2 PRO    40      19.299   8.743  23.310  1.00
26.94        H
ATOM    538  N   GLY    41      17.797  13.399  21.849
1.00105.24           N
ATOM    539  CA  GLY    41      18.019  14.054  20.561
```

```
1.00147.55              C
ATOM    540  C   GLY    41       19.252  14.950  20.611
1.00161.74              C
ATOM    541  O   GLY    41       19.593  15.606  19.627
1.00244.37              O
ATOM    542  HN  GLY    41       17.260  13.850  22.530
1.00121.79              H
ATOM    543  HA1 GLY    41       17.159  14.657  20.317
1.00184.40              H
ATOM    544  HA2 GLY    41       18.157  13.305  19.794
1.00157.34              H
ATOM    545  N   LEU    42       19.913  14.978  21.768
1.00162.48              N
ATOM    546  CA  LEU    42       21.111  15.803  21.948
1.00191.48              C
ATOM    547  C   LEU    42       22.359  14.930  21.993
1.00166.66              C
ATOM    548  O   LEU    42       22.352  13.845  22.575
1.00251.38              O
ATOM    549  CB  LEU    42       20.999  16.595  23.254
1.00307.99              C
ATOM    550  CG  LEU    42       19.660  17.341  23.295
1.00452.28              C
ATOM    551  CD1 LEU    42       19.575  18.150  24.590
1.00681.24              C
ATOM    552  CD2 LEU    42       19.547  18.282  22.085
1.00527.46              C
ATOM    553  HN  LEU    42       19.590  14.436  22.518
1.00195.47              H
ATOM    554  HA  LEU    42       21.202  16.499  21.126
1.00229.77              H
ATOM    555  HB1 LEU    42       21.807  17.307  23.310
1.00342.49              H
ATOM    556  HB2 LEU    42       21.058  15.915  24.091
1.00335.33              H
ATOM    557  HG  LEU    42       18.853  16.623  23.270
1.00427.79              H
ATOM    558 HD11 LEU    42       19.591  17.479  25.438
1.00909.71              H
ATOM    559 HD12 LEU    42       18.655  18.718  24.601
1.00706.80              H
ATOM    560 HD13 LEU    42       20.414  18.826  24.650
1.00858.82              H
ATOM    561 HD21 LEU    42       19.228  17.717  21.221
1.00631.69              H
ATOM    562 HD22 LEU    42       20.506  18.738  21.885
1.00628.06              H
ATOM    563 HD23 LEU    42       18.820  19.054  22.293
1.00656.75              H
ATOM    564  N   THR    43       23.435  15.413  21.371
1.00178.52              N
ATOM    565  CA  THR    43       24.701  14.678  21.335
```

```
1.00240.84           C
ATOM    566  C   THR    43       25.869  15.613  21.646
1.00372.31           C
ATOM    567  O   THR    43       26.723  15.854  20.794
1.00500.13           O
ATOM    568  CB  THR    43       24.904  14.064  19.950
1.00354.13           C
ATOM    569  CG2 THR    43       23.790  13.055  19.669
1.00463.04           C
ATOM    570  OG1 THR    43       24.876  15.091  18.969
1.00508.50           O
ATOM    571  HN  THR    43       23.376  16.284  20.925
1.00229.59           H
ATOM    572  HA  THR    43       24.682  13.881  22.065
1.00249.90           H
ATOM    573  HB  THR    43       25.856  13.559  19.921
1.00481.58           H
ATOM    574  HG1 THR    43       25.140  14.707  18.130
1.00624.31           H
ATOM    575 HG21 THR    43       23.770  12.312  20.453
1.00650.95           H
ATOM    576 HG22 THR    43       23.972  12.572  18.720
1.00580.73           H
ATOM    577 HG23 THR    43       22.839  13.568  19.635
1.00571.99           H
ATOM    578  N   PRO    44       25.917  16.140  22.842
1.00488.39           N
ATOM    579  CA  PRO    44       27.002  17.071  23.267
1.00760.13           C
ATOM    580  C   PRO    44       28.315  16.333  23.519
1.00735.36           C
ATOM    581  O   PRO    44       29.382  16.945  23.585
1.00999.99           O
ATOM    582  CB  PRO    44       26.448  17.697  24.554
1.00999.99           C
ATOM    583  CG  PRO    44       25.545  16.650  25.124
1.00856.94           C
ATOM    584  CD  PRO    44       24.945  15.904  23.926
1.00548.09           C
ATOM    585  HA  PRO    44       27.145  17.840  22.523
1.00939.00           H
ATOM    586  HB1 PRO    44       25.881  18.588  24.324
1.00999.99           H
ATOM    587  HB2 PRO    44       27.250  17.928  25.243
1.00999.99           H
ATOM    588  HG1 PRO    44       24.757  17.107  25.703
1.00999.99           H
ATOM    589  HG2 PRO    44       26.115  15.969  25.746
1.00853.53           H
ATOM    590  HD1 PRO    44       23.983  16.314  23.658
1.00602.45           H
ATOM    591  HD2 PRO    44       24.864  14.847  24.142
```

```
1.00463.32           H
ATOM    592  N   ILE    45      28.226  15.014  23.659
1.00518.33           N
ATOM    593  CA  ILE    45      29.409  14.201  23.905
1.00582.79           C
ATOM    594  C   ILE    45      30.476  14.472  22.849
1.00795.93           C
ATOM    595  O   ILE    45      30.170  14.609  21.665
1.00898.75           O
ATOM    596  CB  ILE    45      29.035  12.717  23.886
1.00474.41           C
ATOM    597  CG1 ILE    45      30.249  11.880  24.300
1.00516.90           C
ATOM    598  CG2 ILE    45      28.602  12.318  22.474
1.00760.75           C
ATOM    599  CD1 ILE    45      29.806  10.441  24.573
1.00566.81           C
ATOM    600  HN  ILE    45      27.348  14.583  23.598
1.00395.72           H
ATOM    601  HA  ILE    45      29.806  14.447  24.878
1.00711.06           H
ATOM    602  HB  ILE    45      28.222  12.542  24.575
1.00477.81           H
ATOM    603 HG11 ILE    45      30.684  12.296  25.195
1.00624.28           H
ATOM    604 HG12 ILE    45      30.980  11.888  23.504
1.00744.84           H
ATOM    605 HG21 ILE    45      29.468  12.279  21.829
1.00920.57           H
ATOM    606 HG22 ILE    45      27.902  13.049  22.093
1.00999.99           H
ATOM    607 HG23 ILE    45      28.130  11.348  22.502
1.00865.46           H
ATOM    608 HD11 ILE    45      30.677   9.819  24.719
1.00658.89           H
ATOM    609 HD12 ILE    45      29.237  10.073  23.732
1.00734.30           H
ATOM    610 HD13 ILE    45      29.192  10.416  25.461
1.00675.84           H
ATOM    611  N   ALA    46      31.728  14.547  23.284
1.00999.99           N
ATOM    612  CA  ALA    46      32.832  14.803  22.367
1.00999.99           C
ATOM    613  C   ALA    46      33.081  13.591  21.477
1.00999.99           C
ATOM    614  CB  ALA    46      34.103  15.130  23.155
1.00999.99           C
ATOM    615  OT1 ALA    46      32.214  13.280  20.675
1.00999.99           O
ATOM    616  OT2 ALA    46      34.134  12.989  21.608
1.00999.99           O
ATOM    617  HN  ALA    46      31.914  14.428  24.240
```

```
1.00999.99           H
ATOM    618  HA  ALA    46       32.582  15.649  21.745
1.00999.99           H
ATOM    619  HB1 ALA    46       34.487  14.227  23.607
1.00999.99           H
ATOM    620  HB2 ALA    46       33.874  15.850  23.927
1.00999.99           H
ATOM    621  HB3 ALA    46       34.845  15.541  22.486
1.00999.99           H
ENDMDL
TER
MODEL       15
ATOM      1  N   GLY     1       27.181   0.115  14.390
1.00999.99           N
ATOM      2  CA  GLY     1       26.672   0.645  13.092
1.00999.99           C
ATOM      3  C   GLY     1       25.287   1.249  13.297
1.00999.99           C
ATOM      4  O   GLY     1       24.967   1.741  14.378
1.00999.99           O
ATOM      5  HA1 GLY     1       26.608  -0.159  12.374
1.00999.99           H
ATOM      6  HA2 GLY     1       27.347   1.404  12.725
1.00999.99           H
ATOM      7  HT1 GLY     1       28.175  -0.168  14.282
1.00999.99           H
ATOM      8  HT2 GLY     1       26.614  -0.711  14.673
1.00999.99           H
ATOM      9  HT3 GLY     1       27.109   0.852  15.118
1.00999.99           H
ATOM     10  N   LEU     2       24.470   1.206  12.250
1.00999.99           N
ATOM     11  CA  LEU     2       23.117   1.753  12.321
1.00895.59           C
ATOM     12  C   LEU     2       22.173   0.753  12.982
1.00532.27           C
ATOM     13  O   LEU     2       22.386  -0.458  12.907
1.00625.21           O
ATOM     14  CB  LEU     2       22.610   2.076  10.912
1.00999.99           C
ATOM     15  CG  LEU     2       23.579   3.036  10.211
1.00999.99           C
ATOM     16  CD1 LEU     2       23.080   3.301   8.789
1.00999.99           C
ATOM     17  CD2 LEU     2       23.664   4.364  10.986
1.00999.99           C
ATOM     18  HN  LEU     2       24.780   0.802  11.412
1.00999.99           H
ATOM     19  HA  LEU     2       23.130   2.659  12.906
1.00890.87           H
ATOM     20  HB1 LEU     2       21.636   2.539  10.980
1.00924.07           H
```

```
ATOM     21  HB2 LEU     2      22.533   1.163  10.341
1.00999.99           H
ATOM     22  HG  LEU     2      24.559   2.582  10.165
1.00999.99           H
ATOM     23 HD11 LEU     2      23.692   4.064   8.328
1.00999.99           H
ATOM     24 HD12 LEU     2      22.054   3.637   8.823
1.00999.99           H
ATOM     25 HD13 LEU     2      23.142   2.393   8.209
1.00999.99           H
ATOM     26 HD21 LEU     2      22.691   4.615  11.388
1.00999.99           H
ATOM     27 HD22 LEU     2      23.991   5.152  10.324
1.00999.99           H
ATOM     28 HD23 LEU     2      24.373   4.264  11.793
1.00999.99           H
ATOM     29  N   CYS     3      21.128   1.265  13.626
1.00271.28           N
ATOM     30  CA  CYS     3      20.160   0.402  14.293
1.00104.50           C
ATOM     31  C   CYS     3      19.420  -0.454  13.269  1.00
90.98           C
ATOM     32  O   CYS     3      19.179  -0.021  12.142
1.00207.28           O
ATOM     33  CB  CYS     3      19.151   1.247  15.076  1.00
37.83           C
ATOM     34  SG  CYS     3      19.991   2.089  16.441  1.00
81.56           S
ATOM     35  HN  CYS     3      21.007   2.237  13.651
1.00296.55           H
ATOM     36  HA  CYS     3      20.682  -0.247  14.980
1.00166.45           H
ATOM     37  HB1 CYS     3      18.376   0.608  15.471  1.00
40.38           H
ATOM     38  HB2 CYS     3      18.711   1.982  14.419  1.00
98.45           H
ATOM     39  N   SER     4      19.061  -1.670  13.668  1.00
85.31           N
ATOM     40  CA  SER     4      18.348  -2.578  12.775  1.00
92.94           C
ATOM     41  C   SER     4      17.026  -1.963  12.351  1.00
67.37           C
ATOM     42  O   SER     4      16.626  -2.044  11.190
1.00102.28           O
ATOM     43  CB  SER     4      18.051  -3.890  13.492
1.00123.83           C
ATOM     44  OG  SER     4      17.480  -4.810  12.569
1.00178.89           O
ATOM     45  HN  SER     4      19.279  -1.961  14.578
1.00163.03           H
ATOM     46  HA  SER     4      18.951  -2.775  11.903
1.00129.78           H
```

```
ATOM     47  HB1 SER     4      17.348  -3.699  14.299
1.00105.26          H
ATOM     48  HB2 SER     4      18.960  -4.304  13.896
1.00156.87          H
ATOM     49  HG  SER     4      18.160  -5.056  11.937
1.00218.16          H
ATOM     50  N   GLU     5      16.343  -1.368  13.323  1.00
41.67          N
ATOM     51  CA  GLU     5      15.044  -0.754  13.086  1.00
41.21          C
ATOM     52  C   GLU     5      14.898   0.519  13.912  1.00
41.92          C
ATOM     53  O   GLU     5      15.859   0.990  14.519  1.00
74.72          O
ATOM     54  CB  GLU     5      13.948  -1.746  13.465  1.00
49.24          C
ATOM     55  CG  GLU     5      14.105  -2.115  14.933  1.00
55.48          C
ATOM     56  CD  GLU     5      13.194  -3.288  15.279  1.00
92.06          C
ATOM     57  OE1 GLU     5      13.207  -3.706  16.426
1.00206.49          O
ATOM     58  OE2 GLU     5      12.498  -3.755  14.392
1.00197.24          O1-
ATOM     59  HN  GLU     5      16.716  -1.358  14.229  1.00
45.52          H
ATOM     60  HA  GLU     5      14.946  -0.511  12.046  1.00
60.63          H
ATOM     61  HB1 GLU     5      14.039  -2.634  12.859  1.00
61.59          H
ATOM     62  HB2 GLU     5      12.987  -1.296  13.307  1.00
63.66          H
ATOM     63  HG1 GLU     5      13.842  -1.267  15.547  1.00
58.03          H
ATOM     64  HG2 GLU     5      15.131  -2.387  15.115  1.00
57.77          H
ATOM     65  N   ASN     6      13.690   1.074  13.932  1.00
50.73          N
ATOM     66  CA  ASN     6      13.439   2.294  14.690  1.00
71.04          C
ATOM     67  C   ASN     6      13.738   2.065  16.167  1.00
65.17          C
ATOM     68  O   ASN     6      14.292   2.934  16.841  1.00
94.86          O
ATOM     69  CB  ASN     6      11.982   2.726  14.520  1.00
94.83          C
ATOM     70  CG  ASN     6      11.754   3.253  13.108
1.00160.94          C
ATOM     71  ND2 ASN     6      10.542   3.300  12.625
1.00244.88          N
ATOM     72  OD1 ASN     6      12.706   3.634  12.427
1.00219.73          O
```

```
ATOM     73  HN  ASN     6       12.959   0.657  13.430  1.00
72.81           H
ATOM     74  HA  ASN     6       14.082   3.077  14.317  1.00
96.02           H
ATOM     75  HB1 ASN     6       11.752   3.504  15.233
1.00111.09           H
ATOM     76  HB2 ASN     6       11.335   1.878  14.695  1.00
98.13           H
ATOM     77 HD21 ASN     6        9.786   2.997  13.170
1.00272.43           H
ATOM     78 HD22 ASN     6       10.390   3.638  11.718
1.00336.78           H
ATOM     79  N   GLY     7       13.372   0.884  16.663  1.00
51.80           N
ATOM     80  CA  GLY     7       13.606   0.528  18.064  1.00
71.47           C
ATOM     81  C   GLY     7       14.250  -0.849  18.164  1.00
41.04           C
ATOM     82  O   GLY     7       13.572  -1.849  18.403  1.00
44.54           O
ATOM     83  HN  GLY     7       12.939   0.233  16.071  1.00
45.49           H
ATOM     84  HA1 GLY     7       12.665   0.513  18.588
1.00103.52           H
ATOM     85  HA2 GLY     7       14.258   1.259  18.525
1.00108.67           H
ATOM     86  N   ASP     8       15.564  -0.894  17.979  1.00
27.00           N
ATOM     87  CA  ASP     8       16.295  -2.153  18.049  1.00
14.15           C
ATOM     88  C   ASP     8       16.156  -2.777  19.431  1.00
9.37           C
ATOM     89  O   ASP     8       15.945  -3.983  19.562  1.00
14.61           O
ATOM     90  CB  ASP     8       17.775  -1.919  17.735  1.00
15.06           C
ATOM     91  CG  ASP     8       18.519  -3.249  17.707  1.00
21.22           C
ATOM     92  OD1 ASP     8       19.674  -3.251  17.312
1.00119.56           O
ATOM     93  OD2 ASP     8       17.925  -4.247  18.079
1.00133.27           O1-
ATOM     94  HN  ASP     8       16.049  -0.064  17.792  1.00
35.63           H
ATOM     95  HA  ASP     8       15.889  -2.833  17.317  1.00
20.92           H
ATOM     96  HB1 ASP     8       18.204  -1.285  18.497  1.00
29.39           H
ATOM     97  HB2 ASP     8       17.866  -1.435  16.774  1.00
44.14           H
ATOM     98  N   CYS     9       16.277  -1.946  20.456  1.00
5.93           N
```

ATOM   99 CA CYS   9   16.161 -2.421 21.829 1.00
7.54   C
ATOM   100 C   CYS   9   14.770 -2.992 22.076 1.00
14.60   C
ATOM   101 O   CYS   9   14.613 -4.031 22.716 1.00
25.78   O
ATOM   102 CB CYS   9   16.437 -1.273 22.791 1.00
6.63   C
ATOM   103 SG CYS   9   18.189 -0.873 22.699 1.00
10.91   S
ATOM   104 HN CYS   9   16.444 -0.997 20.285 1.00
6.51   H
ATOM   105 HA CYS   9   16.900 -3.186 21.994 1.00
11.12   H
ATOM   106 HB1 CYS   9   16.191 -1.566 23.798 1.00
11.28   H
ATOM   107 HB2 CYS   9   15.850 -0.410 22.508 1.00
5.04   H
ATOM   108 N   ALA   10   13.767 -2.303 21.547 1.00
16.26   N
ATOM   109 CA ALA   10   12.380 -2.733 21.688 1.00
32.04   C
ATOM   110 C   ALA   10   11.477 -1.869 20.810 1.00
45.47   C
ATOM   111 O   ALA   10   11.869 -0.780 20.391
1.00119.04   O
ATOM   112 CB ALA   10   11.940 -2.636 23.152 1.00
30.10   C
ATOM   113 HN ALA   10   13.965 -1.488 21.041 1.00
12.81   H
ATOM   114 HA ALA   10   12.300 -3.762 21.365 1.00
47.14   H
ATOM   115 HB1 ALA   10   12.759 -2.925 23.794 1.00
84.37   H
ATOM   116 HB2 ALA   10   11.101 -3.297 23.322
1.00118.20   H
ATOM   117 HB3 ALA   10   11.648 -1.622 23.378
1.00117.32   H
ATOM   118 N   ALA   11   10.272 -2.355 20.528 1.00
30.62   N
ATOM   119 CA ALA   11   9.343 -1.603 19.693 1.00
35.24   C
ATOM   120 C   ALA   11   9.013 -0.255 20.331 1.00
22.78   C
ATOM   121 O   ALA   11   9.034   0.779 19.663 1.00
53.33   O
ATOM   122 CB ALA   11   8.054 -2.403 19.496 1.00
60.49   C
ATOM   123 HN ALA   11   10.007 -3.229 20.884 1.00
50.37   H
ATOM   124 HA ALA   11   9.797 -1.433 18.729 1.00
41.70   H

```
ATOM    125  HB1 ALA    11        7.485  -1.978  18.682
1.00157.06          H
ATOM    126  HB2 ALA    11        7.466  -2.368  20.401
1.00148.83          H
ATOM    127  HB3 ALA    11        8.299  -3.430  19.266
1.00137.06          H
ATOM    128  N   ASP    12        8.718  -0.272  21.627  1.00
18.98         N
ATOM    129  CA  ASP    12        8.396   0.957  22.344  1.00
31.05         C
ATOM    130  C   ASP    12        9.641   1.825  22.504  1.00
22.98         C
ATOM    131  O   ASP    12        9.577   3.050  22.410  1.00
37.14         O
ATOM    132  CB  ASP    12        7.824   0.624  23.723  1.00
48.35         C
ATOM    133  CG  ASP    12        6.427   0.029  23.580
1.00104.83          C
ATOM    134  OD1 ASP    12        5.869   0.131  22.500
1.00258.73          O
ATOM    135  OD2 ASP    12        5.936  -0.519  24.552
1.00213.46          O1-
ATOM    136  HN  ASP    12        8.723  -1.125  22.111  1.00
36.19         H
ATOM    137  HA  ASP    12        7.656   1.507  21.783  1.00
49.76         H
ATOM    138  HB1 ASP    12        7.769   1.525  24.316  1.00
95.71         H
ATOM    139  HB2 ASP    12        8.469  -0.090  24.217  1.00
54.37         H
ATOM    140  N   GLU    13       10.772   1.171  22.753  1.00
17.60         N
ATOM    141  CA  GLU    13       12.038   1.872  22.935  1.00
12.01         C
ATOM    142  C   GLU    13       12.596   2.351  21.597  1.00
10.07         C
ATOM    143  O   GLU    13       12.121   1.946  20.535  1.00
12.81         O
ATOM    144  CB  GLU    13       13.043   0.940  23.601  1.00
11.74         C
ATOM    145  CG  GLU    13       12.497   0.495  24.960  1.00
13.49         C
ATOM    146  CD  GLU    13       12.548   1.654  25.948
1.00142.36          C
ATOM    147  OE1 GLU    13       13.150   2.660  25.618
1.00339.06          O
ATOM    148  OE2 GLU    13       11.985   1.519  27.021
1.00335.28          O1-
ATOM    149  HN  GLU    13       10.752   0.194  22.820  1.00
28.36         H
ATOM    150  HA  GLU    13       11.882   2.726  23.576  1.00
13.06         H
```

```
ATOM    151  HB1 GLU    13       13.975   1.462  23.743  1.00
13.76           H
ATOM    152  HB2 GLU    13       13.206   0.075  22.974  1.00
9.94            H
ATOM    153  HG1 GLU    13       13.088  -0.323  25.335  1.00
66.02           H
ATOM    154  HG2 GLU    13       11.473   0.174  24.845  1.00
56.23           H
ATOM    155  N   CYS    14       13.612   3.215  21.657  1.00
9.12            N
ATOM    156  CA  CYS    14       14.239   3.752  20.445  1.00
10.69           C
ATOM    157  C   CYS    14       15.668   3.233  20.313  1.00
9.56            C
ATOM    158  O   CYS    14       16.203   2.619  21.234  1.00
13.62           O
ATOM    159  CB  CYS    14       14.264   5.283  20.501  1.00
13.96           C
ATOM    160  SG  CYS    14       15.504   5.826  21.695  1.00
42.96           S
ATOM    161  HN  CYS    14       13.948   3.498  22.535  1.00
9.76            H
ATOM    162  HA  CYS    14       13.675   3.444  19.575  1.00
15.54           H
ATOM    163  HB1 CYS    14       13.298   5.650  20.803  1.00
50.72           H
ATOM    164  HB2 CYS    14       14.509   5.675  19.525  1.00
46.13           H
ATOM    165  N   CYS    15       16.280   3.500  19.163  1.00
9.60            N
ATOM    166  CA  CYS    15       17.653   3.073  18.911  1.00
9.28            C
ATOM    167  C   CYS    15       18.306   4.025  17.918  1.00
9.92            C
ATOM    168  O   CYS    15       17.764   4.276  16.842  1.00
13.31           O
ATOM    169  CB  CYS    15       17.663   1.653  18.339  1.00
12.90           C
ATOM    170  SG  CYS    15       19.373   1.106  18.097  1.00
39.11           S
ATOM    171  HN  CYS    15       15.803   4.003  18.471  1.00
13.12           H
ATOM    172  HA  CYS    15       18.211   3.085  19.838  1.00
8.26            H
ATOM    173  HB1 CYS    15       17.146   1.642  17.391  1.00
24.82           H
ATOM    174  HB2 CYS    15       17.166   0.985  19.027  1.00
27.31           H
ATOM    175  N   VAL    16       19.468   4.564  18.280  1.00
9.56            N
ATOM    176  CA  VAL    16       20.168   5.495  17.399  1.00
11.99           C
```

```
ATOM    177  C   VAL    16      21.675   5.382  17.592  1.00
7.73           C
ATOM    178  O   VAL    16      22.174   5.425  18.717  1.00
8.83           O
ATOM    179  CB  VAL    16      19.711   6.925  17.698  1.00
18.37           C
ATOM    180  CG1 VAL    16      20.047   7.280  19.147  1.00
39.61           C
ATOM    181  CG2 VAL    16      20.424   7.894  16.754
1.00115.21           C
ATOM    182  HN  VAL    16      19.859   4.337  19.151  1.00
9.76           H
ATOM    183  HA  VAL    16      19.934   5.263  16.370  1.00
17.27           H
ATOM    184  HB  VAL    16      18.644   6.996  17.548  1.00
52.63           H
ATOM    185 HG11 VAL    16      19.764   6.462  19.793
1.00128.07           H
ATOM    186 HG12 VAL    16      19.505   8.169  19.435
1.00154.13           H
ATOM    187 HG13 VAL    16      21.108   7.461  19.240
1.00135.74           H
ATOM    188 HG21 VAL    16      20.287   7.569  15.734
1.00229.05           H
ATOM    189 HG22 VAL    16      21.479   7.915  16.988
1.00261.82           H
ATOM    190 HG23 VAL    16      20.008   8.884  16.875
1.00210.07           H
ATOM    191  N   ASP    17      22.399   5.233  16.481  1.00
14.34           N
ATOM    192  CA  ASP    17      23.858   5.110  16.523  1.00
11.90           C
ATOM    193  C   ASP    17      24.520   6.298  15.838  1.00
13.15           C
ATOM    194  O   ASP    17      24.136   6.690  14.736  1.00
25.96           O
ATOM    195  CB  ASP    17      24.292   3.819  15.827  1.00
20.80           C
ATOM    196  CG  ASP    17      23.644   2.618  16.508  1.00
28.72           C
ATOM    197  OD1 ASP    17      23.717   2.541  17.724
1.00135.42           O
ATOM    198  OD2 ASP    17      23.081   1.795  15.804
1.00114.17           O1-
ATOM    199  HN  ASP    17      21.943   5.205  15.614  1.00
28.29           H
ATOM    200  HA  ASP    17      24.191   5.075  17.552  1.00
10.49           H
ATOM    201  HB1 ASP    17      25.368   3.726  15.886  1.00
22.42           H
ATOM    202  HB2 ASP    17      23.991   3.851  14.791  1.00
29.52           H
```

```
ATOM    203  N   THR    18      25.534   6.854  16.495  1.00
11.55          N
ATOM    204  CA  THR    18      26.281   7.990  15.957  1.00
17.10          C
ATOM    205  C   THR    18      27.766   7.672  15.957  1.00
10.63          C
ATOM    206  O   THR    18      28.211   6.768  16.661  1.00
6.49          O
ATOM    207  CB  THR    18      26.031   9.242  16.800  1.00
28.52          C
ATOM    208  CG2 THR    18      24.618   9.763  16.552  1.00
45.38          C
ATOM    209  OG1 THR    18      26.194   8.924  18.172  1.00
26.49          O
ATOM    210  HN  THR    18      25.796   6.483  17.364  1.00
13.96          H
ATOM    211  HA  THR    18      25.967   8.185  14.940  1.00
26.43          H
ATOM    212  HB  THR    18      26.742  10.006  16.523  1.00
37.43          H
ATOM    213  HG1 THR    18      25.433   9.266  18.648  1.00
71.62          H
ATOM    214 HG21 THR    18      23.924   8.938  16.558
1.00100.44          H
ATOM    215 HG22 THR    18      24.585  10.258  15.592
1.00129.42          H
ATOM    216 HG23 THR    18      24.355  10.465  17.329
1.00156.21          H
ATOM    217  N   VAL    19      28.527   8.426  15.178  1.00
16.32          N
ATOM    218  CA  VAL    19      29.951   8.233  15.097  1.00
13.34          C
ATOM    219  C   VAL    19      30.546   9.258  14.155  1.00
25.88          C
ATOM    220  O   VAL    19      30.245   9.300  12.963  1.00
42.11          O
ATOM    221  CB  VAL    19      30.304   6.822  14.625  1.00
15.48          C
ATOM    222  CG1 VAL    19      29.528   6.479  13.349  1.00
29.06          C
ATOM    223  CG2 VAL    19      31.812   6.754  14.344  1.00
20.67          C
ATOM    224  HN  VAL    19      28.126   9.145  14.656  1.00
26.89          H
ATOM    225  HA  VAL    19      30.373   8.382  16.081  1.00
8.08          H
ATOM    226  HB  VAL    19      30.051   6.120  15.401  1.00
11.49          H
ATOM    227 HG11 VAL    19      28.492   6.757  13.468
1.00137.43          H
ATOM    228 HG12 VAL    19      29.595   5.417  13.163  1.00
93.29          H
```

```
ATOM    229 HG13 VAL    19       29.951   7.017  12.514
1.00108.69           H
ATOM    230 HG21 VAL    19       32.024   7.249  13.406  1.00
99.42           H
ATOM    231 HG22 VAL    19       32.124   5.722  14.288
1.00111.36           H
ATOM    232 HG23 VAL    19       32.349   7.253  15.140  1.00
77.11           H
ATOM    233  N   PHE    20       31.383  10.080  14.727  1.00
24.58           N
ATOM    234  CA  PHE    20       32.055  11.143  14.004  1.00
40.34           C
ATOM    235  C   PHE    20       33.456  10.715  13.600  1.00
40.37           C
ATOM    236  O   PHE    20       33.837  10.822  12.433  1.00
66.02           O
ATOM    237  CB  PHE    20       32.123  12.396  14.892  1.00
48.79           C
ATOM    238  CG  PHE    20       32.241  12.016  16.362  1.00
35.75           C
ATOM    239  CD1 PHE    20       31.170  11.388  17.033  1.00
30.20           C
ATOM    240  CD2 PHE    20       33.417  12.315  17.064  1.00
40.95           C
ATOM    241  CE1 PHE    20       31.290  11.064  18.386  1.00
30.41           C
ATOM    242  CE2 PHE    20       33.530  11.985  18.420  1.00
45.84           C
ATOM    243  CZ  PHE    20       32.468  11.361  19.081  1.00
40.81           C
ATOM    244  HN  PHE    20       31.545   9.975  15.678  1.00
16.73           H
ATOM    245  HA  PHE    20       31.494  11.379  13.108  1.00
59.91           H
ATOM    246  HB1 PHE    20       31.233  12.961  14.752  1.00
67.15           H
ATOM    247  HB2 PHE    20       32.970  13.003  14.607  1.00
58.17           H
ATOM    248  HD1 PHE    20       30.256  11.147  16.506  1.00
33.34           H
ATOM    249  HD2 PHE    20       34.240  12.796  16.558  1.00
49.35           H
ATOM    250  HE1 PHE    20       30.470  10.582  18.896  1.00
30.61           H
ATOM    251  HE2 PHE    20       34.438  12.214  18.957  1.00
61.27           H
ATOM    252  HZ  PHE    20       32.556  11.108  20.126  1.00
52.66           H
ATOM    253  N   GLU    21       34.231  10.260  14.576  1.00
41.31           N
ATOM    254  CA  GLU    21       35.594   9.855  14.341  1.00
56.16           C
```

```
ATOM    255  C   GLU    21       35.812   8.397  14.713  1.00
58.61           C
ATOM    256  O   GLU    21       34.886   7.695  15.117
1.00201.19           O
ATOM    257  CB  GLU    21       36.472  10.744  15.190  1.00
57.37           C
ATOM    258  CG  GLU    21       36.225  10.455  16.670
1.00193.56           C
ATOM    259  CD  GLU    21       36.878  11.538  17.525
1.00304.62           C
ATOM    260  OE1 GLU    21       36.674  11.522  18.728
1.00451.81           O
ATOM    261  OE2 GLU    21       37.574  12.367  16.964
1.00442.88           O1-
ATOM    262  HN  GLU    21       33.892  10.227  15.488  1.00
50.82           H
ATOM    263  HA  GLU    21       35.857   9.998  13.302  1.00
85.72           H
ATOM    264  HB1 GLU    21       36.223  11.770  14.982  1.00
46.37           H
ATOM    265  HB2 GLU    21       37.494  10.561  14.952
1.00131.81           H
ATOM    266  HG1 GLU    21       36.649   9.499  16.924
1.00314.91           H
ATOM    267  HG2 GLU    21       35.165  10.436  16.860
1.00266.92           H
ATOM    268  N   GLY    22       37.051   7.958  14.564  1.00
86.70           N
ATOM    269  CA  GLY    22       37.418   6.574  14.875
1.00104.66           C
ATOM    270  C   GLY    22       37.912   6.448  16.313  1.00
71.50           C
ATOM    271  O   GLY    22       38.079   5.341  16.827  1.00
89.87           O
ATOM    272  HN  GLY    22       37.737   8.580  14.237
1.00214.64           H
ATOM    273  HA1 GLY    22       38.206   6.259  14.208
1.00152.47           H
ATOM    274  HA2 GLY    22       36.559   5.932  14.737
1.00118.15           H
ATOM    275  N   ASP    23       38.149   7.585  16.952  1.00
44.68           N
ATOM    276  CA  ASP    23       38.632   7.595  18.328  1.00
43.33           C
ATOM    277  C   ASP    23       37.617   6.961  19.271  1.00
37.55           C
ATOM    278  O   ASP    23       37.976   6.174  20.147  1.00
61.37           O
ATOM    279  CB  ASP    23       38.905   9.031  18.773  1.00
40.74           C
ATOM    280  CG  ASP    23       39.575   9.035  20.142
1.00141.86           C
```

```
ATOM    281  OD1 ASP    23       39.617   7.985  20.762
1.00328.18             O
ATOM    282  OD2 ASP    23       40.037  10.088  20.551
1.00304.58             O1-
ATOM    283  HN  ASP    23       38.005   8.435  16.488  1.00
40.86           H
ATOM    284  HA  ASP    23       39.550   7.035  18.379  1.00
72.12           H
ATOM    285  HB1 ASP    23       37.971   9.568  18.833  1.00
94.15           H
ATOM    286  HB2 ASP    23       39.550   9.515  18.054  1.00
93.26           H
ATOM    287  N   MET    24       36.352   7.320  19.093  1.00
23.24           N
ATOM    288  CA  MET    24       35.287   6.791  19.944  1.00
32.50           C
ATOM    289  C   MET    24       33.943   6.829  19.222  1.00
25.13           C
ATOM    290  O   MET    24       33.680   7.728  18.421  1.00
54.40           O
ATOM    291  CB  MET    24       35.201   7.619  21.230  1.00
47.36           C
ATOM    292  CG  MET    24       34.107   7.056  22.140
1.00151.73             C
ATOM    293  SD  MET    24       34.115   7.948  23.716
1.00209.23             S
ATOM    294  CE  MET    24       33.498   9.536  23.099
1.00243.29             C
ATOM    295  HN  MET    24       36.132   7.957  18.384  1.00
17.32           H
ATOM    296  HA  MET    24       35.515   5.769  20.204  1.00
48.94           H
ATOM    297  HB1 MET    24       34.967   8.644  20.980
1.00124.40             H
ATOM    298  HB2 MET    24       36.150   7.582  21.744
1.00166.96             H
ATOM    299  HG1 MET    24       34.294   6.007  22.320
1.00331.71             H
ATOM    300  HG2 MET    24       33.143   7.171  21.665
1.00302.30             H
ATOM    301  HE1 MET    24       33.045  10.084  23.913
1.00340.44             H
ATOM    302  HE2 MET    24       32.762   9.367  22.331
1.00373.88             H
ATOM    303  HE3 MET    24       34.321  10.106  22.689
1.00386.81             H
ATOM    304  N   VAL    25       33.088   5.849  19.522  1.00
22.44           N
ATOM    305  CA  VAL    25       31.760   5.767  18.910  1.00
15.37           C
ATOM    306  C   VAL    25       30.683   6.141  19.924  1.00
17.47           C
```

ATOM 307 O VAL 25 30.783 5.816 21.107 1.00
29.52 O
ATOM 308 CB VAL 25 31.495 4.351 18.382 1.00
23.36 C
ATOM 309 CG1 VAL 25 30.028 4.232 17.932 1.00
58.39 C
ATOM 310 CG2 VAL 25 32.418 4.078 17.191 1.00
55.25 C
ATOM 311 HN VAL 25 33.356 5.166 20.173 1.00
48.11 H
ATOM 312 HA VAL 25 31.703 6.462 18.079 1.00
9.25 H
ATOM 313 HB VAL 25 31.691 3.633 19.165 1.00
52.45 H
ATOM 314 HG11 VAL 25 29.713 5.158 17.472
1.00171.07 H
ATOM 315 HG12 VAL 25 29.407 4.031 18.791
1.00166.22 H
ATOM 316 HG13 VAL 25 29.928 3.423 17.221
1.00134.76 H
ATOM 317 HG21 VAL 25 33.441 4.023 17.532
1.00141.88 H
ATOM 318 HG22 VAL 25 32.321 4.873 16.468
1.00184.50 H
ATOM 319 HG23 VAL 25 32.142 3.140 16.731
1.00133.90 H
ATOM 320 N THR 26 29.656 6.834 19.439 1.00
13.60 N
ATOM 321 CA THR 26 28.545 7.276 20.281 1.00
21.19 C
ATOM 322 C THR 26 27.235 6.631 19.833 1.00
16.55 C
ATOM 323 O THR 26 26.921 6.601 18.643 1.00
11.43 O
ATOM 324 CB THR 26 28.429 8.794 20.186 1.00
27.07 C
ATOM 325 CG2 THR 26 27.220 9.283 20.988 1.00
44.39 C
ATOM 326 OG1 THR 26 29.613 9.383 20.703 1.00
34.75 O
ATOM 327 HN THR 26 29.647 7.061 18.487 1.00
10.81 H
ATOM 328 HA THR 26 28.735 7.005 21.311 1.00
33.16 H
ATOM 329 HB THR 26 28.312 9.076 19.147 1.00
20.60 H
ATOM 330 HG1 THR 26 30.026 8.749 21.293 1.00
77.72 H
ATOM 331 HG21 THR 26 27.234 10.362 21.038
1.00126.99 H
ATOM 332 HG22 THR 26 27.266 8.877 21.989
1.00108.32 H

ATOM 333 HG23 THR 26 26.308 8.958 20.511
1.00151.99 H
ATOM 334 N ARG 27 26.473 6.118 20.797 1.00
22.38 N
ATOM 335 CA ARG 27 25.193 5.474 20.503 1.00
20.21 C
ATOM 336 C ARG 27 24.245 5.610 21.689 1.00
16.96 C
ATOM 337 O ARG 27 24.672 5.921 22.801 1.00
20.06 O
ATOM 338 CB ARG 27 25.415 3.992 20.192 1.00
22.14 C
ATOM 339 CG ARG 27 25.991 3.296 21.425
1.00124.30 C
ATOM 340 CD ARG 27 26.437 1.879 21.052
1.00109.38 C
ATOM 341 NE ARG 27 25.284 1.074 20.661
1.00227.73 N
ATOM 342 CZ ARG 27 24.557 0.421 21.565
1.00426.12 C
ATOM 343 NH1 ARG 27 23.535 -0.296 21.186
1.00767.09 N1+
ATOM 344 NH2 ARG 27 24.865 0.500 22.830
1.00581.78 N
ATOM 345 HN ARG 27 26.777 6.174 21.728 1.00
30.75 H
ATOM 346 HA ARG 27 24.747 5.948 19.642 1.00
23.13 H
ATOM 347 HB1 ARG 27 26.109 3.896 19.370 1.00
87.46 H
ATOM 348 HB2 ARG 27 24.475 3.535 19.926
1.00103.36 H
ATOM 349 HG1 ARG 27 25.236 3.241 22.195
1.00281.97 H
ATOM 350 HG2 ARG 27 26.840 3.855 21.792
1.00276.19 H
ATOM 351 HD1 ARG 27 26.928 1.424 21.902
1.00183.60 H
ATOM 352 HD2 ARG 27 27.130 1.931 20.228
1.00142.93 H
ATOM 353 HE ARG 27 25.041 1.011 19.713
1.00372.53 H
ATOM 354 HH11 ARG 27 23.298 -0.356 20.216
1.00910.59 H
ATOM 355 HH12 ARG 27 22.989 -0.787 21.864
1.00999.99 H
ATOM 356 HH21 ARG 27 25.648 1.050 23.122
1.00532.54 H
ATOM 357 HH22 ARG 27 24.320 0.008 23.510
1.00948.84 H
ATOM 358 N SER 28 22.952 5.380 21.449 1.00
14.60 N

ATOM 359 CA SER 28 21.955 5.487 22.515 1.00
14.92 C
ATOM 360 C SER 28 20.797 4.526 22.272 1.00
12.12 C
ATOM 361 O SER 28 20.440 4.238 21.130 1.00
13.41 O
ATOM 362 CB SER 28 21.425 6.919 22.594 1.00
23.26 C
ATOM 363 OG SER 28 20.449 7.002 23.625
1.00146.32 O
ATOM 364 HN SER 28 22.663 5.138 20.543 1.00
15.23 H
ATOM 365 HA SER 28 22.418 5.238 23.460 1.00
15.44 H
ATOM 366 HB1 SER 28 20.987 7.193 21.645 1.00
88.73 H
ATOM 367 HB2 SER 28 22.236 7.593 22.818
1.00124.68 H
ATOM 368 HG SER 28 20.872 7.370 24.404
1.00242.47 H
ATOM 369 N CYS 29 20.215 4.036 23.362 1.00
10.48 N
ATOM 370 CA CYS 29 19.094 3.108 23.278 1.00
9.97 C
ATOM 371 C CYS 29 18.477 2.932 24.663 1.00
11.60 C
ATOM 372 O CYS 29 19.124 2.422 25.579 1.00
15.64 O
ATOM 373 CB CYS 29 19.568 1.751 22.729 1.00
9.52 C
ATOM 374 SG CYS 29 18.222 0.949 21.846 1.00
10.40 S
ATOM 375 HN CYS 29 20.543 4.310 24.243 1.00
11.10 H
ATOM 376 HA CYS 29 18.349 3.517 22.611 1.00
11.08 H
ATOM 377 HB1 CYS 29 19.877 1.107 23.542 1.00
9.33 H
ATOM 378 HB2 CYS 29 20.395 1.900 22.055 1.00
10.78 H
ATOM 379 N GLU 30 17.233 3.370 24.819 1.00
13.52 N
ATOM 380 CA GLU 30 16.552 3.268 26.105 1.00
17.41 C
ATOM 381 C GLU 30 15.908 1.899 26.284 1.00
10.81 C
ATOM 382 O GLU 30 15.670 1.181 25.315 1.00
23.22 O
ATOM 383 CB GLU 30 15.491 4.362 26.210 1.00
37.83 C
ATOM 384 CG GLU 30 16.178 5.725 26.180
1.00108.13 C

```
ATOM    385  CD  GLU    30      16.947   5.950  27.475
1.00240.52           C
ATOM    386  OE1 GLU    30      17.767   6.853  27.506
1.00422.56           O
ATOM    387  OE2 GLU    30      16.705   5.216  28.420
1.00410.81           O1-
ATOM    388  HN  GLU    30      16.765   3.777  24.055  1.00
15.61        H
ATOM    389  HA  GLU    30      17.273   3.416  26.896  1.00
24.60        H
ATOM    390  HB1 GLU    30      14.949   4.253  27.135  1.00
72.67        H
ATOM    391  HB2 GLU    30      14.809   4.284  25.376  1.00
56.47        H
ATOM    392  HG1 GLU    30      15.436   6.501  26.064
1.00196.66           H
ATOM    393  HG2 GLU    30      16.864   5.758  25.346
1.00140.67           H
ATOM    394  N   LYS    31      15.631   1.541  27.540  1.00
11.96        N
ATOM    395  CA  LYS    31      15.010   0.254  27.859  1.00
11.40        C
ATOM    396  C   LYS    31      13.736   0.471  28.668  1.00
10.99        C
ATOM    397  O   LYS    31      13.671   1.365  29.512  1.00
12.86        O
ATOM    398  CB  LYS    31      15.980  -0.604  28.672  1.00
21.40        C
ATOM    399  CG  LYS    31      17.225  -0.905  27.834  1.00
57.81        C
ATOM    400  CD  LYS    31      18.186  -1.779  28.642
1.00115.52           C
ATOM    401  CE  LYS    31      19.423  -2.092  27.799
1.00250.23           C
ATOM    402  NZ  LYS    31      20.149  -0.828  27.492
1.00462.10           N1+
ATOM    403  HN  LYS    31      15.848   2.160  28.268  1.00
26.13        H
ATOM    404  HA  LYS    31      14.763  -0.270  26.948  1.00
13.87        H
ATOM    405  HB1 LYS    31      15.500  -1.532  28.943  1.00
37.63        H
ATOM    406  HB2 LYS    31      16.266  -0.072  29.566  1.00
47.19        H
ATOM    407  HG1 LYS    31      17.716   0.022  27.575
1.00126.32           H
ATOM    408  HG2 LYS    31      16.935  -1.424  26.932
1.00114.19           H
ATOM    409  HD1 LYS    31      17.693  -2.700  28.911
1.00198.45           H
ATOM    410  HD2 LYS    31      18.484  -1.254  29.538
1.00200.14           H
```

```
ATOM    411  HE1 LYS    31       19.120  -2.567  26.878
1.00374.64           H
ATOM    412  HE2 LYS    31       20.073  -2.758  28.347
1.00403.27           H
ATOM    413  HZ1 LYS    31       20.251  -0.267  28.362
1.00627.38           H
ATOM    414  HZ2 LYS    31       19.610  -0.283  26.788
1.00622.26           H
ATOM    415  HZ3 LYS    31       21.090  -1.051  27.114
1.00619.20           H
ATOM    416  N   THR    32       12.726  -0.353  28.407  1.00
15.51           N
ATOM    417  CA  THR    32       11.456  -0.242  29.124  1.00
22.44           C
ATOM    418  C   THR    32       11.522  -1.006  30.442  1.00
26.60           C
ATOM    419  O   THR    32       11.767  -2.212  30.463  1.00
57.43           O
ATOM    420  CB  THR    32       10.317  -0.799  28.265  1.00
56.28           C
ATOM    421  CG2 THR    32        9.002  -0.715  29.040
1.00102.70           C
ATOM    422  OG1 THR    32       10.213  -0.041  27.068
1.00111.17           O
ATOM    423  HN  THR    32       12.836  -1.049  27.726  1.00
18.79           H
ATOM    424  HA  THR    32       11.257   0.800  29.332  1.00
19.67           H
ATOM    425  HB  THR    32       10.520  -1.828  28.022  1.00
84.25           H
ATOM    426  HG1 THR    32        9.988  -0.644  26.355
1.00205.03           H
ATOM    427 HG21 THR    32        9.016  -1.423  29.856
1.00220.71           H
ATOM    428 HG22 THR    32        8.180  -0.947  28.381
1.00174.67           H
ATOM    429 HG23 THR    32        8.878   0.284  29.433
1.00217.44           H
ATOM    430  N   THR    33       11.296  -0.292  31.541  1.00
22.47           N
ATOM    431  CA  THR    33       11.327  -0.903  32.867  1.00
41.60           C
ATOM    432  C   THR    33       10.458  -0.134  33.825  1.00
38.03           C
ATOM    433  O   THR    33       10.912   0.785  34.505  1.00
52.73           O
ATOM    434  CB  THR    33       12.763  -0.960  33.398  1.00
63.25           C
ATOM    435  CG2 THR    33       13.581  -1.956  32.574
1.00121.65           C
ATOM    436  OG1 THR    33       13.351   0.329  33.305  1.00
96.20           O
```

```
ATOM    437  HN  THR    33      11.106   0.666  31.460  1.00
23.82          H
ATOM    438  HA  THR    33      10.930  -1.899  32.814  1.00
67.12          H
ATOM    439  HB  THR    33      12.751  -1.277  34.430
1.00107.59          H
ATOM    440  HG1 THR    33      13.741   0.415  32.432
1.00176.27          H
ATOM    441 HG21 THR    33      13.020  -2.870  32.450
1.00255.23          H
ATOM    442 HG22 THR    33      14.507  -2.171  33.087
1.00245.37          H
ATOM    443 HG23 THR    33      13.796  -1.531  31.606
1.00185.78          H
ATOM    444  N   GLY    34       9.201  -0.539  33.885  1.00
40.93          N
ATOM    445  CA  GLY    34       8.268   0.097  34.779  1.00
45.59          C
ATOM    446  C   GLY    34       7.885   1.482  34.272  1.00
40.97          C
ATOM    447  O   GLY    34       6.722   1.883  34.343  1.00
82.17          O
ATOM    448  HN  GLY    34       8.904  -1.289  33.328  1.00
54.00          H
ATOM    449  HA1 GLY    34       8.741   0.184  35.736  1.00
54.41          H
ATOM    450  HA2 GLY    34       7.381  -0.514  34.868  1.00
62.09          H
ATOM    451  N   ASN    35       8.873   2.209  33.751  1.00
32.51          N
ATOM    452  CA  ASN    35       8.644   3.556  33.218  1.00
33.20          C
ATOM    453  C   ASN    35       9.343   3.716  31.873  1.00
23.17          C
ATOM    454  O   ASN    35      10.465   3.246  31.685  1.00
27.01          O
ATOM    455  CB  ASN    35       9.175   4.602  34.198  1.00
54.51          C
ATOM    456  CG  ASN    35       8.468   4.467  35.542  1.00
80.73          C
ATOM    457  ND2 ASN    35       9.167   4.498  36.643
1.00220.46          N
ATOM    458  OD1 ASN    35       7.245   4.327  35.589
1.00117.80          O
ATOM    459  HN  ASN    35       9.778   1.831  33.718  1.00
51.15          H
ATOM    460  HA  ASN    35       7.583   3.715  33.079  1.00
47.21          H
ATOM    461  HB1 ASN    35       8.995   5.589  33.797  1.00
68.31          H
ATOM    462  HB2 ASN    35      10.238   4.459  34.334  1.00
57.75          H
```

```
ATOM    463 HD21 ASN    35      10.139   4.608  36.602
1.00405.39           H
ATOM    464 HD22 ASN    35       8.719   4.412  37.511
1.00245.64           H
ATOM    465  N   PHE    36       8.671   4.377  30.937  1.00
35.72           N
ATOM    466  CA  PHE    36       9.222   4.592  29.610  1.00
33.41           C
ATOM    467  C   PHE    36      10.094   5.843  29.577  1.00
28.51           C
ATOM    468  O   PHE    36       9.864   6.792  30.327  1.00
45.23           O
ATOM    469  CB  PHE    36       8.075   4.742  28.617  1.00
67.53           C
ATOM    470  CG  PHE    36       7.301   3.447  28.550  1.00
86.71           C
ATOM    471  CD1 PHE    36       6.227   3.234  29.422
1.00106.51           C
ATOM    472  CD2 PHE    36       7.650   2.464  27.615  1.00
99.48           C
ATOM    473  CE1 PHE    36       5.503   2.037  29.361
1.00134.48           C
ATOM    474  CE2 PHE    36       6.926   1.268  27.555
1.00134.27           C
ATOM    475  CZ  PHE    36       5.852   1.054  28.428
1.00149.48           C
ATOM    476  HN  PHE    36       7.779   4.723  31.139  1.00
62.87           H
ATOM    477  HA  PHE    36       9.819   3.737  29.325  1.00
28.53           H
ATOM    478  HB1 PHE    36       8.470   4.975  27.646  1.00
73.56           H
ATOM    479  HB2 PHE    36       7.420   5.538  28.941  1.00
88.42           H
ATOM    480  HD1 PHE    36       5.959   3.992  30.144
1.00110.20           H
ATOM    481  HD2 PHE    36       8.480   2.627  26.943  1.00
93.07           H
ATOM    482  HE1 PHE    36       4.675   1.872  30.034
1.00155.06           H
ATOM    483  HE2 PHE    36       7.195   0.510  26.834
1.00159.46           H
ATOM    484  HZ  PHE    36       5.294   0.132  28.380
1.00183.25           H
ATOM    485  N   THR    37      11.094   5.839  28.699  1.00
20.06           N
ATOM    486  CA  THR    37      11.997   6.975  28.562  1.00
27.18           C
ATOM    487  C   THR    37      12.599   7.001  27.161  1.00
41.87           C
ATOM    488  O   THR    37      13.225   6.036  26.723
1.00169.86           O
```

```
ATOM    489  CB  THR    37      13.114   6.888  29.608  1.00
26.52           C
ATOM    490  CG2 THR    37      13.516   5.426  29.820  1.00
75.69           C
ATOM    491  OG1 THR    37      14.238   7.633  29.162  1.00
81.95           O
ATOM    492  HN  THR    37      11.224   5.057  28.125  1.00
18.47           H
ATOM    493  HA  THR    37      11.441   7.889  28.718  1.00
38.80           H
ATOM    494  HB  THR    37      12.763   7.297  30.545  1.00
81.60           H
ATOM    495  HG1 THR    37      14.712   7.949  29.934
1.00188.80           H
ATOM    496 HG21 THR    37      12.799   4.948  30.471
1.00196.98           H
ATOM    497 HG22 THR    37      14.496   5.384  30.274
1.00196.23           H
ATOM    498 HG23 THR    37      13.537   4.914  28.870
1.00168.92           H
ATOM    499  N   GLU    38      12.389   8.109  26.462  1.00
26.53           N
ATOM    500  CA  GLU    38      12.897   8.266  25.101  1.00
30.29           C
ATOM    501  C   GLU    38      14.367   8.680  25.101  1.00
25.39           C
ATOM    502  O   GLU    38      14.828   9.379  26.004  1.00
51.60           O
ATOM    503  CB  GLU    38      12.071   9.319  24.359  1.00
54.82           C
ATOM    504  CG  GLU    38      12.527   9.400  22.900
1.00165.84           C
ATOM    505  CD  GLU    38      11.654  10.389  22.135
1.00292.92           C
ATOM    506  OE1 GLU    38      10.759  10.953  22.744
1.00414.19           O
ATOM    507  OE2 GLU    38      11.894  10.569  20.953
1.00537.65           O1-
ATOM    508  HN  GLU    38      11.873   8.835  26.867  1.00
84.50           H
ATOM    509  HA  GLU    38      12.801   7.323  24.581  1.00
32.15           H
ATOM    510  HB1 GLU    38      12.206  10.279  24.830
1.00144.82           H
ATOM    511  HB2 GLU    38      11.026   9.043  24.394
1.00106.49           H
ATOM    512  HG1 GLU    38      12.447   8.425  22.444
1.00303.31           H
ATOM    513  HG2 GLU    38      13.556   9.729  22.864
1.00323.99           H
ATOM    514  N   CYS    39      15.092   8.248  24.071  1.00
15.84           N
```

```
ATOM    515  CA  CYS    39       16.513   8.576  23.934  1.00
14.30          C
ATOM    516  C   CYS    39       16.758  10.041  24.324  1.00
22.31          C
ATOM    517  O   CYS    39       15.818  10.830  24.412  1.00
32.92          O
ATOM    518  CB  CYS    39       16.982   8.313  22.476  1.00
18.08          C
ATOM    519  SG  CYS    39       15.575   7.826  21.450  1.00
17.41          S
ATOM    520  HN  CYS    39       14.661   7.700  23.383  1.00
24.87          H
ATOM    521  HA  CYS    39       17.076   7.944  24.604  1.00
14.60          H
ATOM    522  HB1 CYS    39       17.707   7.511  22.467  1.00
27.71          H
ATOM    523  HB2 CYS    39       17.430   9.201  22.055  1.00
34.91          H
ATOM    524  N   PRO    40       17.993  10.407  24.558  1.00
27.27          N
ATOM    525  CA  PRO    40       18.353  11.800  24.947  1.00
47.66          C
ATOM    526  C   PRO    40       18.310  12.751  23.753  1.00
71.54          C
ATOM    527  O   PRO    40       18.683  12.387  22.640  1.00
87.70          O
ATOM    528  CB  PRO    40       19.777  11.653  25.499  1.00
53.85          C
ATOM    529  CG  PRO    40       20.352  10.500  24.743  1.00
46.04          C
ATOM    530  CD  PRO    40       19.184   9.543  24.472  1.00
25.70          C
ATOM    531  HA  PRO    40       17.697  12.151  25.727  1.00
58.04          H
ATOM    532  HB1 PRO    40       19.748  11.428  26.555  1.00
67.93          H
ATOM    533  HB2 PRO    40       20.349  12.556  25.320  1.00
70.99          H
ATOM    534  HG1 PRO    40       21.106  10.000  25.334  1.00
65.91          H
ATOM    535  HG2 PRO    40       20.780  10.847  23.809  1.00
54.99          H
ATOM    536  HD1 PRO    40       19.142   8.771  25.223  1.00
24.22          H
ATOM    537  HD2 PRO    40       19.269   9.115  23.485  1.00
26.94          H
ATOM    538  N   GLY    41       17.857  13.972  24.002
1.00105.24          N
ATOM    539  CA  GLY    41       17.771  14.976  22.950
1.00147.55          C
ATOM    540  C   GLY    41       19.154  15.306  22.400
1.00161.74          C
```

ATOM 541 O GLY 41 19.331 15.486 21.196
1.00244.37 O
ATOM 542 HN GLY 41 17.578 14.200 24.910
1.00121.79 H
ATOM 543 HA1 GLY 41 17.326 15.875 23.350
1.00184.40 H
ATOM 544 HA2 GLY 41 17.153 14.598 22.150
1.00157.34 H
ATOM 545 N LEU 42 20.131 15.385 23.300
1.00162.48 N
ATOM 546 CA LEU 42 21.513 15.696 22.919
1.00191.48 C
ATOM 547 C LEU 42 22.426 14.512 23.225
1.00166.66 C
ATOM 548 O LEU 42 22.195 13.767 24.178
1.00251.38 O
ATOM 549 CB LEU 42 22.001 16.925 23.693
1.00307.99 C
ATOM 550 CG LEU 42 21.051 18.105 23.454
1.00452.28 C
ATOM 551 CD1 LEU 42 21.521 19.304 24.284
1.00681.24 C
ATOM 552 CD2 LEU 42 21.042 18.482 21.963
1.00527.46 C
ATOM 553 HN LEU 42 19.920 15.232 24.243
1.00195.47 H
ATOM 554 HA LEU 42 21.564 15.907 21.861
1.00229.77 H
ATOM 555 HB1 LEU 42 22.991 17.190 23.355
1.00342.49 H
ATOM 556 HB2 LEU 42 22.031 16.695 24.748
1.00335.33 H
ATOM 557 HG LEU 42 20.053 17.827 23.763
1.00427.79 H
ATOM 558 HD11 LEU 42 21.408 19.080 25.334
1.00909.71 H
ATOM 559 HD12 LEU 42 20.926 20.169 24.034
1.00706.80 H
ATOM 560 HD13 LEU 42 22.560 19.506 24.068
1.00858.82 H
ATOM 561 HD21 LEU 42 20.361 17.833 21.432
1.00631.69 H
ATOM 562 HD22 LEU 42 22.035 18.375 21.552
1.00628.06 H
ATOM 563 HD23 LEU 42 20.716 19.507 21.848
1.00656.75 H
ATOM 564 N THR 43 23.462 14.342 22.409
1.00178.52 N
ATOM 565 CA THR 43 24.399 13.243 22.602
1.00240.84 C
ATOM 566 C THR 43 25.210 13.452 23.887
1.00372.31 C

```
ATOM    567  O   THR    43       25.466  14.592  24.277
1.00500.13          O
ATOM    568  CB  THR    43       25.345  13.168  21.398
1.00354.13          C
ATOM    569  CG2 THR    43       24.525  13.013  20.116
1.00463.04          C
ATOM    570  OG1 THR    43       26.115  14.360  21.329
1.00508.50          O
ATOM    571  HN  THR    43       23.595  14.966  21.665
1.00229.59          H
ATOM    572  HA  THR    43       23.842  12.323  22.668
1.00249.90          H
ATOM    573  HB  THR    43       26.004  12.323  21.501
1.00481.58          H
ATOM    574  HG1 THR    43       25.774  14.972  21.987
1.00624.31          H
ATOM    575 HG21 THR    43       23.763  12.261  20.265
1.00650.95          H
ATOM    576 HG22 THR    43       25.174  12.711  19.307
1.00580.73          H
ATOM    577 HG23 THR    43       24.057  13.955  19.872
1.00571.99          H
ATOM    578  N   PRO    44       25.627  12.393  24.548
1.00488.39          N
ATOM    579  CA  PRO    44       26.429  12.503  25.804
1.00760.13          C
ATOM    580  C   PRO    44       27.867  12.935  25.522
1.00735.36          C
ATOM    581  O   PRO    44       28.702  12.981  26.425
1.00999.99          O
ATOM    582  CB  PRO    44       26.371  11.087  26.390
1.00999.99          C
ATOM    583  CG  PRO    44       26.223  10.194  25.203
1.00856.94          C
ATOM    584  CD  PRO    44       25.385  10.979  24.188
1.00548.09          C
ATOM    585  HA  PRO    44       25.962  13.196  26.485
1.00939.00          H
ATOM    586  HB1 PRO    44       25.516  10.983  27.042
1.00999.99          H
ATOM    587  HB2 PRO    44       27.283  10.858  26.931
1.00999.99          H
ATOM    588  HG1 PRO    44       25.707   9.284  25.476
1.00999.99          H
ATOM    589  HG2 PRO    44       27.196   9.960  24.788
1.00853.53          H
ATOM    590  HD1 PRO    44       24.337  10.742  24.296
1.00602.45          H
ATOM    591  HD2 PRO    44       25.723  10.773  23.183
1.00463.32          H
ATOM    592  N   ILE    45       28.148  13.246  24.260
1.00518.33          N
```

ATOM 593 CA ILE 45 29.487 13.665 23.865
1.00582.79 C
ATOM 594 C ILE 45 29.892 14.923 24.626
1.00795.93 C
ATOM 595 O ILE 45 31.010 15.024 25.130
1.00898.75 O
ATOM 596 CB ILE 45 29.520 13.940 22.359
1.00474.41 C
ATOM 597 CG1 ILE 45 29.322 12.627 21.598
1.00516.90 C
ATOM 598 CG2 ILE 45 30.872 14.546 21.972
1.00760.75 C
ATOM 599 CD1 ILE 45 29.044 12.925 20.122
1.00566.81 C
ATOM 600 HN ILE 45 27.442 13.186 23.581
1.00395.72 H
ATOM 601 HA ILE 45 30.181 12.877 24.093
1.00711.06 H
ATOM 602 HB ILE 45 28.729 14.631 22.103
1.00477.81 H
ATOM 603 HG11 ILE 45 28.486 12.089 22.020
1.00624.28 H
ATOM 604 HG12 ILE 45 30.216 12.025 21.681
1.00744.84 H
ATOM 605 HG21 ILE 45 30.915 15.574 22.301
1.00920.57 H
ATOM 606 HG22 ILE 45 30.993 14.507 20.899
1.00999.99 H
ATOM 607 HG23 ILE 45 31.666 13.986 22.445
1.00865.46 H
ATOM 608 HD11 ILE 45 28.764 12.012 19.617
1.00658.89 H
ATOM 609 HD12 ILE 45 29.932 13.333 19.665
1.00734.30 H
ATOM 610 HD13 ILE 45 28.238 13.640 20.044
1.00675.84 H
ATOM 611 N ALA 46 28.976 15.875 24.699
1.00999.99 N
ATOM 612 CA ALA 46 29.241 17.127 25.399
1.00999.99 C
ATOM 613 C ALA 46 30.530 17.763 24.889
1.00999.99 C
ATOM 614 CB ALA 46 29.358 16.869 26.902
1.00999.99 C
ATOM 615 OT1 ALA 46 31.549 17.595 25.538
1.00999.99 O
ATOM 616 OT2 ALA 46 30.479 18.409 23.855
1.00999.99 O
ATOM 617 HN ALA 46 28.104 15.733 24.276
1.00999.99 H
ATOM 618 HA ALA 46 28.422 17.807 25.224
1.00999.99 H

```
ATOM    619  HB1 ALA    46       28.444  16.424  27.263
1.00999.99           H
ATOM    620  HB2 ALA    46       29.530  17.805  27.416
1.00999.99           H
ATOM    621  HB3 ALA    46       30.184  16.200  27.090
1.00999.99           H
ENDMDL
TER
MODEL       16
ATOM      1  N   GLY     1       27.053  -1.121  13.325
1.00999.99           N
ATOM      2  CA  GLY     1       25.792  -1.801  13.736
1.00999.99           C
ATOM      3  C   GLY     1       24.649  -0.791  13.747
1.00999.99           C
ATOM      4  O   GLY     1       24.244  -0.309  14.804
1.00999.99           O
ATOM      5  HA1 GLY     1       25.911  -2.215  14.724
1.00999.99           H
ATOM      6  HA2 GLY     1       25.567  -2.593  13.037
1.00999.99           H
ATOM      7  HT1 GLY     1       26.929  -0.697  12.385
1.00999.99           H
ATOM      8  HT2 GLY     1       27.826  -1.817  13.292
1.00999.99           H
ATOM      9  HT3 GLY     1       27.285  -0.376  14.011
1.00999.99           H
ATOM     10  N   LEU     2       24.133  -0.476  12.564
1.00999.99           N
ATOM     11  CA  LEU     2       23.035   0.478  12.450
1.00895.59           C
ATOM     12  C   LEU     2       21.737  -0.142  12.958
1.00532.27           C
ATOM     13  O   LEU     2       21.533  -1.350  12.851
1.00625.21           O
ATOM     14  CB  LEU     2       22.859   0.900  10.989
1.00999.99           C
ATOM     15  CG  LEU     2       24.165   1.500  10.452
1.00999.99           C
ATOM     16  CD1 LEU     2       23.990   1.834   8.967
1.00999.99           C
ATOM     17  CD2 LEU     2       24.520   2.779  11.232
1.00999.99           C
ATOM     18  HN  LEU     2       24.493  -0.892  11.754
1.00999.99           H
ATOM     19  HA  LEU     2       23.260   1.351  13.043
1.00890.87           H
ATOM     20  HB1 LEU     2       22.073   1.637  10.919
1.00924.07           H
ATOM     21  HB2 LEU     2       22.593   0.034  10.397
1.00999.99           H
ATOM     22  HG  LEU     2       24.962   0.777  10.562
```

```
1.00999.99           H
ATOM     23 HD11 LEU     2       24.947   2.090   8.540
1.00999.99           H
ATOM     24 HD12 LEU     2       23.316   2.671   8.864
1.00999.99           H
ATOM     25 HD13 LEU     2       23.581   0.977   8.451
1.00999.99           H
ATOM     26 HD21 LEU     2       25.156   3.409  10.627
1.00999.99           H
ATOM     27 HD22 LEU     2       25.044   2.513  12.138
1.00999.99           H
ATOM     28 HD23 LEU     2       23.616   3.317  11.482
1.00999.99           H
ATOM     29  N   CYS     3       20.861   0.694  13.509
1.00271.28           N
ATOM     30  CA  CYS     3       19.586   0.211  14.028
1.00104.50           C
ATOM     31  C   CYS     3       18.764  -0.439  12.917  1.00
90.98          C
ATOM     32  O   CYS     3       18.627   0.115  11.827
1.00207.28           O
ATOM     33  CB  CYS     3       18.792   1.372  14.640  1.00
37.83          C
ATOM     34  SG  CYS     3       19.374   1.695  16.321  1.00
81.56          S
ATOM     35  HN  CYS     3       21.076   1.650  13.568
1.00296.55           H
ATOM     36  HA  CYS     3       19.779  -0.520  14.797
1.00166.45           H
ATOM     37  HB1 CYS     3       17.742   1.118  14.673  1.00
40.38          H
ATOM     38  HB2 CYS     3       18.928   2.258  14.038  1.00
98.45          H
ATOM     39  N   SER     4       18.206  -1.609  13.211  1.00
85.31          N
ATOM     40  CA  SER     4       17.385  -2.319  12.238  1.00
92.94          C
ATOM     41  C   SER     4       16.140  -1.507  11.925  1.00
67.37          C
ATOM     42  O   SER     4       15.711  -1.410  10.776
1.00102.28           O
ATOM     43  CB  SER     4       16.946  -3.666  12.806
1.00123.83           C
ATOM     44  OG  SER     4       16.277  -4.405  11.792
1.00178.89           O
ATOM     45  HN  SER     4       18.341  -1.996  14.101
1.00163.03           H
ATOM     46  HA  SER     4       17.950  -2.480  11.334
1.00129.78           H
ATOM     47  HB1 SER     4       16.269  -3.491  13.638
1.00105.26           H
ATOM     48  HB2 SER     4       17.804  -4.219  13.149
```

```
1.00156.87            H
ATOM     49  HG  SER     4      15.540  -4.867  12.198
1.00218.16            H
ATOM     50  N   GLU     5      15.552  -0.948  12.979  1.00
41.67           N
ATOM     51  CA  GLU     5      14.332  -0.160  12.857  1.00
41.21           C
ATOM     52  C   GLU     5      14.375   1.046  13.793  1.00
41.92           C
ATOM     53  O   GLU     5      15.435   1.409  14.302  1.00
74.72           O
ATOM     54  CB  GLU     5      13.132  -1.039  13.199  1.00
49.24           C
ATOM     55  CG  GLU     5      13.301  -1.581  14.611  1.00
55.48           C
ATOM     56  CD  GLU     5      12.257  -2.658  14.887  1.00
92.06           C
ATOM     57  OE1 GLU     5      12.260  -3.192  15.985
1.00206.49            O
ATOM     58  OE2 GLU     5      11.470  -2.935  13.996
1.00197.24            O1-
ATOM     59  HN  GLU     5      15.941  -1.085  13.868  1.00
45.52           H
ATOM     60  HA  GLU     5      14.229   0.186  11.847  1.00
60.63           H
ATOM     61  HB1 GLU     5      13.078  -1.861  12.503  1.00
61.59           H
ATOM     62  HB2 GLU     5      12.233  -0.457  13.141  1.00
63.66           H
ATOM     63  HG1 GLU     5      13.182  -0.777  15.322  1.00
58.03           H
ATOM     64  HG2 GLU     5      14.287  -2.001  14.706  1.00
57.77           H
ATOM     65  N   ASN     6      13.220   1.665  14.013  1.00
50.73           N
ATOM     66  CA  ASN     6      13.146   2.833  14.886  1.00
71.04           C
ATOM     67  C   ASN     6      13.594   2.478  16.300  1.00
65.17           C
ATOM     68  O   ASN     6      14.288   3.258  16.953  1.00
94.86           O
ATOM     69  CB  ASN     6      11.712   3.365  14.924  1.00
94.83           C
ATOM     70  CG  ASN     6      11.364   4.024  13.594
1.00160.94            C
ATOM     71  ND2 ASN     6      10.112   4.181  13.262
1.00244.88            N
ATOM     72  OD1 ASN     6      12.256   4.407  12.837
1.00219.73            O
ATOM     73  HN  ASN     6      12.405   1.336  13.577  1.00
72.81           H
ATOM     74  HA  ASN     6      13.793   3.605  14.496  1.00
```

```
96.02           H
ATOM     75  HB1 ASN     6       11.622   4.093  15.717
1.00111.09           H
ATOM     76  HB2 ASN     6       11.031   2.548  15.108  1.00
98.13           H
ATOM     77 HD21 ASN     6        9.402   3.875  13.866
1.00272.43           H
ATOM     78 HD22 ASN     6        9.880   4.603  12.408
1.00336.78           H
ATOM     79  N   GLY     7       13.197   1.296  16.767  1.00
51.80           N
ATOM     80  CA  GLY     7       13.563   0.833  18.110  1.00
71.47           C
ATOM     81  C   GLY     7       14.284  -0.507  18.041  1.00
41.04           C
ATOM     82  O   GLY     7       13.667  -1.565  18.163  1.00
44.54           O
ATOM     83  HN  GLY     7       12.648   0.717  16.196  1.00
45.49           H
ATOM     84  HA1 GLY     7       12.668   0.719  18.698
1.00103.52           H
ATOM     85  HA2 GLY     7       14.210   1.560  18.585
1.00108.67           H
ATOM     86  N   ASP     8       15.597  -0.455  17.842  1.00
27.00           N
ATOM     87  CA  ASP     8       16.397  -1.671  17.754  1.00
14.15           C
ATOM     88  C   ASP     8       16.346  -2.454  19.061  1.00
9.37           C
ATOM     89  O   ASP     8       16.206  -3.678  19.056  1.00
14.61           O
ATOM     90  CB  ASP     8       17.849  -1.317  17.419  1.00
15.06           C
ATOM     91  CG  ASP     8       18.673  -2.592  17.272  1.00
21.22           C
ATOM     92  OD1 ASP     8       19.824  -2.488  16.879
1.00119.56           O
ATOM     93  OD2 ASP     8       18.143  -3.655  17.555
1.00133.27           O1-
ATOM     94  HN  ASP     8       16.033   0.417  17.751  1.00
35.63           H
ATOM     95  HA  ASP     8       16.002  -2.288  16.968  1.00
20.92           H
ATOM     96  HB1 ASP     8       18.263  -0.713  18.210  1.00
29.39           H
ATOM     97  HB2 ASP     8       17.876  -0.766  16.492  1.00
44.14           H
ATOM     98  N   CYS     9       16.460  -1.745  20.177  1.00
5.93           N
ATOM     99  CA  CYS     9       16.426  -2.392  21.485  1.00
7.54           C
ATOM    100  C   CYS     9       15.083  -3.081  21.699  1.00
```

14.60 C
ATOM 101 O CYS 9 15.016 -4.210 22.187 1.00
25.78 O
ATOM 102 CB CYS 9 16.643 -1.361 22.581 1.00
6.63 C
ATOM 103 SG CYS 9 18.348 -0.793 22.505 1.00
10.91 S
ATOM 104 HN CYS 9 16.570 -0.774 20.118 1.00
6.51 H
ATOM 105 HA CYS 9 17.219 -3.116 21.539 1.00
11.12 H
ATOM 106 HB1 CYS 9 16.460 -1.811 23.543 1.00
11.28 H
ATOM 107 HB2 CYS 9 15.975 -0.525 22.436 1.00
5.04 H
ATOM 108 N ALA 10 14.019 -2.384 21.320 1.00
16.26 N
ATOM 109 CA ALA 10 12.665 -2.910 21.457 1.00
32.04 C
ATOM 110 C ALA 10 11.692 -2.052 20.651 1.00
45.47 C
ATOM 111 O ALA 10 12.036 -0.952 20.221
1.00119.04 O
ATOM 112 CB ALA 10 12.250 -2.935 22.934 1.00
30.10 C
ATOM 113 HN ALA 10 14.149 -1.493 20.936 1.00
12.81 H
ATOM 114 HA ALA 10 12.644 -3.918 21.070 1.00
47.14 H
ATOM 115 HB1 ALA 10 13.104 -3.190 23.543 1.00
84.37 H
ATOM 116 HB2 ALA 10 11.473 -3.672 23.078
1.00118.20 H
ATOM 117 HB3 ALA 10 11.879 -1.963 23.225
1.00117.32 H
ATOM 118 N ALA 11 10.484 -2.560 20.443 1.00
30.62 N
ATOM 119 CA ALA 11 9.482 -1.823 19.678 1.00
35.24 C
ATOM 120 C ALA 11 9.151 -0.488 20.344 1.00
22.78 C
ATOM 121 O ALA 11 9.009 0.530 19.669 1.00
53.33 O
ATOM 122 CB ALA 11 8.208 -2.660 19.551 1.00
60.49 C
ATOM 123 HN ALA 11 10.262 -3.444 20.805 1.00
50.37 H
ATOM 124 HA ALA 11 9.870 -1.633 18.689 1.00
41.70 H
ATOM 125 HB1 ALA 11 7.412 -2.044 19.155
1.00157.06 H
ATOM 126 HB2 ALA 11 7.924 -3.032 20.523

```
1.00148.83           H
ATOM    127  HB3 ALA    11        8.387  -3.489  18.884
1.00137.06           H
ATOM    128  N   ASP    12        9.023  -0.501  21.669  1.00
18.98           N
ATOM    129  CA  ASP    12        8.700   0.719  22.410  1.00
31.05           C
ATOM    130  C   ASP    12        9.953   1.540  22.699  1.00
22.98           C
ATOM    131  O   ASP    12        9.903   2.769  22.757  1.00
37.14           O
ATOM    132  CB  ASP    12        8.017   0.355  23.730  1.00
48.35           C
ATOM    133  CG  ASP    12        6.632  -0.222  23.459
1.00104.83           C
ATOM    134  OD1 ASP    12        6.153  -0.063  22.349
1.00258.73           O
ATOM    135  OD2 ASP    12        6.071  -0.813  24.367
1.00213.46           O1-
ATOM    136  HN  ASP    12        9.142  -1.342  22.156  1.00
36.19           H
ATOM    137  HA  ASP    12        8.018   1.318  21.824  1.00
49.76           H
ATOM    138  HB1 ASP    12        7.922   1.240  24.341  1.00
95.71           H
ATOM    139  HB2 ASP    12        8.615  -0.379  24.252  1.00
54.37           H
ATOM    140  N   GLU    13       11.076   0.855  22.890  1.00
17.60           N
ATOM    141  CA  GLU    13       12.337   1.529  23.184  1.00
12.01           C
ATOM    142  C   GLU    13       12.920   2.166  21.923  1.00
10.07           C
ATOM    143  O   GLU    13       12.559   1.792  20.807  1.00
12.81           O
ATOM    144  CB  GLU    13       13.329   0.524  23.766  1.00
11.74           C
ATOM    145  CG  GLU    13       12.764  -0.037  25.075  1.00
13.49           C
ATOM    146  CD  GLU    13       13.595  -1.224  25.542
1.00142.36           C
ATOM    147  OE1 GLU    13       14.648  -1.447  24.969
1.00339.06           O
ATOM    148  OE2 GLU    13       13.165  -1.898  26.464
1.00335.28           O1-
ATOM    149  HN  GLU    13       11.055  -0.124  22.838  1.00
28.36           H
ATOM    150  HA  GLU    13       12.158   2.302  23.917  1.00
13.06           H
ATOM    151  HB1 GLU    13       14.268   1.017  23.963  1.00
13.76           H
ATOM    152  HB2 GLU    13       13.485  -0.280  23.062  1.00
```

```
9.94           H
ATOM    153  HG1 GLU    13       11.745  -0.353  24.917  1.00
66.02           H
ATOM    154  HG2 GLU    13       12.785   0.734  25.831  1.00
56.23           H
ATOM    155  N   CYS    14       13.824   3.131  22.110  1.00
9.12           N
ATOM    156  CA  CYS    14       14.461   3.823  20.983  1.00
10.69           C
ATOM    157  C   CYS    14       15.955   3.542  20.974  1.00
9.56           C
ATOM    158  O   CYS    14       16.562   3.340  22.025  1.00
13.62           O
ATOM    159  CB  CYS    14       14.232   5.332  21.088  1.00
13.96           C
ATOM    160  SG  CYS    14       15.130   6.161  19.750  1.00
42.96           S
ATOM    161  HN  CYS    14       14.075   3.383  23.024  1.00
9.76           H
ATOM    162  HA  CYS    14       14.034   3.472  20.053  1.00
15.54           H
ATOM    163  HB1 CYS    14       14.592   5.688  22.042  1.00
50.72           H
ATOM    164  HB2 CYS    14       13.177   5.544  21.001  1.00
46.13           H
ATOM    165  N   CYS    15       16.548   3.537  19.782  1.00
9.60           N
ATOM    166  CA  CYS    15       17.979   3.283  19.641  1.00
9.28           C
ATOM    167  C   CYS    15       18.582   4.277  18.665  1.00
9.92           C
ATOM    168  O   CYS    15       17.892   4.791  17.783  1.00
13.31           O
ATOM    169  CB  CYS    15       18.209   1.865  19.113  1.00
12.90           C
ATOM    170  SG  CYS    15       17.663   1.777  17.391  1.00
39.11           S
ATOM    171  HN  CYS    15       16.014   3.712  18.979  1.00
13.12           H
ATOM    172  HA  CYS    15       18.470   3.386  20.600  1.00
8.26           H
ATOM    173  HB1 CYS    15       17.642   1.163  19.704  1.00
24.82           H
ATOM    174  HB2 CYS    15       19.259   1.623  19.172  1.00
27.31           H
ATOM    175  N   VAL    16       19.867   4.545  18.821  1.00
9.56           N
ATOM    176  CA  VAL    16       20.537   5.481  17.936  1.00
11.99           C
ATOM    177  C   VAL    16       22.049   5.364  18.081  1.00
7.73           C
ATOM    178  O   VAL    16       22.601   5.597  19.156  1.00
```

```
8.83           O
ATOM    179  CB  VAL    16       20.072   6.905  18.261  1.00
18.37           C
ATOM    180  CG1 VAL    16       20.647   7.360  19.609  1.00
39.61           C
ATOM    181  CG2 VAL    16       20.529   7.851  17.150
1.00115.21           C
ATOM    182  HN  VAL    16       20.372   4.108  19.540  1.00
9.76           H
ATOM    183  HA  VAL    16       20.265   5.253  16.917  1.00
17.27           H
ATOM    184  HB  VAL    16       18.992   6.917  18.315  1.00
52.63           H
ATOM    185 HG11 VAL    16       20.642   6.531  20.305
1.00128.07           H
ATOM    186 HG12 VAL    16       20.041   8.160  20.006
1.00154.13           H
ATOM    187 HG13 VAL    16       21.660   7.710  19.475
1.00135.74           H
ATOM    188 HG21 VAL    16       21.567   7.663  16.922
1.00229.05           H
ATOM    189 HG22 VAL    16       20.410   8.873  17.476
1.00261.82           H
ATOM    190 HG23 VAL    16       19.929   7.681  16.268
1.00210.07           H
ATOM    191  N   ASP    17       22.718   4.989  16.989  1.00
14.34           N
ATOM    192  CA  ASP    17       24.175   4.835  17.001  1.00
11.90           C
ATOM    193  C   ASP    17       24.851   6.017  16.317  1.00
13.15           C
ATOM    194  O   ASP    17       24.635   6.267  15.131  1.00
25.96           O
ATOM    195  CB  ASP    17       24.567   3.543  16.283  1.00
20.80           C
ATOM    196  CG  ASP    17       24.157   2.337  17.120  1.00
28.72           C
ATOM    197  OD1 ASP    17       23.837   2.529  18.282
1.00135.42           O
ATOM    198  OD2 ASP    17       24.169   1.239  16.589
1.00114.17           O1-
ATOM    199  HN  ASP    17       22.223   4.808  16.163  1.00
28.29           H
ATOM    200  HA  ASP    17       24.522   4.778  18.024  1.00
10.49           H
ATOM    201  HB1 ASP    17       25.638   3.527  16.135  1.00
22.42           H
ATOM    202  HB2 ASP    17       24.071   3.501  15.325  1.00
29.52           H
ATOM    203  N   THR    18       25.684   6.728  17.072  1.00
11.55           N
ATOM    204  CA  THR    18       26.418   7.879  16.543  1.00
```

```
17.10           C
ATOM    205  C   THR    18       27.886   7.517  16.367  1.00
10.63           C
ATOM    206  O   THR    18       28.347   6.502  16.886  1.00
6.49           O
ATOM    207  CB  THR    18       26.294   9.071  17.495  1.00
28.52           C
ATOM    208  CG2 THR    18       24.914   9.708  17.358  1.00
45.38           C
ATOM    209  OG1 THR    18       26.484   8.627  18.829  1.00
26.49           O
ATOM    210  HN  THR    18       25.820   6.467  18.007  1.00
13.96           H
ATOM    211  HA  THR    18       26.011   8.158  15.580  1.00
26.43           H
ATOM    212  HB  THR    18       27.048   9.807  17.254  1.00
37.43           H
ATOM    213  HG1 THR    18       25.815   9.043  19.378  1.00
71.62           H
ATOM    214 HG21 THR    18       24.746  10.383  18.184
1.00100.44           H
ATOM    215 HG22 THR    18       24.158   8.937  17.362
1.00129.42           H
ATOM    216 HG23 THR    18       24.864  10.258  16.429
1.00156.21           H
ATOM    217  N   VAL    19       28.612   8.360  15.641  1.00
16.32           N
ATOM    218  CA  VAL    19       30.018   8.149  15.398  1.00
13.34           C
ATOM    219  C   VAL    19       30.535   9.250  14.496  1.00
25.88           C
ATOM    220  O   VAL    19       30.072   9.446  13.372  1.00
42.11           O
ATOM    221  CB  VAL    19       30.283   6.780  14.770  1.00
15.48           C
ATOM    222  CG1 VAL    19       29.327   6.544  13.597  1.00
29.06           C
ATOM    223  CG2 VAL    19       31.734   6.731  14.271  1.00
20.67           C
ATOM    224  HN  VAL    19       28.197   9.160  15.269  1.00
26.89           H
ATOM    225  HA  VAL    19       30.542   8.203  16.342  1.00
8.08           H
ATOM    226  HB  VAL    19       30.135   6.019  15.517  1.00
11.49           H
ATOM    227 HG11 VAL    19       29.432   5.529  13.247
1.00137.43           H
ATOM    228 HG12 VAL    19       29.563   7.227  12.796  1.00
93.29           H
ATOM    229 HG13 VAL    19       28.309   6.708  13.921
1.00108.69           H
ATOM    230 HG21 VAL    19       31.814   7.284  13.346  1.00
```

```
99.42           H
ATOM    231 HG22 VAL    19       32.027   5.706  14.107
1.00111.36           H
ATOM    232 HG23 VAL    19       32.383   7.182  15.011  1.00
77.11           H
ATOM    233  N   PHE    20       31.484   9.971  15.032  1.00
24.58           N
ATOM    234  CA  PHE    20       32.102  11.094  14.349  1.00
40.34           C
ATOM    235  C   PHE    20       33.373  10.671  13.618  1.00
40.37           C
ATOM    236  O   PHE    20       33.335  10.237  12.466  1.00
66.02           O
ATOM    237  CB  PHE    20       32.427  12.194  15.385  1.00
48.79           C
ATOM    238  CG  PHE    20       32.644  11.593  16.766  1.00
35.75           C
ATOM    239  CD1 PHE    20       31.573  11.006  17.468  1.00
30.20           C
ATOM    240  CD2 PHE    20       33.914  11.640  17.358  1.00
40.95           C
ATOM    241  CE1 PHE    20       31.780  10.472  18.743  1.00
30.41           C
ATOM    242  CE2 PHE    20       34.117  11.102  18.634  1.00
45.84           C
ATOM    243  CZ  PHE    20       33.052  10.518  19.326  1.00
40.81           C
ATOM    244  HN  PHE    20       31.763   9.750  15.937  1.00
16.73           H
ATOM    245  HA  PHE    20       31.407  11.496  13.624  1.00
59.91           H
ATOM    246  HB1 PHE    20       31.608  12.873  15.435  1.00
67.15           H
ATOM    247  HB2 PHE    20       33.310  12.741  15.087  1.00
58.17           H
ATOM    248  HD1 PHE    20       30.587  10.957  17.021  1.00
33.34           H
ATOM    249  HD2 PHE    20       34.736  12.092  16.828  1.00
49.35           H
ATOM    250  HE1 PHE    20       30.958  10.024  19.277  1.00
30.61           H
ATOM    251  HE2 PHE    20       35.099  11.137  19.084  1.00
61.27           H
ATOM    252  HZ  PHE    20       33.209  10.104  20.310  1.00
52.66           H
ATOM    253  N   GLU    21       34.493  10.836  14.297  1.00
41.31           N
ATOM    254  CA  GLU    21       35.795  10.515  13.734  1.00
56.16           C
ATOM    255  C   GLU    21       36.072   9.010  13.806  1.00
58.61           C
ATOM    256  O   GLU    21       36.922   8.495  13.079
```

1.00201.19 O
ATOM 257 CB GLU 21 36.850 11.328 14.504 1.00
57.37 C
ATOM 258 CG GLU 21 38.228 10.652 14.458
1.00193.56 C
ATOM 259 CD GLU 21 39.293 11.605 14.990
1.00304.62 C
ATOM 260 OE1 GLU 21 40.404 11.558 14.489
1.00451.81 O
ATOM 261 OE2 GLU 21 38.981 12.367 15.889
1.00442.88 O1-
ATOM 262 HN GLU 21 34.445 11.205 15.203 1.00
50.82 H
ATOM 263 HA GLU 21 35.810 10.819 12.698 1.00
85.72 H
ATOM 264 HB1 GLU 21 36.527 11.428 15.529 1.00
46.37 H
ATOM 265 HB2 GLU 21 36.928 12.315 14.064
1.00131.81 H
ATOM 266 HG1 GLU 21 38.464 10.386 13.438
1.00314.91 H
ATOM 267 HG2 GLU 21 38.211 9.760 15.067
1.00266.92 H
ATOM 268 N GLY 22 35.353 8.310 14.678 1.00
86.70 N
ATOM 269 CA GLY 22 35.534 6.866 14.826
1.00104.66 C
ATOM 270 C GLY 22 36.519 6.544 15.944 1.00
71.50 C
ATOM 271 O GLY 22 36.653 5.389 16.349 1.00
89.87 O
ATOM 272 HN GLY 22 34.688 8.770 15.233
1.00214.64 H
ATOM 273 HA1 GLY 22 35.905 6.450 13.900
1.00152.47 H
ATOM 274 HA2 GLY 22 34.582 6.412 15.058
1.00118.15 H
ATOM 275 N ASP 23 37.200 7.567 16.450 1.00
44.68 N
ATOM 276 CA ASP 23 38.154 7.360 17.534 1.00
43.33 C
ATOM 277 C ASP 23 37.431 6.789 18.744 1.00
37.55 C
ATOM 278 O ASP 23 37.922 5.878 19.411 1.00
61.37 O
ATOM 279 CB ASP 23 38.823 8.684 17.909 1.00
40.74 C
ATOM 280 CG ASP 23 39.870 8.449 18.993
1.00141.86 C
ATOM 281 OD1 ASP 23 39.937 7.340 19.494
1.00328.18 O
ATOM 282 OD2 ASP 23 40.589 9.384 19.307

1.00304.58 O1-
ATOM 283 HN ASP 23 37.051 8.470 16.100 1.00
40.86 H
ATOM 284 HA ASP 23 38.909 6.661 17.211 1.00
72.12 H
ATOM 285 HB1 ASP 23 38.076 9.372 18.277 1.00
94.15 H
ATOM 286 HB2 ASP 23 39.300 9.105 17.035 1.00
93.26 H
ATOM 287 N MET 24 36.247 7.333 19.009 1.00
23.24 N
ATOM 288 CA MET 24 35.419 6.888 20.127 1.00
32.50 C
ATOM 289 C MET 24 33.962 6.823 19.684 1.00
25.13 C
ATOM 290 O MET 24 33.277 7.845 19.636 1.00
54.40 O
ATOM 291 CB MET 24 35.550 7.870 21.292 1.00
47.36 C
ATOM 292 CG MET 24 37.024 8.014 21.675
1.00151.73 C
ATOM 293 SD MET 24 37.175 9.161 23.070
1.00209.23 S
ATOM 294 CE MET 24 36.658 10.665 22.203
1.00243.29 C
ATOM 295 HN MET 24 35.916 8.050 18.428 1.00
17.32 H
ATOM 296 HA MET 24 35.735 5.907 20.452 1.00
48.94 H
ATOM 297 HB1 MET 24 34.995 7.499 22.140
1.00124.40 H
ATOM 298 HB2 MET 24 35.157 8.832 20.996
1.00166.96 H
ATOM 299 HG1 MET 24 37.580 8.396 20.831
1.00331.71 H
ATOM 300 HG2 MET 24 37.418 7.050 21.957
1.00302.30 H
ATOM 301 HE1 MET 24 37.116 11.524 22.674
1.00340.44 H
ATOM 302 HE2 MET 24 35.584 10.761 22.255
1.00373.88 H
ATOM 303 HE3 MET 24 36.960 10.610 21.168
1.00386.81 H
ATOM 304 N VAL 25 33.493 5.626 19.352 1.00
22.44 N
ATOM 305 CA VAL 25 32.115 5.472 18.905 1.00
15.37 C
ATOM 306 C VAL 25 31.142 5.793 20.029 1.00
17.47 C
ATOM 307 O VAL 25 31.412 5.530 21.201 1.00
29.52 O
ATOM 308 CB VAL 25 31.857 4.057 18.390 1.00

```
23.36           C
ATOM    309  CG1 VAL    25       30.376   3.927  18.006  1.00
58.39           C
ATOM    310  CG2 VAL    25       32.731   3.795  17.160  1.00
55.25           C
ATOM    311  HN  VAL    25       34.080   4.844  19.400  1.00
48.11           H
ATOM    312  HA  VAL    25       31.941   6.165  18.096  1.00
9.25           H
ATOM    313  HB  VAL    25       32.093   3.341  19.165  1.00
52.45           H
ATOM    314 HG11 VAL    25       29.786   3.779  18.897
1.00171.07           H
ATOM    315 HG12 VAL    25       30.244   3.084  17.344
1.00166.22           H
ATOM    316 HG13 VAL    25       30.051   4.832  17.507
1.00134.76           H
ATOM    317 HG21 VAL    25       32.742   2.735  16.946
1.00141.88           H
ATOM    318 HG22 VAL    25       33.737   4.135  17.354
1.00184.50           H
ATOM    319 HG23 VAL    25       32.327   4.328  16.313
1.00133.90           H
ATOM    320  N   THR    26       30.010   6.379  19.653  1.00
13.60           N
ATOM    321  CA  THR    26       28.976   6.767  20.611  1.00
21.19           C
ATOM    322  C   THR    26       27.625   6.169  20.228  1.00
16.55           C
ATOM    323  O   THR    26       27.243   6.175  19.058  1.00
11.43           O
ATOM    324  CB  THR    26       28.877   8.289  20.630  1.00
27.07           C
ATOM    325  CG2 THR    26       27.750   8.728  21.568  1.00
44.39           C
ATOM    326  OG1 THR    26       30.110   8.828  21.084  1.00
34.75           O
ATOM    327  HN  THR    26       29.868   6.567  18.702  1.00
10.81           H
ATOM    328  HA  THR    26       29.245   6.423  21.601  1.00
33.16           H
ATOM    329  HB  THR    26       28.677   8.641  19.627  1.00
20.60           H
ATOM    330  HG1 THR    26       30.820   8.357  20.641  1.00
77.72           H
ATOM    331 HG21 THR    26       27.865   8.239  22.523
1.00126.99           H
ATOM    332 HG22 THR    26       26.796   8.461  21.140
1.00108.32           H
ATOM    333 HG23 THR    26       27.795   9.798  21.705
1.00151.99           H
ATOM    334  N   ARG    27       26.905   5.656  21.224  1.00
```

```
22.38           N
ATOM    335  CA  ARG    27      25.592   5.057  20.988  1.00
20.21           C
ATOM    336  C   ARG    27      24.695   5.229  22.210  1.00
16.96           C
ATOM    337  O   ARG    27      25.179   5.479  23.315  1.00
20.06           O
ATOM    338  CB  ARG    27      25.749   3.567  20.665  1.00
22.14           C
ATOM    339  CG  ARG    27      26.313   2.832  21.883
1.00124.30           C
ATOM    340  CD  ARG    27      26.642   1.388  21.499
1.00109.38           C
ATOM    341  NE  ARG    27      27.138   0.658  22.660
1.00227.73           N
ATOM    342  CZ  ARG    27      28.404   0.764  23.050
1.00426.12           C
ATOM    343  NH1 ARG    27      28.825   0.097  24.091
1.00767.09           N1+
ATOM    344  NH2 ARG    27      29.227   1.535  22.394
1.00581.78           N
ATOM    345  HN  ARG    27      27.262   5.682  22.136  1.00
30.75           H
ATOM    346  HA  ARG    27      25.125   5.547  20.145  1.00
23.13           H
ATOM    347  HB1 ARG    27      26.424   3.449  19.831  1.00
87.46           H
ATOM    348  HB2 ARG    27      24.785   3.152  20.408
1.00103.36           H
ATOM    349  HG1 ARG    27      25.583   2.833  22.677
1.00281.97           H
ATOM    350  HG2 ARG    27      27.212   3.330  22.219
1.00276.19           H
ATOM    351  HD1 ARG    27      27.396   1.387  20.725
1.00183.60           H
ATOM    352  HD2 ARG    27      25.750   0.905  21.130
1.00142.93           H
ATOM    353  HE  ARG    27      26.526   0.078  23.162
1.00372.53           H
ATOM    354 HH11 ARG    27      28.195  -0.493  24.594
1.00910.59           H
ATOM    355 HH12 ARG    27      29.778   0.176  24.383
1.00999.99           H
ATOM    356 HH21 ARG    27      28.905   2.045  21.597
1.00532.54           H
ATOM    357 HH22 ARG    27      30.180   1.614  22.685
1.00948.84           H
ATOM    358  N   SER    28      23.385   5.099  22.005  1.00
14.60           N
ATOM    359  CA  SER    28      22.427   5.247  23.099  1.00
14.92           C
ATOM    360  C   SER    28      21.163   4.434  22.826  1.00
```

12.12 C
ATOM 361 O SER 28 20.782 4.226 21.675 1.00
13.41 O
ATOM 362 CB SER 28 22.058 6.721 23.275 1.00
23.26 C
ATOM 363 OG SER 28 21.128 6.848 24.343
1.00146.32 O
ATOM 364 HN SER 28 23.057 4.902 21.103 1.00
15.23 H
ATOM 365 HA SER 28 22.877 4.892 24.015 1.00
15.44 H
ATOM 366 HB1 SER 28 21.623 7.094 22.357 1.00
88.73 H
ATOM 367 HB2 SER 28 22.944 7.290 23.507
1.00124.68 H
ATOM 368 HG SER 28 21.142 7.760 24.641
1.00242.47 H
ATOM 369 N CYS 29 20.519 3.984 23.898 1.00
10.48 N
ATOM 370 CA CYS 29 19.296 3.198 23.778 1.00
9.97 C
ATOM 371 C CYS 29 18.651 3.036 25.151 1.00
11.60 C
ATOM 372 O CYS 29 19.298 2.596 26.102 1.00
15.64 O
ATOM 373 CB CYS 29 19.612 1.820 23.174 1.00
9.52 C
ATOM 374 SG CYS 29 18.158 1.199 22.319 1.00
10.40 S
ATOM 375 HN CYS 29 20.868 4.191 24.789 1.00
11.10 H
ATOM 376 HA CYS 29 18.607 3.718 23.125 1.00
11.08 H
ATOM 377 HB1 CYS 29 19.883 1.121 23.956 1.00
9.33 H
ATOM 378 HB2 CYS 29 20.425 1.905 22.473 1.00
10.78 H
ATOM 379 N GLU 30 17.378 3.404 25.251 1.00
13.52 N
ATOM 380 CA GLU 30 16.660 3.305 26.519 1.00
17.41 C
ATOM 381 C GLU 30 16.086 1.907 26.714 1.00
10.81 C
ATOM 382 O GLU 30 15.789 1.204 25.749 1.00
23.22 O
ATOM 383 CB GLU 30 15.530 4.330 26.552 1.00
37.83 C
ATOM 384 CG GLU 30 16.117 5.733 26.392
1.00108.13 C
ATOM 385 CD GLU 30 16.940 6.097 27.622
1.00240.52 C
ATOM 386 OE1 GLU 30 17.735 7.017 27.527

1.00422.56 O
ATOM 387 OE2 GLU 30 16.763 5.450 28.642
1.00410.81 O1-
ATOM 388 HN GLU 30 16.914 3.754 24.462 1.00
15.61 H
ATOM 389 HA GLU 30 17.342 3.517 27.329 1.00
24.60 H
ATOM 390 HB1 GLU 30 15.009 4.265 27.495 1.00
72.67 H
ATOM 391 HB2 GLU 30 14.840 4.132 25.743 1.00
56.47 H
ATOM 392 HG1 GLU 30 15.317 6.446 26.273
1.00196.66 H
ATOM 393 HG2 GLU 30 16.751 5.755 25.517
1.00140.67 H
ATOM 394 N LYS 31 15.926 1.512 27.978 1.00
11.96 N
ATOM 395 CA LYS 31 15.378 0.197 28.314 1.00
11.40 C
ATOM 396 C LYS 31 14.059 0.358 29.060 1.00
10.99 C
ATOM 397 O LYS 31 13.901 1.275 29.868 1.00
12.86 O
ATOM 398 CB LYS 31 16.364 -0.572 29.193 1.00
21.40 C
ATOM 399 CG LYS 31 17.657 -0.821 28.414 1.00
57.81 C
ATOM 400 CD LYS 31 18.640 -1.601 29.289
1.00115.52 C
ATOM 401 CE LYS 31 19.923 -1.870 28.503
1.00250.23 C
ATOM 402 NZ LYS 31 20.573 -0.574 28.158
1.00462.10 N1+
ATOM 403 HN LYS 31 16.179 2.123 28.702 1.00
26.13 H
ATOM 404 HA LYS 31 15.201 -0.369 27.409 1.00
13.87 H
ATOM 405 HB1 LYS 31 15.931 -1.519 29.477 1.00
37.63 H
ATOM 406 HB2 LYS 31 16.580 0.006 30.079 1.00
47.19 H
ATOM 407 HG1 LYS 31 18.098 0.124 28.136
1.00126.32 H
ATOM 408 HG2 LYS 31 17.436 -1.392 27.524
1.00114.19 H
ATOM 409 HD1 LYS 31 18.194 -2.541 29.581
1.00198.45 H
ATOM 410 HD2 LYS 31 18.872 -1.022 30.173
1.00200.14 H
ATOM 411 HE1 LYS 31 19.685 -2.406 27.597
1.00374.64 H
ATOM 412 HE2 LYS 31 20.597 -2.461 29.106

```
1.00403.27           H
ATOM    413  HZ1 LYS    31      19.920   0.001  27.591
1.00627.38           H
ATOM    414  HZ2 LYS    31      21.440  -0.757  27.611
1.00622.26           H
ATOM    415  HZ3 LYS    31      20.815  -0.063  29.030
1.00619.20           H
ATOM    416  N   THR    32      13.115  -0.532  28.784  1.00
15.51          N
ATOM    417  CA  THR    32      11.813  -0.470  29.435  1.00
22.44          C
ATOM    418  C   THR    32      11.928  -0.879  30.900  1.00
26.60          C
ATOM    419  O   THR    32      12.421  -1.960  31.219  1.00
57.43          O
ATOM    420  CB  THR    32      10.826  -1.393  28.718  1.00
56.28          C
ATOM    421  CG2 THR    32       9.461  -1.315  29.402
1.00102.70           C
ATOM    422  OG1 THR    32      10.704  -0.989  27.362
1.00111.17           O
ATOM    423  HN  THR    32      13.296  -1.237  28.131  1.00
18.79          H
ATOM    424  HA  THR    32      11.444   0.543  29.382  1.00
19.67          H
ATOM    425  HB  THR    32      11.187  -2.409  28.760  1.00
84.25          H
ATOM    426  HG1 THR    32      11.237  -1.580  26.826
1.00205.03           H
ATOM    427 HG21 THR    32       9.523  -1.760  30.385
1.00220.71           H
ATOM    428 HG22 THR    32       8.731  -1.849  28.811
1.00174.67           H
ATOM    429 HG23 THR    32       9.164  -0.280  29.494
1.00217.44           H
ATOM    430  N   THR    33      11.467  -0.002  31.784  1.00
22.47          N
ATOM    431  CA  THR    33      11.514  -0.267  33.219  1.00
41.60          C
ATOM    432  C   THR    33      10.434   0.497  33.932  1.00
38.03          C
ATOM    433  O   THR    33      10.639   1.620  34.391  1.00
52.73          O
ATOM    434  CB  THR    33      12.882   0.111  33.791  1.00
63.25          C
ATOM    435  CG2 THR    33      13.950  -0.852  33.268
1.00121.65           C
ATOM    436  OG1 THR    33      13.205   1.438  33.398  1.00
96.20          O
ATOM    437  HN  THR    33      11.085   0.842  31.466  1.00
23.82          H
ATOM    438  HA  THR    33      11.333  -1.310  33.399  1.00
```

```
67.12              H
ATOM    439  HB  THR    33      12.848   0.053  34.868
1.00107.59              H
ATOM    440  HG1 THR    33      13.684   1.392  32.568
1.00176.27              H
ATOM    441 HG21 THR    33      14.157  -0.632  32.231
1.00255.23              H
ATOM    442 HG22 THR    33      13.596  -1.867  33.357
1.00245.37              H
ATOM    443 HG23 THR    33      14.855  -0.734  33.847
1.00185.78              H
ATOM    444  N   GLY    34       9.280  -0.139  34.040  1.00
40.93              N
ATOM    445  CA  GLY    34       8.168   0.473  34.720  1.00
45.59              C
ATOM    446  C   GLY    34       7.567   1.597  33.883  1.00
40.97              C
ATOM    447  O   GLY    34       6.351   1.675  33.711  1.00
82.17              O
ATOM    448  HN  GLY    34       9.185  -1.039  33.665  1.00
54.00              H
ATOM    449  HA1 GLY    34       8.531   0.871  35.646  1.00
54.41              H
ATOM    450  HA2 GLY    34       7.412  -0.276  34.918  1.00
62.09              H
ATOM    451  N   ASN    35       8.434   2.463  33.355  1.00
32.51              N
ATOM    452  CA  ASN    35       7.993   3.584  32.523  1.00
33.20              C
ATOM    453  C   ASN    35       8.875   3.701  31.284  1.00
23.17              C
ATOM    454  O   ASN    35      10.094   3.545  31.361  1.00
27.01              O
ATOM    455  CB  ASN    35       8.059   4.887  33.322  1.00
54.51              C
ATOM    456  CG  ASN    35       7.241   4.763  34.593  1.00
80.73              C
ATOM    457  ND2 ASN    35       5.970   4.511  34.511
1.00220.46              N
ATOM    458  OD1 ASN    35       7.774   4.900  35.694
1.00117.80              O
ATOM    459  HN  ASN    35       9.391   2.342  33.526  1.00
51.15              H
ATOM    460  HA  ASN    35       6.971   3.421  32.207  1.00
47.21              H
ATOM    461  HB1 ASN    35       7.670   5.694  32.722  1.00
68.31              H
ATOM    462  HB2 ASN    35       9.080   5.093  33.583  1.00
57.75              H
ATOM    463 HD21 ASN    35       5.546   4.403  33.636
1.00405.39              H
ATOM    464 HD22 ASN    35       5.442   4.431  35.325
```

```
1.00245.64           H
ATOM    465  N   PHE    36        8.251   3.972  30.143  1.00
35.72           N
ATOM    466  CA  PHE    36        8.977   4.103  28.891  1.00
33.41           C
ATOM    467  C   PHE    36        9.544   5.513  28.742  1.00
28.51           C
ATOM    468  O   PHE    36        8.951   6.482  29.215  1.00
45.23           O
ATOM    469  CB  PHE    36        8.029   3.807  27.735  1.00
67.53           C
ATOM    470  CG  PHE    36        7.586   2.365  27.806  1.00
86.71           C
ATOM    471  CD1 PHE    36        6.443   2.024  28.539
1.00106.51           C
ATOM    472  CD2 PHE    36        8.312   1.372  27.139  1.00
99.48           C
ATOM    473  CE1 PHE    36        6.026   0.689  28.605
1.00134.48           C
ATOM    474  CE2 PHE    36        7.896   0.037  27.206
1.00134.27           C
ATOM    475  CZ  PHE    36        6.753  -0.305  27.939
1.00149.48           C
ATOM    476  HN  PHE    36        7.280   4.081  30.140  1.00
62.87           H
ATOM    477  HA  PHE    36        9.788   3.390  28.870  1.00
28.53           H
ATOM    478  HB1 PHE    36        8.534   3.982  26.804  1.00
73.56           H
ATOM    479  HB2 PHE    36        7.165   4.454  27.802  1.00
88.42           H
ATOM    480  HD1 PHE    36        5.883   2.791  29.054
1.00110.20           H
ATOM    481  HD2 PHE    36        9.195   1.634  26.573  1.00
93.07           H
ATOM    482  HE1 PHE    36        5.145   0.426  29.170
1.00155.06           H
ATOM    483  HE2 PHE    36        8.457  -0.730  26.691
1.00159.46           H
ATOM    484  HZ  PHE    36        6.433  -1.335  27.989
1.00183.25           H
ATOM    485  N   THR    37       10.695   5.621  28.076  1.00
20.06           N
ATOM    486  CA  THR    37       11.345   6.913  27.862  1.00
27.18           C
ATOM    487  C   THR    37       11.611   7.125  26.376  1.00
41.87           C
ATOM    488  O   THR    37       10.822   6.711  25.526
1.00169.86           O
ATOM    489  CB  THR    37       12.666   6.968  28.631  1.00
26.52           C
ATOM    490  CG2 THR    37       12.423   6.594  30.094  1.00
```

75.69 C
ATOM 491 OG1 THR 37 13.587 6.055 28.051 1.00
81.95 O
ATOM 492 HN THR 37 11.120 4.814 27.720 1.00
18.47 H
ATOM 493 HA THR 37 10.702 7.709 28.217 1.00
38.80 H
ATOM 494 HB THR 37 13.070 7.967 28.583 1.00
81.60 H
ATOM 495 HG1 THR 37 13.129 5.571 27.359
1.00188.80 H
ATOM 496 HG21 THR 37 12.108 5.561 30.153
1.00196.98 H
ATOM 497 HG22 THR 37 11.653 7.229 30.505
1.00196.23 H
ATOM 498 HG23 THR 37 13.336 6.723 30.655
1.00168.92 H
ATOM 499 N GLU 38 12.726 7.778 26.070 1.00
26.53 N
ATOM 500 CA GLU 38 13.090 8.047 24.681 1.00
30.29 C
ATOM 501 C GLU 38 14.597 8.269 24.555 1.00
25.39 C
ATOM 502 O GLU 38 15.222 8.859 25.435 1.00
51.60 O
ATOM 503 CB GLU 38 12.338 9.287 24.181 1.00
54.82 C
ATOM 504 CG GLU 38 12.642 9.533 22.696
1.00165.84 C
ATOM 505 CD GLU 38 12.136 8.366 21.855
1.00292.92 C
ATOM 506 OE1 GLU 38 11.278 7.644 22.337
1.00414.19 O
ATOM 507 OE2 GLU 38 12.613 8.213 20.743
1.00537.65 O1-
ATOM 508 HN GLU 38 13.312 8.083 26.790 1.00
84.50 H
ATOM 509 HA GLU 38 12.811 7.199 24.073 1.00
32.15 H
ATOM 510 HB1 GLU 38 12.647 10.149 24.754
1.00144.82 H
ATOM 511 HB2 GLU 38 11.276 9.136 24.308
1.00106.49 H
ATOM 512 HG1 GLU 38 13.707 9.639 22.557
1.00303.31 H
ATOM 513 HG2 GLU 38 12.148 10.440 22.379
1.00323.99 H
ATOM 514 N CYS 39 15.168 7.797 23.452 1.00
15.84 N
ATOM 515 CA CYS 39 16.599 7.953 23.213 1.00
14.30 C
ATOM 516 C CYS 39 16.957 9.441 23.126 1.00

```
22.31           C
ATOM    517  O   CYS    39       16.134  10.249  22.697  1.00
32.92           O
ATOM    518  CB  CYS    39       16.972   7.260  21.899  1.00
18.08           C
ATOM    519  SG  CYS    39       15.870   7.846  20.587  1.00
17.41           S
ATOM    520  HN  CYS    39       14.617   7.340  22.783  1.00
24.87           H
ATOM    521  HA  CYS    39       17.141   7.489  24.020  1.00
14.60           H
ATOM    522  HB1 CYS    39       16.867   6.193  22.011  1.00
27.71           H
ATOM    523  HB2 CYS    39       17.994   7.498  21.645  1.00
34.91           H
ATOM    524  N   PRO    40       18.153   9.826  23.515  1.00
27.27           N
ATOM    525  CA  PRO    40       18.582  11.255  23.462  1.00
47.66           C
ATOM    526  C   PRO    40       18.919  11.692  22.038  1.00
71.54           C
ATOM    527  O   PRO    40       19.601  10.980  21.303  1.00
87.70           O
ATOM    528  CB  PRO    40       19.826  11.280  24.358  1.00
53.85           C
ATOM    529  CG  PRO    40       20.416   9.916  24.209  1.00
46.04           C
ATOM    530  CD  PRO    40       19.227   8.963  24.042  1.00
25.70           C
ATOM    531  HA  PRO    40       17.821  11.891  23.880  1.00
58.04           H
ATOM    532  HB1 PRO    40       19.544  11.452  25.386  1.00
67.93           H
ATOM    533  HB2 PRO    40       20.523  12.040  24.025  1.00
70.99           H
ATOM    534  HG1 PRO    40       20.978   9.650  25.093  1.00
65.91           H
ATOM    535  HG2 PRO    40       21.054   9.882  23.334  1.00
54.99           H
ATOM    536  HD1 PRO    40       18.938   8.545  24.994  1.00
24.22           H
ATOM    537  HD2 PRO    40       19.465   8.181  23.335  1.00
26.94           H
ATOM    538  N   GLY    41       18.438  12.869  21.661
1.00105.24           N
ATOM    539  CA  GLY    41       18.698  13.396  20.327
1.00147.55           C
ATOM    540  C   GLY    41       20.189  13.627  20.120
1.00161.74           C
ATOM    541  O   GLY    41       20.740  13.305  19.068
1.00244.37           O
ATOM    542  HN  GLY    41       17.905  13.393  22.293
```

```
1.00121.79            H
ATOM    543  HA1 GLY    41       18.175  14.331  20.204
1.00184.40            H
ATOM    544  HA2 GLY    41       18.343  12.691  19.593
1.00157.34            H
ATOM    545  N   LEU    42       20.834  14.186  21.141
1.00162.48            N
ATOM    546  CA  LEU    42       22.273  14.467  21.094
1.00191.48            C
ATOM    547  C   LEU    42       22.962  13.863  22.314
1.00166.66            C
ATOM    548  O   LEU    42       22.432  13.914  23.423
1.00251.38            O
ATOM    549  CB  LEU    42       22.507  15.979  21.080
1.00307.99            C
ATOM    550  CG  LEU    42       21.794  16.615  19.880
1.00452.28            C
ATOM    551  CD1 LEU    42       21.942  18.137  19.958
1.00681.24            C
ATOM    552  CD2 LEU    42       22.415  16.106  18.565
1.00527.46            C
ATOM    553  HN  LEU    42       20.331  14.411  21.949
1.00195.47            H
ATOM    554  HA  LEU    42       22.703  14.034  20.202
1.00229.77            H
ATOM    555  HB1 LEU    42       23.567  16.178  21.013
1.00342.49            H
ATOM    556  HB2 LEU    42       22.120  16.408  21.993
1.00335.33            H
ATOM    557  HG  LEU    42       20.745  16.355  19.909
1.00427.79            H
ATOM    558 HD11 LEU    42       21.284  18.601  19.240
1.00909.71            H
ATOM    559 HD12 LEU    42       22.964  18.412  19.741
1.00706.80            H
ATOM    560 HD13 LEU    42       21.683  18.472  20.952
1.00858.82            H
ATOM    561 HD21 LEU    42       23.483  15.998  18.683
1.00631.69            H
ATOM    562 HD22 LEU    42       22.213  16.811  17.771
1.00628.06            H
ATOM    563 HD23 LEU    42       21.982  15.153  18.308
1.00656.75            H
ATOM    564  N   THR    43       24.150  13.294  22.104
1.00178.52            N
ATOM    565  CA  THR    43       24.911  12.683  23.199
1.00240.84            C
ATOM    566  C   THR    43       26.159  13.526  23.515
1.00372.31            C
ATOM    567  O   THR    43       27.042  13.651  22.667
1.00500.13            O
ATOM    568  CB  THR    43       25.361  11.281  22.797
```

```
1.00354.13              C
ATOM    569  CG2 THR    43       26.039  10.602  23.988
1.00463.04              C
ATOM    570  OG1 THR    43       24.232  10.518  22.396
1.00508.50              O
ATOM    571  HN  THR    43       24.526  13.284  21.199
1.00229.59              H
ATOM    572  HA  THR    43       24.282  12.600  24.068
1.00249.90              H
ATOM    573  HB  THR    43       26.061  11.352  21.987
1.00481.58              H
ATOM    574  HG1 THR    43       23.970  10.814  21.521
1.00624.31              H
ATOM    575 HG21 THR    43       26.949  11.128  24.232
1.00650.95              H
ATOM    576 HG22 THR    43       26.271   9.579  23.734
1.00580.73              H
ATOM    577 HG23 THR    43       25.374  10.619  24.840
1.00571.99              H
ATOM    578  N   PRO    44       26.268  14.103  24.694
1.00488.39              N
ATOM    579  CA  PRO    44       27.451  14.931  25.069
1.00760.13              C
ATOM    580  C   PRO    44       28.621  14.074  25.550
1.00735.36              C
ATOM    581  O   PRO    44       29.028  14.160  26.710
1.00999.99              O
ATOM    582  CB  PRO    44       26.905  15.803  26.201
1.00999.99              C
ATOM    583  CG  PRO    44       25.916  14.923  26.895
1.00856.94              C
ATOM    584  CD  PRO    44       25.295  14.039  25.803
1.00548.09              C
ATOM    585  HA  PRO    44       27.752  15.554  24.243
1.00939.00              H
ATOM    586  HB1 PRO    44       26.408  16.673  25.798
1.00999.99              H
ATOM    587  HB2 PRO    44       27.701  16.099  26.874
1.00999.99              H
ATOM    588  HG1 PRO    44       25.149  15.517  27.368
1.00999.99              H
ATOM    589  HG2 PRO    44       26.422  14.311  27.633
1.00853.53              H
ATOM    590  HD1 PRO    44       24.344  14.438  25.485
1.00602.45              H
ATOM    591  HD2 PRO    44       25.187  13.018  26.149
1.00463.32              H
ATOM    592  N   ILE    45       29.157  13.247  24.658
1.00518.33              N
ATOM    593  CA  ILE    45       30.277  12.383  25.015
1.00582.79              C
ATOM    594  C   ILE    45       31.533  13.210  25.273
```

```
1.00795.93           C
ATOM    595  O   ILE    45       31.796  14.193  24.579
1.00898.75           O
ATOM    596  CB  ILE    45       30.548  11.381  23.893
1.00474.41           C
ATOM    597  CG1 ILE    45       31.629  10.395  24.344
1.00516.90           C
ATOM    598  CG2 ILE    45       31.027  12.126  22.644
1.00760.75           C
ATOM    599  CD1 ILE    45       31.699   9.225  23.361
1.00566.81           C
ATOM    600  HN  ILE    45       28.794  13.217  23.749
1.00395.72           H
ATOM    601  HA  ILE    45       30.027  11.841  25.913
1.00711.06           H
ATOM    602  HB  ILE    45       29.639  10.842  23.663
1.00477.81           H
ATOM    603 HG11 ILE    45       31.388  10.022  25.327
1.00624.28           H
ATOM    604 HG12 ILE    45       32.584  10.898  24.374
1.00744.84           H
ATOM    605 HG21 ILE    45       31.013  11.455  21.798
1.00920.57           H
ATOM    606 HG22 ILE    45       32.034  12.484  22.804
1.00999.99           H
ATOM    607 HG23 ILE    45       30.373  12.963  22.452
1.00865.46           H
ATOM    608 HD11 ILE    45       31.923   9.598  22.374
1.00658.89           H
ATOM    609 HD12 ILE    45       30.750   8.710  23.346
1.00734.30           H
ATOM    610 HD13 ILE    45       32.473   8.540  23.672
1.00675.84           H
ATOM    611  N   ALA    46       32.307  12.802  26.275
1.00999.99           N
ATOM    612  CA  ALA    46       33.535  13.512  26.617
1.00999.99           C
ATOM    613  C   ALA    46       34.332  12.729  27.654
1.00999.99           C
ATOM    614  CB  ALA    46       33.200  14.898  27.168
1.00999.99           C
ATOM    615  OT1 ALA    46       35.492  12.451  27.395
1.00999.99           O
ATOM    616  OT2 ALA    46       33.773  12.416  28.692
1.00999.99           O
ATOM    617  HN  ALA    46       32.047  12.012  26.792
1.00999.99           H
ATOM    618  HA  ALA    46       34.133  13.625  25.726
1.00999.99           H
ATOM    619  HB1 ALA    46       32.504  14.802  27.989
1.00999.99           H
ATOM    620  HB2 ALA    46       32.756  15.498  26.387
```

```
1.00999.99            H
ATOM    621  HB3 ALA    46       34.105  15.374  27.518
1.00999.99            H
ENDMDL
TER
MODEL       17
ATOM      1  N   GLY     1       26.097  -3.915  15.312
1.00999.99            N
ATOM      2  CA  GLY     1       25.979  -2.924  16.418
1.00999.99            C
ATOM      3  C   GLY     1       25.032  -1.804  16.002
1.00999.99            C
ATOM      4  O   GLY     1       24.700  -0.930  16.801
1.00999.99            O
ATOM      5  HA1 GLY     1       26.952  -2.506  16.632
1.00999.99            H
ATOM      6  HA2 GLY     1       25.595  -3.413  17.302
1.00999.99            H
ATOM      7  HT1 GLY     1       27.093  -4.193  15.198
1.00999.99            H
ATOM      8  HT2 GLY     1       25.752  -3.491  14.427
1.00999.99            H
ATOM      9  HT3 GLY     1       25.529  -4.756  15.538
1.00999.99            H
ATOM     10  N   LEU     2       24.601  -1.839  14.745
1.00999.99            N
ATOM     11  CA  LEU     2       23.690  -0.822  14.232
1.00895.59            C
ATOM     12  C   LEU     2       22.272  -1.077  14.735
1.00532.27            C
ATOM     13  O   LEU     2       21.884  -2.222  14.966
1.00625.21            O
ATOM     14  CB  LEU     2       23.697  -0.834  12.701
1.00999.99            C
ATOM     15  CG  LEU     2       25.124  -0.618  12.180
1.00999.99            C
ATOM     16  CD1 LEU     2       25.115  -0.703  10.650
1.00999.99            C
ATOM     17  CD2 LEU     2       25.645   0.761  12.619
1.00999.99            C
ATOM     18  HN  LEU     2       24.898  -2.560  14.153
1.00999.99            H
ATOM     19  HA  LEU     2       24.012   0.148  14.578
1.00890.87            H
ATOM     20  HB1 LEU     2       23.059  -0.044  12.334
1.00924.07            H
ATOM     21  HB2 LEU     2       23.326  -1.786  12.350
1.00999.99            H
ATOM     22  HG  LEU     2       25.768  -1.391  12.576
1.00999.99            H
ATOM     23 HD11 LEU     2       24.558   0.130  10.247
1.00999.99            H
```

```
ATOM     24 HD12 LEU     2       24.652  -1.628  10.343
1.00999.99           H
ATOM     25 HD13 LEU     2       26.130  -0.669  10.283
1.00999.99           H
ATOM     26 HD21 LEU     2       26.428   1.087  11.948
1.00999.99           H
ATOM     27 HD22 LEU     2       26.046   0.693  13.620
1.00999.99           H
ATOM     28 HD23 LEU     2       24.837   1.481  12.603
1.00999.99           H
ATOM     29  N   CYS     3       21.501  -0.006  14.902
1.00271.28           N
ATOM     30  CA  CYS     3       20.129  -0.139  15.379
1.00104.50           C
ATOM     31  C   CYS     3       19.297  -0.955  14.398  1.00
90.98           C
ATOM     32  O   CYS     3       18.487  -1.790  14.799
1.00207.28           O
ATOM     33  CB  CYS     3       19.496   1.241  15.565  1.00
37.83           C
ATOM     34  SG  CYS     3       20.239   2.066  16.995  1.00
81.56           S
ATOM     35  HN  CYS     3       21.860   0.883  14.703
1.00296.55           H
ATOM     36  HA  CYS     3       20.142  -0.644  16.328
1.00166.45           H
ATOM     37  HB1 CYS     3       18.434   1.131  15.727  1.00
40.38           H
ATOM     38  HB2 CYS     3       19.664   1.837  14.680  1.00
98.45           H
ATOM     39  N   SER     4       19.500  -0.710  13.109  1.00
85.31           N
ATOM     40  CA  SER     4       18.760  -1.428  12.074  1.00
92.94           C
ATOM     41  C   SER     4       17.329  -0.910  11.982  1.00
67.37           C
ATOM     42  O   SER     4       16.724  -0.904  10.910
1.00102.28           O
ATOM     43  CB  SER     4       18.741  -2.931  12.388
1.00123.83           C
ATOM     44  OG  SER     4       18.690  -3.663  11.171
1.00178.89           O
ATOM     45  HN  SER     4       20.159  -0.034  12.851
1.00163.03           H
ATOM     46  HA  SER     4       19.250  -1.277  11.124
1.00129.78           H
ATOM     47  HB1 SER     4       17.874  -3.168  13.000
1.00105.26           H
ATOM     48  HB2 SER     4       19.637  -3.199  12.924
1.00156.87           H
ATOM     49  HG  SER     4       17.801  -4.010  11.070
1.00218.16           H
```

```
ATOM     50  N   GLU     5       16.790  -0.494  13.123  1.00
41.67           N
ATOM     51  CA  GLU     5       15.422   0.008  13.185  1.00
41.21           C
ATOM     52  C   GLU     5       15.277   1.034  14.305  1.00
41.92           C
ATOM     53  O   GLU     5       16.167   1.183  15.139  1.00
74.72           O
ATOM     54  CB  GLU     5       14.464  -1.157  13.416  1.00
49.24           C
ATOM     55  CG  GLU     5       14.867  -1.882  14.693  1.00
55.48           C
ATOM     56  CD  GLU     5       14.062  -3.168  14.843  1.00
92.06           C
ATOM     57  OE1 GLU     5       13.249  -3.440  13.975
1.00206.49           O
ATOM     58  OE2 GLU     5       14.272  -3.864  15.823
1.00197.24           O1-
ATOM     59  HN  GLU     5       17.321  -0.538  13.945  1.00
45.52           H
ATOM     60  HA  GLU     5       15.175   0.473  12.252  1.00
60.63           H
ATOM     61  HB1 GLU     5       14.518  -1.842  12.583  1.00
61.59           H
ATOM     62  HB2 GLU     5       13.459  -0.784  13.513  1.00
63.66           H
ATOM     63  HG1 GLU     5       14.683  -1.243  15.542  1.00
58.03           H
ATOM     64  HG2 GLU     5       15.916  -2.115  14.641  1.00
57.77           H
ATOM     65  N   ASN     6       14.155   1.743  14.313  1.00
50.73           N
ATOM     66  CA  ASN     6       13.915   2.759  15.331  1.00
71.04           C
ATOM     67  C   ASN     6       13.912   2.142  16.727  1.00
65.17           C
ATOM     68  O   ASN     6       14.435   2.729  17.674  1.00
94.86           O
ATOM     69  CB  ASN     6       12.572   3.448  15.077  1.00
94.83           C
ATOM     70  CG  ASN     6       12.668   4.342  13.847
1.00160.94           C
ATOM     71  ND2 ASN     6       11.577   4.702  13.228
1.00244.88           N
ATOM     72  OD1 ASN     6       13.766   4.721  13.438
1.00219.73           O
ATOM     73  HN  ASN     6       13.480   1.588  13.619  1.00
72.81           H
ATOM     74  HA  ASN     6       14.699   3.499  15.277  1.00
96.02           H
ATOM     75  HB1 ASN     6       12.308   4.048  15.936
1.00111.09           H
```

```
ATOM     76  HB2 ASN     6      11.810   2.698  14.918  1.00
98.13           H
ATOM     77 HD21 ASN     6      10.705   4.400  13.556
1.00272.43           H
ATOM     78 HD22 ASN     6      11.630   5.277  12.436
1.00336.78           H
ATOM     79  N   GLY     7      13.318   0.955  16.850  1.00
51.80           N
ATOM     80  CA  GLY     7      13.248   0.260  18.140  1.00
71.47           C
ATOM     81  C   GLY     7      14.024  -1.049  18.102  1.00
41.04           C
ATOM     82  O   GLY     7      13.442  -2.132  18.164  1.00
44.54           O
ATOM     83  HN  GLY     7      12.917   0.538  16.061  1.00
45.49           H
ATOM     84  HA1 GLY     7      12.219   0.044  18.362
1.00103.52           H
ATOM     85  HA2 GLY     7      13.653   0.889  18.922
1.00108.67           H
ATOM     86  N   ASP     8      15.340  -0.940  18.002  1.00
27.00           N
ATOM     87  CA  ASP     8      16.194  -2.123  17.955  1.00
14.15           C
ATOM     88  C   ASP     8      16.078  -2.920  19.248  1.00
9.37           C
ATOM     89  O   ASP     8      15.894  -4.137  19.228  1.00
14.61           O
ATOM     90  CB  ASP     8      17.651  -1.712  17.748  1.00
15.06           C
ATOM     91  CG  ASP     8      18.515  -2.951  17.538  1.00
21.22           C
ATOM     92  OD1 ASP     8      18.017  -4.042  17.768
1.00119.56           O
ATOM     93  OD2 ASP     8      19.661  -2.795  17.152
1.00133.27           O1-
ATOM     94  HN  ASP     8      15.744  -0.050  17.957  1.00
35.63           H
ATOM     95  HA  ASP     8      15.887  -2.744  17.134  1.00
20.92           H
ATOM     96  HB1 ASP     8      18.000  -1.179  18.619  1.00
29.39           H
ATOM     97  HB2 ASP     8      17.723  -1.070  16.883  1.00
44.14           H
ATOM     98  N   CYS     9      16.191  -2.223  20.368  1.00
5.93           N
ATOM     99  CA  CYS     9      16.099  -2.867  21.671  1.00
7.54           C
ATOM    100  C   CYS     9      14.706  -3.457  21.862  1.00
14.60           C
ATOM    101  O   CYS     9      14.545  -4.571  22.364  1.00
25.78           O
```

```
ATOM    102  CB  CYS     9      16.392  -1.850  22.764  1.00
6.63          C
ATOM    103  SG  CYS     9      18.127  -1.388  22.651  1.00
10.91         S
ATOM    104  HN  CYS     9      16.336  -1.256  20.316  1.00
6.51          H
ATOM    105  HA  CYS     9      16.836  -3.650  21.726  1.00
11.12         H
ATOM    106  HB1 CYS     9      16.207  -2.285  23.731  1.00
11.28         H
ATOM    107  HB2 CYS     9      15.772  -0.977  22.630  1.00
5.04          H
ATOM    108  N   ALA    10      13.707  -2.694  21.437  1.00
16.26         N
ATOM    109  CA  ALA    10      12.312  -3.114  21.529  1.00
32.04         C
ATOM    110  C   ALA    10      11.440  -2.139  20.742  1.00
45.47         C
ATOM    111  O   ALA    10      11.876  -1.036  20.419
1.00119.04         O
ATOM    112  CB  ALA    10      11.859  -3.166  22.991  1.00
30.10         C
ATOM    113  HN  ALA    10      13.915  -1.824  21.038  1.00
12.81         H
ATOM    114  HA  ALA    10      12.215  -4.099  21.094  1.00
47.14         H
ATOM    115  HB1 ALA    10      12.658  -3.562  23.601  1.00
84.37         H
ATOM    116  HB2 ALA    10      10.992  -3.805  23.076
1.00118.20         H
ATOM    117  HB3 ALA    10      11.606  -2.172  23.332
1.00117.32         H
ATOM    118  N   ALA    11      10.216  -2.546  20.425  1.00
30.62         N
ATOM    119  CA  ALA    11       9.320  -1.679  19.663  1.00
35.24         C
ATOM    120  C   ALA    11       9.073  -0.371  20.409  1.00
22.78         C
ATOM    121  O   ALA    11       9.140   0.710  19.821  1.00
53.33         O
ATOM    122  CB  ALA    11       7.988  -2.391  19.421  1.00
60.49         C
ATOM    123  HN  ALA    11       9.914  -3.436  20.698  1.00
50.37         H
ATOM    124  HA  ALA    11       9.775  -1.459  18.709  1.00
41.70         H
ATOM    125  HB1 ALA    11       7.304  -1.717  18.928
1.00157.06         H
ATOM    126  HB2 ALA    11       7.569  -2.702  20.367
1.00148.83         H
ATOM    127  HB3 ALA    11       8.151  -3.258  18.797
1.00137.06         H
```

ATOM 128 N ASP 12 8.795 -0.473 21.703 1.00
18.98 N
ATOM 129 CA ASP 12 8.548 0.713 22.518 1.00
31.05 C
ATOM 130 C ASP 12 9.840 1.494 22.747 1.00
22.98 C
ATOM 131 O ASP 12 9.839 2.724 22.769 1.00
37.14 O
ATOM 132 CB ASP 12 7.954 0.301 23.866 1.00
48.35 C
ATOM 133 CG ASP 12 6.523 -0.194 23.678
1.00104.83 C
ATOM 134 OD1 ASP 12 5.997 -0.789 24.602
1.00258.73 O
ATOM 135 OD2 ASP 12 5.977 0.030 22.610
1.00213.46 O1-
ATOM 136 HN ASP 12 8.761 -1.359 22.119 1.00
36.19 H
ATOM 137 HA ASP 12 7.840 1.347 22.006 1.00
49.76 H
ATOM 138 HB1 ASP 12 7.952 1.152 24.531 1.00
95.71 H
ATOM 139 HB2 ASP 12 8.553 -0.488 24.296 1.00
54.37 H
ATOM 140 N GLU 13 10.934 0.762 22.931 1.00
17.60 N
ATOM 141 CA GLU 13 12.235 1.380 23.179 1.00
12.01 C
ATOM 142 C GLU 13 12.796 2.014 21.907 1.00
10.07 C
ATOM 143 O GLU 13 12.366 1.691 20.799 1.00
12.81 O
ATOM 144 CB GLU 13 13.203 0.320 23.704 1.00
11.74 C
ATOM 145 CG GLU 13 12.740 -0.136 25.093 1.00
13.49 C
ATOM 146 CD GLU 13 13.457 -1.419 25.494
1.00142.36 C
ATOM 147 OE1 GLU 13 14.089 -2.010 24.639
1.00339.06 O
ATOM 148 OE2 GLU 13 13.357 -1.795 26.649
1.00335.28 O1-
ATOM 149 HN GLU 13 10.865 -0.214 22.913 1.00
28.36 H
ATOM 150 HA GLU 13 12.121 2.146 23.930 1.00
13.06 H
ATOM 151 HB1 GLU 13 14.194 0.742 23.778 1.00
13.76 H
ATOM 152 HB2 GLU 13 13.219 -0.523 23.030 1.00
9.94 H
ATOM 153 HG1 GLU 13 11.675 -0.314 25.075 1.00
66.02 H

```
ATOM    154  HG2 GLU    13       12.962   0.637  25.815  1.00
56.23           H
ATOM    155  N   CYS    14       13.759   2.925  22.078  1.00
9.12           N
ATOM    156  CA  CYS    14       14.384   3.617  20.947  1.00
10.69           C
ATOM    157  C   CYS    14       15.832   3.169  20.784  1.00
9.56           C
ATOM    158  O   CYS    14       16.440   2.664  21.723  1.00
13.62           O
ATOM    159  CB  CYS    14       14.343   5.130  21.179  1.00
13.96           C
ATOM    160  SG  CYS    14       15.130   5.978  19.785  1.00
42.96           S
ATOM    161  HN  CYS    14       14.056   3.138  22.987  1.00
9.76           H
ATOM    162  HA  CYS    14       13.843   3.391  20.037  1.00
15.54           H
ATOM    163  HB1 CYS    14       14.872   5.368  22.090  1.00
50.72           H
ATOM    164  HB2 CYS    14       13.316   5.454  21.263  1.00
46.13           H
ATOM    165  N   CYS    15       16.376   3.366  19.585  1.00
9.60           N
ATOM    166  CA  CYS    15       17.759   2.988  19.297  1.00
9.28           C
ATOM    167  C   CYS    15       18.337   3.936  18.255  1.00
9.92           C
ATOM    168  O   CYS    15       17.719   4.173  17.218  1.00
13.31           O
ATOM    169  CB  CYS    15       17.816   1.554  18.767  1.00
12.90           C
ATOM    170  SG  CYS    15       19.544   1.034  18.591  1.00
39.11           S
ATOM    171  HN  CYS    15       15.838   3.779  18.877  1.00
13.12           H
ATOM    172  HA  CYS    15       18.348   3.056  20.203  1.00
8.26           H
ATOM    173  HB1 CYS    15       17.326   1.507  17.805  1.00
24.82           H
ATOM    174  HB2 CYS    15       17.311   0.895  19.459  1.00
27.31           H
ATOM    175  N   VAL    16       19.520   4.480  18.527  1.00
9.56           N
ATOM    176  CA  VAL    16       20.151   5.403  17.588  1.00
11.99           C
ATOM    177  C   VAL    16       21.666   5.299  17.678  1.00
7.73           C
ATOM    178  O   VAL    16       22.240   5.351  18.766  1.00
8.83           O
ATOM    179  CB  VAL    16       19.706   6.833  17.889  1.00
18.37           C
```

```
ATOM    180  CG1 VAL    16       20.223   7.257  19.264  1.00
39.61           C
ATOM    181  CG2 VAL    16       20.262   7.775  16.819
1.00115.21           C
ATOM    182  HN  VAL    16       19.977   4.259  19.368  1.00
9.76            H
ATOM    183  HA  VAL    16       19.848   5.149  16.580  1.00
17.27           H
ATOM    184  HB  VAL    16       18.626   6.881  17.884  1.00
52.63           H
ATOM    185 HG11 VAL    16       21.284   7.456  19.203
1.00128.07           H
ATOM    186 HG12 VAL    16       20.046   6.465  19.974
1.00154.13           H
ATOM    187 HG13 VAL    16       19.707   8.150  19.584
1.00135.74           H
ATOM    188 HG21 VAL    16       21.341   7.779  16.869
1.00229.05           H
ATOM    189 HG22 VAL    16       19.887   8.774  16.990
1.00261.82           H
ATOM    190 HG23 VAL    16       19.947   7.435  15.844
1.00210.07           H
ATOM    191  N   ASP    17       22.309   5.144  16.526  1.00
14.34           N
ATOM    192  CA  ASP    17       23.763   5.023  16.475  1.00
11.90           C
ATOM    193  C   ASP    17       24.411   6.384  16.255  1.00
13.15           C
ATOM    194  O   ASP    17       24.183   7.038  15.236  1.00
25.96           O
ATOM    195  CB  ASP    17       24.162   4.083  15.336  1.00
20.80           C
ATOM    196  CG  ASP    17       23.520   2.716  15.544  1.00
28.72           C
ATOM    197  OD1 ASP    17       22.910   2.223  14.609
1.00135.42           O
ATOM    198  OD2 ASP    17       23.648   2.182  16.633
1.00114.17           O1-
ATOM    199  HN  ASP    17       21.796   5.102  15.690  1.00
28.29           H
ATOM    200  HA  ASP    17       24.123   4.610  17.406  1.00
10.49           H
ATOM    201  HB1 ASP    17       25.236   3.974  15.321  1.00
22.42           H
ATOM    202  HB2 ASP    17       23.829   4.497  14.395  1.00
29.52           H
ATOM    203  N   THR    18       25.236   6.800  17.215  1.00
11.55           N
ATOM    204  CA  THR    18       25.939   8.080  17.128  1.00
17.10           C
ATOM    205  C   THR    18       27.395   7.848  16.756  1.00
10.63           C
```

ATOM 206 O THR 18 27.911 6.743 16.914 1.00
6.49 O
ATOM 207 CB THR 18 25.870 8.811 18.472 1.00
28.52 C
ATOM 208 CG2 THR 18 24.452 9.325 18.708 1.00
45.38 C
ATOM 209 OG1 THR 18 26.236 7.918 19.511 1.00
26.49 O
ATOM 210 HN THR 18 25.382 6.227 17.996 1.00
13.96 H
ATOM 211 HA THR 18 25.477 8.699 16.371 1.00
26.43 H
ATOM 212 HB THR 18 26.552 9.647 18.459 1.00
37.43 H
ATOM 213 HG1 THR 18 25.477 7.368 19.710 1.00
71.62 H
ATOM 214 HG21 THR 18 24.275 10.189 18.085
1.00100.44 H
ATOM 215 HG22 THR 18 24.341 9.602 19.745
1.00129.42 H
ATOM 216 HG23 THR 18 23.741 8.551 18.463
1.00156.21 H
ATOM 217 N VAL 19 28.052 8.899 16.278 1.00
16.32 N
ATOM 218 CA VAL 19 29.442 8.823 15.896 1.00
13.34 C
ATOM 219 C VAL 19 29.895 10.173 15.383 1.00
25.88 C
ATOM 220 O VAL 19 29.389 10.692 14.387 1.00
42.11 O
ATOM 221 CB VAL 19 29.679 7.760 14.824 1.00
15.48 C
ATOM 222 CG1 VAL 19 28.736 7.982 13.637 1.00
29.06 C
ATOM 223 CG2 VAL 19 31.136 7.853 14.350 1.00
20.67 C
ATOM 224 HN VAL 19 27.596 9.757 16.195 1.00
26.89 H
ATOM 225 HA VAL 19 30.026 8.572 16.769 1.00
8.08 H
ATOM 226 HB VAL 19 29.503 6.786 15.248 1.00
11.49 H
ATOM 227 HG11 VAL 19 29.102 8.799 13.031
1.00137.43 H
ATOM 228 HG12 VAL 19 27.747 8.220 14.000 1.00
93.29 H
ATOM 229 HG13 VAL 19 28.694 7.084 13.039
1.00108.69 H
ATOM 230 HG21 VAL 19 31.400 6.959 13.807 1.00
99.42 H
ATOM 231 HG22 VAL 19 31.788 7.963 15.208
1.00111.36 H

```
ATOM    232 HG23 VAL    19      31.249   8.715  13.707  1.00
77.11           H
ATOM    233  N   PHE    20      30.838  10.733  16.097  1.00
24.58           N
ATOM    234  CA  PHE    20      31.389  12.039  15.778  1.00
40.34           C
ATOM    235  C   PHE    20      32.762  11.917  15.138  1.00
40.37           C
ATOM    236  O   PHE    20      32.970  12.343  14.002  1.00
66.02           O
ATOM    237  CB  PHE    20      31.493  12.868  17.065  1.00
48.79           C
ATOM    238  CG  PHE    20      31.873  11.989  18.246  1.00
35.75           C
ATOM    239  CD1 PHE    20      30.974  11.025  18.743  1.00
30.20           C
ATOM    240  CD2 PHE    20      33.120  12.156  18.864  1.00
40.95           C
ATOM    241  CE1 PHE    20      31.329  10.240  19.843  1.00
30.41           C
ATOM    242  CE2 PHE    20      33.470  11.364  19.964  1.00
45.84           C
ATOM    243  CZ  PHE    20      32.575  10.406  20.452  1.00
40.81           C
ATOM    244  HN  PHE    20      31.159  10.257  16.881  1.00
16.73           H
ATOM    245  HA  PHE    20      30.731  12.553  15.087  1.00
59.91           H
ATOM    246  HB1 PHE    20      30.549  13.317  17.259  1.00
67.15           H
ATOM    247  HB2 PHE    20      32.231  13.648  16.941  1.00
58.17           H
ATOM    248  HD1 PHE    20      30.008  10.882  18.278  1.00
33.34           H
ATOM    249  HD2 PHE    20      33.813  12.894  18.489  1.00
49.35           H
ATOM    250  HE1 PHE    20      30.638   9.502  20.220  1.00
30.61           H
ATOM    251  HE2 PHE    20      34.430  11.495  20.437  1.00
61.27           H
ATOM    252  HZ  PHE    20      32.848   9.797  21.302  1.00
52.66           H
ATOM    253  N   GLU    21      33.706  11.372  15.891  1.00
41.31           N
ATOM    254  CA  GLU    21      35.067  11.238  15.424  1.00
56.16           C
ATOM    255  C   GLU    21      35.386   9.814  15.002  1.00
58.61           C
ATOM    256  O   GLU    21      34.532   8.928  15.034
1.00201.19           O
ATOM    257  CB  GLU    21      35.974  11.670  16.555  1.00
57.37           C
```

ATOM 258 CG GLU 21 35.899 10.663 17.704
1.00193.56 C
ATOM 259 CD GLU 21 36.618 11.223 18.928
1.00304.62 C
ATOM 260 OE1 GLU 21 36.542 10.599 19.975
1.00451.81 O
ATOM 261 OE2 GLU 21 37.235 12.266 18.801
1.00442.88 O1-
ATOM 262 HN GLU 21 33.496 11.083 16.800 1.00
50.82 H
ATOM 263 HA GLU 21 35.233 11.898 14.583 1.00
85.72 H
ATOM 264 HB1 GLU 21 35.640 12.631 16.906 1.00
46.37 H
ATOM 265 HB2 GLU 21 36.981 11.743 16.200
1.00131.81 H
ATOM 266 HG1 GLU 21 36.369 9.742 17.404
1.00314.91 H
ATOM 267 HG2 GLU 21 34.866 10.472 17.947
1.00266.92 H
ATOM 268 N GLY 22 36.631 9.617 14.592 1.00
86.70 N
ATOM 269 CA GLY 22 37.097 8.303 14.141
1.00104.66 C
ATOM 270 C GLY 22 37.745 7.521 15.278 1.00
71.50 C
ATOM 271 O GLY 22 38.035 6.333 15.140 1.00
89.87 O
ATOM 272 HN GLY 22 37.252 10.378 14.595
1.00214.64 H
ATOM 273 HA1 GLY 22 37.823 8.440 13.353
1.00152.47 H
ATOM 274 HA2 GLY 22 36.260 7.738 13.754
1.00118.15 H
ATOM 275 N ASP 23 37.975 8.195 16.396 1.00
44.68 N
ATOM 276 CA ASP 23 38.596 7.555 17.550 1.00
43.33 C
ATOM 277 C ASP 23 37.745 6.397 18.053 1.00
37.55 C
ATOM 278 O ASP 23 38.258 5.321 18.362 1.00
61.37 O
ATOM 279 CB ASP 23 38.779 8.574 18.673 1.00
40.74 C
ATOM 280 CG ASP 23 39.551 7.947 19.827
1.00141.86 C
ATOM 281 OD1 ASP 23 39.912 8.674 20.738
1.00328.18 O
ATOM 282 OD2 ASP 23 39.771 6.747 19.785
1.00304.58 O1-
ATOM 283 HN ASP 23 37.730 9.141 16.445 1.00
40.86 H

ATOM 284 HA ASP 23 39.563 7.177 17.262 1.00
72.12 H
ATOM 285 HB1 ASP 23 37.808 8.892 19.024 1.00
94.15 H
ATOM 286 HB2 ASP 23 39.322 9.428 18.297 1.00
93.26 H
ATOM 287 N MET 24 36.442 6.628 18.141 1.00
23.24 N
ATOM 288 CA MET 24 35.521 5.601 18.618 1.00
32.50 C
ATOM 289 C MET 24 34.092 5.899 18.175 1.00
25.13 C
ATOM 290 O MET 24 33.801 6.979 17.663 1.00
54.40 O
ATOM 291 CB MET 24 35.581 5.516 20.145 1.00
47.36 C
ATOM 292 CG MET 24 35.163 6.858 20.752
1.00151.73 C
ATOM 293 SD MET 24 35.345 6.788 22.551
1.00209.23 S
ATOM 294 CE MET 24 34.735 8.454 22.907
1.00243.29 C
ATOM 295 HN MET 24 36.095 7.506 17.885 1.00
17.32 H
ATOM 296 HA MET 24 35.820 4.647 18.208 1.00
48.94 H
ATOM 297 HB1 MET 24 36.589 5.284 20.453
1.00124.40 H
ATOM 298 HB2 MET 24 34.912 4.741 20.488
1.00166.96 H
ATOM 299 HG1 MET 24 34.131 7.060 20.504
1.00331.71 H
ATOM 300 HG2 MET 24 35.789 7.643 20.357
1.00302.30 H
ATOM 301 HE1 MET 24 35.190 8.817 23.818
1.00340.44 H
ATOM 302 HE2 MET 24 33.664 8.428 23.029
1.00373.88 H
ATOM 303 HE3 MET 24 34.986 9.111 22.086
1.00386.81 H
ATOM 304 N VAL 25 33.204 4.926 18.380 1.00
22.44 N
ATOM 305 CA VAL 25 31.792 5.069 18.007 1.00
15.37 C
ATOM 306 C VAL 25 30.900 4.954 19.238 1.00
17.47 C
ATOM 307 O VAL 25 31.184 4.189 20.160 1.00
29.52 O
ATOM 308 CB VAL 25 31.405 3.991 16.989 1.00
23.36 C
ATOM 309 CG1 VAL 25 29.888 4.029 16.740 1.00
58.39 C

```
ATOM    310  CG2 VAL    25       32.152   4.252  15.676  1.00
55.25           C
ATOM    311  HN  VAL    25       33.502   4.090  18.794  1.00
48.11           H
ATOM    312  HA  VAL    25       31.632   6.044  17.558  1.00
9.25           H
ATOM    313  HB  VAL    25       31.682   3.019  17.373  1.00
52.45           H
ATOM    314 HG11 VAL    25       29.662   3.593  15.778
1.00171.07           H
ATOM    315 HG12 VAL    25       29.541   5.052  16.763
1.00166.22           H
ATOM    316 HG13 VAL    25       29.385   3.468  17.514
1.00134.76           H
ATOM    317 HG21 VAL    25       31.806   3.561  14.922
1.00141.88           H
ATOM    318 HG22 VAL    25       33.212   4.115  15.830
1.00184.50           H
ATOM    319 HG23 VAL    25       31.963   5.264  15.350
1.00133.90           H
ATOM    320  N   THR    26       29.825   5.738  19.245  1.00
13.60           N
ATOM    321  CA  THR    26       28.881   5.756  20.363  1.00
21.19           C
ATOM    322  C   THR    26       27.490   5.298  19.924  1.00
16.55           C
ATOM    323  O   THR    26       26.973   5.738  18.897  1.00
11.43           O
ATOM    324  CB  THR    26       28.799   7.178  20.908  1.00
27.07           C
ATOM    325  CG2 THR    26       27.774   7.245  22.044  1.00
44.39           C
ATOM    326  OG1 THR    26       30.077   7.563  21.398  1.00
34.75           O
ATOM    327  HN  THR    26       29.667   6.331  18.482  1.00
10.81           H
ATOM    328  HA  THR    26       29.233   5.104  21.150  1.00
33.16           H
ATOM    329  HB  THR    26       28.503   7.847  20.110  1.00
20.60           H
ATOM    330  HG1 THR    26       29.952   7.986  22.250  1.00
77.72           H
ATOM    331 HG21 THR    26       26.779   7.121  21.643
1.00126.99           H
ATOM    332 HG22 THR    26       27.846   8.203  22.537
1.00108.32           H
ATOM    333 HG23 THR    26       27.977   6.459  22.756
1.00151.99           H
ATOM    334  N   ARG    27       26.886   4.416  20.723  1.00
22.38           N
ATOM    335  CA  ARG    27       25.545   3.895  20.438  1.00
20.21           C
```

```
ATOM    336  C   ARG    27       24.641   4.108  21.651  1.00
16.96           C
ATOM    337  O   ARG    27       25.106   4.066  22.789  1.00
20.06           O
ATOM    338  CB  ARG    27       25.620   2.399  20.122  1.00
22.14           C
ATOM    339  CG  ARG    27       26.499   2.176  18.889
1.00124.30           C
ATOM    340  CD  ARG    27       26.747   0.678  18.706
1.00109.38           C
ATOM    341  NE  ARG    27       27.517   0.152  19.829
1.00227.73           N
ATOM    342  CZ  ARG    27       27.790  -1.145  19.928
1.00426.12           C
ATOM    343  NH1 ARG    27       28.477  -1.592  20.944
1.00767.09           N1+
ATOM    344  NH2 ARG    27       27.372  -1.972  19.008
1.00581.78           N
ATOM    345  HN  ARG    27       27.351   4.112  21.531  1.00
30.75           H
ATOM    346  HA  ARG    27       25.122   4.414  19.588  1.00
23.13           H
ATOM    347  HB1 ARG    27       24.629   2.022  19.927  1.00
87.46           H
ATOM    348  HB2 ARG    27       26.046   1.875  20.967
1.00103.36           H
ATOM    349  HG1 ARG    27       27.442   2.682  19.022
1.00281.97           H
ATOM    350  HG2 ARG    27       25.999   2.568  18.015
1.00276.19           H
ATOM    351  HD1 ARG    27       27.295   0.516  17.788
1.00183.60           H
ATOM    352  HD2 ARG    27       25.802   0.162  18.652
1.00142.93           H
ATOM    353  HE  ARG    27       27.835   0.766  20.523
1.00372.53           H
ATOM    354 HH11 ARG    27       28.797  -0.958  21.648
1.00910.59           H
ATOM    355 HH12 ARG    27       28.683  -2.567  21.018
1.00999.99           H
ATOM    356 HH21 ARG    27       26.845  -1.629  18.231
1.00532.54           H
ATOM    357 HH22 ARG    27       27.577  -2.947  19.082
1.00948.84           H
ATOM    358  N   SER    28       23.348   4.344  21.409  1.00
14.60           N
ATOM    359  CA  SER    28       22.400   4.569  22.506  1.00
14.92           C
ATOM    360  C   SER    28       21.115   3.778  22.280  1.00
12.12           C
ATOM    361  O   SER    28       20.746   3.484  21.144  1.00
13.41           O
```

ATOM 362 CB SER 28 22.071 6.057 22.612 1.00
23.26 C
ATOM 363 OG SER 28 23.266 6.783 22.869
1.00146.32 O
ATOM 364 HN SER 28 23.024 4.372 20.483 1.00
15.23 H
ATOM 365 HA SER 28 22.844 4.246 23.439 1.00
15.44 H
ATOM 366 HB1 SER 28 21.366 6.210 23.418 1.00
88.73 H
ATOM 367 HB2 SER 28 21.638 6.400 21.689
1.00124.68 H
ATOM 368 HG SER 28 23.546 7.195 22.046
1.00242.47 H
ATOM 369 N CYS 29 20.439 3.441 23.374 1.00
10.48 N
ATOM 370 CA CYS 29 19.195 2.687 23.290 1.00
9.97 C
ATOM 371 C CYS 29 18.463 2.738 24.630 1.00
11.60 C
ATOM 372 O CYS 29 19.062 2.519 25.683 1.00
15.64 O
ATOM 373 CB CYS 29 19.488 1.233 22.896 1.00
9.52 C
ATOM 374 SG CYS 29 18.064 0.533 22.053 1.00
10.40 S
ATOM 375 HN CYS 29 20.781 3.708 24.253 1.00
11.10 H
ATOM 376 HA CYS 29 18.566 3.135 22.535 1.00
11.08 H
ATOM 377 HB1 CYS 29 19.692 0.641 23.778 1.00
9.33 H
ATOM 378 HB2 CYS 29 20.340 1.198 22.238 1.00
10.78 H
ATOM 379 N GLU 30 17.170 3.037 24.582 1.00
13.52 N
ATOM 380 CA GLU 30 16.369 3.126 25.798 1.00
17.41 C
ATOM 381 C GLU 30 15.923 1.749 26.273 1.00
10.81 C
ATOM 382 O GLU 30 15.819 0.810 25.485 1.00
23.22 O
ATOM 383 CB GLU 30 15.135 3.991 25.547 1.00
37.83 C
ATOM 384 CG GLU 30 15.566 5.398 25.143
1.00108.13 C
ATOM 385 CD GLU 30 16.211 6.104 26.331
1.00240.52 C
ATOM 386 OE1 GLU 30 16.083 5.601 27.435
1.00422.56 O
ATOM 387 OE2 GLU 30 16.824 7.138 26.121
1.00410.81 O1-

ATOM 388 HN GLU 30 16.749 3.209 23.715 1.00
15.61 H
ATOM 389 HA GLU 30 16.960 3.588 26.574 1.00
24.60 H
ATOM 390 HB1 GLU 30 14.542 4.042 26.449 1.00
72.67 H
ATOM 391 HB2 GLU 30 14.546 3.553 24.753 1.00
56.47 H
ATOM 392 HG1 GLU 30 14.701 5.956 24.823
1.00196.66 H
ATOM 393 HG2 GLU 30 16.276 5.337 24.332
1.00140.67 H
ATOM 394 N LYS 31 15.644 1.651 27.572 1.00
11.96 N
ATOM 395 CA LYS 31 15.185 0.398 28.174 1.00
11.40 C
ATOM 396 C LYS 31 13.855 0.633 28.880 1.00
10.99 C
ATOM 397 O LYS 31 13.635 1.691 29.469 1.00
12.86 O
ATOM 398 CB LYS 31 16.217 -0.109 29.182 1.00
21.40 C
ATOM 399 CG LYS 31 17.528 -0.425 28.458 1.00
57.81 C
ATOM 400 CD LYS 31 18.555 -0.946 29.465
1.00115.52 C
ATOM 401 CE LYS 31 19.859 -1.280 28.739
1.00250.23 C
ATOM 402 NZ LYS 31 20.421 -0.039 28.134
1.00462.10 N1+
ATOM 403 HN LYS 31 15.737 2.444 28.140 1.00
26.13 H
ATOM 404 HA LYS 31 15.048 -0.349 27.405 1.00
13.87 H
ATOM 405 HB1 LYS 31 15.845 -1.004 29.658 1.00
37.63 H
ATOM 406 HB2 LYS 31 16.390 0.650 29.931 1.00
47.19 H
ATOM 407 HG1 LYS 31 17.906 0.471 27.992
1.00126.32 H
ATOM 408 HG2 LYS 31 17.348 -1.177 27.703
1.00114.19 H
ATOM 409 HD1 LYS 31 18.173 -1.837 29.941
1.00198.45 H
ATOM 410 HD2 LYS 31 18.742 -0.189 30.214
1.00200.14 H
ATOM 411 HE1 LYS 31 19.663 -2.002 27.960
1.00374.64 H
ATOM 412 HE2 LYS 31 20.568 -1.692 29.442
1.00403.27 H
ATOM 413 HZ1 LYS 31 20.650 0.640 28.887
1.00627.38 H

```
ATOM    414  HZ2 LYS    31      19.721   0.379  27.489
1.00622.26            H
ATOM    415  HZ3 LYS    31      21.286  -0.272  27.606
1.00619.20            H
ATOM    416  N   THR    32      12.967  -0.352  28.813  1.00
15.51           N
ATOM    417  CA  THR    32      11.655  -0.228  29.446  1.00
22.44           C
ATOM    418  C   THR    32      11.724  -0.638  30.913  1.00
26.60           C
ATOM    419  O   THR    32      12.093  -1.766  31.239  1.00
57.43           O
ATOM    420  CB  THR    32      10.638  -1.106  28.714  1.00
56.28           C
ATOM    421  CG2 THR    32       9.275  -0.991  29.399
1.00102.70            C
ATOM    422  OG1 THR    32      10.531  -0.675  27.364
1.00111.17            O
ATOM    423  HN  THR    32      13.191  -1.171  28.326  1.00
18.79           H
ATOM    424  HA  THR    32      11.329   0.801  29.387  1.00
19.67           H
ATOM    425  HB  THR    32      10.962  -2.133  28.739  1.00
84.25           H
ATOM    426  HG1 THR    32       9.852  -1.203  26.938
1.00205.03            H
ATOM    427 HG21 THR    32       9.031   0.052  29.543
1.00220.71            H
ATOM    428 HG22 THR    32       9.312  -1.487  30.357
1.00174.67            H
ATOM    429 HG23 THR    32       8.521  -1.456  28.782
1.00217.44            H
ATOM    430  N   THR    33      11.359   0.290  31.791  1.00
22.47           N
ATOM    431  CA  THR    33      11.374   0.032  33.228  1.00
41.60           C
ATOM    432  C   THR    33      10.350   0.885  33.925  1.00
38.03           C
ATOM    433  O   THR    33      10.641   1.990  34.386  1.00
52.73           O
ATOM    434  CB  THR    33      12.763   0.305  33.810  1.00
63.25           C
ATOM    435  CG2 THR    33      13.752  -0.748  33.304
1.00121.65            C
ATOM    436  OG1 THR    33      13.197   1.597  33.406  1.00
96.20           O
ATOM    437  HN  THR    33      11.073   1.169  31.466  1.00
23.82           H
ATOM    438  HA  THR    33      11.108  -0.990  33.415  1.00
67.12           H
ATOM    439  HB  THR    33      12.714   0.260  34.885
1.00107.59            H
```

```
ATOM    440  HG1 THR    33       13.295   1.593  32.452
1.00176.27           H
ATOM    441 HG21 THR    33       14.669  -0.678  33.868
1.00255.23           H
ATOM    442 HG22 THR    33       13.957  -0.577  32.258
1.00245.37           H
ATOM    443 HG23 THR    33       13.326  -1.733  33.431
1.00185.78           H
ATOM    444  N   GLY    34        9.147   0.349  34.015  1.00
40.93           N
ATOM    445  CA  GLY    34        8.078   1.050  34.677  1.00
45.59           C
ATOM    446  C   GLY    34        7.580   2.216  33.829  1.00
40.97           C
ATOM    447  O   GLY    34        6.381   2.353  33.585  1.00
82.17           O
ATOM    448  HN  GLY    34        8.984  -0.541  33.639  1.00
54.00           H
ATOM    449  HA1 GLY    34        8.457   1.421  35.607  1.00
54.41           H
ATOM    450  HA2 GLY    34        7.262   0.365  34.865  1.00
62.09           H
ATOM    451  N   ASN    35        8.514   3.054  33.375  1.00
32.51           N
ATOM    452  CA  ASN    35        8.173   4.212  32.545  1.00
33.20           C
ATOM    453  C   ASN    35        9.074   4.270  31.316  1.00
23.17           C
ATOM    454  O   ASN    35       10.280   4.040  31.408  1.00
27.01           O
ATOM    455  CB  ASN    35        8.337   5.500  33.354  1.00
54.51           C
ATOM    456  CG  ASN    35        7.414   5.474  34.568  1.00
80.73           C
ATOM    457  ND2 ASN    35        7.869   5.855  35.730
1.00220.46           N
ATOM    458  OD1 ASN    35        6.247   5.098  34.454
1.00117.80           O
ATOM    459  HN  ASN    35        9.454   2.889  33.600  1.00
51.15           H
ATOM    460  HA  ASN    35        7.144   4.136  32.218  1.00
47.21           H
ATOM    461  HB1 ASN    35        8.087   6.347  32.733  1.00
68.31           H
ATOM    462  HB2 ASN    35        9.361   5.586  33.684  1.00
57.75           H
ATOM    463 HD21 ASN    35        8.798   6.154  35.819
1.00405.39           H
ATOM    464 HD22 ASN    35        7.283   5.839  36.515
1.00245.64           H
ATOM    465  N   PHE    36        8.481   4.573  30.166  1.00
35.72           N
```

```
ATOM    466  CA  PHE    36       9.226   4.656  28.921  1.00
33.41           C
ATOM    467  C   PHE    36       9.867   6.034  28.772  1.00
28.51           C
ATOM    468  O   PHE    36       9.334   7.030  29.262  1.00
45.23           O
ATOM    469  CB  PHE    36       8.275   4.407  27.757  1.00
67.53           C
ATOM    470  CG  PHE    36       7.703   3.012  27.858  1.00
86.71           C
ATOM    471  CD1 PHE    36       6.526   2.796  28.586
1.00106.51           C
ATOM    472  CD2 PHE    36       8.342   1.939  27.224  1.00
99.48           C
ATOM    473  CE1 PHE    36       5.986   1.507  28.679
1.00134.48           C
ATOM    474  CE2 PHE    36       7.802   0.650  27.318
1.00134.27           C
ATOM    475  CZ  PHE    36       6.625   0.435  28.045
1.00149.48           C
ATOM    476  HN  PHE    36       7.518   4.742  30.150  1.00
62.87           H
ATOM    477  HA  PHE    36       9.998   3.902  28.912  1.00
28.53           H
ATOM    478  HB1 PHE    36       8.809   4.507  26.832  1.00
73.56           H
ATOM    479  HB2 PHE    36       7.471   5.128  27.788  1.00
88.42           H
ATOM    480  HD1 PHE    36       6.035   3.623  29.076
1.00110.20           H
ATOM    481  HD2 PHE    36       9.251   2.103  26.665  1.00
93.07           H
ATOM    482  HE1 PHE    36       5.080   1.340  29.239
1.00155.06           H
ATOM    483  HE2 PHE    36       8.293  -0.177  26.829
1.00159.46           H
ATOM    484  HZ  PHE    36       6.210  -0.560  28.116
1.00183.25           H
ATOM    485  N   THR    37      11.013   6.088  28.089  1.00
20.06           N
ATOM    486  CA  THR    37      11.724   7.349  27.875  1.00
27.18           C
ATOM    487  C   THR    37      11.958   7.574  26.385  1.00
41.87           C
ATOM    488  O   THR    37      11.126   7.212  25.554
1.00169.86           O
ATOM    489  CB  THR    37      13.068   7.317  28.606  1.00
26.52           C
ATOM    490  CG2 THR    37      12.846   6.943  30.072  1.00
75.69           C
ATOM    491  OG1 THR    37      13.915   6.357  27.993  1.00
81.95           O
```

ATOM 492 HN THR 37 11.390 5.265 27.720 1.00
18.47 H
ATOM 493 HA THR 37 11.137 8.170 28.263 1.00
38.80 H
ATOM 494 HB THR 37 13.530 8.290 28.555 1.00
81.60 H
ATOM 495 HG1 THR 37 14.602 6.122 28.621
1.00188.80 H
ATOM 496 HG21 THR 37 12.071 7.569 30.489
1.00196.98 H
ATOM 497 HG22 THR 37 13.762 7.089 30.625
1.00196.23 H
ATOM 498 HG23 THR 37 12.546 5.907 30.137
1.00168.92 H
ATOM 499 N GLU 38 13.094 8.178 26.057 1.00
26.53 N
ATOM 500 CA GLU 38 13.435 8.456 24.662 1.00
30.29 C
ATOM 501 C GLU 38 14.951 8.510 24.475 1.00
25.39 C
ATOM 502 O GLU 38 15.671 9.056 25.311 1.00
51.60 O
ATOM 503 CB GLU 38 12.819 9.790 24.232 1.00
54.82 C
ATOM 504 CG GLU 38 13.104 10.035 22.747
1.00165.84 C
ATOM 505 CD GLU 38 12.413 11.315 22.287
1.00292.92 C
ATOM 506 OE1 GLU 38 11.756 11.937 23.106
1.00414.19 O
ATOM 507 OE2 GLU 38 12.552 11.653 21.124
1.00537.65 O1-
ATOM 508 HN GLU 38 13.714 8.443 26.766 1.00
84.50 H
ATOM 509 HA GLU 38 13.036 7.671 24.037 1.00
32.15 H
ATOM 510 HB1 GLU 38 13.248 10.591 24.816
1.00144.82 H
ATOM 511 HB2 GLU 38 11.750 9.760 24.393
1.00106.49 H
ATOM 512 HG1 GLU 38 12.734 9.202 22.169
1.00303.31 H
ATOM 513 HG2 GLU 38 14.169 10.130 22.598
1.00323.99 H
ATOM 514 N CYS 39 15.422 7.938 23.371 1.00
15.84 N
ATOM 515 CA CYS 39 16.851 7.925 23.075 1.00
14.30 C
ATOM 516 C CYS 39 17.393 9.360 23.042 1.00
22.31 C
ATOM 517 O CYS 39 16.650 10.296 22.747 1.00
32.92 O

ATOM 518 CB CYS 39 17.084 7.240 21.721 1.00
18.08 C
ATOM 519 SG CYS 39 15.779 7.727 20.566 1.00
17.41 S
ATOM 520 HN CYS 39 14.800 7.521 22.741 1.00
24.87 H
ATOM 521 HA CYS 39 17.358 7.362 23.843 1.00
14.60 H
ATOM 522 HB1 CYS 39 17.065 6.168 21.853 1.00
27.71 H
ATOM 523 HB2 CYS 39 18.043 7.536 21.322 1.00
34.91 H
ATOM 524 N PRO 40 18.658 9.557 23.340 1.00
27.27 N
ATOM 525 CA PRO 40 19.275 10.915 23.343 1.00
47.66 C
ATOM 526 C PRO 40 19.502 11.437 21.926 1.00
71.54 C
ATOM 527 O PRO 40 19.590 10.666 20.971 1.00
87.70 O
ATOM 528 CB PRO 40 20.600 10.703 24.083 1.00
53.85 C
ATOM 529 CG PRO 40 20.967 9.287 23.789 1.00
46.04 C
ATOM 530 CD PRO 40 19.642 8.521 23.703 1.00
25.70 C
ATOM 531 HA PRO 40 18.658 11.604 23.897 1.00
58.04 H
ATOM 532 HB1 PRO 40 20.462 10.836 25.146 1.00
67.93 H
ATOM 533 HB2 PRO 40 21.359 11.382 23.713 1.00
70.99 H
ATOM 534 HG1 PRO 40 21.575 8.881 24.582 1.00
65.91 H
ATOM 535 HG2 PRO 40 21.497 9.230 22.845 1.00
54.99 H
ATOM 536 HD1 PRO 40 19.392 8.085 24.658 1.00
24.22 H
ATOM 537 HD2 PRO 40 19.694 7.766 22.937 1.00
26.94 H
ATOM 538 N GLY 41 19.595 12.756 21.806
1.00105.24 N
ATOM 539 CA GLY 41 19.811 13.401 20.510
1.00147.55 C
ATOM 540 C GLY 41 21.273 13.793 20.332
1.00161.74 C
ATOM 541 O GLY 41 22.079 13.011 19.829
1.00244.37 O
ATOM 542 HN GLY 41 19.516 13.308 22.608
1.00121.79 H
ATOM 543 HA1 GLY 41 19.202 14.290 20.453
1.00184.40 H

ATOM 544 HA2 GLY 41 19.524 12.725 19.714
1.00157.34 H
ATOM 545 N LEU 42 21.606 15.012 20.742
1.00162.48 N
ATOM 546 CA LEU 42 22.975 15.504 20.617
1.00191.48 C
ATOM 547 C LEU 42 23.865 14.904 21.702
1.00166.66 C
ATOM 548 O LEU 42 23.429 14.702 22.836
1.00251.38 O
ATOM 549 CB LEU 42 22.992 17.030 20.732
1.00307.99 C
ATOM 550 CG LEU 42 22.056 17.646 19.685
1.00452.28 C
ATOM 551 CD1 LEU 42 22.056 19.170 19.847
1.00681.24 C
ATOM 552 CD2 LEU 42 22.531 17.277 18.268
1.00527.46 C
ATOM 553 HN LEU 42 20.920 15.594 21.131
1.00195.47 H
ATOM 554 HA LEU 42 23.365 15.222 19.652
1.00229.77 H
ATOM 555 HB1 LEU 42 23.997 17.391 20.570
1.00342.49 H
ATOM 556 HB2 LEU 42 22.664 17.319 21.721
1.00335.33 H
ATOM 557 HG LEU 42 21.054 17.272 19.839
1.00427.79 H
ATOM 558 HD11 LEU 42 23.016 19.565 19.551
1.00909.71 H
ATOM 559 HD12 LEU 42 21.866 19.421 20.880
1.00706.80 H
ATOM 560 HD13 LEU 42 21.283 19.598 19.224
1.00858.82 H
ATOM 561 HD21 LEU 42 22.150 16.300 18.006
1.00631.69 H
ATOM 562 HD22 LEU 42 23.612 17.262 18.236
1.00628.06 H
ATOM 563 HD23 LEU 42 22.161 18.004 17.558
1.00656.75 H
ATOM 564 N THR 43 25.114 14.624 21.343
1.00178.52 N
ATOM 565 CA THR 43 26.062 14.049 22.290
1.00240.84 C
ATOM 566 C THR 43 26.488 15.104 23.318
1.00372.31 C
ATOM 567 O THR 43 26.514 16.295 23.006
1.00500.13 O
ATOM 568 CB THR 43 27.292 13.546 21.525
1.00354.13 C
ATOM 569 CG2 THR 43 26.850 12.572 20.431
1.00463.04 C

ATOM 570 OG1 THR 43 27.964 14.650 20.936
1.00508.50 O
ATOM 571 HN THR 43 25.403 14.809 20.426
1.00229.59 H
ATOM 572 HA THR 43 25.594 13.219 22.790
1.00249.90 H
ATOM 573 HB THR 43 27.961 13.039 22.201
1.00481.58 H
ATOM 574 HG1 THR 43 28.468 14.325 20.186
1.00624.31 H
ATOM 575 HG21 THR 43 26.270 11.776 20.872
1.00650.95 H
ATOM 576 HG22 THR 43 27.719 12.159 19.943
1.00580.73 H
ATOM 577 HG23 THR 43 26.246 13.098 19.705
1.00571.99 H
ATOM 578 N PRO 44 26.829 14.709 24.527
1.00488.39 N
ATOM 579 CA PRO 44 27.263 15.670 25.583
1.00760.13 C
ATOM 580 C PRO 44 28.692 16.156 25.350
1.00735.36 C
ATOM 581 O PRO 44 29.167 17.069 26.025
1.00999.99 O
ATOM 582 CB PRO 44 27.154 14.851 26.873
1.00999.99 C
ATOM 583 CG PRO 44 27.424 13.447 26.445
1.00856.94 C
ATOM 584 CD PRO 44 26.841 13.318 25.031
1.00548.09 C
ATOM 585 HA PRO 44 26.586 16.510 25.627
1.00939.00 H
ATOM 586 HB1 PRO 44 26.158 14.928 27.285
1.00999.99 H
ATOM 587 HB2 PRO 44 27.889 15.181 27.599
1.00999.99 H
ATOM 588 HG1 PRO 44 26.933 12.749 27.108
1.00999.99 H
ATOM 589 HG2 PRO 44 28.490 13.262 26.431
1.00853.53 H
ATOM 590 HD1 PRO 44 25.837 12.925 25.070
1.00602.45 H
ATOM 591 HD2 PRO 44 27.475 12.693 24.420
1.00463.32 H
ATOM 592 N ILE 45 29.372 15.532 24.393
1.00518.33 N
ATOM 593 CA ILE 45 30.747 15.900 24.082
1.00582.79 C
ATOM 594 C ILE 45 30.808 17.312 23.510
1.00795.93 C
ATOM 595 O ILE 45 30.079 17.649 22.577
1.00898.75 O

```
ATOM    596  CB  ILE    45      31.336  14.914  23.071
1.00474.41          C
ATOM    597  CG1 ILE    45      31.384  13.516  23.692
1.00516.90          C
ATOM    598  CG2 ILE    45      32.753  15.353  22.696
1.00760.75          C
ATOM    599  CD1 ILE    45      31.721  12.488  22.611
1.00566.81          C
ATOM    600  HN  ILE    45      28.942  14.808  23.891
1.00395.72          H
ATOM    601  HA  ILE    45      31.331  15.861  24.988
1.00711.06          H
ATOM    602  HB  ILE    45      30.718  14.895  22.184
1.00477.81          H
ATOM    603 HG11 ILE    45      30.421  13.280  24.123
1.00624.28          H
ATOM    604 HG12 ILE    45      32.139  13.491  24.463
1.00744.84          H
ATOM    605 HG21 ILE    45      33.309  15.579  23.594
1.00920.57          H
ATOM    606 HG22 ILE    45      32.705  16.233  22.071
1.00999.99          H
ATOM    607 HG23 ILE    45      33.246  14.558  22.158
1.00865.46          H
ATOM    608 HD11 ILE    45      30.905  12.422  21.908
1.00658.89          H
ATOM    609 HD12 ILE    45      31.879  11.523  23.070
1.00734.30          H
ATOM    610 HD13 ILE    45      32.619  12.791  22.093
1.00675.84          H
ATOM    611  N   ALA    46      31.686  18.135  24.075
1.00999.99          N
ATOM    612  CA  ALA    46      31.838  19.511  23.615
1.00999.99          C
ATOM    613  C   ALA    46      32.083  19.549  22.110
1.00999.99          C
ATOM    614  CB  ALA    46      33.007  20.178  24.341
1.00999.99          C
ATOM    615  OT1 ALA    46      32.851  18.728  21.633
1.00999.99          O
ATOM    616  OT2 ALA    46      31.499  20.396  21.455
1.00999.99          O
ATOM    617  HN  ALA    46      32.242  17.811  24.815
1.00999.99          H
ATOM    618  HA  ALA    46      30.932  20.056  23.836
1.00999.99          H
ATOM    619  HB1 ALA    46      33.936  19.742  24.005
1.00999.99          H
ATOM    620  HB2 ALA    46      32.902  20.031  25.405
1.00999.99          H
ATOM    621  HB3 ALA    46      33.008  21.237  24.123
1.00999.99          H
```

```
ENDMDL
TER
MODEL        18
ATOM      1  N   GLY     1       24.815   4.662  10.387
1.00999.99           N
ATOM      2  CA  GLY     1       24.690   4.279  11.823
1.00999.99           C
ATOM      3  C   GLY     1       23.215   4.205  12.204
1.00999.99           C
ATOM      4  O   GLY     1       22.733   4.996  13.015
1.00999.99           O
ATOM      5  HA1 GLY     1       25.180   5.020  12.436
1.00999.99           H
ATOM      6  HA2 GLY     1       25.153   3.315  11.980
1.00999.99           H
ATOM      7  HT1 GLY     1       24.124   4.126   9.823
1.00999.99           H
ATOM      8  HT2 GLY     1       25.777   4.446  10.054
1.00999.99           H
ATOM      9  HT3 GLY     1       24.629   5.679  10.281
1.00999.99           H
ATOM     10  N   LEU     2       22.502   3.248  11.611
1.00999.99           N
ATOM     11  CA  LEU     2       21.074   3.065  11.888
1.00895.59           C
ATOM     12  C   LEU     2       20.826   1.701  12.526
1.00532.27           C
ATOM     13  O   LEU     2       21.483   0.718  12.182
1.00625.21           O
ATOM     14  CB  LEU     2       20.277   3.163  10.585
1.00999.99           C
ATOM     15  CG  LEU     2       20.562   4.502   9.893
1.00999.99           C
ATOM     16  CD1 LEU     2       19.808   4.551   8.562
1.00999.99           C
ATOM     17  CD2 LEU     2       20.105   5.667  10.789
1.00999.99           C
ATOM     18  HN  LEU     2       22.945   2.648  10.974
1.00999.99           H
ATOM     19  HA  LEU     2       20.730   3.832  12.567
1.00890.87           H
ATOM     20  HB1 LEU     2       19.221   3.093  10.804
1.00924.07           H
ATOM     21  HB2 LEU     2       20.562   2.353   9.929
1.00999.99           H
ATOM     22  HG  LEU     2       21.623   4.586   9.705
1.00999.99           H
ATOM     23 HD11 LEU     2       20.123   5.418   8.001
1.00999.99           H
ATOM     24 HD12 LEU     2       18.746   4.610   8.751
1.00999.99           H
ATOM     25 HD13 LEU     2       20.023   3.658   7.993
```

```
1.00999.99           H
ATOM     26 HD21 LEU     2       19.195   5.396  11.304
1.00999.99           H
ATOM     27 HD22 LEU     2       19.925   6.545  10.183
1.00999.99           H
ATOM     28 HD23 LEU     2       20.876   5.890  11.512
1.00999.99           H
ATOM     29  N   CYS     3       19.873   1.649  13.450
1.00271.28           N
ATOM     30  CA  CYS     3       19.545   0.399  14.124
1.00104.50           C
ATOM     31  C   CYS     3       18.677  -0.474  13.222  1.00
90.98           C
ATOM     32  O   CYS     3       18.394  -0.110  12.080
1.00207.28           O
ATOM     33  CB  CYS     3       18.803   0.682  15.433  1.00
37.83           C
ATOM     34  SG  CYS     3       19.838   1.714  16.502  1.00
81.56           S
ATOM     35  HN  CYS     3       19.380   2.464  13.680
1.00296.55           H
ATOM     36  HA  CYS     3       20.459  -0.130  14.348
1.00166.45           H
ATOM     37  HB1 CYS     3       18.589  -0.251  15.933  1.00
40.38           H
ATOM     38  HB2 CYS     3       17.879   1.195  15.221  1.00
98.45           H
ATOM     39  N   SER     4       18.264  -1.627  13.735  1.00
85.31           N
ATOM     40  CA  SER     4       17.436  -2.544  12.959  1.00
92.94           C
ATOM     41  C   SER     4       16.148  -1.861  12.531  1.00
67.37           C
ATOM     42  O   SER     4       15.707  -1.997  11.390
1.00102.28           O
ATOM     43  CB  SER     4       17.068  -3.760  13.803
1.00123.83           C
ATOM     44  OG  SER     4       16.405  -4.714  12.984
1.00178.89           O
ATOM     45  HN  SER     4       18.525  -1.869  14.649
1.00163.03           H
ATOM     46  HA  SER     4       17.979  -2.870  12.086
1.00129.78           H
ATOM     47  HB1 SER     4       16.406  -3.443  14.603
1.00105.26           H
ATOM     48  HB2 SER     4       17.957  -4.200  14.223
1.00156.87           H
ATOM     49  HG  SER     4       16.467  -5.569  13.415
1.00218.16           H
ATOM     50  N   GLU     5       15.537  -1.148  13.469  1.00
41.67           N
ATOM     51  CA  GLU     5       14.279  -0.465  13.203  1.00
```

```
41.21           C
ATOM     52  C   GLU     5       14.120   0.746  14.107  1.00
41.92           C
ATOM     53  O   GLU     5       13.072   0.947  14.721  1.00
74.72           O
ATOM     54  CB  GLU     5       13.130  -1.434  13.424  1.00
49.24           C
ATOM     55  CG  GLU     5       13.228  -2.026  14.824  1.00
55.48           C
ATOM     56  CD  GLU     5       12.202  -3.141  14.994  1.00
92.06           C
ATOM     57  OE1 GLU     5       11.472  -3.397  14.051
1.00206.49           O
ATOM     58  OE2 GLU     5       12.161  -3.722  16.067
1.00197.24           O1-
ATOM     59  HN  GLU     5       15.931  -1.096  14.364  1.00
45.52           H
ATOM     60  HA  GLU     5       14.263  -0.143  12.181  1.00
60.63           H
ATOM     61  HB1 GLU     5       13.196  -2.221  12.698  1.00
61.59           H
ATOM     62  HB2 GLU     5       12.196  -0.916  13.316  1.00
63.66           H
ATOM     63  HG1 GLU     5       13.044  -1.254  15.556  1.00
58.03           H
ATOM     64  HG2 GLU     5       14.219  -2.424  14.965  1.00
57.77           H
ATOM     65  N   ASN     6       15.179   1.538  14.180  1.00
50.73           N
ATOM     66  CA  ASN     6       15.197   2.747  15.007  1.00
71.04           C
ATOM     67  C   ASN     6       14.967   2.422  16.482  1.00
65.17           C
ATOM     68  O   ASN     6       15.056   3.296  17.343  1.00
94.86           O
ATOM     69  CB  ASN     6       14.128   3.732  14.529  1.00
94.83           C
ATOM     70  CG  ASN     6       14.551   4.369  13.210
1.00160.94           C
ATOM     71  ND2 ASN     6       13.650   4.914  12.442
1.00244.88           N
ATOM     72  OD1 ASN     6       15.735   4.366  12.870
1.00219.73           O
ATOM     73  HN  ASN     6       15.976   1.300  13.663  1.00
72.81           H
ATOM     74  HA  ASN     6       16.164   3.217  14.907  1.00
96.02           H
ATOM     75  HB1 ASN     6       14.001   4.497  15.273
1.00111.09           H
ATOM     76  HB2 ASN     6       13.193   3.213  14.391  1.00
98.13           H
ATOM     77 HD21 ASN     6       12.707   4.914  12.714
```

```
1.00272.43          H
ATOM     78 HD22 ASN     6      13.913   5.326  11.592
1.00336.78          H
ATOM     79  N   GLY     7      14.672   1.163  16.759  1.00
51.80          N
ATOM     80  CA  GLY     7      14.430   0.717  18.128  1.00
71.47          C
ATOM     81  C   GLY     7      14.700  -0.774  18.257  1.00
41.04          C
ATOM     82  O   GLY     7      13.808  -1.553  18.593  1.00
44.54          O
ATOM     83  HN  GLY     7      14.617   0.522  16.032  1.00
45.49          H
ATOM     84  HA1 GLY     7      13.405   0.911  18.386
1.00103.52          H
ATOM     85  HA2 GLY     7      15.079   1.257  18.802
1.00108.67          H
ATOM     86  N   ASP     8      15.936  -1.161  17.981  1.00
27.00          N
ATOM     87  CA  ASP     8      16.327  -2.557  18.059  1.00
14.15          C
ATOM     88  C   ASP     8      16.150  -3.090  19.477  1.00
9.37           C
ATOM     89  O   ASP     8      15.893  -4.277  19.675  1.00
14.61          O
ATOM     90  CB  ASP     8      17.785  -2.703  17.622  1.00
15.06          C
ATOM     91  CG  ASP     8      18.692  -1.859  18.510  1.00
21.22          C
ATOM     92  OD1 ASP     8      19.894  -1.906  18.307
1.00119.56          O
ATOM     93  OD2 ASP     8      18.173  -1.179  19.378
1.00133.27          O1-
ATOM     94  HN  ASP     8      16.597  -0.495  17.715  1.00
35.63          H
ATOM     95  HA  ASP     8      15.706  -3.132  17.391  1.00
20.92          H
ATOM     96  HB1 ASP     8      17.884  -2.376  16.598  1.00
29.39          H
ATOM     97  HB2 ASP     8      18.073  -3.734  17.694  1.00
44.14          H
ATOM     98  N   CYS     9      16.281  -2.206  20.461  1.00
5.93           N
ATOM     99  CA  CYS     9      16.121  -2.607  21.853  1.00
7.54           C
ATOM    100  C   CYS     9      14.707  -3.120  22.086  1.00
14.60          C
ATOM    101  O   CYS     9      14.496  -4.140  22.744  1.00
25.78          O
ATOM    102  CB  CYS     9      16.390  -1.416  22.764  1.00
6.63           C
ATOM    103  SG  CYS     9      18.145  -1.032  22.693  1.00
```

```
10.91           S
ATOM    104  HN  CYS     9      16.480  -1.271  20.248  1.00
6.51           H
ATOM    105  HA  CYS     9      16.834  -3.379  22.082  1.00
11.12           H
ATOM    106  HB1 CYS     9      16.117  -1.661  23.778  1.00
11.28           H
ATOM    107  HB2 CYS     9      15.816  -0.564  22.429  1.00
5.04           H
ATOM    108  N   ALA    10      13.746  -2.399  21.522  1.00
16.26           N
ATOM    109  CA  ALA    10      12.338  -2.756  21.637  1.00
32.04           C
ATOM    110  C   ALA    10      11.503  -1.811  20.778  1.00
45.47           C
ATOM    111  O   ALA    10      11.973  -0.739  20.392
1.00119.04           O
ATOM    112  CB  ALA    10      11.883  -2.677  23.098  1.00
30.10           C
ATOM    113  HN  ALA    10      13.994  -1.604  21.007  1.00
12.81           H
ATOM    114  HA  ALA    10      12.202  -3.766  21.280  1.00
47.14           H
ATOM    115  HB1 ALA    10      10.994  -3.276  23.231  1.00
84.37           H
ATOM    116  HB2 ALA    10      11.667  -1.652  23.356
1.00118.20           H
ATOM    117  HB3 ALA    10      12.667  -3.052  23.740
1.00117.32           H
ATOM    118  N   ALA    11      10.270  -2.201  20.478  1.00
30.62           N
ATOM    119  CA  ALA    11       9.405  -1.358  19.661  1.00
35.24           C
ATOM    120  C   ALA    11       9.173  -0.011  20.340  1.00
22.78           C
ATOM    121  O   ALA    11       9.260   1.040  19.704  1.00
53.33           O
ATOM    122  CB  ALA    11       8.061  -2.053  19.431  1.00
60.49           C
ATOM    123  HN  ALA    11       9.938  -3.063  20.808  1.00
50.37           H
ATOM    124  HA  ALA    11       9.879  -1.191  18.705  1.00
41.70           H
ATOM    125  HB1 ALA    11       8.232  -3.090  19.178
1.00157.06           H
ATOM    126  HB2 ALA    11       7.537  -1.566  18.624
1.00148.83           H
ATOM    127  HB3 ALA    11       7.468  -1.996  20.332
1.00137.06           H
ATOM    128  N   ASP    12       8.883  -0.050  21.637  1.00
18.98           N
ATOM    129  CA  ASP    12       8.645   1.173  22.397  1.00
```

```
31.05           C
ATOM    130  C   ASP    12         9.948   1.940  22.621  1.00
22.98           C
ATOM    131  O   ASP    12         9.970   3.170  22.580  1.00
37.14           O
ATOM    132  CB  ASP    12         8.015   0.832  23.748  1.00
48.35           C
ATOM    133  CG  ASP    12         6.581   0.355  23.548
1.00104.83           C
ATOM    134  OD1 ASP    12         6.059   0.546  22.461
1.00258.73           O
ATOM    135  OD2 ASP    12         6.023  -0.194  24.484
1.00213.46           O1-
ATOM    136  HN  ASP    12         8.831  -0.917  22.091  1.00
36.19           H
ATOM    137  HA  ASP    12         7.962   1.800  21.845  1.00
49.76           H
ATOM    138  HB1 ASP    12         8.014   1.712  24.375  1.00
95.71           H
ATOM    139  HB2 ASP    12         8.589   0.051  24.226  1.00
54.37           H
ATOM    140  N   GLU    13        11.026   1.203  22.869  1.00
17.60           N
ATOM    141  CA  GLU    13        12.329   1.816  23.114  1.00
12.01           C
ATOM    142  C   GLU    13        12.924   2.393  21.832  1.00
10.07           C
ATOM    143  O   GLU    13        12.561   1.988  20.727  1.00
12.81           O
ATOM    144  CB  GLU    13        13.287   0.780  23.700  1.00
11.74           C
ATOM    145  CG  GLU    13        12.762   0.311  25.059  1.00
13.49           C
ATOM    146  CD  GLU    13        12.891   1.433  26.082
1.00142.36           C
ATOM    147  OE1 GLU    13        12.267   1.331  27.125
1.00339.06           O
ATOM    148  OE2 GLU    13        13.611   2.378  25.808
1.00335.28           O1-
ATOM    149  HN  GLU    13        10.943   0.226  22.898  1.00
28.36           H
ATOM    150  HA  GLU    13        12.206   2.614  23.831  1.00
13.06           H
ATOM    151  HB1 GLU    13        14.261   1.223  23.825  1.00
13.76           H
ATOM    152  HB2 GLU    13        13.358  -0.064  23.029  1.00
9.94           H
ATOM    153  HG1 GLU    13        13.334  -0.542  25.390  1.00
66.02           H
ATOM    154  HG2 GLU    13        11.722   0.032  24.966  1.00
56.23           H
ATOM    155  N   CYS    14        13.847   3.345  21.992  1.00
```

9.12 N
ATOM 156 CA CYS 14 14.507 3.988 20.852 1.00
10.69 C
ATOM 157 C CYS 14 15.927 3.452 20.690 1.00
9.56 C
ATOM 158 O CYS 14 16.525 2.958 21.644 1.00
13.62 O
ATOM 159 CB CYS 14 14.560 5.501 21.065 1.00
13.96 C
ATOM 160 SG CYS 14 15.483 6.267 19.707 1.00
42.96 S
ATOM 161 HN CYS 14 14.094 3.620 22.902 1.00
9.76 H
ATOM 162 HA CYS 14 13.949 3.783 19.949 1.00
15.54 H
ATOM 163 HB1 CYS 14 15.051 5.716 22.002 1.00
50.72 H
ATOM 164 HB2 CYS 14 13.555 5.897 21.086 1.00
46.13 H
ATOM 165 N CYS 15 16.460 3.556 19.474 1.00
9.60 N
ATOM 166 CA CYS 15 17.813 3.081 19.189 1.00
9.28 C
ATOM 167 C CYS 15 18.455 3.951 18.118 1.00
9.92 C
ATOM 168 O CYS 15 17.870 4.176 17.059 1.00
13.31 O
ATOM 169 CB CYS 15 17.765 1.631 18.706 1.00
12.90 C
ATOM 170 SG CYS 15 19.446 1.048 18.370 1.00
39.11 S
ATOM 171 HN CYS 15 15.936 3.963 18.752 1.00
13.12 H
ATOM 172 HA CYS 15 18.410 3.133 20.088 1.00
8.26 H
ATOM 173 HB1 CYS 15 17.176 1.571 17.802 1.00
24.82 H
ATOM 174 HB2 CYS 15 17.317 1.012 19.469 1.00
27.31 H
ATOM 175 N VAL 16 19.663 4.440 18.391 1.00
9.56 N
ATOM 176 CA VAL 16 20.364 5.283 17.432 1.00
11.99 C
ATOM 177 C VAL 16 21.868 5.171 17.633 1.00
7.73 C
ATOM 178 O VAL 16 22.358 5.214 18.761 1.00
8.83 O
ATOM 179 CB VAL 16 19.913 6.736 17.597 1.00
18.37 C
ATOM 180 CG1 VAL 16 20.302 7.244 18.986 1.00
39.61 C
ATOM 181 CG2 VAL 16 20.580 7.599 16.528

```
1.00115.21           C
ATOM    182  HN  VAL    16       20.092   4.230  19.253  1.00
9.76           H
ATOM    183  HA  VAL    16       20.123   4.957  16.430  1.00
17.27           H
ATOM    184  HB  VAL    16       18.840   6.791  17.486  1.00
52.63           H
ATOM    185 HG11 VAL    16       19.949   6.551  19.734
1.00128.07           H
ATOM    186 HG12 VAL    16       19.854   8.212  19.153
1.00154.13           H
ATOM    187 HG13 VAL    16       21.377   7.330  19.051
1.00135.74           H
ATOM    188 HG21 VAL    16       20.350   7.201  15.552
1.00229.05           H
ATOM    189 HG22 VAL    16       21.650   7.594  16.678
1.00261.82           H
ATOM    190 HG23 VAL    16       20.210   8.610  16.602
1.00210.07           H
ATOM    191  N   ASP    17       22.598   5.012  16.531  1.00
14.34           N
ATOM    192  CA  ASP    17       24.053   4.879  16.593  1.00
11.90           C
ATOM    193  C   ASP    17       24.737   6.192  16.233  1.00
13.15           C
ATOM    194  O   ASP    17       24.582   6.703  15.123  1.00
25.96           O
ATOM    195  CB  ASP    17       24.513   3.786  15.625  1.00
20.80           C
ATOM    196  CG  ASP    17       24.098   2.416  16.150  1.00
28.72           C
ATOM    197  OD1 ASP    17       23.725   2.334  17.309
1.00135.42           O
ATOM    198  OD2 ASP    17       24.159   1.468  15.385
1.00114.17           O1-
ATOM    199  HN  ASP    17       22.151   4.974  15.661  1.00
28.29           H
ATOM    200  HA  ASP    17       24.347   4.596  17.594  1.00
10.49           H
ATOM    201  HB1 ASP    17       25.588   3.821  15.531  1.00
22.42           H
ATOM    202  HB2 ASP    17       24.063   3.951  14.659  1.00
29.52           H
ATOM    203  N   THR    18       25.508   6.725  17.179  1.00
11.55           N
ATOM    204  CA  THR    18       26.238   7.974  16.965  1.00
17.10           C
ATOM    205  C   THR    18       27.694   7.677  16.640  1.00
10.63           C
ATOM    206  O   THR    18       28.175   6.569  16.872  1.00
6.49           O
ATOM    207  CB  THR    18       26.165   8.848  18.220  1.00
```

28.52 C
ATOM 208 CG2 THR 18 24.777 9.475 18.339 1.00
45.38 C
ATOM 209 OG1 THR 18 26.426 8.050 19.362 1.00
26.49 O
ATOM 210 HN THR 18 25.598 6.262 18.038 1.00
13.96 H
ATOM 211 HA THR 18 25.796 8.515 16.138 1.00
26.43 H
ATOM 212 HB THR 18 26.902 9.633 18.156 1.00
37.43 H
ATOM 213 HG1 THR 18 25.614 7.599 19.602 1.00
71.62 H
ATOM 214 HG21 THR 18 24.692 10.287 17.632
1.00100.44 H
ATOM 215 HG22 THR 18 24.640 9.854 19.341
1.00129.42 H
ATOM 216 HG23 THR 18 24.024 8.730 18.128
1.00156.21 H
ATOM 217 N VAL 19 28.388 8.681 16.116 1.00
16.32 N
ATOM 218 CA VAL 19 29.783 8.555 15.766 1.00
13.34 C
ATOM 219 C VAL 19 30.248 9.848 15.132 1.00
25.88 C
ATOM 220 O VAL 19 29.731 10.299 14.111 1.00
42.11 O
ATOM 221 CB VAL 19 30.028 7.380 14.820 1.00
15.48 C
ATOM 222 CG1 VAL 19 29.029 7.421 13.660 1.00
29.06 C
ATOM 223 CG2 VAL 19 31.459 7.469 14.270 1.00
20.67 C
ATOM 224 HN VAL 19 27.955 9.542 15.974 1.00
26.89 H
ATOM 225 HA VAL 19 30.352 8.393 16.671 1.00
8.08 H
ATOM 226 HB VAL 19 29.911 6.458 15.367 1.00
11.49 H
ATOM 227 HG11 VAL 19 29.113 6.514 13.082
1.00137.43 H
ATOM 228 HG12 VAL 19 29.242 8.271 13.029 1.00
93.29 H
ATOM 229 HG13 VAL 19 28.026 7.506 14.051
1.00108.69 H
ATOM 230 HG21 VAL 19 31.743 6.518 13.842 1.00
99.42 H
ATOM 231 HG22 VAL 19 32.139 7.723 15.072
1.00111.36 H
ATOM 232 HG23 VAL 19 31.503 8.236 13.510 1.00
77.11 H
ATOM 233 N PHE 20 31.211 10.438 15.787 1.00

```
24.58           N
ATOM    234  CA  PHE    20      31.786  11.705  15.376  1.00
40.34           C
ATOM    235  C   PHE    20      33.030  11.501  14.516  1.00
40.37           C
ATOM    236  O   PHE    20      32.946  11.338  13.299  1.00
66.02           O
ATOM    237  CB  PHE    20      32.142  12.523  16.639  1.00
48.79           C
ATOM    238  CG  PHE    20      32.447  11.602  17.813  1.00
35.75           C
ATOM    239  CD1 PHE    20      31.429  10.824  18.398  1.00
30.20           C
ATOM    240  CD2 PHE    20      33.747  11.542  18.333  1.00
40.95           C
ATOM    241  CE1 PHE    20      31.719   9.994  19.485  1.00
30.41           C
ATOM    242  CE2 PHE    20      34.034  10.710  19.420  1.00
45.84           C
ATOM    243  CZ  PHE    20      33.020   9.934  19.996  1.00
40.81           C
ATOM    244  HN  PHE    20      31.530  10.013  16.598  1.00
16.73           H
ATOM    245  HA  PHE    20      31.057  12.259  14.799  1.00
59.91           H
ATOM    246  HB1 PHE    20      31.313  13.138  16.895  1.00
67.15           H
ATOM    247  HB2 PHE    20      32.995  13.158  16.446  1.00
58.17           H
ATOM    248  HD1 PHE    20      30.420  10.855  18.006  1.00
33.34           H
ATOM    249  HD2 PHE    20      34.528  12.139  17.895  1.00
49.35           H
ATOM    250  HE1 PHE    20      30.937   9.400  19.931  1.00
30.61           H
ATOM    251  HE2 PHE    20      35.037  10.662  19.815  1.00
61.27           H
ATOM    252  HZ  PHE    20      33.242   9.293  20.834  1.00
52.66           H
ATOM    253  N   GLU    21      34.174  11.541  15.170  1.00
41.31           N
ATOM    254  CA  GLU    21      35.455  11.396  14.499  1.00
56.16           C
ATOM    255  C   GLU    21      35.773   9.919  14.251  1.00
58.61           C
ATOM    256  O   GLU    21      36.571   9.588  13.374
1.00201.19           O
ATOM    257  CB  GLU    21      36.520  12.075  15.374  1.00
57.37           C
ATOM    258  CG  GLU    21      37.927  11.537  15.074
1.00193.56           C
ATOM    259  CD  GLU    21      38.208  11.614  13.577
```

```
1.00304.62           C
ATOM    260  OE1 GLU    21      38.703  10.639  13.035
1.00451.81           O
ATOM    261  OE2 GLU    21      37.922  12.647  12.993
1.00442.88           O1-
ATOM    262  HN  GLU    21      34.157  11.693  16.139  1.00
50.82          H
ATOM    263  HA  GLU    21      35.410  11.905  13.547  1.00
85.72          H
ATOM    264  HB1 GLU    21      36.276  11.903  16.412  1.00
46.37          H
ATOM    265  HB2 GLU    21      36.506  13.141  15.184
1.00131.81           H
ATOM    266  HG1 GLU    21      38.000  10.511  15.401
1.00314.91           H
ATOM    267  HG2 GLU    21      38.657  12.132  15.604
1.00266.92           H
ATOM    268  N   GLY    22      35.143   9.036  15.019  1.00
86.70          N
ATOM    269  CA  GLY    22      35.365   7.597  14.864
1.00104.66           C
ATOM    270  C   GLY    22      36.418   7.089  15.846  1.00
71.50          C
ATOM    271  O   GLY    22      36.686   5.889  15.907  1.00
89.87          O
ATOM    272  HN  GLY    22      34.512   9.353  15.700
1.00214.64           H
ATOM    273  HA1 GLY    22      35.695   7.389  13.856
1.00152.47           H
ATOM    274  HA2 GLY    22      34.436   7.078  15.045
1.00118.15           H
ATOM    275  N   ASP    23      37.002   7.997  16.621  1.00
44.68          N
ATOM    276  CA  ASP    23      38.010   7.599  17.599  1.00
43.33          C
ATOM    277  C   ASP    23      37.393   6.613  18.581  1.00
37.55          C
ATOM    278  O   ASP    23      38.016   5.626  18.972  1.00
61.37          O
ATOM    279  CB  ASP    23      38.529   8.826  18.353  1.00
40.74          C
ATOM    280  CG  ASP    23      39.402   9.672  17.434
1.00141.86           C
ATOM    281  OD1 ASP    23      39.763   9.184  16.377
1.00328.18           O
ATOM    282  OD2 ASP    23      39.697  10.799  17.802
1.00304.58           O1-
ATOM    283  HN  ASP    23      36.750   8.940  16.541  1.00
40.86          H
ATOM    284  HA  ASP    23      38.832   7.124  17.087  1.00
72.12          H
ATOM    285  HB1 ASP    23      39.111   8.504  19.204  1.00
```

```
94.15           H
ATOM    286  HB2 ASP    23       37.690   9.415  18.695  1.00
93.26           H
ATOM    287  N   MET    24       36.148   6.885  18.951  1.00
23.24           N
ATOM    288  CA  MET    24       35.404   6.026  19.864  1.00
32.50           C
ATOM    289  C   MET    24       33.924   6.100  19.514  1.00
25.13           C
ATOM    290  O   MET    24       33.263   7.102  19.784  1.00
54.40           O
ATOM    291  CB  MET    24       35.620   6.475  21.313  1.00
47.36           C
ATOM    292  CG  MET    24       34.875   5.534  22.268
1.00151.73           C
ATOM    293  SD  MET    24       33.133   6.024  22.370
1.00209.23           S
ATOM    294  CE  MET    24       33.328   7.489  23.425
1.00243.29           C
ATOM    295  HN  MET    24       35.708   7.680  18.582  1.00
17.32           H
ATOM    296  HA  MET    24       35.743   5.005  19.754  1.00
48.94           H
ATOM    297  HB1 MET    24       35.245   7.481  21.434
1.00124.40           H
ATOM    298  HB2 MET    24       36.677   6.457  21.540
1.00166.96           H
ATOM    299  HG1 MET    24       35.322   5.593  23.250
1.00331.71           H
ATOM    300  HG2 MET    24       34.941   4.519  21.906
1.00302.30           H
ATOM    301  HE1 MET    24       32.872   8.340  22.941
1.00340.44           H
ATOM    302  HE2 MET    24       34.374   7.693  23.589
1.00373.88           H
ATOM    303  HE3 MET    24       32.846   7.311  24.376
1.00386.81           H
ATOM    304  N   VAL    25       33.411   5.044  18.889  1.00
22.44           N
ATOM    305  CA  VAL    25       32.011   5.027  18.487  1.00
15.37           C
ATOM    306  C   VAL    25       31.088   5.081  19.694  1.00
17.47           C
ATOM    307  O   VAL    25       31.395   4.542  20.759  1.00
29.52           O
ATOM    308  CB  VAL    25       31.696   3.782  17.660  1.00
23.36           C
ATOM    309  CG1 VAL    25       30.210   3.800  17.279  1.00
58.39           C
ATOM    310  CG2 VAL    25       32.553   3.784  16.392  1.00
55.25           C
ATOM    311  HN  VAL    25       33.985   4.278  18.680  1.00
```

```
48.11            H
ATOM    312  HA  VAL    25       31.823   5.896  17.875  1.00
9.25           H
ATOM    313  HB  VAL    25       31.908   2.896  18.244  1.00
52.45            H
ATOM    314 HG11 VAL    25       29.921   4.805  16.995
1.00171.07            H
ATOM    315 HG12 VAL    25       29.620   3.485  18.125
1.00166.22            H
ATOM    316 HG13 VAL    25       30.040   3.130  16.451
1.00134.76            H
ATOM    317 HG21 VAL    25       32.372   2.878  15.832
1.00141.88            H
ATOM    318 HG22 VAL    25       33.598   3.836  16.663
1.00184.50            H
ATOM    319 HG23 VAL    25       32.295   4.639  15.784
1.00133.90            H
ATOM    320  N   THR    26       29.957   5.755  19.514  1.00
13.60            N
ATOM    321  CA  THR    26       28.966   5.920  20.575  1.00
21.19            C
ATOM    322  C   THR    26       27.592   5.434  20.119  1.00
16.55            C
ATOM    323  O   THR    26       27.150   5.738  19.010  1.00
11.43            O
ATOM    324  CB  THR    26       28.893   7.398  20.947  1.00
27.07            C
ATOM    325  CG2 THR    26       27.815   7.619  22.012  1.00
44.39            C
ATOM    326  OG1 THR    26       30.154   7.811  21.454  1.00
34.75            O
ATOM    327  HN  THR    26       29.787   6.167  18.640  1.00
10.81            H
ATOM    328  HA  THR    26       29.266   5.356  21.448  1.00
33.16            H
ATOM    329  HB  THR    26       28.655   7.973  20.061  1.00
20.60            H
ATOM    330  HG1 THR    26       30.171   8.771  21.460  1.00
77.72            H
ATOM    331 HG21 THR    26       26.839   7.449  21.583
1.00126.99            H
ATOM    332 HG22 THR    26       27.874   8.633  22.377
1.00108.32            H
ATOM    333 HG23 THR    26       27.972   6.933  22.831
1.00151.99            H
ATOM    334  N   ARG    27       26.918   4.679  20.988  1.00
22.38            N
ATOM    335  CA  ARG    27       25.584   4.146  20.684  1.00
20.21            C
ATOM    336  C   ARG    27       24.618   4.485  21.817  1.00
16.96            C
ATOM    337  O   ARG    27       25.024   4.576  22.976  1.00
```

20.06 O
ATOM 338 CB ARG 27 25.661 2.627 20.519 1.00
22.14 C
ATOM 339 CG ARG 27 26.606 2.282 19.366
1.00124.30 C
ATOM 340 CD ARG 27 26.867 0.776 19.353
1.00109.38 C
ATOM 341 NE ARG 27 27.583 0.377 20.559
1.00227.73 N
ATOM 342 CZ ARG 27 27.843 -0.901 20.812
1.00426.12 C
ATOM 343 NH1 ARG 27 28.490 -1.233 21.898
1.00767.09 N1+
ATOM 344 NH2 ARG 27 27.453 -1.825 19.978
1.00581.78 N
ATOM 345 HN ARG 27 27.324 4.475 21.856 1.00
30.75 H
ATOM 346 HA ARG 27 25.216 4.583 19.766 1.00
23.13 H
ATOM 347 HB1 ARG 27 24.677 2.238 20.304 1.00
87.46 H
ATOM 348 HB2 ARG 27 26.031 2.185 21.433
1.00103.36 H
ATOM 349 HG1 ARG 27 27.541 2.806 19.497
1.00281.97 H
ATOM 350 HG2 ARG 27 26.154 2.578 18.430
1.00276.19 H
ATOM 351 HD1 ARG 27 27.457 0.523 18.484
1.00183.60 H
ATOM 352 HD2 ARG 27 25.925 0.250 19.310
1.00142.93 H
ATOM 353 HE ARG 27 27.879 1.064 21.192
1.00372.53 H
ATOM 354 HH11 ARG 27 28.790 -0.526 22.537
1.00910.59 H
ATOM 355 HH12 ARG 27 28.686 -2.194 22.087
1.00999.99 H
ATOM 356 HH21 ARG 27 26.956 -1.570 19.148
1.00532.54 H
ATOM 357 HH22 ARG 27 27.649 -2.787 20.170
1.00948.84 H
ATOM 358 N SER 28 23.337 4.672 21.486 1.00
14.60 N
ATOM 359 CA SER 28 22.329 5.001 22.499 1.00
14.92 C
ATOM 360 C SER 28 21.089 4.132 22.327 1.00
12.12 C
ATOM 361 O SER 28 20.700 3.801 21.206 1.00
13.41 O
ATOM 362 CB SER 28 21.941 6.476 22.385 1.00
23.26 C
ATOM 363 OG SER 28 23.079 7.283 22.654

1.00146.32 O
ATOM 364 HN SER 28 23.061 4.587 20.546 1.00
15.23 H
ATOM 365 HA SER 28 22.737 4.828 23.486 1.00
15.44 H
ATOM 366 HB1 SER 28 21.153 6.695 23.095 1.00
88.73 H
ATOM 367 HB2 SER 28 21.589 6.682 21.388
1.00124.68 H
ATOM 368 HG SER 28 23.693 7.186 21.923
1.00242.47 H
ATOM 369 N CYS 29 20.472 3.769 23.445 1.00
10.48 N
ATOM 370 CA CYS 29 19.273 2.941 23.412 1.00
9.97 C
ATOM 371 C CYS 29 18.674 2.832 24.814 1.00
11.60 C
ATOM 372 O CYS 29 19.292 2.269 25.718 1.00
15.64 O
ATOM 373 CB CYS 29 19.615 1.543 22.869 1.00
9.52 C
ATOM 374 SG CYS 29 18.190 0.860 22.010 1.00
10.40 S
ATOM 375 HN CYS 29 20.826 4.067 24.308 1.00
11.10 H
ATOM 376 HA CYS 29 18.549 3.404 22.758 1.00
11.08 H
ATOM 377 HB1 CYS 29 19.877 0.876 23.684 1.00
9.33 H
ATOM 378 HB2 CYS 29 20.441 1.609 22.183 1.00
10.78 H
ATOM 379 N GLU 30 17.473 3.371 24.990 1.00
13.52 N
ATOM 380 CA GLU 30 16.814 3.323 26.290 1.00
17.41 C
ATOM 381 C GLU 30 16.184 1.956 26.522 1.00
10.81 C
ATOM 382 O GLU 30 15.815 1.263 25.575 1.00
23.22 O
ATOM 383 CB GLU 30 15.740 4.409 26.376 1.00
37.83 C
ATOM 384 CG GLU 30 16.387 5.784 26.205
1.00108.13 C
ATOM 385 CD GLU 30 17.311 6.077 27.381
1.00240.52 C
ATOM 386 OE1 GLU 30 17.143 5.446 28.413
1.00422.56 O
ATOM 387 OE2 GLU 30 18.173 6.927 27.235
1.00410.81 O1-
ATOM 388 HN GLU 30 17.021 3.808 24.235 1.00
15.61 H
ATOM 389 HA GLU 30 17.548 3.502 27.061 1.00

```
24.60           H
ATOM    390  HB1 GLU    30       15.252   4.360  27.337  1.00
72.67           H
ATOM    391  HB2 GLU    30       15.011   4.255  25.592  1.00
56.47           H
ATOM    392  HG1 GLU    30       15.615   6.538  26.163
1.00196.66           H
ATOM    393  HG2 GLU    30       16.955   5.803  25.287
1.00140.67           H
ATOM    394  N   LYS    31       16.064   1.568  27.792  1.00
11.96           N
ATOM    395  CA  LYS    31       15.476   0.276  28.153  1.00
11.40           C
ATOM    396  C   LYS    31       14.181   0.484  28.928  1.00
10.99           C
ATOM    397  O   LYS    31       14.073   1.404  29.740  1.00
12.86           O
ATOM    398  CB  LYS    31       16.459  -0.520  29.012  1.00
21.40           C
ATOM    399  CG  LYS    31       17.738  -0.784  28.209  1.00
57.81           C
ATOM    400  CD  LYS    31       18.635  -1.781  28.955
1.00115.52           C
ATOM    401  CE  LYS    31       19.266  -1.110  30.180
1.00250.23           C
ATOM    402  NZ  LYS    31       20.316  -2.005  30.746
1.00462.10           N1+
ATOM    403  HN  LYS    31       16.378   2.165  28.503  1.00
26.13           H
ATOM    404  HA  LYS    31       15.259  -0.291  27.256  1.00
13.87           H
ATOM    405  HB1 LYS    31       16.012  -1.461  29.295  1.00
37.63           H
ATOM    406  HB2 LYS    31       16.695   0.048  29.897  1.00
47.19           H
ATOM    407  HG1 LYS    31       18.271   0.144  28.072
1.00126.32           H
ATOM    408  HG2 LYS    31       17.475  -1.192  27.244
1.00114.19           H
ATOM    409  HD1 LYS    31       19.418  -2.121  28.293
1.00198.45           H
ATOM    410  HD2 LYS    31       18.044  -2.626  29.274
1.00200.14           H
ATOM    411  HE1 LYS    31       18.508  -0.934  30.928
1.00374.64           H
ATOM    412  HE2 LYS    31       19.712  -0.170  29.890
1.00403.27           H
ATOM    413  HZ1 LYS    31       19.875  -2.875  31.104
1.00627.38           H
ATOM    414  HZ2 LYS    31       20.805  -1.517  31.524
1.00622.26           H
ATOM    415  HZ3 LYS    31       21.002  -2.249  30.003
```

```
1.00619.20           H
ATOM    416  N   THR    32       13.201  -0.374  28.674  1.00
15.51          N
ATOM    417  CA  THR    32       11.917  -0.272  29.354  1.00
22.44          C
ATOM    418  C   THR    32       12.054  -0.676  30.817  1.00
26.60          C
ATOM    419  O   THR    32       12.537  -1.764  31.132  1.00
57.43          O
ATOM    420  CB  THR    32       10.887  -1.171  28.662  1.00
56.28          C
ATOM    421  CG2 THR    32        9.536  -1.041  29.367
1.00102.70           C
ATOM    422  OG1 THR    32       10.755  -0.775  27.305
1.00111.17           O
ATOM    423  HN  THR    32       13.343  -1.085  28.017  1.00
18.79          H
ATOM    424  HA  THR    32       11.575   0.752  29.303  1.00
19.67          H
ATOM    425  HB  THR    32       11.216  -2.197  28.710  1.00
84.25          H
ATOM    426  HG1 THR    32       11.000   0.152  27.239
1.00205.03           H
ATOM    427 HG21 THR    32        9.263   0.002  29.430
1.00220.71           H
ATOM    428 HG22 THR    32        9.607  -1.456  30.361
1.00174.67           H
ATOM    429 HG23 THR    32        8.786  -1.576  28.806
1.00217.44           H
ATOM    430  N   THR    33       11.620   0.211  31.706  1.00
22.47          N
ATOM    431  CA  THR    33       11.689  -0.050  33.141  1.00
41.60          C
ATOM    432  C   THR    33       10.612   0.709  33.867  1.00
38.03          C
ATOM    433  O   THR    33       10.814   1.836  34.316  1.00
52.73          O
ATOM    434  CB  THR    33       13.061   0.340  33.693  1.00
63.25          C
ATOM    435  CG2 THR    33       14.127  -0.618  33.159
1.00121.65           C
ATOM    436  OG1 THR    33       13.370   1.668  33.290  1.00
96.20          O
ATOM    437  HN  THR    33       11.244   1.058  31.390  1.00
23.82          H
ATOM    438  HA  THR    33       11.518  -1.093  33.328  1.00
67.12          H
ATOM    439  HB  THR    33       13.044   0.287  34.770
1.00107.59           H
ATOM    440  HG1 THR    33       13.999   1.619  32.567
1.00176.27           H
ATOM    441 HG21 THR    33       15.045  -0.478  33.710
```

```
1.00255.23           H
ATOM    442 HG22 THR    33      14.303  -0.413  32.112
1.00245.37           H
ATOM    443 HG23 THR    33      13.788  -1.637  33.274
1.00185.78           H
ATOM    444  N   GLY    34       9.465   0.065  33.993  1.00
40.93           N
ATOM    445  CA  GLY    34       8.357   0.671  34.685  1.00
45.59           C
ATOM    446  C   GLY    34       7.744   1.792  33.853  1.00
40.97           C
ATOM    447  O   GLY    34       6.522   1.899  33.740  1.00
82.17           O
ATOM    448  HN  GLY    34       9.372  -0.838  33.625  1.00
54.00           H
ATOM    449  HA1 GLY    34       8.728   1.071  35.607  1.00
54.41           H
ATOM    450  HA2 GLY    34       7.608  -0.079  34.892  1.00
62.09           H
ATOM    451  N   ASN    35       8.604   2.623  33.265  1.00
32.51           N
ATOM    452  CA  ASN    35       8.152   3.738  32.431  1.00
33.20           C
ATOM    453  C   ASN    35       8.998   3.824  31.165  1.00
23.17           C
ATOM    454  O   ASN    35      10.215   3.642  31.206  1.00
27.01           O
ATOM    455  CB  ASN    35       8.260   5.051  33.206  1.00
54.51           C
ATOM    456  CG  ASN    35       7.480   4.957  34.504  1.00
80.73           C
ATOM    457  ND2 ASN    35       6.207   4.711  34.468
1.00220.46           N
ATOM    458  OD1 ASN    35       8.050   5.113  35.585
1.00117.80           O
ATOM    459  HN  ASN    35       9.566   2.481  33.391  1.00
51.15           H
ATOM    460  HA  ASN    35       7.119   3.584  32.148  1.00
47.21           H
ATOM    461  HB1 ASN    35       7.861   5.854  32.607  1.00
68.31           H
ATOM    462  HB2 ASN    35       9.290   5.250  33.432  1.00
57.75           H
ATOM    463 HD21 ASN    35       5.754   4.587  33.608
1.00405.39           H
ATOM    464 HD22 ASN    35       5.705   4.650  35.298
1.00245.64           H
ATOM    465  N   PHE    36       8.345   4.098  30.041  1.00
35.72           N
ATOM    466  CA  PHE    36       9.036   4.202  28.765  1.00
33.41           C
ATOM    467  C   PHE    36       9.638   5.593  28.588  1.00
```

28.51 C
ATOM 468 O PHE 36 9.075 6.585 29.052 1.00
45.23 O
ATOM 469 CB PHE 36 8.045 3.926 27.640 1.00
67.53 C
ATOM 470 CG PHE 36 7.588 2.489 27.718 1.00
86.71 C
ATOM 471 CD1 PHE 36 6.468 2.155 28.489
1.00106.51 C
ATOM 472 CD2 PHE 36 8.281 1.492 27.020 1.00
99.48 C
ATOM 473 CE1 PHE 36 6.040 0.824 28.562
1.00134.48 C
ATOM 474 CE2 PHE 36 7.854 0.162 27.094
1.00134.27 C
ATOM 475 CZ PHE 36 6.733 -0.173 27.864
1.00149.48 C
ATOM 476 HN PHE 36 7.376 4.229 30.065 1.00
62.87 H
ATOM 477 HA PHE 36 9.825 3.466 28.725 1.00
28.53 H
ATOM 478 HB1 PHE 36 8.522 4.099 26.694 1.00
73.56 H
ATOM 479 HB2 PHE 36 7.193 4.582 27.741 1.00
88.42 H
ATOM 480 HD1 PHE 36 5.935 2.926 29.027
1.00110.20 H
ATOM 481 HD2 PHE 36 9.147 1.750 26.427 1.00
93.07 H
ATOM 482 HE1 PHE 36 5.176 0.567 29.156
1.00155.06 H
ATOM 483 HE2 PHE 36 8.388 -0.607 26.555
1.00159.46 H
ATOM 484 HZ PHE 36 6.403 -1.199 27.920
1.00183.25 H
ATOM 485 N THR 37 10.786 5.658 27.913 1.00
20.06 N
ATOM 486 CA THR 37 11.469 6.927 27.672 1.00
27.18 C
ATOM 487 C THR 37 11.769 7.081 26.186 1.00
41.87 C
ATOM 488 O THR 37 11.007 6.622 25.335
1.00169.86 O
ATOM 489 CB THR 37 12.774 6.974 28.467 1.00
26.52 C
ATOM 490 CG2 THR 37 12.492 6.653 29.935 1.00
75.69 C
ATOM 491 OG1 THR 37 13.685 6.021 27.937 1.00
81.95 O
ATOM 492 HN THR 37 11.187 4.836 27.567 1.00
18.47 H
ATOM 493 HA THR 37 10.837 7.747 27.986 1.00

38.80 H
ATOM 494 HB THR 37 13.205 7.962 28.395 1.00
81.60 H
ATOM 495 HG1 THR 37 13.326 5.146 28.099
1.00188.80 H
ATOM 496 HG21 THR 37 11.653 7.241 30.276
1.00196.98 H
ATOM 497 HG22 THR 37 13.363 6.889 30.528
1.00196.23 H
ATOM 498 HG23 THR 37 12.262 5.603 30.035
1.00168.92 H
ATOM 499 N GLU 38 12.884 7.730 25.884 1.00
26.53 N
ATOM 500 CA GLU 38 13.284 7.946 24.499 1.00
30.29 C
ATOM 501 C GLU 38 14.772 8.275 24.415 1.00
25.39 C
ATOM 502 O GLU 38 15.335 8.883 25.325 1.00
51.60 O
ATOM 503 CB GLU 38 12.471 9.093 23.894 1.00
54.82 C
ATOM 504 CG GLU 38 12.815 9.242 22.409
1.00165.84 C
ATOM 505 CD GLU 38 11.957 10.334 21.781
1.00292.92 C
ATOM 506 OE1 GLU 38 11.157 10.916 22.496
1.00414.19 O
ATOM 507 OE2 GLU 38 12.109 10.572 20.595
1.00537.65 O1-
ATOM 508 HN GLU 38 13.448 8.067 26.609 1.00
84.50 H
ATOM 509 HA GLU 38 13.092 7.046 23.934 1.00
32.15 H
ATOM 510 HB1 GLU 38 12.705 10.012 24.409
1.00144.82 H
ATOM 511 HB2 GLU 38 11.416 8.879 23.998
1.00106.49 H
ATOM 512 HG1 GLU 38 12.630 8.305 21.903
1.00303.31 H
ATOM 513 HG2 GLU 38 13.859 9.503 22.307
1.00323.99 H
ATOM 514 N CYS 39 15.402 7.872 23.315 1.00
15.84 N
ATOM 515 CA CYS 39 16.823 8.134 23.122 1.00
14.30 C
ATOM 516 C CYS 39 17.075 9.646 23.090 1.00
22.31 C
ATOM 517 O CYS 39 16.202 10.408 22.674 1.00
32.92 O
ATOM 518 CB CYS 39 17.283 7.503 21.805 1.00
18.08 C
ATOM 519 SG CYS 39 16.165 8.009 20.476 1.00

17.41 S
ATOM 520 HN CYS 39 14.901 7.396 22.622 1.00
24.87 H
ATOM 521 HA CYS 39 17.371 7.687 23.935 1.00
14.60 H
ATOM 522 HB1 CYS 39 17.273 6.427 21.897 1.00
27.71 H
ATOM 523 HB2 CYS 39 18.285 7.834 21.576 1.00
34.91 H
ATOM 524 N PRO 40 18.232 10.103 23.517 1.00
27.27 N
ATOM 525 CA PRO 40 18.554 11.558 23.524 1.00
47.66 C
ATOM 526 C PRO 40 18.850 12.085 22.122 1.00
71.54 C
ATOM 527 O PRO 40 19.590 11.469 21.355 1.00
87.70 O
ATOM 528 CB PRO 40 19.792 11.642 24.426 1.00
53.85 C
ATOM 529 CG PRO 40 20.475 10.326 24.244 1.00
46.04 C
ATOM 530 CD PRO 40 19.357 9.299 24.033 1.00
25.70 C
ATOM 531 HA PRO 40 17.746 12.117 23.968 1.00
58.04 H
ATOM 532 HB1 PRO 40 19.498 11.766 25.458 1.00
67.93 H
ATOM 533 HB2 PRO 40 20.437 12.455 24.116 1.00
70.99 H
ATOM 534 HG1 PRO 40 21.045 10.071 25.125 1.00
65.91 H
ATOM 535 HG2 PRO 40 21.123 10.362 23.376 1.00
54.99 H
ATOM 536 HD1 PRO 40 19.084 8.834 24.969 1.00
24.22 H
ATOM 537 HD2 PRO 40 19.657 8.557 23.309 1.00
26.94 H
ATOM 538 N GLY 41 18.268 13.233 21.803
1.00105.24 N
ATOM 539 CA GLY 41 18.473 13.850 20.497
1.00147.55 C
ATOM 540 C GLY 41 19.934 14.230 20.300
1.00161.74 C
ATOM 541 O GLY 41 20.488 14.070 19.212
1.00244.37 O
ATOM 542 HN GLY 41 17.694 13.674 22.460
1.00121.79 H
ATOM 543 HA1 GLY 41 17.865 14.738 20.425
1.00184.40 H
ATOM 544 HA2 GLY 41 18.180 13.154 19.727
1.00157.34 H
ATOM 545 N LEU 42 20.550 14.736 21.363

1.00162.48 N
ATOM 546 CA LEU 42 21.952 15.148 21.316
1.00191.48 C
ATOM 547 C LEU 42 22.853 14.022 21.815
1.00166.66 C
ATOM 548 O LEU 42 22.438 13.196 22.628
1.00251.38 O
ATOM 549 CB LEU 42 22.159 16.387 22.188
1.00307.99 C
ATOM 550 CG LEU 42 21.146 17.470 21.802
1.00452.28 C
ATOM 551 CD1 LEU 42 21.373 18.707 22.677
1.00681.24 C
ATOM 552 CD2 LEU 42 21.315 17.843 20.321
1.00527.46 C
ATOM 553 HN LEU 42 20.049 14.839 22.199
1.00195.47 H
ATOM 554 HA LEU 42 22.226 15.387 20.298
1.00229.77 H
ATOM 555 HB1 LEU 42 23.159 16.766 22.043
1.00342.49 H
ATOM 556 HB2 LEU 42 22.023 16.121 23.228
1.00335.33 H
ATOM 557 HG LEU 42 20.145 17.097 21.968
1.00427.79 H
ATOM 558 HD11 LEU 42 22.357 19.107 22.488
1.00909.71 H
ATOM 559 HD12 LEU 42 21.288 18.432 23.718
1.00706.80 H
ATOM 560 HD13 LEU 42 20.629 19.455 22.441
1.00858.82 H
ATOM 561 HD21 LEU 42 20.895 18.824 20.143
1.00631.69 H
ATOM 562 HD22 LEU 42 20.800 17.120 19.707
1.00628.06 H
ATOM 563 HD23 LEU 42 22.365 17.851 20.065
1.00656.75 H
ATOM 564 N THR 43 24.085 13.995 21.319
1.00178.52 N
ATOM 565 CA THR 43 25.036 12.965 21.722
1.00240.84 C
ATOM 566 C THR 43 25.387 13.123 23.206
1.00372.31 C
ATOM 567 O THR 43 25.379 14.237 23.726
1.00500.13 O
ATOM 568 CB THR 43 26.307 13.091 20.875
1.00354.13 C
ATOM 569 CG2 THR 43 25.937 13.066 19.392
1.00463.04 C
ATOM 570 OG1 THR 43 26.957 14.318 21.181
1.00508.50 O
ATOM 571 HN THR 43 24.361 14.677 20.673

```
1.00229.59           H
ATOM    572  HA  THR    43      24.593  11.998  21.550
1.00249.90           H
ATOM    573  HB  THR    43      26.972  12.270  21.089
1.00481.58           H
ATOM    574  HG1 THR    43      27.782  14.346  20.695
1.00624.31           H
ATOM    575 HG21 THR    43      25.322  13.922  19.160
1.00650.95           H
ATOM    576 HG22 THR    43      25.393  12.160  19.171
1.00580.73           H
ATOM    577 HG23 THR    43      26.838  13.098  18.796
1.00571.99           H
ATOM    578  N   PRO    44      25.697  12.047  23.900
1.00488.39           N
ATOM    579  CA  PRO    44      26.055  12.115  25.347
1.00760.13           C
ATOM    580  C   PRO    44      27.460  12.677  25.549
1.00735.36           C
ATOM    581  O   PRO    44      27.867  12.974  26.672
1.00999.99           O
ATOM    582  CB  PRO    44      25.963  10.654  25.806
1.00999.99           C
ATOM    583  CG  PRO    44      26.295   9.860  24.586
1.00856.94           C
ATOM    584  CD  PRO    44      25.745  10.657  23.400
1.00548.09           C
ATOM    585  HA  PRO    44      25.334  12.711  25.884
1.00939.00           H
ATOM    586  HB1 PRO    44      24.959  10.426  26.133
1.00999.99           H
ATOM    587  HB2 PRO    44      26.672  10.457  26.599
1.00999.99           H
ATOM    588  HG1 PRO    44      25.823   8.889  24.628
1.00999.99           H
ATOM    589  HG2 PRO    44      27.370   9.747  24.500
1.00853.53           H
ATOM    590  HD1 PRO    44      24.754  10.319  23.140
1.00602.45           H
ATOM    591  HD2 PRO    44      26.411  10.577  22.551
1.00463.32           H
ATOM    592  N   ILE    45      28.193  12.813  24.449
1.00518.33           N
ATOM    593  CA  ILE    45      29.553  13.333  24.508
1.00582.79           C
ATOM    594  C   ILE    45      29.553  14.753  25.059
1.00795.93           C
ATOM    595  O   ILE    45      30.368  15.100  25.915
1.00898.75           O
ATOM    596  CB  ILE    45      30.173  13.334  23.107
1.00474.41           C
ATOM    597  CG1 ILE    45      30.207  11.903  22.539
```

```
1.00516.90           C
ATOM    598  CG2 ILE    45       31.593  13.901  23.171
1.00760.75           C
ATOM    599  CD1 ILE    45       30.965  10.950  23.474
1.00566.81           C
ATOM    600  HN  ILE    45       27.815  12.555  23.583
1.00395.72           H
ATOM    601  HA  ILE    45       30.139  12.710  25.156
1.00711.06           H
ATOM    602  HB  ILE    45       29.574  13.959  22.460
1.00477.81           H
ATOM    603 HG11 ILE    45       30.693  11.916  21.575
1.00624.28           H
ATOM    604 HG12 ILE    45       29.194  11.547  22.418
1.00744.84           H
ATOM    605 HG21 ILE    45       32.098  13.713  22.236
1.00920.57           H
ATOM    606 HG22 ILE    45       32.134  13.427  23.975
1.00999.99           H
ATOM    607 HG23 ILE    45       31.548  14.966  23.346
1.00865.46           H
ATOM    608 HD11 ILE    45       30.288  10.568  24.225
1.00658.89           H
ATOM    609 HD12 ILE    45       31.779  11.471  23.956
1.00734.30           H
ATOM    610 HD13 ILE    45       31.360  10.126  22.899
1.00675.84           H
ATOM    611  N   ALA    46       28.634  15.568  24.563
1.00999.99           N
ATOM    612  CA  ALA    46       28.530  16.952  25.008
1.00999.99           C
ATOM    613  C   ALA    46       27.896  17.024  26.393
1.00999.99           C
ATOM    614  CB  ALA    46       27.686  17.758  24.017
1.00999.99           C
ATOM    615  OT1 ALA    46       28.622  17.270  27.343
1.00999.99           O
ATOM    616  OT2 ALA    46       26.695  16.830  26.485
1.00999.99           O
ATOM    617  HN  ALA    46       28.014  15.231  23.885
1.00999.99           H
ATOM    618  HA  ALA    46       29.519  17.382  25.052
1.00999.99           H
ATOM    619  HB1 ALA    46       28.245  17.903  23.105
1.00999.99           H
ATOM    620  HB2 ALA    46       27.443  18.717  24.448
1.00999.99           H
ATOM    621  HB3 ALA    46       26.775  17.219  23.800
1.00999.99           H
ENDMDL
TER
MODEL        19
```

```
ATOM      1  N   GLY     1       27.688   0.229  15.683
1.00999.99           N
ATOM      2  CA  GLY     1       26.988  -0.149  14.421
1.00999.99           C
ATOM      3  C   GLY     1       25.732   0.701  14.262
1.00999.99           C
ATOM      4  O   GLY     1       25.568   1.716  14.938
1.00999.99           O
ATOM      5  HA1 GLY     1       26.710  -1.191  14.461
1.00999.99           H
ATOM      6  HA2 GLY     1       27.647   0.018  13.581
1.00999.99           H
ATOM      7  HT1 GLY     1       27.899  -0.629  16.232
1.00999.99           H
ATOM      8  HT2 GLY     1       27.076   0.858  16.243
1.00999.99           H
ATOM      9  HT3 GLY     1       28.575   0.719  15.454
1.00999.99           H
ATOM     10  N   LEU     2       24.845   0.277  13.361
1.00999.99           N
ATOM     11  CA  LEU     2       23.595   0.999  13.108
1.00895.59           C
ATOM     12  C   LEU     2       22.413   0.226  13.685
1.00532.27           C
ATOM     13  O   LEU     2       22.396  -1.005  13.658
1.00625.21           O
ATOM     14  CB  LEU     2       23.397   1.174  11.599
1.00999.99           C
ATOM     15  CG  LEU     2       24.617   1.871  10.986
1.00999.99           C
ATOM     16  CD1 LEU     2       24.420   1.992   9.473
1.00999.99           C
ATOM     17  CD2 LEU     2       24.788   3.272  11.599
1.00999.99           C
ATOM     18  HN  LEU     2       25.036  -0.541  12.854
1.00999.99           H
ATOM     19  HA  LEU     2       23.635   1.975  13.571
1.00890.87           H
ATOM     20  HB1 LEU     2       22.517   1.774  11.421
1.00924.07           H
ATOM     21  HB2 LEU     2       23.268   0.204  11.141
1.00999.99           H
ATOM     22  HG  LEU     2       25.501   1.280  11.185
1.00999.99           H
ATOM     23 HD11 LEU     2       24.072   1.048   9.078
1.00999.99           H
ATOM     24 HD12 LEU     2       25.358   2.253   9.007
1.00999.99           H
ATOM     25 HD13 LEU     2       23.689   2.759   9.266
1.00999.99           H
ATOM     26 HD21 LEU     2       25.314   3.192  12.539
1.00999.99           H
```

ATOM 27 HD22 LEU 2 23.818 3.719 11.766
1.00999.99 H
ATOM 28 HD23 LEU 2 25.358 3.898 10.925
1.00999.99 H
ATOM 29 N CYS 3 21.427 0.951 14.206
1.00271.28 N
ATOM 30 CA CYS 3 20.250 0.309 14.781
1.00104.50 C
ATOM 31 C CYS 3 19.485 -0.451 13.701 1.00
90.98 C
ATOM 32 O CYS 3 19.395 0.001 12.560
1.00207.28 O
ATOM 33 CB CYS 3 19.331 1.358 15.415 1.00
37.83 C
ATOM 34 SG CYS 3 20.176 2.154 16.804 1.00
81.56 S
ATOM 35 HN CYS 3 21.491 1.929 14.200
1.00296.55 H
ATOM 36 HA CYS 3 20.566 -0.387 15.544
1.00166.45 H
ATOM 37 HB1 CYS 3 18.430 0.879 15.769 1.00
40.38 H
ATOM 38 HB2 CYS 3 19.074 2.103 14.677 1.00
98.45 H
ATOM 39 N SER 4 18.938 -1.605 14.067 1.00
85.31 N
ATOM 40 CA SER 4 18.185 -2.416 13.115 1.00
92.94 C
ATOM 41 C SER 4 16.994 -1.637 12.591 1.00
67.37 C
ATOM 42 O SER 4 16.689 -1.662 11.399
1.00102.28 O
ATOM 43 CB SER 4 17.663 -3.676 13.799
1.00123.83 C
ATOM 44 OG SER 4 17.057 -4.519 12.828
1.00178.89 O
ATOM 45 HN SER 4 19.042 -1.917 14.990
1.00163.03 H
ATOM 46 HA SER 4 18.825 -2.697 12.293
1.00129.78 H
ATOM 47 HB1 SER 4 16.926 -3.389 14.545
1.00105.26 H
ATOM 48 HB2 SER 4 18.474 -4.201 14.276
1.00156.87 H
ATOM 49 HG SER 4 17.749 -4.855 12.254
1.00218.16 H
ATOM 50 N GLU 5 16.310 -0.966 13.509 1.00
41.67 N
ATOM 51 CA GLU 5 15.125 -0.193 13.169 1.00
41.21 C
ATOM 52 C GLU 5 14.990 1.010 14.096 1.00
41.92 C

```
ATOM     53  O   GLU     5       15.939   1.380  14.787  1.00
74.72           O
ATOM     54  CB  GLU     5       13.893  -1.084  13.290  1.00
49.24           C
ATOM     55  CG  GLU     5       13.828  -1.654  14.701  1.00
55.48           C
ATOM     56  CD  GLU     5       12.745  -2.726  14.785  1.00
92.06           C
ATOM     57  OE1 GLU     5       12.109  -2.978  13.775
1.00206.49           O
ATOM     58  OE2 GLU     5       12.568  -3.279  15.858
1.00197.24           O1-
ATOM     59  HN  GLU     5       16.601  -1.007  14.443  1.00
45.52           H
ATOM     60  HA  GLU     5       15.202   0.151  12.157  1.00
60.63           H
ATOM     61  HB1 GLU     5       13.959  -1.893  12.580  1.00
61.59           H
ATOM     62  HB2 GLU     5       13.010  -0.505  13.096  1.00
63.66           H
ATOM     63  HG1 GLU     5       13.603  -0.862  15.399  1.00
58.03           H
ATOM     64  HG2 GLU     5       14.783  -2.087  14.944  1.00
57.77           H
ATOM     65  N   ASN     6       13.810   1.618  14.106  1.00
50.73           N
ATOM     66  CA  ASN     6       13.573   2.778  14.955  1.00
71.04           C
ATOM     67  C   ASN     6       13.786   2.411  16.419  1.00
65.17           C
ATOM     68  O   ASN     6       14.347   3.189  17.191  1.00
94.86           O
ATOM     69  CB  ASN     6       12.146   3.290  14.756  1.00
94.83           C
ATOM     70  CG  ASN     6       12.013   3.947  13.387
1.00160.94           C
ATOM     71  ND2 ASN     6       10.830   4.086  12.852
1.00244.88           N
ATOM     72  OD1 ASN     6       13.013   4.342  12.787
1.00219.73           O
ATOM     73  HN  ASN     6       13.088   1.282  13.535  1.00
72.81           H
ATOM     74  HA  ASN     6       14.267   3.560  14.684  1.00
96.02           H
ATOM     75  HB1 ASN     6       11.914   4.013  15.523
1.00111.09           H
ATOM     76  HB2 ASN     6       11.456   2.461  14.824  1.00
98.13           H
ATOM     77 HD21 ASN     6       10.036   3.770  13.331
1.00272.43           H
ATOM     78 HD22 ASN     6       10.736   4.508  11.972
1.00336.78           H
```

```
ATOM     79  N   GLY     7      13.341   1.211  16.793  1.00
51.80           N
ATOM     80  CA  GLY     7      13.487   0.726  18.168  1.00
71.47           C
ATOM     81  C   GLY     7      14.129  -0.655  18.182  1.00
41.04           C
ATOM     82  O   GLY     7      13.452  -1.665  18.372  1.00
44.54           O
ATOM     83  HN  GLY     7      12.908   0.636  16.129  1.00
45.49           H
ATOM     84  HA1 GLY     7      12.516   0.665  18.626
1.00103.52           H
ATOM     85  HA2 GLY     7      14.105   1.411  18.737
1.00108.67           H
ATOM     86  N   ASP     8      15.439  -0.689  17.978  1.00
27.00           N
ATOM     87  CA  ASP     8      16.171  -1.951  17.963  1.00
14.15           C
ATOM     88  C   ASP     8      16.052  -2.656  19.308  1.00
9.37           C
ATOM     89  O   ASP     8      15.837  -3.866  19.370  1.00
14.61           O
ATOM     90  CB  ASP     8      17.646  -1.698  17.648  1.00
15.06           C
ATOM     91  CG  ASP     8      18.388  -3.024  17.529  1.00
21.22           C
ATOM     92  OD1 ASP     8      19.238  -3.282  18.366
1.00119.56           O
ATOM     93  OD2 ASP     8      18.097  -3.764  16.604
1.00133.27           O1-
ATOM     94  HN  ASP     8      15.921   0.150  17.831  1.00
35.63           H
ATOM     95  HA  ASP     8      15.756  -2.586  17.200  1.00
20.92           H
ATOM     96  HB1 ASP     8      18.086  -1.112  18.441  1.00
29.39           H
ATOM     97  HB2 ASP     8      17.726  -1.157  16.716  1.00
44.14           H
ATOM     98  N   CYS     9      16.192  -1.889  20.380  1.00
5.93           N
ATOM     99  CA  CYS     9      16.097  -2.446  21.724  1.00
7.54           C
ATOM    100  C   CYS     9      14.711  -3.034  21.956  1.00
14.60           C
ATOM    101  O   CYS     9      14.564  -4.108  22.538  1.00
25.78           O
ATOM    102  CB  CYS     9      16.383  -1.361  22.752  1.00
6.63           C
ATOM    103  SG  CYS     9      18.134  -0.953  22.669  1.00
10.91           S
ATOM    104  HN  CYS     9      16.360  -0.932  20.264  1.00
6.51           H
```

```
ATOM    105  HA  CYS     9      16.838  -3.220  21.832  1.00
11.12           H
ATOM    106  HB1 CYS     9      16.146  -1.719  23.741  1.00
11.28           H
ATOM    107  HB2 CYS     9      15.794  -0.484  22.531  1.00
5.04           H
ATOM    108  N   ALA    10      13.700  -2.316  21.484  1.00
16.26           N
ATOM    109  CA  ALA    10      12.317  -2.755  21.623  1.00
32.04           C
ATOM    110  C   ALA    10      11.401  -1.864  20.785  1.00
45.47           C
ATOM    111  O   ALA    10      11.811  -0.797  20.332
1.00119.04           O
ATOM    112  CB  ALA    10      11.892  -2.712  23.094  1.00
30.10           C
ATOM    113  HN  ALA    10      13.890  -1.472  21.024  1.00
12.81           H
ATOM    114  HA  ALA    10      12.237  -3.771  21.265  1.00
47.14           H
ATOM    115  HB1 ALA    10      12.723  -3.012  23.717  1.00
84.37           H
ATOM    116  HB2 ALA    10      11.064  -3.388  23.252
1.00118.20           H
ATOM    117  HB3 ALA    10      11.591  -1.709  23.357
1.00117.32           H
ATOM    118  N   ALA    11      10.166  -2.306  20.580  1.00
30.62           N
ATOM    119  CA  ALA    11       9.218  -1.530  19.790  1.00
35.24           C
ATOM    120  C   ALA    11       8.972  -0.164  20.428  1.00
22.78           C
ATOM    121  O   ALA    11       8.916   0.853  19.736  1.00
53.33           O
ATOM    122  CB  ALA    11       7.892  -2.286  19.677  1.00
60.49           C
ATOM    123  HN  ALA    11       9.890  -3.165  20.963  1.00
50.37           H
ATOM    124  HA  ALA    11       9.621  -1.386  18.800  1.00
41.70           H
ATOM    125  HB1 ALA    11       7.586  -2.626  20.656
1.00157.06           H
ATOM    126  HB2 ALA    11       8.018  -3.137  19.024
1.00148.83           H
ATOM    127  HB3 ALA    11       7.136  -1.629  19.271
1.00137.06           H
ATOM    128  N   ASP    12       8.829  -0.146  21.750  1.00
18.98           N
ATOM    129  CA  ASP    12       8.590   1.102  22.470  1.00
31.05           C
ATOM    130  C   ASP    12       9.896   1.863  22.693  1.00
22.98           C
```

```
ATOM    131  O   ASP    12        9.914   3.094  22.700  1.00
37.14          O
ATOM    132  CB  ASP    12        7.937   0.805  23.821  1.00
48.35          C
ATOM    133  CG  ASP    12        6.515   0.295  23.613
1.00104.83          C
ATOM    134  OD1 ASP    12        5.955  -0.242  24.554
1.00258.73          O
ATOM    135  OD2 ASP    12        6.008   0.450  22.514
1.00213.46          O1-
ATOM    136  HN  ASP    12        8.883  -0.987  22.251  1.00
36.19          H
ATOM    137  HA  ASP    12        7.920   1.719  21.891  1.00
49.76          H
ATOM    138  HB1 ASP    12        7.910   1.708  24.412  1.00
95.71          H
ATOM    139  HB2 ASP    12        8.514   0.053  24.341  1.00
54.37          H
ATOM    140  N   GLU    13       10.982   1.121  22.882  1.00
17.60          N
ATOM    141  CA  GLU    13       12.287   1.733  23.117  1.00
12.01          C
ATOM    142  C   GLU    13       12.863   2.322  21.831  1.00
10.07          C
ATOM    143  O   GLU    13       12.481   1.929  20.728  1.00
12.81          O
ATOM    144  CB  GLU    13       13.252   0.694  23.683  1.00
11.74          C
ATOM    145  CG  GLU    13       12.753   0.227  25.053  1.00
13.49          C
ATOM    146  CD  GLU    13       12.899   1.351  26.072
1.00142.36          C
ATOM    147  OE1 GLU    13       13.599   2.304  25.775
1.00339.06          O
ATOM    148  OE2 GLU    13       12.308   1.243  27.133
1.00335.28          O1-
ATOM    149  HN  GLU    13       10.904   0.145  22.873  1.00
28.36          H
ATOM    150  HA  GLU    13       12.172   2.526  23.841  1.00
13.06          H
ATOM    151  HB1 GLU    13       14.231   1.133  23.790  1.00
13.76          H
ATOM    152  HB2 GLU    13       13.308  -0.150  23.011  1.00
9.94          H
ATOM    153  HG1 GLU    13       13.330  -0.626  25.375  1.00
66.02          H
ATOM    154  HG2 GLU    13       11.711  -0.051  24.979  1.00
56.23          H
ATOM    155  N   CYS    14       13.788   3.274  21.986  1.00
9.12          N
ATOM    156  CA  CYS    14       14.429   3.931  20.842  1.00
10.69          C
```

ATOM 157 C CYS 14 15.874 3.467 20.697 1.00
9.56 C
ATOM 158 O CYS 14 16.503 3.057 21.670 1.00
13.62 O
ATOM 159 CB CYS 14 14.403 5.448 21.035 1.00
13.96 C
ATOM 160 SG CYS 14 15.364 6.239 19.719 1.00
42.96 S
ATOM 161 HN CYS 14 14.048 3.540 22.894 1.00
9.76 H
ATOM 162 HA CYS 14 13.890 3.688 19.936 1.00
15.54 H
ATOM 163 HB1 CYS 14 14.832 5.696 21.994 1.00
50.72 H
ATOM 164 HB2 CYS 14 13.382 5.799 20.996 1.00
46.13 H
ATOM 165 N CYS 15 16.397 3.540 19.473 1.00
9.60 N
ATOM 166 CA CYS 15 17.777 3.134 19.197 1.00
9.28 C
ATOM 167 C CYS 15 18.387 4.070 18.164 1.00
9.92 C
ATOM 168 O CYS 15 17.807 4.290 17.101 1.00
13.31 O
ATOM 169 CB CYS 15 17.805 1.700 18.658 1.00
12.90 C
ATOM 170 SG CYS 15 19.521 1.161 18.440 1.00
39.11 S
ATOM 171 HN CYS 15 15.847 3.883 18.737 1.00
13.12 H
ATOM 172 HA CYS 15 18.360 3.182 20.106 1.00
8.26 H
ATOM 173 HB1 CYS 15 17.293 1.664 17.708 1.00
24.82 H
ATOM 174 HB2 CYS 15 17.309 1.044 19.358 1.00
27.31 H
ATOM 175 N VAL 16 19.557 4.622 18.472 1.00
9.56 N
ATOM 176 CA VAL 16 20.219 5.531 17.544 1.00
11.99 C
ATOM 177 C VAL 16 21.726 5.460 17.736 1.00
7.73 C
ATOM 178 O VAL 16 22.225 5.581 18.855 1.00
8.83 O
ATOM 179 CB VAL 16 19.721 6.958 17.775 1.00
18.37 C
ATOM 180 CG1 VAL 16 20.155 7.441 19.160 1.00
39.61 C
ATOM 181 CG2 VAL 16 20.300 7.879 16.703
1.00115.21 C
ATOM 182 HN VAL 16 19.987 4.415 19.334 1.00
9.76 H

```
ATOM    183  HA  VAL    16      19.985   5.239  16.529  1.00
17.27          H
ATOM    184  HB  VAL    16      18.641   6.973  17.716  1.00
52.63          H
ATOM    185 HG11 VAL    16      19.591   8.323  19.427
1.00128.07          H
ATOM    186 HG12 VAL    16      21.209   7.679  19.145
1.00154.13          H
ATOM    187 HG13 VAL    16      19.971   6.663  19.886
1.00135.74          H
ATOM    188 HG21 VAL    16      19.978   7.541  15.729
1.00229.05          H
ATOM    189 HG22 VAL    16      21.378   7.858  16.754
1.00261.82          H
ATOM    190 HG23 VAL    16      19.951   8.887  16.868
1.00210.07          H
ATOM    191  N   ASP    17      22.448   5.247  16.637  1.00
14.34          N
ATOM    192  CA  ASP    17      23.907   5.142  16.692  1.00
11.90          C
ATOM    193  C   ASP    17      24.575   6.370  16.090  1.00
13.15          C
ATOM    194  O   ASP    17      24.428   6.656  14.902  1.00
25.96          O
ATOM    195  CB  ASP    17      24.362   3.892  15.941  1.00
20.80          C
ATOM    196  CG  ASP    17      23.758   2.651  16.593  1.00
28.72          C
ATOM    197  OD1 ASP    17      23.229   1.823  15.872
1.00135.42          O
ATOM    198  OD2 ASP    17      23.835   2.549  17.806
1.00114.17          O1-
ATOM    199  HN  ASP    17      21.993   5.147  15.775  1.00
28.29          H
ATOM    200  HA  ASP    17      24.220   5.053  17.719  1.00
10.49          H
ATOM    201  HB1 ASP    17      25.439   3.826  15.978  1.00
22.42          H
ATOM    202  HB2 ASP    17      24.040   3.952  14.912  1.00
29.52          H
ATOM    203  N   THR    18      25.333   7.077  16.926  1.00
11.55          N
ATOM    204  CA  THR    18      26.060   8.267  16.491  1.00
17.10          C
ATOM    205  C   THR    18      27.530   7.923  16.306  1.00
10.63          C
ATOM    206  O   THR    18      27.988   6.876  16.761  1.00
6.49          O
ATOM    207  CB  THR    18      25.925   9.381  17.533  1.00
28.52          C
ATOM    208  CG2 THR    18      24.511   9.955  17.496  1.00
45.38          C
```

```
ATOM    209  OG1 THR    18       26.197   8.858  18.823  1.00
26.49           O
ATOM    210  HN  THR    18       25.418   6.781  17.856  1.00
13.96           H
ATOM    211  HA  THR    18       25.658   8.617  15.550  1.00
26.43           H
ATOM    212  HB  THR    18       26.630  10.166  17.309  1.00
37.43           H
ATOM    213  HG1 THR    18       25.702   8.040  18.921  1.00
71.62           H
ATOM    214 HG21 THR    18       24.276  10.266  16.489
1.00100.44           H
ATOM    215 HG22 THR    18       24.452  10.806  18.158
1.00129.42           H
ATOM    216 HG23 THR    18       23.807   9.200  17.814
1.00156.21           H
ATOM    217  N   VAL    19       28.264   8.814  15.653  1.00
16.32           N
ATOM    218  CA  VAL    19       29.672   8.610  15.422  1.00
13.34           C
ATOM    219  C   VAL    19       30.236   9.792  14.667  1.00
25.88           C
ATOM    220  O   VAL    19       29.846  10.089  13.538  1.00
42.11           O
ATOM    221  CB  VAL    19       29.941   7.326  14.637  1.00
15.48           C
ATOM    222  CG1 VAL    19       29.094   7.297  13.360  1.00
29.06           C
ATOM    223  CG2 VAL    19       31.431   7.276  14.270  1.00
20.67           C
ATOM    224  HN  VAL    19       27.856   9.638  15.332  1.00
26.89           H
ATOM    225  HA  VAL    19       30.170   8.540  16.378  1.00
8.08           H
ATOM    226  HB  VAL    19       29.695   6.478  15.252  1.00
11.49           H
ATOM    227 HG11 VAL    19       29.529   7.953  12.621
1.00137.43           H
ATOM    228 HG12 VAL    19       28.089   7.625  13.584  1.00
93.29           H
ATOM    229 HG13 VAL    19       29.063   6.289  12.973
1.00108.69           H
ATOM    230 HG21 VAL    19       32.024   7.577  15.125  1.00
99.42           H
ATOM    231 HG22 VAL    19       31.621   7.956  13.451
1.00111.36           H
ATOM    232 HG23 VAL    19       31.700   6.273  13.977  1.00
77.11           H
ATOM    233  N   PHE    20       31.150  10.459  15.322  1.00
24.58           N
ATOM    234  CA  PHE    20       31.806  11.629  14.769  1.00
40.34           C
```

```
ATOM    235  C   PHE    20       33.134  11.251  14.127  1.00
40.37          C
ATOM    236  O   PHE    20       33.328  11.452  12.928  1.00
66.02          O
ATOM    237  CB  PHE    20       32.027  12.669  15.884  1.00
48.79          C
ATOM    238  CG  PHE    20       32.191  11.996  17.240  1.00
35.75          C
ATOM    239  CD1 PHE    20       31.115  11.308  17.837  1.00
30.20          C
ATOM    240  CD2 PHE    20       33.416  12.081  17.915  1.00
40.95          C
ATOM    241  CE1 PHE    20       31.277  10.709  19.090  1.00
30.41          C
ATOM    242  CE2 PHE    20       33.573  11.478  19.168  1.00
45.84          C
ATOM    243  CZ  PHE    20       32.505  10.794  19.755  1.00
40.81          C
ATOM    244  HN  PHE    20       31.382  10.156  16.215  1.00
16.73          H
ATOM    245  HA  PHE    20       31.171  12.067  14.008  1.00
59.91          H
ATOM    246  HB1 PHE    20       31.181  13.315  15.920  1.00
67.15          H
ATOM    247  HB2 PHE    20       32.906  13.261  15.667  1.00
58.17          H
ATOM    248  HD1 PHE    20       30.162  11.231  17.328  1.00
33.34          H
ATOM    249  HD2 PHE    20       34.243  12.608  17.465  1.00
49.35          H
ATOM    250  HE1 PHE    20       30.454  10.183  19.545  1.00
30.61          H
ATOM    251  HE2 PHE    20       34.521  11.543  19.682  1.00
61.27          H
ATOM    252  HZ  PHE    20       32.628  10.330  20.723  1.00
52.66          H
ATOM    253  N   GLU    21       34.056  10.732  14.932  1.00
41.31          N
ATOM    254  CA  GLU    21       35.368  10.366  14.450  1.00
56.16          C
ATOM    255  C   GLU    21       35.592   8.862  14.544  1.00
58.61          C
ATOM    256  O   GLU    21       34.670   8.099  14.838
1.00201.19          O
ATOM    257  CB  GLU    21       36.371  11.102  15.308  1.00
57.37          C
ATOM    258  CG  GLU    21       36.298  10.579  16.739
1.00193.56          C
ATOM    259  CD  GLU    21       37.080  11.500  17.670
1.00304.62          C
ATOM    260  OE1 GLU    21       37.019  11.289  18.869
1.00451.81          O
```

ATOM 261 OE2 GLU 21 37.728 12.404 17.168
1.00442.88 O1-
ATOM 262 HN GLU 21 33.868 10.620 15.883 1.00
50.82 H
ATOM 263 HA GLU 21 35.492 10.679 13.422 1.00
85.72 H
ATOM 264 HB1 GLU 21 36.126 12.153 15.301 1.00
46.37 H
ATOM 265 HB2 GLU 21 37.354 10.952 14.915
1.00131.81 H
ATOM 266 HG1 GLU 21 36.717 9.590 16.775
1.00314.91 H
ATOM 267 HG2 GLU 21 35.270 10.537 17.052
1.00266.92 H
ATOM 268 N GLY 22 36.825 8.444 14.277 1.00
86.70 N
ATOM 269 CA GLY 22 37.181 7.023 14.314
1.00104.66 C
ATOM 270 C GLY 22 37.774 6.620 15.663 1.00
71.50 C
ATOM 271 O GLY 22 37.923 5.433 15.953 1.00
89.87 O
ATOM 272 HN GLY 22 37.508 9.109 14.043
1.00214.64 H
ATOM 273 HA1 GLY 22 37.908 6.825 13.542
1.00152.47 H
ATOM 274 HA2 GLY 22 36.299 6.425 14.126
1.00118.15 H
ATOM 275 N ASP 23 38.122 7.609 16.479 1.00
44.68 N
ATOM 276 CA ASP 23 38.711 7.334 17.787 1.00
43.33 C
ATOM 277 C ASP 23 37.750 6.544 18.669 1.00
37.55 C
ATOM 278 O ASP 23 38.162 5.633 19.388 1.00
61.37 O
ATOM 279 CB ASP 23 39.072 8.645 18.486 1.00
40.74 C
ATOM 280 CG ASP 23 40.258 9.301 17.786
1.00141.86 C
ATOM 281 OD1 ASP 23 40.507 10.465 18.053
1.00328.18 O
ATOM 282 OD2 ASP 23 40.898 8.631 16.994
1.00304.58 O1-
ATOM 283 HN ASP 23 37.993 8.536 16.193 1.00
40.86 H
ATOM 284 HA ASP 23 39.609 6.758 17.649 1.00
72.12 H
ATOM 285 HB1 ASP 23 39.332 8.443 19.513 1.00
94.15 H
ATOM 286 HB2 ASP 23 38.222 9.311 18.459 1.00
93.26 H

ATOM 287 N MET 24 36.474 6.903 18.619 1.00
23.24 N
ATOM 288 CA MET 24 35.466 6.225 19.431 1.00
32.50 C
ATOM 289 C MET 24 34.075 6.392 18.824 1.00
25.13 C
ATOM 290 O MET 24 33.805 7.370 18.125 1.00
54.40 O
ATOM 291 CB MET 24 35.477 6.791 20.853 1.00
47.36 C
ATOM 292 CG MET 24 34.443 6.054 21.707
1.00151.73 C
ATOM 293 SD MET 24 34.603 6.576 23.434
1.00209.23 S
ATOM 294 CE MET 24 33.987 8.268 23.234
1.00243.29 C
ATOM 295 HN MET 24 36.207 7.641 18.033 1.00
17.32 H
ATOM 296 HA MET 24 35.700 5.171 19.476 1.00
48.94 H
ATOM 297 HB1 MET 24 35.233 7.842 20.822
1.00124.40 H
ATOM 298 HB2 MET 24 36.460 6.662 21.283
1.00166.96 H
ATOM 299 HG1 MET 24 34.612 4.990 21.637
1.00331.71 H
ATOM 300 HG2 MET 24 33.450 6.284 21.351
1.00302.30 H
ATOM 301 HE1 MET 24 33.643 8.639 24.189
1.00340.44 H
ATOM 302 HE2 MET 24 33.168 8.276 22.533
1.00373.88 H
ATOM 303 HE3 MET 24 34.783 8.899 22.863
1.00386.81 H
ATOM 304 N VAL 25 33.195 5.427 19.098 1.00
22.44 N
ATOM 305 CA VAL 25 31.823 5.458 18.583 1.00
15.37 C
ATOM 306 C VAL 25 30.821 5.579 19.727 1.00
17.47 C
ATOM 307 O VAL 25 30.993 4.983 20.790 1.00
29.52 O
ATOM 308 CB VAL 25 31.530 4.190 17.777 1.00
23.36 C
ATOM 309 CG1 VAL 25 30.045 4.165 17.371 1.00
58.39 C
ATOM 310 CG2 VAL 25 32.410 4.179 16.523 1.00
55.25 C
ATOM 311 HN VAL 25 33.474 4.676 19.662 1.00
48.11 H
ATOM 312 HA VAL 25 31.702 6.317 17.931 1.00
9.25 H

ATOM 313 HB VAL 25 31.751 3.322 18.382 1.00
52.45 H
ATOM 314 HG11 VAL 25 29.459 3.768 18.186
1.00171.07 H
ATOM 315 HG12 VAL 25 29.915 3.542 16.499
1.00166.22 H
ATOM 316 HG13 VAL 25 29.712 5.170 17.149
1.00134.76 H
ATOM 317 HG21 VAL 25 33.446 4.074 16.811
1.00141.88 H
ATOM 318 HG22 VAL 25 32.278 5.104 15.983
1.00184.50 H
ATOM 319 HG23 VAL 25 32.126 3.349 15.891
1.00133.90 H
ATOM 320 N THR 26 29.778 6.372 19.494 1.00
13.60 N
ATOM 321 CA THR 26 28.735 6.607 20.494 1.00
21.19 C
ATOM 322 C THR 26 27.410 5.965 20.079 1.00
16.55 C
ATOM 323 O THR 26 26.958 6.126 18.946 1.00
11.43 O
ATOM 324 CB THR 26 28.545 8.114 20.651 1.00
27.07 C
ATOM 325 CG2 THR 26 27.365 8.402 21.581 1.00
44.39 C
ATOM 326 OG1 THR 26 29.730 8.681 21.191 1.00
34.75 O
ATOM 327 HN THR 26 29.711 6.823 18.626 1.00
10.81 H
ATOM 328 HA THR 26 29.042 6.197 21.446 1.00
33.16 H
ATOM 329 HB THR 26 28.351 8.547 19.678 1.00
20.60 H
ATOM 330 HG1 THR 26 30.262 7.968 21.551 1.00
77.72 H
ATOM 331 HG21 THR 26 27.442 7.783 22.463
1.00126.99 H
ATOM 332 HG22 THR 26 26.439 8.183 21.069
1.00108.32 H
ATOM 333 HG23 THR 26 27.379 9.443 21.870
1.00151.99 H
ATOM 334 N ARG 27 26.789 5.242 21.016 1.00
22.38 N
ATOM 335 CA ARG 27 25.504 4.580 20.762 1.00
20.21 C
ATOM 336 C ARG 27 24.543 4.832 21.922 1.00
16.96 C
ATOM 337 O ARG 27 24.973 4.964 23.068 1.00
20.06 O
ATOM 338 CB ARG 27 25.717 3.074 20.594 1.00
22.14 C

```
ATOM    339  CG  ARG    27       26.631   2.814  19.394
1.00124.30           C
ATOM    340  CD  ARG    27       27.013   1.333  19.355
1.00109.38           C
ATOM    341  NE  ARG    27       27.805   0.985  20.527
1.00227.73           N
ATOM    342  CZ  ARG    27       28.179  -0.270  20.755
1.00426.12           C
ATOM    343  NH1 ARG    27       28.885  -0.559  21.813
1.00767.09           N1+
ATOM    344  NH2 ARG    27       27.839  -1.213  19.919
1.00581.78           N
ATOM    345  HN  ARG    27       27.199   5.159  21.903  1.00
30.75           H
ATOM    346  HA  ARG    27       25.067   4.975  19.858  1.00
23.13           H
ATOM    347  HB1 ARG    27       24.765   2.592  20.428  1.00
87.46           H
ATOM    348  HB2 ARG    27       26.172   2.672  21.488
1.00103.36           H
ATOM    349  HG1 ARG    27       27.525   3.411  19.486
1.00281.97           H
ATOM    350  HG2 ARG    27       26.114   3.077  18.483
1.00276.19           H
ATOM    351  HD1 ARG    27       27.587   1.137  18.461
1.00183.60           H
ATOM    352  HD2 ARG    27       26.116   0.733  19.340
1.00142.93           H
ATOM    353  HE  ARG    27       28.065   1.687  21.160
1.00372.53           H
ATOM    354 HH11 ARG    27       29.145   0.165  22.453
1.00910.59           H
ATOM    355 HH12 ARG    27       29.167  -1.502  21.985
1.00999.99           H
ATOM    356 HH21 ARG    27       27.298  -0.991  19.108
1.00532.54           H
ATOM    357 HH22 ARG    27       28.120  -2.157  20.090
1.00948.84           H
ATOM    358  N   SER    28       23.238   4.900  21.628  1.00
14.60           N
ATOM    359  CA  SER    28       22.239   5.140  22.673  1.00
14.92           C
ATOM    360  C   SER    28       21.016   4.255  22.465  1.00
12.12           C
ATOM    361  O   SER    28       20.648   3.940  21.333  1.00
13.41           O
ATOM    362  CB  SER    28       21.817   6.610  22.661  1.00
23.26           C
ATOM    363  OG  SER    28       22.945   7.425  22.954
1.00146.32           O
ATOM    364  HN  SER    28       22.941   4.788  20.696  1.00
15.23           H
```

ATOM 365 HA SER 28 22.668 4.912 23.640 1.00
15.44 H
ATOM 366 HB1 SER 28 21.044 6.767 23.401 1.00
88.73 H
ATOM 367 HB2 SER 28 21.438 6.870 21.688
1.00124.68 H
ATOM 368 HG SER 28 23.093 7.396 23.902
1.00242.47 H
ATOM 369 N CYS 29 20.387 3.862 23.568 1.00
10.48 N
ATOM 370 CA CYS 29 19.200 3.019 23.505 1.00
9.97 C
ATOM 371 C CYS 29 18.592 2.872 24.898 1.00
11.60 C
ATOM 372 O CYS 29 19.215 2.311 25.799 1.00
15.64 O
ATOM 373 CB CYS 29 19.563 1.637 22.934 1.00
9.52 C
ATOM 374 SG CYS 29 18.154 0.958 22.043 1.00
10.40 S
ATOM 375 HN CYS 29 20.724 4.152 24.441 1.00
11.10 H
ATOM 376 HA CYS 29 18.477 3.488 22.856 1.00
11.08 H
ATOM 377 HB1 CYS 29 19.824 0.956 23.736 1.00
9.33 H
ATOM 378 HB2 CYS 29 20.397 1.729 22.259 1.00
10.78 H
ATOM 379 N GLU 30 17.376 3.381 25.071 1.00
13.52 N
ATOM 380 CA GLU 30 16.705 3.300 26.365 1.00
17.41 C
ATOM 381 C GLU 30 16.074 1.925 26.558 1.00
10.81 C
ATOM 382 O GLU 30 15.721 1.252 25.592 1.00
23.22 O
ATOM 383 CB GLU 30 15.627 4.380 26.462 1.00
37.83 C
ATOM 384 CG GLU 30 16.278 5.760 26.339
1.00108.13 C
ATOM 385 CD GLU 30 17.167 6.027 27.548
1.00240.52 C
ATOM 386 OE1 GLU 30 18.033 6.881 27.446
1.00422.56 O
ATOM 387 OE2 GLU 30 16.968 5.377 28.561
1.00410.81 O1-
ATOM 388 HN GLU 30 16.923 3.819 24.320 1.00
15.61 H
ATOM 389 HA GLU 30 17.431 3.462 27.148 1.00
24.60 H
ATOM 390 HB1 GLU 30 15.125 4.306 27.415 1.00
72.67 H

```
ATOM    391  HB2 GLU    30       14.911   4.248  25.664  1.00
56.47           H
ATOM    392  HG1 GLU    30       15.509   6.514  26.287
1.00196.66           H
ATOM    393  HG2 GLU    30       16.876   5.793  25.440
1.00140.67           H
ATOM    394  N   LYS    31       15.937   1.513  27.819  1.00
11.96           N
ATOM    395  CA  LYS    31       15.345   0.214  28.147  1.00
11.40           C
ATOM    396  C   LYS    31       14.060   0.407  28.940  1.00
10.99           C
ATOM    397  O   LYS    31       13.957   1.318  29.762  1.00
12.86           O
ATOM    398  CB  LYS    31       16.332  -0.611  28.975  1.00
21.40           C
ATOM    399  CG  LYS    31       17.585  -0.894  28.144  1.00
57.81           C
ATOM    400  CD  LYS    31       18.568  -1.726  28.969
1.00115.52           C
ATOM    401  CE  LYS    31       19.839  -1.973  28.154
1.00250.23           C
ATOM    402  NZ  LYS    31       19.503  -2.764  26.936
1.00462.10           N1+
ATOM    403  HN  LYS    31       16.240   2.097  28.546  1.00
26.13           H
ATOM    404  HA  LYS    31       15.119  -0.326  27.237  1.00
13.87           H
ATOM    405  HB1 LYS    31       15.870  -1.547  29.256  1.00
37.63           H
ATOM    406  HB2 LYS    31       16.603  -0.062  29.863  1.00
47.19           H
ATOM    407  HG1 LYS    31       18.052   0.039  27.867
1.00126.32           H
ATOM    408  HG2 LYS    31       17.311  -1.438  27.252
1.00114.19           H
ATOM    409  HD1 LYS    31       18.116  -2.674  29.221
1.00198.45           H
ATOM    410  HD2 LYS    31       18.818  -1.194  29.875
1.00200.14           H
ATOM    411  HE1 LYS    31       20.551  -2.520  28.753
1.00374.64           H
ATOM    412  HE2 LYS    31       20.267  -1.026  27.861
1.00403.27           H
ATOM    413  HZ1 LYS    31       19.970  -2.343  26.108
1.00627.38           H
ATOM    414  HZ2 LYS    31       18.471  -2.758  26.793
1.00622.26           H
ATOM    415  HZ3 LYS    31       19.831  -3.743  27.057
1.00619.20           H
ATOM    416  N   THR    32       13.079  -0.451  28.685  1.00
15.51           N
```

```
ATOM    417  CA  THR    32       11.803  -0.360  29.381  1.00
22.44           C
ATOM    418  C   THR    32       11.944  -0.845  30.819  1.00
26.60           C
ATOM    419  O   THR    32       12.396  -1.963  31.068  1.00
57.43           O
ATOM    420  CB  THR    32       10.751  -1.200  28.649  1.00
56.28           C
ATOM    421  CG2 THR    32        9.399  -1.068  29.354
1.00102.70           C
ATOM    422  OG1 THR    32       10.631  -0.740  27.311
1.00111.17           O
ATOM    423  HN  THR    32       13.216  -1.155  28.018  1.00
18.79           H
ATOM    424  HA  THR    32       11.481   0.671  29.388  1.00
19.67           H
ATOM    425  HB  THR    32       11.051  -2.235  28.649  1.00
84.25           H
ATOM    426  HG1 THR    32       10.676  -1.503  26.730
1.00205.03           H
ATOM    427 HG21 THR    32        9.470  -1.472  30.354
1.00220.71           H
ATOM    428 HG22 THR    32        8.651  -1.613  28.800
1.00174.67           H
ATOM    429 HG23 THR    32        9.122  -0.025  29.407
1.00217.44           H
ATOM    430  N   THR    33       11.553   0.006  31.761  1.00
22.47           N
ATOM    431  CA  THR    33       11.634  -0.334  33.178  1.00
41.60           C
ATOM    432  C   THR    33       10.602   0.428  33.964  1.00
38.03           C
ATOM    433  O   THR    33       10.859   1.521  34.469  1.00
52.73           O
ATOM    434  CB  THR    33       13.031  -0.032  33.725  1.00
63.25           C
ATOM    435  CG2 THR    33       14.048  -0.997  33.111
1.00121.65           C
ATOM    436  OG1 THR    33       13.386   1.304  33.396  1.00
96.20           O
ATOM    437  HN  THR    33       11.202   0.882  31.498  1.00
23.82           H
ATOM    438  HA  THR    33       11.422  -1.377  33.312  1.00
67.12           H
ATOM    439  HB  THR    33       13.031  -0.150  34.797
1.00107.59           H
ATOM    440  HG1 THR    33       13.928   1.651  34.109
1.00176.27           H
ATOM    441 HG21 THR    33       13.663  -2.004  33.158
1.00255.23           H
ATOM    442 HG22 THR    33       14.975  -0.941  33.663
1.00245.37           H
```

```
ATOM    443 HG23 THR    33      14.226  -0.726  32.081
1.00185.78          H
ATOM    444  N   GLY    34       9.432  -0.175  34.077  1.00
40.93          N
ATOM    445  CA  GLY    34       8.363   0.437  34.822  1.00
45.59          C
ATOM    446  C   GLY    34       7.786   1.630  34.070  1.00
40.97          C
ATOM    447  O   GLY    34       6.571   1.826  34.034  1.00
82.17          O
ATOM    448  HN  GLY    34       9.295  -1.052  33.664  1.00
54.00          H
ATOM    449  HA1 GLY    34       8.765   0.765  35.759  1.00
54.41          H
ATOM    450  HA2 GLY    34       7.585  -0.292  34.997  1.00
62.09          H
ATOM    451  N   ASN    35       8.670   2.425  33.464  1.00
32.51          N
ATOM    452  CA  ASN    35       8.252   3.603  32.701  1.00
33.20          C
ATOM    453  C   ASN    35       8.983   3.655  31.363  1.00
23.17          C
ATOM    454  O   ASN    35      10.172   3.348  31.281  1.00
27.01          O
ATOM    455  CB  ASN    35       8.555   4.876  33.494  1.00
54.51          C
ATOM    456  CG  ASN    35       7.796   4.860  34.817  1.00
80.73          C
ATOM    457  ND2 ASN    35       8.402   5.234  35.910
1.00220.46          N
ATOM    458  OD1 ASN    35       6.621   4.496  34.855
1.00117.80          O
ATOM    459  HN  ASN    35       9.624   2.212  33.528  1.00
51.15          H
ATOM    460  HA  ASN    35       7.187   3.556  32.513  1.00
47.21          H
ATOM    461  HB1 ASN    35       8.249   5.736  32.917  1.00
68.31          H
ATOM    462  HB2 ASN    35       9.616   4.932  33.689  1.00
57.75          H
ATOM    463 HD21 ASN    35       9.337   5.524  35.878
1.00405.39          H
ATOM    464 HD22 ASN    35       7.920   5.227  36.764
1.00245.64          H
ATOM    465  N   PHE    36       8.260   4.040  30.314  1.00
35.72          N
ATOM    466  CA  PHE    36       8.835   4.128  28.981  1.00
33.41          C
ATOM    467  C   PHE    36       9.455   5.503  28.753  1.00
28.51          C
ATOM    468  O   PHE    36       8.941   6.512  29.237  1.00
45.23          O
```

ATOM 469 CB PHE 36 7.733 3.894 27.952 1.00
67.53 C
ATOM 470 CG PHE 36 7.203 2.487 28.089 1.00
86.71 C
ATOM 471 CD1 PHE 36 6.144 2.225 28.967
1.00106.51 C
ATOM 472 CD2 PHE 36 7.763 1.448 27.338 1.00
99.48 C
ATOM 473 CE1 PHE 36 5.644 0.924 29.092
1.00134.48 C
ATOM 474 CE2 PHE 36 7.265 0.146 27.464
1.00134.27 C
ATOM 475 CZ PHE 36 6.205 -0.116 28.341
1.00149.48 C
ATOM 476 HN PHE 36 7.318 4.268 30.436 1.00
62.87 H
ATOM 477 HA PHE 36 9.594 3.368 28.861 1.00
28.53 H
ATOM 478 HB1 PHE 36 8.132 4.033 26.966 1.00
73.56 H
ATOM 479 HB2 PHE 36 6.932 4.599 28.119 1.00
88.42 H
ATOM 480 HD1 PHE 36 5.713 3.028 29.548
1.00110.20 H
ATOM 481 HD2 PHE 36 8.582 1.648 26.662 1.00
93.07 H
ATOM 482 HE1 PHE 36 4.828 0.722 29.769
1.00155.06 H
ATOM 483 HE2 PHE 36 7.696 -0.656 26.884
1.00159.46 H
ATOM 484 HZ PHE 36 5.820 -1.121 28.437
1.00183.25 H
ATOM 485 N THR 37 10.561 5.541 28.007 1.00
20.06 N
ATOM 486 CA THR 37 11.244 6.787 27.709 1.00
27.18 C
ATOM 487 C THR 37 11.598 6.829 26.231 1.00
41.87 C
ATOM 488 O THR 37 10.987 6.146 25.409
1.00169.86 O
ATOM 489 CB THR 37 12.519 6.898 28.547 1.00
26.52 C
ATOM 490 CG2 THR 37 12.181 6.690 30.024 1.00
75.69 C
ATOM 491 OG1 THR 37 13.448 5.907 28.127 1.00
81.95 O
ATOM 492 HN THR 37 10.927 4.715 27.642 1.00
18.47 H
ATOM 493 HA THR 37 10.599 7.623 27.940 1.00
38.80 H
ATOM 494 HB THR 37 12.953 7.877 28.416 1.00
81.60 H

```
ATOM    495  HG1 THR    37       13.447   5.887  27.167
1.00188.80           H
ATOM    496 HG21 THR    37       11.503   7.464  30.350
1.00196.98           H
ATOM    497 HG22 THR    37       13.087   6.733  30.611
1.00196.23           H
ATOM    498 HG23 THR    37       11.714   5.725  30.154
1.00168.92           H
ATOM    499  N   GLU    38       12.587   7.640  25.908  1.00
26.53          N
ATOM    500  CA  GLU    38       13.043   7.793  24.526  1.00
30.29          C
ATOM    501  C   GLU    38       14.532   8.123  24.484  1.00
25.39          C
ATOM    502  O   GLU    38       15.083   8.673  25.439  1.00
51.60          O
ATOM    503  CB  GLU    38       12.266   8.914  23.826  1.00
54.82          C
ATOM    504  CG  GLU    38       10.799   8.510  23.651
1.00165.84           C
ATOM    505  CD  GLU    38       10.056   9.583  22.862
1.00292.92           C
ATOM    506  OE1 GLU    38       10.667  10.593  22.554
1.00414.19           O
ATOM    507  OE2 GLU    38        8.887   9.380  22.581
1.00537.65           O1-
ATOM    508  HN  GLU    38       13.023   8.142  26.618  1.00
84.50          H
ATOM    509  HA  GLU    38       12.877   6.869  23.991  1.00
32.15          H
ATOM    510  HB1 GLU    38       12.702   9.100  22.856
1.00144.82           H
ATOM    511  HB2 GLU    38       12.321   9.814  24.422
1.00106.49           H
ATOM    512  HG1 GLU    38       10.338   8.402  24.620
1.00303.31           H
ATOM    513  HG2 GLU    38       10.745   7.571  23.120
1.00323.99           H
ATOM    514  N   CYS    39       15.174   7.797  23.367  1.00
15.84          N
ATOM    515  CA  CYS    39       16.594   8.077  23.203  1.00
14.30          C
ATOM    516  C   CYS    39       16.821   9.593  23.172  1.00
22.31          C
ATOM    517  O   CYS    39       15.942  10.337  22.735  1.00
32.92          O
ATOM    518  CB  CYS    39       17.089   7.450  21.896  1.00
18.08          C
ATOM    519  SG  CYS    39       16.012   7.971  20.536  1.00
17.41          S
ATOM    520  HN  CYS    39       14.682   7.372  22.637  1.00
24.87          H
```

```
ATOM    521  HA  CYS    39      17.134   7.642  24.029  1.00
14.60         H
ATOM    522  HB1 CYS    39      17.068   6.375  21.980  1.00
27.71         H
ATOM    523  HB2 CYS    39      18.098   7.775  21.697  1.00
34.91         H
ATOM    524  N   PRO    40      17.957  10.072  23.623  1.00
27.27         N
ATOM    525  CA  PRO    40      18.249  11.534  23.633  1.00
47.66         C
ATOM    526  C   PRO    40      18.522  12.071  22.230  1.00
71.54         C
ATOM    527  O   PRO    40      19.312  11.503  21.475  1.00
87.70         O
ATOM    528  CB  PRO    40      19.488  11.647  24.531  1.00
53.85         C
ATOM    529  CG  PRO    40      20.185  10.335  24.376  1.00
46.04         C
ATOM    530  CD  PRO    40      19.082   9.291  24.166  1.00
25.70         C
ATOM    531  HA  PRO    40      17.429  12.073  24.081  1.00
58.04         H
ATOM    532  HB1 PRO    40      19.195  11.791  25.560  1.00
67.93         H
ATOM    533  HB2 PRO    40      20.124  12.461  24.204  1.00
70.99         H
ATOM    534  HG1 PRO    40      20.748  10.098  25.268  1.00
65.91         H
ATOM    535  HG2 PRO    40      20.845  10.364  23.517  1.00
54.99         H
ATOM    536  HD1 PRO    40      18.802   8.836  25.104  1.00
24.22         H
ATOM    537  HD2 PRO    40      19.402   8.543  23.458  1.00
26.94         H
ATOM    538  N   GLY    41      17.861  13.169  21.897
1.00105.24         N
ATOM    539  CA  GLY    41      18.029  13.791  20.589
1.00147.55         C
ATOM    540  C   GLY    41      19.457  14.282  20.396
1.00161.74         C
ATOM    541  O   GLY    41      20.029  14.157  19.313
1.00244.37         O
ATOM    542  HN  GLY    41      17.249  13.570  22.545
1.00121.79         H
ATOM    543  HA1 GLY    41      17.354  14.630  20.505
1.00184.40         H
ATOM    544  HA2 GLY    41      17.795  13.070  19.823
1.00157.34         H
ATOM    545  N   LEU    42      20.028  14.846  21.458
1.00162.48         N
ATOM    546  CA  LEU    42      21.396  15.367  21.415
1.00191.48         C
```

ATOM 547 C LEU 42 22.326 14.481 22.238
1.00166.66 C
ATOM 548 O LEU 42 21.966 14.024 23.323
1.00251.38 O
ATOM 549 CB LEU 42 21.427 16.790 21.980
1.00307.99 C
ATOM 550 CG LEU 42 20.437 17.678 21.217
1.00452.28 C
ATOM 551 CD1 LEU 42 20.436 19.078 21.839
1.00681.24 C
ATOM 552 CD2 LEU 42 20.844 17.773 19.736
1.00527.46 C
ATOM 553 HN LEU 42 19.515 14.916 22.289
1.00195.47 H
ATOM 554 HA LEU 42 21.750 15.387 20.395
1.00229.77 H
ATOM 555 HB1 LEU 42 22.422 17.195 21.876
1.00342.49 H
ATOM 556 HB2 LEU 42 21.156 16.767 23.025
1.00335.33 H
ATOM 557 HG LEU 42 19.445 17.254 21.293
1.00427.79 H
ATOM 558 HD11 LEU 42 21.451 19.442 21.907
1.00909.71 H
ATOM 559 HD12 LEU 42 20.003 19.033 22.827
1.00706.80 H
ATOM 560 HD13 LEU 42 19.854 19.747 21.221
1.00858.82 H
ATOM 561 HD21 LEU 42 21.921 17.806 19.655
1.00631.69 H
ATOM 562 HD22 LEU 42 20.425 18.667 19.298
1.00628.06 H
ATOM 563 HD23 LEU 42 20.470 16.909 19.204
1.00656.75 H
ATOM 564 N THR 43 23.526 14.245 21.716
1.00178.52 N
ATOM 565 CA THR 43 24.500 13.414 22.411
1.00240.84 C
ATOM 566 C THR 43 25.038 14.153 23.643
1.00372.31 C
ATOM 567 O THR 43 25.104 15.381 23.644
1.00500.13 O
ATOM 568 CB THR 43 25.656 13.084 21.461
1.00354.13 C
ATOM 569 CG2 THR 43 25.101 12.458 20.181
1.00463.04 C
ATOM 570 OG1 THR 43 26.356 14.278 21.140
1.00508.50 O
ATOM 571 HN THR 43 23.758 14.637 20.849
1.00229.59 H
ATOM 572 HA THR 43 24.022 12.498 22.714
1.00249.90 H

ATOM 573 HB THR 43 26.330 12.389 21.933
1.00481.58 H
ATOM 574 HG1 THR 43 26.658 14.210 20.231
1.00624.31 H
ATOM 575 HG21 THR 43 25.915 12.233 19.509
1.00650.95 H
ATOM 576 HG22 THR 43 24.422 13.151 19.706
1.00580.73 H
ATOM 577 HG23 THR 43 24.574 11.547 20.426
1.00571.99 H
ATOM 578 N PRO 44 25.430 13.448 24.685
1.00488.39 N
ATOM 579 CA PRO 44 25.973 14.093 25.916
1.00760.13 C
ATOM 580 C PRO 44 27.418 14.539 25.716
1.00735.36 C
ATOM 581 O PRO 44 27.973 15.275 26.532
1.00999.99 O
ATOM 582 CB PRO 44 25.869 12.984 26.966
1.00999.99 C
ATOM 583 CG PRO 44 26.045 11.720 26.190
1.00856.94 C
ATOM 584 CD PRO 44 25.410 11.975 24.817
1.00548.09 C
ATOM 585 HA PRO 44 25.357 14.930 26.205
1.00939.00 H
ATOM 586 HB1 PRO 44 24.895 13.000 27.434
1.00999.99 H
ATOM 587 HB2 PRO 44 26.648 13.090 27.712
1.00999.99 H
ATOM 588 HG1 PRO 44 25.540 10.902 26.682
1.00999.99 H
ATOM 589 HG2 PRO 44 27.101 11.497 26.083
1.00853.53 H
ATOM 590 HD1 PRO 44 24.394 11.611 24.797
1.00602.45 H
ATOM 591 HD2 PRO 44 26.001 11.513 24.040
1.00463.32 H
ATOM 592 N ILE 45 28.018 14.077 24.624
1.00518.33 N
ATOM 593 CA ILE 45 29.398 14.421 24.318
1.00582.79 C
ATOM 594 C ILE 45 29.490 15.840 23.765
1.00795.93 C
ATOM 595 O ILE 45 28.793 16.192 22.814
1.00898.75 O
ATOM 596 CB ILE 45 29.965 13.436 23.295
1.00474.41 C
ATOM 597 CG1 ILE 45 29.952 12.026 23.889
1.00516.90 C
ATOM 598 CG2 ILE 45 31.402 13.830 22.948
1.00760.75 C

```
ATOM    599  CD1 ILE    45       30.274  11.007  22.795
1.00566.81            C
ATOM    600  HN  ILE    45       27.523  13.490  24.015
1.00395.72            H
ATOM    601  HA  ILE    45       29.981  14.356  25.222
1.00711.06            H
ATOM    602  HB  ILE    45       29.360  13.459  22.400
1.00477.81            H
ATOM    603 HG11 ILE    45       28.974  11.816  24.295
1.00624.28            H
ATOM    604 HG12 ILE    45       30.692  11.959  24.673
1.00744.84            H
ATOM    605 HG21 ILE    45       31.959  13.998  23.859
1.00920.57            H
ATOM    606 HG22 ILE    45       31.396  14.734  22.358
1.00999.99            H
ATOM    607 HG23 ILE    45       31.868  13.035  22.384
1.00865.46            H
ATOM    608 HD11 ILE    45       30.120  10.008  23.175
1.00658.89            H
ATOM    609 HD12 ILE    45       31.303  11.121  22.489
1.00734.30            H
ATOM    610 HD13 ILE    45       29.626  11.171  21.947
1.00675.84            H
ATOM    611  N   ALA    46       30.353  16.650  24.369
1.00999.99            N
ATOM    612  CA  ALA    46       30.528  18.029  23.929
1.00999.99            C
ATOM    613  C   ALA    46       31.385  18.085  22.669
1.00999.99            C
ATOM    614  CB  ALA    46       31.189  18.851  25.036
1.00999.99            C
ATOM    615  OT1 ALA    46       30.843  18.386  21.618
1.00999.99            O
ATOM    616  OT2 ALA    46       32.573  17.826  22.773
1.00999.99            O
ATOM    617  HN  ALA    46       30.882  16.315  25.122
1.00999.99            H
ATOM    618  HA  ALA    46       29.559  18.453  23.711
1.00999.99            H
ATOM    619  HB1 ALA    46       32.060  18.328  25.401
1.00999.99            H
ATOM    620  HB2 ALA    46       30.489  18.994  25.846
1.00999.99            H
ATOM    621  HB3 ALA    46       31.486  19.813  24.643
1.00999.99            H
ENDMDL
TER
MODEL       20
ATOM      1  N   GLY     1       27.247  -1.647  14.623
1.00999.99            N
ATOM      2  CA  GLY     1       26.490  -1.262  15.848
```

```
1.00999.99           C
ATOM      3  C   GLY     1       25.306  -0.384  15.459
1.00999.99           C
ATOM      4  O   GLY     1       24.737   0.316  16.297
1.00999.99           O
ATOM      5  HA1 GLY     1       27.137  -0.712  16.514
1.00999.99           H
ATOM      6  HA2 GLY     1       26.133  -2.152  16.346
1.00999.99           H
ATOM      7  HT1 GLY     1       26.629  -2.194  13.991
1.00999.99           H
ATOM      8  HT2 GLY     1       28.068  -2.227  14.892
1.00999.99           H
ATOM      9  HT3 GLY     1       27.572  -0.792  14.132
1.00999.99           H
ATOM     10  N   LEU     2       24.938  -0.427  14.182
1.00999.99           N
ATOM     11  CA  LEU     2       23.818   0.369  13.691
1.00895.59           C
ATOM     12  C   LEU     2       22.496  -0.228  14.166
1.00532.27           C
ATOM     13  O   LEU     2       22.356  -1.447  14.261
1.00625.21           O
ATOM     14  CB  LEU     2       23.839   0.409  12.161
1.00999.99           C
ATOM     15  CG  LEU     2       25.175   0.982  11.671
1.00999.99           C
ATOM     16  CD1 LEU     2       25.204   0.950  10.139
1.00999.99           C
ATOM     17  CD2 LEU     2       25.343   2.433  12.161
1.00999.99           C
ATOM     18  HN  LEU     2       25.428  -1.004  13.559
1.00999.99           H
ATOM     19  HA  LEU     2       23.905   1.373  14.069
1.00890.87           H
ATOM     20  HB1 LEU     2       23.032   1.034  11.808
1.00924.07           H
ATOM     21  HB2 LEU     2       23.714  -0.592  11.774
1.00999.99           H
ATOM     22  HG  LEU     2       25.984   0.376  12.055
1.00999.99           H
ATOM     23 HD11 LEU     2       24.310   1.418   9.753
1.00999.99           H
ATOM     24 HD12 LEU     2       25.248  -0.075   9.802
1.00999.99           H
ATOM     25 HD13 LEU     2       26.073   1.485   9.785
1.00999.99           H
ATOM     26 HD21 LEU     2       24.384   2.931  12.165
1.00999.99           H
ATOM     27 HD22 LEU     2       26.019   2.964  11.507
1.00999.99           H
ATOM     28 HD23 LEU     2       25.750   2.429  13.161
```

```
1.00999.99           H
ATOM     29  N   CYS     3       21.527   0.635  14.463
1.00271.28           N
ATOM     30  CA  CYS     3       20.225   0.168  14.925
1.00104.50           C
ATOM     31  C   CYS     3       19.486  -0.535  13.791  1.00
90.98           C
ATOM     32  O   CYS     3       19.468  -0.054  12.659
1.00207.28           O
ATOM     33  CB  CYS     3       19.382   1.350  15.421  1.00
37.83           C
ATOM     34  SG  CYS     3       20.194   2.140  16.835  1.00
81.56           S
ATOM     35  HN  CYS     3       21.691   1.597  14.369
1.00296.55           H
ATOM     36  HA  CYS     3       20.368  -0.527  15.739
1.00166.45           H
ATOM     37  HB1 CYS     3       18.407   0.993  15.720  1.00
40.38           H
ATOM     38  HB2 CYS     3       19.271   2.070  14.624  1.00
98.45           H
ATOM     39  N   SER     4       18.877  -1.674  14.102  1.00
85.31           N
ATOM     40  CA  SER     4       18.138  -2.431  13.097  1.00
92.94           C
ATOM     41  C   SER     4       16.992  -1.595  12.556  1.00
67.37           C
ATOM     42  O   SER     4       16.724  -1.579  11.355
1.00102.28           O
ATOM     43  CB  SER     4       17.550  -3.693  13.718
1.00123.83           C
ATOM     44  OG  SER     4       16.941  -4.477  12.700
1.00178.89           O
ATOM     45  HN  SER     4       18.923  -2.010  15.022
1.00163.03           H
ATOM     46  HA  SER     4       18.799  -2.704  12.290
1.00129.78           H
ATOM     47  HB1 SER     4       16.803  -3.407  14.453
1.00105.26           H
ATOM     48  HB2 SER     4       18.327  -4.265  14.197
1.00156.87           H
ATOM     49  HG  SER     4       17.639  -4.859  12.163
1.00218.16           H
ATOM     50  N   GLU     5       16.304  -0.924  13.470  1.00
41.67           N
ATOM     51  CA  GLU     5       15.157  -0.100  13.117  1.00
41.21           C
ATOM     52  C   GLU     5       15.031   1.075  14.080  1.00
41.92           C
ATOM     53  O   GLU     5       15.980   1.411  14.789  1.00
74.72           O
ATOM     54  CB  GLU     5       13.891  -0.954  13.167  1.00
```

```
49.24           C
ATOM     55  CG  GLU     5       13.770  -1.600  14.544  1.00
55.48           C
ATOM     56  CD  GLU     5       12.689  -2.675  14.525  1.00
92.06           C
ATOM     57  OE1 GLU     5       12.860  -3.646  13.807
1.00206.49           O
ATOM     58  OE2 GLU     5       11.707  -2.511  15.228
1.00197.24           O1-
ATOM     59  HN  GLU     5       16.564  -1.000  14.412  1.00
45.52           H
ATOM     60  HA  GLU     5       15.282   0.274  12.120  1.00
60.63           H
ATOM     61  HB1 GLU     5       13.945  -1.724  12.414  1.00
61.59           H
ATOM     62  HB2 GLU     5       13.034  -0.334  12.986  1.00
63.66           H
ATOM     63  HG1 GLU     5       13.514  -0.847  15.273  1.00
58.03           H
ATOM     64  HG2 GLU     5       14.715  -2.044  14.805  1.00
57.77           H
ATOM     65  N   ASN     6       13.857   1.697  14.104  1.00
50.73           N
ATOM     66  CA  ASN     6       13.630   2.832  14.989  1.00
71.04           C
ATOM     67  C   ASN     6       13.829   2.413  16.442  1.00
65.17           C
ATOM     68  O   ASN     6       14.393   3.160  17.243  1.00
94.86           O
ATOM     69  CB  ASN     6       12.210   3.367  14.800  1.00
94.83           C
ATOM     70  CG  ASN     6       12.092   4.071  13.452
1.00160.94           C
ATOM     71  ND2 ASN     6       10.914   4.245  12.919
1.00244.88           N
ATOM     72  OD1 ASN     6       13.100   4.471  12.869
1.00219.73           O
ATOM     73  HN  ASN     6       13.134   1.388  13.519  1.00
72.81           H
ATOM     74  HA  ASN     6       14.334   3.614  14.749  1.00
96.02           H
ATOM     75  HB1 ASN     6       11.983   4.068  15.589
1.00111.09           H
ATOM     76  HB2 ASN     6       11.510   2.546  14.838  1.00
98.13           H
ATOM     77 HD21 ASN     6       10.113   3.926  13.386
1.00272.43           H
ATOM     78 HD22 ASN     6       10.829   4.696  12.053
1.00336.78           H
ATOM     79  N   GLY     7       13.368   1.207  16.773  1.00
51.80           N
ATOM     80  CA  GLY     7       13.499   0.675  18.132  1.00
```

```
71.47           C
ATOM     81  C   GLY     7       14.145  -0.704  18.106  1.00
41.04           C
ATOM     82  O   GLY     7       13.475  -1.720  18.283  1.00
44.54           O
ATOM     83  HN  GLY     7       12.932   0.660  16.087  1.00
45.49           H
ATOM     84  HA1 GLY     7       12.523   0.594  18.576
1.00103.52           H
ATOM     85  HA2 GLY     7       14.109   1.340  18.731
1.00108.67           H
ATOM     86  N   ASP     8       15.452  -0.727  17.880  1.00
27.00           N
ATOM     87  CA  ASP     8       16.188  -1.984  17.826  1.00
14.15           C
ATOM     88  C   ASP     8       16.097  -2.718  19.157  1.00
9.37           C
ATOM     89  O   ASP     8       15.893  -3.931  19.198  1.00
14.61           O
ATOM     90  CB  ASP     8       17.656  -1.719  17.490  1.00
15.06           C
ATOM     91  CG  ASP     8       18.399  -3.039  17.315  1.00
21.22           C
ATOM     92  OD1 ASP     8       19.534  -3.002  16.869
1.00119.56           O
ATOM     93  OD2 ASP     8       17.822  -4.067  17.630
1.00133.27           O1-
ATOM     94  HN  ASP     8       15.928   0.117  17.745  1.00
35.63           H
ATOM     95  HA  ASP     8       15.763  -2.606  17.057  1.00
20.92           H
ATOM     96  HB1 ASP     8       18.110  -1.159  18.292  1.00
29.39           H
ATOM     97  HB2 ASP     8       17.717  -1.148  16.574  1.00
44.14           H
ATOM     98  N   CYS     9       16.245  -1.972  20.244  1.00
5.93           N
ATOM     99  CA  CYS     9       16.175  -2.561  21.575  1.00
7.54           C
ATOM    100  C   CYS     9       14.801  -3.177  21.806  1.00
14.60           C
ATOM    101  O   CYS     9       14.678  -4.277  22.345  1.00
25.78           O
ATOM    102  CB  CYS     9       16.436  -1.496  22.628  1.00
6.63           C
ATOM    103  SG  CYS     9       18.172  -1.025  22.547  1.00
10.91           S
ATOM    104  HN  CYS     9       16.403  -1.011  20.148  1.00
6.51           H
ATOM    105  HA  CYS     9       16.934  -3.320  21.664  1.00
11.12           H
ATOM    106  HB1 CYS     9       16.218  -1.892  23.607  1.00
```

```
11.28           H
ATOM    107  HB2 CYS     9      15.814  -0.634  22.437  1.00
5.04           H
ATOM    108  N   ALA    10      13.775  -2.452  21.380  1.00
16.26           N
ATOM    109  CA  ALA    10      12.398  -2.910  21.522  1.00
32.04           C
ATOM    110  C   ALA    10      11.469  -2.029  20.688  1.00
45.47           C
ATOM    111  O   ALA    10      11.870  -0.967  20.215
1.00119.04           O
ATOM    112  CB  ALA    10      11.976  -2.875  22.996  1.00
30.10           C
ATOM    113  HN  ALA    10      13.949  -1.588  20.955  1.00
12.81           H
ATOM    114  HA  ALA    10      12.331  -3.926  21.163  1.00
47.14           H
ATOM    115  HB1 ALA    10      12.814  -3.156  23.617  1.00
84.37           H
ATOM    116  HB2 ALA    10      11.164  -3.569  23.155
1.00118.20           H
ATOM    117  HB3 ALA    10      11.652  -1.879  23.260
1.00117.32           H
ATOM    118  N   ALA    11      10.233  -2.477  20.506  1.00
30.62           N
ATOM    119  CA  ALA    11       9.268  -1.717  19.720  1.00
35.24           C
ATOM    120  C   ALA    11       9.015  -0.343  20.338  1.00
22.78           C
ATOM    121  O   ALA    11       8.915   0.657  19.627  1.00
53.33           O
ATOM    122  CB  ALA    11       7.950  -2.487  19.632  1.00
60.49           C
ATOM    123  HN  ALA    11       9.966  -3.333  20.903  1.00
50.37           H
ATOM    124  HA  ALA    11       9.658  -1.584  18.723  1.00
41.70           H
ATOM    125  HB1 ALA    11       7.199  -1.865  19.168
1.00157.06           H
ATOM    126  HB2 ALA    11       7.625  -2.761  20.625
1.00148.83           H
ATOM    127  HB3 ALA    11       8.092  -3.380  19.042
1.00137.06           H
ATOM    128  N   ASP    12       8.904  -0.300  21.664  1.00
18.98           N
ATOM    129  CA  ASP    12       8.651   0.960  22.367  1.00
31.05           C
ATOM    130  C   ASP    12       9.952   1.711  22.644  1.00
22.98           C
ATOM    131  O   ASP    12       9.967   2.940  22.706  1.00
37.14           O
ATOM    132  CB  ASP    12       7.936   0.678  23.690  1.00
```

```
48.35           C
ATOM    133  CG  ASP    12       6.520   0.182  23.420
1.00104.83           C
ATOM    134  OD1 ASP    12       6.062   0.333  22.300
1.00258.73           O
ATOM    135  OD2 ASP    12       5.912  -0.343  24.339
1.00213.46           O1-
ATOM    136  HN  ASP    12       8.986  -1.129  22.181  1.00
36.19           H
ATOM    137  HA  ASP    12       8.013   1.581  21.756  1.00
49.76           H
ATOM    138  HB1 ASP    12       7.892   1.585  24.273  1.00
95.71           H
ATOM    139  HB2 ASP    12       8.482  -0.076  24.238  1.00
54.37           H
ATOM    140  N   GLU    13      11.038   0.966  22.821  1.00
17.60           N
ATOM    141  CA  GLU    13      12.337   1.572  23.104  1.00
12.01           C
ATOM    142  C   GLU    13      12.940   2.188  21.842  1.00
10.07           C
ATOM    143  O   GLU    13      12.633   1.768  20.726  1.00
12.81           O
ATOM    144  CB  GLU    13      13.279   0.514  23.673  1.00
11.74           C
ATOM    145  CG  GLU    13      12.718   0.014  25.007  1.00
13.49           C
ATOM    146  CD  GLU    13      13.460  -1.238  25.455
1.00142.36           C
ATOM    147  OE1 GLU    13      14.658  -1.299  25.243
1.00339.06           O
ATOM    148  OE2 GLU    13      12.820  -2.118  26.006
1.00335.28           O1-
ATOM    149  HN  GLU    13      10.965  -0.010  22.767  1.00
28.36           H
ATOM    150  HA  GLU    13      12.203   2.349  23.842  1.00
13.06           H
ATOM    151  HB1 GLU    13      14.254   0.947  23.835  1.00
13.76           H
ATOM    152  HB2 GLU    13      13.359  -0.310  22.979  1.00
9.94           H
ATOM    153  HG1 GLU    13      11.668  -0.213  24.893  1.00
66.02           H
ATOM    154  HG2 GLU    13      12.836   0.786  25.754  1.00
56.23           H
ATOM    155  N   CYS    14      13.802   3.193  22.029  1.00
9.12           N
ATOM    156  CA  CYS    14      14.450   3.875  20.903  1.00
10.69           C
ATOM    157  C   CYS    14      15.904   3.435  20.770  1.00
9.56           C
ATOM    158  O   CYS    14      16.556   3.114  21.760  1.00
```

13.62 O
ATOM 159 CB CYS 14 14.401 5.392 21.110 1.00
13.96 C
ATOM 160 SG CYS 14 15.330 6.207 19.784 1.00
42.96 S
ATOM 161 HN CYS 14 14.007 3.484 22.944 1.00
9.76 H
ATOM 162 HA CYS 14 13.928 3.635 19.987 1.00
15.54 H
ATOM 163 HB1 CYS 14 14.840 5.640 22.065 1.00
50.72 H
ATOM 164 HB2 CYS 14 13.375 5.725 21.089 1.00
46.13 H
ATOM 165 N CYS 15 16.405 3.428 19.534 1.00
9.60 N
ATOM 166 CA CYS 15 17.789 3.035 19.265 1.00
9.28 C
ATOM 167 C CYS 15 18.413 4.011 18.278 1.00
9.92 C
ATOM 168 O CYS 15 17.886 4.218 17.186 1.00
13.31 O
ATOM 169 CB CYS 15 17.827 1.623 18.671 1.00
12.90 C
ATOM 170 SG CYS 15 19.549 1.101 18.445 1.00
39.11 S
ATOM 171 HN CYS 15 15.835 3.704 18.786 1.00
13.12 H
ATOM 172 HA CYS 15 18.360 3.048 20.184 1.00
8.26 H
ATOM 173 HB1 CYS 15 17.323 1.622 17.716 1.00
24.82 H
ATOM 174 HB2 CYS 15 17.329 0.939 19.342 1.00
27.31 H
ATOM 175 N VAL 16 19.533 4.616 18.664 1.00
9.56 N
ATOM 176 CA VAL 16 20.209 5.573 17.793 1.00
11.99 C
ATOM 177 C VAL 16 21.718 5.441 17.938 1.00
7.73 C
ATOM 178 O VAL 16 22.252 5.472 19.047 1.00
8.83 O
ATOM 179 CB VAL 16 19.765 6.995 18.150 1.00
18.37 C
ATOM 180 CG1 VAL 16 20.355 7.407 19.502 1.00
39.61 C
ATOM 181 CG2 VAL 16 20.240 7.961 17.065
1.00115.21 C
ATOM 182 HN VAL 16 19.914 4.417 19.548 1.00
9.76 H
ATOM 183 HA VAL 16 19.942 5.374 16.763 1.00
17.27 H
ATOM 184 HB VAL 16 18.687 7.026 18.209 1.00

```
52.63           H
ATOM    185 HG11 VAL    16      21.402   7.644  19.381
1.00128.07           H
ATOM    186 HG12 VAL    16      20.248   6.592  20.203
1.00154.13           H
ATOM    187 HG13 VAL    16      19.830   8.273  19.874
1.00135.74           H
ATOM    188 HG21 VAL    16      19.650   7.815  16.173
1.00229.05           H
ATOM    189 HG22 VAL    16      21.281   7.772  16.844
1.00261.82           H
ATOM    190 HG23 VAL    16      20.124   8.976  17.411
1.00210.07           H
ATOM    191  N   ASP    17      22.403   5.283  16.806  1.00
14.34           N
ATOM    192  CA  ASP    17      23.858   5.134  16.805  1.00
11.90           C
ATOM    193  C   ASP    17      24.533   6.369  16.225  1.00
13.15           C
ATOM    194  O   ASP    17      24.330   6.714  15.060  1.00
25.96           O
ATOM    195  CB  ASP    17      24.246   3.905  15.987  1.00
20.80           C
ATOM    196  CG  ASP    17      23.608   2.664  16.600  1.00
28.72           C
ATOM    197  OD1 ASP    17      22.747   2.086  15.961
1.00135.42           O
ATOM    198  OD2 ASP    17      23.993   2.308  17.702
1.00114.17           O1-
ATOM    199  HN  ASP    17      21.919   5.260  15.954  1.00
28.29           H
ATOM    200  HA  ASP    17      24.209   4.993  17.817  1.00
10.49           H
ATOM    201  HB1 ASP    17      25.320   3.794  15.990  1.00
22.42           H
ATOM    202  HB2 ASP    17      23.900   4.026  14.970  1.00
29.52           H
ATOM    203  N   THR    18      25.350   7.022  17.046  1.00
11.55           N
ATOM    204  CA  THR    18      26.080   8.216  16.622  1.00
17.10           C
ATOM    205  C   THR    18      27.540   7.870  16.380  1.00
10.63           C
ATOM    206  O   THR    18      28.004   6.802  16.775  1.00
6.49           O
ATOM    207  CB  THR    18      25.991   9.302  17.695  1.00
28.52           C
ATOM    208  CG2 THR    18      24.543   9.767  17.841  1.00
45.38           C
ATOM    209  OG1 THR    18      26.455   8.785  18.933  1.00
26.49           O
ATOM    210  HN  THR    18      25.476   6.686  17.958  1.00
```

```
13.96           H
ATOM    211  HA  THR    18       25.653   8.597  15.703  1.00
26.43           H
ATOM    212  HB  THR    18       26.604  10.143  17.407  1.00
37.43           H
ATOM    213  HG1 THR    18       25.734   8.299  19.341  1.00
71.62           H
ATOM    214 HG21 THR    18       24.436  10.322  18.761
1.00100.44           H
ATOM    215 HG22 THR    18       23.889   8.909  17.858
1.00129.42           H
ATOM    216 HG23 THR    18       24.285  10.401  17.006
1.00156.21           H
ATOM    217  N   VAL    19       28.260   8.788  15.750  1.00
16.32           N
ATOM    218  CA  VAL    19       29.662   8.596  15.471  1.00
13.34           C
ATOM    219  C   VAL    19       30.195   9.804  14.733  1.00
25.88           C
ATOM    220  O   VAL    19       29.766  10.133  13.627  1.00
42.11           O
ATOM    221  CB  VAL    19       29.916   7.332  14.648  1.00
15.48           C
ATOM    222  CG1 VAL    19       29.011   7.314  13.413  1.00
29.06           C
ATOM    223  CG2 VAL    19       31.388   7.314  14.210  1.00
20.67           C
ATOM    224  HN  VAL    19       27.845   9.627  15.480  1.00
26.89           H
ATOM    225  HA  VAL    19       30.188   8.506  16.410  1.00
8.08           H
ATOM    226  HB  VAL    19       29.713   6.468  15.259  1.00
11.49           H
ATOM    227 HG11 VAL    19       28.006   7.591  13.694
1.00137.43           H
ATOM    228 HG12 VAL    19       29.003   6.322  12.988  1.00
93.29           H
ATOM    229 HG13 VAL    19       29.387   8.014  12.681
1.00108.69           H
ATOM    230 HG21 VAL    19       31.527   8.012  13.396  1.00
99.42           H
ATOM    231 HG22 VAL    19       31.658   6.322  13.888
1.00111.36           H
ATOM    232 HG23 VAL    19       32.015   7.611  15.043  1.00
77.11           H
ATOM    233  N   PHE    20       31.123  10.458  15.381  1.00
24.58           N
ATOM    234  CA  PHE    20       31.754  11.654  14.854  1.00
40.34           C
ATOM    235  C   PHE    20       33.116  11.330  14.262  1.00
40.37           C
ATOM    236  O   PHE    20       33.310  11.384  13.049  1.00
```

```
66.02           O
ATOM    237  CB  PHE    20       31.906  12.683  15.988  1.00
48.79           C
ATOM    238  CG  PHE    20       32.126  11.987  17.323  1.00
35.75           C
ATOM    239  CD1 PHE    20       31.096  11.228  17.918  1.00
30.20           C
ATOM    240  CD2 PHE    20       33.355  12.117  17.983  1.00
40.95           C
ATOM    241  CE1 PHE    20       31.306  10.609  19.152  1.00
30.41           C
ATOM    242  CE2 PHE    20       33.561  11.493  19.219  1.00
45.84           C
ATOM    243  CZ  PHE    20       32.537  10.740  19.802  1.00
40.81           C
ATOM    244  HN  PHE    20       31.383  10.129  16.258  1.00
16.73           H
ATOM    245  HA  PHE    20       31.130  12.080  14.077  1.00
59.91           H
ATOM    246  HB1 PHE    20       31.020  13.266  16.044  1.00
67.15           H
ATOM    247  HB2 PHE    20       32.743  13.337  15.781  1.00
58.17           H
ATOM    248  HD1 PHE    20       30.141  11.113  17.421  1.00
33.34           H
ATOM    249  HD2 PHE    20       34.147  12.697  17.536  1.00
49.35           H
ATOM    250  HE1 PHE    20       30.517  10.029  19.602  1.00
30.61           H
ATOM    251  HE2 PHE    20       34.511  11.593  19.722  1.00
61.27           H
ATOM    252  HZ  PHE    20       32.696  10.260  20.757  1.00
52.66           H
ATOM    253  N   GLU    21       34.063  11.026  15.137  1.00
41.31           N
ATOM    254  CA  GLU    21       35.416  10.731  14.724  1.00
56.16           C
ATOM    255  C   GLU    21       35.649   9.236  14.582  1.00
58.61           C
ATOM    256  O   GLU    21       34.738   8.427  14.759
1.00201.19           O
ATOM    257  CB  GLU    21       36.346  11.313  15.769  1.00
57.37           C
ATOM    258  CG  GLU    21       36.204  10.534  17.078
1.00193.56           C
ATOM    259  CD  GLU    21       36.891  11.291  18.210
1.00304.62           C
ATOM    260  OE1 GLU    21       36.734  10.883  19.348
1.00451.81           O
ATOM    261  OE2 GLU    21       37.564  12.266  17.921
1.00442.88           O1-
ATOM    262  HN  GLU    21       33.856  11.026  16.093  1.00
```

```
50.82           H
ATOM    263  HA  GLU    21      35.622  11.212  13.778  1.00
85.72           H
ATOM    264  HB1 GLU    21      36.068  12.339  15.936  1.00
46.37           H
ATOM    265  HB2 GLU    21      37.360  11.261  15.421
1.00131.81           H
ATOM    266  HG1 GLU    21      36.659   9.566  16.967
1.00314.91           H
ATOM    267  HG2 GLU    21      35.159  10.409  17.309
1.00266.92           H
ATOM    268  N   GLY    22      36.885   8.888  14.254  1.00
86.70           N
ATOM    269  CA  GLY    22      37.270   7.485  14.074
1.00104.66           C
ATOM    270  C   GLY    22      37.872   6.913  15.352  1.00
71.50           C
ATOM    271  O   GLY    22      38.085   5.706  15.465  1.00
89.87           O
ATOM    272  HN  GLY    22      37.555   9.595  14.132
1.00214.64           H
ATOM    273  HA1 GLY    22      38.002   7.420  13.283
1.00152.47           H
ATOM    274  HA2 GLY    22      36.401   6.904  13.798
1.00118.15           H
ATOM    275  N   ASP    23      38.147   7.790  16.310  1.00
44.68           N
ATOM    276  CA  ASP    23      38.732   7.370  17.578  1.00
43.33           C
ATOM    277  C   ASP    23      37.819   6.387  18.301  1.00
37.55           C
ATOM    278  O   ASP    23      38.277   5.381  18.841  1.00
61.37           O
ATOM    279  CB  ASP    23      38.968   8.590  18.468  1.00
40.74           C
ATOM    280  CG  ASP    23      39.750   8.184  19.713
1.00141.86           C
ATOM    281  OD1 ASP    23      39.949   6.996  19.902
1.00328.18           O
ATOM    282  OD2 ASP    23      40.137   9.068  20.460
1.00304.58           O1-
ATOM    283  HN  ASP    23      37.962   8.737  16.157  1.00
40.86           H
ATOM    284  HA  ASP    23      39.677   6.892  17.386  1.00
72.12           H
ATOM    285  HB1 ASP    23      38.015   9.002  18.766  1.00
94.15           H
ATOM    286  HB2 ASP    23      39.525   9.335  17.919  1.00
93.26           H
ATOM    287  N   MET    24      36.527   6.690  18.313  1.00
23.24           N
ATOM    288  CA  MET    24      35.554   5.828  18.982  1.00
```

32.50 C
ATOM 289 C MET 24 34.145 6.080 18.451 1.00
25.13 C
ATOM 290 O MET 24 33.907 7.042 17.723 1.00
54.40 O
ATOM 291 CB MET 24 35.592 6.075 20.492 1.00
47.36 C
ATOM 292 CG MET 24 35.239 7.534 20.783
1.00151.73 C
ATOM 293 SD MET 24 35.402 7.850 22.558
1.00209.23 S
ATOM 294 CE MET 24 34.926 9.596 22.529
1.00243.29 C
ATOM 295 HN MET 24 36.224 7.507 17.869 1.00
17.32 H
ATOM 296 HA MET 24 35.816 4.797 18.794 1.00
48.94 H
ATOM 297 HB1 MET 24 36.582 5.865 20.866
1.00124.40 H
ATOM 298 HB2 MET 24 34.878 5.427 20.979
1.00166.96 H
ATOM 299 HG1 MET 24 34.222 7.728 20.474
1.00331.71 H
ATOM 300 HG2 MET 24 35.910 8.183 20.239
1.00302.30 H
ATOM 301 HE1 MET 24 35.463 10.127 23.303
1.00340.44 H
ATOM 302 HE2 MET 24 33.867 9.684 22.706
1.00373.88 H
ATOM 303 HE3 MET 24 35.165 10.019 21.563
1.00386.81 H
ATOM 304 N VAL 25 33.215 5.197 18.822 1.00
22.44 N
ATOM 305 CA VAL 25 31.818 5.306 18.384 1.00
15.37 C
ATOM 306 C VAL 25 30.886 5.472 19.579 1.00
17.47 C
ATOM 307 O VAL 25 31.134 4.938 20.660 1.00
29.52 O
ATOM 308 CB VAL 25 31.420 4.056 17.591 1.00
23.36 C
ATOM 309 CG1 VAL 25 29.910 4.079 17.296 1.00
58.39 C
ATOM 310 CG2 VAL 25 32.205 4.027 16.274 1.00
55.25 C
ATOM 311 HN VAL 25 33.474 4.449 19.401 1.00
48.11 H
ATOM 312 HA VAL 25 31.708 6.175 17.740 1.00
9.25 H
ATOM 313 HB VAL 25 31.659 3.175 18.171 1.00
52.45 H
ATOM 314 HG11 VAL 25 29.592 5.094 17.106

1.00171.07 H
ATOM 315 HG12 VAL 25 29.374 3.693 18.150
1.00166.22 H
ATOM 316 HG13 VAL 25 29.696 3.465 16.432
1.00134.76 H
ATOM 317 HG21 VAL 25 31.836 3.224 15.653
1.00141.88 H
ATOM 318 HG22 VAL 25 33.252 3.869 16.483
1.00184.50 H
ATOM 319 HG23 VAL 25 32.078 4.968 15.759
1.00133.90 H
ATOM 320 N THR 26 29.816 6.238 19.370 1.00
13.60 N
ATOM 321 CA THR 26 28.833 6.513 20.418 1.00
21.19 C
ATOM 322 C THR 26 27.434 6.061 19.998 1.00
16.55 C
ATOM 323 O THR 26 27.020 6.269 18.858 1.00
11.43 O
ATOM 324 CB THR 26 28.821 8.014 20.695 1.00
27.07 C
ATOM 325 CG2 THR 26 27.716 8.355 21.697 1.00
44.39 C
ATOM 326 OG1 THR 26 30.082 8.401 21.221 1.00
34.75 O
ATOM 327 HN THR 26 29.689 6.641 18.486 1.00
10.81 H
ATOM 328 HA THR 26 29.110 5.995 21.327 1.00
33.16 H
ATOM 329 HB THR 26 28.641 8.539 19.766 1.00
20.60 H
ATOM 330 HG1 THR 26 30.508 7.618 21.578 1.00
77.72 H
ATOM 331 HG21 THR 26 27.854 9.365 22.053
1.00126.99 H
ATOM 332 HG22 THR 26 27.763 7.670 22.530
1.00108.32 H
ATOM 333 HG23 THR 26 26.753 8.270 21.215
1.00151.99 H
ATOM 334 N ARG 27 26.706 5.449 20.933 1.00
22.38 N
ATOM 335 CA ARG 27 25.347 4.978 20.661 1.00
20.21 C
ATOM 336 C ARG 27 24.507 5.004 21.936 1.00
16.96 C
ATOM 337 O ARG 27 25.046 4.996 23.041 1.00
20.06 O
ATOM 338 CB ARG 27 25.392 3.555 20.096 1.00
22.14 C
ATOM 339 CG ARG 27 25.966 2.600 21.146
1.00124.30 C
ATOM 340 CD ARG 27 26.165 1.218 20.523

```
1.00109.38            C
ATOM    341  NE  ARG    27      26.701   0.291  21.514
1.00227.73            N
ATOM    342  CZ  ARG    27      27.040  -0.950  21.179
1.00426.12            C
ATOM    343  NH1 ARG    27      27.519  -1.760  22.083
1.00767.09            N1+
ATOM    344  NH2 ARG    27      26.896  -1.356  19.949
1.00581.78            N
ATOM    345  HN  ARG    27      27.088   5.318  21.826  1.00
30.75           H
ATOM    346  HA  ARG    27      24.888   5.629  19.933  1.00
23.13           H
ATOM    347  HB1 ARG    27      26.018   3.537  19.216  1.00
87.46           H
ATOM    348  HB2 ARG    27      24.392   3.242  19.833
1.00103.36            H
ATOM    349  HG1 ARG    27      25.281   2.522  21.976
1.00281.97            H
ATOM    350  HG2 ARG    27      26.915   2.978  21.496
1.00276.19            H
ATOM    351  HD1 ARG    27      26.852   1.296  19.693
1.00183.60            H
ATOM    352  HD2 ARG    27      25.216   0.847  20.166
1.00142.93            H
ATOM    353  HE  ARG    27      26.813   0.589  22.440
1.00372.53            H
ATOM    354 HH11 ARG    27      27.629  -1.448  23.027
1.00910.59            H
ATOM    355 HH12 ARG    27      27.774  -2.694  21.833
1.00999.99            H
ATOM    356 HH21 ARG    27      26.530  -0.735  19.256
1.00532.54            H
ATOM    357 HH22 ARG    27      27.152  -2.290  19.698
1.00948.84            H
ATOM    358  N   SER    28      23.180   5.044  21.777  1.00
14.60           N
ATOM    359  CA  SER    28      22.277   5.080  22.932  1.00
14.92           C
ATOM    360  C   SER    28      21.025   4.246  22.676  1.00
12.12           C
ATOM    361  O   SER    28      20.610   4.059  21.532  1.00
13.41           O
ATOM    362  CB  SER    28      21.874   6.525  23.228  1.00
23.26           C
ATOM    363  OG  SER    28      23.037   7.282  23.538
1.00146.32            O
ATOM    364  HN  SER    28      22.799   5.053  20.871  1.00
15.23           H
ATOM    365  HA  SER    28      22.787   4.680  23.797  1.00
15.44           H
ATOM    366  HB1 SER    28      21.187   6.542  24.064  1.00
```

88.73 H
ATOM 367 HB2 SER 28 21.393 6.952 22.364
1.00124.68 H
ATOM 368 HG SER 28 22.931 7.638 24.423
1.00242.47 H
ATOM 369 N CYS 29 20.423 3.759 23.757 1.00
10.48 N
ATOM 370 CA CYS 29 19.210 2.955 23.657 1.00
9.97 C
ATOM 371 C CYS 29 18.574 2.811 25.040 1.00
11.60 C
ATOM 372 O CYS 29 19.206 2.314 25.972 1.00
15.64 O
ATOM 373 CB CYS 29 19.537 1.572 23.068 1.00
9.52 C
ATOM 374 SG CYS 29 18.113 0.948 22.160 1.00
10.40 S
ATOM 375 HN CYS 29 20.796 3.953 24.641 1.00
11.10 H
ATOM 376 HA CYS 29 18.513 3.459 23.005 1.00
11.08 H
ATOM 377 HB1 CYS 29 19.778 0.874 23.860 1.00
9.33 H
ATOM 378 HB2 CYS 29 20.375 1.651 22.397 1.00
10.78 H
ATOM 379 N GLU 30 17.329 3.261 25.173 1.00
13.52 N
ATOM 380 CA GLU 30 16.631 3.188 26.456 1.00
17.41 C
ATOM 381 C GLU 30 15.989 1.822 26.662 1.00
10.81 C
ATOM 382 O GLU 30 15.677 1.117 25.703 1.00
23.22 O
ATOM 383 CB GLU 30 15.552 4.270 26.528 1.00
37.83 C
ATOM 384 CG GLU 30 16.202 5.647 26.410
1.00108.13 C
ATOM 385 CD GLU 30 17.066 5.925 27.636
1.00240.52 C
ATOM 386 OE1 GLU 30 16.858 5.268 28.643
1.00422.56 O
ATOM 387 OE2 GLU 30 17.922 6.790 27.550
1.00410.81 O1-
ATOM 388 HN GLU 30 16.873 3.657 24.398 1.00
15.61 H
ATOM 389 HA GLU 30 17.341 3.360 27.252 1.00
24.60 H
ATOM 390 HB1 GLU 30 15.033 4.198 27.472 1.00
72.67 H
ATOM 391 HB2 GLU 30 14.849 4.131 25.719 1.00
56.47 H
ATOM 392 HG1 GLU 30 15.433 6.401 26.338

```
1.00196.66          H
ATOM    393  HG2 GLU    30      16.818   5.677  25.524
1.00140.67          H
ATOM    394  N   LYS    31      15.790   1.461  27.931  1.00
11.96          N
ATOM    395  CA  LYS    31      15.175   0.181  28.288  1.00
11.40          C
ATOM    396  C   LYS    31      13.865   0.419  29.029  1.00
10.99          C
ATOM    397  O   LYS    31      13.747   1.365  29.809  1.00
12.86          O
ATOM    398  CB  LYS    31      16.122  -0.623  29.179  1.00
21.40          C
ATOM    399  CG  LYS    31      17.395  -0.960  28.400  1.00
57.81          C
ATOM    400  CD  LYS    31      18.341  -1.766  29.292
1.00115.52          C
ATOM    401  CE  LYS    31      19.615  -2.099  28.514
1.00250.23          C
ATOM    402  NZ  LYS    31      20.541  -2.873  29.386
1.00462.10          N1+
ATOM    403  HN  LYS    31      16.057   2.075  28.647  1.00
26.13          H
ATOM    404  HA  LYS    31      14.973  -0.390  27.392  1.00
13.87          H
ATOM    405  HB1 LYS    31      15.638  -1.538  29.488  1.00
37.63          H
ATOM    406  HB2 LYS    31      16.376  -0.039  30.052  1.00
47.19          H
ATOM    407  HG1 LYS    31      17.882  -0.047  28.094
1.00126.32          H
ATOM    408  HG2 LYS    31      17.137  -1.544  27.528
1.00114.19          H
ATOM    409  HD1 LYS    31      17.858  -2.681  29.597
1.00198.45          H
ATOM    410  HD2 LYS    31      18.595  -1.183  30.166
1.00200.14          H
ATOM    411  HE1 LYS    31      20.095  -1.184  28.200
1.00374.64          H
ATOM    412  HE2 LYS    31      19.361  -2.688  27.644
1.00403.27          H
ATOM    413  HZ1 LYS    31      20.328  -3.888  29.304
1.00627.38          H
ATOM    414  HZ2 LYS    31      20.420  -2.571  30.374
1.00622.26          H
ATOM    415  HZ3 LYS    31      21.523  -2.703  29.088
1.00619.20          H
ATOM    416  N   THR    32      12.881  -0.438  28.779  1.00
15.51          N
ATOM    417  CA  THR    32      11.584  -0.301  29.430  1.00
22.44          C
ATOM    418  C   THR    32      11.638  -0.833  30.858  1.00
```

```
26.60           C
ATOM    419  O   THR    32       12.000  -1.986  31.092  1.00
57.43           O
ATOM    420  CB  THR    32       10.519  -1.065  28.635  1.00
56.28           C
ATOM    421  CG2 THR    32        9.161  -0.929  29.329
1.00102.70           C
ATOM    422  OG1 THR    32       10.438  -0.528  27.322
1.00111.17           O
ATOM    423  HN  THR    32       13.031  -1.170  28.146  1.00
18.79           H
ATOM    424  HA  THR    32       11.313   0.743  29.456  1.00
19.67           H
ATOM    425  HB  THR    32       10.788  -2.107  28.581  1.00
84.25           H
ATOM    426  HG1 THR    32        9.933   0.287  27.365
1.00205.03           H
ATOM    427 HG21 THR    32        9.183  -1.452  30.273
1.00220.71           H
ATOM    428 HG22 THR    32        8.394  -1.357  28.701
1.00174.67           H
ATOM    429 HG23 THR    32        8.947   0.115  29.500
1.00217.44           H
ATOM    430  N   THR    33       11.266   0.018  31.810  1.00
22.47           N
ATOM    431  CA  THR    33       11.265  -0.365  33.220  1.00
41.60           C
ATOM    432  C   THR    33       10.247   0.438  33.984  1.00
38.03           C
ATOM    433  O   THR    33       10.548   1.495  34.540  1.00
52.73           O
ATOM    434  CB  THR    33       12.652  -0.166  33.836  1.00
63.25           C
ATOM    435  CG2 THR    33       13.633  -1.174  33.234
1.00121.65           C
ATOM    436  OG1 THR    33       13.101   1.155  33.570  1.00
96.20           O
ATOM    437  HN  THR    33       10.982   0.922  31.560  1.00
23.82           H
ATOM    438  HA  THR    33       10.982  -1.396  33.313  1.00
67.12           H
ATOM    439  HB  THR    33       12.594  -0.319  34.903
1.00107.59           H
ATOM    440  HG1 THR    33       13.670   1.124  32.797
1.00176.27           H
ATOM    441 HG21 THR    33       13.865  -0.888  32.219
1.00255.23           H
ATOM    442 HG22 THR    33       13.190  -2.158  33.240
1.00245.37           H
ATOM    443 HG23 THR    33       14.541  -1.184  33.821
1.00185.78           H
ATOM    444  N   GLY    34        9.037  -0.090  34.022  1.00
```

40.93 N
ATOM 445 CA GLY 34 7.973 0.564 34.740 1.00
45.59 C
ATOM 446 C GLY 34 7.494 1.809 34.003 1.00
40.97 C
ATOM 447 O GLY 34 6.298 2.096 33.962 1.00
82.17 O
ATOM 448 HN GLY 34 8.865 -0.944 33.572 1.00
54.00 H
ATOM 449 HA1 GLY 34 8.353 0.841 35.703 1.00
54.41 H
ATOM 450 HA2 GLY 34 7.148 -0.125 34.863 1.00
62.09 H
ATOM 451 N ASN 35 8.439 2.545 33.416 1.00
32.51 N
ATOM 452 CA ASN 35 8.115 3.764 32.670 1.00
33.20 C
ATOM 453 C ASN 35 8.891 3.805 31.357 1.00
23.17 C
ATOM 454 O ASN 35 10.057 3.415 31.300 1.00
27.01 O
ATOM 455 CB ASN 35 8.467 4.997 33.504 1.00
54.51 C
ATOM 456 CG ASN 35 7.670 4.994 34.805 1.00
80.73 C
ATOM 457 ND2 ASN 35 8.265 5.309 35.924
1.00220.46 N
ATOM 458 OD1 ASN 35 6.476 4.698 34.803
1.00117.80 O
ATOM 459 HN ASN 35 9.375 2.261 33.483 1.00
51.15 H
ATOM 460 HA ASN 35 7.057 3.783 32.449 1.00
47.21 H
ATOM 461 HB1 ASN 35 8.228 5.887 32.942 1.00
68.31 H
ATOM 462 HB2 ASN 35 9.524 4.987 33.731 1.00
57.75 H
ATOM 463 HD21 ASN 35 9.216 5.545 35.923
1.00405.39 H
ATOM 464 HD22 ASN 35 7.760 5.310 36.764
1.00245.64 H
ATOM 465 N PHE 36 8.233 4.279 30.302 1.00
35.72 N
ATOM 466 CA PHE 36 8.853 4.370 28.989 1.00
33.41 C
ATOM 467 C PHE 36 9.538 5.722 28.812 1.00
28.51 C
ATOM 468 O PHE 36 9.066 6.737 29.325 1.00
45.23 O
ATOM 469 CB PHE 36 7.774 4.208 27.924 1.00
67.53 C
ATOM 470 CG PHE 36 7.172 2.827 28.015 1.00

```
86.71            C
ATOM    471  CD1 PHE    36        6.068   2.602  28.846
1.00106.51            C
ATOM    472  CD2 PHE    36        7.712   1.772  27.268  1.00
99.48            C
ATOM    473  CE1 PHE    36        5.502   1.324  28.929
1.00134.48            C
ATOM    474  CE2 PHE    36        7.147   0.494  27.352
1.00134.27            C
ATOM    475  CZ  PHE    36        6.041   0.271  28.182
1.00149.48            C
ATOM    476  HN  PHE    36        7.306   4.573  30.406  1.00
62.87            H
ATOM    477  HA  PHE    36        9.582   3.581  28.875  1.00
28.53            H
ATOM    478  HB1 PHE    36        8.209   4.346  26.953  1.00
73.56            H
ATOM    479  HB2 PHE    36        7.002   4.948  28.080  1.00
88.42            H
ATOM    480  HD1 PHE    36        5.653   3.416  29.424
1.00110.20            H
ATOM    481  HD2 PHE    36        8.565   1.943  26.629  1.00
93.07            H
ATOM    482  HE1 PHE    36        4.650   1.152  29.570
1.00155.06            H
ATOM    483  HE2 PHE    36        7.561  -0.318  26.775
1.00159.46            H
ATOM    484  HZ  PHE    36        5.604  -0.715  28.246
1.00183.25            H
ATOM    485  N   THR    37       10.648   5.735  28.073  1.00
20.06            N
ATOM    486  CA  THR    37       11.390   6.958  27.819  1.00
27.18            C
ATOM    487  C   THR    37       11.717   7.055  26.338  1.00
41.87            C
ATOM    488  O   THR    37       11.039   6.463  25.496
1.00169.86            O
ATOM    489  CB  THR    37       12.685   6.966  28.633  1.00
26.52            C
ATOM    490  CG2 THR    37       12.367   6.693  30.104  1.00
75.69            C
ATOM    491  OG1 THR    37       13.559   5.960  28.139  1.00
81.95            O
ATOM    492  HN  THR    37       10.978   4.906  27.682  1.00
18.47            H
ATOM    493  HA  THR    37       10.792   7.815  28.102  1.00
38.80            H
ATOM    494  HB  THR    37       13.161   7.930  28.546  1.00
81.60            H
ATOM    495  HG1 THR    37       14.039   5.591  28.884
1.00188.80            H
ATOM    496 HG21 THR    37       11.554   7.328  30.420
```

```
1.00196.98           H
ATOM    497 HG22 THR    37       13.240   6.899  30.705
1.00196.23           H
ATOM    498 HG23 THR    37       12.083   5.657  30.225
1.00168.92           H
ATOM    499  N   GLU    38       12.759   7.805  26.032  1.00
26.53           N
ATOM    500  CA  GLU    38       13.196   7.994  24.648  1.00
30.29           C
ATOM    501  C   GLU    38       14.708   8.180  24.582  1.00
25.39           C
ATOM    502  O   GLU    38       15.327   8.650  25.536  1.00
51.60           O
ATOM    503  CB  GLU    38       12.517   9.228  24.040  1.00
54.82           C
ATOM    504  CG  GLU    38       11.010   8.988  23.908
1.00165.84           C
ATOM    505  CD  GLU    38       10.353  10.186  23.228
1.00292.92           C
ATOM    506  OE1 GLU    38        9.165  10.113  22.963
1.00414.19           O
ATOM    507  OE2 GLU    38       11.049  11.157  22.984
1.00537.65           O1-
ATOM    508  HN  GLU    38       13.248   8.235  26.756  1.00
84.50           H
ATOM    509  HA  GLU    38       12.926   7.125  24.065  1.00
32.15           H
ATOM    510  HB1 GLU    38       12.934   9.422  23.064
1.00144.82           H
ATOM    511  HB2 GLU    38       12.688  10.082  24.679
1.00106.49           H
ATOM    512  HG1 GLU    38       10.579   8.859  24.887
1.00303.31           H
ATOM    513  HG2 GLU    38       10.836   8.100  23.318
1.00323.99           H
ATOM    514  N   CYS    39       15.296   7.821  23.445  1.00
15.84           N
ATOM    515  CA  CYS    39       16.735   7.967  23.261  1.00
14.30           C
ATOM    516  C   CYS    39       17.106   9.453  23.273  1.00
22.31           C
ATOM    517  O   CYS    39       16.298  10.294  22.879  1.00
32.92           O
ATOM    518  CB  CYS    39       17.148   7.337  21.929  1.00
18.08           C
ATOM    519  SG  CYS    39       16.030   7.908  20.625  1.00
17.41           S
ATOM    520  HN  CYS    39       14.753   7.462  22.714  1.00
24.87           H
ATOM    521  HA  CYS    39       17.241   7.455  24.065  1.00
14.60           H
ATOM    522  HB1 CYS    39       17.094   6.261  22.006  1.00
```

27.71 H
ATOM 523 HB2 CYS 39 18.158 7.630 21.689 1.00
34.91 H
ATOM 524 N PRO 40 18.293 9.804 23.715 1.00
27.27 N
ATOM 525 CA PRO 40 18.728 11.230 23.766 1.00
47.66 C
ATOM 526 C PRO 40 19.039 11.786 22.379 1.00
71.54 C
ATOM 527 O PRO 40 19.766 11.173 21.596 1.00
87.70 O
ATOM 528 CB PRO 40 19.980 11.190 24.649 1.00
53.85 C
ATOM 529 CG PRO 40 20.547 9.825 24.437 1.00
46.04 C
ATOM 530 CD PRO 40 19.346 8.899 24.207 1.00
25.70 C
ATOM 531 HA PRO 40 17.969 11.832 24.242 1.00
58.04 H
ATOM 532 HB1 PRO 40 19.713 11.322 25.687 1.00
67.93 H
ATOM 533 HB2 PRO 40 20.688 11.953 24.345 1.00
70.99 H
ATOM 534 HG1 PRO 40 21.097 9.503 25.309 1.00
65.91 H
ATOM 535 HG2 PRO 40 21.195 9.823 23.567 1.00
54.99 H
ATOM 536 HD1 PRO 40 19.038 8.439 25.134 1.00
24.22 H
ATOM 537 HD2 PRO 40 19.581 8.151 23.466 1.00
26.94 H
ATOM 538 N GLY 41 18.480 12.953 22.091
1.00105.24 N
ATOM 539 CA GLY 41 18.695 13.604 20.804
1.00147.55 C
ATOM 540 C GLY 41 20.156 14.001 20.625
1.00161.74 C
ATOM 541 O GLY 41 20.718 13.873 19.538
1.00244.37 O
ATOM 542 HN GLY 41 17.915 13.387 22.761
1.00121.79 H
ATOM 543 HA1 GLY 41 18.079 14.488 20.747
1.00184.40 H
ATOM 544 HA2 GLY 41 18.414 12.925 20.015
1.00157.34 H
ATOM 545 N LEU 42 20.761 14.493 21.704
1.00162.48 N
ATOM 546 CA LEU 42 22.162 14.923 21.677
1.00191.48 C
ATOM 547 C LEU 42 23.034 13.958 22.471
1.00166.66 C
ATOM 548 O LEU 42 22.586 13.358 23.448

```
1.00251.38            O
ATOM    549  CB  LEU    42      22.288  16.325  22.282
1.00307.99            C
ATOM    550  CG  LEU    42      21.361  17.301  21.547
1.00452.28            C
ATOM    551  CD1 LEU    42      21.467  18.682  22.202
1.00681.24            C
ATOM    552  CD2 LEU    42      21.767  17.398  20.065
1.00527.46            C
ATOM    553  HN  LEU    42      20.252  14.572  22.537
1.00195.47            H
ATOM    554  HA  LEU    42      22.517  14.952  20.657
1.00229.77            H
ATOM    555  HB1 LEU    42      23.309  16.664  22.189
1.00342.49            H
ATOM    556  HB2 LEU    42      22.016  16.289  23.327
1.00335.33            H
ATOM    557  HG  LEU    42      20.342  16.948  21.620
1.00427.79            H
ATOM    558 HD11 LEU    42      20.657  19.307  21.857
1.00909.71            H
ATOM    559 HD12 LEU    42      22.410  19.135  21.935
1.00706.80            H
ATOM    560 HD13 LEU    42      21.408  18.576  23.275
1.00858.82            H
ATOM    561 HD21 LEU    42      21.322  16.582  19.516
1.00631.69            H
ATOM    562 HD22 LEU    42      22.843  17.349  19.977
1.00628.06            H
ATOM    563 HD23 LEU    42      21.417  18.336  19.652
1.00656.75            H
ATOM    564  N   THR    43      24.286  13.818  22.048
1.00178.52            N
ATOM    565  CA  THR    43      25.218  12.927  22.729
1.00240.84            C
ATOM    566  C   THR    43      25.584  13.499  24.103
1.00372.31            C
ATOM    567  O   THR    43      25.602  14.718  24.278
1.00500.13            O
ATOM    568  CB  THR    43      26.485  12.771  21.881
1.00354.13            C
ATOM    569  CG2 THR    43      26.107  12.290  20.480
1.00463.04            C
ATOM    570  OG1 THR    43      27.148  14.024  21.789
1.00508.50            O
ATOM    571  HN  THR    43      24.590  14.324  21.265
1.00229.59            H
ATOM    572  HA  THR    43      24.752  11.964  22.845
1.00249.90            H
ATOM    573  HB  THR    43      27.143  12.050  22.337
1.00481.58            H
ATOM    574  HG1 THR    43      28.054  13.861  21.516
```

1.00624.31 H
ATOM 575 HG21 THR 43 25.434 11.449 20.558
1.00650.95 H
ATOM 576 HG22 THR 43 26.999 11.989 19.949
1.00580.73 H
ATOM 577 HG23 THR 43 25.622 13.091 19.942
1.00571.99 H
ATOM 578 N PRO 44 25.885 12.667 25.077
1.00488.39 N
ATOM 579 CA PRO 44 26.261 13.143 26.439
1.00760.13 C
ATOM 580 C PRO 44 27.695 13.663 26.468
1.00735.36 C
ATOM 581 O PRO 44 28.117 14.310 27.426
1.00999.99 O
ATOM 582 CB PRO 44 26.101 11.892 27.307
1.00999.99 C
ATOM 583 CG PRO 44 26.413 10.760 26.385
1.00856.94 C
ATOM 584 CD PRO 44 25.906 11.190 25.002
1.00548.09 C
ATOM 585 HA PRO 44 25.579 13.909 26.772
1.00939.00 H
ATOM 586 HB1 PRO 44 25.085 11.810 27.665
1.00999.99 H
ATOM 587 HB2 PRO 44 26.794 11.913 28.139
1.00999.99 H
ATOM 588 HG1 PRO 44 25.900 9.863 26.701
1.00999.99 H
ATOM 589 HG2 PRO 44 27.483 10.588 26.358
1.00853.53 H
ATOM 590 HD1 PRO 44 24.912 10.810 24.827
1.00602.45 H
ATOM 591 HD2 PRO 44 26.587 10.856 24.233
1.00463.32 H
ATOM 592 N ILE 45 28.437 13.365 25.406
1.00518.33 N
ATOM 593 CA ILE 45 29.824 13.794 25.309
1.00582.79 C
ATOM 594 C ILE 45 29.905 15.295 25.054
1.00795.93 C
ATOM 595 O ILE 45 29.267 15.815 24.138
1.00898.75 O
ATOM 596 CB ILE 45 30.521 13.044 24.172
1.00474.41 C
ATOM 597 CG1 ILE 45 30.521 11.545 24.480
1.00516.90 C
ATOM 598 CG2 ILE 45 31.964 13.537 24.042
1.00760.75 C
ATOM 599 CD1 ILE 45 30.973 10.769 23.242
1.00566.81 C
ATOM 600 HN ILE 45 28.044 12.842 24.676

```
1.00395.72            H
ATOM    601  HA  ILE    45       30.325  13.565  26.235
1.00711.06            H
ATOM    602  HB  ILE    45       29.995  13.224  23.246
1.00477.81            H
ATOM    603 HG11 ILE    45       29.523  11.233  24.753
1.00624.28            H
ATOM    604 HG12 ILE    45       31.197  11.345  25.299
1.00744.84            H
ATOM    605 HG21 ILE    45       32.430  13.547  25.016
1.00920.57            H
ATOM    606 HG22 ILE    45       31.967  14.537  23.632
1.00999.99            H
ATOM    607 HG23 ILE    45       32.512  12.878  23.387
1.00865.46            H
ATOM    608 HD11 ILE    45       30.933   9.710  23.446
1.00658.89            H
ATOM    609 HD12 ILE    45       31.986  11.050  22.992
1.00734.30            H
ATOM    610 HD13 ILE    45       30.320  11.001  22.413
1.00675.84            H
ATOM    611  N   ALA    46       30.692  15.987  25.871
1.00999.99            N
ATOM    612  CA  ALA    46       30.849  17.430  25.726
1.00999.99            C
ATOM    613  C   ALA    46       31.808  17.752  24.584
1.00999.99            C
ATOM    614  CB  ALA    46       31.382  18.029  27.028
1.00999.99            C
ATOM    615  OT1 ALA    46       31.335  17.957  23.479
1.00999.99            O
ATOM    616  OT2 ALA    46       33.002  17.788  24.832
1.00999.99            O
ATOM    617  HN  ALA    46       31.177  15.519  26.583
1.00999.99            H
ATOM    618  HA  ALA    46       29.886  17.867  25.508
1.00999.99            H
ATOM    619  HB1 ALA    46       31.698  19.047  26.853
1.00999.99            H
ATOM    620  HB2 ALA    46       32.222  17.446  27.375
1.00999.99            H
ATOM    621  HB3 ALA    46       30.601  18.019  27.775
1.00999.99            H
ENDMDL
END
```
